# Supplementary material for: Physiological reprogramming in vivo mediated by Sox4 pioneer factor activity
Source: bioRxiv. 2023 Feb 14:2023.02.14.528556. Preprint. [Version 1] doi: 10.1101/2023.02.14.528556 (PMC9948957; doi:10.1101/2023.02.14.528556)
Supplement: Supplement 2 [file media-2.pdf]

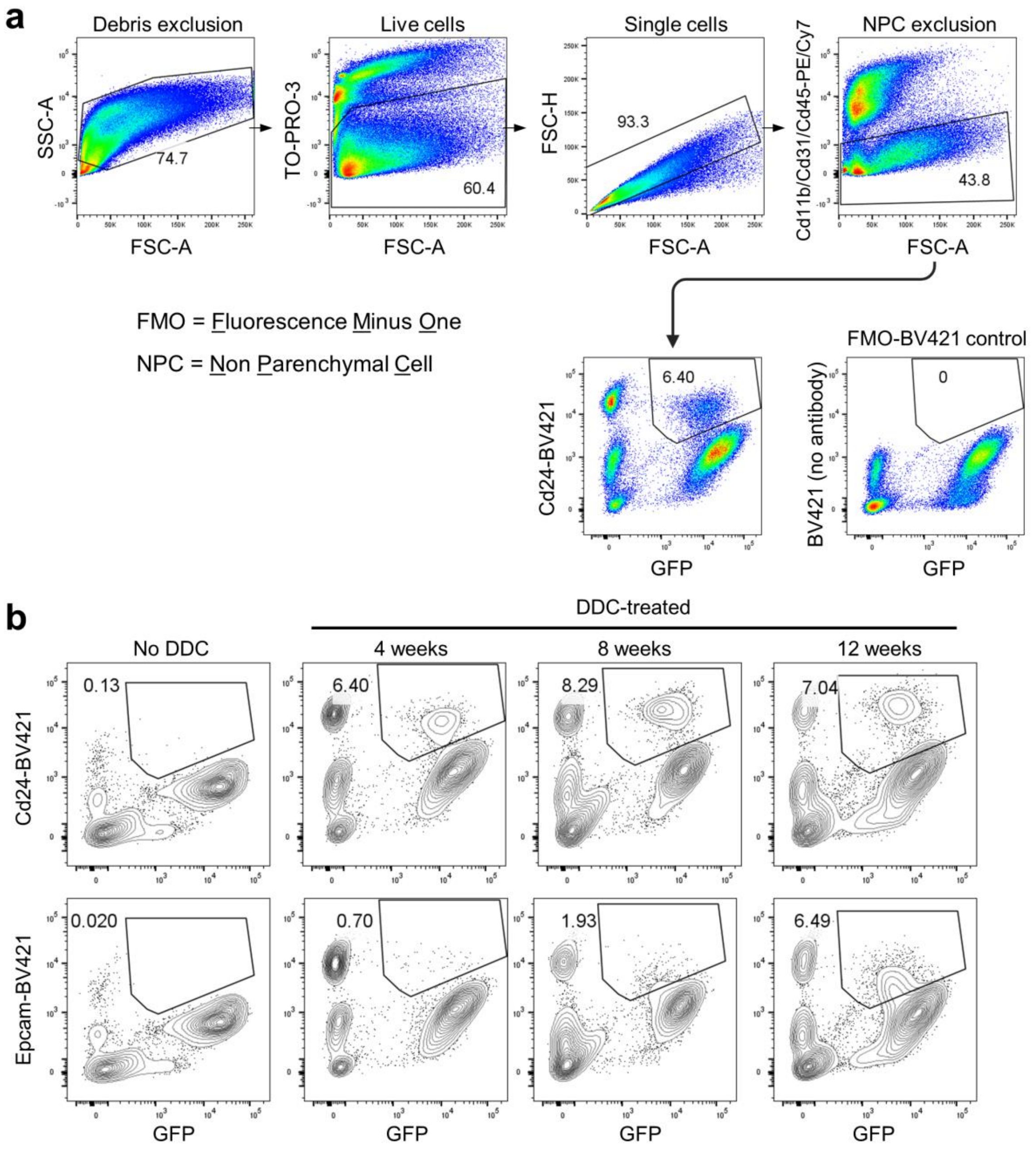

Supplementary Fig. 1. Gating strategy for flow cytometry of DDC-induced reprogrammed cells and the representative results of each time point, related to Extended Data Fig. 1c.

(a) Representative plots of gating strategy for flow cytometry of whole liver cells challenged with DDC for the designated time points (**Extended Data Fig. 1c**). Cells harvested from normal livers (No DDC) serve as the week 0 control.

(b) Representative plots indicating the reprogrammed cell fractions at the designated time points. The rectangle gates indicate the GFP/BV421 double positive cells with percentage of the total parental population. The percentages shown in **Extended Data Fig. 1c** are calculated as (GFP+/BV421+)/(GFP+) ratios.

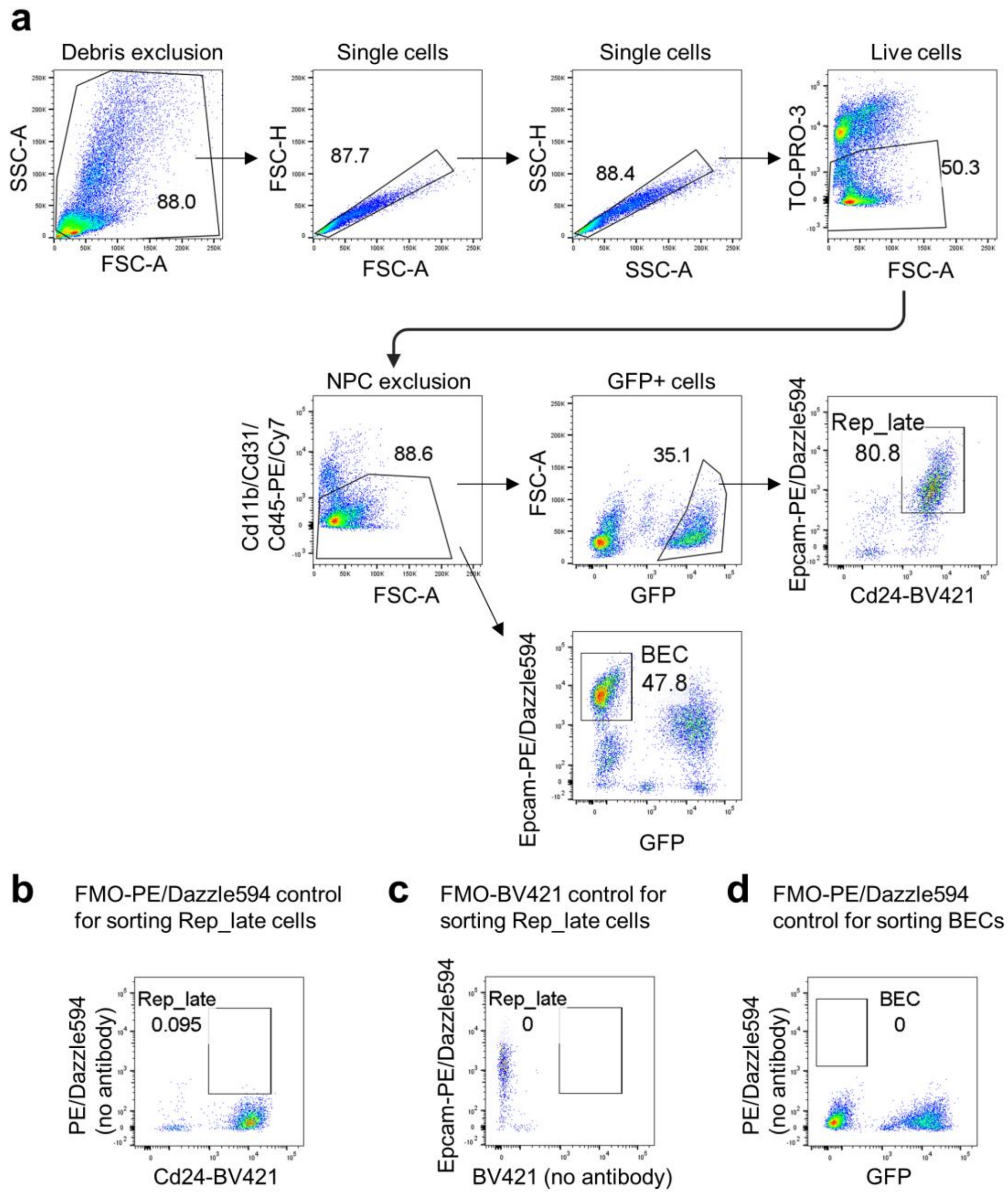

Supplementary Fig. 2. Gating strategy for FACS sorting of Rep_late and biliary epithelial cells, related to Extended Data Fig. 1e, f.

(a) Representative plots of gating strategy for FACS sorting of Rep_late and biliary epithelial cells using Epcam+ fraction which was enriched by MACS (**Extended Data Fig. 1e**).

(b) Representative plots of PE/Dazzle594-fluorescence minus one (FMO) control, which was used to determine the threshold of Epcam expression of Rep_late cells.

(c) Representative plots of BV421-FMO control, which was used to determine the threshold of Cd24 expression of Rep_late cells.

(d) Representative plots of PE/Dazzle594-FMO control, which was used to determine the threshold of Cd24 expression of biliary epithelial cells.

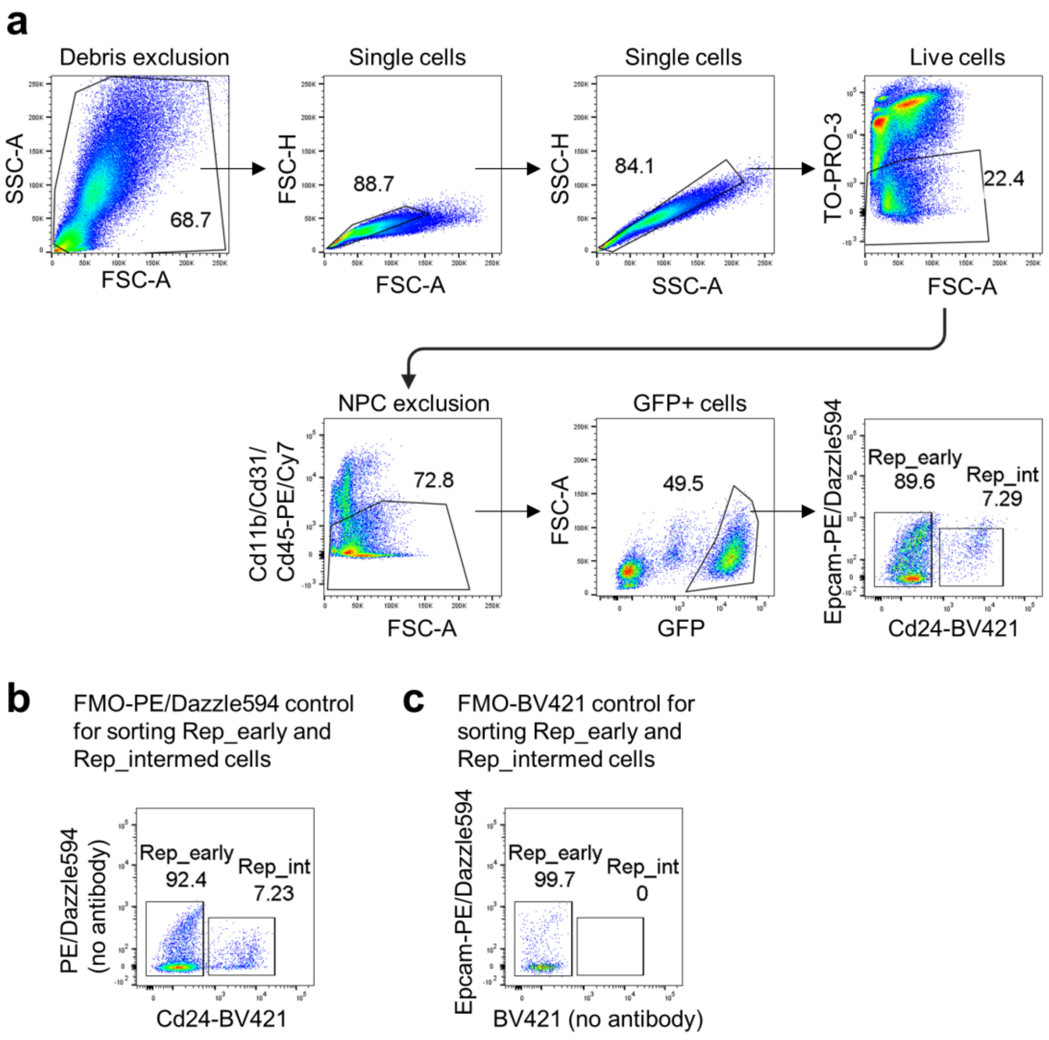

Supplementary Fig. 3. Gating strategy for FACS sorting of Rep_early and Rep_intermed cells, related to Extended Data Fig. 1e, f.

(a) Representative plots of gating strategy for FACS sorting of Rep_early and Rep_intermed cells using Epcam- fraction which was enriched by MACS (**Extended Data Fig. 1e**).

(b) Representative plots of PE/Dazzle594-FMO control, which was used to determine the threshold of Epcam expression of Rep_early and Rep_intermed cells.

(c) Representative plots of BV421-FMO control, which was used to determine the threshold of Cd24 expression of Rep_early and Rep_intermed cells.

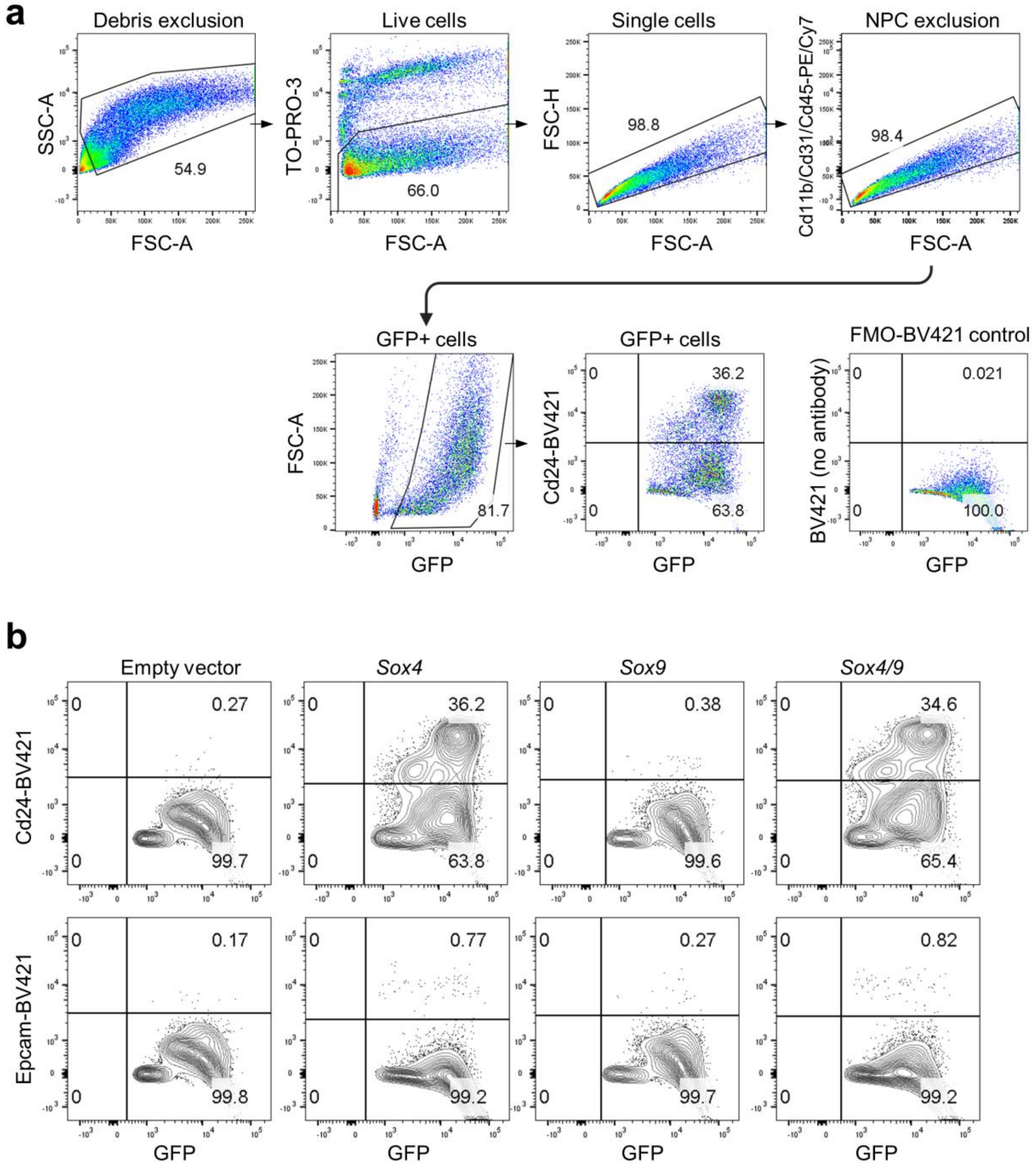

Supplementary Fig. 4. Gating strategy for determination of reprogramming efficiency of hepatocytes expressing *Sox4*, *Sox9* or both, related to Fig. 1b, Extended Data Fig. 3g.

(a) Representative plots of gating strategy for flow cytometry of whole liver cells expressing Sox4, Sox9 or both at 7 dpi (experimental design described in **Fig. 1a**). Empty vector-injected cells serve as a control.

(b) Representative plots indicating the reprogrammed cell fractions in the early (Cd24) and late (Epcam) stages. The percentages shown in the right top quadrant indicates GFP/BV421 double positive ratios based on the total GFP positive cells.

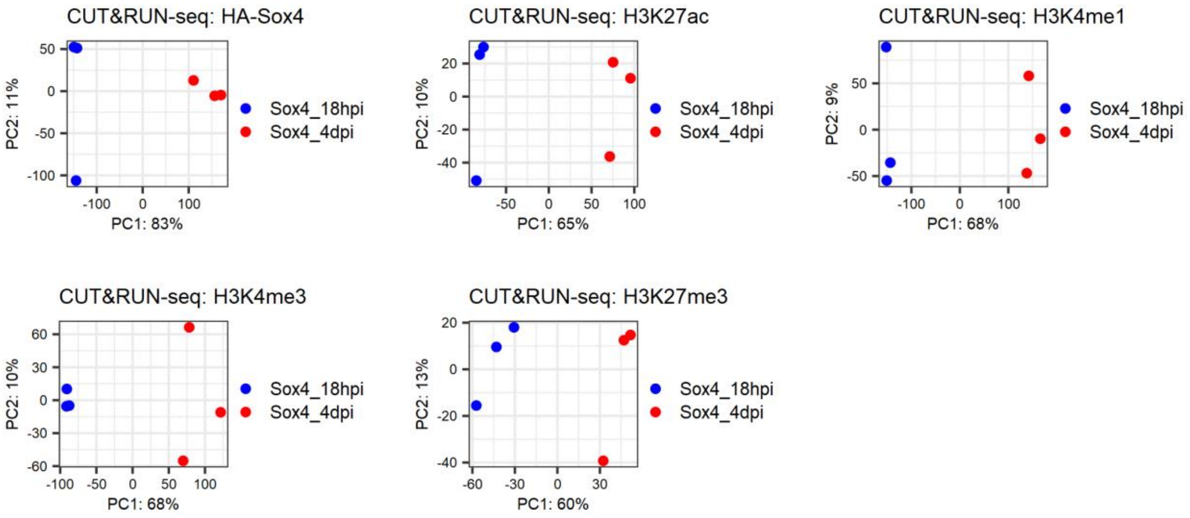

Supplementary Figure. 5. Validation of consistency in CUT&RUN-seq data among three replicates in terms of PCA mapping.

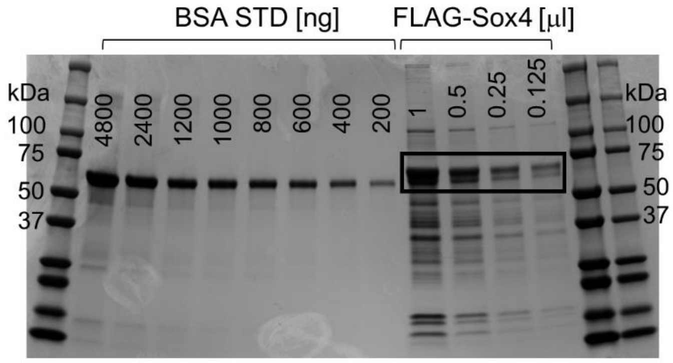

Supplementary Figure 6. Quantification of purified FLAG-Sox4 by SDS-PAGE using a BSA standard. Rectangle region corresponds to the typical Sox4 size, and used for quantification.
