# Supplementary material for: Physiological reprogramming in vivo mediated by Sox4 pioneer factor activity
Source: bioRxiv. 2023 Feb 14:2023.02.14.528556. Preprint. [Version 1] doi: 10.1101/2023.02.14.528556 (PMC9948957; doi:10.1101/2023.02.14.528556)
Supplement: Supplement 3 [file media-3.pdf]

Table S1. Differentially expressed genes between hepatocytes and early reprogrammed cells.

Upregulated genes in Rep_early v.s. Hep

Gene_id	baseMean	log2FoldChange	lfcSE	pvalue	padj
Mfge8	6902.877868	6.065226137	0.136211178	0	0
Tagln2	5569.887589	5.300897757	0.150242574	3.27E-273	2.09E-269
Nid1	3343.827712	5.7155129	0.174352408	8.72E-237	4.19E-233
Anxa2	5627.70021	7.162708988	0.237551702	6.37E-200	2.44E-196
Anxa5	16437.78852	2.385633882	0.080287084	3.16E-194	1.01E-190
Serpinb6a	1972.53765	5.36032416	0.189417256	1.25E-176	3.43E-173
Flna	12926.99246	4.106605221	0.146557837	3.31E-173	7.95E-170
Cd63	2860.789729	6.72470182	0.24159987	3.79E-171	8.08E-168
Ahnak	4276.591661	4.789840727	0.178770851	3.95E-159	7.58E-156
Spp1	104686.7999	7.040125069	0.263957036	5.64E-158	9.83E-155
Osbpl3	3492.147106	6.595340804	0.254853038	8.76E-148	1.40E-144
Dmpk	2014.690643	5.961753319	0.241753759	1.91E-135	2.82E-132
Pygb	2106.44206	4.071633822	0.168784115	1.35E-129	1.85E-126
Cystm1	913.1600743	3.198601854	0.134338378	3.73E-126	4.77E-123
Emp2	1701.891287	3.553394165	0.15337867	1.57E-119	1.89E-116
Fads3	1438.431683	2.858082654	0.124984406	3.19E-116	3.60E-113
Bicc1	7310.942721	5.707049078	0.250390487	5.86E-115	6.25E-112
S100a11	2499.42336	6.44429242	0.285647586	1.32E-112	1.27E-109
Cstb	2245.495281	2.695056312	0.120281901	3.81E-111	3.48E-108
Ubd	999.3194965	7.071871326	0.327565344	2.35E-103	1.96E-100
Lama5	7209.224019	6.125436108	0.285874083	2.03E-102	1.62E-99
Ddr1	4937.617359	5.842408139	0.278226491	6.95E-98	5.13E-95
Ly6d	1741.198405	6.245572843	0.302363885	8.25E-96	5.86E-93
Abcc4	1273.192084	6.039638554	0.303929939	1.82E-88	1.13E-85
Npdc1	1128.275668	4.460223271	0.225142566	3.37E-88	2.02E-85
Iqgap1	3693.490198	3.518485929	0.17764993	3.95E-88	2.30E-85
Prelp	1636.616549	5.253405862	0.264935555	4.83E-88	2.72E-85
Cybb	849.2809416	6.131587285	0.311438476	4.42E-87	2.43E-84
Krt18	21172.22313	2.281766701	0.116067439	6.20E-87	3.30E-84
Dusp4	769.6146092	3.905223968	0.200453407	5.03E-85	2.61E-82
Entpd2	539.9128162	3.064079829	0.158924728	1.43E-83	7.02E-81
Unc13b	1376.866657	3.346520376	0.174792871	1.19E-82	5.71E-80
Pam	1955.879668	4.265502789	0.222306528	2.77E-82	1.30E-79
Steap2	1723.323568	3.255639626	0.172812814	4.13E-80	1.89E-77
Golm1	1057.67312	3.412252829	0.18159298	1.40E-79	6.10E-77
Rragd	3357.50487	8.533547168	0.449484709	3.86E-79	1.64E-76
Lamc1	1937.260399	2.405785597	0.130429419	7.81E-77	3.26E-74
Smpd3	1931.635961	3.219283797	0.178156907	5.54E-74	2.21E-71
Mtmr11	638.9640192	2.711456442	0.149776292	5.75E-74	2.25E-71

Tpm1	3669.779031	1.969787971	0.109380433	1.95E-73	7.49E-71
Pak6	1667.587799	6.796154113	0.376401661	5.30E-72	1.99E-69
Pitpnm1	915.804418	3.49999021	0.199764463	1.59E-69	5.75E-67
Sh3pxd2b	1590.483526	4.097549805	0.235924959	3.41E-68	1.21E-65
Trim47	1675.333833	4.766001389	0.275350312	9.91E-68	3.40E-65
Rhbdf1	1328.09275	2.415335991	0.139989623	1.50E-67	4.98E-65
Krt8	33827.50579	2.421114881	0.140276161	1.85E-67	6.00E-65
Ets2	3247.801541	2.476794935	0.144702216	1.02E-66	3.28E-64
Epb41l1	1148.814833	6.10598282	0.354674979	5.77E-66	1.82E-63
Itga6	830.2633331	2.929508393	0.172405414	2.04E-65	6.31E-63
Vcam1	4509.077815	4.452857942	0.263563753	1.17E-64	3.50E-62
Gsta1	510.5820908	4.384058327	0.263775244	3.56E-63	9.76E-61
Ifi27l2b	869.8349867	2.833053728	0.172834823	1.66E-61	4.09E-59
Rras	1192.43813	1.956790621	0.119527855	5.13E-61	1.25E-58
Slc43a2	4679.899199	2.927934201	0.179091129	8.12E-61	1.95E-58
Myl12a	2480.199164	1.582518612	0.098195289	4.35E-59	1.02E-56
Tgfbr2	1823.698814	1.729527716	0.107950139	1.73E-58	4.00E-56
Itpr3	1859.686378	6.32988895	0.391653212	3.79E-58	8.66E-56
Agrn	18322.92867	1.354391997	0.084964472	9.15E-58	2.06E-55
Cav2	808.5762741	3.934850365	0.247073595	9.25E-58	2.06E-55
Cd9	3123.118331	3.227552883	0.203971503	1.90E-57	4.14E-55
Arrb1	1004.049177	4.435945078	0.278474054	1.88E-57	4.14E-55
Sprr1a	552.0048189	7.873098531	0.49176401	4.70E-57	1.01E-54
Myo1c	2795.299184	1.643693798	0.10421127	7.70E-57	1.64E-54
Abcc5	1363.308835	4.307275423	0.272732737	9.72E-57	2.05E-54
Pkd2	2618.315516	1.299077417	0.082403876	2.31E-56	4.71E-54
Tm4sf4	12492.0944	2.254533345	0.143880083	3.10E-56	6.26E-54
Uap1l1	1267.481248	3.230985037	0.206471359	4.39E-56	8.78E-54
F11r	6594.67141	1.065004901	0.06785749	5.20E-56	1.02E-53
Slpi	513.3845618	7.841733221	0.495750681	8.04E-56	1.56E-53
Ccdc120	701.478924	2.7353855	0.175348676	1.02E-55	1.96E-53
Chmp4c	1792.032984	2.57072206	0.165199459	1.91E-55	3.60E-53
Specc1	764.9099083	2.560918827	0.164213717	2.02E-55	3.76E-53
Gm3776	443.2839314	4.882928116	0.316917903	9.28E-55	1.71E-52
Plet1	2059.923758	4.378116434	0.284634133	1.71E-54	3.09E-52
Cbr3	476.737001	6.886760472	0.444770273	2.34E-54	4.20E-52
Myo7a	1894.705031	2.466126278	0.160222232	2.47E-54	4.38E-52
Ezr	2894.776709	2.87829062	0.188868377	2.30E-53	4.06E-51
Cdc42se1	1443.370978	1.835668448	0.12036433	2.98E-53	5.20E-51
Lgals1	1864.264472	3.175279343	0.210624093	1.82E-52	3.11E-50
Pakap	831.1717092	3.800837076	0.251035397	1.99E-52	3.38E-50
Slc25a24	2130.409856	8.482650468	0.549972335	2.85E-52	4.76E-50
Fmo2	1337.029237	4.824042454	0.322467263	1.36E-51	2.21E-49

Tmsb10	3903.761089	6.517295322	0.440652378	1.17E-49	1.87E-47
Tes	1160.343998	3.126335362	0.212722772	1.19E-49	1.89E-47
Rhoc	1087.529876	3.559151763	0.243687322	2.41E-49	3.77E-47
Rgs5	21587.48284	4.769915975	0.325203791	2.63E-49	4.06E-47
Clcf1	3063.556035	5.499211505	0.375927555	4.71E-49	7.17E-47
Rab3d	893.8767309	1.757507478	0.120002228	5.80E-49	8.77E-47
Nck2	675.8478451	3.586077168	0.247280689	4.34E-48	6.45E-46
Abca5	846.7686621	1.859706203	0.129034818	7.14E-48	1.05E-45
Plxna3	616.2926403	4.664045333	0.322244045	1.68E-47	2.46E-45
Mapk3	2336.977053	1.35331405	0.094069505	1.81E-47	2.64E-45
Alpk1	590.6005021	3.490807743	0.244233093	6.67E-47	9.47E-45
Fkbp1a	2034.011696	1.141112239	0.079713842	6.77E-47	9.56E-45
Gipc2	518.4771754	7.929229395	0.545405083	1.22E-46	1.70E-44
Rtn4	8745.166298	1.663808646	0.117022187	1.50E-46	2.08E-44
Id1	742.5083659	4.768532588	0.337546186	1.85E-46	2.54E-44
Klc1	939.6043412	1.453668533	0.102702742	4.93E-46	6.61E-44
Tnfrsf12a	1602.336135	3.313808985	0.235692302	5.60E-46	7.46E-44
Unc5b	1262.892053	4.190946346	0.297046843	6.17E-46	8.11E-44
Vim	1716.491899	4.364972921	0.308692278	7.14E-46	9.32E-44
Haus8	512.4162491	3.189711041	0.226988403	7.50E-46	9.73E-44
Tax1bp3	722.2085877	2.283144067	0.162700463	1.69E-45	2.18E-43
Ano6	1739.712585	2.174876743	0.154955357	2.23E-45	2.85E-43
Isyna1	933.0269572	2.20292636	0.157253905	2.55E-45	3.24E-43
Lgals3	560.0719328	8.660884979	0.60497043	2.73E-45	3.44E-43
Rcan2	244.3838137	3.558365153	0.255039958	3.93E-45	4.90E-43
Maoa	483.5631152	2.099010841	0.150783447	7.58E-45	9.21E-43
Spats2l	390.0388806	4.905438452	0.351016692	2.74E-44	3.23E-42
Bmp6	1444.196603	7.760724324	0.553839089	4.82E-44	5.60E-42
Arhgap27	584.3936866	4.89693387	0.353199544	5.20E-44	5.98E-42
Card10	3561.730514	2.165259751	0.157795172	1.03E-43	1.17E-41
Lpl	1437.187349	3.226958871	0.23610241	1.57E-43	1.76E-41
Vill	1270.715557	5.545270807	0.401338377	3.20E-43	3.57E-41
App	11500.26785	1.941963033	0.143049308	7.47E-43	8.14E-41
Csf1	2009.083438	3.34209467	0.247487286	1.23E-42	1.34E-40
Flt4	577.5153147	3.576803115	0.265414499	2.61E-42	2.77E-40
Tmem176b	12340.78038	1.430152547	0.105844708	3.29E-42	3.45E-40
Pdlim7	626.095726	2.313363721	0.17133085	3.35E-42	3.50E-40
Crtap	533.1823875	2.623322816	0.195054637	5.80E-42	5.95E-40
Arpc1b	2020.229584	2.074927896	0.154667044	6.65E-42	6.75E-40
Nfkbiz	2187.735202	2.593001157	0.195162358	2.24E-41	2.25E-39
Alpl	901.7863714	1.65785534	0.125012645	8.90E-41	8.89E-39
Rere	2387.41737	1.181615955	0.088935879	1.05E-40	1.04E-38
Slc25a4	1174.065257	5.018113948	0.380332792	1.79E-40	1.73E-38

Mvp	3555.029373	2.550804481	0.194824204	3.79E-40	3.65E-38
Igsf8	870.8610881	2.366784767	0.180538664	6.26E-40	6.01E-38
Arhgap44	513.1554464	2.774073505	0.211867739	6.99E-40	6.64E-38
Sptan1	8904.858141	1.067579888	0.081280901	7.70E-40	7.28E-38
Tmem176a	7915.062951	1.281894443	0.098207905	1.58E-39	1.47E-37
Synpo	2028.95591	2.415900575	0.186481128	2.57E-39	2.37E-37
Miga1	644.6403984	2.776972734	0.214605272	5.58E-39	5.12E-37
Rab34	937.8339107	2.199872603	0.170866369	1.40E-38	1.28E-36
Src	1239.87662	2.261548031	0.176239468	1.81E-38	1.64E-36
Tcaf1	653.8295039	2.628778477	0.205245143	3.26E-38	2.94E-36
Ankrd10	1032.873478	1.838440966	0.143854252	4.03E-38	3.60E-36
Serpine1	310.4900576	5.338339815	0.419646556	6.42E-38	5.67E-36
Pak1	808.1342862	2.848483587	0.224014514	9.20E-38	8.03E-36
Gls	777.5880481	2.868965432	0.226553971	1.13E-37	9.75E-36
Stmn1	794.0286794	2.986853977	0.23596896	1.13E-37	9.75E-36
Plin4	464.4996555	4.865574617	0.385960264	1.41E-37	1.21E-35
Anxa4	5265.124635	1.064128906	0.083836898	2.61E-37	2.21E-35
Rbbp8	559.8969069	2.177598556	0.172964754	3.66E-37	3.07E-35
Abcb1a	580.5758382	3.337000092	0.267716311	1.00E-36	8.31E-35
Scn1b	411.1688752	3.07038867	0.247620674	2.93E-36	2.33E-34
Sh3bp4	768.7533521	2.627110222	0.211164951	3.78E-36	3.00E-34
H2-Q1	166.7637522	4.036511237	0.326807556	3.93E-36	3.10E-34
Adamts7	987.7749654	2.387136401	0.193789322	8.29E-36	6.41E-34
Epb41l2	992.8211022	2.727838102	0.220305086	1.06E-35	8.17E-34
Nedd9	1243.650118	2.354471723	0.191081015	1.10E-35	8.41E-34
Adam11	957.3567386	2.472978795	0.203073	5.08E-35	3.84E-33
Col4a1	3848.690724	2.598377989	0.215274437	1.53E-34	1.14E-32
Aldoa	4625.542072	1.096374005	0.090270359	2.25E-34	1.67E-32
Klhdc8b	721.2995738	1.968671887	0.162985528	2.27E-34	1.67E-32
Map4k4	2315.772347	2.704978691	0.224434	2.42E-34	1.78E-32
Lrtm2	254.542963	6.209269919	0.513525314	2.57E-34	1.88E-32
Ppl	4769.684527	3.499315563	0.292544153	4.25E-34	3.09E-32
Cavin2	553.8028122	2.897721863	0.240800824	5.17E-34	3.73E-32
Pea15a	1016.954419	2.088163857	0.174333388	7.19E-34	5.17E-32
Lysmd2	189.4115612	3.138069751	0.264448395	2.50E-33	1.76E-31
Fhod1	756.0451718	2.694196099	0.227648404	5.00E-33	3.49E-31
Maged2	781.4167773	1.73423385	0.146505414	5.81E-33	4.03E-31
Phldb1	1087.579966	2.600419711	0.219823103	7.31E-33	5.02E-31
1700017B05Rik	1170.847373	1.349801579	0.114172266	8.00E-33	5.48E-31
Ptgr1	622.257811	2.007667132	0.170338596	8.32E-33	5.68E-31
Tmem200b	172.8857394	3.165547408	0.270147316	1.47E-32	9.97E-31
St14	1708.65254	6.203218893	0.521202728	1.70E-32	1.15E-30
Btg3	340.3712981	2.110539816	0.180646174	2.47E-32	1.66E-30

B4galt5	1641.815408	1.697711819	0.145164392	2.69E-32	1.80E-30
Pcolce	380.9320447	2.339844832	0.200186498	2.87E-32	1.90E-30
Amdhd2	922.0458705	1.46192043	0.125340278	4.86E-32	3.21E-30
Mcm6	958.1815709	2.726722362	0.236289665	9.40E-32	6.14E-30
Tbc1d24	1001.858609	1.226433491	0.105501113	9.93E-32	6.46E-30
Ankrd13b	647.7782181	1.936814313	0.167185761	1.05E-31	6.79E-30
Arhgef16	1015.504197	1.183540664	0.101941558	1.46E-31	9.39E-30
Them6	404.6603918	1.872701574	0.162873261	2.35E-31	1.50E-29
Nrg1	434.418342	3.760777178	0.330709076	5.14E-31	3.23E-29
Sirpa	1056.623764	2.014559228	0.176645922	8.94E-31	5.54E-29
Klf6	3236.996363	2.931819051	0.259353832	1.11E-30	6.87E-29
Osbpl7	397.1441368	3.242965085	0.284589123	1.19E-30	7.33E-29
Galns	510.338008	1.87579659	0.165419099	1.41E-30	8.59E-29
Nagk	612.1433348	1.863566791	0.164630223	1.84E-30	1.11E-28
Gltp	363.8767919	3.31169326	0.292703977	2.46E-30	1.47E-28
Smoc2	255.4244332	2.492784852	0.221829867	3.26E-30	1.94E-28
Cavin1	1055.494728	2.877739709	0.254545549	3.33E-30	1.97E-28
Cxcl16	1465.568382	3.230345683	0.287174911	3.45E-30	2.03E-28
Cul7	1027.888616	1.441612402	0.127745022	4.44E-30	2.59E-28
Wnk4	352.5355701	2.753561244	0.245218663	4.63E-30	2.69E-28
Chrm3	305.1106829	2.9719887	0.264553574	5.37E-30	3.10E-28
Cul9	988.5653489	1.50595628	0.13407224	6.55E-30	3.76E-28
Lamb2	2218.309935	5.284396506	0.463143935	6.88E-30	3.93E-28
Espn	100.1849283	4.141020633	0.367439205	7.75E-30	4.42E-28
Ankrd27	1384.88522	1.044874906	0.092851758	9.43E-30	5.34E-28
Sox4	6011.501005	7.541602947	0.656659124	1.35E-29	7.59E-28
Hook2	1043.872935	1.45946583	0.130809238	1.63E-29	9.12E-28
St3gal2	772.6672223	3.483148869	0.311650773	1.75E-29	9.79E-28
Rab31	697.802115	4.538106139	0.407706248	2.61E-29	1.45E-27
Mthfd1l	302.4856827	2.477359287	0.22401344	3.04E-29	1.68E-27
Slc20a1	1066.056153	2.218991498	0.201089422	3.52E-29	1.93E-27
Rap1gap	1801.710336	1.693510931	0.15296843	3.55E-29	1.94E-27
Plxnd1	1309.856131	2.73995077	0.248387086	3.88E-29	2.12E-27
Olfm2	234.8928339	2.999580844	0.272766885	4.66E-29	2.53E-27
Ppp1r9b	1239.144456	3.066432383	0.277055515	4.85E-29	2.63E-27
Bcam	2672.586742	3.647534805	0.328783173	4.97E-29	2.69E-27
Large1	622.4581527	1.891168589	0.171639438	5.24E-29	2.83E-27
Abcc12	98.95334943	3.944793818	0.358759024	5.35E-29	2.87E-27
Cpe	3309.694741	14.11836314	2.496479642	5.81E-29	3.08E-27
Serpina7	1702.527086	3.449701963	0.315735902	6.29E-29	3.33E-27
Gata6	1445.944863	1.479491087	0.134270128	7.72E-29	4.07E-27
Pld2	760.4973709	1.669087884	0.151745915	8.46E-29	4.45E-27
Sorl1	796.2105098	2.866406362	0.26164578	9.16E-29	4.79E-27

Nfkb2	1428.58115	2.186380285	0.200435645	1.41E-28	7.32E-27
Renbp	183.3495117	4.841444901	0.44240731	1.75E-28	9.01E-27
Gstm5	824.4358722	1.599790126	0.146221103	1.87E-28	9.60E-27
Cd44	5217.688761	5.853114876	0.537569429	1.88E-28	9.61E-27
Ptpn21	1813.38248	1.261775269	0.115330554	2.22E-28	1.13E-26
Frmd6	1107.315075	2.086454107	0.191654133	2.46E-28	1.25E-26
Dennd11	778.6740688	1.332412419	0.122407369	3.82E-28	1.92E-26
Stk10	198.7867947	2.608680636	0.241345208	4.08E-28	2.04E-26
Tnfaip3	884.3747103	2.198027528	0.203187873	4.34E-28	2.16E-26
Usp20	584.2091082	1.686670957	0.155878044	5.74E-28	2.85E-26
Atpif1	624.1112407	1.636047827	0.151269622	7.21E-28	3.56E-26
Aldh3b1	313.3033028	2.382088236	0.221000269	8.75E-28	4.29E-26
Rasal1	1177.640221	7.839031057	0.700945993	1.12E-27	5.49E-26
Mgat4b	2191.490491	1.726584121	0.161090059	1.89E-27	9.09E-26
Flot1	1403.780142	1.738779532	0.162566765	2.00E-27	9.57E-26
Ptprs	1970.867571	1.890543076	0.17662053	2.07E-27	9.90E-26
Parp3	1163.188464	1.052847382	0.098028661	2.67E-27	1.27E-25
Gm20559	371.8978432	2.929219903	0.27444	3.50E-27	1.65E-25
Mcm2	443.1561198	2.590796837	0.243541365	3.53E-27	1.66E-25
Map3k1	3685.649888	1.529283646	0.143008937	5.99E-27	2.80E-25
Acot9	396.9432547	2.753169934	0.260926837	6.10E-27	2.84E-25
Itpripl2	712.9640184	4.377999239	0.41312416	6.32E-27	2.94E-25
Fam102b	539.5275745	6.097952841	0.566588207	1.05E-26	4.85E-25
Lcn2	9438.525141	4.258323722	0.409637172	1.17E-26	5.40E-25
Epn2	669.5473783	1.769305152	0.168247832	1.42E-26	6.48E-25
Sestd1	1132.164538	2.585101138	0.246541438	1.67E-26	7.60E-25
Arhgdia	4321.844229	1.142602011	0.108496148	1.79E-26	8.10E-25
Slc5a6	367.1006678	1.660326891	0.15823807	1.88E-26	8.49E-25
Dtx3	837.0699689	2.471236291	0.236711702	2.12E-26	9.55E-25
Rad51b	414.2208351	5.54381123	0.521719019	2.25E-26	1.01E-24
Tmem106a	256.2543295	1.942280267	0.185852239	2.55E-26	1.14E-24
Sgce	311.2840929	2.476255068	0.23735701	2.84E-26	1.27E-24
Lpar2	298.2543136	3.217060493	0.307304524	3.31E-26	1.47E-24
Trip10	650.5203082	1.670836826	0.160426722	4.75E-26	2.10E-24
Clip2	531.2050821	2.91459061	0.28267509	6.94E-26	3.04E-24
Kctd17	347.6478245	2.276255469	0.219738524	6.92E-26	3.04E-24
Ppp1r18	407.6433091	4.450850917	0.426988059	7.41E-26	3.23E-24
Gss	2150.458779	1.089322957	0.104383037	8.41E-26	3.64E-24
Mbp	759.4911749	4.925468889	0.470011399	1.16E-25	5.03E-24
Rai1	1483.517215	1.132202026	0.109099461	1.24E-25	5.33E-24
Cd36	1639.383397	2.34095487	0.228477291	1.43E-25	6.12E-24
Ctps	712.0956619	1.659065908	0.161210377	1.68E-25	7.11E-24
Tmc6	564.392668	1.59500117	0.155043791	1.78E-25	7.52E-24

Ccdc88c	847.9117176	3.62146795	0.350830778	1.90E-25	7.99E-24
Ier3	1501.956101	5.786743638	0.558972517	2.11E-25	8.82E-24
Cln6	255.0913744	3.353779228	0.329637397	2.34E-25	9.78E-24
Efhd2	2328.641903	1.653273095	0.161371391	2.60E-25	1.08E-23
Ywhah	2545.35393	1.256148445	0.122391085	3.35E-25	1.38E-23
Pip4k2a	149.09379	4.18435387	0.40637381	3.37E-25	1.39E-23
Crim1	956.6138562	1.240846181	0.121008723	3.64E-25	1.49E-23
Myo9b	3108.436524	1.339131184	0.131077232	4.55E-25	1.86E-23
Map3k14	444.9455037	2.20341747	0.216496263	4.84E-25	1.97E-23
Pawr	1259.389142	1.776298506	0.175042291	5.37E-25	2.18E-23
Plpp2	1312.809125	1.766536982	0.174319988	7.04E-25	2.83E-23
Epdr1	380.2303894	2.588141523	0.255375465	7.63E-25	3.06E-23
Ahnak2	1008.818222	12.92373979	2.502389971	8.20E-25	3.28E-23
Gal3st1	171.7064654	3.470761588	0.343109497	9.22E-25	3.68E-23
Als2cl	2160.895612	1.387127881	0.137197139	1.10E-24	4.39E-23
Septin6	240.672614	3.445001111	0.340123648	1.49E-24	5.90E-23
Cgref1	117.1709408	3.12736073	0.309239645	1.49E-24	5.90E-23
Cd14	1958.505159	6.13513578	0.619672604	2.55E-24	9.94E-23
Ajuba	1944.842221	2.023153766	0.202696518	2.58E-24	1.00E-22
Pkmyt1	249.9166312	3.872474487	0.38522058	2.91E-24	1.13E-22
Sstr2	166.7671628	3.125101211	0.313632421	2.92E-24	1.13E-22
Chpf	458.4690335	4.026061561	0.399408367	3.21E-24	1.24E-22
Aldh18a1	365.8207181	3.170255093	0.317836464	3.58E-24	1.38E-22
Sh2d4a	967.132892	1.241274358	0.12391401	4.12E-24	1.57E-22
Tead1	1920.464134	1.492962968	0.150009209	6.54E-24	2.48E-22
Scamp5	838.6320103	1.095167426	0.109741122	7.75E-24	2.93E-22
Mmp14	4077.948657	1.486992406	0.15047288	1.08E-23	4.04E-22
Prr15l	824.9309506	6.004080608	0.592554657	1.15E-23	4.29E-22
Rtkn	415.7613345	1.726990929	0.175022595	1.24E-23	4.61E-22
Zmynd15	465.3682816	3.31301414	0.33582341	1.25E-23	4.64E-22
Cpeb1	455.0611438	2.66780218	0.272568397	1.35E-23	4.97E-22
Clu	256025.3423	1.347028038	0.136283725	1.38E-23	5.09E-22
Hdac7	1556.245244	4.024136923	0.408504548	1.55E-23	5.70E-22
H19	160.3576547	7.994986	0.794896445	1.68E-23	6.16E-22
Slc6a8	1337.444188	1.608487242	0.163718116	2.09E-23	7.62E-22
Pgd	2587.353257	1.467658954	0.149643188	2.45E-23	8.88E-22
Spaca6	362.1219917	3.352226337	0.343799782	2.60E-23	9.40E-22
Trip6	957.7624527	1.273577305	0.130349684	3.42E-23	1.23E-21
Pafah1b3	461.3010025	2.356397054	0.241845088	3.56E-23	1.28E-21
Epha1	966.5023088	1.083896703	0.110661004	3.77E-23	1.34E-21
Maff	732.9096626	2.493058284	0.257037216	3.76E-23	1.34E-21
Gm11843	224.6431216	3.417844363	0.353864655	4.04E-23	1.44E-21
Rcn1	668.736671	4.230281686	0.428845171	4.44E-23	1.57E-21

Pfkp	497.9061665	4.634677078	0.475523031	4.55E-23	1.60E-21
Arsa	1561.34212	1.37777063	0.141187316	4.64E-23	1.63E-21
Il3ra	300.73524	3.637347603	0.372771696	4.78E-23	1.67E-21
Numbl	253.1200083	4.858491272	0.491482438	5.42E-23	1.89E-21
Pdgfb	1215.488821	5.185749978	0.52466783	6.21E-23	2.16E-21
Ssbp4	537.9822375	3.564507303	0.368848017	6.61E-23	2.30E-21
Mapk7	311.1175863	2.903077475	0.299707351	7.66E-23	2.66E-21
Lynx1	827.3898248	3.105128967	0.320365456	8.82E-23	3.05E-21
Foxp4	2932.029124	1.151870181	0.118905114	1.21E-22	4.19E-21
Gsta2	1064.69359	1.986691795	0.207499922	1.59E-22	5.44E-21
Elovl7	741.0674179	8.09463339	0.804774181	2.12E-22	7.22E-21
Plekhg2	926.1263878	1.57372618	0.164356991	2.31E-22	7.86E-21
Vcl	1443.456179	1.005088541	0.104345627	2.57E-22	8.70E-21
Fbf1	763.0083194	1.573624386	0.164905234	3.41E-22	1.14E-20
Abhd2	9356.064568	1.068066736	0.111376937	3.92E-22	1.31E-20
Micall2	254.2785188	3.435031812	0.358469558	4.18E-22	1.40E-20
Efemp1	1823.420415	4.752561098	0.492800016	5.47E-22	1.82E-20
Galnt10	665.5387705	1.119192041	0.117403734	5.89E-22	1.96E-20
Tspan17	229.8664152	3.067068087	0.325027156	6.16E-22	2.04E-20
Amot	1461.64766	1.202611047	0.126710513	7.28E-22	2.40E-20
Msrb3	486.8743528	1.192098674	0.125464685	7.42E-22	2.44E-20
Ccn1	15724.42294	3.034057867	0.3247315	8.55E-22	2.80E-20
Pqlc3	189.9758091	3.835245686	0.408993301	1.50E-21	4.86E-20
Rarg	572.9018362	2.183394641	0.233951344	1.50E-21	4.86E-20
Ttc39a	267.6521604	2.202105938	0.235359508	1.51E-21	4.86E-20
Tpm4	2393.122075	1.720202672	0.184003563	1.65E-21	5.32E-20
Synj2	884.6744228	2.071044236	0.221934015	1.68E-21	5.41E-20
Vopp1	335.0683253	1.750613213	0.186997586	1.99E-21	6.39E-20
Smad7	783.8921838	1.773309471	0.190536926	2.61E-21	8.29E-20
Rasa2	307.3882813	1.825850499	0.19716163	5.10E-21	1.59E-19
Shc2	1090.548853	9.387457823	0.943881129	5.45E-21	1.69E-19
Spry1	453.7389473	4.097251714	0.446173455	8.19E-21	2.51E-19
Vars	2957.296763	1.282576065	0.139653887	1.25E-20	3.78E-19
Sall1	1215.771741	1.077904565	0.117269692	1.33E-20	4.02E-19
Vat1	2251.366067	1.566495578	0.171605204	1.53E-20	4.61E-19
Ypel4	147.3591434	5.447297918	0.592308986	1.68E-20	5.02E-19
Jag1	2747.116342	2.145327946	0.235969225	1.71E-20	5.12E-19
Cd68	66.96384398	3.577859007	0.394519638	1.73E-20	5.15E-19
Casp12	818.8026255	7.780384341	0.817313033	2.45E-20	7.20E-19
Mgst3	503.0049499	1.765362149	0.195095197	2.74E-20	8.05E-19
Pgm2	331.3203627	1.480459325	0.163203587	3.08E-20	9.01E-19
Myo15b	651.916575	6.549127312	0.694702291	3.39E-20	9.89E-19
Ugdh	5793.248318	1.156439417	0.127142443	3.82E-20	1.11E-18

Tgfb2	1009.420013	6.585584989	0.701378901	4.02E-20	1.16E-18
Mapt	70.6686372	5.407855072	0.590224047	4.17E-20	1.20E-18
Zfp704	1516.746185	1.591581278	0.176351649	4.62E-20	1.33E-18
Mapk8ip3	1372.431787	1.229733391	0.13604765	5.08E-20	1.45E-18
Agfg1	1702.21483	1.05120464	0.116032068	5.30E-20	1.52E-18
Adamts1	1448.208397	1.652299992	0.184740049	7.47E-20	2.11E-18
Syt12	261.8848708	3.240875284	0.365354021	7.86E-20	2.21E-18
Hkdc1	820.3382632	11.58168327	2.444787278	9.86E-20	2.75E-18
Rrs1	600.3031398	1.391446468	0.155724025	1.09E-19	3.02E-18
Vsig10	1193.512276	2.377438714	0.267838541	1.16E-19	3.21E-18
Zc2hc1a	370.0259412	3.591245472	0.400648149	1.20E-19	3.32E-18
Akr1b3	295.984315	1.576617875	0.177225425	1.30E-19	3.59E-18
Ubqln2	1997.223593	1.133147721	0.126699218	1.36E-19	3.74E-18
Cachd1	202.4523962	6.909570749	0.755197715	1.51E-19	4.14E-18
Sertad1	208.1011079	1.929759632	0.217910256	1.60E-19	4.38E-18
Gm30692	59.74695032	5.467056215	0.609375502	1.61E-19	4.39E-18
Col1a1	287.0758492	7.08515035	0.797604679	1.65E-19	4.50E-18
Lrrc8b	514.4137737	2.853441122	0.322772026	1.89E-19	5.11E-18
Arl6ip5	1454.711163	1.092037525	0.123242007	3.17E-19	8.50E-18
Pacs1	485.1246709	1.858731294	0.21149142	3.20E-19	8.57E-18
Tlnrd1	768.2721929	1.962854899	0.224062787	3.52E-19	9.41E-18
Adamtsl4	372.6859035	4.572801291	0.521882131	4.78E-19	1.27E-17
Kifc3	5117.250248	1.517249816	0.172638169	5.26E-19	1.39E-17
Cpne8	1053.091013	2.746020626	0.318099222	5.78E-19	1.52E-17
Sik1	2631.830037	2.176952343	0.251859412	6.87E-19	1.80E-17
Per3	920.3512384	2.248823213	0.259780037	7.13E-19	1.86E-17
Rbpms	2343.026099	1.057972039	0.120641761	7.14E-19	1.86E-17
Jpt1	993.5537819	1.340788871	0.153598063	7.29E-19	1.90E-17
Slx4	295.4564903	1.73294193	0.19929581	7.54E-19	1.96E-17
Lmna	3808.725539	1.310368398	0.150269889	7.73E-19	2.01E-17
Nfkbie	825.1573438	2.115699006	0.244664644	7.83E-19	2.03E-17
Gpx3	580.382517	7.530804759	0.829499765	8.57E-19	2.21E-17
Scd2	2893.259598	4.541717746	0.533527927	9.26E-19	2.38E-17
Pvr	1029.550134	1.127761245	0.129307862	9.30E-19	2.39E-17
Icam1	1380.017496	1.786781949	0.206611116	9.61E-19	2.46E-17
Sparc	3332.374321	2.58709178	0.301478489	1.01E-18	2.57E-17
Tox	167.0679494	1.978142194	0.228968598	1.02E-18	2.61E-17
Arhgef2	883.8819265	5.799117381	0.649830334	1.05E-18	2.67E-17
Slc11a1	90.88817745	2.731701884	0.318191242	1.15E-18	2.91E-17
Ttll7	196.8109785	5.233184295	0.591497142	1.36E-18	3.43E-17
Ngf	821.5664978	10.9723711	2.400109807	1.83E-18	4.58E-17
Osmr	728.5720933	4.94686219	0.568965671	1.86E-18	4.66E-17
Ccdc9b	488.6145843	3.435218431	0.398027382	1.95E-18	4.87E-17

Tspan8	2652.598426	10.90850171	2.321897702	1.97E-18	4.90E-17
Ehd2	874.5795891	3.652187695	0.420982198	2.02E-18	5.03E-17
Smtn	179.215168	2.979542859	0.348917853	2.11E-18	5.24E-17
Panx1	187.9800615	2.865435423	0.333356699	2.31E-18	5.73E-17
Tnip1	1633.710541	1.267445407	0.147652285	2.45E-18	6.06E-17
Map3k6	206.4725233	3.282308149	0.386254895	2.79E-18	6.88E-17
Wfdc2	803.7245441	2.8590404	0.339042971	3.45E-18	8.46E-17
Nol4l	351.6902361	3.098056482	0.36438619	3.56E-18	8.71E-17
Elf3	545.9594396	2.666579422	0.315361136	4.17E-18	1.02E-16
Apobec3	1030.778116	5.700670243	0.652291516	4.84E-18	1.18E-16
AI506816	838.3826187	1.369974026	0.161364509	5.34E-18	1.30E-16
Iqce	668.231522	1.323358055	0.155797112	5.74E-18	1.39E-16
Myl12b	270.2849819	2.396116196	0.283430787	6.09E-18	1.47E-16
Vegfb	442.2222335	1.460852362	0.172524717	6.44E-18	1.55E-16
Slc7a11	289.7717956	12.44780641	2.714338604	6.81E-18	1.64E-16
Ttyh3	1151.164908	1.492815384	0.177437849	1.01E-17	2.40E-16
Flt1	91.95648371	4.593917652	0.544980558	1.04E-17	2.47E-16
Adamts10	323.1297261	2.71577664	0.324269601	1.27E-17	2.98E-16
Nenf	428.8913011	1.233511162	0.1470308	1.78E-17	4.13E-16
Nrg4	319.757649	1.859741158	0.22425002	1.86E-17	4.32E-16
Tnks1bp1	3117.175824	1.016201972	0.121632563	2.02E-17	4.68E-16
Syne4	206.0681172	2.17355425	0.263314209	2.55E-17	5.87E-16
Ccdc88a	262.5824066	3.46820428	0.418176334	2.68E-17	6.16E-16
Enc1	2788.519373	1.350095108	0.162505868	2.69E-17	6.16E-16
Gstm3	9891.051661	1.637389148	0.197998608	2.83E-17	6.47E-16
Klf7	669.0837074	1.529097258	0.184087438	2.85E-17	6.49E-16
Spink1	106.7319113	6.455879152	0.759897383	2.86E-17	6.50E-16
Nt5c3b	304.9629369	1.733738121	0.209372396	3.03E-17	6.87E-16
Dpysl3	1541.559428	5.558369038	0.652995278	3.18E-17	7.22E-16
2010003K11Rik	269.8857951	2.094390797	0.255146057	3.26E-17	7.39E-16
Tgif1	2104.999954	1.126115295	0.136493887	3.40E-17	7.70E-16
Cand2	348.2391153	2.746722163	0.334988936	3.56E-17	8.04E-16
Tmprss2	865.3803796	1.679572724	0.204034333	3.98E-17	8.95E-16
Fgd1	263.1404083	1.787635711	0.217024614	4.20E-17	9.40E-16
Defb1	80.18627089	3.745101019	0.461309305	4.34E-17	9.69E-16
Col4a5	1479.027157	2.187206002	0.267866432	4.45E-17	9.94E-16
Stxbp1	365.8815636	3.605812953	0.433978882	4.51E-17	1.01E-15
Lig1	583.7963409	2.411929002	0.296397984	4.68E-17	1.04E-15
Myo5c	899.6359621	10.54674543	2.369988354	5.05E-17	1.12E-15
Pdgfa	613.5389746	1.063220867	0.128675528	5.64E-17	1.24E-15
Pcgf2	515.508143	1.513284696	0.184502398	5.80E-17	1.28E-15
Zfand2a	394.3956612	1.256052281	0.152603884	5.85E-17	1.29E-15
Prrg4	366.0548761	2.009109165	0.245392679	5.96E-17	1.31E-15

9830144P21Rik	62.84849137	3.430372598	0.422370203	6.10E-17	1.34E-15
Cep170	942.8738129	1.262028169	0.153518958	6.57E-17	1.44E-15
Id3	1361.904331	2.231918914	0.276239675	7.77E-17	1.69E-15
B4galt6	2330.660997	9.493170406	1.236019743	8.16E-17	1.77E-15
Limd2	808.6726302	1.020268841	0.124025935	8.26E-17	1.79E-15
Apoa4	1084.809155	2.574369734	0.319829303	8.53E-17	1.84E-15
Alcam	7033.895068	1.144314154	0.139321968	9.59E-17	2.06E-15
Marcksl1	1259.36562	2.898905894	0.357032033	1.08E-16	2.32E-15
Camk1	677.6305656	1.353527217	0.166009218	1.26E-16	2.69E-15
Homer3	249.8333661	3.713452702	0.452521862	1.30E-16	2.77E-15
Gsta4	1446.511618	1.264161554	0.1554045	1.36E-16	2.89E-15
Per1	1792.911099	2.420168978	0.302538558	1.39E-16	2.96E-15
Slc1a5	134.5869544	2.419238875	0.299707885	1.44E-16	3.07E-15
S100a6	1433.97565	9.441068076	1.242079158	1.49E-16	3.17E-15
Mex3d	432.687381	1.806657029	0.223485285	1.55E-16	3.27E-15
Pck2	692.4583247	1.498686599	0.185544987	1.65E-16	3.48E-15
Myo7b	162.8584993	3.167986075	0.392831138	1.82E-16	3.83E-15
Cdc42ep1	2642.241788	1.06530294	0.131366749	1.92E-16	4.03E-15
Cyp4f16	359.3505711	1.27443297	0.157697232	1.92E-16	4.04E-15
Bin1	559.9559464	1.006838035	0.123977285	2.08E-16	4.36E-15
Card19	422.0498646	1.564645143	0.194741138	2.09E-16	4.38E-15
Cd2ap	6195.42875	1.141804536	0.141378671	2.47E-16	5.14E-15
Dip2a	536.4515405	1.173703506	0.145333731	2.57E-16	5.35E-15
Plscr1	2805.771494	1.752765057	0.219933191	3.18E-16	6.58E-15
Prmt2	389.0165	4.001318137	0.493296808	3.25E-16	6.72E-15
Cfb	373.2758448	1.25846604	0.156539638	3.25E-16	6.72E-15
Ankrd12	565.6495619	1.762030938	0.221709883	3.55E-16	7.32E-15
Zdhhc2	235.6616781	2.229451334	0.280825042	3.84E-16	7.90E-15
Fndc10	160.4928545	7.059745241	0.852976429	3.86E-16	7.93E-15
Gpx2	225.5354571	5.416038674	0.663856197	4.18E-16	8.56E-15
Il17rb	482.2129692	1.307884867	0.163828229	4.26E-16	8.73E-15
Zyx	2597.269208	1.382143599	0.173642437	4.56E-16	9.31E-15
Pparg	190.8325974	1.659186094	0.209336399	4.57E-16	9.31E-15
Megf8	1795.980567	1.105714762	0.138747675	4.68E-16	9.53E-15
Ntn4	703.8153015	2.844129568	0.357252906	4.78E-16	9.71E-15
Patz1	628.759567	1.238664255	0.154729579	4.81E-16	9.76E-15
Plscr3	1050.621523	1.272575947	0.159631416	5.00E-16	1.01E-14
Inava	848.6684542	8.097525449	0.948546344	5.09E-16	1.03E-14
Naip2	377.4846798	2.028465016	0.257831797	5.22E-16	1.06E-14
Tspan15	164.8650929	3.304526894	0.417084546	5.36E-16	1.08E-14
Sema3b	343.1791066	3.184715238	0.406138204	6.05E-16	1.22E-14
Prom1	1700.99516	5.846588177	0.71229252	6.22E-16	1.25E-14
Rbp1	1190.235878	1.314084798	0.165993615	7.23E-16	1.45E-14

2200002D01Rik	422.0612183	1.356453845	0.171434288	7.36E-16	1.47E-14
Sat1	2158.541359	1.549654307	0.196797014	7.65E-16	1.53E-14
Igdcc4	576.4080511	10.08013172	2.367457897	8.23E-16	1.64E-14
Caskin2	917.0766179	1.142152281	0.144343236	8.41E-16	1.68E-14
Cldn4	2945.534617	10.17558507	2.325285256	8.56E-16	1.71E-14
Cys1	897.4977421	2.234828933	0.286123684	8.57E-16	1.71E-14
Ccn5	429.3534084	10.19206185	2.393076996	8.90E-16	1.77E-14
Fgfr2	2376.307682	1.022390816	0.128867786	9.18E-16	1.82E-14
Ihh	144.1151708	2.907589523	0.376446988	9.91E-16	1.96E-14
Tmem237	245.9704624	1.511901485	0.19236502	1.09E-15	2.16E-14
Il10rb	334.0185372	1.892494164	0.241750512	1.11E-15	2.19E-14
Adrb2	115.8926575	4.649546714	0.590003496	1.16E-15	2.29E-14
H2-T-ps	65.49388414	4.871006565	0.628917792	1.29E-15	2.53E-14
Plp2	422.2736418	3.283069555	0.41926012	1.32E-15	2.58E-14
Elmo1	189.5489611	1.970488318	0.253282592	1.37E-15	2.67E-14
Irak3	201.5284826	4.037228502	0.524647102	1.40E-15	2.72E-14
Gas6	8246.924178	1.37541654	0.176142238	1.51E-15	2.92E-14
Myof	3592.60382	7.899529148	0.93788107	1.62E-15	3.12E-14
Gmip	381.6356426	1.329280952	0.170079018	1.72E-15	3.30E-14
Llgl1	950.5135454	1.006586729	0.128184249	1.80E-15	3.45E-14
Pofut2	689.6872368	1.0793335	0.137564519	1.87E-15	3.59E-14
Dusp10	443.5759271	1.62355643	0.209698213	2.14E-15	4.08E-14
Usp43	282.5132071	9.936082669	2.378376387	2.14E-15	4.08E-14
Itga3	4405.744063	1.05445371	0.134799658	2.16E-15	4.12E-14
Pmepa1	571.701155	3.453832681	0.444822411	2.43E-15	4.62E-14
Ldb1	1551.88774	1.183519308	0.151944758	2.44E-15	4.65E-14
Slc48a1	1834.725727	1.335626303	0.171711154	2.55E-15	4.85E-14
Eif6	2382.439995	1.403160481	0.181630495	2.61E-15	4.95E-14
Phlda2	49.27107646	3.134135861	0.408665469	2.80E-15	5.30E-14
Sdk1	46.62161858	4.518592074	0.577819945	3.28E-15	6.19E-14
Cfap44	169.760744	9.937909651	2.418428301	3.46E-15	6.52E-14
Arrb2	557.5798128	1.342413208	0.173976743	3.93E-15	7.39E-14
Cbr1	2163.358895	1.400926063	0.182053447	4.16E-15	7.81E-14
Prkx	470.6073335	1.247864092	0.162230723	4.30E-15	8.05E-14
Rph3al	222.2133515	1.866409109	0.244357763	4.48E-15	8.37E-14
Filip1l	853.4650318	3.08632359	0.401652774	5.11E-15	9.50E-14
Glis3	1805.987742	8.841594063	1.225152713	5.20E-15	9.66E-14
Nt5c	624.4954379	1.140433676	0.148338593	5.68E-15	1.05E-13
Gsn	204.4074316	3.857084161	0.502755594	5.82E-15	1.07E-13
Capg	357.6854752	3.7082518	0.482248863	6.56E-15	1.21E-13
Sfi1	715.685044	1.560446439	0.205428087	6.75E-15	1.24E-13
Ccl2	1991.525862	9.041125329	1.26222352	7.04E-15	1.29E-13
Arrdc1	598.5550887	2.471477818	0.327419114	7.07E-15	1.29E-13

Smpdl3b	85.33293135	4.15930076	0.563881611	1.30E-14	2.35E-13
Sema6b	136.7105071	2.093610489	0.281300481	1.39E-14	2.48E-13
Wls	710.2318948	4.071127002	0.539510151	1.62E-14	2.88E-13
Gramd1b	1296.929223	5.409544651	0.695736507	2.05E-14	3.61E-13
Pwwp2b	389.8822049	1.777423812	0.238556101	2.06E-14	3.63E-13
Ift57	380.7265344	2.608226327	0.349150272	2.08E-14	3.65E-13
Myzap	218.2572142	4.970773497	0.648445325	2.17E-14	3.80E-13
Wtip	322.9003128	3.515185108	0.468978906	2.45E-14	4.26E-13
Tubb2b	799.4274759	2.87809359	0.387469377	2.77E-14	4.79E-13
Cntln	203.4023102	2.935633569	0.394364355	2.94E-14	5.09E-13
Cyp21a1	42.28162249	5.49506169	0.734014519	3.53E-14	6.05E-13
Igsf1	246.8485605	2.415866465	0.329061509	4.21E-14	7.18E-13
Sntb2	658.9291161	1.233292766	0.167740926	4.27E-14	7.28E-13
Gm10382	298.4206319	2.226799275	0.306080665	4.36E-14	7.41E-13
Kif21b	199.0264851	4.567312151	0.620614962	4.53E-14	7.69E-13
Ralgds	504.2243016	2.523327932	0.343992567	4.53E-14	7.69E-13
Plekha1	577.5528988	1.091967932	0.147256012	4.83E-14	8.17E-13
Dnah1	50.18948693	3.666703906	0.505082562	4.93E-14	8.33E-13
Tbc1d1	937.9133007	1.729766156	0.236403502	5.27E-14	8.86E-13
Hspb1	1610.709725	1.67880924	0.230132431	5.26E-14	8.86E-13
Myc	751.0874706	2.394666373	0.331499956	5.47E-14	9.17E-13
Adcy6	1714.314775	1.043379418	0.141058317	5.50E-14	9.22E-13
Neurl1a	470.1662382	6.289350378	0.819773881	5.86E-14	9.80E-13
Ccdc34	319.247354	1.526609487	0.207988375	6.81E-14	1.14E-12
Col1a2	210.6582226	5.423233876	0.761530567	6.99E-14	1.16E-12
Scube3	1894.471044	6.101474677	0.794659842	7.96E-14	1.32E-12
Klf16	267.4824656	1.593769781	0.219776548	8.10E-14	1.34E-12
Ppp1r14b	821.5301011	1.586678494	0.218453495	8.12E-14	1.34E-12
Arhgap22	127.3198972	9.407839398	2.396282636	8.27E-14	1.37E-12
Iffo2	802.0168097	1.352752527	0.185704598	8.79E-14	1.45E-12
Gpnmb	193.3997859	8.41311897	1.116122528	8.99E-14	1.48E-12
Sinhcaf	306.9271048	1.965234792	0.270704947	9.15E-14	1.51E-12
Smc4	580.4512356	1.0567421	0.144232034	9.66E-14	1.59E-12
Nlgn2	821.4250603	2.32958015	0.323903456	9.92E-14	1.63E-12
Evpl	386.3438727	3.968232614	0.540819763	1.03E-13	1.69E-12
Evc2	494.090182	2.650587829	0.366515433	1.11E-13	1.81E-12
Hsf4	173.3766554	1.301727075	0.179120715	1.13E-13	1.84E-12
Bhlhe40	4417.571025	1.27399246	0.175508923	1.14E-13	1.86E-12
Cdc42ep5	191.1716294	2.144607477	0.299182939	1.14E-13	1.86E-12
Dvl2	358.4362429	1.294864977	0.178430315	1.20E-13	1.95E-12
Hmox1	397.8150982	2.890228123	0.407621006	1.21E-13	1.97E-12
Ssh3	1102.583597	1.11789478	0.153664256	1.33E-13	2.16E-12
Slc4a9	108.0643503	4.824545097	0.663537731	1.38E-13	2.23E-12

Plekhh1	484.7890274	2.536306974	0.352099695	1.52E-13	2.44E-12
Mturn	357.8250301	2.132234429	0.299701922	1.82E-13	2.89E-12
Nob1	296.8376211	1.114670298	0.154150323	1.89E-13	3.00E-12
Aqp7	53.23588197	3.293284335	0.467614718	2.14E-13	3.40E-12
Sgk1	893.018067	1.550121763	0.216346617	2.16E-13	3.42E-12
Tspo	2103.880392	1.088771587	0.151299916	2.26E-13	3.56E-12
Tinag	63.22689619	9.360824484	2.441283056	2.30E-13	3.62E-12
Rhno1	221.0462134	1.284141767	0.178941597	2.41E-13	3.80E-12
Arntl2	239.926964	2.071478919	0.291970425	2.46E-13	3.86E-12
Abi2	345.2022163	1.723331505	0.242872308	2.83E-13	4.43E-12
Wfdc3	166.8394908	8.266362177	1.240793664	2.92E-13	4.55E-12
Gadd45b	590.3505394	2.331339284	0.330751167	2.96E-13	4.60E-12
Nop56	712.0553827	1.060775713	0.147933665	3.02E-13	4.70E-12
Gm47163	168.2346981	9.409919651	2.397409148	3.02E-13	4.70E-12
Spire2	89.62898808	5.543209872	0.760937436	3.09E-13	4.80E-12
Slc10a2	1044.596959	1.74876222	0.247186736	3.15E-13	4.89E-12
Ncoa7	222.6560308	3.342814474	0.467941696	3.17E-13	4.91E-12
Cbarp	627.3869157	1.75722071	0.248660038	3.32E-13	5.13E-12
Pisd-ps1	346.6669703	1.15240251	0.16135603	3.42E-13	5.27E-12
Cdk1	134.3869496	3.015381815	0.431957898	3.81E-13	5.86E-12
Eln	69.43323387	5.286429256	0.741216175	3.90E-13	5.99E-12
Wipf3	295.4003071	1.413399144	0.199936929	4.01E-13	6.16E-12
Gar1	254.5375941	1.396639205	0.197694868	4.48E-13	6.83E-12
Srgap2	1732.919358	1.073052406	0.15083579	4.49E-13	6.84E-12
Arhgap11a	186.5055447	2.553012091	0.36783346	4.68E-13	7.12E-12
Wee1	633.3205705	2.022429872	0.290956551	5.21E-13	7.87E-12
AC120557.1	465.4717434	1.176882359	0.16619793	5.44E-13	8.21E-12
2410002F23Rik	543.5089778	1.328471673	0.18861521	5.49E-13	8.28E-12
Glis2	1730.018636	8.809953528	2.237531616	6.06E-13	9.11E-12
Piezo1	568.0378654	1.502228805	0.214289837	6.29E-13	9.44E-12
Cldn7	1159.772996	8.068632723	1.222013888	6.83E-13	1.02E-11
Nrm	108.2153474	3.38671312	0.480545466	7.23E-13	1.08E-11
Orm2	812.1729721	2.562952358	0.375057217	8.87E-13	1.31E-11
Sh3bgrl3	845.8108824	2.235424199	0.321804299	8.91E-13	1.32E-11
Chst14	157.8270391	2.026879686	0.292547027	1.03E-12	1.52E-11
Crybg2	82.83462426	1.7847412	0.258483513	1.12E-12	1.65E-11
Zfp408	282.5098689	1.122015948	0.160297596	1.13E-12	1.66E-11
Mlph	956.576832	5.745949519	0.786308039	1.14E-12	1.67E-11
Dhrs13	175.7217994	1.67164684	0.242707753	1.15E-12	1.68E-11
Fam110c	159.0920429	1.804321355	0.262003058	1.29E-12	1.89E-11
Gimap8	199.25466	1.874356537	0.272025847	1.43E-12	2.09E-11
Stab1	192.5229586	1.833407689	0.269085332	1.58E-12	2.31E-11
Ccnb2	93.98681835	5.41486695	0.770585981	1.71E-12	2.49E-11

S100g	48.57264044	9.313432101	2.519951141	1.80E-12	2.61E-11
Relb	949.3516041	3.176315277	0.475024541	1.83E-12	2.65E-11
Vasp	1151.082075	1.371632556	0.19993795	1.84E-12	2.65E-11
Ppan	430.995834	1.154335246	0.167510102	2.04E-12	2.94E-11
Anxa9	147.648838	2.408113369	0.35515959	2.29E-12	3.28E-11
F2rl1	687.9524739	8.617552664	2.262344641	2.39E-12	3.42E-11
Cx3cl1	553.4927526	8.666055885	2.285650701	2.61E-12	3.72E-11
Col6a1	131.2314135	4.429895782	0.643249825	2.65E-12	3.78E-11
Zfp579	418.3026074	1.209827611	0.177423212	2.80E-12	3.99E-11
Tpd52	1203.018971	1.004409371	0.146491308	3.12E-12	4.44E-11
Ccdc80	80.17158069	3.601156	0.540525792	3.17E-12	4.50E-11
Fmnl3	172.9649336	4.569497886	0.658031552	3.20E-12	4.54E-11
Ltbp2	2001.585713	3.982499634	0.596264017	3.33E-12	4.71E-11
Tuft1	1638.192115	1.601015198	0.237840347	3.44E-12	4.86E-11
Hes1	3064.428263	2.678215214	0.406414655	3.75E-12	5.28E-11
Fam124a	562.6914112	1.821034351	0.272036956	3.86E-12	5.43E-11
Nek8	575.843703	1.225468334	0.180629845	3.94E-12	5.53E-11
Fam83g	249.1450262	1.416076534	0.209666376	3.98E-12	5.59E-11
Fyb2	313.8812169	1.424998137	0.211473754	4.01E-12	5.63E-11
Zfp7	227.0214703	1.744920581	0.259467489	4.24E-12	5.93E-11
Mfsd10	530.4308295	1.637285965	0.244682387	4.76E-12	6.63E-11
Plekha2	518.5000918	2.961048389	0.437768795	4.85E-12	6.74E-11
Tlr2	197.9122868	2.040330664	0.307392512	4.99E-12	6.92E-11
Fes	209.5557772	1.632999609	0.24477706	5.14E-12	7.10E-11
Loxl2	261.5046318	3.874581135	0.569524814	5.14E-12	7.10E-11
Hp	63465.89234	1.186893633	0.176629469	6.34E-12	8.69E-11
Mki67	612.7062076	7.531150431	1.086438786	7.29E-12	9.93E-11
Plekhg5	368.7457472	1.335803242	0.200747646	7.90E-12	1.07E-10
Neurl3	937.2942732	3.956371852	0.611967006	7.94E-12	1.08E-10
Nol3	60.02810625	2.187199064	0.33337137	8.46E-12	1.14E-10
Guca2b	85.09988825	8.649640188	2.365316634	8.80E-12	1.19E-10
Snta1	1188.451695	1.010995639	0.150850069	8.90E-12	1.20E-10
Otud1	549.4273923	1.689784833	0.257044216	9.09E-12	1.22E-10
Car13	246.9664239	5.543975998	0.795994561	9.29E-12	1.25E-10
Gcnt2	860.2779428	1.042873812	0.156473359	9.64E-12	1.29E-10
Syt14	302.476795	5.596505878	0.806526088	9.73E-12	1.30E-10
Tcim	1708.433264	1.729479867	0.264314766	1.08E-11	1.44E-10
Col6a3	98.86697856	9.844594406	2.57178346	1.24E-11	1.64E-10
Knstrn	85.9622077	4.31841862	0.657242429	1.27E-11	1.68E-10
Casp4	284.5309296	9.397646294	2.451985561	1.29E-11	1.70E-10
Morc4	210.0469946	1.463428801	0.2228235	1.29E-11	1.71E-10
Exoc6b	566.5901023	1.039320969	0.156725978	1.40E-11	1.85E-10
Lrrc8e	194.3214274	3.977729113	0.59329945	1.41E-11	1.85E-10

Abcc1	531.2414062	5.821230627	0.854936967	1.47E-11	1.93E-10
Rdh13	280.8483168	1.078458977	0.163321473	1.51E-11	1.99E-10
Ankrd6	231.7641769	4.763687262	0.700734425	1.52E-11	1.99E-10
Dqx1	286.2680548	1.467582427	0.224672525	1.57E-11	2.05E-10
Gm4673	242.5809454	1.514772367	0.23239163	1.60E-11	2.09E-10
Fblim1	1361.651429	7.512501745	1.201124075	1.65E-11	2.15E-10
Herc3	481.0671561	1.307929978	0.199814343	1.71E-11	2.22E-10
Tbc1d4	316.2216909	1.521537964	0.23334522	1.96E-11	2.54E-10
Serpinb8	97.19369215	1.759521464	0.272357401	1.98E-11	2.57E-10
Agpat1	579.3948924	1.553560255	0.239771306	2.00E-11	2.60E-10
Slc5a1	1330.675187	6.67838095	0.956184566	2.09E-11	2.69E-10
Ehbp1l1	179.9912164	1.480174276	0.228184644	2.12E-11	2.73E-10
Anks6	165.4853608	2.625652071	0.408050474	2.34E-11	3.01E-10
C1qtnf6	270.8704403	3.021773557	0.466315968	2.35E-11	3.03E-10
Csf2ra	283.1402822	1.805228092	0.279063563	2.43E-11	3.12E-10
Cxcl10	146.0387893	2.367615842	0.372548664	2.44E-11	3.13E-10
Baiap2	1499.209392	1.246869164	0.192102899	2.52E-11	3.23E-10
Nrip2	99.37550179	4.004308238	0.6081303	2.54E-11	3.25E-10
Insyn1	111.7776807	6.826303978	0.996311023	2.77E-11	3.54E-10
Slc12a4	914.6871322	1.110268608	0.170521423	2.87E-11	3.65E-10
Trim35	1506.964685	2.750828434	0.425290412	3.08E-11	3.91E-10
Neu3	117.7387385	1.58643996	0.24865844	3.31E-11	4.20E-10
Nampt	1349.240771	1.501199785	0.233917478	3.36E-11	4.26E-10
Itpkc	261.729967	1.258166003	0.195063276	3.53E-11	4.46E-10
Tlcd5	236.7492689	3.306970822	0.521351977	3.55E-11	4.48E-10
Enoph1	220.9560518	1.130364816	0.174772195	4.05E-11	5.06E-10
Ctdspl	482.493607	1.120809157	0.173295756	4.15E-11	5.17E-10
Sdsl	456.2238763	1.144650673	0.177617915	4.36E-11	5.43E-10
Bcl9l	2085.715665	1.047034324	0.16225093	4.47E-11	5.57E-10
Fam131c	129.5904495	1.494171255	0.234381066	4.60E-11	5.71E-10
H2-Q5	311.7463863	1.491542463	0.234035268	4.63E-11	5.74E-10
Gm45819	82.04792913	3.189830214	0.509479561	4.66E-11	5.78E-10
Csnk1e	773.958128	1.074707687	0.166726467	4.76E-11	5.90E-10
Lmcd1	250.8541308	5.20931328	0.806036107	5.19E-11	6.40E-10
Cchcr1	625.7546817	1.132003158	0.176356224	5.30E-11	6.54E-10
Tpcn2	203.2300929	1.464675702	0.23033684	5.35E-11	6.59E-10
Jrk	125.6152616	1.781513381	0.28134357	5.38E-11	6.61E-10
Tulp3	339.4172723	5.321162943	0.796582922	5.42E-11	6.66E-10
Gm49172	178.1896256	3.135176596	0.490665667	5.52E-11	6.78E-10
Snrpf	163.218997	1.833368849	0.291343018	5.90E-11	7.23E-10
Ephb6	336.9424549	3.022673894	0.476006376	6.06E-11	7.41E-10
Dbf4	120.8693932	1.813587748	0.288508863	6.38E-11	7.79E-10
Cdkn2c	312.9823092	1.358814889	0.214454637	6.41E-11	7.82E-10

Cav1	1004.066112	3.040962185	0.484336783	6.48E-11	7.90E-10
Nfatc2	327.7043917	7.308322628	1.224124474	6.61E-11	8.04E-10
Eps8l3	66.40454041	8.821691834	2.454717166	6.83E-11	8.31E-10
Gpx7	100.0423599	1.691048634	0.268413386	7.47E-11	9.05E-10
Fgf21	40.13117571	3.860698577	0.62677586	7.89E-11	9.53E-10
Apbb1	340.3294945	3.610176224	0.565181044	7.97E-11	9.62E-10
Sfn	352.3405021	3.963015761	0.634177483	8.00E-11	9.65E-10
Gabbr1	495.1106324	2.856661874	0.452631501	8.03E-11	9.67E-10
Comtd1	59.46361853	2.570726588	0.416075375	8.07E-11	9.72E-10
Prdm16	278.5639074	3.897578806	0.60827606	8.08E-11	9.72E-10
Dsn1	106.9846128	2.206328295	0.352638828	8.82E-11	1.06E-09
Ccdc82	468.2548225	1.0470342	0.164728752	8.83E-11	1.06E-09
Dpysl2	148.3984993	2.394409612	0.384202671	9.04E-11	1.08E-09
Sybu	37.60756693	5.034609742	0.790208496	9.61E-11	1.15E-09
Marchf9	316.2670096	1.993360951	0.318745554	9.94E-11	1.18E-09
Rgs2	502.9169095	7.095573751	1.089254356	1.03E-10	1.23E-09
H2-Q2	124.1725493	3.669721921	0.581715287	1.09E-10	1.30E-09
Cdc20	133.4927589	3.402912703	0.553887809	1.10E-10	1.30E-09
Tmem229a	684.456143	7.860531943	2.209715479	1.14E-10	1.36E-09
Glipr2	242.2080239	3.277315653	0.533939217	1.15E-10	1.37E-09
Sytl5	513.7245561	7.227549904	1.227715004	1.19E-10	1.41E-09
Rdh9	144.3379357	1.772722975	0.287500498	1.27E-10	1.50E-09
Eif4e3	274.3497036	1.8954037	0.308814547	1.32E-10	1.56E-09
Tspan4	886.0963286	1.08119825	0.17217918	1.33E-10	1.56E-09
Rbm3	1762.268476	1.348716725	0.216911684	1.37E-10	1.61E-09
Bex3	306.8733245	3.723316377	0.594707423	1.37E-10	1.61E-09
Pprc1	570.1787801	1.403734303	0.226495909	1.48E-10	1.73E-09
Cxcl14	137.5969544	2.430300815	0.401219173	1.58E-10	1.84E-09
Timp3	835.1460626	2.688924084	0.443664895	1.71E-10	1.99E-09
Rhoq	728.9108823	1.102072961	0.176832204	1.79E-10	2.08E-09
Ift43	203.7368131	1.516401515	0.24541028	1.79E-10	2.08E-09
Lrrc45	516.0403133	1.079780555	0.173351956	1.86E-10	2.16E-09
Ccdc57	205.859654	1.253392004	0.201928563	1.89E-10	2.19E-09
Cyp2a5	16867.11266	1.475141434	0.239877959	1.90E-10	2.20E-09
Msantd3	155.5618807	7.102979291	1.224326422	1.97E-10	2.28E-09
Baiap2l2	96.56837681	7.974499056	2.306392867	2.02E-10	2.33E-09
Cdh17	307.0721841	7.903678765	2.254752119	2.09E-10	2.41E-09
Rasl10b	46.12216366	3.396238993	0.566703875	2.13E-10	2.44E-09
Ifngr1	1341.758505	1.259200985	0.2037477	2.13E-10	2.45E-09
Col16a1	1913.69806	5.318405537	0.833094537	2.25E-10	2.57E-09
Srd5a3	345.3488126	1.026748029	0.165433597	2.34E-10	2.67E-09
Crip1	289.1873147	5.412603141	0.853545344	2.41E-10	2.74E-09
Col5a1	79.65745786	4.293633439	0.702054912	2.49E-10	2.82E-09

Kcnj8	108.4920928	5.22573058	0.817825576	2.52E-10	2.87E-09
Ifrd1	911.3777563	1.68362181	0.27838932	2.68E-10	3.03E-09
Cdt1	223.480793	1.385042377	0.226569178	2.69E-10	3.04E-09
Dapp1	411.7185136	6.17312927	0.937658028	2.73E-10	3.09E-09
Srxn1	2117.593055	1.14585438	0.186174206	2.74E-10	3.10E-09
Usp35	163.7154416	1.673264214	0.274706143	2.75E-10	3.10E-09
Btc	328.365126	1.454405186	0.237725209	2.80E-10	3.16E-09
Racgap1	167.0399256	3.305965073	0.552806968	2.83E-10	3.19E-09
Zfp791	86.93940525	1.582360176	0.260002829	2.94E-10	3.31E-09
Timp1	96.65089006	7.964159145	2.311178285	2.95E-10	3.32E-09
Grk3	432.1468736	1.16698008	0.190578456	3.18E-10	3.55E-09
Cdca3	109.9265191	2.515626774	0.423534952	3.31E-10	3.68E-09
A330074K22Rik	125.2870763	7.785046189	2.272554832	3.42E-10	3.80E-09
Syde1	471.9481987	6.151342545	0.939992732	3.46E-10	3.84E-09
Atp6v0d2	83.47036861	2.373746714	0.3998361	3.47E-10	3.85E-09
Ccn2	5388.782483	6.962097689	1.205158124	3.52E-10	3.90E-09
Phactr2	475.4612223	1.278472553	0.209478388	3.54E-10	3.92E-09
Pls1	575.5978219	1.0979144	0.179407933	3.60E-10	3.98E-09
Gm20008	148.508298	1.890463673	0.315266496	3.61E-10	4.00E-09
Sptlc2	1670.012782	1.088715269	0.177874813	3.62E-10	4.01E-09
Ap3m2	167.7451292	1.90531106	0.315569259	3.66E-10	4.04E-09
Lgmn	1085.643284	1.499850032	0.248603896	3.68E-10	4.06E-09
Top2a	483.1935635	4.006513094	0.686057692	3.71E-10	4.09E-09
Cep192	492.0943633	1.197429718	0.196724864	4.00E-10	4.40E-09
Zfp651	409.0902105	3.977342632	0.641495264	4.11E-10	4.51E-09
Lipn	34.2203007	8.399919902	2.499241632	4.17E-10	4.56E-09
Hid1	442.212484	3.700815549	0.632114235	4.21E-10	4.60E-09
Samd1	361.15989	1.182504675	0.194910322	4.37E-10	4.77E-09
Dsg1c	553.2878547	2.782065038	0.475841261	4.44E-10	4.84E-09
Baz1a	555.3098189	1.112946318	0.1828369	4.65E-10	5.05E-09
Foxm1	141.6650028	4.740969834	0.772900801	4.89E-10	5.29E-09
Cdc25a	362.6038333	1.007835625	0.165344449	4.96E-10	5.37E-09
Jade2	953.8595446	1.712156335	0.288721838	5.08E-10	5.49E-09
Ciart	136.3040224	2.905502758	0.500185142	5.53E-10	5.95E-09
Krt8-ps	71.82928407	1.529486908	0.255806433	5.96E-10	6.41E-09
Uhrf1	378.206148	4.951064796	0.854853891	6.00E-10	6.44E-09
Slc5a9	47.01619117	7.781077733	2.322663943	6.05E-10	6.48E-09
Hcn3	136.3199744	1.380371401	0.231333827	6.49E-10	6.95E-09
Rassf1	204.8080249	1.248318574	0.207691604	6.55E-10	7.01E-09
Col3a1	373.5077786	4.848008469	0.85391695	6.83E-10	7.29E-09
Coq8b	575.2329452	1.017488593	0.168674992	6.97E-10	7.43E-09
Gm16505	92.69693653	7.902156887	2.323500362	7.46E-10	7.92E-09
Adap1	133.0891506	3.493769474	0.583072886	7.56E-10	8.03E-09

Csn3	23.81410256	8.359174399	2.527512328	8.37E-10	8.84E-09
Actg1	14618.63977	1.295591435	0.219214096	8.72E-10	9.20E-09
Angptl2	57.6142951	3.523627929	0.59771543	8.76E-10	9.25E-09
Timp2	2446.47702	3.554942833	0.612963769	8.81E-10	9.29E-09
Cit	163.2408144	6.084396343	0.960767602	8.92E-10	9.39E-09
Gas8	300.9570558	1.343207404	0.226409954	9.06E-10	9.53E-09
Ccnd1	2705.489028	1.714465814	0.293556489	9.34E-10	9.82E-09
Tprn	347.3203135	1.286483145	0.217176249	9.38E-10	9.85E-09
Nrp2	304.8150418	1.39995417	0.23646924	9.45E-10	9.92E-09
Heg1	3677.045021	1.412927446	0.24003737	9.89E-10	1.04E-08
AA986860	446.68068	3.166529081	0.557446514	1.00E-09	1.05E-08
Ift122	507.7082696	1.031284792	0.172676992	1.08E-09	1.12E-08
Ildr2	1117.511288	1.23310175	0.208890304	1.13E-09	1.18E-08
Wnt4	683.4328774	5.966623124	0.94489678	1.21E-09	1.25E-08
Zfp90	224.9905554	1.795027893	0.308107943	1.33E-09	1.38E-08
Mcm3	349.9815276	1.886556264	0.327896287	1.41E-09	1.45E-08
Gm47283	340.8689251	1.273843174	0.217686942	1.46E-09	1.50E-08
4930503L19Rik	179.7783678	3.278401566	0.561170547	1.47E-09	1.51E-08
Tmem71	118.1168895	1.720382647	0.298392941	1.56E-09	1.60E-08
Gm16559	139.9860421	2.018156177	0.353273559	1.58E-09	1.62E-08
Icosl	55.80391743	2.667548595	0.471736772	1.70E-09	1.73E-08
Afap1	518.9602312	4.24062621	0.705228652	1.71E-09	1.74E-08
Elapor1	640.0389491	1.896868245	0.331089805	1.72E-09	1.75E-08
Styk1	38.49326863	7.73363198	2.35315541	1.75E-09	1.78E-08
CAAA01147332.1	282.2392826	1.958146069	0.343553841	1.77E-09	1.80E-08
Cerk	281.5153262	1.966432793	0.339976372	1.78E-09	1.80E-08
Trpm4	296.6896639	2.556860285	0.44642365	1.84E-09	1.87E-08
Tchp	218.0761285	1.39568539	0.239854479	1.86E-09	1.89E-08
Emp1	41.72783695	7.822141134	2.362101541	1.88E-09	1.90E-08
Trpv4	353.5537042	7.50176785	2.237050229	1.89E-09	1.91E-08
Gm49599	86.69629351	1.831574884	0.318687147	1.89E-09	1.91E-08
Zfp28	153.3318207	1.857848205	0.32376935	2.00E-09	2.01E-08
Fhl3	270.4051534	6.748173865	1.239932795	2.03E-09	2.04E-08
Lrp8	50.6908175	5.021881166	0.837078462	2.07E-09	2.08E-08
Taf1d	544.3843068	1.212172664	0.209015097	2.08E-09	2.09E-08
Npc1l1	24.68061548	7.696588746	2.372063678	2.10E-09	2.10E-08
Bmyc	120.5819691	2.003104071	0.348241175	2.13E-09	2.13E-08
Gm42892	122.7300029	2.035904155	0.356371041	2.14E-09	2.13E-08
Clcn5	442.4630899	1.070898514	0.183598318	2.18E-09	2.18E-08
Fosl2	1002.982682	1.448841275	0.252296472	2.27E-09	2.26E-08
Kctd12	316.8826791	1.256036186	0.217002904	2.40E-09	2.38E-08
Rnf145	257.9462439	1.246347952	0.215827478	2.43E-09	2.41E-08
Adgrg1	2979.999731	1.213633239	0.209602197	2.44E-09	2.42E-08

Gbp8	195.621099	2.231227894	0.391121725	2.57E-09	2.54E-08
Cers6	759.3318803	1.470205147	0.258581005	2.78E-09	2.73E-08
Tmem86a	403.5285835	3.079321767	0.559883227	2.84E-09	2.79E-08
Tle6	219.2206023	1.602653819	0.281056618	2.85E-09	2.79E-08
Fam83a	45.01744728	2.717663183	0.489039279	2.96E-09	2.89E-08
Osbpl10	69.4691096	7.52703269	2.296801331	3.02E-09	2.95E-08
Evc	678.8361527	1.818137863	0.322330442	3.42E-09	3.32E-08
Smad6	231.3298228	1.335425952	0.234861498	3.78E-09	3.66E-08
Dnttip1	433.5773599	1.003711247	0.174487433	3.88E-09	3.75E-08
Col6a2	77.93271812	3.047846075	0.547570472	4.02E-09	3.88E-08
Irx3	142.4074215	3.23355851	0.58929251	4.08E-09	3.93E-08
Mcm5	323.9799471	2.368238768	0.430425769	4.32E-09	4.16E-08
Ctnnal1	289.8493947	1.20037026	0.210985147	4.48E-09	4.31E-08
Il1r1	1044.662453	1.234362472	0.218001548	4.60E-09	4.41E-08
Hspa1b	1231.268256	1.750587224	0.316109388	5.16E-09	4.90E-08
Scai	499.9084881	1.105576612	0.194783123	5.29E-09	5.01E-08
Rcan3	288.8356164	3.198483721	0.565109106	5.86E-09	5.54E-08
Acot3	44.61349219	2.540078691	0.464866717	6.08E-09	5.73E-08
Tacc3	166.9694321	1.828869275	0.329805099	6.08E-09	5.73E-08
C2cd4c	122.5220656	6.467225411	1.229729411	6.13E-09	5.77E-08
Phgdh	206.8520836	4.661905922	0.797399526	6.81E-09	6.38E-08
Klhl13	455.4124823	1.173876326	0.209167462	6.89E-09	6.45E-08
Tuba1a	638.7868892	2.441128578	0.441798078	6.91E-09	6.46E-08
Fbn1	40.7176792	4.058631318	0.751045631	8.41E-09	7.79E-08
Fosb	3546.966915	4.131943617	0.789453147	8.42E-09	7.80E-08
CT010467.1	1492.916346	2.87244282	0.540332501	8.50E-09	7.86E-08
Il34	213.3188316	6.517460726	1.255391162	9.02E-09	8.32E-08
Nrxn2	33.09278625	4.431649255	0.824590045	9.13E-09	8.41E-08
Ccne1	49.27501703	2.303241181	0.423126327	9.18E-09	8.45E-08
Acnat2	1061.621396	1.417594425	0.257184557	9.21E-09	8.48E-08
Tpx2	124.6648931	4.58273023	0.827404269	9.25E-09	8.52E-08
1810064F22Rik	163.7644254	1.718834233	0.314828284	9.27E-09	8.53E-08
Mlkl	292.4996137	1.125287891	0.202525795	1.00E-08	9.19E-08
Igfbp6	53.5334736	7.139954661	2.273885654	1.02E-08	9.35E-08
Kif5c	79.68858742	7.127334382	2.244175972	1.03E-08	9.46E-08
Arl10	177.8805636	1.143709676	0.205655413	1.04E-08	9.50E-08
Tead4	177.9536918	6.939960359	2.173175355	1.10E-08	1.00E-07
Adarb1	407.6972947	1.103252161	0.198812778	1.10E-08	1.01E-07
Sap30	117.8815048	1.294601031	0.234482141	1.11E-08	1.01E-07
Gab2	171.2554556	3.019303413	0.548520646	1.13E-08	1.03E-07
Gas2l1	1124.24699	1.100628635	0.198905854	1.14E-08	1.04E-07
Fstl1	360.0454495	5.716005802	0.981557518	1.18E-08	1.07E-07
Plat	563.8693799	7.06459065	2.187768168	1.18E-08	1.07E-07

Chrnb1	999.15239	6.24009944	1.202616745	1.20E-08	1.09E-07
Zfp251	198.7566525	1.486118771	0.270778644	1.23E-08	1.12E-07
Zdhhc8	586.9771956	1.027586622	0.185850464	1.26E-08	1.14E-07
Serpinh1	630.7138437	2.38677323	0.444386775	1.29E-08	1.16E-07
Cfap69	284.8246777	3.985429599	0.706291958	1.29E-08	1.17E-07
Pgap4	192.0518371	6.931632728	2.184489631	1.30E-08	1.17E-07
Elf4	469.0925564	3.980524912	0.705250893	1.31E-08	1.18E-07
Bgn	567.8177548	3.130807726	0.600073643	1.34E-08	1.20E-07
C230037L18Rik	28.31317739	3.653558701	0.670039307	1.34E-08	1.21E-07
Abr	933.1223191	1.322727226	0.241227333	1.35E-08	1.21E-07
C1qtnf1	105.6272893	1.798016656	0.332669027	1.39E-08	1.24E-07
AI314278	17.25479758	7.59677328	2.433145305	1.40E-08	1.25E-07
Fbn2	20.35124697	7.514174945	2.402986024	1.47E-08	1.31E-07
Asb11	17.31992749	7.639602063	2.460162984	1.53E-08	1.36E-07
Zfp984	144.0600448	1.572075644	0.291177167	1.53E-08	1.37E-07
Mt1	3292.755801	3.36226035	0.649187764	1.55E-08	1.38E-07
Cenpt	190.8069437	1.507948342	0.278730559	1.56E-08	1.39E-07
Kntc1	117.5399113	7.376013393	2.322164615	1.72E-08	1.52E-07
Mrc2	22.41579253	7.291806068	2.350416042	1.78E-08	1.58E-07
Dapk2	189.1217962	1.397756836	0.259656524	1.88E-08	1.66E-07
Plaur	449.2057924	5.527077153	0.957556644	1.93E-08	1.69E-07
Itga2	1252.831031	3.311275372	0.61594128	1.93E-08	1.69E-07
Anln	148.3797662	4.075848628	0.740664493	2.10E-08	1.83E-07
Sparcl1	46.78317022	6.654295351	1.33233663	2.16E-08	1.89E-07
Gm9844	118.5485654	6.880603531	2.208200567	2.18E-08	1.90E-07
Ccnb1	77.85568271	7.549456781	2.374418192	2.21E-08	1.93E-07
Slc25a27	153.1267644	2.034625069	0.383336508	2.24E-08	1.96E-07
Grhl1	84.43651385	1.737734247	0.328173595	2.29E-08	2.00E-07
Hbegf	1056.484044	3.500870582	0.687566095	2.32E-08	2.02E-07
Sema4c	375.3262674	2.15164622	0.404274078	2.36E-08	2.05E-07
Bmp8b	48.836341	2.901572583	0.54400262	2.38E-08	2.07E-07
Ltbp3	2784.312219	3.151670151	0.588911876	2.45E-08	2.12E-07
Ust	125.2792792	6.794974917	2.19431097	2.48E-08	2.14E-07
Chml	189.291024	1.095073286	0.20305718	2.55E-08	2.19E-07
Samd10	337.0175874	1.210618677	0.222325821	2.60E-08	2.24E-07
Ptgds	115.6210816	2.289328045	0.436580625	2.69E-08	2.31E-07
Incenp	238.8731454	1.739904053	0.330862405	2.73E-08	2.34E-07
Mllt3	244.0864129	1.223670007	0.228955269	2.73E-08	2.34E-07
2700081O15Rik	611.4329317	5.404332328	0.943055019	2.75E-08	2.36E-07
Mansc1	327.2258063	1.439937087	0.270636576	2.81E-08	2.41E-07
Slc51b	20.9999369	4.21211864	0.782381579	2.81E-08	2.41E-07
Hmmr	76.57121725	4.896085568	0.902497931	2.83E-08	2.42E-07
Abcc9	115.2394672	2.316644214	0.444226049	3.11E-08	2.65E-07

Cmtm7	121.3594006	2.676241441	0.509864618	3.25E-08	2.75E-07
Kif22	82.95101749	2.361109716	0.452050077	3.26E-08	2.76E-07
Junb	9983.881613	1.043911932	0.19594229	3.41E-08	2.88E-07
Prrg1	122.3684907	1.760140088	0.333345758	3.41E-08	2.89E-07
Tsku	2146.539779	1.636502397	0.313448814	3.58E-08	3.02E-07
Adamts14	236.3056715	4.401431761	0.798145314	3.88E-08	3.25E-07
Adamts2	80.26977386	3.413832767	0.65656357	4.02E-08	3.37E-07
Loxl1	116.9155828	6.885479939	2.238636539	4.13E-08	3.44E-07
Lrrc1	276.4700916	2.802733493	0.532418406	4.63E-08	3.84E-07
Ckap2l	65.8260631	6.896433525	2.266351276	4.83E-08	3.99E-07
Dnase1l1	162.9690528	1.02476575	0.193101733	5.04E-08	4.15E-07
Mmp2	40.60420078	4.557750416	0.896028938	5.16E-08	4.24E-07
Gm50475	47.08808995	2.819677072	0.556813122	5.33E-08	4.38E-07
Olfr1372-ps1	1136.60357	3.569965703	0.678724906	5.56E-08	4.55E-07
Mmp24	104.7642708	6.685015543	2.208798232	5.88E-08	4.80E-07
Gins2	77.07097145	3.250999347	0.621942447	6.02E-08	4.90E-07
Mvb12b	509.0379139	1.136112925	0.216262508	6.12E-08	4.97E-07
Klf4	96.95380269	4.486096548	0.83764774	6.12E-08	4.97E-07
Capn5	751.634702	1.105607076	0.21139209	6.13E-08	4.97E-07
Areg	17.28864923	3.531640551	0.69518728	6.17E-08	5.01E-07
Ptpre	506.8055087	1.368809114	0.264388226	6.27E-08	5.08E-07
Ifnlr1	119.579872	4.333658598	0.802091167	6.51E-08	5.26E-07
Necap2	440.021486	1.049215578	0.200135253	6.58E-08	5.31E-07
Pfkfb2	390.6373624	1.134220401	0.217458614	6.63E-08	5.34E-07
Ccna2	142.806624	3.066567531	0.614280122	6.95E-08	5.58E-07
Il1rn	1510.476814	1.433600222	0.278876903	7.27E-08	5.83E-07
Map2k6	324.2190256	1.035894431	0.199252118	7.55E-08	6.04E-07
Lamc2	1703.188094	6.429480123	2.104919827	8.17E-08	6.52E-07
Eif4ebp1	774.164529	1.182089283	0.22898844	8.74E-08	6.97E-07
Mapk4	63.89398407	2.207414274	0.436301932	9.37E-08	7.44E-07
Gm609	1525.888356	5.20713071	0.953000926	9.39E-08	7.45E-07
Snhg18	191.3589224	2.288502093	0.451047654	9.45E-08	7.50E-07
Cx3cr1	20.23214343	4.665202892	0.89969161	9.47E-08	7.51E-07
Dcbld1	251.2185711	1.187531944	0.230675045	9.53E-08	7.55E-07
Diaph3	65.97491546	3.338215741	0.662727536	9.60E-08	7.60E-07
Gm13387	100.6394888	6.518476087	2.168480682	9.64E-08	7.63E-07
Zbtb12	225.3564426	1.3595333	0.266619331	9.73E-08	7.69E-07
Igfbp7	7378.973716	1.60169106	0.319100854	9.91E-08	7.82E-07
Gm31522	31.12292373	2.482419309	0.50429319	1.04E-07	8.16E-07
Kif20a	89.27884143	2.387981151	0.481072953	1.08E-07	8.52E-07
4933431E20Rik	482.1316081	1.006471041	0.19517724	1.11E-07	8.70E-07
Mogat2	53.67526517	2.310425265	0.465751729	1.13E-07	8.85E-07
Slc24a3	37.75884176	4.365768009	0.833642101	1.13E-07	8.86E-07

Ephb2	132.6122714	6.540414218	2.204565122	1.13E-07	8.89E-07
Septin8	893.075375	2.770334848	0.55304064	1.16E-07	9.07E-07
Adamtsl2	34.3794196	5.608688058	1.071589266	1.27E-07	9.85E-07
Prc1	183.748832	4.208209551	0.866441091	1.29E-07	1.00E-06
Dusp5	545.1608575	4.613140124	0.984041488	1.31E-07	1.01E-06
Bcl2	255.0492226	5.113148082	0.945954379	1.32E-07	1.02E-06
Gm42679	48.17681847	6.694781277	2.263129577	1.34E-07	1.04E-06
Pbx4	56.05103632	2.164990974	0.438871015	1.34E-07	1.04E-06
Serpinb6b	1144.125425	2.220252349	0.457930102	1.40E-07	1.09E-06
Kif20b	118.4769305	2.486285275	0.506859302	1.43E-07	1.11E-06
Ttc12	183.9360617	2.670952844	0.528140216	1.43E-07	1.11E-06
Sema5a	1725.479122	5.154471542	0.96545607	1.53E-07	1.18E-06
Fbxw17	162.9102618	5.053957283	0.937555151	1.53E-07	1.18E-06
Gm49405	56.69017637	2.280571317	0.458809115	1.54E-07	1.19E-06
Mis18a	82.19128069	1.473937871	0.294718737	1.56E-07	1.20E-06
Fgr	115.1481992	2.023874308	0.407295338	1.61E-07	1.24E-06
Parm1	343.1141576	5.032063006	0.93475696	1.63E-07	1.26E-06
Syn2	50.11048499	6.563305452	2.247170584	1.69E-07	1.29E-06
Ccl7	119.4539432	6.641701519	2.237663793	1.69E-07	1.29E-06
Inpp5j	66.75223651	5.751795815	1.230111497	1.76E-07	1.34E-06
Crym	115.0287656	1.45736888	0.293844989	1.86E-07	1.42E-06
Calml4	19.65055435	6.6560405	2.299605264	1.87E-07	1.42E-06
Rasgrp3	69.87697963	1.929287025	0.394001144	1.90E-07	1.44E-06
Card14	29.6980435	3.18483262	0.640362849	1.90E-07	1.45E-06
Tmem159	169.569811	1.495855019	0.301108491	1.92E-07	1.46E-06
A930004J17Rik	117.7716395	1.115000009	0.22184277	1.93E-07	1.46E-06
Bex2	29.23493238	4.040013231	0.812836954	1.96E-07	1.48E-06
Tmem253	45.00653487	1.84941934	0.378567127	1.98E-07	1.50E-06
Iqgap3	168.6417664	6.277448294	1.369790988	1.99E-07	1.51E-06
Il17rd	376.2844544	2.837973601	0.568774882	2.00E-07	1.52E-06
Rpl38	1555.587897	1.051516486	0.209597223	2.12E-07	1.59E-06
Ninl	391.144416	1.749573211	0.354556917	2.15E-07	1.62E-06
Kifc5b	55.79256431	2.777947365	0.573442202	2.21E-07	1.66E-06
Slc52a3	240.9556715	4.977133505	0.937232689	2.24E-07	1.68E-06
Slc7a1	304.9468346	3.635141273	0.717820085	2.26E-07	1.69E-06
2310034O05Rik	18.68221474	3.667325731	0.766960857	2.27E-07	1.70E-06
Kctd10	489.3811112	1.155985559	0.232497665	2.33E-07	1.74E-06
Nmrk1	366.0884245	1.180117305	0.237927252	2.36E-07	1.76E-06
Il7	63.06247507	2.840581078	0.574013927	2.41E-07	1.80E-06
Igf2bp2	124.9533154	1.784463775	0.369256773	2.49E-07	1.85E-06
Rad51	87.02254047	2.76109372	0.575642957	2.49E-07	1.85E-06
Ifit2	209.9903753	1.201110615	0.242340531	2.72E-07	2.01E-06
Mpp2	194.2380619	6.269401943	2.156360812	2.78E-07	2.05E-06

Tceal5	37.35941911	3.841812315	0.780259814	2.91E-07	2.15E-06
Gm38357	39.04917212	3.415816085	0.735360887	2.92E-07	2.15E-06
Nudt18	428.7179947	1.098812179	0.222137141	2.92E-07	2.15E-06
Kif4	58.56447958	6.73298051	2.314255068	3.05E-07	2.24E-06
Sirt6	243.4401286	1.169449432	0.237695918	3.15E-07	2.31E-06
Paqr4	174.4009437	2.456454152	0.502182911	3.16E-07	2.32E-06
Gspt2	109.016343	1.635850026	0.336359836	3.20E-07	2.34E-06
Flvcr2	109.0095871	1.571334406	0.327149548	3.21E-07	2.35E-06
Olfml3	24.33583492	5.85521851	1.295637154	3.23E-07	2.36E-06
Pdzk1ip1	816.5625522	1.273689167	0.26107294	3.23E-07	2.36E-06
Plcd3	694.4379766	4.926945034	0.944047585	3.24E-07	2.36E-06
Stambpl1	107.1529584	2.612150789	0.541877776	3.33E-07	2.43E-06
Coro6	147.3621	5.604309865	1.22864057	3.38E-07	2.46E-06
Rasgrf2	28.76391353	3.235811292	0.671442935	3.44E-07	2.50E-06
Cnbd2	79.13870619	3.313549832	0.705481195	3.47E-07	2.52E-06
Eid2	118.0403526	2.594163664	0.541707494	3.49E-07	2.53E-06
Prelid2	35.53598776	3.382396595	0.710092059	3.67E-07	2.66E-06
Thbs2	26.43254022	6.446489606	2.26873503	3.76E-07	2.72E-06
Cd63-ps	16.8973978	6.551076311	2.30878993	3.87E-07	2.80E-06
Ddah2	682.6662607	4.029150719	0.802094475	4.08E-07	2.93E-06
Dlgap5	59.25108218	6.654183735	2.307358095	4.13E-07	2.97E-06
Mroh3	28.90483728	6.419422108	2.262702434	4.44E-07	3.18E-06
Dcdc2a	2247.349072	1.811259525	0.378209745	4.59E-07	3.28E-06
Cbx6	2013.689666	1.752295449	0.369299044	4.63E-07	3.31E-06
Dok4	84.57630924	2.768796871	0.575652295	4.65E-07	3.32E-06
Tctn3	174.3446301	1.490256308	0.310599701	4.89E-07	3.48E-06
Pamr1	2420.952237	5.401551386	1.19219528	4.92E-07	3.50E-06
Cpxm1	14.83367858	6.379284805	2.282021712	4.99E-07	3.54E-06
Pdgfrb	31.44606993	5.915185109	1.340853409	5.05E-07	3.58E-06
Trim59	100.6685281	6.065686221	2.15431686	5.24E-07	3.71E-06
AU020206	151.1958078	1.339263899	0.279793567	5.46E-07	3.86E-06
Adgrg2	198.8521732	2.974046135	0.621116993	5.47E-07	3.86E-06
Cacna1b	17.66387989	4.255074816	0.874437255	5.48E-07	3.87E-06
Bok	865.7545512	1.217387136	0.254629066	5.51E-07	3.89E-06
Cyb561	990.9136657	1.034203274	0.214086187	5.52E-07	3.90E-06
Hells	177.1201959	1.995949466	0.432591898	5.54E-07	3.91E-06
Gm42928	45.85838748	3.263115795	0.696916957	5.63E-07	3.96E-06
H2-T10	114.2765341	1.1108225	0.230397459	5.63E-07	3.96E-06
Erf	1483.739293	1.002092594	0.20755973	5.64E-07	3.97E-06
Cdkn3	24.09208298	5.86145715	1.334629818	5.67E-07	3.99E-06
Cers5	280.2544323	1.947577649	0.412726689	5.74E-07	4.04E-06
Scn8a	226.5834904	1.617171334	0.345467546	5.76E-07	4.05E-06
Fam107a	234.8449596	4.118637166	0.914796051	5.79E-07	4.07E-06

Nucb2	1194.176929	1.051702334	0.218526308	5.85E-07	4.10E-06
Plekhh3	372.2277056	1.007746427	0.208508856	5.89E-07	4.13E-06
Spice1	247.1774444	1.1462832	0.239579389	5.94E-07	4.16E-06
Mrgbp	256.963942	1.000226856	0.207514735	6.03E-07	4.22E-06
Ikbip	283.2262314	1.031158333	0.213518722	6.09E-07	4.25E-06
Col5a2	41.54715246	2.724628788	0.601872366	6.12E-07	4.27E-06
Cenpf	188.2855359	6.762917018	2.325610033	6.15E-07	4.30E-06
Camk2n2	81.7164672	1.727503465	0.371731598	6.19E-07	4.32E-06
Cd24a	7914.647318	5.420387893	1.213440278	6.36E-07	4.43E-06
Gm30177	32.55434467	2.166411947	0.47332882	6.39E-07	4.45E-06
Guca2a	14.58698859	6.498904635	2.325196717	6.48E-07	4.51E-06
Rpl38-ps2	91.22699044	1.310724941	0.277131912	6.58E-07	4.57E-06
Gm43361	47.76247661	3.265722338	0.734726105	6.61E-07	4.59E-06
Ica1	157.4927787	1.385839708	0.293632005	6.64E-07	4.61E-06
Hddc2	112.5066194	1.748588671	0.37428144	6.64E-07	4.61E-06
Nqo1	307.819888	1.008314025	0.209836857	6.95E-07	4.80E-06
Basp1	1021.738777	6.838959823	2.313480943	7.20E-07	4.95E-06
Taf1c	170.1973506	1.348845142	0.285836701	7.24E-07	4.98E-06
Misp	318.2898526	1.780824828	0.378770999	7.24E-07	4.98E-06
Cavin3	114.724337	5.967181179	2.141662243	7.57E-07	5.20E-06
Mgp	11.72916205	6.7965482	2.427076831	7.59E-07	5.21E-06
Ctxn1	427.1688268	3.091021297	0.647899757	7.68E-07	5.27E-06
AC110211.1	550.5848049	1.11764698	0.235624651	7.70E-07	5.28E-06
Zscan2	273.3660954	1.08074573	0.226834848	7.70E-07	5.28E-06
Hspa1a	340.0292337	2.084041139	0.454571605	7.91E-07	5.41E-06
Rrm2	220.1373501	2.089879035	0.463281777	7.92E-07	5.41E-06
Tedc1	70.58738618	6.095145459	2.189290356	7.95E-07	5.43E-06
Spns3	97.22993108	4.776205028	0.955147743	8.04E-07	5.48E-06
Fry	397.0166883	1.070835555	0.225811948	8.55E-07	5.81E-06
Mamld1	384.3744535	5.826664467	2.085372876	8.57E-07	5.82E-06
Kcnb2	14.68221559	6.393320019	2.349520465	8.72E-07	5.92E-06
Mid1	149.4161712	1.252899593	0.26654996	9.19E-07	6.22E-06
Clba1	95.96405526	1.560875032	0.337700109	9.22E-07	6.23E-06
Poc1a	62.45623405	1.326340548	0.284795279	9.55E-07	6.44E-06
Ddit3	209.6482995	1.680055387	0.369326999	9.67E-07	6.51E-06
Vldlr	740.5016774	1.116832651	0.237410609	9.76E-07	6.56E-06
Zfp469	396.8982863	5.797120226	2.087195056	9.88E-07	6.63E-06
Odad1	306.2584663	3.895122241	0.809089279	1.01E-06	6.79E-06
Tgfb1	235.723318	1.451585123	0.313268444	1.02E-06	6.82E-06
Ltb	613.3930778	4.036881911	0.84881222	1.02E-06	6.84E-06
Gm13751	9.869464431	6.697344673	2.451626066	1.05E-06	7.04E-06
Gm14964	9.575115214	6.589986639	2.416964692	1.09E-06	7.30E-06
Zfp429	81.41093935	1.539041199	0.334393758	1.11E-06	7.42E-06

Gm44639	19.74551052	6.416915824	2.324711174	1.12E-06	7.47E-06
1110051M20Rik	220.5302099	1.237421897	0.266716907	1.13E-06	7.54E-06
Magohb	78.62208101	1.52193291	0.333265885	1.18E-06	7.81E-06
Tekt5	15.40230972	4.858936624	1.005117822	1.18E-06	7.85E-06
Lum	43.07692162	3.899092423	0.898997318	1.19E-06	7.88E-06
Gm36283	60.01923427	1.728347274	0.381465863	1.19E-06	7.88E-06
Gm37422	70.97964144	1.57656986	0.345159407	1.22E-06	8.07E-06
Gas2l3	197.406415	1.644034387	0.364375961	1.30E-06	8.52E-06
Veph1	205.4741235	1.694207912	0.37629996	1.42E-06	9.27E-06
Clstn1	1488.206091	2.589768745	0.564592946	1.43E-06	9.35E-06
Ccdc68	72.84594249	1.886774027	0.418269057	1.46E-06	9.48E-06
Eda	64.36601547	1.289054945	0.281437421	1.48E-06	9.61E-06
Plet1os	77.76262449	2.339742726	0.524608878	1.48E-06	9.63E-06
Gm26532	19.68934037	6.280488859	2.30132031	1.53E-06	9.94E-06
Rims2	147.3124243	4.091503655	0.889158316	1.56E-06	1.01E-05
Epb41l4a	490.01875	5.641944996	2.065620117	1.56E-06	1.01E-05
Enkd1	98.5784875	2.487150405	0.548799566	1.59E-06	1.03E-05
B9d1	123.0617653	2.220110217	0.489236412	1.59E-06	1.03E-05
Kcnq1ot1	171.0707925	1.528452193	0.340905242	1.60E-06	1.03E-05
Gm37352	23.01723207	6.381250512	2.331581613	1.65E-06	1.07E-05
Nin	271.069687	1.061500496	0.229890538	1.66E-06	1.07E-05
Gm43360	32.79507819	2.742360944	0.634135996	1.66E-06	1.07E-05
Gm45457	158.8485913	1.750143389	0.39452483	1.69E-06	1.09E-05
Ercc1	171.0147798	1.025928234	0.221773755	1.69E-06	1.09E-05
Trim62	91.27831857	1.598035053	0.356366523	1.70E-06	1.09E-05
9530077C05Rik	113.1858101	3.353490793	0.725504085	1.71E-06	1.10E-05
Shisa4	490.9216844	5.60012464	2.059433666	1.78E-06	1.14E-05
Hcar2	54.58956443	1.67547259	0.378554186	1.82E-06	1.16E-05
Creb5	259.0509532	5.669887973	2.092213195	1.85E-06	1.18E-05
Psd	180.1177899	1.794823721	0.406695231	1.86E-06	1.19E-05
Krt5	7.169073494	6.311278124	2.398361671	2.00E-06	1.27E-05
Oxct1	696.4409434	1.848885966	0.416536721	2.01E-06	1.28E-05
1500011B03Rik	110.7294255	2.003541958	0.452573774	2.03E-06	1.29E-05
Trim24	1029.7912	1.140546694	0.251757772	2.07E-06	1.31E-05
Plk1	69.1291971	4.937527612	1.067104272	2.08E-06	1.31E-05
Airn	113.8688522	1.829342446	0.413913847	2.14E-06	1.35E-05
Fam83d	43.90729814	6.086997123	2.258090991	2.18E-06	1.37E-05
Fkbp5	1265.795796	1.198004249	0.266126602	2.19E-06	1.38E-05
Atn1	147.1075151	1.239309075	0.275592724	2.20E-06	1.38E-05
Eid3	18.31802696	2.626024859	0.606745182	2.25E-06	1.41E-05
Btbd19	44.23332552	1.901600389	0.439437317	2.27E-06	1.42E-05
Gpat3	142.7667544	1.012571205	0.222793909	2.30E-06	1.44E-05
Frmd4a	522.0092215	1.567292154	0.356219409	2.39E-06	1.49E-05

Ank1	96.47599374	5.677405476	2.119114902	2.40E-06	1.50E-05
Psat1	122.1402971	1.763176857	0.402941759	2.50E-06	1.56E-05
Bicd1	254.2985166	5.541822658	2.07840897	2.55E-06	1.58E-05
Foxj1	1158.630639	5.533364813	2.048482894	2.55E-06	1.59E-05
Gm36638	17.66924104	3.381081098	0.776750737	2.67E-06	1.65E-05
Cdca8	74.4217961	1.780856938	0.40870706	2.67E-06	1.65E-05
Lrmda	28.40672573	2.452215131	0.57592115	2.69E-06	1.66E-05
Dab1	428.2911511	4.528700013	0.962067532	2.76E-06	1.70E-05
Krt23	775.5902267	2.427149613	0.579895225	2.77E-06	1.71E-05
Smim3	170.244785	1.632552605	0.371832846	2.81E-06	1.74E-05
Cxcl1	1410.42543	1.197292228	0.269265261	2.84E-06	1.75E-05
Ager	70.79660825	5.597680676	2.126525825	2.86E-06	1.76E-05
Fignl1	63.27404966	3.359627027	0.790860909	2.92E-06	1.80E-05
Cyp2b10	495.0599361	2.708062491	0.654499787	2.99E-06	1.84E-05
Krtcap3	182.9199105	1.452092376	0.327944798	3.00E-06	1.84E-05
Lmntd2	142.4642597	1.212896734	0.273353252	3.04E-06	1.86E-05
Cd200	290.627671	5.482876447	2.062716617	3.05E-06	1.87E-05
Meltf	168.9532373	5.187468139	1.267902244	3.05E-06	1.87E-05
Bub1	40.9483308	5.757220829	2.19311505	3.09E-06	1.89E-05
Dzip1l	765.3826729	4.424719531	0.939359696	3.15E-06	1.92E-05
Ier5l	202.692531	4.464321126	0.954128976	3.20E-06	1.96E-05
Pbk	51.82096269	6.147589683	2.302068933	3.27E-06	1.99E-05
Coprs	78.59748785	1.402646686	0.320755881	3.29E-06	2.00E-05
Spag5	98.07731213	2.235902853	0.53164572	3.30E-06	2.01E-05
Rnase1	18.03063134	6.219351567	2.344416269	3.30E-06	2.01E-05
Plch1	155.1805093	4.946187649	1.199196144	3.30E-06	2.01E-05
Aoc2	78.73395579	1.727015238	0.397935032	3.31E-06	2.01E-05
Sgcb	72.74187539	2.074149292	0.477652411	3.42E-06	2.08E-05
A230083G16Rik	84.11065141	1.26034423	0.28723823	3.44E-06	2.09E-05
Adamtsl5	21.26029068	3.281355351	0.763390627	3.45E-06	2.09E-05
Timp4	11.93167421	6.215124545	2.360865337	3.64E-06	2.20E-05
Mab21l4	94.28698676	1.830515728	0.425798664	3.67E-06	2.22E-05
Plk3	1881.600877	1.327117813	0.305047741	3.77E-06	2.27E-05
Thbs1	3384.490382	4.78680403	1.159180156	3.80E-06	2.28E-05
Neurl1b	148.6441284	5.199983919	1.202959658	3.99E-06	2.39E-05
Gata3	18.3510448	3.735672986	0.879099884	4.07E-06	2.43E-05
Gm49327	73.64457218	1.714605192	0.399102674	4.15E-06	2.48E-05
Arhgef6	72.6600184	2.292753299	0.544965387	4.36E-06	2.60E-05
Srd5a2	311.6276207	1.069747558	0.243248271	4.37E-06	2.60E-05
Cdc6	46.05270897	5.8418265	2.235959205	4.39E-06	2.61E-05
Apela	11.29400117	6.787019728	2.598543123	4.40E-06	2.61E-05
Dtl	105.5778503	2.673504057	0.64577672	4.40E-06	2.62E-05
Cidec	51.46699202	1.647814473	0.388464468	4.42E-06	2.63E-05

Ska2	113.6722477	1.75947524	0.409665741	4.56E-06	2.70E-05
Thsd7a	160.8625997	5.488184273	2.10004551	4.59E-06	2.72E-05
Arid5a	221.6635287	1.718997979	0.407209952	4.59E-06	2.72E-05
Gpc3	30.3801853	5.1871583	1.307265221	4.82E-06	2.84E-05
Opn3	29.28124358	1.982829834	0.471492462	4.86E-06	2.86E-05
Gpsm1	252.0806411	4.332374266	0.941202921	4.87E-06	2.87E-05
Itgb3	33.89830086	4.276056598	1.050663403	4.96E-06	2.92E-05
Grip1	88.74446334	2.768498687	0.64962761	4.99E-06	2.93E-05
Tmod1	116.5443777	5.466932369	2.117613015	5.03E-06	2.95E-05
Tmem132a	566.3561289	3.116685412	0.708552135	5.11E-06	3.00E-05
Tnfrsf11a	85.1337696	3.656744739	0.827062702	5.11E-06	3.00E-05
Nradd	310.3414058	1.520342326	0.354692646	5.26E-06	3.08E-05
AC156638.1	14.4341119	2.901294846	0.69329885	5.81E-06	3.38E-05
Slc1a1	42.56718334	4.976498462	1.259949482	5.86E-06	3.41E-05
Prkab2	176.5981521	1.114822692	0.256824197	5.88E-06	3.42E-05
Kbtbd8	41.077784	2.097666941	0.51306654	5.95E-06	3.45E-05
Ltbp4	477.6364358	1.460915624	0.347990697	6.03E-06	3.50E-05
Nusap1	103.2291263	1.865363607	0.449285706	6.15E-06	3.56E-05
Myo5a	131.6653019	1.783379346	0.421179515	6.21E-06	3.59E-05
Fgd3	516.9420966	4.232429063	0.928299947	6.31E-06	3.65E-05
Gm31084	64.30040656	1.277049089	0.298612945	6.44E-06	3.72E-05
Clspn	67.72578872	6.453016153	2.426119804	6.49E-06	3.74E-05
Gm42929	51.01133385	1.983599048	0.484041624	6.50E-06	3.75E-05
Pkia	312.5624011	4.228968874	0.930553335	6.58E-06	3.79E-05
Cyp2c55	164.3975735	1.469794629	0.351862437	6.69E-06	3.85E-05
Kazn	95.36966977	2.619690718	0.618477668	6.83E-06	3.93E-05
Krt20	69.46770226	5.785304751	2.240382092	6.83E-06	3.93E-05
Cldn23	84.22403011	2.926135344	0.693831376	6.91E-06	3.97E-05
Pdgfra	23.43576109	4.035270894	0.968256176	7.04E-06	4.03E-05
Tmem45b	118.8257564	4.809373722	1.220847869	7.04E-06	4.03E-05
Stil	69.22256311	4.325037761	0.969454336	7.11E-06	4.07E-05
Clca3a1	42.1005888	3.518865187	0.850935009	7.19E-06	4.11E-05
Rad54l	52.05831429	5.466114035	2.160300864	7.50E-06	4.28E-05
Tppp3	90.84479318	5.336603104	2.107607969	7.58E-06	4.32E-05
Slc9a7	104.8055513	1.648736613	0.397363235	7.69E-06	4.38E-05
Tmem51	317.5507215	1.090466088	0.256893616	7.77E-06	4.43E-05
Ptgfr	404.575504	4.777509347	1.217560493	7.79E-06	4.44E-05
Tcf19	209.2972139	1.482470437	0.35842119	7.81E-06	4.45E-05
H2ax	330.2827875	1.029131497	0.240937524	7.87E-06	4.47E-05
Gm43980	118.8122645	1.551092057	0.371003283	8.04E-06	4.56E-05
Ntf3	25.71582979	5.525211208	1.464831714	8.08E-06	4.58E-05
Tonsl	222.5322796	1.08891905	0.256166782	8.16E-06	4.62E-05
Kif3c	322.9603925	2.223669519	0.53085084	8.27E-06	4.68E-05

Plod2	169.7300511	5.357262671	2.118261367	8.47E-06	4.78E-05
Hmgcll1	64.0633418	5.272578315	2.103657536	8.55E-06	4.82E-05
Zfp40	108.3734807	1.0450924	0.24544268	8.69E-06	4.89E-05
Snhg12	158.070228	1.064533015	0.250861354	8.80E-06	4.95E-05
Gm47528	13.4638242	2.696997802	0.680743312	8.93E-06	5.02E-05
Spata6	279.0047109	4.219084762	0.953543084	8.98E-06	5.04E-05
Dpt	12.71434071	6.370245147	2.470051311	9.01E-06	5.06E-05
Tpbgl	34.86131448	1.605213787	0.392277194	9.08E-06	5.09E-05
Aebp1	176.1054284	3.022040163	0.74066663	9.26E-06	5.18E-05
Ap1s2	23.28046215	2.313877289	0.566612724	9.34E-06	5.22E-05
Gm42793	566.2139603	5.103539087	2.021151174	9.39E-06	5.25E-05
Sox9	3447.10055	1.129306224	0.269384333	9.64E-06	5.38E-05
Slc41a3	133.3096149	1.748075087	0.434720101	9.69E-06	5.41E-05
Wdr72	204.2332789	5.186832959	2.071526921	9.81E-06	5.47E-05
Fkbp1b	31.87372976	5.281791428	2.129185071	9.84E-06	5.48E-05
Zfp599	94.56903607	2.553227487	0.614727607	9.89E-06	5.51E-05
Scube1	10.99974653	5.900974115	2.345862511	1.01E-05	5.60E-05
Cacna2d4	15.39788686	3.668014638	0.87700017	1.02E-05	5.67E-05
Chaf1b	172.7907734	1.470577803	0.35979666	1.03E-05	5.73E-05
Il20rb	44.7960398	2.666399297	0.671588238	1.04E-05	5.79E-05
Tbx1	580.1924842	6.52604175	2.453957738	1.05E-05	5.80E-05
Pde10a	38.64573954	3.648433073	0.872933957	1.05E-05	5.84E-05
Fam171b	48.01591245	5.379853316	2.162141477	1.07E-05	5.90E-05
Lox	8.969308205	5.62088054	2.274016058	1.08E-05	5.96E-05
Egflam	1011.28862	5.07215619	2.013230905	1.08E-05	5.97E-05
Tmem54	68.25101052	5.288468257	2.141059525	1.10E-05	6.07E-05
Ets1	789.878056	1.189127219	0.286188242	1.11E-05	6.12E-05
Mtcl1	872.6430068	5.033686639	2.006110637	1.12E-05	6.19E-05
Fchsd1	471.3226952	1.490974401	0.366693345	1.13E-05	6.21E-05
Adam15	279.7673447	2.214774758	0.53884825	1.13E-05	6.23E-05
Mok	55.98178475	5.165339038	2.088991851	1.13E-05	6.24E-05
Vil1	365.724313	4.131215971	0.94091626	1.14E-05	6.29E-05
Ube2c	73.83491087	3.036677985	0.78796265	1.15E-05	6.30E-05
Dynlt1f	121.9569769	1.819596348	0.451013275	1.15E-05	6.31E-05
Gm16011	838.1269171	5.073602197	2.020593866	1.15E-05	6.31E-05
Rad18	107.0307597	1.325354069	0.322977409	1.15E-05	6.33E-05
Nebl	109.6906408	2.759198772	0.668466792	1.16E-05	6.34E-05
Cfap54	86.12145659	3.073273383	0.740709761	1.17E-05	6.40E-05
Slco1a6	35.08598132	5.291378177	2.145761021	1.17E-05	6.41E-05
Tnfsf12	179.1421571	1.234735425	0.299911553	1.19E-05	6.48E-05
Gm44950	93.05090018	1.242152584	0.301032642	1.22E-05	6.68E-05
Mamdc4	65.2279345	1.639664944	0.408687175	1.23E-05	6.73E-05
Cib3	873.5887824	1.053118916	0.253590018	1.24E-05	6.74E-05

Atp2b2	133.9032802	1.104102488	0.266975815	1.27E-05	6.93E-05
Ncapg	58.65207334	3.015850385	0.769338174	1.28E-05	6.94E-05
Usp11	325.6033467	1.596630077	0.393344884	1.28E-05	6.95E-05
Sh2d4b	143.4454641	5.0710651	2.048795179	1.29E-05	6.98E-05
Gm17024	91.74932521	2.596917498	0.684850091	1.32E-05	7.15E-05
Gm42937	35.817212	2.671703941	0.6979712	1.32E-05	7.17E-05
4930579G18Rik	93.24855243	1.347684514	0.330137212	1.33E-05	7.21E-05
Gpr153	21.26597132	5.332790754	2.180003004	1.35E-05	7.31E-05
Spata24	46.40203174	1.929925115	0.484015135	1.38E-05	7.42E-05
Ntrk2	431.6986098	2.775914337	0.744290111	1.40E-05	7.53E-05
Ccdc18	66.93967832	2.714133786	0.664223547	1.42E-05	7.62E-05
Upk3b	9.530091929	5.441419047	2.236923301	1.42E-05	7.63E-05
Usp2	403.8005691	2.67823653	0.715361082	1.43E-05	7.66E-05
Wdr62	79.69927961	1.084292711	0.262966596	1.46E-05	7.84E-05
Fam241a	77.1853476	1.202865165	0.294844694	1.47E-05	7.88E-05
Ntn1	103.2084884	2.815578033	0.699934569	1.48E-05	7.90E-05
Bub1b	113.7057321	2.559407749	0.656292665	1.49E-05	7.96E-05
Noct	758.1771916	2.099979863	0.549172296	1.51E-05	8.04E-05
Tmc5	43.86023151	5.316177924	2.176321236	1.52E-05	8.10E-05
Hcn2	58.37663793	1.660608709	0.417106609	1.52E-05	8.13E-05
Atf3	3243.267135	3.501087967	0.970810065	1.53E-05	8.18E-05
Ccdc3	8.914326112	5.426077422	2.242072199	1.53E-05	8.18E-05
Ankrd55	8.762867722	4.851581873	1.312593033	1.55E-05	8.26E-05
Tnfsfm13	29.82880007	2.48014418	0.63925822	1.55E-05	8.27E-05
Gm16091	37.80693799	4.226320648	1.005008673	1.58E-05	8.42E-05
Gm49590	37.39943702	2.988705338	0.759187315	1.60E-05	8.49E-05
Nup62cl	19.01550381	2.968428803	0.793025116	1.63E-05	8.65E-05
Rgl1	100.54401	1.389878005	0.347811371	1.68E-05	8.91E-05
Crocc	215.0210224	1.028986459	0.250512513	1.70E-05	8.98E-05
Napepld	101.6244337	1.103767036	0.270662463	1.70E-05	8.98E-05
Nupr1	99.07289363	2.748898562	0.68474329	1.72E-05	9.05E-05
Pkhd1	4275.810134	1.034782472	0.253481084	1.73E-05	9.12E-05
Gm43481	75.31890678	1.427438807	0.357094666	1.74E-05	9.16E-05
Zfp248	87.29342852	1.519682955	0.380375953	1.75E-05	9.24E-05
Melk	62.82479422	5.104031128	2.116564577	1.81E-05	9.54E-05
Espl1	82.51814812	3.81367336	0.969287739	1.82E-05	9.59E-05
Fmod	9.003728375	5.586354399	2.305855697	1.83E-05	9.62E-05
Phf11d	136.5903438	1.650966081	0.415427875	1.86E-05	9.75E-05
Pask	113.5348826	1.388149427	0.345006105	1.87E-05	9.78E-05
Nek5	134.9824918	2.441587593	0.609011787	1.90E-05	9.95E-05
Cip2a	129.6753678	1.126413764	0.277913706	1.91E-05	0.000100074
Enkur	81.68257409	4.524795494	1.219060433	1.92E-05	0.000100349
Ophn1	153.9617263	1.033753489	0.25303709	1.95E-05	0.000101776

Scara3	5836.559404	4.044353303	0.9613204	1.96E-05	0.000102367
Tmem154	53.94579418	3.644957655	0.917402278	1.98E-05	0.000103097
Arhgap4	82.53477645	1.32805815	0.334767804	1.98E-05	0.000103187
Syn3	79.4794392	1.521475167	0.385892623	2.00E-05	0.000104028
9130017K11Rik	40.95060515	5.074228847	2.119481366	2.06E-05	0.000107217
Trim16	136.1153518	4.954477951	2.072727104	2.15E-05	0.000111121
Fhl1	84.42554324	2.561032531	0.667033763	2.19E-05	0.000113244
Rhpn1	60.97190403	4.944660878	2.073196871	2.22E-05	0.000114649
Rnf180	214.8265372	4.89799181	2.036346935	2.23E-05	0.000115263
2410006H16Rik	204.3508789	1.1891509	0.299241858	2.26E-05	0.000116802
A930033H14Rik	65.19165595	2.453203713	0.667991037	2.29E-05	0.000118047
Emilin1	63.80878955	1.690573447	0.442470521	2.31E-05	0.000119271
H1f5	10.73173961	5.020664461	1.415599102	2.33E-05	0.000120065
Flrt3	300.4607345	4.996895454	2.075754312	2.35E-05	0.000121195
Sh3d21	149.3586842	1.679767542	0.433302031	2.43E-05	0.000124626
Sync	32.68998573	4.174756707	1.028589562	2.44E-05	0.000125305
Cygb	21.58253731	3.302836487	0.886898706	2.50E-05	0.000127977
Cuedc1	613.8840144	2.688356111	0.688076924	2.51E-05	0.000128326
Flt3l	83.04877609	1.178931078	0.298044703	2.51E-05	0.000128597
Anxa3	1721.356436	2.92662012	0.743571103	2.54E-05	0.000130125
Mroh6	20.6931891	1.880626774	0.493719806	2.61E-05	0.000133321
Gm11639	11.88286842	4.213299371	1.050276979	2.66E-05	0.000135508
Nemp2	99.53917871	3.998026528	0.971806659	2.71E-05	0.000137679
Pdp1	126.881524	1.249560049	0.318314001	2.71E-05	0.000137888
Id4	12.35752963	4.724417717	1.233545431	2.72E-05	0.000138392
Atp10d	151.5524357	1.094222404	0.275781901	2.78E-05	0.00014103
Zfp689	93.77918479	1.055554862	0.263872592	2.80E-05	0.000141905
Tcf24	347.3894377	1.386413189	0.359639989	2.88E-05	0.000145331
Aspm	105.6446613	2.523102932	0.684025874	2.95E-05	0.000148729
Msln	683.1667915	4.375112494	1.21165931	3.00E-05	0.000151019
Mbnl3	26.88781988	2.131658328	0.569326604	3.01E-05	0.000151271
Arhgap36	56.66891399	1.69276794	0.445812798	3.01E-05	0.000151366
Efemp2	333.6761589	2.418444235	0.62643162	3.05E-05	0.000153097
Spon1	401.3325519	4.058606435	1.008009381	3.07E-05	0.000153955
Ankrd1	2770.215599	3.639268681	0.968069255	3.08E-05	0.000154746
Adamts15	163.6299297	2.591399636	0.70375158	3.10E-05	0.000155428
Gm19221	41.19545013	1.779400085	0.462743487	3.12E-05	0.000156507
Smim24	22.60151548	2.542057895	0.667556699	3.15E-05	0.000157586
Spindoc	186.573418	1.007092956	0.254006188	3.24E-05	0.000161874
Gm46516	90.23536574	1.527192985	0.404334704	3.24E-05	0.000161939
Azin2	115.3553129	1.662815524	0.441757641	3.32E-05	0.000165312
Selenom	95.91489829	4.812880506	2.062683533	3.38E-05	0.00016831
Gm47950	24.94691639	3.274789592	0.8319668	3.40E-05	0.00016934

Fkbp10	123.6029324	4.805247581	2.067422692	3.70E-05	0.0001834
Palm	760.8906651	1.395376363	0.368843553	3.72E-05	0.00018398
Plxna4	14.42372022	4.561309782	1.309622286	3.75E-05	0.00018555
Cdc25c	11.88814144	5.089224713	2.216911871	3.77E-05	0.000186243
Cdca7l	107.6290371	3.252087819	0.834259485	3.83E-05	0.000189155
Cenpe	89.47404783	4.04798151	1.030247002	3.88E-05	0.000191165
5930420M18Rik	34.9787551	1.91632513	0.514576957	3.89E-05	0.000191608
Gm10030	35.15347142	1.53955992	0.411733163	3.98E-05	0.000195637
Adgra2	71.92095866	1.958842881	0.529359291	4.01E-05	0.000197114
Mcm4	383.1632486	1.148698202	0.298493902	4.04E-05	0.000198439
Oacyl	29.06736327	5.03947488	2.174058113	4.06E-05	0.000199198
Svep1	11.10221346	5.568966754	2.370495912	4.08E-05	0.000199878
Pkm	452.1244789	1.127151066	0.29123535	4.11E-05	0.000201334
Ampd3	249.1452166	2.084586597	0.574765894	4.19E-05	0.000204965
Dcn	140.1508072	2.008684321	0.561717727	4.24E-05	0.000207356
B4galt2	210.7762572	4.639218108	2.006973524	4.34E-05	0.000211628
Ccdc74a	30.78210296	3.042114664	0.816264268	4.37E-05	0.000213118
Fam189b	109.872702	2.513136276	0.659432683	4.38E-05	0.000213358
Cklf	86.76632974	1.888388191	0.50564954	4.52E-05	0.000220038
Magi2	24.37552289	4.862626508	2.127022844	4.54E-05	0.000220581
Dhrs9	150.1315802	1.232848343	0.325281558	4.56E-05	0.000221476
Zdhhc1	360.0125597	1.103380682	0.285249034	4.59E-05	0.000222963
Sapcd2	24.14546453	4.844139792	2.13411354	4.60E-05	0.000223314
Gnb5	138.5540797	4.669622579	2.036292785	4.62E-05	0.00022413
Sod3	498.9041449	1.756315711	0.478948662	4.70E-05	0.000227578
Dbp	1420.647959	1.758304453	0.487625981	4.70E-05	0.000227774
Pclaf	73.92880739	3.447311665	0.967715717	4.70E-05	0.000227825
Cep112	99.60240558	2.784584294	0.731445618	4.75E-05	0.000229687
Gm12498	13.03505867	2.204678947	0.617240854	4.78E-05	0.000231015
Gm16170	103.5760646	1.325275517	0.351746826	5.02E-05	0.000241957
Lrrc39	32.42581701	1.539705894	0.41786769	5.02E-05	0.000242141
Zc3hav1l	191.1381486	3.121407119	0.808182675	5.09E-05	0.000245088
Akr1b10	104.6207861	1.742579447	0.474168298	5.18E-05	0.000249191
Sh3tc1	77.0993071	1.812977071	0.487282658	5.22E-05	0.000250872
Meis3	184.4185266	4.697316273	2.054632015	5.23E-05	0.000251206
Cep85	6002.535814	1.046538291	0.27348913	5.27E-05	0.000253378
Lbh	256.5669059	1.638200518	0.441423655	5.28E-05	0.000253825
Kif18b	46.11416149	5.055764474	2.211184598	5.39E-05	0.000258358
Dusp6	2465.49765	1.038256222	0.272125173	5.43E-05	0.000260244
Mapk8ip1	662.1728763	1.544379543	0.410904713	5.46E-05	0.000261306
Ticrr	34.79120974	5.094563142	2.228538682	5.54E-05	0.000265311
Fa2h	18.45245771	4.919936871	2.178988557	5.55E-05	0.000265679
Gsta5	9.110892409	3.417843344	0.97259663	5.57E-05	0.00026649

Birc5	61.90512381	1.291859272	0.345881608	5.63E-05	0.000269076
Atp10a	571.1271433	2.119111531	0.585367589	5.73E-05	0.000273628
Fanca	103.4861522	1.178477277	0.313035916	5.78E-05	0.000275244
Gadd45a	297.0520095	1.390108355	0.379793351	6.01E-05	0.000285356
Gm13056	26.31396775	3.800867541	0.977123568	6.05E-05	0.000287057
Dusp13	4.426200121	5.318768659	2.369982295	6.07E-05	0.000287613
Gm42941	16.39181064	4.430192874	1.320529003	6.09E-05	0.000288344
Sbsn	49.57013045	2.523571676	0.683723213	6.11E-05	0.000289253
Ttk	33.32754751	4.377312291	1.300585031	6.15E-05	0.000290878
Smim22	101.2672811	1.541051475	0.427776008	6.39E-05	0.000301935
Nuf2	45.99483259	4.505041867	1.35904702	6.61E-05	0.000311198
Ggt1	88.49727745	4.105732854	1.200575257	6.66E-05	0.000313278
Zfp9	119.4940582	1.067545975	0.284897678	6.90E-05	0.000323758
Matn2	28.0783044	4.203870242	1.245819103	6.93E-05	0.000324981
Gm19705	100.0284431	1.332302884	0.365380129	7.18E-05	0.000335841
Adamts5	143.7100283	2.465060951	0.674839633	7.20E-05	0.000336288
Celsr2	469.216707	1.356561622	0.365215887	7.26E-05	0.000338644
Peg13	293.5557734	1.25390951	0.342633138	7.30E-05	0.000340279
Klc3	225.384967	3.671962271	0.949307687	7.35E-05	0.000342356
Frzb	7.619930775	5.514693076	2.435586588	7.41E-05	0.000345216
Gm45540	29.42838719	1.696265539	0.4791364	7.44E-05	0.000346286
Ect2	151.2672817	1.475188691	0.411805496	7.57E-05	0.000352079
Bambi	173.723106	1.102851415	0.296841063	7.58E-05	0.000352463
Lockd	11.66896182	4.947936464	2.235588734	7.60E-05	0.000353204
Gm9821	113.6853601	2.047164678	0.570309258	7.70E-05	0.000357518
Col8a1	6.672028532	5.198338922	2.333610459	7.70E-05	0.000357766
Marcks	2298.459478	1.283776152	0.349639126	7.73E-05	0.000358898
BC016579	79.32058624	2.759563182	0.757081436	7.73E-05	0.000358898
Cdca5	31.9065435	2.790616995	0.796107992	7.80E-05	0.000361704
Snn	228.0071003	2.700528781	0.742194115	8.30E-05	0.000382974
Notch3	20.13278178	4.334382087	1.325395124	8.34E-05	0.000384936
Sulf1	14.0474772	5.212491051	2.337376205	8.60E-05	0.000396015
Tcea2	81.58101803	1.634444584	0.457148174	8.62E-05	0.000396841
Crb2	43.57258625	4.641396163	2.115706026	8.63E-05	0.000397224
Adam32	16.57665323	3.328520108	0.936649261	8.71E-05	0.000400687
Lrrc75b	146.8020911	4.44132203	2.022347134	8.76E-05	0.000402621
Col6a5	5.27860694	5.336946052	2.410794677	8.88E-05	0.00040764
Lpcat4	351.3912223	2.642126514	0.726976157	9.15E-05	0.000419703
Ttc22	103.1412851	4.454241054	2.043216439	9.35E-05	0.000427996
Cenpi	25.55071118	4.605654407	2.117653678	9.46E-05	0.000432873
Mis18bp1	53.623487	1.884096064	0.536832277	9.50E-05	0.000433724
Nbea	139.2233913	1.514836417	0.42309152	9.56E-05	0.000436315
Kif2c	46.20484629	2.194906891	0.633195654	9.62E-05	0.000438689

Spsb1	115.0903515	4.037087136	1.220679079	9.62E-05	0.000438689
Ttc16	26.38531768	2.291194574	0.667647435	9.71E-05	0.000442314
9330162G02Rik	47.41581936	1.549350474	0.441743903	9.73E-05	0.000443218
Ubtd2	107.105615	1.196949505	0.328895564	9.82E-05	0.000446953
Spa17	49.73769875	1.812983628	0.514927607	9.96E-05	0.000452692
1700016C15Rik	8.044629714	4.948298898	2.271653566	0.000100195	0.000455325
Ndc80	42.83197799	4.747139156	2.17572357	0.000100536	0.000456554
Cyp1b1	20.68097439	4.57684133	2.12264414	0.000100746	0.000457289
Gm17415	11.77397772	3.178763143	0.891040212	0.000101242	0.000459103
Plppr3	87.84527235	1.433314874	0.407133111	0.000101998	0.000462207
Lrrn4	7.565095521	4.54440529	1.441773115	0.000103822	0.000469915
Gm15895	30.23636599	4.739754909	2.172001399	0.000104349	0.000472081
Parvb	16.27627889	2.351840247	0.698821571	0.000104932	0.00047443
Fut10	98.96711906	1.30026216	0.363210066	0.000105074	0.000474913
Gm43305	2986.452655	2.847499225	0.903791098	0.000105829	0.000477986
Depdc1a	21.46708159	4.744392009	2.188530546	0.000105886	0.00047813
Per2	1000.550869	1.529209143	0.444074933	0.000107229	0.000483626
Gm44284	23.56829357	4.409002928	1.396226324	0.000107523	0.000484837
Gpm6b	96.80940341	4.373673639	2.028448829	0.000108789	0.000490317
Pdlim2	73.82258096	3.583402941	0.958636801	0.00011065	0.00049777
Gm36026	15.10109659	3.149752772	0.88829703	0.000111963	0.000502969
Gm50232	19.89243577	4.560505768	2.127111527	0.000115555	0.000517528
Gm38393	50.05274479	1.321587778	0.372370135	0.000115621	0.000517705
Gm45774	8.920323414	4.700211091	1.525704843	0.000115892	0.000518557
St6galnac2	262.5772668	4.418576657	2.047050308	0.000118995	0.000531323
Chtf18	70.2161767	1.951786679	0.574200332	0.000121612	0.000542504
Dio3os	128.3888857	1.794087228	0.520845071	0.000121909	0.000543701
Zscan30	39.37612708	2.228497499	0.638811649	0.0001224	0.000545386
Gm3788	51.63671794	4.38484346	2.053363012	0.000122997	0.000547665
Trmt13	125.3649653	1.028019608	0.290629983	0.000123061	0.000547822
Gm31814	27.73860891	1.581301433	0.46171801	0.000123852	0.000551214
Asf1b	32.78993591	2.150573822	0.638934885	0.000124803	0.000555191
Galnt12	115.1086543	4.299563011	2.015229114	0.000125597	0.000558334
Fam72a	25.79667033	1.859612221	0.551547261	0.000126569	0.000562264
Angptl8	1888.43215	2.566213761	0.813765313	0.000128146	0.000569009
Gm5148	80.88554426	1.58037271	0.45214093	0.000128294	0.000569366
Nkd2	5.213609525	4.93956301	2.312316746	0.000128316	0.000569366
Gm43127	35.73598559	2.728310684	0.789722083	0.000130001	0.000576045
Rem2	130.7020789	2.350168162	0.689238099	0.000130332	0.000577377
Trpc1	71.14294356	2.909527067	0.808461921	0.000131356	0.000581376
4732463B04Rik	26.17044548	1.622573644	0.476397612	0.000131648	0.000582266
Mark1	55.00052415	4.316740209	2.038171077	0.000132628	0.000586201
Gm37558	6.569173257	4.934093029	2.306341382	0.000132608	0.000586201

Mast1	97.858653	1.406320962	0.409173263	0.000138107	0.000607583
Npb	27.61570628	1.89955837	0.575384208	0.00013824	0.000607922
Wdr31	110.092143	2.322090029	0.66483858	0.000138454	0.000608583
Scn2a	11.20260729	4.181356306	1.334667366	0.000138512	0.000608697
Plcg2	222.8589234	3.495122816	0.949988045	0.000141091	0.000618754
Cdsn	164.7328046	4.257224853	2.004960323	0.00014149	0.000620366
Arhgef39	24.56724148	4.10671061	1.308602119	0.000141776	0.000621335
Gpr137b	91.55136536	3.517470816	0.962538692	0.000143876	0.000629533
6330403L08Rik	85.06653915	1.287983631	0.371464071	0.000144776	0.000633326
Garnl3	107.4779596	2.347902753	0.678044484	0.000145536	0.00063578
Ptpn14	1536.555308	2.196393577	0.685148024	0.000147431	0.000642449
Cdr2l	152.11532	3.449164652	0.938566671	0.000151945	0.000659723
Oas1g	50.4381167	2.2548192	0.66445428	0.00015267	0.000662423
Large2	252.7174892	1.331213503	0.384648762	0.000155474	0.000673675
Cenps	15.31394597	4.639400368	2.199704832	0.000155664	0.000674345
Spdl1	52.38987669	1.583359202	0.464457238	0.000158146	0.000683707
Steap1	60.49469526	2.358104293	0.689547222	0.000159736	0.00068965
Gm11944	34.30504677	1.964514097	0.578184362	0.000162742	0.000701996
Gm43808	17.80249199	1.891615271	0.565271052	0.000167708	0.000720986
Gdpd5	486.8092151	2.267943333	0.657527448	0.00016833	0.000723116
Snhg16	54.79123552	1.203635608	0.34466447	0.000168436	0.000723306
Prph	21.41619843	3.579775318	1.009880463	0.000169019	0.000725647
Gm38228	24.01816397	4.357292847	2.0930472	0.000169061	0.000725666
Aurkb	72.99132965	2.366192265	0.744734999	0.000170316	0.000730725
Thsd4	1093.608678	4.119139809	1.957947268	0.000172202	0.000737169
Nkain1	86.4395535	4.194625175	2.014476342	0.000172918	0.000739571
Kctd11	141.6557435	1.074227041	0.306066706	0.000177011	0.000755899
Ly6k	6.819531188	5.264081168	2.478612815	0.000177533	0.000757961
6530402F18Rik	150.7293879	3.785671412	1.199593014	0.000177574	0.000757966
Slc44a2	994.903984	1.490102595	0.433630126	0.00017829	0.000760855
Cacnb3	476.2427147	3.742243613	1.180777891	0.000178714	0.000762331
Plk4	99.29275816	1.125724562	0.322471137	0.000178965	0.000763058
Ptrh1	72.44651728	1.027286409	0.292088967	0.000179637	0.000765751
Pnldc1	15.22607229	1.980428525	0.613730608	0.0001826	0.000777862
Atad2	306.9481756	1.097049113	0.315885449	0.000182658	0.00077794
Dusp9	43.86067883	2.895207149	0.836294974	0.000184749	0.000786322
A4galt	97.65944013	4.225193843	2.030577564	0.000185407	0.00078877
Nr4a1	1954.582923	2.788735402	0.942083911	0.00018571	0.000789884
2510016D11Rik	37.99216227	1.349304227	0.394875491	0.00018803	0.000799045
Fhdc1	81.407836	3.807565433	1.217906186	0.000188674	0.000801602
Ydjc	39.60354317	1.886485074	0.570029133	0.000189047	0.000803012
Ptk7	626.2119198	3.733561695	1.1870352	0.000189419	0.000804338
P3h3	160.2122771	2.835271768	0.815784886	0.000192404	0.000815283

Atp8a1	174.8818103	1.0838655	0.312229356	0.000196009	0.000828913
Boc	14.34893349	4.043758977	1.335636512	0.000196722	0.000831744
Cebpd	140.1707693	1.021620137	0.293730865	0.000199032	0.000840544
Hand2	6.578559141	5.032955692	2.406970081	0.000199379	0.00084168
Gm29585	10.59441738	4.364099295	2.137643924	0.000208264	0.000876451
Chst8	81.45846187	4.292007321	2.093078685	0.000212488	0.000893484
Atp6v0e2	282.3084529	2.467473428	0.729077304	0.000219482	0.000920474
1700003F12Rik	21.4230467	3.040561062	0.922225508	0.000221144	0.000927037
Zfp677	79.17358079	1.509598263	0.450530773	0.000223076	0.000934727
Kif15	56.74713637	3.473541805	1.004609571	0.000224224	0.000938924
Muc13	6.186834385	4.51198505	2.219190748	0.00022468	0.000940627
Gm28172	20.36134357	1.861197377	0.57677776	0.000227106	0.000949748
Prr11	45.58237529	3.8174744	1.254586628	0.000229108	0.000957692
Nmb	19.11236823	3.439289839	0.989778937	0.000229851	0.000960179
Nr4a2	47.60476213	2.166437593	0.673827259	0.000235309	0.00098107
Gm20548	16.93634852	2.420750813	0.747290401	0.000238431	0.000992997
Rbms3	396.3315416	3.336771051	0.948807727	0.000239048	0.000995133
Ncaph	125.5472099	1.525362395	0.467473513	0.00023953	0.000996709
Gm48427	22.69402501	4.352576182	2.146063481	0.000243518	0.00101111
Parpbp	28.47248195	3.921106439	1.31709818	0.000243739	0.001011689
Gem	69.64046124	4.083679592	2.02029354	0.000244984	0.0010161
C230096K16Rik	29.55870902	1.949012737	0.62181121	0.00024768	0.001025951
Bicdl1	120.5742969	1.141034061	0.339660993	0.000248227	0.001027551
Trim6	32.17250932	3.059169581	0.949664181	0.000257373	0.001062888
Cdc7	29.52995471	4.308817769	2.136197697	0.000258296	0.00106647
Tnc	12.4546839	4.210448024	1.466721768	0.000258844	0.001068506
Ercc6l	41.86769784	1.946055722	0.599582996	0.000260341	0.001073991
Tcf21	5.002477697	4.773639129	2.355671774	0.000260754	0.00107523
Egr3	456.9616079	5.800917185	2.764170962	0.000266838	0.00109843
Gm45853	12.19484937	4.287710069	2.14278607	0.000267863	0.001102417
3010003L21Rik	29.82646201	2.430314948	0.762047795	0.000268298	0.001103968
Rmi2	11.1887712	4.396994049	2.195117233	0.000272109	0.001118451
Mthfd2	70.40004096	1.184509215	0.355695284	0.000275921	0.001132178
Gm11419	30.20609575	1.469205525	0.455974319	0.000277334	0.001137734
Gm44321	27.87095241	2.510529602	0.768348555	0.000280299	0.001149159
Pgm5	36.6665419	3.696045059	1.23416183	0.000286036	0.001170928
Nbl1	36.84218494	4.449931653	2.2138775	0.000289879	0.001184451
Ace	88.97615753	2.287687683	0.734055988	0.000293189	0.001197148
Mt2	13.09228646	3.20083719	1.075183315	0.000297118	0.001211643
Scel	34.66803291	4.018410485	2.028182436	0.000297627	0.001213204
1700007J10Rik	12.75055289	4.131833476	2.095759956	0.000303867	0.001237062
Nr2c2ap	24.6422567	1.796407508	0.574415994	0.000304025	0.001237256
Arhgef25	326.3183505	2.261424631	0.703408279	0.000304084	0.001237256

Upp1	82.66157653	1.149523673	0.346030464	0.00031146	0.001265292
B130055M24Rik	43.2211138	1.753339593	0.553970968	0.000312596	0.00126883
Siglecf	4.047078919	4.879502896	2.442061021	0.000313604	0.001272114
Neil3	28.39842296	4.779099327	2.373014265	0.000314961	0.001277079
B3glct	159.1523964	1.030114007	0.306091039	0.000316001	0.001281025
Rcan1	613.603878	1.209300018	0.370152873	0.000319722	0.001295289
Polq	44.58854552	3.720043565	1.26898555	0.000320888	0.001299192
Bard1	44.89485022	3.441873466	1.044041626	0.000322388	0.001304163
Abcb1b	71.35472353	1.415740237	0.443232108	0.000322534	0.001304478
Fam131a	54.12628782	3.631582854	1.225959338	0.000323209	0.001306932
Gm43197	66.11778939	3.605310785	1.214086188	0.000325958	0.001316661
Fst	416.2013913	1.073652265	0.323528356	0.000326523	0.00131839
Gm44164	19.09565313	3.377697696	1.013776513	0.000326937	0.001319781
Lif	633.8391944	1.679832	0.545848409	0.000328739	0.001326499
Mllt11	89.3895556	1.151215389	0.346930165	0.000331691	0.001337284
Gm42659	25.12638908	1.652249801	0.519459004	0.000331633	0.001337284
Dkk3	77.99882565	3.268664269	0.96466029	0.0003339	0.001345798
Cyp2j9	248.4499369	1.058869196	0.31896594	0.000336516	0.001355029
Troap	31.33847961	4.188332204	2.131107652	0.000340532	0.00136919
Gm15567	16.48719522	2.129968966	0.688402158	0.000341924	0.001373925
Chsy1	142.2913045	1.325597505	0.412665385	0.000343778	0.00137964
Jdp2	98.06765882	2.419536176	0.753329795	0.000349357	0.001399811
Itga8	15.07959948	3.338958269	1.118376631	0.000350295	0.001402568
Ncapd2	462.4268365	1.052547442	0.31848714	0.000350538	0.001403248
Ksr1	84.52119396	2.073329986	0.655307812	0.000353379	0.001412852
D130040H23Rik	62.39691195	3.237048187	0.954997999	0.00035369	0.001413804
Dnajb1	703.3704621	1.096400512	0.333845878	0.000356297	0.001423334
Aldh1a2	206.6968837	3.65604912	1.260043936	0.000357532	0.001427675
Lama2	14.06551884	3.712463857	1.202108199	0.000363631	0.00145082
Pole2	45.15455352	2.445888777	0.770663329	0.000374523	0.001490867
Iqank1	53.56789908	1.390456655	0.432397866	0.00038238	0.001519305
Mst1r	60.31536141	1.551694049	0.496795195	0.000382473	0.001519361
Mex3b	87.36450112	2.708010662	0.836830615	0.000382721	0.001520032
Sftpa1	26.40572689	1.521896962	0.489947769	0.000385301	0.001529645
G930009F23Rik	214.0800398	3.514190947	1.197949074	0.000387544	0.001537916
Kif23	174.949481	3.216002648	0.959742291	0.000387663	0.001538069
Gm12592	13.80618582	4.112772623	2.129258331	0.00038963	0.001545553
H1f10	92.64661293	2.471432532	0.787186966	0.000391891	0.001552599
Ar	47.5156975	1.459913014	0.469019244	0.000393407	0.001557639
Optc	3.987759323	4.347394202	2.254499592	0.000395473	0.001563883
Chek1	71.09400761	1.660357589	0.539235086	0.00039729	0.001569773
Ablim2	64.02134382	1.005469867	0.304024667	0.000397563	0.001570528
Carmil3	73.0246665	3.257980006	0.988408669	0.000400386	0.001581031

Scarf2	441.453877	3.396920372	1.06679351	0.000402558	0.001588625
Depdc1b	11.88848391	4.089841133	2.134316432	0.000413348	0.001628528
Phf21b	24.1227677	3.264435191	0.993623823	0.000416879	0.001641092
Cdca7	70.59290676	1.27767643	0.401706427	0.000421828	0.001658392
B3gnt2	75.77479875	1.009550206	0.307172344	0.00042238	0.001660024
Ankrd9	115.9023408	1.094610146	0.338022223	0.000423418	0.00166342
Bex1	110.4057192	3.859801255	2.007966645	0.000423665	0.001664051
Dbn1	703.0422279	3.449008605	1.185078544	0.000424392	0.001666567
Dubr	99.05120722	2.016518102	0.645114569	0.000427153	0.001676035
Sema6c	85.88822162	2.465511597	0.797026152	0.000430033	0.001686304
9530034E10Rik	9.284664292	2.390121399	0.818819893	0.000433379	0.001698728
Mmgt2	103.1822023	1.133796904	0.348850746	0.000434842	0.00170342
Gm17690	48.54150409	1.480674357	0.478903197	0.000435754	0.001706642
Hamp	4920.090558	1.291498587	0.409917364	0.000439904	0.00172009
Gm16617	16.29209584	3.921248534	2.061523407	0.000441913	0.001727595
Gm37856	8.856355769	4.188515577	2.194537395	0.000442395	0.001729126
Kirrel	803.298023	3.47667109	1.209375451	0.000443875	0.001734555
Sgo1	33.78505758	3.347255018	1.053034736	0.000451017	0.001761749
Ltbp1	91.28606414	3.513683026	1.230046897	0.000452972	0.001769025
Pcdh19	127.6479121	3.795037163	1.995461242	0.000456812	0.001783296
BB123696	4.212275409	4.607753136	2.408400891	0.00045706	0.0017839
Peg10	20.65387201	1.613964069	0.528885693	0.000458507	0.001789185
Ranbp17	84.76697498	1.931145519	0.626543155	0.000462608	0.001804088
C1qtnf12	64.81150577	1.715607183	0.56178119	0.000463183	0.001805716
D430020J02Rik	69.9641887	2.663264382	0.839235204	0.000464759	0.001811369
Auts2	442.3884988	3.724642788	1.946367405	0.000466038	0.001815985
Gm26586	14.39900978	2.833279619	0.921136734	0.000470829	0.001831371
Misp3	12.44467478	2.752171788	0.944456219	0.000476177	0.001850235
Zfp37	74.42362999	3.488630142	1.228405876	0.000480404	0.001864772
Artn	15.14717652	4.242503633	2.234407233	0.000482556	0.001872368
2900089D17Rik	27.76272719	1.98737669	0.674141967	0.000485619	0.001883111
Slc4a3	871.2598589	3.430091056	1.200364186	0.000486174	0.001884503
A630001O12Rik	7.320688426	3.704038181	1.341745848	0.000486131	0.001884503
Rasl10a	91.96391447	1.841673538	0.602756181	0.00049893	0.001929271
Hspa12a	46.63508459	1.628114205	0.531862259	0.000503582	0.001945691
Lhfp	342.1030529	2.416698601	0.880802991	0.00050415	0.001947126
Plekha4	81.79254494	1.272039609	0.402504711	0.000504157	0.001947126
Astn2	3.85465702	4.382164101	2.324150612	0.000504265	0.001947154
Chrdl2	38.3528237	3.466596898	1.226052403	0.000506379	0.001954356
Ankle1	32.154782	3.613874928	1.306800411	0.000506864	0.001955616
Tnfaip8	301.0220114	3.109903551	0.95122495	0.000511191	0.001970723
Tmem130	134.6827915	3.738716791	1.982608101	0.000511435	0.00197127
B430203G13Rik	8.558409291	4.045823071	2.155934415	0.00051821	0.001995377

1700058P15Rik	21.23741341	2.679941397	0.861338575	0.000521019	0.002005792
Knl1	59.67721292	4.108122756	1.589763836	0.000534328	0.002054144
Pde6c	11.66684245	3.008855076	1.031572644	0.000539256	0.002071865
Gucy1a1	10.65437665	3.50292482	1.176964352	0.000558834	0.002141061
Plscr4	45.94166742	2.686415134	0.877161124	0.000564846	0.002161938
Wnt5a	178.9172445	1.210647404	0.386608535	0.000568918	0.002176655
Gm45221	51.30545664	1.227740685	0.393199567	0.000573279	0.00219072
Tgfb3	391.4622532	3.626623046	1.945069553	0.000575856	0.002199769
Gdf10	13.69962413	2.744937691	0.996773593	0.000576393	0.00220043
Pwwp4c	12.896929	3.506202603	1.273099284	0.000580531	0.002215787
D730005E14Rik	39.29941939	3.85075685	2.074776825	0.000581981	0.002220436
Gm45244	6.694165504	2.877844892	0.976617744	0.000585267	0.0022312
Colca2	45.28046216	2.666956803	0.874418504	0.000587747	0.00224021
Zfp185	247.3816632	3.625537447	1.953010856	0.00058955	0.002246634
Lrrc75a	30.08233169	2.660414309	0.869872464	0.000590944	0.002251054
Rptoros	8.135022389	3.45880598	1.160261709	0.000596357	0.002270319
Cox7a1	30.53113275	1.441669585	0.478060755	0.000601899	0.002290509
Gm16184	56.58035589	2.287484768	0.74749046	0.000602983	0.002294179
Map1a	24.78475105	3.890268459	2.11139732	0.000609415	0.00231544
Socs3	416.64382	1.96173464	0.708915567	0.000613148	0.002328699
Zfp462	599.0929267	1.793595923	0.589455444	0.000615332	0.002336069
2310040G24Rik	80.17449762	1.009887865	0.318543078	0.000622617	0.002361859
Cldn34c1	41.91100176	1.75488273	0.581466347	0.000627606	0.002379844
Ubxn11	90.05028327	3.651332742	1.985447912	0.000629273	0.002385692
Fut1	14.15410487	3.573236467	1.329876423	0.00063157	0.002392039
Rin1	181.2400013	2.249603125	0.735986957	0.000633059	0.002396261
Rcn3	66.14532956	2.389016253	0.804202176	0.000634786	0.002401377
Scn7a	701.9877249	3.586881918	1.94559045	0.000643911	0.002434455
Smtnl2	266.6309909	1.191404791	0.380090473	0.000645986	0.002440857
Fanci	41.16299358	2.751759434	0.937223708	0.00065636	0.002477131
Gm44899	10.47805105	3.927268544	2.147069522	0.000657465	0.002480812
Plxdc2	199.3478982	3.996729844	2.169430284	0.000658056	0.002482065
Adm	12.13534652	2.750375068	0.934248518	0.000664367	0.002504392
Mfap4	4.013781849	4.302855089	2.344912698	0.000667929	0.00251535
Cenph	30.96775385	2.173733869	0.740486137	0.000673672	0.002534989
Mbd4	101.9419594	1.184206411	0.380691958	0.000676799	0.002546254
Snai3	5.707285013	4.591066168	2.485037807	0.000680573	0.002558951
Nrarp	30.05384366	3.753444103	2.061078192	0.000681645	0.002562478
Sarm1	77.55475636	1.720647581	0.593420207	0.000683375	0.002567471
Cmya5	7.184232572	3.888521548	2.148685967	0.000703403	0.002638588
Gm44949	24.90828829	1.991991512	0.70196196	0.000707877	0.002653775
Ccsap	22.63345117	2.212806074	0.762674306	0.000708005	0.002653775
Gm17359	32.00249849	1.885467504	0.656048129	0.000708172	0.002653882

Htra3	41.1353314	3.035577171	0.968061471	0.000715569	0.002678987
Gm7628	45.33476507	1.279408742	0.418554064	0.000717058	0.002684036
Rgs11	123.9733222	3.068821298	0.990800126	0.000725884	0.002714958
Rbm38	93.73252033	1.148969037	0.372920227	0.000729701	0.002726577
Dnah7b	249.2872674	3.272020142	1.197509464	0.000730056	0.002727369
Gm37818	10.70577781	3.517524119	1.33553252	0.000741078	0.002764781
Gm15911	7.192873984	3.985535775	2.210174212	0.000750851	0.00279806
Kif14	36.35849824	3.361800028	1.252236111	0.000751157	0.002798576
Prtg	145.4384458	3.521488407	1.949911529	0.000751612	0.002798641
Eme1	16.47134209	3.839561993	2.132899902	0.00075141	0.002798641
Spire1	47.906106	1.824673034	0.619366174	0.000764207	0.002838928
Sfxn3	763.4394252	2.499499007	0.83161615	0.00077763	0.002883767
Atp8b2	593.136272	1.28136368	0.421555574	0.000779666	0.002890759
Antxr1	37.66410249	1.940398789	0.663172029	0.00079631	0.002943939
Gm29361	23.06675745	1.390265069	0.464864885	0.000798722	0.002951719
Tekt2	12.19176898	3.950652773	2.206458154	0.000805758	0.002975429
Doc2g	42.89293358	1.087826841	0.352673309	0.0008077	0.002982028
Hyls1	52.89767621	1.011371975	0.326360108	0.000815255	0.003007316
Slc9a5	110.2848241	3.232383822	1.198778237	0.0008201	0.003023152
Tnxb	33.73380439	1.570910683	0.553024636	0.000822493	0.003031199
Lor	22.55616014	1.558363134	0.535324512	0.000831152	0.003056262
Chaf1a	127.8561661	1.090152746	0.358333794	0.000835002	0.003068658
Fbln1	14.84826511	2.435478475	0.897370998	0.000836131	0.003072219
Tox3	113.4933192	3.505747748	1.97308373	0.000851064	0.003119918
Kitl	1002.121375	1.623172765	0.556824509	0.000858666	0.003143582
Gm15347	27.08999816	3.554361676	2.010340627	0.000859382	0.003145568
Gm44202	9.146276607	1.89737111	0.682351853	0.000860312	0.003147207
Cdca2	48.40059752	2.39929546	0.912361745	0.00086521	0.003163314
4833438C02Rik	49.28965929	1.43245428	0.4937364	0.00087748	0.003203293
Sorbs2os	45.74983092	1.780675004	0.611156667	0.000880729	0.003212712
Gm18228	31.06247993	1.708564916	0.605744959	0.000882002	0.003216741
Srpx2	4.138825762	4.372707776	2.453295238	0.000885398	0.0032279
Gm5577	15.21096952	3.714293144	2.10681614	0.000890417	0.003244965
Orc1	17.1672895	3.644918036	2.074453072	0.000900186	0.003279321
Pmaip1	184.9748214	2.485884204	0.841084499	0.000901339	0.003282277
2610020C07Rik	24.42932108	1.864766313	0.677068864	0.000920472	0.003343703
Fos	8435.534606	1.229709011	0.420985756	0.00092466	0.003357645
Pkhd1l1	12.58094383	3.930543532	2.238599619	0.000925201	0.003358337
Zgrf1	75.24032242	1.006605419	0.327214264	0.000931883	0.003378759
5430405H02Rik	21.11507078	1.884269282	0.668107369	0.000948735	0.003431429
Ccdc116	11.65287636	3.745154944	2.145425389	0.000953083	0.003444559
Synm	50.19422357	1.373094735	0.4701663	0.000957617	0.003458062
Mxd3	33.93526848	2.097844141	0.740067387	0.000962731	0.00347289

Barx2	11.63264111	4.223760957	1.820495341	0.000964373	0.003478158
Gpr17	50.16404841	1.163872611	0.392283438	0.000965317	0.003480909
Il15	62.20420724	1.042596004	0.342841401	0.000982407	0.003537883
Mex3a	576.340449	2.884834276	0.946170168	0.000988272	0.003555413
Hdx	69.25697737	3.489501557	2.001147098	0.000992175	0.003567033
Gm17300	15.38577159	2.641759987	1.002956119	0.001009552	0.003624753
Nbdy	58.24988229	1.049426055	0.346608513	0.001012095	0.003632527
Flrt1	43.12652168	2.516354136	0.877283579	0.001015039	0.003642411
6230400D17Rik	37.48373846	1.291358675	0.443285417	0.001024481	0.003674234
Kif11	135.2306519	1.39910746	0.493088327	0.001047027	0.003744594
R74862	31.01268452	1.872080323	0.656947953	0.001052967	0.003761633
Atp1a2	5.776526169	3.806817459	2.209796603	0.001060922	0.00378723
Gm830	656.8243657	3.099701072	1.186349281	0.001069299	0.003811465
Sh3bgr	16.84616092	3.716590303	2.154657504	0.001072079	0.003819952
Noxa1	59.2643073	1.278163459	0.441250324	0.001080331	0.003845786
Ak7	13.38465702	1.977907005	0.738008858	0.001082961	0.003853718
Cd34	9.532041398	3.406300612	1.379486329	0.001084651	0.003859016
Gm12207	7.288773403	3.815767398	2.223290042	0.001089415	0.003872631
Ntng2	276.1655324	2.87912133	0.963187631	0.001099673	0.003905946
Myom1	58.95770212	1.68136818	0.594661309	0.001115339	0.003956463
Greb1l	110.5681478	1.293193722	0.453616453	0.00112077	0.003974261
Vcan	1058.375989	2.876690311	0.972756657	0.001130398	0.004004702
Fancd2	54.80600656	1.830425957	0.644078847	0.001133968	0.004016606
Gm43313	6.554451951	3.642804881	2.1451559	0.001162365	0.004105068
Fam83c	21.92192279	2.119311828	0.775140801	0.001171986	0.004136003
Ccdc146	27.77701718	1.700311016	0.615871444	0.001176643	0.004150147
Tead2	505.5702706	1.449913856	0.497608436	0.001188807	0.004188434
Shcbp1	42.82312221	2.0666411	0.754495944	0.001206595	0.004244092
Gm41442	13.48279305	2.700362582	1.016106174	0.001215537	0.004271631
Dok1	91.43570034	2.374446533	0.82442228	0.001217334	0.004277162
Tmem52	25.79017284	3.179255524	1.267175323	0.001222969	0.004294602
Mir7678	2.548734967	4.173578512	2.459992357	0.001228704	0.004313164
Cfap52	17.88567296	3.421088285	2.034532655	0.001249364	0.004376081
Pnpla3	16.08431259	1.894386803	0.726004531	0.001258799	0.004406714
Gm26873	48.12395484	1.10053698	0.37742222	0.001259235	0.004407437
Pygm	8.289811685	3.598207595	2.142495793	0.001267699	0.004435445
Vps37d	141.9612825	1.459341127	0.52056105	0.001304276	0.004546841
Dusp8	222.6902988	1.159139455	0.405409032	0.001304092	0.004546841
Kcnk4	3.900643381	4.598202043	2.713815748	0.001311128	0.004566579
Pclo	35.60066956	1.344550415	0.475699706	0.00131232	0.004569903
Gm31224	39.33679131	2.414107966	0.862987816	0.001319967	0.004591533
Sptb	177.5237466	1.051887163	0.357286474	0.001321759	0.004596932
Cdh3	298.4689924	4.204766578	2.522657955	0.001323989	0.004603022

Zfp61	69.43787509	1.799539129	0.644171799	0.001325756	0.00460833
Gm38077	40.23889417	1.023521113	0.347440142	0.001329592	0.004619153
Gm37893	51.22166638	1.018755206	0.34907233	0.001373102	0.004755675
Paqr8	84.49051886	3.266313615	1.962653092	0.00138026	0.004778742
2010016I18Rik	23.22370327	1.694827166	0.619882361	0.001382701	0.004786331
Slc22a17	272.2368661	2.403979319	0.870947085	0.00138824	0.004802906
2700099C18Rik	22.49674793	2.160055126	0.790422869	0.001403569	0.004848947
Ciita	27.92394648	1.453957789	0.521381094	0.00140641	0.004855264
Cep55	42.01876417	3.080370888	1.24256857	0.001418815	0.004891052
Mmp11	278.7296515	1.88089839	0.674586608	0.001424478	0.004908809
Zfp57	176.9971555	3.02590955	1.212563032	0.001424226	0.004908809
Fxyd3	331.782777	3.226110266	1.950147261	0.001429695	0.004925018
Tmem151a	80.14549747	1.918866638	0.695943153	0.001434899	0.004940285
Msn	1386.492042	1.441152523	0.537970197	0.001440552	0.004957079
S100b	78.17816672	3.254658818	1.96548699	0.00144113	0.004958181
Gm37309	31.13839739	1.440887916	0.530490418	0.001473503	0.005059582
Gm10130	51.61521536	1.174845095	0.413921903	0.001485346	0.005096597
Abcc8	6.264116068	3.923184828	2.383825028	0.001491868	0.005114405
Sult2b1	100.1641722	3.229511637	1.963560529	0.001503464	0.005147723
Agap2	179.0883124	1.06450003	0.37235435	0.001504861	0.005150666
Mdh1b	5.578590861	3.478317381	1.533965233	0.00150967	0.005166206
Galnt15	87.77506188	3.832053941	2.349281872	0.001513121	0.00517709
Smpd5	46.5935142	3.026846097	1.230216907	0.001556441	0.005312996
Tmem198	10.13745932	3.448706892	2.116164017	0.001562589	0.005332086
Gm12454	13.74532642	3.479093891	2.141282905	0.001570751	0.005358983
Gm37979	4.80373724	3.593070552	2.211749792	0.001573261	0.005366594
Gm42664	69.00238182	1.576141938	0.589093958	0.00159549	0.00543662
Cdr2	33.47884048	3.085773373	1.288307128	0.00160139	0.005451884
Adam12	17.45693238	3.36131193	2.072476489	0.00160498	0.005459749
AF357399	7.429024261	3.029065805	1.146360579	0.001606832	0.005464595
Gm43289	5.28696424	2.744662016	1.097263675	0.001615837	0.005491328
Gm39556	6.661985151	3.310080616	1.438220835	0.001625894	0.005522571
Clvs2	3.291105887	4.21658955	2.592659331	0.001635962	0.005553819
Mir3058	19.7724729	1.471316821	0.543565411	0.001671852	0.00566464
Gm44200	7.713401625	3.592631596	2.232284623	0.001685037	0.00570327
Gm17501	37.0080403	2.543293164	1.0986391	0.001690671	0.005720323
Zswim3	58.35830633	1.02385143	0.357461496	0.001691953	0.005722641
2810408A11Rik	79.58755612	2.963878335	1.216634903	0.001693014	0.005725221
Capn6	16.05002096	3.232806098	1.405201312	0.001699113	0.005744835
Plpp7	33.67716631	1.345972131	0.496017946	0.001704953	0.005762549
Niban3	36.70527066	1.909679787	0.735339717	0.001711487	0.005779542
Gm19265	13.38801451	3.022688447	1.164866098	0.001736893	0.005857694
Gm38253	37.44126495	1.393856399	0.513924282	0.001768229	0.005955438

Gm29438	20.22954277	2.390317773	0.90097401	0.001768913	0.005956696
Kcnab1	346.1624048	2.046237004	0.76156073	0.001771167	0.005961147
Traip	26.9616821	1.836059226	0.696361738	0.001772601	0.005963882
Gm17971	15.02317223	1.99865243	0.813620426	0.001793828	0.00603107
Tmem138	79.62277757	1.051055885	0.370797459	0.001801004	0.006053077
Mapk11	128.9754024	2.702015206	0.960619562	0.001813172	0.006085448
Gm44167	4.278832306	3.758563314	1.75994623	0.001814009	0.00608719
Art4	8.077303282	3.526919454	1.655448816	0.001847061	0.006185125
Mustn1	9.138583595	2.949721587	1.136912206	0.00185898	0.006221779
Gm42986	10.74769537	2.46163304	0.959512013	0.001865279	0.006239596
Gm27042	20.15360012	3.001325951	1.272528922	0.00186809	0.006246822
Ggn	26.65313382	3.020458853	1.286589715	0.001872096	0.006259127
Gm26797	28.2310719	2.308377051	0.862611522	0.001898728	0.00633823
Tyro3	92.90752832	1.712314661	0.639061575	0.001909265	0.006371188
Nfkbid	57.95808475	2.698345945	0.970688643	0.001921648	0.006409168
Gm19337	5.318327064	4.074453521	2.596631978	0.00193314	0.006443017
Exo1	47.0714357	2.846277495	1.076654806	0.001945632	0.006477902
Efna4	171.0829815	3.041243056	1.925855709	0.001969353	0.006550061
Dscc1	19.53961521	3.483885872	2.224177321	0.001982534	0.006590476
Gm45033	5.232767472	3.431093305	2.193604662	0.001992077	0.006617612
Gm10791	25.0397129	2.935864481	1.246334942	0.002002672	0.006648207
Gldn	8.604034614	2.014472934	0.843535288	0.002031157	0.00673704
Gm19409	6.266217993	3.365549204	1.575693416	0.002048818	0.006789651
Ceacam16	59.87706592	3.071692629	1.964065027	0.002058957	0.006819717
Gm38372	53.31086977	1.031462741	0.370037823	0.002062137	0.006827893
Gm26203	3.787269938	3.785150689	2.437698557	0.002067041	0.00684039
Anxa1	177.3922241	2.727367304	1.010620205	0.002068504	0.006843071
Gm33782	14.5099141	3.386729613	2.185765133	0.002080296	0.006877341
Gm43462	7.257643157	3.407481293	2.19684089	0.00208111	0.006878847
Ncapg2	193.4186314	1.020852071	0.367152365	0.002096433	0.006919757
Pmp22	32.38315648	2.275045954	0.860195536	0.002099734	0.006928473
Cyp2s1	166.3646658	3.063039752	1.964651349	0.002106628	0.006948829
Gm43328	24.87482657	1.266386797	0.472465641	0.002127833	0.007010341
Gm43857	19.55026254	1.681726929	0.657145645	0.00215015	0.007075993
Fam180a	4.565605533	4.133918483	2.695645263	0.002165054	0.007117085
Gm10605	4.343357819	3.151390829	1.43240056	0.002168374	0.007125559
Ephb3	96.56682762	3.077551609	1.986061672	0.00216968	0.007127409
P4htm	58.75262553	3.054567072	1.970908354	0.002173305	0.007138095
Cdc25b	96.33583443	1.324273464	0.493690578	0.002178933	0.007155358
Gm6483	31.26319663	1.285087905	0.478438734	0.002190417	0.007186919
Tnfrsf9	23.39224724	2.7728806	1.063979257	0.002202908	0.007224199
Casc1	34.67512958	3.076537876	1.992604087	0.002204605	0.00722606
Gm31036	42.07344191	1.457278766	0.554606135	0.002234895	0.007316594

Hmgn3	62.0266755	1.661319322	0.627183713	0.002249537	0.007359505
Pwwp3b	134.36951	2.627730786	0.962284985	0.002254171	0.007372152
Tbx20	7.83135407	2.880557821	1.146481898	0.002274329	0.007433011
2610306O10Rik	12.92297495	2.741592506	1.04492218	0.002277474	0.007440758
Gm22009	18.46412462	2.43525994	1.154484579	0.002287813	0.007469449
A730063M14Rik	28.3071877	1.165413025	0.43416675	0.002299645	0.00750425
Gm17146	4.039816762	3.744415625	2.458909262	0.002302623	0.007512688
Dync2li1	358.8915966	1.146291775	0.415286744	0.002316379	0.007552435
Pde3a	4.467158648	3.243792478	1.53700232	0.002317516	0.007554859
Gm31718	26.08707724	1.426305797	0.561056651	0.002323718	0.007571218
4930517G19Rik	5.365800659	3.520268411	2.322168949	0.002339467	0.007618652
Gm39041	12.58055945	3.220993763	1.542564152	0.002380311	0.00773853
Bnip5	37.92618617	1.594044828	0.652256343	0.002389368	0.007765345
1700126G02Rik	10.37224857	2.701851323	1.02889172	0.002400023	0.007795362
Epha3	3.546563899	3.814521749	2.533159657	0.002400229	0.007795362
Fam171a2	180.1094731	1.913440556	0.7352084	0.002401096	0.007796859
Gm43080	29.19929072	1.179957313	0.440549255	0.002421698	0.007857111
Fstl3	140.4054707	2.185397937	0.833796518	0.002451358	0.007939916
Tmem252	74.90748319	3.031258127	2.005645788	0.002461541	0.007970209
Rab7b	22.00571704	3.063181064	2.028432504	0.002479598	0.00802191
Fam81b	3.600792726	3.329050835	1.608906881	0.002479332	0.00802191
Rftn1	4.25952445	3.499793674	2.333758373	0.002489159	0.008051482
Gm47258	8.562933854	3.161945135	2.099349816	0.002498732	0.008079115
H2-DMa	18.03442717	1.770552428	0.718188869	0.002501422	0.008082975
Megf6	426.8945823	1.830283483	0.718515935	0.002503749	0.008088396
AC132288.1	21.70584802	3.038385467	2.013531127	0.002516638	0.008122569
Gm26542	6.722667478	3.333280365	2.226883453	0.002531424	0.008166173
1810062O18Rik	11.53635832	2.281287651	0.901019018	0.002536384	0.008176674
4933440N22Rik	20.83218877	1.560920407	0.633553222	0.002541553	0.008191963
5330438I03Rik	3.174904464	3.476048799	2.328471436	0.002554844	0.008230654
Cdh11	3.77104791	3.665208747	2.468108384	0.002564902	0.008258899
Rasd2	15.43831834	3.265452119	2.187743598	0.002586013	0.0083185
Lgals2	27.41397135	2.847255241	1.270368879	0.002592716	0.008338665
Sgo2a	26.88238683	2.7456485	1.09212409	0.002602359	0.008366975
Pcdh12	16.16690976	1.345238117	0.528193335	0.00262546	0.008436905
Fxyd7	11.92970501	1.907914951	0.771069134	0.002628014	0.008443699
A430019L02Rik	7.166400337	3.512674033	2.384580763	0.002629815	0.008446658
Snord3a	2.611901433	3.958123579	2.673950305	0.00264191	0.008484084
Gm42798	9.679139786	2.746470127	1.099884763	0.002649682	0.008506196
Gm38155	32.54138812	1.005179247	0.36895357	0.002655366	0.008523018
Rpl24	12.63447043	1.729028686	0.702024618	0.002665738	0.008554879
Ccdc33	8.690959668	3.430568501	2.320877815	0.002671608	0.00857085
Cercam	130.8035382	2.874501841	1.919895351	0.002685179	0.008612949

Ggt7	100.5594811	2.881830836	1.927403607	0.002699266	0.00865524
Wnt7a	247.4432558	2.850182858	1.908249804	0.002719595	0.008713147
Gm43654	12.26695543	2.122730439	0.884672396	0.002724694	0.008726569
Zfp566	30.87606071	1.604670484	0.635095055	0.00277442	0.008865117
Gm38248	27.16281525	1.58917582	0.642177444	0.002776919	0.008871626
Cenpw	33.22837866	1.21147708	0.467966717	0.002783115	0.008885504
Sema3c	808.0787374	2.835758222	1.908535034	0.002784177	0.008887415
Cyp3a57	4.307190447	3.58317541	1.849638934	0.002798352	0.008923759
Cacna1c	17.17773824	2.733680886	1.109588144	0.002804852	0.008942998
Colec11	18.86180813	1.660775349	0.704235996	0.002840675	0.009051204
Gm16144	7.251655938	3.229751392	2.216061732	0.002866493	0.009127404
Gm44669	12.14047858	1.739032575	0.734712435	0.002890149	0.009193679
Gm19510	4.773269998	3.063555227	1.519279265	0.003021495	0.009562256
Gm47694	17.57766441	1.759700678	0.74124864	0.003042553	0.009624136
Gm13073	5.237082	3.254589116	2.260063296	0.003075009	0.009713992
Fermt1	704.7645499	1.36107847	0.526995722	0.003103493	0.009800746
1700112D23Rik	11.43709138	1.611565431	0.675853719	0.003106187	0.009807641
Ttyh1	34.00293444	1.409940229	0.559496168	0.003181134	0.01001298
Zfp382	23.0605672	2.717515691	1.243833885	0.003195673	0.010052148
Ncs1	179.9732706	2.810471968	1.947270909	0.00320911	0.010092761
Gm49204	33.81194715	1.312350587	0.519991953	0.003224676	0.010136744
Gm42940	7.250312264	2.856314396	1.3708083	0.00325148	0.010209286
Fgf9	12.49361046	3.069066423	2.158654432	0.003276922	0.01027572
Gm21451	11.04033359	3.010061715	2.11096758	0.00328661	0.010299367
Gm15344	52.92045438	1.487687794	0.632264836	0.003311255	0.010363061
6820402A03Rik	16.0052439	3.024251691	2.125492035	0.003314246	0.010370731
Ccdc180	7.860542593	3.06675288	2.152471726	0.003358538	0.010502477
Septin4	69.74843512	1.439045765	0.593115717	0.003371301	0.010537238
Cd28	4.103774605	3.178001146	2.253316598	0.003383818	0.010567754
Gm45223	24.7620963	1.725022982	0.770315064	0.003389765	0.010582882
Tnfrsf18	38.84963068	1.64481998	0.696554402	0.003395879	0.010600248
Nav1	156.9112604	1.031081432	0.394278009	0.003421309	0.010669215
Gm48146	5.572446707	3.09727543	2.207316355	0.003436556	0.0107098
Xrra1	12.72276311	2.641362577	1.103287541	0.003450689	0.010748607
Gm28988	5.973808944	3.316650265	2.398826421	0.003475815	0.010821603
1700109K24Rik	33.96624116	1.031115226	0.393459016	0.003524688	0.010954217
Gm25788	3.102578659	3.177490256	2.275659154	0.003533134	0.010975134
Gm47690	14.34442471	1.971593798	0.831745111	0.003546626	0.011013479
Gm26724	31.13228853	1.273967891	0.503144992	0.003548982	0.011019013
Zfp69	27.76231811	1.271657905	0.512972293	0.003561328	0.011053767
Dmtn	47.84883153	2.63761492	1.226529684	0.003565492	0.011064903
Htra1	11.64470605	2.252489971	0.968792359	0.003578814	0.011102654
E330037G11Rik	8.224862645	2.204570773	0.931256293	0.003603179	0.01117463

Gm48137	8.980195408	2.6160181	1.098642856	0.003608301	0.011188707
Gm49085	3.623409313	3.083536556	2.217524757	0.00362961	0.011249328
9430062P05Rik	30.52276144	1.572347779	0.647884944	0.003630554	0.011250438
Cux2	42.65236181	3.286771244	1.863066964	0.003646635	0.011292976
Gprc5a	291.4088848	2.736533453	1.954442245	0.003682161	0.011388903
Cdhr1	8.286868632	3.246483943	2.380396708	0.003734361	0.011529298
Fhl2	28.85805277	2.822362178	2.023802947	0.00374007	0.011543734
Gm15512	3.016083546	3.395223527	2.492421995	0.003752594	0.011574417
Dsel	88.43937983	2.621019961	1.234371869	0.003762242	0.011600447
Zfp52	62.16139758	1.232937409	0.486546997	0.003787846	0.011668146
Mtss2	276.92488	1.241596668	0.492961937	0.003788675	0.011668828
Pla2g3	6.076133202	3.104556903	2.262322378	0.003790097	0.011671333
Pla2r1	111.4959879	2.074616269	0.849340846	0.003803954	0.011704614
Gm19144	15.3883404	1.890735879	0.830826967	0.003818964	0.011741388
4930533B01Rik	3.330729411	3.373406563	2.496428129	0.003822395	0.011748172
Mfap2	4.987026876	3.056765338	2.232117494	0.003838962	0.011789649
Snhg4	35.85122458	1.067283398	0.414100811	0.003852299	0.011825984
Lamc3	489.9696401	2.797666517	2.066425126	0.003880484	0.011901929
Actc1	7.191257163	2.886459638	1.368746974	0.003901418	0.0119604
Gm49961	3.999913995	2.824569618	1.432789565	0.003927045	0.012033197
Gm42726	3.677621472	3.481736121	2.629018995	0.003939659	0.012066071
Gm49322	11.8910708	2.188998509	0.951879273	0.003977795	0.012167337
A430057M04Rik	13.99972847	2.629281442	1.255139004	0.003981517	0.012176779
Prkg2	7.563089527	2.708642147	1.331588275	0.003985288	0.01218637
Hand2os1	6.42301618	3.038691118	1.675054816	0.003988325	0.012193714
Col4a6	492.2254006	2.618116473	1.893442568	0.003997448	0.012215768
Arl4c	1434.469653	2.187303809	0.981505262	0.004020408	0.012280064
Nnat	39.03537696	1.075823186	0.423330752	0.004043248	0.012343052
Upk1a	4.949891591	3.079021257	2.283694414	0.004056591	0.012374826
Gm16153	40.57858706	1.064458139	0.4204974	0.004092552	0.012468495
Gabra3	3.306427611	3.454481298	2.630394893	0.004093144	0.012468495
Folh1	3.747788327	3.530304493	2.656674306	0.00415382	0.012635276
Igsf23	26.5883022	1.802004265	0.809128223	0.004176652	0.012696679
Smim38	10.62981048	2.873379201	2.134049339	0.004242142	0.012877404
Lekr1	32.10689104	2.608384597	1.280316239	0.004244635	0.012882933
Acta2	4.015993473	3.448144853	2.663619524	0.004247983	0.012891056
Rnf122	127.1009787	1.808047934	0.755543828	0.00429743	0.013020521
Olfml2b	95.59418086	2.633212271	1.93735774	0.004310724	0.013053934
Stc2	10.35118102	2.895930568	2.168484599	0.004322937	0.013079218
Gm45342	5.795273633	2.884950369	2.155683495	0.004331776	0.013103895
Gabrb3	8.176214594	1.987386892	0.960223523	0.004377011	0.013228223
Cfap157	30.2241518	1.316034959	0.544738694	0.004398336	0.013286396
Gnai1	39.75036631	2.688961537	1.387881217	0.004421125	0.013348932

Zmynd12	12.25651761	1.84480821	0.82832002	0.004424062	0.013355699
Gm36423	4.42713105	3.161443345	2.439886421	0.004426428	0.013360738
Adgrd1	3.607110523	3.119977002	2.377212744	0.004433759	0.01337866
Gpm6a	168.999635	2.634150149	1.951809639	0.00445365	0.013434455
Gm43379	24.30277837	1.393460374	0.594247209	0.004494402	0.013548865
Sntg1	3.33253655	3.084588942	2.362940556	0.004500503	0.013562994
Gm44280	6.745677236	2.661972164	1.367329309	0.004604797	0.01385337
Plvap	38.10282421	1.103659759	0.449666818	0.004612879	0.013873338
Gm15774	7.440708877	2.629181077	1.33740674	0.004652169	0.013971797
Gm14239	4.197925734	3.138199253	2.461073694	0.004661529	0.013993336
Spint1	1028.786132	2.547793559	1.925425725	0.004726944	0.014167543
Scn5a	37.09287596	2.974949343	2.347710598	0.004759653	0.014247776
Gm22748	13.74215579	2.806539143	1.568535948	0.004787392	0.014317412
Gm48342	15.08042945	2.202001582	1.22523806	0.004797756	0.014343938
Gm43256	5.569577246	2.655679761	1.378620951	0.004817635	0.014398883
Gprin2	52.00996557	2.599959517	1.968528159	0.004819061	0.014400904
Zfp827	327.1924531	2.451617929	1.199818243	0.004887459	0.014578059
Rad51ap1	31.72731454	1.990728704	0.926531603	0.004901701	0.014613725
Rasl12	24.8138623	2.716544429	2.095435419	0.004904668	0.0146203
Islr	7.516523445	2.299326606	1.114778762	0.00490771	0.014624823
Gm37233	13.89206539	2.636918923	2.012514644	0.004931917	0.014690116
Gm38319	31.95420162	1.042144434	0.42330653	0.005010205	0.014897869
Nyap1	27.84093236	1.523497191	0.660706773	0.005024483	0.01493801
Gm7525	3.792390722	2.910360231	2.271440279	0.005025701	0.014939316
Ypel1	115.1976185	1.084340449	0.43409775	0.005067416	0.015047002
Lca5l	25.93240982	2.600374678	1.990459146	0.005069001	0.015048778
Slco3a1	2517.869175	1.458400718	0.624747717	0.005077111	0.015064136
Mmp23	96.6510357	2.015129234	0.886414391	0.005098857	0.015120767
Gm42658	16.93035635	1.934524958	0.898121061	0.005128711	0.015191403
Cd248	3.136082681	3.220975011	2.629525665	0.005155875	0.015260176
Adamts12	8.718033689	2.621588805	1.287852497	0.005252303	0.015509609
2010310C07Rik	3.732800427	2.848629756	1.621579378	0.005277725	0.015570302
Gm44171	4.056537807	3.055206571	2.48533655	0.005321693	0.015680731
Gm48062	26.56335601	1.473564479	0.636459402	0.005321504	0.015680731
Fxyd6	66.25037056	2.446893993	1.225852364	0.005325739	0.015690245
Rps10	38.58873571	1.043458697	0.427191558	0.005348786	0.015743644
Wdr66	7.95880314	2.729303842	2.16185516	0.005411177	0.015912643
Ppp1r42	10.94565539	1.652101548	0.77422424	0.005413961	0.01591595
Gm50462	19.34308168	1.891630992	0.875252141	0.005426124	0.015946822
Akr1b8	262.9965729	1.665367357	0.739315386	0.005440962	0.015985534
Gm49838	4.429623233	2.929220985	1.860436379	0.005450176	0.016010153
Gm15718	8.324807322	1.953166598	0.919051553	0.005464639	0.016047726
Gm31763	12.17222518	1.609282285	0.731960955	0.005472097	0.016067168

Itgbl1	2.580473122	3.19025557	2.66350919	0.005499009	0.016141248
Stx11	183.0128873	1.478569083	0.631193858	0.005529866	0.016221898
Palb2	55.5130709	1.041723795	0.427677486	0.005588234	0.016378099
Ccdc148	135.7895271	1.0599838	0.43031839	0.00563335	0.016497728
Fbxl12os	21.97974478	1.657570088	0.735594123	0.005655797	0.016553362
Gm42548	4.894239027	2.799843723	2.275328234	0.005660854	0.016565636
Gm25835	16.48387159	1.567672777	0.736699417	0.005672267	0.016591447
Gm11839	2.912797392	2.883923139	2.357902134	0.005744129	0.016781182
Mgst2	29.24544916	2.603738224	2.095332094	0.005801892	0.016937041
Lrrc27	29.65200797	2.310607967	1.009140099	0.005841643	0.017032919
Egr2	373.9044641	2.401209719	1.975687392	0.005862482	0.017077549
Gm17092	24.59156564	1.445104298	0.640308611	0.005870278	0.017095069
Sfmbt2	22.23386178	2.494355794	1.337620314	0.005876779	0.017111405
4930533K18Rik	14.03039637	2.564267852	2.060585489	0.005895106	0.017154356
Rap1gap2	25.59721036	1.956813256	0.870320017	0.005919692	0.017215459
Srf	830.0578623	1.085961661	0.460794002	0.0059299	0.01723992
Lrrc9	210.0672633	2.397521182	1.898791358	0.005940216	0.017267297
Fosl1	7.67853594	2.721632913	2.237543834	0.005965978	0.017336929
Trdc	3.659248734	2.981106764	2.539360409	0.005972397	0.01734714
Zfp850	47.88739691	1.142609023	0.478642141	0.005984367	0.017377211
Nat14	63.57177795	2.297690876	1.017461115	0.006017686	0.017460751
9930120I10Rik	10.12155039	1.733788004	0.812076696	0.006101007	0.017681126
Ovgp1	15.60259825	1.737183752	0.81876182	0.00616195	0.017833506
Slc27a3	190.5969161	1.084557855	0.45640247	0.00620041	0.017936701
Gm23608	2.75310151	2.772213296	2.304540018	0.006203956	0.017944252
Tlr1	8.113039658	2.629920857	2.170060967	0.006227277	0.018006278
Gm7665	10.29634377	2.618711045	2.159220017	0.006230303	0.018012315
Heph	61.35622795	2.418252688	1.943776989	0.006255569	0.018079914
Gm22107	10.72992931	2.135768206	1.064897359	0.006323447	0.018240385
Gm47430	12.59300245	2.340997916	1.0875417	0.006472466	0.018639421
Ska1	10.33649996	2.611716315	2.17487603	0.006486149	0.018673221
Slco5a1	52.26001801	2.390829447	1.94266983	0.00649724	0.018693933
Gm43389	4.852571574	2.691338338	2.28532815	0.006503489	0.018706302
Gm37860	6.381536231	2.418845334	1.179210557	0.00655457	0.018841933
Aoc1	43.49828331	2.35505531	1.236694708	0.006571904	0.018877626
4930483K19Rik	10.08886839	1.650358484	0.825772976	0.006571265	0.018877626
Gm30881	8.991270544	2.44220307	1.338327723	0.006587063	0.018915507
Txndc2	3.02333417	2.913958467	2.553038695	0.00662229	0.018999837
Fgl2	11.74524803	1.655937402	0.806120286	0.006629441	0.019017281
Abhd16b	9.931723088	1.994841975	1.019470745	0.006642347	0.019040927
Orai2	152.4521594	2.202317137	0.956597691	0.00665473	0.019067025
Pcdhga12	22.9081637	1.910043943	0.863881089	0.006662969	0.01908778
4933427E11Rik	3.488329757	2.845233903	2.511771317	0.006669145	0.019096921

Gm19345	24.77349442	2.39340649	1.96210649	0.006709005	0.019199598
Gm49396	30.74948687	1.094109005	0.465357865	0.006729287	0.01924903
Gm26628	18.88031369	1.271443937	0.561594515	0.006748681	0.019290131
Gm5526	17.67386057	1.233524383	0.552803686	0.006757732	0.019313126
Orm3	60.33650209	1.054602592	0.451029889	0.006766309	0.019334758
Asb5	7.405377834	2.019686928	0.974012105	0.006769325	0.019340496
Inhba	1380.10769	1.094137799	0.476942326	0.006789403	0.019394974
Slc10a6	3.110259815	3.616464827	3.553227715	0.006801996	0.019425165
Gm42672	8.582683681	2.037486682	0.995531965	0.006821262	0.019474388
Gm6583	3.719172248	2.701257177	2.350976889	0.00687218	0.019605173
Gm12870	3.158036353	2.745192783	2.388648877	0.006883471	0.019631548
Gm37660	4.679421993	2.801156074	2.529172286	0.006926283	0.019730191
Rasip1	24.05769303	1.954618674	0.927744517	0.006970881	0.019842505
Mettl24	8.23009628	2.660194558	2.349966856	0.006979945	0.019862415
Krt7	1733.540362	2.344290749	2.006134001	0.006984693	0.019872531
Gm20492	8.699766359	1.895677357	0.952669163	0.007040735	0.020011663
4933401D09Rik	6.418892664	2.636469436	2.308384129	0.007079959	0.020105281
Gm18935	7.325786067	2.55768158	2.204018482	0.007082534	0.020109618
Gm49783	16.4405106	1.400383428	0.647145378	0.007172856	0.020335984
Gm45718	22.41987685	1.193770011	0.529642396	0.007199752	0.020406209
Haspin	19.60890872	1.769716476	0.892196337	0.007229254	0.020483775
Mypopos	23.12913482	1.231704555	0.539779568	0.007231362	0.020486725
Gm17816	6.664100445	2.555959193	2.267177844	0.007387291	0.020882237
4933413C19Rik	11.13654784	2.439297326	2.101343119	0.007454637	0.0210571
Mxra8	46.19668268	1.251718631	0.578817071	0.007514746	0.021208159
Pcdhgb8	6.684915383	2.537687211	2.259302739	0.00757213	0.021351271
9130230N09Rik	13.99822747	1.228871397	0.556302966	0.007575289	0.02135704
Tmem17	29.41035105	1.150749253	0.509629801	0.00758146	0.021371297
Gins3	39.21095441	1.031971412	0.44114145	0.007600745	0.02141308
Kbtbd11	11.75345388	2.348532957	1.332376272	0.007617528	0.021454062
Gm4202	16.47243123	1.534973003	0.730151546	0.007699006	0.021664461
Sema3d	2.952288737	2.713813223	2.512232249	0.007701372	0.021667942
Armcx4	66.7184097	1.53870181	0.708987147	0.007742129	0.021763467
Gm30363	2.261207557	2.765145616	2.601943496	0.00778498	0.021874313
Slc2a4	11.57222567	1.55242221	0.759538942	0.007817525	0.021946478
Arhgef4	15.84248574	2.344889319	2.044812126	0.007969155	0.022336215
Dio3	27.50947446	1.898143196	0.920633016	0.008010034	0.022437681
Gm10676	7.515737432	2.332984899	1.352894387	0.008022166	0.022465108
Gm38380	3.289518435	3.015063812	3.566171613	0.008031478	0.022484572
Osbpl5	222.0661148	1.634217576	0.763181619	0.008188461	0.022854094
Arhgap27os1	5.576378585	2.665043581	2.636842721	0.008242834	0.02299157
Meg3	151.6559593	1.532446103	0.812356011	0.008252733	0.023010053
Ecscr	215.56994	1.410977494	0.646512332	0.008251881	0.023010053

Csrnp1	687.2792817	1.123102948	0.513299124	0.008339439	0.023211338
Cenpp	12.92350135	1.408777656	0.659119291	0.008350273	0.023228017
Itgb8	868.3872494	1.583192024	0.738133514	0.008365012	0.023258901
Uckl1os	20.15096387	1.317176962	0.612858304	0.008453001	0.023476344
Foxf1	6.762725972	2.50228593	2.357941871	0.008458476	0.02348336
5830432E09Rik	5.129791833	2.644624532	2.652357418	0.008462122	0.023488079
Ggta1	148.3715572	1.625808453	0.76896027	0.008478152	0.023522366
Fxyd5	70.55527254	1.129577018	0.505000093	0.008480765	0.023526216
Zfp991	28.85866783	1.74572593	0.876381358	0.008537634	0.023650478
Ms4a4d	113.1788769	1.636913463	0.775298815	0.008591323	0.023771061
Gm44008	5.844467025	2.375192367	2.161626405	0.008602801	0.023789099
Dnajc27	62.92121303	1.057391117	0.463803032	0.008611981	0.023811055
Gm28809	7.204385908	2.494590294	2.432251987	0.008697328	0.024026491
Msantd1	15.23182293	1.526337869	0.722443319	0.008709981	0.024049179
Gm4131	6.573778702	1.790794012	0.920687063	0.008735155	0.024109934
4933413J09Rik	6.189738237	2.368581688	2.170539486	0.008746866	0.024138785
Dusp18	167.9395725	2.131183483	1.867012224	0.008767316	0.024191743
Gm49971	8.931135157	2.18385392	1.064811338	0.008793584	0.024250275
Cspg4	179.356123	2.128860462	1.869385878	0.008836678	0.024344631
Lhx2	2.875511283	2.683284681	2.82480236	0.008853722	0.024384583
Gm7575	14.76773412	2.269899315	2.075793465	0.008943583	0.024601426
1520401A03Rik	3.16923942	2.578396734	2.605270372	0.008956095	0.024627656
Gm27241	3.773819224	2.542600436	2.535917289	0.008988509	0.024706171
Pif1	16.98965703	2.238154993	1.306593602	0.009026495	0.024791617
Tcp10b	5.197854709	2.320958841	1.260022878	0.009093659	0.024966584
Rnasel	44.06982505	1.094887186	0.489404381	0.009098386	0.024973951
Cldn6	552.9833547	2.054571696	0.945064362	0.009098946	0.024973951
Gpr55	7.246611866	1.937668593	1.009396673	0.009120876	0.02501983
Hrob	14.98934867	2.229211693	1.159086641	0.009136924	0.02505459
Gm10399	9.633103791	2.253823003	2.052672021	0.009179084	0.025154333
Gm8234	8.025496987	2.268793323	2.095604848	0.009230018	0.025272261
Nes	206.388938	2.087777944	0.997467669	0.009303488	0.025433511
Zfr2	73.37308608	1.037391682	0.455891289	0.009338922	0.025523105
Gm45104	17.19345097	2.275904911	1.251440107	0.009396868	0.025666852
Adcy3	107.2905637	1.020327641	0.452834504	0.009414109	0.025706625
Gm15247	6.029789644	2.378618098	2.323432579	0.009432191	0.025752337
Gpc6	20.7949628	1.844490102	0.929454601	0.00946665	0.025835393
Cpz	2.85900685	2.724859599	4.285009086	0.009485455	0.025879353
Osbpl6	41.5480278	1.592056977	0.758962373	0.009531911	0.025987628
Gm43375	7.996667041	2.29244476	1.464047105	0.009531807	0.025987628
Gm50383	7.469388308	1.871113435	1.056522288	0.0095721	0.026082374
Tssk4	7.553674766	2.354566644	2.347163209	0.009672721	0.026311721
Gm15903	5.14904422	2.257507131	1.414732689	0.009684439	0.026336131

Cfap100	11.67333179	1.489373224	0.778236858	0.00971882	0.026418396
Gm48624	9.394557583	2.231810854	1.385652335	0.009802375	0.026615362
Slc16a4	27.37380509	1.110580776	0.510447035	0.009866884	0.026752667
BC034090	243.2802067	2.105413367	1.212024642	0.009873617	0.02676714
Spock2	7.957970366	2.21903341	2.091828962	0.00994812	0.02695008
Aunip	8.520587304	2.235464184	1.40300729	0.00995689	0.026970032
Csdc2	6.220364987	2.21591915	1.192893601	0.009959983	0.026970796
Lrat	16.74285981	1.400501385	0.724666328	0.009998815	0.027064493
Plekhh2	204.7882611	2.059525951	1.906849832	0.010007377	0.02708385
Xylt1	67.43737179	2.081477078	1.912077007	0.010026916	0.027129078
Plac9a	13.8860135	1.826651255	0.930297143	0.010130876	0.027387187
AC109619.1	5.658209907	2.326676001	2.368793071	0.010153135	0.027435766
Lime1	4.516010185	2.288491866	1.510223461	0.010167098	0.02746963
Gm15411	16.52105336	1.342250895	0.664765745	0.0101986	0.02754311
Gfpt2	35.4402724	2.088149548	1.932906996	0.010214724	0.027578893
Gm49733	2.33498306	2.594734889	3.174375739	0.010224342	0.027597094
Gm4430	24.68542368	1.202633329	0.584512389	0.010283974	0.027730749
Emp3	21.37145743	1.475090134	0.716974566	0.010294039	0.027753989
Gm16892	8.836143211	2.100794375	1.067997716	0.01047482	0.028196791
Gm48027	9.020376118	2.133679736	1.118069345	0.010528451	0.028297015
Slc25a43	14.03718716	2.09088823	1.947124927	0.010529362	0.028297015
Gm16061	5.770378399	1.767852494	1.021260508	0.010549049	0.028338018
Nfe2l3	152.4786213	1.902675551	1.080485881	0.010599034	0.0284484
Gm50012	12.87070889	1.56770967	0.80886659	0.010668432	0.028622659
B3galt2	4.549389957	2.260578626	1.575174988	0.01067596	0.028638851
Prr15	34.80369642	2.087732572	1.081370717	0.01070163	0.028695681
Fbln2	14.82273622	2.123318687	1.115083877	0.010749075	0.028814847
Tesmin	17.81329935	1.090335005	0.512112541	0.010753149	0.028821744
Gm37314	7.667613238	2.145793457	1.315750516	0.010785119	0.028903395
Frem1	3.40353421	2.40428144	2.988718006	0.010918055	0.029206634
Dclk2	34.67781075	2.052957209	1.94630782	0.010921928	0.029208851
Amd-ps3	5.786207934	2.155309668	2.125575278	0.011015597	0.029426549
Gm50322	5.209072474	2.213254902	2.257939478	0.011060782	0.029530812
Gm37606	4.604368729	2.19776313	1.489402673	0.011231988	0.02995457
B3gat2	25.39900087	1.01214861	0.463312911	0.011266118	0.03003307
Gm28085	11.23016953	2.07004027	1.082866468	0.011292927	0.030091997
Angpt1	193.3652626	1.528709211	0.753441171	0.011321998	0.030156899
Ikzf2	111.5199932	1.976546558	0.955301537	0.011363961	0.03026027
Lrrc4	9.307451823	2.116760888	2.143255387	0.011380044	0.030290489
B130046B21Rik	12.63929454	1.631798365	0.855530291	0.011377339	0.030290489
Ms4a6b	8.732021075	2.119147188	1.332237346	0.011410176	0.030362268
Gprc5b	601.8103835	1.699338239	0.859647544	0.011426308	0.030396766
Mirg	6.077197318	1.714370919	0.988588298	0.011506885	0.030587955

Nhs	19.30302858	2.027792217	1.976826778	0.011513561	0.030594952
Serpini1	5.246819522	2.118541667	2.14209677	0.011536469	0.030651581
Adora2b	4.817169122	2.186310523	2.344849547	0.01155387	0.030685071
D230049E03Rik	6.568291606	2.172661003	1.496662642	0.011643337	0.030903391
Gm26109	5.626024752	2.175329479	1.533370447	0.011738888	0.031133361
Ednra	6.150389198	2.203429916	1.363070267	0.011915881	0.031554806
Gm17055	9.685906737	1.361969598	0.690192218	0.011948476	0.031632391
Pdk3	127.8046482	1.99559426	1.198503833	0.011961233	0.031661798
Pp2d1	3.642966945	2.204640844	1.626582709	0.011983848	0.031708538
Gm6525	3.522392175	2.202739186	2.451969916	0.012017528	0.031775747
Chst11	22.62992807	2.062343693	1.289595136	0.012022026	0.031783263
Oscp1	107.134457	2.009254497	1.209483202	0.0120393	0.031824547
Gm43196	7.514845341	2.075915273	2.168696856	0.012088856	0.03192476
Oip5	30.34520914	1.080690728	0.517979189	0.012139198	0.032053296
Pdgfrl	17.03264509	1.995740912	1.973412802	0.012156822	0.032091
Gm16685	19.05921856	2.099454992	2.360650515	0.012196874	0.032179022
Klf10	1032.178549	1.06379289	0.515208029	0.012226206	0.032247541
LXEJ02004842.1	6.846537566	2.12670562	1.486694949	0.012246444	0.032278741
Dctd	72.43420448	1.956657097	0.974367494	0.012245542	0.032278741
Ttc28	195.9291395	1.236091039	0.593658142	0.012251158	0.032286731
Cyp2a4	1597.00709	1.563997261	0.961775814	0.012277285	0.032333202
Tnfsf13	14.00877902	1.550401407	0.793671011	0.012338345	0.032467458
Gm10941	8.461388809	1.665546967	0.925688336	0.012485223	0.032808971
Angptl7	3.703649572	2.113259498	1.448578865	0.012506538	0.032854204
Ppic	89.98069139	1.333122139	0.679420879	0.012517407	0.032875537
Sstr3	8.594621682	1.705323995	0.984520102	0.012537551	0.032919434
4930458D05Rik	16.45733746	1.219779565	0.622728044	0.012558813	0.032961731
Pacrg	44.73442885	1.66543905	0.838229519	0.012573463	0.032991158
Gm47644	5.114632496	1.686231421	0.995935737	0.012576269	0.032993649
Trib2	31.55208516	1.44470454	0.743231443	0.012577851	0.032993649
Gm43920	2.674949527	2.177245237	3.416797226	0.012593151	0.033024755
Gm34408	2.062744495	2.185380999	2.507779283	0.012630523	0.033100147
Mir3100	2.261605798	1.822323053	5.333204845	0.012636946	0.033112457
Gm13199	11.53778192	1.469618856	0.76427744	0.012679219	0.033218688
Ehf	2155.407858	2.071610557	1.285472075	0.01272191	0.033321439
Gm43912	12.87209034	1.450462229	0.773109708	0.012853522	0.033620273
Gm9316	6.173257469	2.078919315	1.413007015	0.012869798	0.03365367
Gm13590	7.558547287	1.978782434	2.016558686	0.012916065	0.033760855
Cr2	6.630804246	1.477331493	0.799581639	0.012958145	0.03385016
Cdh9	8.48032186	1.97953677	2.017175154	0.013014461	0.033981023
Arhgap27os3	7.581260791	2.095487334	1.490498839	0.013049054	0.034052815
Gm12043	3.321890875	2.114949078	1.54068813	0.013106941	0.034185281
Gm22728	5.987232346	2.015660593	2.186319883	0.013182371	0.034340013

1700001K19Rik	6.608053545	1.967206455	2.016952475	0.013194109	0.034361264
Mybl2	47.36640821	1.025146516	0.478312636	0.013235769	0.034451058
Krt17	81.13175066	1.889216684	1.942816545	0.013288341	0.034573829
Gm10277	16.77985667	1.228434959	0.630483214	0.013316777	0.034633726
Ranbp3l	15.24178826	1.202258367	0.617719774	0.013319536	0.034636207
Nkain2	28.28272803	1.910133454	1.959505569	0.013382432	0.034776203
Stard6	21.42168109	1.678628929	0.887357884	0.013432991	0.034893411
Gm49493	3.811999055	2.021332801	2.17734286	0.013449386	0.034931272
Sult1e1	13.16924194	1.775503573	1.329845161	0.013513178	0.035068478
D430013B06Rik	15.07799609	1.259837283	0.656177283	0.013610284	0.035291847
Psmc3ip	39.81813451	1.179043872	0.585139398	0.013742399	0.035586343
Gm45495	22.55597297	1.021985487	0.490175519	0.013746941	0.035593301
Plcd1	80.30223565	1.815185077	1.813252912	0.013814946	0.035754906
Plekho1	48.68357418	1.030424813	0.489907703	0.013840188	0.035815404
AI854703	13.46673192	1.900274404	1.997253673	0.013908994	0.035959519
Sgpp2	249.1210363	1.801777605	1.811443847	0.01410717	0.036427705
Gm128	2.302288517	2.027088926	2.374655139	0.014199512	0.036626724
Calr4	82.53854361	1.8017779	1.820447167	0.014261536	0.036766944
Prkar2b	63.04920691	1.933275842	1.269127172	0.014411514	0.037103753
4833422C13Rik	30.00353163	1.034008201	0.505836127	0.014487764	0.037255082
Dtna	100.0986315	1.778169074	1.809995696	0.014528382	0.037349523
Tril	71.6891821	1.778009855	1.815827702	0.014622757	0.037556924
Gm19552	2.543286262	1.979168578	2.785918681	0.014775922	0.037909721
Amn	8.829127038	1.613306458	1.02988577	0.014819641	0.038004069
Gm14137	10.90735675	1.665313628	0.907790126	0.014826985	0.03801532
Fbln5	7.237044337	1.612545948	0.938451272	0.014866287	0.038105906
Pkdrej	13.84946535	1.350800161	0.765668856	0.014920996	0.038235924
Gjc2	3.659464191	2.032741907	1.695380475	0.014997815	0.038422515
Gm49730	4.088195752	1.88733596	2.044109436	0.014997777	0.038422515
Gm42791	3.775862461	1.932914831	2.395401895	0.015047033	0.038522895
Best1	13.94106333	1.912823988	1.275987021	0.01507517	0.038574346
Dipk1b	47.91101185	1.767672668	1.834538345	0.01511665	0.038665019
Gm43445	6.677196898	1.863281904	2.01971939	0.015120313	0.038669233
Tmem171	89.03939086	1.953383001	1.626834771	0.015181532	0.038820249
Gm5406	287.4628938	1.585579259	0.831565127	0.015208283	0.038852791
Mir7664	3.324430451	1.912284957	2.761165762	0.015215225	0.038860179
Scg5	6.744962052	1.855813001	2.032990417	0.015238169	0.038903247
Tmem231	201.3557733	1.858124216	1.197089569	0.01531784	0.039075456
E130317F20Rik	29.44808241	1.051836524	0.5267478	0.01541244	0.039285447
Akt3	106.3921158	1.356457342	0.713214678	0.015475854	0.039402176
Paqr6	26.03897718	1.765484168	1.853431253	0.015486087	0.039420811
Ggt5	2.927429784	1.871892939	2.886705309	0.015518336	0.039492426
2610028D06Rik	13.84294097	1.700676602	0.999213909	0.015626016	0.039729581

Agpat4	102.1801253	1.728700806	1.812159623	0.015633653	0.039743732
Rps2-ps9	4.394210282	1.86931382	2.223348449	0.015678355	0.039846815
9230116L04Rik	3.564384409	1.87342925	2.583291384	0.015695624	0.03987746
4833408A19Rik	8.264159065	1.672345634	0.952766558	0.015696646	0.03987746
4930546K05Rik	2.509753153	1.759761263	3.350774643	0.015804111	0.040099279
Gm3235	2.907558384	1.41758886	2.937905316	0.015906735	0.040331106
Col14a1	122.6544386	1.263225863	0.716704865	0.015916588	0.040340101
Dram1	189.4635097	1.444353165	0.768409376	0.015921487	0.040347187
Insyn2a	7.716941292	1.921317265	1.362443197	0.016107138	0.040774749
Igsf3	277.1340391	1.407765972	0.738849838	0.016171111	0.040914937
Gm43745	3.510881433	1.821112318	2.707724017	0.016208154	0.040981645
Pkp1	24.83659901	1.668995047	0.953212464	0.016233583	0.041035128
Mdk	426.8659394	1.823691493	0.985743893	0.016238347	0.041041764
Gm13003	8.82700094	1.807247458	2.042813855	0.016248183	0.041061218
Gm38411	3.525215377	1.834926503	2.402485146	0.016351944	0.041296245
Gm42462	21.58282573	1.878294071	1.328211663	0.016359327	0.041309453
B230377A18Rik	3.884645739	1.820160536	2.220450546	0.016593563	0.041801924
4921507P07Rik	52.29781034	1.107688739	0.567141769	0.016738169	0.042121978
Efcab12	37.74578346	1.840645364	1.239111979	0.016794853	0.042242467
Etv4	263.4641707	1.819339961	0.997147044	0.016803964	0.042254307
Gm15527	2.426759365	1.128953113	2.173914055	0.016922083	0.042515362
Gm26770	15.52200165	1.485999981	0.841689573	0.017064821	0.042854094
Gm29036	1.933820075	1.650274036	2.952681818	0.017274264	0.043317724
Plk-ps1	9.86103978	1.415066299	0.804917231	0.017317431	0.043397599
Gm20400	6.587290003	1.735821234	2.251263362	0.017348018	0.043451554
Gm21781	37.85804223	1.009343172	0.513354147	0.017388314	0.043535437
Mir199a-1	3.720406918	1.743996109	2.422253572	0.017399067	0.043550996
Ppfia3	3.087612223	1.786831829	2.197082698	0.017403275	0.043555848
Gucy1b1	5.84592864	1.581779932	0.978038405	0.017417722	0.043586321
Gm15910	5.031832868	1.752808257	2.05298509	0.017465319	0.043699732
Gm45822	2.220171202	1.798616531	2.247390223	0.017468737	0.043702586
Slc6a19	19.52204303	1.586417562	0.899955657	0.017661351	0.044115448
Gm16059	6.518785437	1.730931342	2.022593579	0.017739056	0.044269209
Ssc5d	8.781582222	1.691800716	1.882683219	0.017780253	0.044360482
Gm40309	4.833785372	1.949110469	1.364582114	0.017906804	0.044624484
Cenpu	23.59966192	1.107746464	0.573996902	0.017972962	0.044765622
Tmprss15	3.734876872	2.043540211	1.651543727	0.018013083	0.044853909
Gm39460	10.97686744	1.182421811	0.646402213	0.018038791	0.04490627
Gm37876	5.529903367	1.702184502	2.224131634	0.018144278	0.045139595
Gm19721	8.019660911	1.681878089	1.881553835	0.018159398	0.04516467
Gm12977	3.552256092	1.730459807	2.20738105	0.018168147	0.0451697
Gm5224	4.196724465	1.709788337	2.331401496	0.018275185	0.045412857
Adamts9	1474.426207	1.245982774	0.666436654	0.018358708	0.045602118

Rerg	3.250120917	1.540944	2.630218305	0.018371736	0.045628572
Capsl	112.9285285	1.148500366	0.59143535	0.018443725	0.045766579
Gm7809	6.347204834	1.694408822	2.152967621	0.018480962	0.04584645
8430423G03Rik	2.506559871	1.708629972	2.373083074	0.018533426	0.045958778
Duox2	27.63473952	1.811253197	1.02828046	0.018681076	0.046277078
Edn1	100.9704134	1.60806293	1.81486893	0.018703785	0.046321375
Hesx1	3.476572002	1.634945448	2.468487805	0.018763467	0.046463188
Gm16234	4.646105372	1.690827502	2.154414211	0.018786529	0.046502293
2810455O05Rik	11.04567627	1.454205374	0.839251775	0.018827964	0.046592838
Gm43362	11.56862139	1.444533329	0.88600621	0.01886351	0.046656737
Gm44014	5.470627906	1.562951756	1.011190581	0.018876701	0.046679928
2610318M16Rik	9.430813731	1.648589274	1.884356321	0.018887993	0.046699237
Gm47071	30.30340303	1.001549772	0.511474519	0.018974141	0.046882031
Gm21112	6.24910811	1.810433609	1.45646827	0.018980665	0.046892115
Macroh2a2	128.8549198	1.096890989	0.572355893	0.01910822	0.047158675
Gm48838	11.70605484	1.61045187	0.985438007	0.019120589	0.047183133
Fbxo48	11.79454991	1.787375298	1.334450527	0.019175381	0.047287939
Mchr1	30.92040084	1.830535497	1.165292309	0.01920525	0.047343347
Gm42615	3.441358014	1.821869102	1.654172518	0.019313722	0.047574078
Myl9	30.57591637	1.764051484	1.279618969	0.019424597	0.047828772
Esco2	66.82551036	1.054991191	0.566032707	0.019536036	0.048072327
Gm36787	12.68743537	1.114443455	0.609635762	0.019588644	0.048171293
Gm42587	3.286162191	1.648602599	2.193389351	0.019603113	0.048194125
Gm26413	6.141299459	1.628547524	2.057627811	0.019607264	0.04819726
Zfp503	9.461740171	1.588158706	0.966125526	0.019609411	0.04819726
Serpina3d-ps	2.280879263	1.169170093	2.289104787	0.019643924	0.048257365
Gm42819	4.434196752	1.640606467	2.049523411	0.019656252	0.048281471
Ccl5	10.20942871	1.786896487	1.421070884	0.019684409	0.048338259
Greb1	24.27093453	1.844112887	1.320360341	0.019809811	0.048590245
Col23a1	21.68003973	1.748494381	1.299070461	0.019821541	0.048607864
Gm49959	5.987021957	1.557779279	2.29360958	0.019912551	0.048786131
H2-DMb1	44.3842243	1.40374117	0.800275171	0.019940786	0.048831347
Gm22362	2.746342044	1.508904723	2.45651245	0.020057474	0.049041023
Nsl1	35.0112321	1.482764734	0.931191848	0.020079322	0.049088186
Gm24430	3.097292663	1.591408657	2.300550139	0.020132285	0.04920513
Gm44085	3.482229855	1.579076731	2.283730455	0.020212293	0.049362956
Gm47218	12.28044485	1.241016282	0.708057835	0.020369084	0.049726892

Downregulated genes in Rep_early v.s. Hep

Gene_id	baseMean	log2FoldChange	lfcSE	pvalue	padj
Slco1a1	3231.833501	-8.155338771	0.228416059	5.37E-280	5.15E-276
Cyp4a14	957.4810408	-6.660245808	0.293704887	8.24E-115	8.32E-112
Cyp4a10	1455.843269	-6.473524164	0.293131506	3.38E-109	2.95E-106
Exoc4	1240.354355	-1.973229567	0.094071177	1.59E-98	1.22E-95
Ccng1	1756.337645	-2.539124388	0.123349334	8.50E-95	5.83E-92
Mdh1	6692.928416	-1.58725566	0.078256822	3.90E-92	2.58E-89
Dcxr	970.2880377	-2.650249035	0.131622353	3.39E-91	2.17E-88
Rdh16f2	901.8944892	-3.181173177	0.165102128	7.90E-84	3.99E-81
Lonp2	4964.667558	-1.286337475	0.068299289	1.16E-79	5.17E-77
Retsat	1990.878222	-2.721065226	0.15047467	4.82E-74	1.97E-71
Pon1	5457.19043	-2.455286053	0.139929937	4.98E-70	1.84E-67
Kyat1	1548.660396	-2.432459765	0.140721374	5.81E-68	2.03E-65
Cyp2c54	2045.687488	-3.977255698	0.231031472	1.09E-67	3.68E-65
Ugt3a1	341.8739437	-4.024371478	0.238947849	8.08E-65	2.46E-62
Aldh6a1	4088.091357	-1.421427248	0.084165193	1.45E-64	4.28E-62
Got1	3111.016453	-1.917838855	0.113844595	1.58E-64	4.58E-62
Ces1b	825.8355939	-2.153339213	0.12806553	2.99E-64	8.56E-62
Ddias	133.2315969	-3.456027597	0.206671035	1.52E-63	4.28E-61
Pcca	1130.442133	-1.265951995	0.075726352	3.30E-63	9.18E-61
Mup3	51356.20701	-6.86420456	0.41331084	3.67E-63	9.92E-61
Il1rap	1299.288952	-2.116105253	0.127224957	4.24E-63	1.13E-60
Acat1	3064.272365	-1.548922763	0.093133673	9.32E-63	2.45E-60
Hibadh	3140.879133	-1.566534891	0.094339704	1.25E-62	3.23E-60
Phyh	14242.3588	-1.767557922	0.106682636	2.02E-62	5.16E-60
Mgst1	26265.261	-1.454535002	0.087766101	2.45E-62	6.18E-60
Acox1	20200.0262	-1.513248965	0.092215809	1.16E-61	2.90E-59
C8g	2198.496907	-1.908470697	0.118189736	1.71E-59	4.05E-57
Decr1	2010.108183	-1.252512104	0.079403515	1.45E-56	3.03E-54
Ppm1k	619.0194521	-2.561785499	0.16317743	1.69E-56	3.48E-54
Nudt7	3653.312666	-3.762544025	0.241137378	5.11E-56	1.01E-53
Cyp2c37	769.3492101	-2.058279292	0.131981768	1.13E-55	2.14E-53
Sucla2	1769.988243	-1.016601407	0.065631381	1.44E-54	2.64E-52
Cyp8b1	2480.616184	-6.574614671	0.435307804	9.28E-53	1.61E-50
Gm31583	130.9256102	-4.591506223	0.30479088	2.33E-52	3.93E-50
Glo1	2621.082912	-1.388159331	0.091875224	3.75E-52	6.21E-50
Scnn1a	247.9029112	-2.837713746	0.188857515	4.04E-52	6.63E-50
Vwa8	2535.465299	-1.183980762	0.080298925	1.07E-49	1.73E-47
Acaa2	7491.870667	-1.292726579	0.087855335	1.52E-49	2.39E-47
Oat	4452.204195	-4.100741006	0.281378695	2.97E-49	4.57E-47
Etfrf1	456.0244435	-1.742800137	0.120148118	1.97E-48	2.95E-46
Cyp4a12b	309.6514245	-6.491080274	0.455461609	2.07E-47	2.98E-45

Kynu	2213.742199	-1.654919413	0.115683981	5.83E-47	8.35E-45
Cyp2e1	26217.94174	-2.834597429	0.199842971	1.02E-46	1.43E-44
Zadh2	1599.427487	-1.012343532	0.071341838	4.62E-46	6.29E-44
Sdr9c7	474.6272133	-3.193574604	0.227630165	4.70E-46	6.35E-44
Fah	5816.048037	-1.35771972	0.096010354	5.68E-46	7.52E-44
Acadsb	1773.716809	-1.642687856	0.117301234	3.01E-45	3.78E-43
Pdcd4	1660.903775	-1.529983886	0.10936494	4.09E-45	5.06E-43
Rmnd5a	2347.188286	-1.164718207	0.08307382	5.10E-45	6.27E-43
Lgals9	6484.278941	-1.281578178	0.091707244	6.27E-45	7.66E-43
Atp5pb	3803.614256	-1.003432967	0.07168266	1.07E-44	1.29E-42
Suox	1211.369839	-1.784363926	0.12858541	1.46E-44	1.75E-42
Glul	3131.906545	-2.679792055	0.194128622	1.76E-44	2.09E-42
Mmut	2419.140154	-1.451865312	0.104671996	2.19E-44	2.59E-42
Gpld1	3098.770974	-1.140681867	0.082170646	3.01E-44	3.52E-42
Cmbl	1582.215554	-1.746297331	0.126627982	5.11E-44	5.90E-42
Dpy19l3	414.095172	-2.253034397	0.163856361	6.36E-44	7.26E-42
Zfp874a	373.1033332	-2.018231254	0.146669151	6.58E-44	7.47E-42
Ces2e	4018.950125	-2.356748062	0.173210025	3.72E-43	4.13E-41
Rmdn2	619.4964329	-1.423596563	0.104139246	3.93E-43	4.33E-41
Sdhb	2021.044614	-1.22440423	0.089713697	6.35E-43	6.97E-41
Sulf2	737.8486678	-3.405075229	0.252835417	1.60E-42	1.73E-40
Dglucy	996.5419743	-1.530209742	0.112979438	1.85E-42	1.99E-40
Ces2a	2508.586908	-2.561341509	0.190100434	2.08E-42	2.22E-40
Adtrp	584.4179189	-1.750152402	0.1293789	2.71E-42	2.86E-40
Dhrs1	1686.328456	-1.156801121	0.085533304	3.95E-42	4.10E-40
Blmh	878.6867204	-1.233203009	0.091225594	4.09E-42	4.22E-40
Etfdh	3039.980827	-1.412518694	0.104913147	6.09E-42	6.22E-40
Decr2	1912.30249	-1.417049048	0.105707731	7.77E-42	7.84E-40
Amy1	1792.166186	-1.33907014	0.10091992	9.02E-41	8.97E-39
Sdr42e1	1075.628772	-1.544847916	0.1167433	1.24E-40	1.22E-38
Cyp4a32	174.0791167	-3.609391447	0.275295684	1.68E-40	1.64E-38
Mmd2	124.1383738	-3.762866366	0.288808929	6.39E-40	6.10E-38
Lrtm1	138.4370808	-4.776193412	0.36755822	1.11E-39	1.04E-37
Lman2	3667.76517	-1.010167461	0.077494313	1.12E-39	1.05E-37
Gm3734	77.73279874	-4.133840328	0.31908453	1.71E-39	1.58E-37
Cd1d1	2649.734967	-1.181738938	0.091671	1.72E-38	1.56E-36
Serpina1b	48559.93478	-1.442946183	0.112628622	3.43E-38	3.07E-36
B630019A10Rik	383.3661657	-1.712065482	0.134171978	5.22E-38	4.64E-36
Gcdh	4483.33069	-1.528541691	0.119739788	6.44E-38	5.67E-36
Acad11	3179.576997	-1.282824485	0.100507098	6.57E-38	5.76E-36
Hsd17b4	4942.699386	-1.101179157	0.086518225	1.52E-37	1.30E-35
Rtn4ip1	375.6156007	-1.250075949	0.098422992	1.76E-37	1.50E-35
Ceacam1	1774.233113	-1.692091098	0.133774411	1.94E-37	1.65E-35

Amt	665.4295758	-1.472989033	0.116604755	3.29E-37	2.77E-35
Crot	8715.258427	-1.908630319	0.152514906	9.62E-37	7.99E-35
Cpox	1623.956429	-2.189605324	0.175232736	1.03E-36	8.50E-35
Gm10319	468.3267566	-2.3544645	0.188220823	1.11E-36	9.14E-35
Commd3	1397.901892	-1.088953569	0.086643171	1.23E-36	1.00E-34
Ei24	2834.461251	-1.112020997	0.088605821	1.48E-36	1.20E-34
Tuba4a	2315.478556	-1.725202486	0.138411437	1.71E-36	1.39E-34
Marchf6	2726.655581	-1.03606771	0.082600965	1.81E-36	1.46E-34
Aldh7a1	6689.508536	-1.346483679	0.107817829	2.25E-36	1.81E-34
Scp2	35011.92052	-1.708716976	0.137037618	2.56E-36	2.05E-34
Lifr	2127.39759	-2.177552772	0.175930126	4.45E-36	3.50E-34
Gm3839	231.9304227	-4.682745767	0.380804438	4.72E-36	3.69E-34
Acpp	101.0676281	-5.564999398	0.450012723	5.31E-36	4.13E-34
Rnf103	1672.8662	-1.244072505	0.100752617	1.58E-35	1.21E-33
Cmtm6	1799.804743	-1.145183347	0.093192647	3.67E-35	2.79E-33
Bet1	721.1215644	-1.34285523	0.109576724	4.39E-35	3.33E-33
Cyp2c50	5996.0609	-2.904267997	0.238920963	5.54E-35	4.17E-33
Fetub	6598.019926	-2.097107324	0.172533539	7.45E-35	5.58E-33
Ahcy	11950.41057	-1.444425856	0.119178137	2.25E-34	1.67E-32
Acsl1	6853.524814	-2.155042533	0.179837264	3.62E-34	2.64E-32
Arhgef37	162.4489336	-2.640792894	0.220584051	4.75E-34	3.44E-32
Rgn	6180.406756	-2.749865054	0.230263686	7.28E-34	5.21E-32
Pnpla8	2023.679281	-1.12968343	0.09409742	1.24E-33	8.76E-32
Cyp4v3	5061.225712	-1.550183594	0.130322819	3.39E-33	2.38E-31
Endog	510.526049	-1.964108923	0.166302067	5.33E-33	3.70E-31
Atp8b5	91.11542403	-5.132207148	0.436548214	8.35E-33	5.68E-31
Hectd2os	147.589944	-4.211624709	0.362516209	2.12E-32	1.43E-30
Cpt2	1647.538098	-1.413094992	0.12063766	2.75E-32	1.83E-30
Lama3	384.3308152	-3.68321651	0.317103575	5.17E-32	3.41E-30
Serpina1d	23703.15904	-2.518734662	0.218750786	6.61E-32	4.33E-30
Ndufb5	909.5620495	-1.19925546	0.103220745	1.12E-31	7.22E-30
Pnpla7	1316.832108	-1.108179886	0.095769084	1.82E-31	1.16E-29
Cyp2c67	1062.441841	-3.27262527	0.286481934	2.42E-31	1.54E-29
Tars	1991.618392	-1.422392885	0.123745552	3.38E-31	2.14E-29
Aadat	705.0290745	-1.927257261	0.168748185	4.24E-31	2.68E-29
Slc30a10	412.4097797	-2.77313028	0.244147895	5.40E-31	3.38E-29
Nars2	324.4498423	-1.758378987	0.154882842	1.30E-30	7.97E-29
Abcd3	6075.360782	-1.624408421	0.143001771	1.37E-30	8.37E-29
Dpyd	5085.169895	-1.526509209	0.134091568	1.71E-30	1.04E-28
Pxmp2	769.3839394	-1.504301377	0.132772851	2.08E-30	1.26E-28
Ncald	313.2759059	-1.871845423	0.165654934	2.18E-30	1.31E-28
Agmat	1547.729597	-1.6670846	0.148165458	2.85E-30	1.70E-28
Thnsl2	904.7990693	-1.00484842	0.08855091	3.25E-30	1.94E-28

Mccc2	854.8531618	-1.168674561	0.103133714	3.41E-30	2.02E-28
Cyp7b1	930.2511745	-3.292929138	0.294404845	4.13E-30	2.41E-28
Cox7a2	1252.534603	-1.015613353	0.089666415	4.13E-30	2.41E-28
Aldh9a1	3308.955386	-1.143234836	0.100873835	5.27E-30	3.05E-28
Tubb4b	3591.948875	-1.3887924	0.12394216	9.43E-30	5.34E-28
Adh6-ps1	195.9580178	-3.370270309	0.304430523	1.32E-29	7.44E-28
Irgm1	911.9365283	-1.296067284	0.11615905	1.90E-29	1.06E-27
Hal	4532.346834	-2.626095942	0.238100636	2.08E-29	1.16E-27
Gstz1	5725.168937	-1.361283779	0.122594827	3.17E-29	1.74E-27
Serpina1c	11044.97746	-1.539334727	0.139476588	5.31E-29	2.86E-27
Paip2b	435.6410582	-1.526052505	0.138204026	5.60E-29	3.00E-27
Slc10a1	4469.959654	-2.251048621	0.205136552	5.67E-29	3.02E-27
Serpina1e	33093.53306	-4.746396576	0.434814696	5.69E-29	3.02E-27
Srd5a1	566.4992275	-2.620386367	0.240030131	9.15E-29	4.79E-27
Atp11c	2915.515123	-1.302392418	0.118574038	1.06E-28	5.53E-27
Sec24d	1963.099585	-1.104401679	0.100296646	1.29E-28	6.70E-27
Adh4	927.309734	-1.750574047	0.160198199	1.48E-28	7.67E-27
Cbs	5761.136914	-1.518435039	0.13904252	1.80E-28	9.26E-27
Pecr	1576.428862	-1.553762476	0.142568879	2.67E-28	1.36E-26
Ephx2	5638.831254	-1.221048587	0.112021293	3.01E-28	1.52E-26
Clec2h	140.0322225	-3.802940333	0.353446548	3.25E-28	1.64E-26
Ethe1	1090.014811	-1.847738526	0.170353764	3.37E-28	1.70E-26
Adh5	4083.387451	-1.034252913	0.094792728	4.24E-28	2.12E-26
Fermt2	1856.403137	-1.036901341	0.095344441	6.17E-28	3.06E-26
Alas2	630.1115107	-2.929192705	0.273228144	6.52E-28	3.23E-26
Gm4952	808.0923256	-2.511536321	0.234157676	7.35E-28	3.62E-26
Nbas	835.6906295	-1.251433267	0.115966904	1.19E-27	5.82E-26
Mindy3	577.7466443	-1.161390654	0.107631601	1.35E-27	6.56E-26
Mdm2	1354.690223	-1.254709691	0.116749335	1.83E-27	8.85E-26
Aldh1l1	17153.78296	-1.711813156	0.160273956	1.87E-27	9.01E-26
Tspan33	253.4158232	-2.258691877	0.212080697	2.16E-27	1.03E-25
Mbl2	6891.189503	-4.533134575	0.428568858	2.26E-27	1.08E-25
Fpgs	1692.398453	-1.555621757	0.146429741	4.56E-27	2.14E-25
Ivd	2440.98636	-1.419968506	0.133705311	5.83E-27	2.73E-25
Gbp10	43.84532083	-4.327395819	0.412992023	1.25E-26	5.76E-25
Cisd1	1209.97067	-1.099060832	0.10378337	1.26E-26	5.79E-25
Pgpep1	1420.750066	-1.273966274	0.12065596	1.26E-26	5.80E-25
Trp53inp1	4879.480625	-1.802949001	0.17149749	1.27E-26	5.82E-25
Lamp2	10509.48394	-1.092099902	0.103448383	1.80E-26	8.16E-25
Uso1	2150.984933	-1.0515924	0.100014392	2.96E-26	1.32E-24
Nit2	1454.790917	-1.012184198	0.096803788	5.78E-26	2.55E-24
Igfals	1327.981817	-1.651826314	0.159924914	6.95E-26	3.04E-24
Car1	65.05378439	-2.685374047	0.260899355	8.41E-26	3.64E-24

Bbox1	1261.82703	-1.110227937	0.107105023	1.31E-25	5.64E-24
Clock	1151.18612	-1.110334802	0.10714877	1.36E-25	5.82E-24
Klf9	2054.218084	-1.432349905	0.138797788	1.41E-25	6.04E-24
Sort1	1877.760543	-1.560391304	0.15149648	1.63E-25	6.95E-24
Ubr3	2560.489468	-1.068619521	0.103189687	1.72E-25	7.27E-24
Sephs2	7898.028626	-1.264942124	0.122907036	1.86E-25	7.85E-24
Etfa	3659.776315	-1.045799093	0.101222006	2.04E-25	8.58E-24
Mpv17l	340.9810783	-2.335271168	0.229132235	2.45E-25	1.02E-23
Tfpi2	590.9337248	-2.067051381	0.202806119	2.86E-25	1.18E-23
Cyp2d40	288.3421865	-2.558826117	0.251723602	2.86E-25	1.18E-23
0610030E20Rik	550.7116438	-1.082782673	0.10539226	3.51E-25	1.44E-23
Slc31a1	2630.225584	-1.10986936	0.109193373	4.63E-25	1.89E-23
Ldhd	2174.866764	-1.219324917	0.119582169	5.40E-25	2.19E-23
Tex2	2484.501089	-1.027672342	0.100527501	5.73E-25	2.32E-23
Ttc39c	4446.110703	-2.183785322	0.21585402	6.36E-25	2.56E-23
Sesn2	418.6599438	-1.667181304	0.165508425	1.45E-24	5.77E-23
Cd302	2259.739564	-1.388967565	0.137820649	2.00E-24	7.81E-23
Acsl5	5281.400977	-1.131016153	0.112241736	2.27E-24	8.86E-23
Cyp2j5	6277.69769	-1.56333438	0.156196391	2.44E-24	9.52E-23
F11	1226.561025	-1.525249948	0.152834122	3.46E-24	1.33E-22
Csrp3	103.5276539	-2.712066808	0.273743682	4.02E-24	1.54E-22
Cat	37763.41738	-1.096861853	0.109329042	4.11E-24	1.57E-22
Enho	174.0155649	-2.027311278	0.204846142	6.05E-24	2.30E-22
Slc27a5	7327.457284	-2.128305617	0.215208446	6.50E-24	2.47E-22
Echs1	3041.637631	-1.037964323	0.104082791	8.23E-24	3.11E-22
Aen	484.0944922	-1.500743548	0.151622406	9.46E-24	3.57E-22
Adipor2	6140.491204	-1.258898085	0.126766193	9.69E-24	3.65E-22
Gm53019	57.0915265	-5.254273301	0.534651364	1.01E-23	3.79E-22
Bscl2	676.9304846	-1.025194761	0.10310531	1.16E-23	4.31E-22
Bach2	164.4623929	-1.728839575	0.175242471	1.18E-23	4.38E-22
Hagh	2232.536391	-1.297625048	0.131302905	1.50E-23	5.53E-22
Slc22a7	142.8917039	-2.904826528	0.297846731	1.62E-23	5.95E-22
Zap70	58.67807472	-3.745911459	0.38521894	2.09E-23	7.62E-22
Mup21	854.3786608	-6.060284584	0.626850192	2.24E-23	8.17E-22
Qdpr	2862.326327	-1.337703446	0.136028567	2.62E-23	9.44E-22
Bhmt-ps1	131.2916615	-3.274101033	0.338354383	2.95E-23	1.06E-21
Clpx	2445.584159	-1.814741441	0.186672023	4.02E-23	1.43E-21
2010315B03Rik	389.9599704	-1.049330356	0.107038412	4.36E-23	1.55E-21
Cyp2d10	9162.501461	-1.35692663	0.138093915	4.39E-23	1.55E-21
Susd4	154.3446031	-8.848544192	0.882111044	4.78E-23	1.67E-21
Aox3	5233.849104	-1.827934952	0.188585085	5.32E-23	1.86E-21
Mpst	1160.581906	-1.50108405	0.154969344	7.94E-23	2.75E-21
Clec2d	770.1801628	-1.559627569	0.162388599	1.59E-22	5.44E-21

Slc19a2	1680.009348	-1.24478646	0.129897468	2.85E-22	9.63E-21
Ghr	4664.985053	-1.671448568	0.175571571	3.29E-22	1.11E-20
Gramd1c	524.3665782	-1.304223405	0.136341416	3.39E-22	1.14E-20
Ehhadh	2941.345095	-1.543835382	0.162098978	3.42E-22	1.15E-20
Elovl5	3343.903538	-1.533803995	0.161887916	6.01E-22	2.00E-20
Dbt	1287.253009	-1.508219802	0.159204857	6.09E-22	2.02E-20
Adhfe1	1281.546138	-1.350557739	0.142739609	7.82E-22	2.57E-20
Lap3	3972.861554	-1.055355521	0.111098649	8.10E-22	2.66E-20
Dnajc28	155.5029066	-1.379688786	0.14627217	1.07E-21	3.49E-20
Phlda3	436.4539258	-2.609378674	0.279822194	1.12E-21	3.65E-20
Albfm1	327.0383192	-1.542737239	0.164068516	1.16E-21	3.77E-20
Tent5c	81.28225421	-4.335161049	0.471057744	2.39E-21	7.63E-20
Slc33a1	804.8745948	-1.239101155	0.132504527	2.73E-21	8.66E-20
Sgpp1	1001.215748	-1.117628597	0.119707973	3.63E-21	1.14E-19
Cyp4a12a	2016.484184	-6.516189965	0.71541282	4.31E-21	1.35E-19
Erbb4	257.6337189	-2.512198723	0.273447401	4.35E-21	1.36E-19
Hsd17b10	1224.27121	-1.197546325	0.128974663	5.00E-21	1.56E-19
Abhd6	379.1311588	-1.216090513	0.131214375	6.11E-21	1.89E-19
Gstk1	901.233392	-1.140158469	0.123092331	7.21E-21	2.22E-19
Abat	4071.826158	-2.160401787	0.236152286	7.84E-21	2.41E-19
Gm20319	240.7847704	-1.93171514	0.211103383	8.30E-21	2.54E-19
Cldn12	1341.156379	-1.018006367	0.109966541	8.75E-21	2.67E-19
Tmem205	3585.068176	-1.11567146	0.121048155	8.77E-21	2.68E-19
Amdhd1	2285.910595	-1.10305088	0.119244178	8.84E-21	2.69E-19
Gfm2	749.4885873	-1.160580437	0.125965867	1.12E-20	3.41E-19
Hsd11b1	4030.393339	-1.989185347	0.21932998	1.67E-20	5.01E-19
Bhmt	28291.84794	-1.945012793	0.215785599	2.00E-20	5.97E-19
C130083M11Rik	64.46695987	-2.304936649	0.254985198	2.04E-20	6.08E-19
Oxnad1	403.4284322	-1.09235833	0.119251034	2.07E-20	6.16E-19
Sult1b1	230.8859947	-2.385750459	0.264690562	2.08E-20	6.17E-19
Gstp1	20998.42207	-1.384083612	0.151996623	2.14E-20	6.32E-19
Gria3	237.8418521	-2.186399651	0.242140915	2.21E-20	6.52E-19
Chpt1	1652.0309	-1.077029892	0.118041331	2.81E-20	8.24E-19
Slco2a1	1684.378736	-1.444612187	0.159118998	3.20E-20	9.36E-19
Agxt	2408.901472	-1.960107522	0.218766188	3.37E-20	9.86E-19
Hacl1	271.9805219	-1.933759612	0.215315026	3.83E-20	1.11E-18
Serpina3k	92629.62925	-2.615991586	0.292892207	4.00E-20	1.16E-18
Sucnr1	286.146724	-2.93376756	0.330041426	5.01E-20	1.44E-18
Ireb2	1947.671418	-1.096294032	0.121470394	6.89E-20	1.96E-18
Cpsf4l	47.67400905	-2.869768738	0.322458943	7.40E-20	2.10E-18
Ebpl	725.6166904	-1.053482133	0.116840232	7.85E-20	2.21E-18
Gm5424	294.2463579	-2.167953737	0.243931411	8.07E-20	2.27E-18
Hsd17b2	1928.589796	-1.574232718	0.176107721	8.29E-20	2.32E-18

Slc22a15	442.4549186	-1.508328188	0.168822535	9.56E-20	2.67E-18
Tmem254a	3434.371436	-1.060612444	0.11826947	1.02E-19	2.83E-18
Grhpr	2484.963076	-1.187574987	0.132446749	1.06E-19	2.94E-18
Mup14	220.2115739	-11.06048669	1.369415625	1.08E-19	2.99E-18
C9	5062.348322	-1.845687865	0.208570976	1.39E-19	3.82E-18
Ero1b	937.4064343	-1.157874689	0.129679986	1.52E-19	4.16E-18
Pdk1	856.9702466	-1.447036961	0.163312262	1.93E-19	5.24E-18
Mug1	47630.71591	-1.966531893	0.223865745	2.22E-19	5.99E-18
Plxna2	794.7845082	-1.760155517	0.200588844	3.27E-19	8.76E-18
Sult2a8	4597.287247	-8.432442461	0.978509713	3.44E-19	9.18E-18
Pbld2	1290.443319	-1.578295277	0.180103561	3.82E-19	1.02E-17
Aadac	5221.332697	-1.634676067	0.186826131	4.10E-19	1.09E-17
Chic1	76.62433719	-7.525369377	0.830090347	5.69E-19	1.50E-17
Tlcd2	949.7024691	-1.64989136	0.18944809	5.88E-19	1.55E-17
Slc25a13	2886.356758	-1.043551198	0.119146024	6.86E-19	1.80E-17
Slc25a42	1176.236855	-1.042468809	0.118946477	7.44E-19	1.93E-17
Enpep	1055.792282	-1.122359445	0.1287227	8.27E-19	2.14E-17
Gm36419	51.08797354	-3.276748686	0.38193606	8.73E-19	2.25E-17
Slc46a1	590.6467949	-1.002634201	0.11458278	9.30E-19	2.39E-17
Gm2788	73.24903233	-3.190205448	0.373007903	1.00E-18	2.56E-17
Tsc22d1	3636.47621	-2.135203582	0.248811637	1.02E-18	2.61E-17
Wdtc1	1945.6282	-1.005742075	0.115878934	1.69E-18	4.25E-17
Fam210a	839.9343699	-1.010876333	0.117020768	2.38E-18	5.89E-17
Ccdc162	182.4211288	-1.517347678	0.177128261	2.58E-18	6.38E-17
Ppdpf	814.6673797	-1.002919085	0.116610603	2.99E-18	7.34E-17
Fam214a	1187.745869	-1.096318189	0.127599263	3.18E-18	7.81E-17
Aaas	378.9971068	-1.162736962	0.136168006	4.74E-18	1.15E-16
Hrg	9857.429302	-1.35650863	0.159326731	5.24E-18	1.27E-16
Gm4756	551.3869792	-1.531727044	0.181542433	7.72E-18	1.85E-16
Slc45a4	462.5715167	-1.099657439	0.129707703	8.70E-18	2.08E-16
Pm20d1	1373.939635	-1.706184579	0.203385638	8.81E-18	2.10E-16
Pnkd	1949.121618	-1.270433715	0.150505674	9.33E-18	2.22E-16
Glyat	2344.817	-1.324979021	0.157166532	9.55E-18	2.27E-16
Abhd3	598.7206968	-1.104845259	0.130761933	1.07E-17	2.54E-16
Urad	520.5567161	-1.510715927	0.1799979	1.09E-17	2.57E-16
Insig2	2707.844627	-2.178644842	0.262670351	1.30E-17	3.04E-16
AU022252	856.6742588	-1.03847757	0.12310557	1.39E-17	3.26E-16
Gbf1	1660.151244	-1.058315763	0.125860693	1.60E-17	3.73E-16
Elac1	188.7118656	-1.369324979	0.163828587	1.72E-17	4.01E-16
Cyp2u1	181.4334045	-2.137142369	0.259283355	2.14E-17	4.94E-16
Atg5	665.5256706	-1.202649826	0.144118052	2.33E-17	5.37E-16
Slc39a8	361.1238199	-1.190460088	0.142929666	2.69E-17	6.16E-16
Scrn3	592.1068952	-1.41623237	0.170773649	2.71E-17	6.19E-16

Slc22a28	30.51193808	-6.060618739	0.721808945	2.85E-17	6.49E-16
Slc25a15	4594.865544	-1.1291272	0.136050027	3.90E-17	8.78E-16
Sardh	8546.791883	-1.56303958	0.189696008	4.00E-17	8.99E-16
Gramd3	581.0196127	-1.051744341	0.126789506	4.17E-17	9.36E-16
Ppp1r3c	791.1485404	-1.786775025	0.218125354	4.19E-17	9.38E-16
Gm30262	110.6788181	-2.086815126	0.256556604	5.26E-17	1.17E-15
Cyb5b	6724.019993	-1.231557749	0.148826503	5.40E-17	1.20E-15
Dgka	268.7516958	-1.262506747	0.153579144	5.82E-17	1.28E-15
4931406C07Rik	2753.613811	-1.051192492	0.12721332	5.91E-17	1.30E-15
Ulk2	1403.785086	-1.038946368	0.126733611	9.35E-17	2.01E-15
Cdkn1a	1294.551865	-2.330747782	0.290348643	1.09E-16	2.34E-15
Paqr9	2312.45477	-3.741789312	0.470304753	1.15E-16	2.46E-15
Plbd1	203.1555263	-2.442609093	0.306107279	1.57E-16	3.31E-15
Acot1	646.2609797	-2.833598381	0.357534104	2.02E-16	4.23E-15
Scp2-ps2	87.58193697	-1.947734789	0.244056424	2.22E-16	4.64E-15
Alb	764512.0377	-1.231695404	0.152860701	2.39E-16	4.98E-15
Coq8a	4650.846139	-1.674665186	0.208911252	2.40E-16	5.00E-15
Slc6a12	829.19693	-1.528991589	0.191737429	2.79E-16	5.79E-15
Igf1	5603.819491	-1.483795387	0.186019925	3.42E-16	7.06E-15
Slc34a2	355.0723003	-3.209464554	0.403713718	3.66E-16	7.54E-15
Thada	378.6913192	-1.066921558	0.133589356	5.44E-16	1.10E-14
Gm4951	1130.968667	-1.359542978	0.172046826	6.93E-16	1.39E-14
Gm16157	72.67797902	-2.265889971	0.290279794	6.99E-16	1.41E-14
Ccnc	440.5525056	-1.03656063	0.130689977	8.96E-16	1.78E-14
Ppp1r9a	796.1139589	-1.572235671	0.200377031	8.98E-16	1.78E-14
Tmem19	1666.704453	-1.384664309	0.176004205	9.08E-16	1.80E-14
Ndrg2	10572.0384	-1.248086008	0.158231276	9.30E-16	1.84E-14
Paox	467.222011	-1.823501685	0.234093977	1.11E-15	2.18E-14
Rnf125	1114.113492	-2.427429624	0.313347681	1.18E-15	2.31E-14
Klhl22	440.1582846	-1.080858587	0.137422515	1.38E-15	2.68E-14
Sult5a1	69.8770676	-8.442070724	1.031999217	1.39E-15	2.70E-14
Msrb1	2494.346088	-1.002363559	0.127287096	1.44E-15	2.79E-14
Myo1b	5169.108824	-1.023618871	0.130866519	1.71E-15	3.29E-14
Mfsd4b3-ps	164.9892399	-1.182404271	0.151318922	1.89E-15	3.63E-14
Hpd	11526.28301	-1.940059718	0.252192454	2.04E-15	3.91E-14
Zfp958	172.4961054	-1.408931858	0.181804722	2.35E-15	4.49E-14
Iigp1	3602.198393	-1.392122722	0.180237291	2.55E-15	4.85E-14
Crem	318.6324204	-1.15703146	0.148707938	2.61E-15	4.95E-14
Sugct	300.8437445	-1.030843991	0.132927029	3.65E-15	6.88E-14
Ebp	1992.520831	-1.164280871	0.150907167	4.09E-15	7.69E-14
Slc38a3	18171.6763	-1.202916303	0.155989771	4.26E-15	7.99E-14
Mgll	559.5147826	-1.504583824	0.196852916	4.86E-15	9.05E-14
Pde9a	226.6531847	-1.449744293	0.190688258	6.83E-15	1.25E-13

Nr0b2	428.6848339	-1.891639451	0.250939744	7.35E-15	1.34E-13
Gm5096	303.7068556	-2.766172999	0.37047435	8.17E-15	1.49E-13
Pigr	15895.96808	-1.566450085	0.207538595	8.95E-15	1.63E-13
Rida	5117.22602	-1.133237282	0.149466345	1.15E-14	2.08E-13
Cyp4f17	360.5819921	-1.299350291	0.171834913	1.17E-14	2.12E-13
Ifit3	105.2919954	-1.699621227	0.226796275	1.24E-14	2.24E-13
Bdh2	151.865255	-2.083118718	0.280214606	1.38E-14	2.48E-13
Nat8f1	1071.00527	-1.548307151	0.206948616	1.53E-14	2.73E-13
Lmbrd2	1412.847547	-1.008211514	0.133501035	1.79E-14	3.18E-13
Tmem86b	739.1731778	-1.575900301	0.21163183	1.94E-14	3.43E-13
Ube2e1	323.423937	-1.023029299	0.135507018	1.97E-14	3.47E-13
Gbp11	38.11692445	-2.554886431	0.346695165	2.21E-14	3.88E-13
Chid1	578.5841363	-1.056366871	0.14078556	2.51E-14	4.37E-13
Mgmt	316.5917011	-1.944793689	0.264396532	2.72E-14	4.73E-13
Slc15a5	31.47402286	-6.053374705	0.812925644	2.77E-14	4.79E-13
Slc22a1	2885.581621	-1.521448171	0.205374227	2.76E-14	4.79E-13
Mup-ps13	30.41902683	-10.40536243	2.998579835	3.06E-14	5.28E-13
Cfhr1	1272.927003	-1.764981543	0.241044512	3.06E-14	5.29E-13
Klhl7	227.559492	-1.095433038	0.146668184	3.09E-14	5.32E-13
Cyp4f14	3521.861278	-1.809710029	0.246174708	3.09E-14	5.32E-13
Nxt2	399.4182943	-1.389691644	0.187479086	3.09E-14	5.32E-13
BC024386	1569.127979	-1.470404029	0.199267577	3.34E-14	5.73E-13
Sec23b	1351.920018	-1.071781252	0.143582087	3.55E-14	6.08E-13
Cyp2d9	12943.9436	-1.809794208	0.246808768	3.58E-14	6.12E-13
Extl1	107.5850365	-3.10173759	0.428639657	4.04E-14	6.90E-13
Lhpp	353.9288928	-1.99488667	0.273642952	4.32E-14	7.35E-13
Kmo	2428.998225	-1.578339522	0.215794799	5.22E-14	8.81E-13
Abhd18	271.6325461	-1.405862665	0.191725191	5.51E-14	9.22E-13
Gnat1	380.1575523	-1.36859185	0.186912177	6.70E-14	1.12E-12
Ifi47	428.2855406	-1.178265446	0.160543559	7.07E-14	1.18E-12
Slc1a2	607.4669923	-3.295359641	0.464289965	8.35E-14	1.38E-12
Casd1	601.5378374	-1.139499792	0.156084838	1.02E-13	1.67E-12
Shmt1	2007.223139	-1.044475438	0.142895578	1.07E-13	1.75E-12
Camk1d	872.5918136	-1.613423265	0.223742189	1.08E-13	1.76E-12
Aqp4	36.54572955	-3.834567953	0.544881772	1.43E-13	2.31E-12
Bco2	233.3934605	-1.290899421	0.178959457	1.54E-13	2.46E-12
Slc17a3	598.3860062	-1.366358091	0.189321331	1.56E-13	2.50E-12
Magt1	1463.989814	-1.21847158	0.169065037	1.81E-13	2.88E-12
Gm16551	158.3694224	-2.223388884	0.314185608	2.15E-13	3.41E-12
Pank1	2084.751402	-1.278228916	0.178899004	2.62E-13	4.12E-12
Etnppl	1624.763176	-1.678792451	0.237720976	2.85E-13	4.45E-12
Gm9826	59.4033759	-2.43846031	0.349566323	3.16E-13	4.91E-12
Gstt3	411.7071088	-1.3222338	0.18593062	3.19E-13	4.94E-12

Acsm1	4203.972657	-1.434672031	0.203120025	3.87E-13	5.94E-12
Cadm4	235.0068656	-2.465215915	0.354357922	4.04E-13	6.19E-12
Lipc	1259.221237	-1.031292917	0.144537595	4.25E-13	6.51E-12
Blm	139.4126154	-1.389109789	0.196825822	4.38E-13	6.69E-12
Afm	2380.799946	-1.993233325	0.28504529	4.43E-13	6.76E-12
Fn3krp	226.3381668	-1.134411511	0.159850714	4.56E-13	6.94E-12
Sult2a7	23.50816231	-9.956504639	2.951813459	4.64E-13	7.05E-12
AI182371	3605.079203	-1.50079906	0.213458275	4.71E-13	7.14E-12
Gnmt	12382.48397	-1.69947121	0.243716648	5.03E-13	7.62E-12
Tln2	157.4549379	-1.198013778	0.16964355	5.32E-13	8.03E-12
Cplane1	772.9753829	-1.27780983	0.181511511	5.79E-13	8.71E-12
Tm7sf2	1431.894175	-1.323814902	0.188713959	6.30E-13	9.45E-12
Spout1	326.1234874	-1.103332324	0.156548304	6.74E-13	1.01E-11
Magix	145.2263832	-1.509991728	0.216469012	6.92E-13	1.03E-11
Adgrf1	130.9530229	-3.54573212	0.519724717	7.60E-13	1.13E-11
Rdh16	461.4338517	-2.070121064	0.302301229	8.75E-13	1.30E-11
Mfsd4b1	435.8614984	-1.102750951	0.157668988	9.86E-13	1.45E-11
Acot11	103.345663	-2.331837656	0.341108384	9.95E-13	1.47E-11
Aldh2	26489.0011	-1.183834361	0.169155191	1.06E-12	1.57E-11
Tymp	264.3628247	-1.897303044	0.278472344	1.43E-12	2.09E-11
Gm45425	28.50175919	-6.084059487	0.889268324	1.55E-12	2.26E-11
Mapk15	381.841014	-1.625579215	0.238339337	1.81E-12	2.62E-11
Mreg	392.8654681	-1.740121022	0.255768953	1.93E-12	2.79E-11
G6pc	2487.090987	-1.878114966	0.278221714	2.40E-12	3.44E-11
Celf2	394.3433022	-2.217946188	0.330138913	2.45E-12	3.51E-11
Slc25a21	125.0229346	-1.841809788	0.273731439	2.69E-12	3.84E-11
Gm35696	32.45528046	-2.732339341	0.409110354	2.71E-12	3.86E-11
Ifit1	179.401872	-1.673887975	0.248259951	2.96E-12	4.21E-11
Afmid	436.4352751	-1.419208296	0.209587158	3.11E-12	4.41E-11
Ddit4	877.0347367	-1.651004447	0.245006316	3.15E-12	4.47E-11
Ugt2a3	2492.393957	-1.797857339	0.26917212	3.76E-12	5.29E-11
Zbtb5	239.0530269	-1.182575621	0.174380449	4.09E-12	5.73E-11
Ces1e	711.5684012	-1.619295417	0.242279398	4.29E-12	5.99E-11
Gtse1	93.65492005	-1.706034081	0.25537884	4.52E-12	6.29E-11
Nxpe2	148.1812551	-1.632884178	0.244224679	4.73E-12	6.59E-11
Xk	89.35518604	-1.620381713	0.242830439	5.01E-12	6.93E-11
Mup12	37.30375135	-8.374178847	1.36717084	5.26E-12	7.27E-11
Comt	10115.36535	-1.148553849	0.169900741	5.33E-12	7.36E-11
Ccnf	268.1785076	-1.631114052	0.245207684	5.60E-12	7.72E-11
Nlrp12	205.4374206	-3.889419547	0.601120168	5.85E-12	8.03E-11
Ndufaf1	380.0744169	-1.105724106	0.164403068	6.57E-12	9.00E-11
Dock4	707.5407348	-1.02509996	0.152268213	6.77E-12	9.26E-11
Nnmt	860.953606	-2.044747924	0.311153199	6.78E-12	9.27E-11

Arl4a	402.0568203	-1.113983764	0.16599853	7.03E-12	9.59E-11
Gulo	4146.630256	-1.369150228	0.205715636	7.57E-12	1.03E-10
Mcm10	407.1965466	-2.189715637	0.335342724	7.87E-12	1.07E-10
Tspyl4	70.44859373	-2.996293197	0.45936199	8.06E-12	1.09E-10
Tfb1m	142.9438176	-1.283150064	0.193649733	9.69E-12	1.30E-10
Neb	369.7871637	-1.530422731	0.23309829	1.10E-11	1.47E-10
Cth	4751.305475	-1.680340982	0.257599257	1.11E-11	1.48E-10
Cyp3a25	4180.551106	-1.621648835	0.247660521	1.16E-11	1.54E-10
Arhgap26	307.9492233	-1.353005252	0.20591908	1.33E-11	1.76E-10
2310001H17Rik	95.95978737	-1.761449345	0.270727985	1.34E-11	1.76E-10
Cyp27a1	3585.908062	-1.156591098	0.175607241	1.53E-11	2.01E-10
Eda2r	190.2811247	-2.04596097	0.317565229	1.62E-11	2.11E-10
Rhobtb3	395.3741534	-1.03600393	0.156995358	1.82E-11	2.37E-10
Abca8b	1247.531189	-1.05266084	0.160094855	2.04E-11	2.65E-10
Srgap3	49.21790938	-6.926701216	1.011940735	2.06E-11	2.66E-10
Tanc2	226.7021055	-1.304012968	0.200532408	2.16E-11	2.78E-10
Galt	608.0470576	-1.012779624	0.15500227	2.70E-11	3.45E-10
Gm45941	27.03596676	-7.902243389	1.336212488	2.84E-11	3.62E-10
Serpina3n	6404.26128	-1.525045953	0.237006247	2.87E-11	3.65E-10
Igfbp2	2614.063083	-1.439906254	0.224686878	3.34E-11	4.23E-10
Mfsd8	188.7976271	-1.017509355	0.156881782	3.64E-11	4.58E-10
Npr2	507.7869207	-1.256461047	0.195578615	3.95E-11	4.95E-10
Gpcpd1	1025.645231	-1.75502162	0.278506742	4.95E-11	6.12E-10
Dapk1	2039.317286	-1.068430626	0.166663974	4.98E-11	6.16E-10
Odf3b	120.9933682	-1.339665737	0.210217945	5.17E-11	6.38E-10
Hyou1	3725.334432	-1.427315633	0.22539155	5.98E-11	7.32E-10
Ugt2b5	11161.03453	-1.593729243	0.253628819	6.41E-11	7.82E-10
Ces1f	3502.330601	-3.503923322	0.576301842	7.67E-11	9.28E-10
Gmppb	355.6339127	-1.133160655	0.17864389	7.89E-11	9.53E-10
Dmgdh	4270.370996	-1.459233393	0.232238645	8.02E-11	9.66E-10
Gpam	5231.991526	-1.247570008	0.197750343	8.41E-11	1.01E-09
Tmie	290.5164944	-1.4979633	0.239433204	8.61E-11	1.03E-09
Rfx4	17.92621913	-3.812527227	0.624740685	1.07E-10	1.27E-09
Dct	14.13264872	-9.060130993	2.867492884	1.17E-10	1.38E-09
4732465J04Rik	135.2761788	-1.485013187	0.239055635	1.27E-10	1.50E-09
Agbl3	127.0944135	-1.359954194	0.218340225	1.32E-10	1.56E-09
Pxmp4	1264.234575	-1.006066441	0.159752264	1.35E-10	1.59E-09
Asl	5484.97917	-1.055247794	0.168842741	1.44E-10	1.69E-09
Hao1	2381.38118	-1.893178006	0.310653311	1.56E-10	1.83E-09
Maob	2149.151945	-1.217205304	0.196259799	1.56E-10	1.83E-09
Ston2	535.3285869	-1.203681906	0.193743996	1.57E-10	1.83E-09
Lactb2	3890.475783	-1.089285886	0.174520699	1.59E-10	1.85E-09
Slc25a45	295.066893	-1.32902454	0.214947266	1.73E-10	2.02E-09

Abca8a	1399.104272	-2.021831333	0.333243805	1.82E-10	2.11E-09
Cdcp1	1036.676466	-1.178944995	0.190651532	2.05E-10	2.37E-09
Gm11844	44.05439073	-2.016056751	0.334261734	2.20E-10	2.52E-09
Apoa2	29949.05955	-1.291313724	0.215692367	2.31E-10	2.64E-09
Hdhd3	253.4069346	-1.031330394	0.16683294	2.37E-10	2.70E-09
Ahcyl2	561.6757661	-1.06874805	0.17310701	2.43E-10	2.76E-09
Acmsd	296.9193473	-1.559356915	0.256503658	2.57E-10	2.91E-09
Ptk6	28.12952245	-3.932598463	0.668502717	2.59E-10	2.93E-09
Mtarc1	4417.622663	-1.106747907	0.179645401	2.63E-10	2.98E-09
Leap2	123.6363937	-2.726980462	0.460354727	2.65E-10	3.00E-09
Plaat3	217.2705145	-1.494136206	0.245818095	2.82E-10	3.17E-09
Pithd1	340.2520987	-1.000470116	0.162447374	3.08E-10	3.45E-09
Capn8	21.36772011	-4.088464325	0.693902246	3.13E-10	3.51E-09
Cyp4f15	1617.125215	-1.223865134	0.20060506	3.15E-10	3.52E-09
Atp5g3	3736.266072	-1.034609517	0.168610886	3.20E-10	3.57E-09
Cfhr2	7372.005222	-1.125502293	0.184157306	3.43E-10	3.82E-09
Gm28438	95.97393006	-2.726438533	0.46464712	3.59E-10	3.97E-09
Cela1	177.1386406	-1.356107854	0.223862035	3.63E-10	4.01E-09
Ugt2b1	5937.994386	-3.045600475	0.521443887	3.71E-10	4.09E-09
Gm37795	57.84462543	-2.03906616	0.341852069	3.79E-10	4.18E-09
mt-Tn	102.0591981	-1.339532619	0.221319058	3.82E-10	4.20E-09
Tbkbp1	141.1041512	-1.263836683	0.208539451	4.00E-10	4.39E-09
Necab1	535.5719681	-1.062930651	0.17406169	4.13E-10	4.53E-09
Slc13a2	20.44750741	-5.713360829	0.929515461	4.17E-10	4.56E-09
Sphk2	1001.934489	-1.205584096	0.199031688	4.44E-10	4.84E-09
BC049987	73.19393298	-2.333943196	0.398424011	4.81E-10	5.22E-09
Ccdc30	146.632766	-1.680709699	0.282203212	4.85E-10	5.25E-09
Ropn1l	119.2868843	-1.14394496	0.188892214	4.86E-10	5.26E-09
Ube2u	57.71230701	-1.766010533	0.297367868	4.97E-10	5.37E-09
D3Ertd751e	181.7250643	-1.126427281	0.186958483	6.20E-10	6.65E-09
4732419C18Rik	63.37222025	-1.469915351	0.247772708	6.85E-10	7.31E-09
Serpina12	440.3608179	-1.377441427	0.231610957	6.96E-10	7.42E-09
Ifit3b	30.71031413	-2.220563342	0.380433645	7.48E-10	7.94E-09
Isoc2a	1097.025947	-1.135151576	0.189735513	7.61E-10	8.07E-09
Apoo	266.4363373	-1.01118389	0.16842001	8.01E-10	8.48E-09
Gm16548	29.03563962	-2.18789378	0.377737798	8.66E-10	9.15E-09
Lamb3	921.3770423	-1.76319929	0.300415071	9.12E-10	9.59E-09
Gna14	101.152346	-8.074602241	2.703678642	9.80E-10	1.03E-08
Gm48633	155.1112973	-1.289445412	0.21845531	1.01E-09	1.05E-08
Tbc1d30	195.1130102	-1.538811047	0.262868922	1.03E-09	1.08E-08
Cfh	18497.17179	-1.685737067	0.289534381	1.06E-09	1.11E-08
N4bp2	626.9326672	-1.089411876	0.183432126	1.07E-09	1.12E-08
Npas2	70.79666511	-4.119782264	0.727317867	1.10E-09	1.15E-08

Madd	487.8038817	-1.076284577	0.181670876	1.19E-09	1.24E-08
Acss2	2949.095796	-1.714870141	0.296560895	1.23E-09	1.27E-08
Rdh7	9274.605396	-1.130826092	0.192891585	1.37E-09	1.41E-08
Asic5	38.89485239	-2.171238563	0.379476294	1.43E-09	1.48E-08
Gls2	4573.752992	-1.497335333	0.259117028	1.68E-09	1.72E-08
Hapln4	178.2555074	-1.399628434	0.241823794	1.70E-09	1.73E-08
Esrrg	11.23201636	-8.421875994	2.797439887	1.84E-09	1.87E-08
Gm4737	51.83017482	-1.933099547	0.33996069	1.87E-09	1.89E-08
Syt11	182.827561	-1.327836027	0.229238752	1.97E-09	1.98E-08
5730414N17Rik	42.67015591	-2.019333902	0.356506184	2.04E-09	2.04E-08
Otc	4505.54963	-1.544743242	0.269776574	2.08E-09	2.08E-08
Cpeb2	1145.829352	-1.289177194	0.223448905	2.18E-09	2.17E-08
9130409I23Rik	192.8731022	-1.366192475	0.237882991	2.35E-09	2.33E-08
Denn2b	1237.020607	-1.354612141	0.235646135	2.44E-09	2.42E-08
Nat8f2	11488.31915	-1.463874985	0.2566412	2.60E-09	2.57E-08
Gm13456	219.0911845	-1.141414425	0.197652787	2.62E-09	2.59E-08
St3gal3	874.0343593	-1.048226922	0.18070517	2.62E-09	2.59E-08
Amer2	58.93190936	-4.954170543	0.8373534	2.83E-09	2.78E-08
Igtp	410.365261	-1.009129237	0.174285094	2.91E-09	2.85E-08
Impg2	38.99951935	-1.969105716	0.35069631	2.92E-09	2.86E-08
Dock8	727.0900506	-1.053611992	0.18200894	2.96E-09	2.89E-08
Tmem8b	156.0274911	-1.770876184	0.313976329	2.99E-09	2.92E-08
Cspg5	18.946972	-3.087276554	0.556753556	3.11E-09	3.03E-08
Vmn1r90	13.82799156	-8.228141244	2.77154793	3.19E-09	3.11E-08
Asap2	484.8955606	-1.426041717	0.251683117	3.47E-09	3.37E-08
Aass	3323.141373	-1.145107886	0.2002433	3.56E-09	3.45E-08
Nfil3	345.3377128	-1.659791179	0.296170896	3.85E-09	3.72E-08
Gm36264	14.7572191	-2.920302065	0.534842699	4.74E-09	4.53E-08
Fut8	152.5306864	-1.52489724	0.273780148	5.79E-09	5.48E-08
Srms	18.07701278	-4.350919648	0.790067088	5.86E-09	5.54E-08
Sc5d	6522.735873	-1.192082158	0.21252338	6.35E-09	5.97E-08
Gm8883	156.9433376	-1.496838915	0.271763972	6.49E-09	6.09E-08
Hgd	5767.565109	-1.443967608	0.260643051	6.86E-09	6.42E-08
Atp8b4	17.3531573	-2.850738248	0.528514228	6.91E-09	6.46E-08
Lipe	230.9963135	-1.122614009	0.199947309	7.04E-09	6.57E-08
Cyp3a11	34493.26965	-1.422145996	0.256789472	7.10E-09	6.62E-08
Ss18l2	223.317077	-1.161608131	0.207513238	7.22E-09	6.73E-08
Dio1	416.1815905	-1.484782037	0.26989159	8.32E-09	7.71E-08
Isoc2b	177.3147908	-1.246569411	0.22468525	8.86E-09	8.18E-08
Pyurf	268.2008094	-1.008737571	0.180571719	9.71E-09	8.92E-08
Tmem14a	106.0498655	-1.051476831	0.188886868	9.85E-09	9.04E-08
Slc17a8	109.4741187	-1.846261529	0.341984154	1.02E-08	9.32E-08
Sgk2	693.6257907	-1.27059685	0.230304532	1.09E-08	9.97E-08

Pltp	76.11284702	-2.758702513	0.522817179	1.10E-08	1.00E-07
Gbp6	97.31930268	-1.517105106	0.279293458	1.25E-08	1.13E-07
Spns2	2975.431914	-1.042041173	0.188895566	1.29E-08	1.16E-07
Svip	306.4687993	-1.063770727	0.192764536	1.32E-08	1.19E-07
Prex1	47.6812679	-2.639228167	0.500541275	1.47E-08	1.32E-07
Fmc1	165.5128956	-1.120857648	0.204516265	1.51E-08	1.34E-07
Ugt1a9	671.7909745	-1.351691045	0.250469244	1.51E-08	1.35E-07
Cyp2d37-ps	575.130604	-1.40373046	0.259652766	1.56E-08	1.38E-07
Ces3b	2566.255037	-4.84463329	0.951094314	1.57E-08	1.39E-07
Arntl	192.6849049	-3.287463827	0.638549238	1.76E-08	1.56E-07
Ugt2b38	48.51642462	-3.62182585	0.705027926	1.77E-08	1.56E-07
Hsd3b3	1558.612985	-1.657230719	0.310738552	1.78E-08	1.57E-07
Slc27a2	7255.737939	-1.110278951	0.204065767	1.88E-08	1.66E-07
Fgf1	737.3589299	-1.558734802	0.292415021	1.95E-08	1.71E-07
Aldh4a1	2507.573566	-1.003584934	0.183757572	1.97E-08	1.73E-07
Apol9b	681.1258834	-1.66424964	0.313601832	1.98E-08	1.74E-07
Gm45871	110.4961469	-1.199708549	0.221605075	2.01E-08	1.76E-07
Zfp951	62.23078478	-1.652736036	0.310980701	2.16E-08	1.89E-07
Abca9	57.00469553	-1.862579224	0.354144353	2.25E-08	1.96E-07
Hsbp1l1	17.9627834	-2.338499316	0.449203209	2.42E-08	2.09E-07
Bbc3	330.5124915	-1.946413906	0.372752075	2.45E-08	2.12E-07
Trmt9b	78.38364621	-1.987449728	0.381027866	2.52E-08	2.17E-07
Gm5621	27.25329181	-2.017616336	0.386980137	2.67E-08	2.30E-07
Gm44243	31.37297085	-2.266665225	0.437968404	2.80E-08	2.40E-07
Med12l	94.44285171	-2.052276416	0.395500979	2.91E-08	2.49E-07
Tmigd1	17.10750995	-3.320607935	0.649632168	2.92E-08	2.49E-07
mt-Te	182.7793095	-1.470773425	0.27943121	3.17E-08	2.69E-07
Tert	316.1095245	-1.213634843	0.22883045	3.51E-08	2.96E-07
Glo1-ps	22.6710828	-2.134269523	0.416152523	3.69E-08	3.10E-07
Pex11g	294.4908088	-1.12141374	0.210988525	3.74E-08	3.14E-07
Ddc	1020.346508	-1.134432505	0.213854294	3.87E-08	3.24E-07
Gm5529	26.35256226	-2.115527092	0.413641034	4.01E-08	3.36E-07
mt-Nd3	350.1705923	-1.499762742	0.288178885	4.13E-08	3.45E-07
Ass1	40142.72455	-1.401246703	0.268652216	4.33E-08	3.60E-07
Apon	3468.369066	-1.125799519	0.212811544	4.32E-08	3.60E-07
9230114K14Rik	40.92338174	-1.79991678	0.349830449	4.34E-08	3.61E-07
1300017J02Rik	6513.128722	-1.248532776	0.238936871	4.82E-08	3.98E-07
Fam151b	50.57162547	-1.469475356	0.283381726	4.94E-08	4.07E-07
Gas1	107.4944131	-1.670518573	0.325963618	5.28E-08	4.34E-07
Gm50136	18.85028508	-4.266012526	0.861203061	5.55E-08	4.55E-07
Echdc1	299.6291448	-1.116756489	0.213008911	5.61E-08	4.60E-07
Fbp1	9434.908133	-1.250957113	0.240378078	5.66E-08	4.63E-07
Slco2b1	1372.102266	-1.380235322	0.269630573	5.69E-08	4.66E-07

Cmah	1943.234371	-2.226498203	0.444788463	5.90E-08	4.82E-07
Gm5524	115.7659489	-1.374618085	0.267199585	6.44E-08	5.20E-07
Inca1	167.934322	-1.188738044	0.2294532	7.08E-08	5.68E-07
Slco1b2	8918.895204	-1.366999714	0.266710746	7.33E-08	5.87E-07
Pde7b	33.4619523	-2.332970473	0.470705071	7.89E-08	6.30E-07
1700007K13Rik	13.80336685	-7.291116065	2.646121654	8.47E-08	6.75E-07
Gm45792	25.65472788	-2.184773802	0.442614191	9.34E-08	7.42E-07
Slc43a1	77.66526007	-2.954706704	0.610170002	9.62E-08	7.61E-07
Xirp1	17.77658105	-2.983696227	0.613354013	9.63E-08	7.62E-07
5033403H07Rik	135.1495714	-1.204424024	0.235304149	9.83E-08	7.76E-07
Haao	3495.218388	-1.216669071	0.238336362	9.99E-08	7.88E-07
Gm36041	64.21392594	-1.896534318	0.3859747	1.26E-07	9.78E-07
Plk2	1956.833191	-1.277811534	0.253047099	1.26E-07	9.84E-07
Gm26684	69.93434537	-1.272635156	0.251938534	1.28E-07	9.94E-07
Slc23a1	875.5246885	-1.025914668	0.20138954	1.42E-07	1.10E-06
Ugt2b36	6904.458939	-1.406881605	0.282092654	1.45E-07	1.12E-06
Gm32461	65.38232439	-1.506897801	0.303978605	1.48E-07	1.14E-06
Slc22a30	1329.295779	-2.341307611	0.488692677	1.60E-07	1.23E-06
Gm6170	35.06236282	-1.717044001	0.348932888	1.62E-07	1.25E-06
Ces1d	4846.406319	-1.26666964	0.253557755	1.66E-07	1.27E-06
Gm30784	27.53769173	-2.609795115	0.549304341	1.71E-07	1.31E-06
Gm15590	84.10377704	-1.314453287	0.26420693	1.74E-07	1.33E-06
Lgalsl	539.7969122	-1.209761524	0.241944347	1.79E-07	1.37E-06
Fam81a	308.2898849	-1.062394098	0.210115983	1.80E-07	1.37E-06
Gm40770	29.03301413	-1.84747738	0.380683929	1.86E-07	1.42E-06
Apol7a	1459.177259	-1.586641378	0.324853109	2.02E-07	1.53E-06
Gm49463	49.70881723	-1.820356233	0.378472126	2.37E-07	1.77E-06
Zfp760	168.7595934	-1.039221805	0.208093571	2.40E-07	1.79E-06
Gvin1	26.21680248	-2.902927533	0.623759124	2.48E-07	1.84E-06
Htra4	10.31369696	-4.5010984	0.939741479	2.52E-07	1.87E-06
Acaa1b	4212.932469	-1.129261474	0.2303716	2.61E-07	1.94E-06
Cyp2f2	8750.464772	-2.044032122	0.432446979	2.70E-07	2.00E-06
0610031O16Rik	118.6314505	-1.395647364	0.28830426	3.10E-07	2.27E-06
Ftcd	5706.832529	-1.139007029	0.232675539	3.36E-07	2.45E-06
Polr3g	125.3474313	-1.117757758	0.228167129	3.39E-07	2.47E-06
Irf5	157.2545146	-1.771578887	0.373620156	3.48E-07	2.53E-06
Gm35164	11.3277361	-6.419631631	1.467072068	3.56E-07	2.58E-06
B4galnt3	33.22448072	-2.126087534	0.456367127	3.92E-07	2.83E-06
Ctcflos	51.00435398	-1.820590451	0.39038017	4.50E-07	3.22E-06
Rnf144a	54.43551313	-2.07343435	0.447535614	4.54E-07	3.24E-06
Fam110a	206.2262549	-1.011498244	0.207537962	4.60E-07	3.28E-06
Gm12909	22.97243683	-3.316328359	0.737162213	4.84E-07	3.45E-06
Cyp4a31	93.13873315	-1.248569911	0.260635616	4.93E-07	3.50E-06

C8b	3746.507413	-1.852659987	0.398559426	4.94E-07	3.51E-06
Sp4	173.6313221	-1.185915764	0.247095976	5.10E-07	3.62E-06
Selenbp2	4616.839794	-2.49718438	0.556492227	6.22E-07	4.34E-06
Gm49431	29.19339011	-1.930807124	0.420901566	6.23E-07	4.34E-06
Tdo2	14666.53154	-1.851407125	0.405290267	6.87E-07	4.76E-06
F9	2549.301488	-1.015947111	0.211897321	6.90E-07	4.77E-06
Egfr	6070.606277	-2.189205139	0.485656438	7.00E-07	4.83E-06
BC023105	50.65969244	-2.025986993	0.4459897	7.03E-07	4.84E-06
Acss3	235.27738	-1.696303887	0.369997503	7.71E-07	5.28E-06
Gpr155	680.0996554	-1.032733407	0.217563034	8.19E-07	5.58E-06
Hmgcs2	18355.00074	-1.085027995	0.230294122	8.95E-07	6.06E-06
Slc25a35	21.6861406	-3.764753117	0.845727995	9.80E-07	6.58E-06
Cyp2d26	7786.721027	-1.031610584	0.217141402	9.88E-07	6.63E-06
Depp1	2367.456344	-1.470427617	0.321716999	1.06E-06	7.11E-06
Nat8f5	79.78016015	-2.840544356	0.65634373	1.10E-06	7.32E-06
St6galnac4	51.12053296	-2.668429318	0.606145291	1.19E-06	7.89E-06
Slc3a1	607.9477193	-1.513876524	0.334112941	1.20E-06	7.94E-06
Rai2	45.47658153	-1.825038352	0.40712445	1.25E-06	8.21E-06
Lrit1	117.4120306	-1.065532447	0.229938677	1.33E-06	8.73E-06
Mup22	187.6972937	-8.599717388	2.115979779	1.38E-06	9.00E-06
Nlrp6	1473.457438	-1.212310835	0.264737618	1.39E-06	9.08E-06
Gm11454	13.77466195	-3.63697022	0.846983616	1.42E-06	9.30E-06
Mup-ps20	11.21091451	-6.349180254	1.552436821	1.43E-06	9.31E-06
Prodh2	1580.196679	-1.358790823	0.300703053	1.53E-06	9.93E-06
Pck1	20769.08758	-1.456535821	0.324854766	1.54E-06	1.00E-05
Celsr1	1462.370175	-1.368046041	0.303605016	1.57E-06	1.02E-05
Apobec1	588.6962835	-1.034948375	0.224629384	1.64E-06	1.06E-05
Ptgs1	42.54643769	-2.098311054	0.47506441	1.64E-06	1.06E-05
Gbp7	217.4561944	-1.100346375	0.240407935	1.68E-06	1.08E-05
Hnf4aos	28.66882509	-1.955908115	0.44905577	1.70E-06	1.09E-05
Pstpip2	313.0269103	-1.1853394	0.261841709	1.89E-06	1.20E-05
Erbb3	2855.224645	-1.052026882	0.230435971	1.89E-06	1.21E-05
Gm30906	11.38690708	-3.986379824	0.950902249	1.91E-06	1.22E-05
Efna3	9.071597771	-3.079098566	0.71517616	1.99E-06	1.26E-05
Gm32281	10.19832376	-3.533422965	0.834979495	2.04E-06	1.29E-05
Plg	23149.35435	-1.080329168	0.238063004	2.06E-06	1.30E-05
Nlrp5-ps	38.30392892	-6.225523891	2.505060972	2.16E-06	1.36E-05
Eef2-ps2	35.16473167	-1.616840508	0.368761514	2.27E-06	1.42E-05
mt-Ty	49.72062794	-1.856784131	0.430653568	2.31E-06	1.45E-05
Ddit4l	493.8019112	-1.043590974	0.23119546	2.45E-06	1.53E-05
Lyrm7	54.67606616	-1.304310198	0.294396762	2.57E-06	1.59E-05
Sh2d3c	103.3775141	-1.365931054	0.311335765	2.91E-06	1.79E-05
Gm6767	26.18073403	-1.547837844	0.357309209	3.09E-06	1.89E-05

BC024139	49.35503527	-1.349122534	0.308800184	3.21E-06	1.96E-05
Gm15417	64.00783042	-1.130855384	0.255239046	3.22E-06	1.97E-05
Serpina4-ps1	160.3037644	-5.18320734	1.334156839	3.25E-06	1.98E-05
Ugt3a2	2728.651214	-2.644524496	0.648995842	3.40E-06	2.06E-05
Gm5869	38.35883434	-1.421770124	0.327078807	3.43E-06	2.08E-05
Cphx1	28.55057676	-1.626864699	0.379637323	3.48E-06	2.11E-05
Izumo4	129.1480145	-1.131606534	0.256499397	3.52E-06	2.13E-05
Zfp125	138.7018529	-1.300241418	0.298346202	3.66E-06	2.21E-05
B3galt1	208.2230636	-1.808404708	0.431212665	3.72E-06	2.24E-05
Glt28d2	80.44252821	-1.11524182	0.253346207	3.74E-06	2.26E-05
Gm32063	32.64284349	-1.826264139	0.433448919	3.76E-06	2.26E-05
Gm38416	33.47614665	-2.779991291	0.683078013	3.77E-06	2.27E-05
Obox4-ps3	4.805751938	-6.878589693	2.731500298	3.80E-06	2.29E-05
Cyp2c68	1720.61075	-1.216304998	0.280381631	4.11E-06	2.46E-05
Adrb3	53.08530868	-1.262207516	0.292810755	4.64E-06	2.74E-05
Asb2	9.078914379	-4.613268452	1.118452063	4.75E-06	2.80E-05
Gm4956	58.92144837	-5.52751578	1.470334446	5.29E-06	3.10E-05
Clstn3	187.6726368	-1.209400752	0.281618424	5.39E-06	3.15E-05
Fam83f	49.37687942	-2.51886035	0.631877965	5.78E-06	3.37E-05
Gm38102	20.10008434	-1.720286468	0.413302425	5.85E-06	3.40E-05
Olfr1033	137.8870896	-1.066768742	0.247176983	5.90E-06	3.42E-05
H2bc21	61.0141481	-1.308659909	0.308379493	5.91E-06	3.43E-05
Aldh8a1	3131.058374	-1.132900889	0.26526503	6.48E-06	3.74E-05
Cfhr4	3571.114705	-1.016146516	0.235805877	6.74E-06	3.88E-05
Akr1c6	10358.50332	-1.290656955	0.306695289	6.80E-06	3.91E-05
mt-Atp8	55.21354411	-1.162791728	0.27391972	7.03E-06	4.03E-05
Gm13340	1442.457308	-1.14938518	0.271106427	7.29E-06	4.17E-05
Mycn	42.32018168	-2.641287552	0.655370665	9.95E-06	5.53E-05
Tpd52l1	102.418502	-1.066724511	0.253706296	9.97E-06	5.54E-05
Kyat3	1641.580225	-1.339983834	0.326938973	1.01E-05	5.64E-05
Ces2c	554.1124305	-1.058875236	0.25355058	1.09E-05	6.00E-05
C8a	8765.084562	-1.22477511	0.297892686	1.14E-05	6.25E-05
Bbs10	76.27645969	-1.327253764	0.325019852	1.17E-05	6.38E-05
1700018L02Rik	21.74536913	-2.096097502	0.53770469	1.18E-05	6.43E-05
Rps23rg1	36.73361085	-1.957323021	0.501065788	1.19E-05	6.49E-05
Amigo2	62.59737571	-1.199921796	0.292057017	1.20E-05	6.53E-05
Gstp-ps	57.66899655	-1.671885757	0.422793921	1.25E-05	6.83E-05
Zdhhc14	56.71879898	-1.176163936	0.286845033	1.33E-05	7.18E-05
Prlr	901.7496998	-1.243718395	0.305902158	1.34E-05	7.23E-05
Gpr12	24.5235708	-1.514704922	0.381964458	1.45E-05	7.80E-05
Bmp7	103.0541266	-1.551016411	0.390102445	1.56E-05	8.29E-05
Igkc	162.6583523	-2.865748096	0.776423999	1.66E-05	8.77E-05
Gm34667	55.09359705	-1.582803349	0.404483944	1.69E-05	8.93E-05

Gm13522	39.39107079	-2.035287636	0.537419783	1.79E-05	9.41E-05
Hhipl1	34.58833831	-1.898909864	0.494019932	1.87E-05	9.82E-05
Wfdc21	2259.028558	-1.267860098	0.321225802	1.97E-05	0.000102551
Slc10a5	528.1282918	-1.164491122	0.291042718	2.00E-05	0.000104024
Gm2814	12.6303219	-2.607079623	0.711918645	2.16E-05	0.000111919
mt-Tw	61.98009368	-1.711027761	0.4490209	2.18E-05	0.000112933
Gm15998	20.91827346	-1.933723838	0.514531888	2.25E-05	0.000116429
1700061I17Rik	11.01779263	-2.463938007	0.656660488	2.39E-05	0.000123006
Cep41	112.9931148	-1.01413815	0.252721532	2.43E-05	0.000124779
Clic5	114.532471	-1.199456387	0.303549121	2.49E-05	0.000127618
Odad3	71.34345341	-1.225909118	0.311746682	2.52E-05	0.000129012
Gm19522	18.02141761	-2.992743086	0.842442565	2.56E-05	0.000130644
Gm40438	21.24035268	-1.852882587	0.497736165	2.75E-05	0.000139729
9330175E14Rik	11.64435636	-2.443181604	0.664743207	2.80E-05	0.000141905
Cyp26b1	12.82435546	-3.185088032	0.895973238	2.92E-05	0.000147359
Gm8566	31.54511448	-1.51385062	0.398565485	2.93E-05	0.00014764
Abcb11	5414.293607	-1.175782571	0.301886161	2.97E-05	0.000149513
Gm44911	4.404663182	-6.551785961	2.812762466	2.98E-05	0.000150208
Osbp2	9.973837542	-2.30271002	0.635331272	3.12E-05	0.000156507
1700028E10Rik	48.09402262	-1.187590187	0.306830546	3.18E-05	0.000159292
AI480526	91.67138818	-1.227957814	0.318027403	3.30E-05	0.000164483
Rpgrip1	18.91572733	-1.890433883	0.508881144	3.31E-05	0.000165083
Tnik	142.4327525	-1.46512248	0.384679698	3.38E-05	0.00016827
Camkk2	295.0702932	-1.048967062	0.268379927	3.44E-05	0.000170984
Fam47e	149.9210212	-1.4629926	0.389297371	3.57E-05	0.000177387
Sspo	17.79419403	-1.722237206	0.465675981	3.85E-05	0.000189733
Etfbkmt	485.1689736	-1.093868737	0.283858698	4.02E-05	0.000197414
Gm26588	23.99824494	-1.557358313	0.420663833	4.04E-05	0.000198049
Tcp11l2	420.1748451	-1.358331576	0.362007655	4.28E-05	0.000209084
1110020A21Rik	17.00214535	-1.597599128	0.434052987	4.44E-05	0.000216507
Gm1600	27.10672324	-2.147561337	0.610588402	4.47E-05	0.000217629
Sned1	246.1928423	-1.058572259	0.27541822	4.50E-05	0.000218954
Fn3k	113.2314708	-1.231184234	0.326463771	4.68E-05	0.000227185
Gbp3	66.83842882	-1.38374793	0.375196271	5.30E-05	0.000254367
Lin7a	128.7885007	-1.754826507	0.491136048	5.30E-05	0.000254367
F13b	2285.429669	-1.10916277	0.293487232	5.37E-05	0.000257671
Gabbr2	212.778895	-1.48633402	0.410276416	5.72E-05	0.000273031
mt-Rnr1	5026.638486	-1.018481365	0.265979537	5.73E-05	0.000273308
Hc	14296.95759	-1.026135281	0.27071618	5.76E-05	0.000274676
Gm43597	46.29901883	-1.382479504	0.375937722	5.89E-05	0.000280284
4833411C07Rik	77.52395679	-1.434891476	0.395105506	6.09E-05	0.000288344
Hhat	87.15673383	-1.296589293	0.350528226	6.13E-05	0.000290035
Gm12669	11.90941133	-1.889705989	0.537390712	6.45E-05	0.000304459

Fitm1	46.20192866	-1.57225439	0.442249759	6.79E-05	0.000319511
Notch1	929.5893766	-1.065811318	0.285398546	6.83E-05	0.000321081
Gm50432	4.630030227	-5.867088108	2.678861987	6.88E-05	0.000323279
Ncmap	229.5432408	-5.041212682	2.379706113	6.95E-05	0.000325839
Vtcn1	294.8244656	-1.277230842	0.347782902	7.07E-05	0.000331113
Ypel2	930.4189795	-1.270606757	0.348827238	7.25E-05	0.000338455
Igha	258.8135344	-2.352599975	0.701778703	7.44E-05	0.00034649
Sh2b2	42.59318919	-1.370631311	0.379378444	7.47E-05	0.000347427
Ighm	4.44484961	-5.761381877	2.663235608	8.02E-05	0.00037117
Ido2	930.1584952	-1.529319435	0.436365038	8.23E-05	0.000380254
Crybb3	29.81360966	-2.911680636	0.90009322	8.44E-05	0.000389144
Cd83	45.87680581	-2.460274718	0.705897376	8.62E-05	0.000396841
Gm6206	15.42147281	-1.856655511	0.538283181	8.85E-05	0.000406588
Sec14l5	11.21496041	-3.501365874	1.105419877	9.20E-05	0.000421403
Hamp2	121.1923577	-2.337349593	0.720043632	9.36E-05	0.000428233
Pcp4l1	28.03302923	-1.354361188	0.381893163	9.44E-05	0.000431812
Selp	8.243238668	-2.971072677	0.912885964	9.64E-05	0.000439708
Adra1b	930.7245795	-1.109674722	0.307298249	0.000100779	0.00045733
Cd59b	38.97008927	-1.376087194	0.392131344	0.000103192	0.000467288
Gm45727	7.6233824	-2.682461087	0.829533917	0.000104121	0.000471159
Nrros	18.05988364	-1.926409724	0.566352991	0.000107014	0.000482884
Cps1	70214.499	-1.50687687	0.437617186	0.000107193	0.00048358
Cfhr3	113.9317049	-1.025716812	0.283341227	0.000111082	0.000499243
Gja10	4.691663376	-4.797467652	1.583107997	0.000112249	0.000504136
Clec4f	54.60471096	-2.253520422	0.698948745	0.000114702	0.00051443
Tmem189	71.32148529	-1.079282428	0.300200657	0.0001157	0.000517938
Gm45338	55.25311974	-1.015752379	0.281309183	0.000116056	0.000519047
Lrrc8c	111.2490945	-1.887726315	0.551048545	0.000117895	0.00052666
Gm14097	27.79823483	-2.078699213	0.639229756	0.00012129	0.000541195
Prox1os	52.47955334	-1.542848162	0.454565219	0.000125134	0.000556533
B930025P03Rik	13.09187764	-3.183173177	1.039307243	0.000126216	0.000560959
Mrc1	32.0589646	-1.770296857	0.533213954	0.000128565	0.000569951
Obox4-ps2	4.11096817	-6.173120329	2.888208974	0.000131556	0.000581996
Atp5pb-ps	12.08419572	-1.750009891	0.523159496	0.000135113	0.000595672
Drc1	68.58000619	-1.527920691	0.448648253	0.000135609	0.000597581
Upp2	1589.728856	-2.003925636	0.624219477	0.000140399	0.000616197
1700001C19Rik	71.9341554	-1.261644707	0.362731544	0.000140919	0.000618143
Slc22a3	21.26046218	-2.022815598	0.628902242	0.000141892	0.000621703
Jchain	55.77767149	-2.408654704	0.750796546	0.000142788	0.00062534
Gm6789	94.99565075	-1.036209726	0.291686336	0.000145836	0.000636801
Mrgprb11-ps	5.197168801	-3.328077099	1.05043512	0.000147108	0.000641481
Caln1	26.24344278	-1.463498014	0.433189292	0.0001485	0.000645934
Car5b	56.19520193	-1.096956469	0.311599104	0.000151663	0.000658937

Gm49024	9.100791611	-2.927127332	0.926784078	0.000152004	0.000659831
Aspa	107.0407348	-1.382612488	0.407462383	0.000156352	0.000677018
4933404O12Rik	39.20438779	-1.342999058	0.394778813	0.000165284	0.000711999
Wdr89	48.13687689	-1.010491989	0.286896304	0.000171651	0.000735304
Siglec1	10.66868237	-3.350971756	1.110297766	0.000184222	0.00078425
1810046K07Rik	15.03150161	-2.590165367	0.861224276	0.000189443	0.000804338
Ackr2	12.13444201	-2.221546882	0.721199699	0.000199598	0.000842419
Gm15746	16.45052063	-1.671110342	0.520065515	0.00020432	0.000860834
Gm15611	7.473531536	-3.701901336	1.273448635	0.000212536	0.000893489
Gm41556	4.992043749	-5.304788902	2.634219569	0.000213944	0.000898819
A630076J17Rik	4.819917767	-3.943787223	1.223926606	0.000216156	0.000907513
Cyp2c23	2472.217608	-1.807660572	0.581407493	0.000229689	0.000959712
Gm12164	18.36929233	-1.582728468	0.491623801	0.00023003	0.000960718
A930007A09Rik	88.24952887	-1.608458691	0.4925813	0.000235635	0.000982203
Gm42568	9.017933735	-2.010017455	0.648181029	0.000243763	0.001011689
Gm45470	46.73531627	-1.18944949	0.355236081	0.000245115	0.001016422
Chp2	7.154584186	-2.468790374	0.81570427	0.000246691	0.001022293
1810008I18Rik	445.7443689	-1.846393954	0.600365402	0.000254751	0.001052965
Slc18a2	31.89179853	-1.263045429	0.385505269	0.000286248	0.001171548
Gm4332	25.17308006	-1.320614431	0.406564472	0.000293988	0.001199899
Stard8	132.1533907	-1.09802691	0.328789569	0.000296546	0.001209568
Gm7895	8.349603608	-2.528926314	0.849926914	0.000313833	0.001272775
Tox2	124.0917439	-1.782428902	0.577904674	0.000316103	0.001281168
Lrit2	25.7335633	-1.315005861	0.4106021	0.000320465	0.001298027
Slc35f1	90.30474071	-4.417135134	2.330719096	0.000320794	0.001299084
Gm40787	7.533144799	-2.716903412	0.957915536	0.000336355	0.001354667
Grem2	128.2819926	-1.085192336	0.32916105	0.000341524	0.001372606
Gm12183	39.77022469	-1.209772025	0.374084509	0.000353299	0.00141283
Tgtp1	189.0783086	-1.100854282	0.336486951	0.000365359	0.001457107
mt-Tl2	78.53900205	-1.463144952	0.471669016	0.000375213	0.001492996
Gm4963	26.87866694	-1.299726823	0.408758765	0.000376717	0.001498048
Gm6542	8.838768437	-2.113123608	0.713491617	0.000377697	0.001501635
5730435O14Rik	7.122775296	-2.645775156	0.933784743	0.000380694	0.001513235
Plag1	13.74807921	-2.28168917	0.758374988	0.000394562	0.001561569
Lrig3	180.1754024	-1.028735152	0.311482867	0.000394829	0.001561978
Nat8	52.55109667	-1.669001015	0.555261818	0.000397921	0.001571619
Gm14279	6.343731913	-2.507405631	0.860942466	0.000414454	0.001632552
Gm5431	12.428755	-4.374155235	2.362810755	0.000416816	0.001641092
Inka2	56.42789364	-1.14813249	0.356308551	0.000419372	0.001649554
Nfam1	5.709927187	-4.249780581	1.59720696	0.000424687	0.001667043
Col25a1	5.17946948	-3.493932451	1.253697492	0.000428224	0.001679897
Adra1a	51.51763476	-1.262193516	0.403701615	0.000469333	0.001826971
Brdt	51.18042461	-1.069490237	0.332632046	0.000471102	0.001832001

Klhl24	1994.956075	-1.006900033	0.311763063	0.000485291	0.001882601
Gm43611	10.74804082	-1.867834216	0.647036553	0.000485393	0.001882614
Rps18-ps5	21.47443429	-1.267595992	0.405035525	0.000489783	0.001897725
Gm11832	12.22925373	-2.144914221	0.76674463	0.000509283	0.001964159
Gm7466	5.152819906	-2.896660578	1.057470988	0.00052914	0.002035017
Gm15564	41.73883714	-1.703124111	0.587098798	0.000537755	0.002066905
A330023F24Rik	20.64642534	-1.44019442	0.478831038	0.000542946	0.002085604
Rnft2	56.9558448	-3.580205582	1.303961886	0.000548138	0.002104706
Rpl39-ps	15.86546197	-1.57112593	0.531474579	0.000575876	0.002199769
Acsm2	27.15450323	-1.694594099	0.589068756	0.00057614	0.00219991
H2bc6	16.65272505	-1.322146476	0.438000162	0.000623235	0.002363737
Insig1	6344.08681	-1.237844804	0.408810278	0.000657831	0.002481703
Srcin1	23.17694225	-2.982754158	1.046451865	0.000662495	0.002497829
P2rx3	20.71700875	-1.169816574	0.382372483	0.000679314	0.002555218
Sox7	8.07751101	-3.616704408	1.37689903	0.000681856	0.002562768
Gtpbp4-ps1	3.955225657	-5.878579997	3.229630729	0.000693565	0.00260321
H3c15	14.72232009	-2.934151177	1.195214171	0.000697794	0.002618568
Gm2996	4.792976417	-2.746614928	1.039153321	0.000707818	0.002653775
9030616G12Rik	34.62347187	-1.785577554	0.646456652	0.000726618	0.002716642
Gng7	57.90539219	-1.427920299	0.480646966	0.000734955	0.002744068
Gm4366	37.83177618	-1.002535872	0.321312861	0.000738303	0.002756035
Gm48768	18.94616329	-1.710349002	0.596259303	0.00073969	0.002760676
Slc26a4	3.810237697	-3.956801353	1.598967833	0.000750277	0.002796997
Slc28a1	8.223945145	-2.006716803	0.742425015	0.000750873	0.00279806
Ces3a	13722.8997	-1.866657972	0.692981161	0.000760984	0.002830241
Wfikkn2	11.76353479	-3.515640099	1.350645523	0.000761794	0.002831695
Prok1	35.54083115	-2.335695178	0.917028073	0.000765827	0.002844394
Gm5822	20.5369602	-1.298138805	0.4364409	0.000766129	0.002844966
Tubb4b-ps2	5.698631028	-3.251552536	1.241518568	0.000775005	0.00287459
Gm6192	43.18435558	-1.038793106	0.337962657	0.000783264	0.002901856
Gm7105	15.78611848	-1.492926087	0.521299088	0.000812152	0.002997308
Gm5560	16.55888807	-1.663092152	0.597806358	0.000815933	0.003008948
Gm37499	15.78804942	-1.792608342	0.638230652	0.000832474	0.003060538
Dnase1l3	34.83746134	-1.772770867	0.653318214	0.000838685	0.003079832
Lama4	1042.066869	-2.096922555	0.747226889	0.000841131	0.003087633
Fam228b	27.5443975	-1.38448318	0.478571774	0.000846609	0.003105958
Gm16418	6.003160582	-2.349737716	0.907460223	0.000853837	0.003126501
Rpsa-ps7	9.861355953	-1.844268544	0.68023298	0.00093056	0.003374598
Zfp-ps	3.088476683	-4.799431888	2.762921235	0.000934295	0.003385182
Pim1	357.6627216	-1.434213318	0.508745079	0.000945682	0.003422322
Tbx3os1	12.29936202	-2.323325966	0.938207277	0.000959881	0.003463912
Cd163	8.136828457	-3.380379305	1.434842357	0.000974923	0.003512908
Map9	133.7705127	-1.224027305	0.41660485	0.000976945	0.003519532

Gm10433	8.677071695	-2.068726496	0.7983805	0.000993984	0.003572868
Olfr1034	29.41337433	-1.170022072	0.399070766	0.00099949	0.003591986
Gm20632	31.8846595	-1.256973253	0.435533274	0.001029164	0.003690339
Gm6900	26.03911609	-1.000788994	0.332839315	0.001031298	0.003695917
Ighg2b	13.01932977	-3.451457513	1.471341494	0.001040294	0.003723292
Gm32511	9.0475539	-1.996481469	0.778989262	0.00104201	0.003728042
Gm45716	32.43482489	-1.164341335	0.398457713	0.001044573	0.003736516
Gm41231	9.056620413	-1.928523356	0.740072823	0.001064874	0.003799217
Gm6988	8.431371036	-1.953143053	0.742007243	0.001092165	0.003880716
Gm6580	5.633104267	-2.986605421	1.245699826	0.001105979	0.003925437
Pfn4	6.808110666	-2.235849194	0.86512369	0.001124423	0.003985742
Capn3	3.927502696	-3.02963013	1.208420909	0.001142085	0.004043119
Loxl4	268.2265955	-1.064176298	0.361536689	0.001145508	0.004052994
Hnf4g	40.52186957	-2.292820333	0.837526499	0.001149608	0.004065951
Ugt1a5	138.0769787	-1.139277713	0.39409326	0.0011538	0.004078573
Gm37691	12.28615943	-1.707054425	0.639754332	0.001163416	0.004108025
Tex12	6.91275814	-2.158427035	0.861398067	0.001193323	0.0042028
Clec12a	3.367056039	-4.140598807	2.502484559	0.001202824	0.004232379
Lrrc51	24.88111659	-1.262903326	0.444961875	0.001232387	0.004325301
Eif4ebp3	19.18865728	-1.530757542	0.568578414	0.001254519	0.004392532
Slc46a3	511.7793038	-1.314172522	0.474323354	0.001271668	0.004448129
Cib2	18.19448312	-1.477895107	0.543625787	0.001284979	0.004492629
3930402G23Rik	4.883040736	-2.545121067	1.03743184	0.001306806	0.004554008
Nhsl2	16.64455973	-3.782571472	2.329244599	0.001306638	0.004554008
Aplp1	281.9969343	-1.23169647	0.430729087	0.001309648	0.004562254
Chac1	57.75495605	-1.319063153	0.477206235	0.001323681	0.004602783
Ehd3	53.85588162	-1.614765733	0.618726289	0.001351646	0.004687284
Cox5b-ps	28.06023937	-1.396363482	0.514873484	0.001370671	0.004748971
Gm5540	17.50603181	-1.43222219	0.530043312	0.001392684	0.004816543
Rsad2	115.2819557	-1.196924286	0.428383913	0.001457159	0.00500794
Gm8798	9.005877727	-1.951978004	0.790304964	0.001499112	0.00513557
Gm49437	13.47180278	-1.436656878	0.537187882	0.001500914	0.005140824
Cct6b	13.51067082	-1.425402104	0.530493409	0.001504617	0.005150666
Gm21399	36.81898051	-1.067431121	0.375114315	0.00151962	0.005198399
Irf8	24.5113928	-1.323520116	0.48093156	0.001527359	0.005223945
Mup6	4.235855954	-4.068779532	2.0943264	0.001540333	0.005265503
9930111J21Rik2	16.82877235	-3.737023654	2.355577495	0.001543461	0.005275256
Insc	200.0343406	-1.125358373	0.400836921	0.001545487	0.005281239
Hsd17b6	1423.229486	-1.854853703	0.769142618	0.001599581	0.005447593
Ighg2c	8.914419762	-3.742191786	1.685594552	0.001611224	0.00547856
Gm7331	19.14797796	-1.282949803	0.47061012	0.001646556	0.005587808
Gm8451	21.85094826	-1.74591801	0.703167457	0.001660872	0.005630418
Hspa9-ps1	3.021863686	-3.548138904	1.642329658	0.001738934	0.005861916

Corin	8.00937149	-1.801321719	0.730621204	0.001760962	0.005933043
Gm37736	15.0241769	-1.786364535	0.738314698	0.001791644	0.006024783
2900052L18Rik	82.86557388	-1.403905696	0.539839198	0.001841898	0.006171067
Gm14270	6.726370858	-1.908721674	0.790583262	0.001915442	0.00639069
Gm21885	17.2224576	-1.499418782	0.589275037	0.001915824	0.006390854
Cd4	3.735848116	-3.910439528	2.009334121	0.001936862	0.006453179
Rpl17-ps5	10.01682001	-1.887983396	0.780541516	0.00194188	0.006468516
Chchd2-ps	8.305355305	-1.754967459	0.718232801	0.001968031	0.0065468
Gm5835	33.60748241	-1.11643606	0.410143415	0.002013045	0.006679175
Gm45424	7.034473134	-3.309308298	1.537756694	0.002039674	0.006764019
Rpsa-ps9	6.425089595	-1.997794458	0.843678786	0.002067337	0.00684039
Fras1	191.4035981	-1.003795259	0.362061989	0.002082518	0.006879945
Klf1	33.83578837	-1.171824047	0.436570434	0.002093501	0.006913848
Gm35315	34.9068849	-1.033827182	0.375078379	0.002132276	0.00702081
Gm10157	4.733434348	-2.216952241	0.954199446	0.002289336	0.007471879
Gm43254	23.12992486	-1.350407458	0.522993784	0.00233864	0.007617252
Gm50454	3.972542918	-2.669146439	1.160888127	0.002369868	0.007708498
Inmt	2946.144281	-1.522570748	0.627459924	0.002389785	0.007765384
Gm34333	20.04864502	-1.228811246	0.47271315	0.002410079	0.007823381
4932422M17Rik	30.09553161	-1.089988983	0.406236249	0.002435615	0.007896924
Gm4890	23.69931967	-1.292500606	0.500839187	0.002438624	0.00790533
Ldhb	209.098389	-1.144262676	0.427031879	0.002441002	0.007909045
0610040B10Rik	22.45368176	-1.328123086	0.52240009	0.002499718	0.008079115
Gm7392	7.007407282	-1.929022804	0.816187806	0.002518888	0.008128465
Cyp2c40	55.53062399	-2.730534091	1.551327485	0.00253277	0.008169141
E030037K01Rik	6.79876147	-2.060610331	0.922781694	0.002557638	0.008238273
Hadhb-ps	17.66734585	-1.152279656	0.442375113	0.002768598	0.008849463
Gm7429	14.25511815	-1.299400897	0.51424469	0.002780899	0.008881382
Trp53cor1	14.67722479	-1.768215257	0.770457473	0.002822316	0.008995694
Cacng7	4.724953302	-2.556241878	1.157244124	0.0028575	0.009103299
Adgre1	9.47130916	-2.804413028	1.476020359	0.002891506	0.009196369
Rpl28-ps3	22.25508918	-1.048829022	0.396303848	0.002897523	0.009213978
Gm12247	11.59624442	-1.402922778	0.574342187	0.002932333	0.009310787
Gm50063	5.946494593	-2.260240508	1.097431292	0.002955389	0.009379342
Serpina3c	5.155331749	-2.677669721	1.351910387	0.00297251	0.009428997
Gm6781	6.521443027	-2.453667497	1.187754243	0.002989335	0.009474538
9230111E07Rik	5.203356706	-3.011922136	1.359700768	0.003045996	0.009633438
Shisa7	17.35976496	-2.884892021	1.374200666	0.003094442	0.009773772
Eif3j2	24.70260858	-1.014730518	0.385102447	0.003140284	0.009902264
Spr-ps1	6.818730786	-3.023307882	1.521142115	0.003142794	0.009908553
Gm42427	4.708310653	-2.266180672	1.027126883	0.003161326	0.009962072
Gm30117	7.717643251	-2.061667634	0.989062833	0.003163683	0.009966224
Ces2b	2.260503465	-4.347746574	3.218499323	0.003175369	0.009999755

Gm5425	7.871936726	-1.789490052	0.803537131	0.003176451	0.010001394
Keg1	1201.063923	-1.740804992	0.80087723	0.003184422	0.010021687
Gm13855	3.685190896	-3.524046037	2.06736647	0.003268128	0.010258202
Cd37	3.568173229	-3.63470317	2.631677515	0.00327864	0.010279428
Ptprc	10.3577448	-2.06003739	0.986378792	0.003284656	0.010296609
Xlr3a	101.2307206	-1.460411072	0.623398924	0.003302315	0.010341828
Gm31717	5.065401613	-2.512936446	1.330247792	0.003418805	0.010663138
Oaz1-ps	43.71968148	-1.099813277	0.430744479	0.003418374	0.010663138
Rpl7a-ps3	1.971937308	-4.059065665	3.073897183	0.003516278	0.010931619
Gm30931	4.827639772	-2.158568353	1.081098435	0.003542418	0.011002192
1700029J07Rik	59.74442814	-1.431870721	0.594004776	0.003550191	0.011020983
Cyp2c69	13.83888871	-2.438875656	1.381411324	0.003567198	0.011068407
BC048644	39.21743241	-1.012784954	0.395032794	0.003709976	0.011466926
Itgb2	7.431826891	-2.437599879	1.264569274	0.003710777	0.011467556
C1qc	23.46457007	-1.835908278	0.874712289	0.003740841	0.011543734
Espnl	3.576752508	-3.719231718	2.82493902	0.003802944	0.011704348
D030034A15Rik	2.653942049	-4.303422269	3.487013265	0.003883081	0.011907992
Gm4013	25.85581498	-1.066255255	0.423457708	0.003966086	0.01213926
Afap1l1	19.95070606	-1.806884635	0.824253125	0.003990656	0.012198897
Gm4742	2.862441683	-2.94609766	1.596022305	0.004115779	0.01253148
Vmn2r20	3.06884068	-3.3481993	2.26687804	0.004277864	0.01297353
Glns-ps1	4.853892041	-2.394906479	1.291388259	0.004311179	0.013053934
Ccn4	27.00820762	-1.353940946	0.574540765	0.0043182	0.013069457
Gm12350	15.32678218	-1.46010015	0.652277676	0.004352624	0.013162813
Gm49602	34.96009954	-1.009266035	0.401370867	0.004524566	0.013629091
Ighv1-80	2.438449771	-3.759561193	3.117619555	0.004564889	0.013741928
Coro2a	44.55569317	-1.196720022	0.495750084	0.004585267	0.013798944
Cyp4f39	6.342779342	-2.062213731	1.100109818	0.004587091	0.013802266
Avil	7.388813982	-2.765799181	1.473026923	0.004636494	0.013934813
Gm6055	7.417165273	-1.56314027	0.711761483	0.004696198	0.014088594
Marchf1	5.872799975	-2.73025959	1.320068618	0.004706481	0.014115029
Saa4	1985.288536	-1.353903913	0.603644119	0.004769979	0.014276462
Obox4-ps1	1.945293539	-3.927963141	3.554741328	0.004882077	0.014566534
Ndufab1-ps	7.053353695	-1.493285632	0.683316154	0.004936955	0.01470081
Zfp36l1-ps	5.994162274	-2.23118269	1.201338607	0.005010131	0.014897869
Slc16a13	72.38522849	-1.568245661	0.696439564	0.005035517	0.014966177
Gm2962	6.568687773	-1.744327203	0.854810518	0.005043221	0.014986755
Ikzf4	63.63816012	-1.422966933	0.620883631	0.005069582	0.015048778
Tubb4b-ps1	14.83640429	-1.485839655	0.691568322	0.00511097	0.015150545
Lrrc19	2.74631201	-3.25137289	2.683548526	0.00511803	0.01516913
Gm7208	4.422769583	-2.530551029	1.549944302	0.005147151	0.015241316
Gm5644	6.207225769	-1.811923299	0.902468658	0.005188163	0.015346182
Gm13651	2.377901949	-3.686775087	3.235149348	0.005224718	0.015440031

Sh3tc2	32.72765566	-2.874259975	2.345260413	0.005228015	0.015447396
Gm12435	10.22639307	-1.282108567	0.558513396	0.005247343	0.015497347
Rpl9-ps7	12.35370604	-1.279261262	0.559656664	0.005268116	0.015546735
Gm11769	2.83371162	-3.217778553	2.694793688	0.005327577	0.015690843
Gm10320	6.053480417	-1.756453791	0.872449869	0.005341902	0.015727129
Gm17040	7.108690824	-1.955968549	1.067212161	0.005368978	0.015800652
Gchfr	532.5179654	-1.345277363	0.613748493	0.005406618	0.015901672
Gm10069	14.94003861	-1.065917235	0.44257198	0.005413404	0.01591595
Gm48488	3.900775578	-2.639493033	1.320758891	0.005482645	0.016095677
Acsl6	4.668498167	-3.212765652	2.719918578	0.005507894	0.016164857
Gm4853	2.999611076	-3.097824695	2.315073172	0.005598079	0.016404448
Gm13835	16.81999965	-1.045115915	0.435160007	0.00564747	0.016534035
Gm5461	2.230800024	-3.32402582	2.900418218	0.005667206	0.016579168
Gm7652	22.64085239	-1.451594286	0.687603646	0.005697771	0.01665589
Plxnc1	21.54129924	-1.377727429	0.634440819	0.005791168	0.016908306
Gm6807	12.36664678	-1.245697199	0.545835919	0.005819311	0.016985308
Cyp46a1	26.66361283	-1.191866531	0.521397061	0.005856441	0.017063462
Myom3	44.89391743	-1.377558556	0.643770467	0.005969948	0.017343213
Gm43444	4.159091746	-2.487476712	1.47015164	0.006247847	0.018060316
Rpl36-ps12	30.03118728	-1.048721307	0.445404137	0.006265338	0.01809997
Gm14288	2.819391318	-3.115558325	2.790313309	0.006291615	0.018166113
Gm7476	3.22583764	-2.566670331	1.352520746	0.006296569	0.018176512
Gm10136	13.86804738	-1.177101342	0.515669123	0.006304952	0.018197975
Commd5	3.689674459	-2.610428202	1.565190732	0.006317616	0.018231785
Gm12185	3.546828791	-2.944565047	2.610382586	0.006452551	0.018584858
Gata4	105.6331586	-1.036589928	0.443866627	0.006522546	0.018755496
Fcna	20.62619477	-1.901554221	1.134498756	0.006852463	0.01955474
Spink5	9.389982952	-1.380879845	0.664143667	0.006941006	0.019766263
Muc1	1063.430292	-2.735687501	2.449547819	0.00707682	0.020102316
Gm42517	20.17534814	-2.716844298	2.473190214	0.00708733	0.020119012
Gm7079	3.762950839	-2.622933969	1.517095279	0.007103173	0.020156291
Gm46411	8.397250568	-1.729859003	0.96067598	0.007304806	0.020673431
Gm11992	38.21948997	-1.097830154	0.483450989	0.007488903	0.021144555
Gm14044	4.155798067	-2.041548829	1.137167816	0.007532946	0.0212564
4933431K14Rik	14.56607374	-1.15898849	0.522028201	0.007804153	0.021919948
Gm28720	7.079678192	-1.698597177	0.941404746	0.008138223	0.022743648
Gm7461	3.047577085	-2.765585699	2.748370796	0.008160225	0.022795179
Gm15634	2.553534082	-2.657891246	2.437676132	0.008261409	0.023030897
Cdnf	7.970934097	-1.613342336	0.863041218	0.008292088	0.023092942
Psma5-ps	3.940272259	-1.924219775	1.064129047	0.008345969	0.023226144
Gm12070	6.939441509	-1.446203228	0.732895775	0.008390715	0.023323611
C1qtnf4	23.25793332	-1.321906726	0.632268833	0.00842137	0.023402042
Gm12989	6.330060436	-1.854522551	1.09732285	0.008455543	0.023480006

C2cd4d	17.81360803	-1.018596592	0.45210683	0.008505362	0.023584222
Gm15962	7.877273386	-1.429084551	0.734241908	0.008504175	0.023584222
Gm45301	5.787820002	-1.672017314	0.956360416	0.008537883	0.023650478
Gm14176	5.735929577	-1.727093724	0.918554545	0.008569794	0.023725175
Gm13420	41.95592989	-1.637379753	0.841149981	0.008574046	0.023733523
Ctcfl	14.99400641	-1.189236141	0.565274836	0.008697412	0.024026491
Gngt1	6.901801064	-1.565584957	0.857091863	0.008798598	0.024257718
Scd3	8.515486382	-1.825295438	0.974498317	0.008806882	0.024272996
Gm13904	4.396824531	-2.149562694	1.320930951	0.008880188	0.024446949
Nfasc	10.49046021	-1.683725059	0.981551501	0.008881958	0.024448316
Gm6392	4.641477395	-1.964264058	1.221940134	0.008929669	0.024569072
Car5a	454.7033229	-1.418462335	0.752942623	0.009023085	0.024790554
Gm6740	5.426895596	-1.905494967	1.23189883	0.009024593	0.024791148
Rasl2-9	6.951730154	-1.805532186	1.117067777	0.009102993	0.024979284
Nwd2	3.654983315	-1.799151565	1.049040682	0.009182002	0.025158738
Gm2387	10.31620413	-1.354919856	0.684575461	0.009223311	0.025261103
Gm44744	10.9894217	-1.372129366	0.70344896	0.009238987	0.02529321
Pspn	3.248545738	-2.438940794	1.717415052	0.009253795	0.025315692
Gm35665	6.216863856	-1.605866411	0.919781698	0.009328803	0.025499083
Fermt3	16.31082138	-1.024438798	0.462822363	0.009414049	0.025706625
Gm10039	8.10734532	-1.337591282	0.674834022	0.009443521	0.025775937
1700023B13Rik	10.79830285	-1.500948471	0.817323973	0.009546098	0.026020231
Gm16731	5.702263737	-1.943645126	1.366042112	0.00958707	0.026112044
Gm5171	3.325419679	-2.410680787	1.730142407	0.009663938	0.026291556
Rpl17-ps4	19.31345191	-1.082892894	0.502519824	0.009694736	0.026360397
Gm20768	2.838797686	-2.443904323	1.857134504	0.009742854	0.026472479
Gm22146	27.49731258	-1.196497051	0.584409171	0.00980921	0.02662262
C330013E15Rik	22.9871098	-1.211068284	0.591534276	0.009895124	0.026817869
Nat8f6	5.004872258	-2.128443415	1.952976287	0.009934702	0.026921331
Ccng2	454.845879	-1.151639871	0.553301744	0.009947723	0.02695008
Gm12216	23.91178644	-1.050136516	0.481426803	0.009958829	0.026970796
Cd300lg	7.296732644	-1.562094121	0.88288769	0.010072958	0.027242128
Gm7658	4.624602457	-1.918988835	1.149196717	0.010125466	0.027376419
Gm10819	6.294443844	-1.505918074	0.819812581	0.010476097	0.028196791
Cyp2b9	126.104431	-1.873082387	1.830678015	0.010488144	0.028217856
Prdx2-ps1	6.342012233	-1.547466876	0.846782102	0.010495091	0.028224677
Gdf11	18.07229697	-2.144814462	1.138896904	0.010522001	0.028285155
Psma8	10.80323587	-1.274610249	0.645554207	0.010531891	0.02829985
Ly6a	120.8567282	-1.069114527	0.501256849	0.010579375	0.028407553
Nat1	13.29458659	-1.273183393	0.653406243	0.010895498	0.029158485
Gm13394	5.195303177	-1.840497723	1.274787202	0.010901437	0.029170311
Gm10053	7.686813391	-1.556024584	0.901268701	0.010920096	0.029208024
Gm6733	7.764481761	-1.407421543	0.749069164	0.010968893	0.029322194

Gm26616	13.2352968	-1.044627639	0.493584715	0.011203222	0.029882007
Pcolce2	3.594650114	-2.305210742	1.785003797	0.011269639	0.030034113
Fut4	46.73384781	-2.159844182	1.415114919	0.011329811	0.030173523
A530083I20Rik	2.965048011	-2.313158503	1.943947039	0.011489164	0.030551275
Spata33	23.5128086	-1.648254303	0.893829995	0.011574863	0.030736571
Gm5457	3.216130893	-1.823191389	1.203114361	0.011673421	0.030972575
Tspan2	48.63978492	-2.143502934	1.426640369	0.011712255	0.031071314
P2ry4	85.58427689	-1.740150626	1.363347844	0.01175469	0.031166657
Gm45609	4.394684989	-1.83836684	1.294944107	0.01178365	0.031234811
Cd180	2.49383644	-2.260288876	4.224480198	0.011821665	0.031318273
Rpl19-ps11	103.5214918	-1.123698491	0.558124237	0.011964626	0.031666409
Gm9575	5.83073288	-1.571565413	0.923432727	0.011969107	0.031673902
Atad3aos	8.491836848	-1.443655895	0.800801228	0.012085865	0.031921254
Gm19935	64.47491711	-2.11379414	2.384780707	0.012220293	0.032236377
Ndufa4l2	11.04792971	-1.17895605	0.601423136	0.01222883	0.032250025
Gm12254	14.68593975	-1.308669651	0.704419704	0.012297987	0.032374578
Mboat1	387.7333064	-1.748089228	0.940241344	0.012348045	0.032488529
Gm43429	13.58241762	-1.089426433	0.534092946	0.012386921	0.032577415
Fabp1	5665.047044	-1.287708626	0.702901587	0.012507572	0.032854204
Cyp3a59	243.0040363	-1.243111817	0.664501371	0.012595888	0.03302742
9530080O11Rik	7.685260137	-1.438876393	0.830109532	0.012695508	0.033256823
Slc4a8	23.06545903	-2.052949786	2.404920863	0.012913553	0.033758887
Hsd3b2	424.6461296	-1.242324088	0.670657825	0.012986938	0.033918389
Fam167a	311.7179687	-2.006513451	1.093846234	0.013124942	0.03422293
Gm47602	10.20789157	-1.613415464	0.909890404	0.013249588	0.03448235
9330121J05Rik	6.751324277	-1.388568147	0.796267457	0.013292434	0.034577153
Oas3	40.17653559	-2.005994138	2.425023921	0.013896965	0.035938102
Flicr	3.600762049	-2.056056024	1.633656901	0.014001887	0.036177954
Rps15-ps2	7.657203354	-1.336013039	0.745101382	0.014045433	0.036278049
Tmem132e	2.479152124	-1.704278994	2.976719441	0.01411146	0.036429704
Car3	11777.77454	-1.555610848	1.127932769	0.014218107	0.036664833
Gm2058	4.451247136	-1.812883206	1.191847165	0.014517382	0.037326242
Gm42777	4.484898925	-1.760378149	1.257562145	0.014577684	0.037466229
Rab40b	11.48975692	-1.290039121	0.712262111	0.014653121	0.037624839
Gm11335	5.503070163	-2.001103904	1.653577163	0.01519802	0.038842084
Gm12250	15.48408481	-1.111556295	0.576988052	0.015203436	0.03885075
Gm19196	9.10640304	-1.447529648	0.834991701	0.015383341	0.039221696
Gm20900	10.69258239	-1.307326069	0.753175446	0.015469519	0.039394312
P2ry6	20.07441305	-1.516208168	0.903102505	0.015825602	0.040146613
Vsig4	19.12971314	-1.5791401	1.186981345	0.015830737	0.040154332
Cd300ld	3.800140866	-2.0936462	1.848280279	0.015926358	0.040354205
Gm15608	14.61158076	-1.175865646	0.634144979	0.015942787	0.040385166
Tagap	7.892845261	-1.84488439	2.428911784	0.01595813	0.040413365

Cyp2d38-ps	135.1252822	-1.291624794	0.773150057	0.016107205	0.040774749
Cdh5	22.75661424	-1.302389371	0.769731423	0.016193545	0.040960896
E230029C05Rik	3.428990898	-1.978125744	1.700082752	0.016228595	0.041027923
Got1l1	3.303518296	-1.789192602	2.058014964	0.016347429	0.041290274
Slc30a2	2.699828127	-1.819052915	2.799433108	0.016386976	0.041357503
Serpina9	4.99340209	-1.657450477	1.971140062	0.01645809	0.041531519
Cdkl1	3.725095484	-1.709062636	3.142923791	0.01656009	0.041734037
Ksr2	16.95337411	-1.814715824	2.420250454	0.016591414	0.041801924
Gm5512	2.63531813	-2.002437391	1.565510832	0.016613612	0.041841447
Gm10232	6.863455904	-1.299578504	0.752566252	0.016707676	0.042056271
B230206L02Rik	14.32575145	-1.085452216	0.571703824	0.01683118	0.042317198
5830444B04Rik	12.4705625	-1.555667466	1.123591208	0.016866154	0.04238847
Igkv6-15	2.966492718	-1.731753522	2.738995294	0.017073973	0.042871465
Gm4887	2.07898295	-1.917803921	1.881456709	0.017176805	0.043107108
Gm37419	3.488445271	-1.73939124	1.167191132	0.017315886	0.043397599
Cyfip2	219.6450768	-1.650337786	1.091078561	0.017322878	0.043399914
Smarce1-ps1	5.030634679	-1.616024416	1.089931415	0.017885121	0.044587342
Gm10644	16.49254918	-1.219396231	0.711528742	0.018104048	0.045057033
Chrm1	8.474492602	-1.883989402	1.764450089	0.018139754	0.045139595
Gm29863	2.282948114	-1.501087434	2.379640567	0.018376485	0.045634463
Wfdc17	8.095122121	-1.424667013	0.950495744	0.018432743	0.045750487
Kiss1r	10.92723229	-1.194624793	0.680464421	0.018615171	0.046143599
Gm17080	21.7268911	-1.095883609	0.605368654	0.019032097	0.047001025
Gm13268	6.858668112	-1.347325342	0.845020115	0.019165692	0.047270119
Gm17802	4.312534003	-1.47637756	1.008268694	0.019280326	0.04750401
Gm8518	3.648777173	-1.679787068	1.200874432	0.019543323	0.048077926
Gm47761	17.34881042	-1.338343661	0.88332782	0.019622117	0.048222315
Folr2	2.972787477	-1.896693923	1.423466781	0.01963256	0.048240794
Gm3362	4.03335458	-1.780287463	1.599512079	0.01972955	0.048431425
Asb14	7.824817107	-1.396581384	0.925671159	0.020250085	0.04944896
