# Supplementary material for: Physiological reprogramming in vivo mediated by Sox4 pioneer factor activity
Source: bioRxiv. 2023 Feb 14:2023.02.14.528556. Preprint. [Version 1] doi: 10.1101/2023.02.14.528556 (PMC9948957; doi:10.1101/2023.02.14.528556)
Supplement: Supplement 4 [file media-4.pdf]

Table S2. List of known hepatocyte and biliary/reprogrammed cell genes.

Hepatocyte genes (source)		Biliary/Reprogrammed cell genes (source)	
Alb	(Tarlow et al., 2014)	*Ccl2*	(Tarlow *et al.*, 2014)
Cyp7a1	(Tarlow *et al.*, 2014)	*Zeb1*	(Tarlow *et al.*, 2014)
F9	(Tarlow *et al.*, 2014)	*Mst1r*	(Tarlow *et al.*, 2014)
Fah	(Tarlow *et al.*, 2014)	*Bmp4*	(Tarlow *et al.*, 2014)
Hgd	(Tarlow *et al.*, 2014)	*Vim*	(Tarlow *et al.*, 2014)
Hnf4a	(Tarlow *et al.*, 2014)	*Bmp1*	(Tarlow *et al.*, 2014)
Tdo2	(Tarlow *et al.*, 2014)	*Foxp2*	(Tarlow *et al.*, 2014)
Cps1	(Schaub et al., 2018)	*Sfrp4*	(Tarlow *et al.*, 2014)
Cyp26a1	(Schaub *et al.*, 2018)	*Etv4*	(Tarlow *et al.*, 2014)
Cyp27a1	(Schaub *et al.*, 2018)	*Dab1*	(Tarlow *et al.*, 2014)
Cyp2c38	(Schaub *et al.*, 2018)	*Kit*	(Tarlow *et al.*, 2014)
Cyp2c67	(Schaub *et al.*, 2018)	*Nes*	(Tarlow *et al.*, 2014)
Cyp2d10	(Schaub *et al.*, 2018)	*Ncam1*	(Tarlow *et al.*, 2014)
Cyp2d11	(Schaub *et al.*, 2018)	*Tbx1*	(Tarlow *et al.*, 2014)
Cyp2d12	(Schaub *et al.*, 2018)	*Mcam*	(Tarlow *et al.*, 2014)
Cyp2d13	(Schaub *et al.*, 2018)	*Tgfb1*	(Tarlow *et al.*, 2014)
Cyp2d40	(Schaub *et al.*, 2018)	*Fzd10*	(Tarlow *et al.*, 2014)
Cyp2d9	(Schaub *et al.*, 2018)	*Cav1*	(Tarlow *et al.*, 2014)
Cyp2j5	(Schaub *et al.*, 2018)	*Yap1*	(Tarlow *et al.*, 2014)
Cyp2r1	(Schaub *et al.*, 2018)	*Cd44*	(Tarlow *et al.*, 2014)
Cyp3a11	(Schaub *et al.*, 2018)	*Jag1*	(Tarlow *et al.*, 2014)
Cyp4f13	(Schaub *et al.*, 2018)	*Hnf1b*	(Tarlow *et al.*, 2014)
Cyp4v3	(Schaub *et al.*, 2018)	*Spp1*	(Tarlow *et al.*, 2014)
Cyp8b1	(Schaub *et al.*, 2018)	*Smo*	(Tarlow *et al.*, 2014)
Foxa3	(Schaub *et al.*, 2018)	*Itga3*	(Tarlow *et al.*, 2014)
Hnf1a	(Schaub *et al.*, 2018)	*Notch2*	(Tarlow *et al.*, 2014)
Mup1	(Schaub *et al.*, 2018)	*Foxj1*	(Tarlow *et al.*, 2014)
Otc	(Schaub *et al.*, 2018)	*Sox9*	(Tarlow *et al.*, 2014)
Tat	(Schaub *et al.*, 2018)	*Ccn1*	(Tarlow *et al.*, 2014)
Trf	(Schaub *et al.*, 2018)	*Tnfrsf12a*	(Tarlow *et al.*, 2014)
Ttr	(Schaub *et al.*, 2018)	*Krt19*	(Tarlow *et al.*, 2014)
Abcb11	(Katsuda et al., 2020)	*Grhl2*	(Tarlow *et al.*, 2014)
Ahr	(Katsuda *et al.*, 2020)	*Cftr*	(Tarlow *et al.*, 2014)
Aldh1a1	(Katsuda *et al.*, 2020)	*Epcam*	(Tarlow *et al.*, 2014)
Ass1	(Katsuda *et al.*, 2020)	*Krt7*	(Tarlow *et al.*, 2014)
Baat	(Katsuda *et al.*, 2020)	*Tacstd2*	(Tarlow *et al.*, 2014)
Cyp1a2	(Katsuda *et al.*, 2020)	*Ccn2*	(Tarlow *et al.*, 2014)
Cyp2e1	(Katsuda *et al.*, 2020)	*Krt17*	(Schaub *et al.*, 2018)
G6pc	(Katsuda *et al.*, 2020)	*Prom1*	(Schaub *et al.*, 2018)
Gsta1	(Katsuda *et al.*, 2020)	*Sstr2*	(Schaub *et al.*, 2018)
Gys2	(Katsuda *et al.*, 2020)	*St14*	(Schaub *et al.*, 2018)
Nr1i2	(Katsuda *et al.*, 2020)	*Cldn4*	(Schaub *et al.*, 2018)

Nr1i3	(Katsuda *et al.*, 2020)	*Muc1*	(Schaub *et al.*, 2018)
Pck1	(Katsuda *et al.*, 2020)	*Sox4*	(Poncy et al., 2015)
Slc10a1	(Katsuda *et al.*, 2020)	*Ezr*	(Merrell et al., 2021)
Serpina7	(Katsuda *et al.*, 2020)	*Cd24a*	(Merrell et al., 2021)
Cebpa	(Jakobsen et al., 2013)	*Igfbp7*	(Merrell et al., 2021)
Cebpb	(Jakobsen *et al.*, 2013)	*Itga6*	(Yanger et al., 2013)
Asgr1	(Peters et al., 2016)	*Cadm1*	(Ito et al., 2007)
Fabp1	(Huang et al., 2016)		

References for Table S2

Huang, H., McIntosh, A.L., Martin, G.G., Landrock, D., Chung, S., Landrock, K.K., Dangott, L.J., Li, S., Kier, A.B., and Schroeder, F. (2016). FABP1: A Novel Hepatic Endocannabinoid and Cannabinoid Binding Protein. Biochemistry *55*, 5243-5255. 10.1021/acs.biochem.6b00446.

Ito, A., Nishikawa, Y., Ohnuma, K., Ohnuma, I., Koma, Y., Sato, A., Enomoto, K., Tsujimura, T., and Yokozaki, H. (2007). SgIGSF is a novel biliary-epithelial cell adhesion molecule mediating duct/ductule development. Hepatology *45*, 684-694. 10.1002/hep.21501.

Jakobsen, J.S., Waage, J., Rapin, N., Bisgaard, H.C., Larsen, F.S., and Porse, B.T. (2013). Temporal mapping of CEBPA and CEBPB binding during liver regeneration reveals dynamic occupancy and specific regulatory codes for homeostatic and cell cycle gene batteries. Genome Res *23*, 592-603. 10.1101/gr.146399.112.

Katsuda, T., Hosaka, K., Matsuzaki, J., Usuba, W., Prieto-Vila, M., Yamaguchi, T., Tsuchiya, A., Terai, S., and Ochiya, T. (2020). Transcriptomic Dissection of Hepatocyte Heterogeneity: Linking Ploidy, Zonation, and Stem/Progenitor Cell Characteristics. Cell Mol Gastroenterol Hepatol *9*, 161-183. 10.1016/j.jcmgh.2019.08.011.

Merrell, A.J., Peng, T., Li, J., Sun, K., Li, B., Katsuda, T., Grompe, M., Tan, K., and Stanger, B.Z. (2021). Dynamic Transcriptional and Epigenetic Changes Drive Cellular Plasticity in the Liver. Hepatology *74*, 444-457. 10.1002/hep.31704.

Peters, D.T., Henderson, C.A., Warren, C.R., Friesen, M., Xia, F., Becker, C.E., Musunuru, K., and Cowan, C.A. (2016). Asialoglycoprotein receptor 1 is a specific cell-surface marker for isolating hepatocytes derived from human pluripotent stem cells. Development *143*, 1475-1481. 10.1242/dev.132209.

Poncy, A., Antoniou, A., Cordi, S., Pierreux, C.E., Jacquemin, P., and Lemaigre, F.P. (2015). Transcription factors SOX4 and SOX9 cooperatively control development of bile ducts. Dev Biol *404*, 136-148. 10.1016/j.ydbio.2015.05.012.

Schaub, J.R., Huppert, K.A., Kurial, S.N.T., Hsu, B.Y., Cast, A.E., Donnelly, B., Karns, R.A., Chen, F., Rezvani, M., Luu, H.Y., et al. (2018). De novo formation of the biliary system by

TGFbeta-mediated hepatocyte transdifferentiation. Nature *557*, 247-251. 10.1038/s41586-018-0075-5.

Tarlow, B.D., Pelz, C., Naugler, W.E., Wakefield, L., Wilson, E.M., Finegold, M.J., and Grompe, M. (2014). Bipotential adult liver progenitors are derived from chronically injured mature hepatocytes. Cell Stem Cell *15*, 605-618. 10.1016/j.stem.2014.09.008.

Yanger, K., Zong, Y., Maggs, L.R., Shapira, S.N., Maddipati, R., Aiello, N.M., Thung, S.N., Wells, R.G., Greenbaum, L.E., and Stanger, B.Z. (2013). Robust cellular reprogramming occurs spontaneously during liver regeneration. Genes Dev *27*, 719-724. 10.1101/gad.207803.112.
