# Supplementary material for: Physiological reprogramming in vivo mediated by Sox4 pioneer factor activity
Source: bioRxiv. 2023 Feb 14:2023.02.14.528556. Preprint. [Version 1] doi: 10.1101/2023.02.14.528556 (PMC9948957; doi:10.1101/2023.02.14.528556)
Supplement: Supplement 5 [file media-5.pdf]

Table S3. GO terms enriched in differentially opened regions in Sox4-expressing hepatocytes compared to empty virus-injected hepatocytes, and GO terms enriched for differentially expressed genes between hepatocytes and reprogrammed cells.

GO terms associated with 6742 genes near newly closed regions

ID	Description	GeneRatio	pvalue	p.adjust
GO:0006631	fatty acid metabolic process	210/5895	2.69E-30	1.70E-26
GO:0008202	steroid metabolic process	162/5895	8.88E-25	2.80E-21
GO:0044282	small molecule catabolic process	165/5895	3.49E-24	7.34E-21
GO:1901615	organic hydroxy compound metabolic process	228/5895	1.07E-23	1.68E-20
GO:0016054	organic acid catabolic process	124/5895	3.60E-22	3.79E-19
GO:0046395	carboxylic acid catabolic process	124/5895	3.60E-22	3.79E-19
GO:0019216	regulation of lipid metabolic process	176/5895	7.25E-22	6.53E-19
GO:0046394	carboxylic acid biosynthetic process	152/5895	1.35E-21	1.07E-18
GO:0016053	organic acid biosynthetic process	152/5895	2.07E-21	1.45E-18
GO:0062012	regulation of small molecule metabolic process	173/5895	2.54E-21	1.60E-18
GO:0006520	cellular amino acid metabolic process	131/5895	2.82E-20	1.62E-17
GO:0006639	acylglycerol metabolic process	86/5895	3.66E-20	1.92E-17
GO:1901605	alpha-amino acid metabolic process	103/5895	6.82E-20	3.31E-17
GO:0006638	neutral lipid metabolic process	86/5895	1.41E-19	6.36E-17

GO:0006790	sulfur compound metabolic process	140/5895	2.40E-19	1.01E-16
GO:0016042	lipid catabolic process	156/5895	3.60E-19	1.42E-16
GO:0072521	purine-containing compound metabolic process	179/5895	3.52E-18	1.31E-15
GO:0006641	triglyceride metabolic process	70/5895	4.98E-18	1.74E-15
GO:0009117	nucleotide metabolic process	188/5895	1.50E-17	4.98E-15
GO:0006753	nucleoside phosphate metabolic process	190/5895	3.70E-17	1.17E-14
GO:0006066	alcohol metabolic process	150/5895	1.02E-16	3.01E-14
GO:1902652	secondary alcohol metabolic process	82/5895	1.05E-16	3.01E-14
GO:0006109	regulation of carbohydrate metabolic process	103/5895	1.46E-16	3.88E-14
GO:0033865	nucleoside bisphosphate metabolic process	64/5895	1.60E-16	3.88E-14
GO:0033875	ribonucleoside bisphosphate metabolic process	64/5895	1.60E-16	3.88E-14
GO:0034032	purine nucleoside bisphosphate metabolic process	64/5895	1.60E-16	3.88E-14
GO:0015711	organic anion transport	197/5895	2.04E-16	4.54E-14
GO:0031589	cell-substrate adhesion	159/5895	2.09E-16	4.54E-14
GO:0008203	cholesterol metabolic process	77/5895	2.14E-16	4.54E-14
GO:0031669	cellular response to nutrient levels	100/5895	2.16E-16	4.54E-14
GO:0010876	lipid localization	180/5895	2.47E-16	5.03E-14
GO:0044262	cellular carbohydrate metabolic process	138/5895	2.84E-16	5.60E-14

GO:1901652	response to peptide	175/5895	4.13E-16	7.90E-14
GO:0019693	ribose phosphate metabolic process	156/5895	4.88E-16	8.91E-14
GO:0042493	response to drug	126/5895	4.94E-16	8.91E-14
GO:0046486	glycerolipid metabolic process	161/5895	5.68E-16	9.91E-14
GO:0009259	ribonucleotide metabolic process	152/5895	5.81E-16	9.91E-14
GO:0009150	purine ribonucleotide metabolic process	148/5895	9.48E-16	1.57E-13
GO:0055088	lipid homeostasis	85/5895	1.27E-15	2.06E-13
GO:0016125	sterol metabolic process	79/5895	2.28E-15	3.59E-13
GO:0045834	positive regulation of lipid metabolic process	90/5895	2.34E-15	3.60E-13
GO:0072330	monocarboxylic acid biosynthetic process	101/5895	2.48E-15	3.65E-13
GO:0030111	regulation of Wnt signaling pathway	140/5895	2.49E-15	3.65E-13
GO:0006163	purine nucleotide metabolic process	157/5895	3.20E-15	4.59E-13
GO:0006637	acyl-CoA metabolic process	55/5895	4.29E-15	5.89E-13
GO:0035383	thioester metabolic process	55/5895	4.29E-15	5.89E-13
GO:0009896	positive regulation of catabolic process	193/5895	4.86E-15	6.52E-13
GO:0090407	organophosphate biosynthetic process	181/5895	5.24E-15	6.88E-13
GO:0005996	monosaccharide metabolic process	124/5895	5.56E-15	7.16E-13
GO:0031331	positive regulation of cellular catabolic process	170/5895	8.93E-15	1.11E-12

GO:0010675	regulation of cellular carbohydrate metabolic process	87/5895	8.94E-15	1.11E-12
GO:0044242	cellular lipid catabolic process	108/5895	1.35E-14	1.64E-12
GO:0042180	cellular ketone metabolic process	105/5895	1.46E-14	1.73E-12
GO:0007264	small GTPase mediated signal transduction	183/5895	1.48E-14	1.73E-12
GO:0031668	cellular response to extracellular stimulus	107/5895	1.81E-14	2.07E-12
GO:0006694	steroid biosynthetic process	86/5895	1.92E-14	2.17E-12
GO:0009267	cellular response to starvation	84/5895	2.11E-14	2.33E-12
GO:0033500	carbohydrate homeostasis	127/5895	2.68E-14	2.92E-12
GO:0006914	autophagy	178/5895	3.34E-14	3.51E-12
GO:0061919	process utilizing autophagic mechanism	178/5895	3.34E-14	3.51E-12
GO:0048732	gland development	193/5895	3.55E-14	3.68E-12
GO:0050673	epithelial cell proliferation	182/5895	4.15E-14	4.22E-12
GO:0042593	glucose homeostasis	126/5895	4.99E-14	5.00E-12
GO:0007623	circadian rhythm	106/5895	5.27E-14	5.19E-12
GO:0016055	Wnt signaling pathway	179/5895	5.52E-14	5.36E-12
GO:0060537	muscle tissue development	192/5895	5.86E-14	5.61E-12
GO:0010906	regulation of glucose metabolic process	72/5895	7.98E-14	7.52E-12
GO:0198738	cell-cell signaling by wnt	179/5895	9.14E-14	8.48E-12

GO:0043434	response to peptide hormone	147/5895	9.29E-14	8.49E-12
GO:0007517	muscle organ development	175/5895	9.55E-14	8.61E-12
GO:0009063	cellular amino acid catabolic process	56/5895	1.37E-13	1.22E-11
GO:0045666	positive regulation of neuron differentiation	191/5895	1.96E-13	1.72E-11
GO:0006869	lipid transport	155/5895	3.01E-13	2.60E-11
GO:1901653	cellular response to peptide	133/5895	3.14E-13	2.67E-11
GO:0042594	response to starvation	90/5895	3.36E-13	2.83E-11
GO:0072329	monocarboxylic acid catabolic process	68/5895	4.13E-13	3.42E-11
GO:0046890	regulation of lipid biosynthetic process	92/5895	4.28E-13	3.51E-11
GO:0071496	cellular response to external stimulus	126/5895	4.79E-13	3.87E-11
GO:0048511	rhythmic process	133/5895	5.72E-13	4.57E-11
GO:0006006	glucose metabolic process	98/5895	7.87E-13	6.21E-11
GO:0071375	cellular response to peptide hormone stimulus	116/5895	8.62E-13	6.71E-11
GO:0007265	Ras protein signal transduction	149/5895	8.84E-13	6.80E-11
GO:0062013	positive regulation of small molecule metabolic process	81/5895	1.27E-12	9.66E-11
GO:0001889	liver development	69/5895	1.49E-12	1.12E-10
GO:0014706	striated muscle tissue development	180/5895	1.61E-12	1.19E-10
GO:0042176	regulation of protein catabolic process	163/5895	1.74E-12	1.27E-10

GO:0051056	regulation of small GTPase mediated signal transduction	121/5895	2.65E-12	1.92E-10
GO:0019395	fatty acid oxidation	61/5895	2.82E-12	2.02E-10
GO:0019318	hexose metabolic process	107/5895	2.97E-12	2.10E-10
GO:0031667	response to nutrient levels	146/5895	3.37E-12	2.36E-10
GO:0001667	ameboidal-type cell migration	172/5895	4.86E-12	3.37E-10
GO:0010976	positive regulation of neuron projection development	154/5895	5.27E-12	3.61E-10
GO:0009062	fatty acid catabolic process	58/5895	6.35E-12	4.31E-10
GO:0061008	hepaticobiliary system development	69/5895	6.52E-12	4.38E-10
GO:0010810	regulation of cell-substrate adhesion	100/5895	7.92E-12	5.26E-10
GO:0046578	regulation of Ras protein signal transduction	109/5895	8.67E-12	5.70E-10
GO:0009895	negative regulation of catabolic process	133/5895	9.93E-12	6.46E-10
GO:0051090	regulation of DNA-binding transcription factor activity	167/5895	1.09E-11	7.01E-10
GO:0048638	regulation of developmental growth	165/5895	1.14E-11	7.26E-10
GO:1901606	alpha-amino acid catabolic process	47/5895	1.70E-11	1.07E-09
GO:0060560	developmental growth involved in morphogenesis	120/5895	2.08E-11	1.30E-09
GO:0051098	regulation of binding	158/5895	2.11E-11	1.30E-09
GO:0009991	response to extracellular stimulus	154/5895	2.28E-11	1.40E-09
GO:0031346	positive regulation of cell projection organization	186/5895	2.34E-11	1.42E-09

GO:0032868	response to insulin	106/5895	2.78E-11	1.67E-09
GO:0090132	epithelium migration	127/5895	2.91E-11	1.73E-09
GO:0034440	lipid oxidation	62/5895	2.95E-11	1.74E-09
GO:0010631	epithelial cell migration	126/5895	3.82E-11	2.23E-09
GO:0055092	sterol homeostasis	53/5895	4.32E-11	2.50E-09
GO:1901617	organic hydroxy compound biosynthetic process	102/5895	4.55E-11	2.61E-09
GO:0090130	tissue migration	127/5895	5.00E-11	2.84E-09
GO:0007160	cell-matrix adhesion	98/5895	5.37E-11	3.02E-09
GO:0010565	regulation of cellular ketone metabolic process	71/5895	5.53E-11	3.08E-09
GO:0016051	carbohydrate biosynthetic process	92/5895	5.57E-11	3.08E-09
GO:0034329	cell junction assembly	168/5895	6.34E-11	3.48E-09
GO:0032869	cellular response to insulin stimulus	93/5895	8.25E-11	4.49E-09
GO:1901342	regulation of vasculature development	145/5895	8.86E-11	4.78E-09
GO:0042632	cholesterol homeostasis	52/5895	9.95E-11	5.32E-09
GO:0050678	regulation of epithelial cell proliferation	150/5895	1.01E-10	5.37E-09
GO:0000209	protein polyubiquitination	105/5895	1.26E-10	6.60E-09
GO:0043542	endothelial cell migration	96/5895	1.80E-10	9.40E-09
GO:0046889	positive regulation of lipid biosynthetic process	55/5895	2.14E-10	1.11E-08

GO:0043648	dicarboxylic acid metabolic process	49/5895	2.24E-10	1.14E-08
GO:0046364	monosaccharide biosynthetic process	49/5895	2.24E-10	1.14E-08
GO:0030522	intracellular receptor signaling pathway	94/5895	2.31E-10	1.16E-08
GO:0001655	urogenital system development	150/5895	2.52E-10	1.26E-08
GO:0006575	cellular modified amino acid metabolic process	79/5895	2.81E-10	1.40E-08
GO:0001676	long-chain fatty acid metabolic process	59/5895	2.98E-10	1.47E-08
GO:0006635	fatty acid beta-oxidation	43/5895	3.22E-10	1.57E-08
GO:0001678	cellular glucose homeostasis	78/5895	3.76E-10	1.82E-08
GO:0045785	positive regulation of cell adhesion	168/5895	4.20E-10	2.02E-08
GO:0010769	regulation of cell morphogenesis involved in differentiation	141/5895	4.95E-10	2.36E-08
GO:0019319	hexose biosynthetic process	46/5895	5.02E-10	2.38E-08
GO:0060070	canonical Wnt signaling pathway	119/5895	5.50E-10	2.59E-08
GO:0046942	carboxylic acid transport	135/5895	5.64E-10	2.63E-08
GO:0120254	olefinic compound metabolic process	64/5895	6.50E-10	3.00E-08
GO:0043547	positive regulation of GTPase activity	98/5895	6.51E-10	3.00E-08
GO:0043087	regulation of GTPase activity	125/5895	6.67E-10	3.05E-08
GO:0042445	hormone metabolic process	89/5895	6.88E-10	3.05E-08
GO:0006695	cholesterol biosynthetic process	33/5895	6.92E-10	3.05E-08

GO:0090207	regulation of triglyceride metabolic process	33/5895	6.92E-10	3.05E-08
GO:1902653	secondary alcohol biosynthetic process	33/5895	6.92E-10	3.05E-08
GO:0060828	regulation of canonical Wnt signaling pathway	102/5895	6.95E-10	3.05E-08
GO:0015849	organic acid transport	136/5895	6.97E-10	3.05E-08
GO:0070371	ERK1 and ERK2 cascade	132/5895	7.57E-10	3.29E-08
GO:1901568	fatty acid derivative metabolic process	74/5895	8.28E-10	3.58E-08
GO:0010256	endomembrane system organization	159/5895	8.65E-10	3.71E-08
GO:1990778	protein localization to cell periphery	144/5895	9.00E-10	3.84E-08
GO:0048193	Golgi vesicle transport	113/5895	9.56E-10	4.03E-08
GO:0040013	negative regulation of locomotion	132/5895	9.58E-10	4.03E-08
GO:0045765	regulation of angiogenesis	130/5895	9.96E-10	4.16E-08
GO:0019218	regulation of steroid metabolic process	58/5895	1.00E-09	4.16E-08
GO:0014855	striated muscle cell proliferation	45/5895	1.17E-09	4.82E-08
GO:0071322	cellular response to carbohydrate stimulus	74/5895	1.20E-09	4.89E-08
GO:0007409	axonogenesis	181/5895	1.20E-09	4.89E-08
GO:0033002	muscle cell proliferation	102/5895	1.23E-09	4.97E-08
GO:0106106	cold-induced thermogenesis	70/5895	1.25E-09	4.99E-08
GO:0120161	regulation of cold-induced thermogenesis	70/5895	1.25E-09	4.99E-08

GO:0051961	negative regulation of nervous system development	144/5895	1.39E-09	5.53E-08
GO:0006094	gluconeogenesis	44/5895	1.53E-09	6.01E-08
GO:0044272	sulfur compound biosynthetic process	48/5895	1.53E-09	6.01E-08
GO:1990845	adaptive thermogenesis	75/5895	1.83E-09	7.14E-08
GO:0016236	macroautophagy	91/5895	1.86E-09	7.19E-08
GO:0009410	response to xenobiotic stimulus	57/5895	2.10E-09	8.07E-08
GO:0010632	regulation of epithelial cell migration	101/5895	2.13E-09	8.15E-08
GO:0030258	lipid modification	106/5895	2.15E-09	8.18E-08
GO:0070372	regulation of ERK1 and ERK2 cascade	125/5895	2.23E-09	8.38E-08
GO:0019217	regulation of fatty acid metabolic process	52/5895	2.23E-09	8.38E-08
GO:0072001	renal system development	133/5895	2.35E-09	8.76E-08
GO:0009743	response to carbohydrate	90/5895	2.45E-09	9.11E-08
GO:0071559	response to transforming growth factor beta	93/5895	2.60E-09	9.59E-08
GO:0043255	regulation of carbohydrate biosynthetic process	56/5895	2.81E-09	1.03E-07
GO:0034284	response to monosaccharide	85/5895	2.92E-09	1.06E-07
GO:0071466	cellular response to xenobiotic stimulus	54/5895	3.20E-09	1.16E-07
GO:0048588	developmental cell growth	111/5895	3.52E-09	1.27E-07
GO:0042063	gliogenesis	134/5895	3.57E-09	1.28E-07

GO:0048738	cardiac muscle tissue development	108/5895	3.61E-09	1.29E-07
GO:0030099	myeloid cell differentiation	158/5895	3.67E-09	1.30E-07
GO:0071326	cellular response to monosaccharide stimulus	70/5895	3.80E-09	1.34E-07
GO:0002573	myeloid leukocyte differentiation	95/5895	4.72E-09	1.66E-07
GO:0033559	unsaturated fatty acid metabolic process	60/5895	4.79E-09	1.67E-07
GO:0006644	phospholipid metabolic process	145/5895	4.81E-09	1.67E-07
GO:0090181	regulation of cholesterol metabolic process	25/5895	4.86E-09	1.68E-07
GO:0006633	fatty acid biosynthetic process	69/5895	5.08E-09	1.74E-07
GO:0009749	response to glucose	83/5895	5.12E-09	1.74E-07
GO:0010721	negative regulation of cell development	149/5895	5.12E-09	1.74E-07
GO:0009746	response to hexose	84/5895	5.26E-09	1.78E-07
GO:0008286	insulin receptor signaling pathway	61/5895	5.35E-09	1.79E-07
GO:0001822	kidney development	127/5895	5.36E-09	1.79E-07
GO:0030336	negative regulation of cell migration	114/5895	5.55E-09	1.84E-07
GO:0042326	negative regulation of phosphorylation	175/5895	5.61E-09	1.85E-07
GO:0045927	positive regulation of growth	125/5895	5.63E-09	1.85E-07
GO:0006805	xenobiotic metabolic process	52/5895	5.71E-09	1.86E-07
GO:1901361	organic cyclic compound catabolic process	166/5895	5.72E-09	1.86E-07

GO:0022612	gland morphogenesis	72/5895	6.16E-09	1.99E-07
GO:0048545	response to steroid hormone	94/5895	6.20E-09	2.00E-07
GO:0071331	cellular response to hexose stimulus	69/5895	7.26E-09	2.32E-07
GO:0009611	response to wounding	181/5895	7.29E-09	2.32E-07
GO:0051271	negative regulation of cellular component movement	129/5895	7.93E-09	2.51E-07
GO:0045444	fat cell differentiation	105/5895	7.96E-09	2.51E-07
GO:0001659	temperature homeostasis	81/5895	8.97E-09	2.82E-07
GO:0001558	regulation of cell growth	164/5895	9.02E-09	2.82E-07
GO:0071333	cellular response to glucose stimulus	68/5895	9.69E-09	3.01E-07
GO:0045017	glycerolipid biosynthetic process	75/5895	1.00E-08	3.09E-07
GO:0071900	regulation of protein serine/threonine kinase activity	165/5895	1.04E-08	3.20E-07
GO:0071560	cellular response to transforming growth factor beta stimulus	90/5895	1.05E-08	3.23E-07
GO:0016126	sterol biosynthetic process	34/5895	1.08E-08	3.30E-07
GO:0050768	negative regulation of neurogenesis	132/5895	1.12E-08	3.39E-07
GO:0010770	positive regulation of cell morphogenesis involved in differentiation	83/5895	1.27E-08	3.85E-07
GO:0010811	positive regulation of cell-substrate adhesion	62/5895	1.28E-08	3.85E-07
GO:2000146	negative regulation of cell motility	117/5895	1.35E-08	4.03E-07
GO:0032922	circadian regulation of gene expression	40/5895	1.41E-08	4.19E-07

GO:0043161	proteasome-mediated ubiquitin-dependent protein catabolic process	153/5895	1.42E-08	4.20E-07
GO:0006084	acetyl-CoA metabolic process	24/5895	1.42E-08	4.20E-07
GO:0010498	proteasomal protein catabolic process	173/5895	1.49E-08	4.37E-07
GO:0045913	positive regulation of carbohydrate metabolic process	47/5895	1.52E-08	4.44E-07
GO:0008643	carbohydrate transport	70/5895	1.54E-08	4.47E-07
GO:0072659	protein localization to plasma membrane	118/5895	1.65E-08	4.79E-07
GO:0010594	regulation of endothelial cell migration	75/5895	1.90E-08	5.47E-07
GO:0060419	heart growth	58/5895	1.91E-08	5.47E-07
GO:0030177	positive regulation of Wnt signaling pathway	64/5895	2.16E-08	6.16E-07
GO:0007266	Rho protein signal transduction	80/5895	2.18E-08	6.19E-07
GO:0035023	regulation of Rho protein signal transduction	65/5895	2.30E-08	6.49E-07
GO:0046165	alcohol biosynthetic process	65/5895	2.30E-08	6.49E-07
GO:0016358	dendrite development	121/5895	2.39E-08	6.69E-07
GO:0030178	negative regulation of Wnt signaling pathway	73/5895	2.44E-08	6.80E-07
GO:0015748	organophosphate ester transport	52/5895	3.29E-08	9.13E-07
GO:1905952	regulation of lipid localization	74/5895	3.45E-08	9.54E-07
GO:0072594	establishment of protein localization to organelle	158/5895	3.48E-08	9.59E-07
GO:0042060	wound healing	134/5895	3.60E-08	9.87E-07

GO:0019915	lipid storage	44/5895	3.61E-08	9.87E-07
GO:1903008	organelle disassembly	50/5895	3.87E-08	1.05E-06
GO:1903034	regulation of response to wounding	81/5895	3.98E-08	1.08E-06
GO:0010506	regulation of autophagy	111/5895	4.86E-08	1.31E-06
GO:0002064	epithelial cell development	104/5895	5.70E-08	1.53E-06
GO:2001233	regulation of apoptotic signaling pathway	153/5895	6.20E-08	1.66E-06
GO:0051235	maintenance of location	124/5895	6.28E-08	1.67E-06
GO:0006111	regulation of gluconeogenesis	32/5895	6.54E-08	1.73E-06
GO:0001503	ossification	147/5895	6.73E-08	1.78E-06
GO:0003018	vascular process in circulatory system	93/5895	6.79E-08	1.78E-06
GO:0043122	regulation of I-kappaB kinase/NF-kappaB signaling	80/5895	6.95E-08	1.82E-06
GO:0007249	I-kappaB kinase/NF-kappaB signaling	90/5895	6.97E-08	1.82E-06
GO:1901293	nucleoside phosphate biosynthetic process	88/5895	7.05E-08	1.83E-06
GO:0051099	positive regulation of binding	85/5895	7.10E-08	1.84E-06
GO:0045860	positive regulation of protein kinase activity	161/5895	7.29E-08	1.88E-06
GO:0022407	regulation of cell-cell adhesion	157/5895	7.37E-08	1.88E-06
GO:0050994	regulation of lipid catabolic process	36/5895	7.39E-08	1.88E-06
GO:0060038	cardiac muscle cell proliferation	36/5895	7.39E-08	1.88E-06

GO:0042391	regulation of membrane potential	164/5895	7.51E-08	1.90E-06
GO:0003007	heart morphogenesis	110/5895	7.80E-08	1.97E-06
GO:0034504	protein localization to nucleus	115/5895	8.40E-08	2.11E-06
GO:0018212	peptidyl-tyrosine modification	125/5895	9.10E-08	2.28E-06
GO:0035265	organ growth	87/5895	9.20E-08	2.29E-06
GO:0009165	nucleotide biosynthetic process	86/5895	9.24E-08	2.30E-06
GO:0090287	regulation of cellular response to growth factor stimulus	111/5895	9.45E-08	2.34E-06
GO:0008652	cellular amino acid biosynthetic process	35/5895	9.73E-08	2.40E-06
GO:0046777	protein autophosphorylation	98/5895	1.03E-07	2.53E-06
GO:0034446	substrate adhesion-dependent cell spreading	52/5895	1.10E-07	2.69E-06
GO:0006091	generation of precursor metabolites and energy	148/5895	1.11E-07	2.69E-06
GO:0048639	positive regulation of developmental growth	90/5895	1.16E-07	2.81E-06
GO:0050808	synapse organization	172/5895	1.17E-07	2.82E-06
GO:1904375	regulation of protein localization to cell periphery	64/5895	1.19E-07	2.86E-06
GO:1903362	regulation of cellular protein catabolic process	102/5895	1.19E-07	2.86E-06
GO:0006892	post-Golgi vesicle-mediated transport	49/5895	1.19E-07	2.86E-06
GO:0015914	phospholipid transport	43/5895	1.24E-07	2.94E-06
GO:0048872	homeostasis of number of cells	126/5895	1.31E-07	3.10E-06

GO:0006913	nucleocytoplasmic transport	119/5895	1.38E-07	3.23E-06
GO:0051169	nuclear transport	119/5895	1.38E-07	3.23E-06
GO:0046460	neutral lipid biosynthetic process	29/5895	1.38E-07	3.23E-06
GO:0046463	acylglycerol biosynthetic process	29/5895	1.38E-07	3.23E-06
GO:0098742	cell-cell adhesion via plasma-membrane adhesion molecules	87/5895	1.54E-07	3.59E-06
GO:0010038	response to metal ion	108/5895	1.59E-07	3.69E-06
GO:0001933	negative regulation of protein phosphorylation	155/5895	1.61E-07	3.71E-06
GO:1903320	regulation of protein modification by small protein conjugation or removal	97/5895	1.68E-07	3.87E-06
GO:0010907	positive regulation of glucose metabolic process	28/5895	1.76E-07	4.04E-06
GO:0018108	peptidyl-tyrosine phosphorylation	123/5895	1.78E-07	4.07E-06
GO:0030900	forebrain development	145/5895	1.80E-07	4.10E-06
GO:0016569	covalent chromatin modification	166/5895	1.84E-07	4.17E-06
GO:0046323	glucose import	43/5895	1.94E-07	4.38E-06
GO:0019369	arachidonic acid metabolic process	38/5895	2.03E-07	4.58E-06
GO:0032102	negative regulation of response to external stimulus	142/5895	2.06E-07	4.62E-06
GO:0001952	regulation of cell-matrix adhesion	56/5895	2.18E-07	4.88E-06
GO:0050810	regulation of steroid biosynthetic process	41/5895	2.21E-07	4.93E-06
GO:0051091	positive regulation of DNA-binding transcription factor activity	103/5895	2.28E-07	5.08E-06

GO:0055017	cardiac muscle tissue growth	52/5895	2.36E-07	5.22E-06
GO:1904659	glucose transmembrane transport	53/5895	2.55E-07	5.62E-06
GO:0050679	positive regulation of epithelial cell proliferation	86/5895	2.58E-07	5.66E-06
GO:0048771	tissue remodeling	82/5895	2.68E-07	5.87E-06
GO:0051052	regulation of DNA metabolic process	140/5895	2.69E-07	5.87E-06
GO:0015749	monosaccharide transmembrane transport	54/5895	2.73E-07	5.94E-06
GO:0070936	protein K48-linked ubiquitination	35/5895	2.88E-07	6.23E-06
GO:0042738	exogenous drug catabolic process	31/5895	2.88E-07	6.23E-06
GO:1901888	regulation of cell junction assembly	88/5895	3.20E-07	6.89E-06
GO:0007162	negative regulation of cell adhesion	112/5895	3.25E-07	6.97E-06
GO:0002040	sprouting angiogenesis	57/5895	3.26E-07	6.97E-06
GO:0060538	skeletal muscle organ development	85/5895	3.35E-07	7.13E-06
GO:0043409	negative regulation of MAPK cascade	74/5895	3.56E-07	7.55E-06
GO:0048813	dendrite morphogenesis	77/5895	3.57E-07	7.55E-06
GO:0045665	negative regulation of neuron differentiation	102/5895	3.64E-07	7.68E-06
GO:0008645	hexose transmembrane transport	53/5895	3.65E-07	7.68E-06
GO:0034219	carbohydrate transmembrane transport	54/5895	3.89E-07	8.15E-06
GO:0031330	negative regulation of cellular catabolic process	99/5895	3.97E-07	8.28E-06

GO:0030072	peptide hormone secretion	110/5895	4.27E-07	8.89E-06
GO:0150115	cell-substrate junction organization	46/5895	4.31E-07	8.94E-06
GO:0043405	regulation of MAP kinase activity	115/5895	4.35E-07	9.00E-06
GO:0030856	regulation of epithelial cell differentiation	68/5895	4.51E-07	9.30E-06
GO:0090090	negative regulation of canonical Wnt signaling pathway	58/5895	4.75E-07	9.77E-06
GO:0009064	glutamine family amino acid metabolic process	35/5895	4.82E-07	9.87E-06
GO:0042737	drug catabolic process	31/5895	5.13E-07	1.05E-05
GO:0022411	cellular component disassembly	140/5895	5.39E-07	1.10E-05
GO:0050680	negative regulation of epithelial cell proliferation	65/5895	5.78E-07	1.17E-05
GO:0043534	blood vessel endothelial cell migration	55/5895	5.79E-07	1.17E-05
GO:0014013	regulation of gliogenesis	67/5895	5.94E-07	1.20E-05
GO:1903828	negative regulation of cellular protein localization	56/5895	6.06E-07	1.22E-05
GO:1905954	positive regulation of lipid localization	50/5895	6.09E-07	1.22E-05
GO:0007179	transforming growth factor beta receptor signaling pathway	72/5895	6.12E-07	1.22E-05
GO:0008654	phospholipid biosynthetic process	73/5895	6.12E-07	1.22E-05
GO:0034754	cellular hormone metabolic process	51/5895	6.52E-07	1.29E-05
GO:0007030	Golgi organization	58/5895	6.56E-07	1.30E-05
GO:0072576	liver morphogenesis	22/5895	6.58E-07	1.30E-05

GO:0017144	drug metabolic process	37/5895	7.11E-07	1.40E-05
GO:0019439	aromatic compound catabolic process	148/5895	7.17E-07	1.40E-05
GO:0099173	postsynapse organization	82/5895	7.30E-07	1.43E-05
GO:0009914	hormone transport	136/5895	7.70E-07	1.50E-05
GO:0009755	hormone-mediated signaling pathway	69/5895	7.99E-07	1.55E-05
GO:0050730	regulation of peptidyl-tyrosine phosphorylation	102/5895	8.48E-07	1.64E-05
GO:0035773	insulin secretion involved in cellular response to glucose stimulus	45/5895	8.55E-07	1.65E-05
GO:0006979	response to oxidative stress	147/5895	8.85E-07	1.70E-05
GO:0006690	icosanoid metabolic process	58/5895	9.01E-07	1.72E-05
GO:0019432	triglyceride biosynthetic process	24/5895	9.03E-07	1.72E-05
GO:0045940	positive regulation of steroid metabolic process	24/5895	9.03E-07	1.72E-05
GO:0034599	cellular response to oxidative stress	106/5895	9.04E-07	1.72E-05
GO:0045165	cell fate commitment	111/5895	9.12E-07	1.73E-05
GO:0060541	respiratory system development	99/5895	9.37E-07	1.77E-05
GO:0000422	autophagy of mitochondrion	36/5895	9.52E-07	1.79E-05
GO:0061726	mitochondrion disassembly	36/5895	9.52E-07	1.79E-05
GO:0030073	insulin secretion	91/5895	9.58E-07	1.79E-05
GO:0034248	regulation of cellular amide metabolic process	147/5895	1.04E-06	1.94E-05

GO:0046326	positive regulation of glucose import	26/5895	1.08E-06	2.00E-05
GO:0050770	regulation of axonogenesis	84/5895	1.13E-06	2.10E-05
GO:0043470	regulation of carbohydrate catabolic process	32/5895	1.14E-06	2.11E-05
GO:0007044	cell-substrate junction assembly	44/5895	1.15E-06	2.12E-05
GO:0062197	cellular response to chemical stress	123/5895	1.16E-06	2.14E-05
GO:0010883	regulation of lipid storage	28/5895	1.17E-06	2.14E-05
GO:0007178	transmembrane receptor protein serine/threonine kinase signaling pathway	134/5895	1.19E-06	2.17E-05
GO:0031647	regulation of protein stability	109/5895	1.20E-06	2.18E-05
GO:0042752	regulation of circadian rhythm	58/5895	1.23E-06	2.23E-05
GO:0001654	eye development	149/5895	1.29E-06	2.34E-05
GO:1901343	negative regulation of vasculature development	61/5895	1.31E-06	2.36E-05
GO:0061572	actin filament bundle organization	72/5895	1.36E-06	2.43E-05
GO:0060021	roof of mouth development	46/5895	1.36E-06	2.43E-05
GO:0006898	receptor-mediated endocytosis	94/5895	1.36E-06	2.43E-05
GO:0051017	actin filament bundle assembly	71/5895	1.36E-06	2.44E-05
GO:0071902	positive regulation of protein serine/threonine kinase activity	110/5895	1.39E-06	2.48E-05
GO:0030301	cholesterol transport	42/5895	1.40E-06	2.48E-05
GO:0009074	aromatic amino acid family catabolic process	15/5895	1.43E-06	2.51E-05

GO:0032239	regulation of nucleobase-containing compound transport	15/5895	1.43E-06	2.51E-05
GO:0045540	regulation of cholesterol biosynthetic process	15/5895	1.43E-06	2.51E-05
GO:0106118	regulation of sterol biosynthetic process	15/5895	1.43E-06	2.51E-05
GO:0031098	stress-activated protein kinase signaling cascade	104/5895	1.45E-06	2.54E-05
GO:0031396	regulation of protein ubiquitination	83/5895	1.46E-06	2.56E-05
GO:2001234	negative regulation of apoptotic signaling pathway	91/5895	1.49E-06	2.59E-05
GO:0048880	sensory system development	151/5895	1.59E-06	2.77E-05
GO:0045732	positive regulation of protein catabolic process	88/5895	1.62E-06	2.81E-05
GO:0003279	cardiac septum development	57/5895	1.63E-06	2.81E-05
GO:0001763	morphogenesis of a branching structure	94/5895	1.68E-06	2.89E-05
GO:0007369	gastrulation	73/5895	1.73E-06	2.97E-05
GO:0010977	negative regulation of neuron projection development	73/5895	1.73E-06	2.97E-05
GO:0018105	peptidyl-serine phosphorylation	122/5895	1.74E-06	2.97E-05
GO:0051403	stress-activated MAPK cascade	99/5895	1.74E-06	2.97E-05
GO:0007034	vacuolar transport	61/5895	1.75E-06	2.97E-05
GO:0008206	bile acid metabolic process	21/5895	1.75E-06	2.97E-05
GO:0060977	coronary vasculature morphogenesis	21/5895	1.75E-06	2.97E-05
GO:0061041	regulation of wound healing	63/5895	1.78E-06	3.00E-05

GO:0070302	regulation of stress-activated protein kinase signaling cascade	92/5895	1.79E-06	3.00E-05
GO:0090276	regulation of peptide hormone secretion	92/5895	1.79E-06	3.00E-05
GO:0001935	endothelial cell proliferation	64/5895	1.79E-06	3.00E-05
GO:0046879	hormone secretion	132/5895	1.82E-06	3.04E-05
GO:0007015	actin filament organization	154/5895	1.85E-06	3.09E-05
GO:0120162	positive regulation of cold-induced thermogenesis	46/5895	1.95E-06	3.23E-05
GO:1903706	regulation of hemopoiesis	150/5895	1.95E-06	3.23E-05
GO:0030307	positive regulation of cell growth	80/5895	1.97E-06	3.25E-05
GO:1990138	neuron projection extension	80/5895	1.97E-06	3.25E-05
GO:0010676	positive regulation of cellular carbohydrate metabolic process	35/5895	2.02E-06	3.32E-05
GO:0015918	sterol transport	42/5895	2.06E-06	3.38E-05
GO:0071692	protein localization to extracellular region	166/5895	2.08E-06	3.39E-05
GO:0016570	histone modification	157/5895	2.14E-06	3.49E-05
GO:0070373	negative regulation of ERK1 and ERK2 cascade	39/5895	2.26E-06	3.67E-05
GO:0006865	amino acid transport	68/5895	2.34E-06	3.79E-05
GO:0010508	positive regulation of autophagy	62/5895	2.35E-06	3.79E-05
GO:0071383	cellular response to steroid hormone stimulus	67/5895	2.35E-06	3.80E-05
GO:0010634	positive regulation of epithelial cell migration	64/5895	2.36E-06	3.80E-05

GO:0150063	visual system development	149/5895	2.39E-06	3.84E-05
GO:1901607	alpha-amino acid biosynthetic process	31/5895	2.52E-06	4.03E-05
GO:0038127	ERBB signaling pathway	51/5895	2.53E-06	4.04E-05
GO:0007519	skeletal muscle tissue development	79/5895	2.54E-06	4.05E-05
GO:1903050	regulation of proteolysis involved in cellular protein catabolic process	87/5895	2.58E-06	4.10E-05
GO:0002791	regulation of peptide secretion	144/5895	2.64E-06	4.19E-05
GO:1904019	epithelial cell apoptotic process	53/5895	2.71E-06	4.29E-05
GO:0045833	negative regulation of lipid metabolic process	46/5895	2.78E-06	4.39E-05
GO:0009306	protein secretion	163/5895	2.80E-06	4.40E-05
GO:0033866	nucleoside bisphosphate biosynthetic process	22/5895	2.91E-06	4.54E-05
GO:0034030	ribonucleoside bisphosphate biosynthetic process	22/5895	2.91E-06	4.54E-05
GO:0034033	purine nucleoside bisphosphate biosynthetic process	22/5895	2.91E-06	4.54E-05
GO:0060562	epithelial tube morphogenesis	138/5895	3.05E-06	4.76E-05
GO:0046324	regulation of glucose import	35/5895	3.16E-06	4.91E-05
GO:0050773	regulation of dendrite development	79/5895	3.20E-06	4.95E-05
GO:0061138	morphogenesis of a branching epithelium	87/5895	3.20E-06	4.95E-05
GO:0035592	establishment of protein localization to extracellular region	163/5895	3.22E-06	4.97E-05
GO:0048754	branching morphogenesis of an epithelial tube	75/5895	3.50E-06	5.38E-05

GO:0034332	adherens junction organization	28/5895	3.56E-06	5.47E-05
GO:0042149	cellular response to glucose starvation	26/5895	3.59E-06	5.50E-05
GO:0050708	regulation of protein secretion	131/5895	3.68E-06	5.62E-05
GO:0031929	TOR signaling	54/5895	3.78E-06	5.74E-05
GO:0090101	negative regulation of transmembrane receptor protein serine/threonine kinase signaling pathway	54/5895	3.78E-06	5.74E-05
GO:0055074	calcium ion homeostasis	169/5895	4.08E-06	6.19E-05
GO:0051054	positive regulation of DNA metabolic process	85/5895	4.22E-06	6.39E-05
GO:0035337	fatty-acyl-CoA metabolic process	13/5895	4.27E-06	6.43E-05
GO:0046831	regulation of RNA export from nucleus	13/5895	4.27E-06	6.43E-05
GO:0021537	telencephalon development	96/5895	4.32E-06	6.49E-05
GO:0006699	bile acid biosynthetic process	15/5895	4.36E-06	6.53E-05
GO:0045446	endothelial cell differentiation	49/5895	4.48E-06	6.68E-05
GO:0015850	organic hydroxy compound transport	95/5895	4.49E-06	6.68E-05
GO:0034976	response to endoplasmic reticulum stress	95/5895	4.49E-06	6.68E-05
GO:0019373	epoxygenase P450 pathway	20/5895	4.61E-06	6.75E-05
GO:0032373	positive regulation of sterol transport	20/5895	4.61E-06	6.75E-05
GO:0032376	positive regulation of cholesterol transport	20/5895	4.61E-06	6.75E-05
GO:0072574	hepatocyte proliferation	20/5895	4.61E-06	6.75E-05

GO:0072575	epithelial cell proliferation involved in liver morphogenesis	20/5895	4.61E-06	6.75E-05
GO:0007219	Notch signaling pathway	73/5895	4.61E-06	6.75E-05
GO:1901861	regulation of muscle tissue development	73/5895	4.61E-06	6.75E-05
GO:0030316	osteoclast differentiation	50/5895	4.64E-06	6.77E-05
GO:0016202	regulation of striated muscle tissue development	72/5895	4.70E-06	6.85E-05
GO:0045216	cell-cell junction organization	81/5895	4.76E-06	6.92E-05
GO:1903076	regulation of protein localization to plasma membrane	51/5895	4.77E-06	6.92E-05
GO:0048041	focal adhesion assembly	39/5895	4.99E-06	7.22E-05
GO:0018209	peptidyl-serine modification	128/5895	5.01E-06	7.23E-05
GO:0072522	purine-containing compound biosynthetic process	68/5895	5.02E-06	7.23E-05
GO:2000181	negative regulation of blood vessel morphogenesis	55/5895	5.16E-06	7.42E-05
GO:0016311	dephosphorylation	133/5895	5.36E-06	7.68E-05
GO:0061178	regulation of insulin secretion involved in cellular response to glucose stimulus	40/5895	5.43E-06	7.76E-05
GO:0050796	regulation of insulin secretion	76/5895	5.44E-06	7.76E-05
GO:0032006	regulation of TOR signaling	46/5895	5.51E-06	7.85E-05
GO:0043112	receptor metabolic process	83/5895	5.56E-06	7.90E-05
GO:0043393	regulation of protein binding	89/5895	5.60E-06	7.94E-05
GO:0090092	regulation of transmembrane receptor protein serine/threonine kinase signaling pathway	94/5895	5.67E-06	8.03E-05

GO:0010893	positive regulation of steroid biosynthetic process	19/5895	5.73E-06	8.06E-05
GO:0090208	positive regulation of triglyceride metabolic process	19/5895	5.73E-06	8.06E-05
GO:0007173	epidermal growth factor receptor signaling pathway	47/5895	5.75E-06	8.06E-05
GO:0010595	positive regulation of endothelial cell migration	47/5895	5.75E-06	8.06E-05
GO:0048634	regulation of muscle organ development	73/5895	5.83E-06	8.16E-05
GO:0003012	muscle system process	143/5895	5.91E-06	8.25E-05
GO:0051057	positive regulation of small GTPase mediated signal transduction	37/5895	6.09E-06	8.48E-05
GO:0030278	regulation of ossification	92/5895	6.12E-06	8.51E-05
GO:0006874	cellular calcium ion homeostasis	163/5895	6.42E-06	8.91E-05
GO:0060249	anatomical structure homeostasis	147/5895	6.44E-06	8.91E-05
GO:0006164	purine nucleotide biosynthetic process	66/5895	6.61E-06	9.13E-05
GO:0046390	ribose phosphate biosynthetic process	65/5895	6.70E-06	9.23E-05
GO:0032259	methylation	125/5895	6.81E-06	9.36E-05
GO:0032872	regulation of stress-activated MAPK cascade	89/5895	6.85E-06	9.40E-05
GO:1900180	regulation of protein localization to nucleus	59/5895	6.98E-06	9.55E-05
GO:0034612	response to tumor necrosis factor	75/5895	7.00E-06	9.55E-05
GO:0021675	nerve development	39/5895	7.28E-06	9.93E-05
GO:0006766	vitamin metabolic process	35/5895	7.36E-06	9.99E-05

GO:0014009	glial cell proliferation	29/5895	7.36E-06	9.99E-05
GO:0007041	lysosomal transport	46/5895	7.65E-06	0.000103388
GO:0032092	positive regulation of protein binding	46/5895	7.65E-06	0.000103388
GO:0032970	regulation of actin filament-based process	143/5895	7.92E-06	0.000106683
GO:0046849	bone remodeling	47/5895	7.93E-06	0.000106683
GO:0007005	mitochondrion organization	164/5895	7.96E-06	0.000106908
GO:0048017	inositol lipid-mediated signaling	68/5895	8.18E-06	0.000109238
GO:0030518	intracellular steroid hormone receptor signaling pathway	48/5895	8.18E-06	0.000109238
GO:0017038	protein import	84/5895	8.19E-06	0.000109238
GO:0050731	positive regulation of peptidyl-tyrosine phosphorylation	77/5895	8.28E-06	0.000110151
GO:0010001	glial cell differentiation	94/5895	8.35E-06	0.000110863
GO:0046700	heterocycle catabolic process	139/5895	8.42E-06	0.000111567
GO:0035384	thioester biosynthetic process	17/5895	8.50E-06	0.000112232
GO:0071616	acyl-CoA biosynthetic process	17/5895	8.50E-06	0.000112232
GO:0046503	glycerolipid catabolic process	33/5895	8.78E-06	0.000115597
GO:0044403	symbiotic process	132/5895	8.93E-06	0.000117358
GO:0090205	positive regulation of cholesterol metabolic process	Oct-95	8.95E-06	0.000117358
GO:0003158	endothelium development	53/5895	8.99E-06	0.000117358

GO:0016525	negative regulation of angiogenesis	53/5895	8.99E-06	0.000117358
GO:0045598	regulation of fat cell differentiation	61/5895	9.00E-06	0.000117358
GO:0001666	response to hypoxia	81/5895	9.06E-06	0.000117635
GO:1904018	positive regulation of vasculature development	81/5895	9.06E-06	0.000117635
GO:0044270	cellular nitrogen compound catabolic process	137/5895	9.33E-06	0.000120876
GO:0048668	collateral sprouting	20/5895	9.36E-06	0.000121064
GO:0032103	positive regulation of response to external stimulus	145/5895	9.51E-06	0.00012276
GO:0035150	regulation of tube size	71/5895	9.70E-06	0.000124907
GO:0010828	positive regulation of glucose transmembrane transport	28/5895	9.87E-06	0.000126537
GO:0060612	adipose tissue development	28/5895	9.87E-06	0.000126537
GO:1901264	carbohydrate derivative transport	34/5895	9.90E-06	0.000126647
GO:0036293	response to decreased oxygen levels	89/5895	1.02E-05	0.000129893
GO:0062014	negative regulation of small molecule metabolic process	45/5895	1.02E-05	0.000129893
GO:1901616	organic hydroxy compound catabolic process	31/5895	1.03E-05	0.000130959
GO:0006606	protein import into nucleus	67/5895	1.06E-05	0.0001341
GO:2001236	regulation of extrinsic apoptotic signaling pathway	67/5895	1.06E-05	0.0001341
GO:0009072	aromatic amino acid family metabolic process	22/5895	1.07E-05	0.000135004
GO:0016032	viral process	112/5895	1.09E-05	0.00013714

GO:0050727	regulation of inflammatory response	131/5895	1.09E-05	0.000137686
GO:0006650	glycerophospholipid metabolic process	96/5895	1.12E-05	0.000140435
GO:0009260	ribonucleotide biosynthetic process	62/5895	1.15E-05	0.000144029
GO:0019221	cytokine-mediated signaling pathway	130/5895	1.15E-05	0.000144029
GO:0005976	polysaccharide metabolic process	50/5895	1.16E-05	0.000144241
GO:0043401	steroid hormone mediated signaling pathway	50/5895	1.16E-05	0.000144241
GO:0009152	purine ribonucleotide biosynthetic process	59/5895	1.18E-05	0.000147442
GO:0010827	regulation of glucose transmembrane transport	41/5895	1.19E-05	0.000147592
GO:0050996	positive regulation of lipid catabolic process	19/5895	1.19E-05	0.000147592
GO:0043467	regulation of generation of precursor metabolites and energy	57/5895	1.20E-05	0.000148106
GO:0046822	regulation of nucleocytoplasmic transport	54/5895	1.20E-05	0.000148106
GO:0010959	regulation of metal ion transport	147/5895	1.30E-05	0.000160656
GO:0043535	regulation of blood vessel endothelial cell migration	43/5895	1.31E-05	0.000160656
GO:1904950	negative regulation of establishment of protein localization	68/5895	1.31E-05	0.000160885
GO:0008361	regulation of cell size	82/5895	1.32E-05	0.000162118
GO:0097006	regulation of plasma lipoprotein particle levels	33/5895	1.33E-05	0.000162765
GO:0051896	regulation of protein kinase B signaling	67/5895	1.34E-05	0.000163565
GO:0007416	synapse assembly	75/5895	1.36E-05	0.000165262

GO:0007031	peroxisome organization	21/5895	1.39E-05	0.000168722
GO:0055090	acylglycerol homeostasis	21/5895	1.39E-05	0.000168722
GO:0070328	triglyceride homeostasis	21/5895	1.39E-05	0.000168722
GO:0031345	negative regulation of cell projection organization	80/5895	1.42E-05	0.000171713
GO:0044264	cellular polysaccharide metabolic process	46/5895	1.44E-05	0.000173952
GO:0048592	eye morphogenesis	72/5895	1.48E-05	0.000178504
GO:0006536	glutamate metabolic process	18/5895	1.50E-05	0.000180517
GO:0090596	sensory organ morphogenesis	112/5895	1.51E-05	0.000181263
GO:0007009	plasma membrane organization	49/5895	1.53E-05	0.000183033
GO:0016052	carbohydrate catabolic process	59/5895	1.53E-05	0.000183033
GO:0035296	regulation of tube diameter	70/5895	1.57E-05	0.000186492
GO:0097746	regulation of blood vessel diameter	70/5895	1.57E-05	0.000186492
GO:0042177	negative regulation of protein catabolic process	55/5895	1.58E-05	0.000187139
GO:0061912	selective autophagy	31/5895	1.59E-05	0.000188391
GO:0045787	positive regulation of cell cycle	126/5895	1.64E-05	0.000194157
GO:0048015	phosphatidylinositol-mediated signaling	66/5895	1.73E-05	0.000204069
GO:0051224	negative regulation of protein transport	66/5895	1.73E-05	0.000204069
GO:0050920	regulation of chemotaxis	85/5895	1.76E-05	0.000206866

GO:1903670	regulation of sprouting angiogenesis	36/5895	1.76E-05	0.000206872
GO:0000266	mitochondrial fission	26/5895	1.77E-05	0.00020777
GO:0050803	regulation of synapse structure or activity	105/5895	1.79E-05	0.000209795
GO:0042692	muscle cell differentiation	138/5895	1.82E-05	0.000212771
GO:0098656	anion transmembrane transport	84/5895	1.83E-05	0.000213162
GO:0001954	positive regulation of cell-matrix adhesion	29/5895	1.86E-05	0.000216195
GO:0042446	hormone biosynthetic process	29/5895	1.86E-05	0.000216195
GO:1901657	glycosyl compound metabolic process	44/5895	1.86E-05	0.000216195
GO:0046339	diacylglycerol metabolic process	17/5895	1.88E-05	0.000217207
GO:0060976	coronary vasculature development	37/5895	1.89E-05	0.00021853
GO:0032007	negative regulation of TOR signaling	24/5895	1.90E-05	0.000219205
GO:0007033	vacuole organization	69/5895	2.01E-05	0.000231726
GO:0046883	regulation of hormone secretion	109/5895	2.06E-05	0.000236499
GO:0060759	regulation of response to cytokine stimulus	51/5895	2.07E-05	0.000237146
GO:0045807	positive regulation of endocytosis	52/5895	2.07E-05	0.000237153
GO:0030323	respiratory tube development	85/5895	2.13E-05	0.000243575
GO:0060043	regulation of cardiac muscle cell proliferation	27/5895	2.13E-05	0.000243575
GO:0050807	regulation of synapse organization	101/5895	2.16E-05	0.000246485

GO:0043010	camera-type eye development	129/5895	2.19E-05	0.00024879
GO:0030100	regulation of endocytosis	84/5895	2.22E-05	0.000251325
GO:0030324	lung development	84/5895	2.22E-05	0.000251325
GO:0060420	regulation of heart growth	40/5895	2.23E-05	0.000252053
GO:0043414	macromolecule methylation	107/5895	2.27E-05	0.000256012
GO:0031334	positive regulation of protein-containing complex assembly	88/5895	2.27E-05	0.000256138
GO:0071248	cellular response to metal ion	63/5895	2.34E-05	0.000262794
GO:0007254	JNK cascade	77/5895	2.38E-05	0.000267062
GO:0002065	columnar/cuboidal epithelial cell differentiation	61/5895	2.43E-05	0.00027282
GO:0045862	positive regulation of proteolysis	121/5895	2.46E-05	0.000275321
GO:0070542	response to fatty acid	28/5895	2.51E-05	0.00027943
GO:1902930	regulation of alcohol biosynthetic process	28/5895	2.51E-05	0.00027943
GO:0046461	neutral lipid catabolic process	23/5895	2.53E-05	0.000281351
GO:0046464	acylglycerol catabolic process	23/5895	2.53E-05	0.000281351
GO:0034381	plasma lipoprotein particle clearance	21/5895	2.55E-05	0.000282497
GO:0003205	cardiac chamber development	75/5895	2.55E-05	0.000282715
GO:0043406	positive regulation of MAP kinase activity	85/5895	2.58E-05	0.00028481
GO:0051170	import into nucleus	67/5895	2.66E-05	0.000293146

GO:0021987	cerebral cortex development	47/5895	2.67E-05	0.00029387
GO:0051259	protein complex oligomerization	84/5895	2.69E-05	0.000295256
GO:0051222	positive regulation of protein transport	122/5895	2.71E-05	0.000297794
GO:0006090	pyruvate metabolic process	51/5895	2.72E-05	0.000297866
GO:0097191	extrinsic apoptotic signaling pathway	87/5895	2.86E-05	0.000312221
GO:0031503	protein-containing complex localization	95/5895	2.86E-05	0.000312221
GO:0048524	positive regulation of viral process	39/5895	2.98E-05	0.000324353
GO:0034764	positive regulation of transmembrane transport	90/5895	3.01E-05	0.000326904
GO:0048593	camera-type eye morphogenesis	61/5895	3.09E-05	0.000335184
GO:1903311	regulation of mRNA metabolic process	97/5895	3.10E-05	0.000335649
GO:1905475	regulation of protein localization to membrane	75/5895	3.13E-05	0.000339115
GO:1902105	regulation of leukocyte differentiation	110/5895	3.16E-05	0.000341594
GO:0061448	connective tissue development	100/5895	3.18E-05	0.000342874
GO:1903364	positive regulation of cellular protein catabolic process	59/5895	3.22E-05	0.000346173
GO:0045542	positive regulation of cholesterol biosynthetic process	Sep-95	3.23E-05	0.000346849
GO:0106120	positive regulation of sterol biosynthetic process	Sep-95	3.23E-05	0.000346849
GO:0045637	regulation of myeloid cell differentiation	84/5895	3.24E-05	0.000347427
GO:1904035	regulation of epithelial cell apoptotic process	42/5895	3.29E-05	0.000352101

GO:1903322	positive regulation of protein modification by small protein conjugation or removal	57/5895	3.33E-05	0.000354258
GO:0110053	regulation of actin filament organization	99/5895	3.33E-05	0.000354258
GO:0045722	positive regulation of gluconeogenesis	14/5895	3.34E-05	0.000354258
GO:2000114	regulation of establishment of cell polarity	14/5895	3.34E-05	0.000354258
GO:0000038	very long-chain fatty acid metabolic process	20/5895	3.36E-05	0.000354551
GO:0009069	serine family amino acid metabolic process	20/5895	3.36E-05	0.000354551
GO:0060479	lung cell differentiation	20/5895	3.36E-05	0.000354551
GO:0010771	negative regulation of cell morphogenesis involved in differentiation	44/5895	3.43E-05	0.000362321
GO:0046328	regulation of JNK cascade	72/5895	3.49E-05	0.000367227
GO:0030038	contractile actin filament bundle assembly	48/5895	3.57E-05	0.000374456
GO:0043149	stress fiber assembly	48/5895	3.57E-05	0.000374456
GO:1901214	regulation of neuron death	130/5895	3.67E-05	0.000384514
GO:0043491	protein kinase B signaling	75/5895	3.83E-05	0.000400987
GO:0045926	negative regulation of growth	96/5895	3.85E-05	0.000401771
GO:0043124	negative regulation of I-kappaB kinase/NF-kappaB signaling	25/5895	3.86E-05	0.000402317
GO:0046885	regulation of hormone biosynthetic process	17/5895	3.87E-05	0.000403008
GO:0090288	negative regulation of cellular response to growth factor stimulus	61/5895	3.90E-05	0.000404989
GO:0007411	axon guidance	92/5895	3.91E-05	0.000405517

GO:1904951	positive regulation of establishment of protein localization	126/5895	3.93E-05	0.000407558
GO:0015718	monocarboxylic acid transport	68/5895	3.99E-05	0.000412327
GO:1903522	regulation of blood circulation	91/5895	4.09E-05	0.000422039
GO:0030902	hindbrain development	67/5895	4.12E-05	0.000422039
GO:0060411	cardiac septum morphogenesis	39/5895	4.13E-05	0.000422039
GO:1905897	regulation of response to endoplasmic reticulum stress	39/5895	4.13E-05	0.000422039
GO:0006605	protein targeting	98/5895	4.13E-05	0.000422039
GO:0010452	histone H3-K36 methylation	Oct-95	4.14E-05	0.000422039
GO:0035376	sterol import	Oct-95	4.14E-05	0.000422039
GO:0070508	cholesterol import	Oct-95	4.14E-05	0.000422039
GO:0051346	negative regulation of hydrolase activity	138/5895	4.14E-05	0.000422039
GO:0060485	mesenchyme development	101/5895	4.21E-05	0.000428365
GO:0048608	reproductive structure development	159/5895	4.22E-05	0.000429154
GO:0009083	branched-chain amino acid catabolic process	Dec-95	4.25E-05	0.000429845
GO:0098974	postsynaptic actin cytoskeleton organization	Dec-95	4.25E-05	0.000429845
GO:0099188	postsynaptic cytoskeleton organization	Dec-95	4.25E-05	0.000429845
GO:0021955	central nervous system neuron axonogenesis	23/5895	4.26E-05	0.000430317
GO:0090630	activation of GTPase activity	41/5895	4.37E-05	0.00044023

GO:0061614	pri-miRNA transcription by RNA polymerase II	29/5895	4.38E-05	0.00044023
GO:1902001	fatty acid transmembrane transport	Nov-95	4.41E-05	0.000443404
GO:0035264	multicellular organism growth	81/5895	4.44E-05	0.000445131
GO:0002761	regulation of myeloid leukocyte differentiation	54/5895	4.47E-05	0.000447223
GO:0009066	aspartate family amino acid metabolic process	21/5895	4.49E-05	0.00044825
GO:0016485	protein processing	85/5895	4.49E-05	0.00044825
GO:0031214	biomineral tissue development	64/5895	4.51E-05	0.000448518
GO:0110148	biomineralization	64/5895	4.51E-05	0.000448518
GO:0061458	reproductive system development	160/5895	4.52E-05	0.000448968
GO:0061387	regulation of extent of cell growth	53/5895	4.53E-05	0.000448968
GO:0002762	negative regulation of myeloid leukocyte differentiation	26/5895	4.55E-05	0.000449918
GO:0022409	positive regulation of cell-cell adhesion	96/5895	4.55E-05	0.000449918
GO:1902414	protein localization to cell junction	52/5895	4.58E-05	0.000452038
GO:2000058	regulation of ubiquitin-dependent protein catabolic process	63/5895	4.64E-05	0.000457068
GO:0097485	neuron projection guidance	92/5895	4.64E-05	0.000457068
GO:0007156	homophilic cell adhesion via plasma membrane adhesion molecules	47/5895	4.69E-05	0.000461181
GO:2000027	regulation of animal organ morphogenesis	79/5895	4.82E-05	0.000473407
GO:0009116	nucleoside metabolic process	35/5895	4.84E-05	0.000473829

GO:0032535	regulation of cellular component size	140/5895	4.84E-05	0.000473829
GO:0032878	regulation of establishment or maintenance of cell polarity	16/5895	4.92E-05	0.000479481
GO:0090114	COPII-coated vesicle budding	16/5895	4.92E-05	0.000479481
GO:0003231	cardiac ventricle development	59/5895	5.15E-05	0.000501439
GO:0010464	regulation of mesenchymal cell proliferation	24/5895	5.19E-05	0.000503594
GO:0060421	positive regulation of heart growth	24/5895	5.19E-05	0.000503594
GO:0032371	regulation of sterol transport	27/5895	5.23E-05	0.00050607
GO:0032374	regulation of cholesterol transport	27/5895	5.23E-05	0.00050607
GO:0045766	positive regulation of angiogenesis	72/5895	5.24E-05	0.000506311
GO:0072089	stem cell proliferation	58/5895	5.27E-05	0.000508632
GO:1901863	positive regulation of muscle tissue development	38/5895	5.50E-05	0.000529446
GO:0048675	axon extension	56/5895	5.51E-05	0.000529446
GO:0072331	signal transduction by p53 class mediator	55/5895	5.61E-05	0.000539069
GO:0034103	regulation of tissue remodeling	39/5895	5.67E-05	0.000543618
GO:0006730	one-carbon metabolic process	22/5895	5.71E-05	0.000546815
GO:1902893	regulation of pri-miRNA transcription by RNA polymerase II	28/5895	5.90E-05	0.000563723
GO:0099003	vesicle-mediated transport in synapse	94/5895	5.92E-05	0.000564658
GO:0001702	gastrulation with mouth forming second	20/5895	5.97E-05	0.000569009

GO:0006110	regulation of glycolytic process	25/5895	6.13E-05	0.000582908
GO:0018205	peptidyl-lysine modification	125/5895	6.30E-05	0.000598651
GO:0051146	striated muscle cell differentiation	111/5895	6.44E-05	0.000609651
GO:1903829	positive regulation of cellular protein localization	111/5895	6.44E-05	0.000609651
GO:0000271	polysaccharide biosynthetic process	35/5895	6.80E-05	0.000643398
GO:0043433	negative regulation of DNA-binding transcription factor activity	64/5895	6.95E-05	0.000656387
GO:0042304	regulation of fatty acid biosynthetic process	23/5895	6.98E-05	0.000658217
GO:0060428	lung epithelium development	26/5895	7.05E-05	0.000664161
GO:0070997	neuron death	140/5895	7.13E-05	0.000669968
GO:0032835	glomerulus development	30/5895	7.14E-05	0.00067045
GO:0006470	protein dephosphorylation	86/5895	7.30E-05	0.000684316
GO:0006417	regulation of translation	122/5895	7.38E-05	0.000691235
GO:0035051	cardiocyte differentiation	68/5895	7.44E-05	0.000695308
GO:0015931	nucleobase-containing compound transport	73/5895	7.47E-05	0.00069708
GO:0060039	pericardium development	17/5895	7.53E-05	0.000700876
GO:0060384	innervation	17/5895	7.53E-05	0.000700876
GO:0045601	regulation of endothelial cell differentiation	21/5895	7.65E-05	0.000710322
GO:0031998	regulation of fatty acid beta-oxidation	14/5895	7.67E-05	0.000710322

GO:0051963	regulation of synapse assembly	51/5895	7.68E-05	0.000710322
GO:1905039	carboxylic acid transmembrane transport	51/5895	7.68E-05	0.000710322
GO:0060487	lung epithelial cell differentiation	19/5895	7.93E-05	0.000732463
GO:0048285	organelle fission	156/5895	8.01E-05	0.000738869
GO:0070374	positive regulation of ERK1 and ERK2 cascade	80/5895	8.04E-05	0.000740873
GO:0001959	regulation of cytokine-mediated signaling pathway	46/5895	8.08E-05	0.000742927
GO:0002262	myeloid cell homeostasis	71/5895	8.09E-05	0.000742927
GO:0106027	neuron projection organization	45/5895	8.11E-05	0.000742927
GO:0055024	regulation of cardiac muscle tissue development	44/5895	8.11E-05	0.000742927
GO:0046579	positive regulation of Ras protein signal transduction	32/5895	8.22E-05	0.000751451
GO:0032881	regulation of polysaccharide metabolic process	24/5895	8.26E-05	0.000754017
GO:0030217	T cell differentiation	100/5895	8.35E-05	0.000761058
GO:0006397	mRNA processing	153/5895	8.36E-05	0.000761314
GO:0043254	regulation of protein-containing complex assembly	146/5895	8.49E-05	0.000771908
GO:0048660	regulation of smooth muscle cell proliferation	64/5895	8.58E-05	0.000777807
GO:0051147	regulation of muscle cell differentiation	64/5895	8.58E-05	0.000777807
GO:0010463	mesenchymal cell proliferation	28/5895	8.81E-05	0.000797505
GO:0043583	ear development	89/5895	8.89E-05	0.00080365

GO:2000377	regulation of reactive oxygen species metabolic process	77/5895	9.18E-05	0.000829006
GO:0009791	post-embryonic development	54/5895	9.22E-05	0.000831226
GO:0032352	positive regulation of hormone metabolic process	13/5895	9.35E-05	0.000841353
GO:0030225	macrophage differentiation	22/5895	9.39E-05	0.000842901
GO:0046164	alcohol catabolic process	22/5895	9.39E-05	0.000842901
GO:0098727	maintenance of cell number	67/5895	9.45E-05	0.000846655
GO:0060324	face development	29/5895	9.61E-05	0.000860416
GO:0008053	mitochondrial fusion	16/5895	9.78E-05	0.00087419
GO:1903825	organic acid transmembrane transport	51/5895	9.82E-05	0.000875865
GO:0045844	positive regulation of striated muscle tissue development	37/5895	0.000100342	0.000892889
GO:0048636	positive regulation of muscle organ development	37/5895	0.000100342	0.000892889
GO:0031099	regeneration	59/5895	0.000101008	0.000897551
GO:0003281	ventricular septum development	38/5895	0.000102475	0.000906891
GO:0097581	lamellipodium organization	38/5895	0.000102475	0.000906891
GO:0071398	cellular response to fatty acid	20/5895	0.000102491	0.000906891
GO:1903708	positive regulation of hemopoiesis	82/5895	0.000104577	0.000924059
GO:0008299	isoprenoid biosynthetic process	18/5895	0.000105156	0.00092706
GO:0032231	regulation of actin filament bundle assembly	46/5895	0.000105211	0.00092706

GO:0007229	integrin-mediated signaling pathway	41/5895	0.00010621	0.000933258
GO:0021954	central nervous system neuron development	41/5895	0.00010621	0.000933258
GO:0001837	epithelial to mesenchymal transition	57/5895	0.000107189	0.000940553
GO:0019058	viral life cycle	81/5895	0.000109554	0.000959963
GO:0032350	regulation of hormone metabolic process	23/5895	0.000111397	0.00097476
GO:0043903	regulation of symbiotic process	80/5895	0.000114746	0.001002675
GO:0045600	positive regulation of fat cell differentiation	32/5895	0.000116375	0.001014105
GO:1904377	positive regulation of protein localization to cell periphery	32/5895	0.000116375	0.001014105
GO:0031348	negative regulation of defense response	83/5895	0.000118485	0.001031066
GO:0002042	cell migration involved in sprouting angiogenesis	27/5895	0.000118806	0.001031414
GO:0061136	regulation of proteasomal protein catabolic process	71/5895	0.000118852	0.001031414
GO:0051260	protein homooligomerization	66/5895	0.000119848	0.001038629
GO:0048762	mesenchymal cell differentiation	82/5895	0.000124233	0.00107515
GO:0048659	smooth muscle cell proliferation	65/5895	0.000124541	0.001076342
GO:0006739	NADP metabolic process	21/5895	0.000126429	0.001091165
GO:0021953	central nervous system neuron differentiation	85/5895	0.000127409	0.00109717
GO:0021932	hindbrain radial glia guided cell migration	Nov-95	0.000127473	0.00109717
GO:0120163	negative regulation of cold-induced thermogenesis	24/5895	0.000128502	0.001104298

GO:0006479	protein methylation	69/5895	0.000129174	0.001104298
GO:0008213	protein alkylation	69/5895	0.000129174	0.001104298
GO:0051100	negative regulation of binding	64/5895	0.00012935	0.001104298
GO:0009100	glycoprotein metabolic process	114/5895	0.00012948	0.001104298
GO:0030282	bone mineralization	49/5895	0.000129864	0.001104298
GO:0005977	glycogen metabolic process	35/5895	0.000130226	0.001104298
GO:0006073	cellular glucan metabolic process	35/5895	0.000130226	0.001104298
GO:0044042	glucan metabolic process	35/5895	0.000130226	0.001104298
GO:0061180	mammary gland epithelium development	35/5895	0.000130226	0.001104298
GO:1903036	positive regulation of response to wounding	35/5895	0.000130226	0.001104298
GO:0048814	regulation of dendrite morphogenesis	48/5895	0.000132148	0.001117587
GO:1901890	positive regulation of cell junction assembly	48/5895	0.000132148	0.001117587
GO:0033157	regulation of intracellular protein transport	84/5895	0.000133713	0.001129312
GO:0030879	mammary gland development	63/5895	0.000134272	0.001132515
GO:0003151	outflow tract morphogenesis	37/5895	0.000136023	0.001145754
GO:0032147	activation of protein kinase activity	87/5895	0.000136282	0.001146405
GO:0032386	regulation of intracellular transport	113/5895	0.000136533	0.001146988
GO:0006568	tryptophan metabolic process	Sep-95	0.000137524	0.001151604

GO:0006586	indolalkylamine metabolic process	Sep-95	0.000137524	0.001151604
GO:0060840	artery development	45/5895	0.000137637	0.001151604
GO:0072593	reactive oxygen species metabolic process	102/5895	0.000137813	0.001151604
GO:0001819	positive regulation of cytokine production	148/5895	0.000138411	0.001152462
GO:0010635	regulation of mitochondrial fusion	Oct-95	0.000138463	0.001152462
GO:0071281	cellular response to iron ion	Oct-95	0.000138463	0.001152462
GO:0090140	regulation of mitochondrial fission	17/5895	0.000139193	0.001157005
GO:1902903	regulation of supramolecular fiber organization	125/5895	0.00014014	0.001163343
GO:0031016	pancreas development	41/5895	0.000140508	0.001164865
GO:0072511	divalent inorganic cation transport	154/5895	0.000141611	0.001172474
GO:0001890	placenta development	66/5895	0.000145934	0.001206681
GO:0048008	platelet-derived growth factor receptor signaling pathway	30/5895	0.00014814	0.001223319
GO:0070838	divalent metal ion transport	153/5895	0.000149414	0.001232229
GO:0045931	positive regulation of mitotic cell cycle	65/5895	0.00015186	0.001250759
GO:0032368	regulation of lipid transport	52/5895	0.000153947	0.001266301
GO:0051101	regulation of DNA binding	51/5895	0.000157835	0.001296591
GO:0030514	negative regulation of BMP signaling pathway	26/5895	0.000160291	0.001313342
GO:0031103	axon regeneration	26/5895	0.000160291	0.001313342

GO:1903052	positive regulation of proteolysis involved in cellular protein catabolic process	50/5895	0.000161605	0.001322275
GO:0044273	sulfur compound catabolic process	14/5895	0.0001618	0.001322275
GO:0030098	lymphocyte differentiation	136/5895	0.000164172	0.001339925
GO:0032956	regulation of actin cytoskeleton organization	122/5895	0.000164607	0.001341735
GO:0045739	positive regulation of DNA repair	33/5895	0.000168645	0.00137134
GO:0018107	peptidyl-threonine phosphorylation	48/5895	0.000168673	0.00137134
GO:0032409	regulation of transporter activity	98/5895	0.000169644	0.001377459
GO:2000781	positive regulation of double-strand break repair	20/5895	0.000170241	0.001380526
GO:0001818	negative regulation of cytokine production	92/5895	0.000170722	0.001382649
GO:0051480	regulation of cytosolic calcium ion concentration	126/5895	0.000171481	0.001386887
GO:0018958	phenol-containing compound metabolic process	47/5895	0.000171905	0.001386887
GO:0031398	positive regulation of protein ubiquitination	47/5895	0.000171905	0.001386887
GO:0061351	neural precursor cell proliferation	71/5895	0.000172419	0.001389262
GO:0045923	positive regulation of fatty acid metabolic process	23/5895	0.000173615	0.001397111
GO:0055021	regulation of cardiac muscle tissue growth	35/5895	0.000177449	0.001426144
GO:0050792	regulation of viral process	74/5895	0.000180361	0.001447006
GO:0032370	positive regulation of lipid transport	36/5895	0.000180503	0.001447006
GO:0050772	positive regulation of axonogenesis	43/5895	0.000181896	0.001454479

GO:0090263	positive regulation of canonical Wnt signaling pathway	43/5895	0.000181896	0.001454479
GO:1902882	regulation of response to oxidative stress	42/5895	0.000183435	0.001464216
GO:0048745	smooth muscle tissue development	16/5895	0.0001838 1	0.001464216
GO:0060045	positive regulation of cardiac muscle cell proliferation	16/5895	0.0001838 1	0.001464216
GO:0001570	vasculogenesis	41/5895	0.000184488	0.001465913
GO:1905037	autophagosome organization	41/5895	0.000184488	0.001465913
GO:0010717	regulation of epithelial to mesenchymal transition	40/5895	0.000185002	0.001468146
GO:0045995	regulation of embryonic development	53/5895	0.000187749	0.001488079
GO:0090257	regulation of muscle system process	87/5895	0.000188019	0.001488345
GO:0050817	coagulation	64/5895	0.000192192	0.001517568
GO:0071356	cellular response to tumor necrosis factor	64/5895	0.000192192	0.001517568
GO:0018022	peptidyl-lysine methylation	52/5895	0.000193211	0.001523708
GO:0003382	epithelial cell morphogenesis	24/5895	0.000195748	0.001541791
GO:0006900	vesicle budding from membrane	29/5895	0.000198966	0.001563234
GO:1903078	positive regulation of protein localization to plasma membrane	29/5895	0.000198966	0.001563234
GO:0007596	blood coagulation	63/5895	0.000200086	0.001570077
GO:0006984	ER-nucleus signaling pathway	21/5895	0.000203037	0.001587314
GO:0034105	positive regulation of tissue remodeling	21/5895	0.000203037	0.001587314

GO:0097009	energy homeostasis	21/5895	0.000203037	0.001587314
GO:0043123	positive regulation of I-kappaB kinase/NF-kappaB signaling	50/5895	0.000203908	0.001588115
GO:0003206	cardiac chamber morphogenesis	57/5895	0.000204805	0.001588115
GO:0008209	androgen metabolic process	13/5895	0.000205154	0.001588115
GO:0009081	branched-chain amino acid metabolic process	13/5895	0.000205154	0.001588115
GO:0010875	positive regulation of cholesterol efflux	13/5895	0.000205154	0.001588115
GO:0019363	pyridine nucleotide biosynthetic process	13/5895	0.000205154	0.001588115
GO:0021535	cell migration in hindbrain	13/5895	0.000205154	0.001588115
GO:0090030	regulation of steroid hormone biosynthetic process	13/5895	0.000205154	0.001588115
GO:0015980	energy derivation by oxidation of organic compounds	88/5895	0.000209227	0.001617665
GO:0007626	locomotory behavior	91/5895	0.000209486	0.001617682
GO:0097193	intrinsic apoptotic signaling pathway	102/5895	0.000212509	0.001639023
GO:0000187	activation of MAPK activity	48/5895	0.000214085	0.001647151
GO:2000278	regulation of DNA biosynthetic process	48/5895	0.000214085	0.001647151
GO:0031663	lipopolysaccharide-mediated signaling pathway	25/5895	0.000216387	0.001662829
GO:0030032	lamellipodium assembly	31/5895	0.000217805	0.001669663
GO:0033692	cellular polysaccharide biosynthetic process	31/5895	0.000217805	0.001669663
GO:0016050	vesicle organization	96/5895	0.000218192	0.001670599

GO:0009636	response to toxic substance	47/5895	0.000218883	0.001671831
GO:0034968	histone lysine methylation	47/5895	0.000218883	0.001671831
GO:0021543	pallium development	60/5895	0.000225109	0.001715487
GO:0055081	anion homeostasis	32/5895	0.000225142	0.001715487
GO:2001020	regulation of response to DNA damage stimulus	80/5895	0.000226405	0.00172303
GO:2001022	positive regulation of response to DNA damage stimulus	45/5895	0.00022767	0.001730568
GO:1905898	positive regulation of response to endoplasmic reticulum stress	19/5895	0.000229309	0.001740925
GO:0038083	peptidyl-tyrosine autophosphorylation	22/5895	0.000234731	0.00177985
GO:0043488	regulation of mRNA stability	43/5895	0.000235028	0.00177985
GO:1900024	regulation of substrate adhesion-dependent cell spreading	26/5895	0.000235282	0.00177985
GO:0045453	bone resorption	34/5895	0.000235897	0.001782367
GO:2001237	negative regulation of extrinsic apoptotic signaling pathway	42/5895	0.000238022	0.001796272
GO:0034637	cellular carbohydrate biosynthetic process	35/5895	0.000239454	0.001804919
GO:0051492	regulation of stress fiber assembly	40/5895	0.000242305	0.001824225
GO:2001057	reactive nitrogen species metabolic process	37/5895	0.000243328	0.001828447
GO:0000045	autophagosome assembly	39/5895	0.000243445	0.001828447
GO:0006767	water-soluble vitamin metabolic process	17/5895	0.000245731	0.001841231
GO:0019433	triglyceride catabolic process	17/5895	0.000245731	0.001841231

GO:0015672	monovalent inorganic cation transport	158/5895	0.000247221	0.001850201
GO:0043536	positive regulation of blood vessel endothelial cell migration	27/5895	0.00025228	0.001883595
GO:0046622	positive regulation of organ growth	27/5895	0.00025228	0.001883595
GO:0042448	progesterone metabolic process	Dec-95	0.00025649	0.001912763
GO:0046474	glycerophospholipid biosynthetic process	49/5895	0.000263163	0.001960204
GO:0007163	establishment or maintenance of cell polarity	80/5895	0.000266665	0.001983952
GO:0090303	positive regulation of wound healing	28/5895	0.000267303	0.001986354
GO:0010821	regulation of mitochondrion organization	55/5895	0.000270804	0.002010001
GO:0002683	negative regulation of immune system process	150/5895	0.000273975	0.002031151
GO:0150116	regulation of cell-substrate junction organization	29/5895	0.000280331	0.002075833
GO:0019827	stem cell population maintenance	64/5895	0.000281592	0.002082724
GO:0008380	RNA splicing	128/5895	0.000284975	0.002105281
GO:0016482	cytosolic transport	59/5895	0.000285643	0.002107747
GO:0055123	digestive system development	53/5895	0.000290143	0.002138446
GO:1905953	negative regulation of lipid localization	24/5895	0.000292299	0.002151825
GO:0033044	regulation of chromosome organization	123/5895	0.000292833	0.002153246
GO:0007599	hemostasis	63/5895	0.000293926	0.002158762
GO:0048661	positive regulation of smooth muscle cell proliferation	44/5895	0.000296126	0.002172395

GO:0016239	positive regulation of macroautophagy	31/5895	0.000300551	0.002199738
GO:0048678	response to axon injury	31/5895	0.000300551	0.002199738
GO:0002221	pattern recognition receptor signaling pathway	57/5895	0.000308795	0.002254849
GO:0034101	erythrocyte homeostasis	57/5895	0.000308795	0.002254849
GO:0097061	dendritic spine organization	41/5895	0.000311228	0.002269982
GO:0046626	regulation of insulin receptor signaling pathway	33/5895	0.00031352	0.002279745
GO:0006103	2-oxoglutarate metabolic process	Nov-95	0.000314381	0.002279745
GO:0055089	fatty acid homeostasis	Nov-95	0.000314381	0.002279745
GO:0060391	positive regulation of SMAD protein signal transduction	Nov-95	0.000314381	0.002279745
GO:0098696	regulation of neurotransmitter receptor localization to postsynaptic specialization membrane	Nov-95	0.000314381	0.002279745
GO:0010866	regulation of triglyceride biosynthetic process	14/5895	0.000317314	0.002279745
GO:0034383	low-density lipoprotein particle clearance	14/5895	0.000317314	0.002279745
GO:0045980	negative regulation of nucleotide metabolic process	14/5895	0.000317314	0.002279745
GO:2000345	regulation of hepatocyte proliferation	14/5895	0.000317314	0.002279745
GO:0018023	peptidyl-lysine trimethylation	25/5895	0.00031738	0.002279745
GO:1904036	negative regulation of epithelial cell apoptotic process	25/5895	0.00031738	0.002279745
GO:0019229	regulation of vasoconstriction	34/5895	0.000317552	0.002279745
GO:0008207	C21-steroid hormone metabolic process	21/5895	0.000317607	0.002279745

GO:0033077	T cell differentiation in thymus	39/5895	0.000318027	0.002279745
GO:0045445	myoblast differentiation	39/5895	0.000318027	0.002279745
GO:0018210	peptidyl-threonine modification	50/5895	0.000319674	0.002279745
GO:0060996	dendritic spine development	50/5895	0.000319674	0.002279745
GO:0006140	regulation of nucleotide metabolic process	35/5895	0.000320109	0.002279745
GO:1904589	regulation of protein import	35/5895	0.000320109	0.002279745
GO:1900076	regulation of cellular response to insulin stimulus	38/5895	0.000320154	0.002279745
GO:1903035	negative regulation of response to wounding	38/5895	0.000320154	0.002279745
GO:0034390	smooth muscle cell apoptotic process	16/5895	0.000328464	0.002333648
GO:0034391	regulation of smooth muscle cell apoptotic process	16/5895	0.000328464	0.002333648
GO:0051153	regulation of striated muscle cell differentiation	49/5895	0.000329513	0.002338471
GO:0030010	establishment of cell polarity	55/5895	0.000333035	0.002360806
GO:0046620	regulation of organ growth	48/5895	0.000339276	0.002400026
GO:0006826	iron ion transport	26/5895	0.000339709	0.002400026
GO:0050775	positive regulation of dendrite morphogenesis	26/5895	0.000339709	0.002400026
GO:0050728	negative regulation of inflammatory response	59/5895	0.00034753	0.002452535
GO:0050919	negative chemotaxis	22/5895	0.000358065	0.002523236
GO:0019751	polyol metabolic process	46/5895	0.000358348	0.002523236

GO:0048839	inner ear development	77/5895	0.000364095	0.00256084
GO:0048679	regulation of axon regeneration	19/5895	0.000371047	0.002604304
GO:0001936	regulation of endothelial cell proliferation	52/5895	0.0003711	0.002604304
GO:0046500	S-adenosylmethionine metabolic process	Oct-95	0.000374281	0.002620796
GO:0046886	positive regulation of hormone biosynthetic process	Oct-95	0.000374281	0.002620796
GO:0014896	muscle hypertrophy	44/5895	0.000376377	0.002632555
GO:0031623	receptor internalization	51/5895	0.000384134	0.002680864
GO:0032434	regulation of proteasomal ubiquitin-dependent protein catabolic process	51/5895	0.000384134	0.002680864
GO:0070988	demethylation	29/5895	0.000389861	0.002717826
GO:0003300	cardiac muscle hypertrophy	42/5895	0.000392726	0.002734776
GO:0010639	negative regulation of organelle organization	137/5895	0.000394681	0.00274233
GO:0003179	heart valve morphogenesis	23/5895	0.000395115	0.00274233
GO:0045911	positive regulation of DNA recombination	23/5895	0.000395115	0.00274233
GO:0061013	regulation of mRNA catabolic process	50/5895	0.000397281	0.002754335
GO:0032412	regulation of ion transmembrane transporter activity	90/5895	0.000400138	0.002769836
GO:0043062	extracellular structure organization	103/5895	0.000400395	0.002769836
GO:0006071	glycerol metabolic process	13/5895	0.000413876	0.002853707
GO:0009110	vitamin biosynthetic process	13/5895	0.000413876	0.002853707

GO:00312<br>90	retinal ganglion cell axon guidance	13/5895	0.0004138<br>76	0.0028537<br>07
GO:00020<br>92	positive regulation of receptor internalization	17/5895	0.0004164<br>29	0.0028557<br>06
GO:00064<br>82	protein demethylation	17/5895	0.0004164<br>29	0.0028557<br>06
GO:00082<br>14	protein dealkylation	17/5895	0.0004164<br>29	0.0028557<br>06
GO:00323<br>65	intracellular lipid transport	17/5895	0.0004164<br>29	0.0028557<br>06
GO:00432<br>76	anoikis	17/5895	0.0004164<br>29	0.0028557<br>06
GO:00301<br>93	regulation of blood coagulation	35/5895	0.0004240<br>7	0.0029049<br>5
GO:00328<br>85	regulation of polysaccharide biosynthetic process	20/5895	0.0004301<br>1	0.0029304<br>17
GO:00463<br>20	regulation of fatty acid oxidation	20/5895	0.0004301<br>1	0.0029304<br>17
GO:00602<br>51	regulation of glial cell proliferation	20/5895	0.0004301<br>1	0.0029304<br>17
GO:00725<br>23	purine-containing compound catabolic process	20/5895	0.0004301<br>1	0.0029304<br>17
GO:00900<br>49	regulation of cell migration involved in sprouting angiogenesis	20/5895	0.0004301<br>1	0.0029304<br>17
GO:00303<br>25	adrenal gland development	15/5895	0.0004385<br>78	0.0029848<br>85
GO:00302<br>18	erythrocyte differentiation	53/5895	0.0004402<br>98	0.0029933<br>65
GO:00525<br>47	regulation of peptidase activity	137/589<br>5	0.0004411<br>28	0.0029957<br>77
GO:00060<br>00	fructose metabolic process	Aug-95	0.0004490<br>05	0.0030329<br>49
GO:00066<br>51	diacylglycerol biosynthetic process	Aug-95	0.0004490<br>05	0.0030329<br>49
GO:00107<br>45	negative regulation of macrophage derived foam cell differentiation	Aug-95	0.0004490<br>05	0.0030329<br>49

GO:0060982	coronary artery morphogenesis	Aug-95	0.000449005	0.003032949
GO:1900103	positive regulation of endoplasmic reticulum unfolded protein response	Aug-95	0.000449005	0.003032949
GO:0043487	regulation of RNA stability	46/5895	0.000449997	0.003036397
GO:0022898	regulation of transmembrane transporter activity	93/5895	0.000453957	0.003059845
GO:0050764	regulation of phagocytosis	45/5895	0.000462892	0.003116739
GO:1902905	positive regulation of supramolecular fiber organization	72/5895	0.00046677	0.0031395
GO:0051604	protein maturation	100/5895	0.000470442	0.003160832
GO:0010469	regulation of signaling receptor activity	51/5895	0.00047406	0.00317837
GO:0099175	regulation of postsynapse organization	51/5895	0.00047406	0.00317837
GO:0006275	regulation of DNA replication	44/5895	0.000475515	0.003184742
GO:0055023	positive regulation of cardiac muscle tissue growth	21/5895	0.000484869	0.003243942
GO:0014897	striated muscle hypertrophy	43/5895	0.000487772	0.003256458
GO:0110020	regulation of actomyosin structure organization	43/5895	0.000487772	0.003256458
GO:0043543	protein acylation	83/5895	0.000498649	0.003320932
GO:0048863	stem cell differentiation	83/5895	0.000498649	0.003320932
GO:0045778	positive regulation of ossification	42/5895	0.000499553	0.003320932
GO:0033673	negative regulation of kinase activity	80/5895	0.000501007	0.003320932
GO:0000096	sulfur amino acid metabolic process	18/5895	0.000502167	0.003320932

GO:0044275	cellular carbohydrate catabolic process	18/5895	0.000502167	0.003320932
GO:0045070	positive regulation of viral genome replication	18/5895	0.000502167	0.003320932
GO:0048194	Golgi vesicle budding	18/5895	0.000502167	0.003320932
GO:1903146	regulation of autophagy of mitochondrion	18/5895	0.000502167	0.003320932
GO:0052548	regulation of endopeptidase activity	115/5895	0.000509696	0.003367041
GO:0071456	cellular response to hypoxia	41/5895	0.00051074	0.003367041
GO:2000060	positive regulation of ubiquitin-dependent protein catabolic process	41/5895	0.00051074	0.003367041
GO:0090068	positive regulation of cell cycle process	88/5895	0.000513716	0.003383128
GO:0060395	SMAD protein signal transduction	40/5895	0.000521199	0.003420929
GO:0006081	cellular aldehyde metabolic process	28/5895	0.000521625	0.003420929
GO:0031102	neuron projection regeneration	28/5895	0.000521625	0.003420929
GO:0046850	regulation of bone remodeling	28/5895	0.000521625	0.003420929
GO:0006814	sodium ion transport	82/5895	0.000525368	0.003441894
GO:0009132	nucleoside diphosphate metabolic process	48/5895	0.000526489	0.003445665
GO:0001569	branching involved in blood vessel morphogenesis	22/5895	0.000534496	0.003478584
GO:0050918	positive chemotaxis	22/5895	0.000534496	0.003478584
GO:0090398	cellular senescence	29/5895	0.000535482	0.003478584
GO:0006656	phosphatidylcholine biosynthetic process	Dec-95	0.00053593	0.003478584

GO:0033145	positive regulation of intracellular steroid hormone receptor signaling pathway	Dec-95	0.00053593	0.003478584
GO:0042730	fibrinolysis	Dec-95	0.00053593	0.003478584
GO:0044539	long-chain fatty acid import into cell	Dec-95	0.00053593	0.003478584
GO:0140354	lipid import into cell	Dec-95	0.00053593	0.003478584
GO:0055007	cardiac muscle cell differentiation	53/5895	0.000538829	0.003493807
GO:0045861	negative regulation of proteolysis	116/5895	0.000544849	0.003529195
GO:0015800	acidic amino acid transport	30/5895	0.000545965	0.003529195
GO:1902369	negative regulation of RNA catabolic process	30/5895	0.000545965	0.003529195
GO:0045786	negative regulation of cell cycle	145/5895	0.000549027	0.003545355
GO:0030198	extracellular matrix organization	102/5895	0.000549661	0.003545822
GO:0048708	astrocyte differentiation	36/5895	0.000552588	0.003552443
GO:0050818	regulation of coagulation	36/5895	0.000552588	0.003552443
GO:0001885	endothelial cell development	31/5895	0.000553331	0.003552443
GO:0007492	endoderm development	31/5895	0.000553331	0.003552443
GO:0099504	synaptic vesicle cycle	81/5895	0.000553503	0.003552443
GO:1900046	regulation of hemostasis	35/5895	0.0005569	0.003570614
GO:1900542	regulation of purine nucleotide metabolic process	34/5895	0.000559375	0.003581752
GO:0033143	regulation of intracellular steroid hormone receptor signaling pathway	33/5895	0.000559773	0.003581752

GO:1903037	regulation of leukocyte cell-cell adhesion	104/5895	0.000561291	0.003587826
GO:0050921	positive regulation of chemotaxis	56/5895	0.000579048	0.003696482
GO:2000379	positive regulation of reactive oxygen species metabolic process	45/5895	0.00057971	0.003696482
GO:0002053	positive regulation of mesenchymal cell proliferation	19/5895	0.000583008	0.003696482
GO:0046329	negative regulation of JNK cascade	19/5895	0.000583008	0.003696482
GO:0070873	regulation of glycogen metabolic process	19/5895	0.000583008	0.003696482
GO:1990748	cellular detoxification	19/5895	0.000583008	0.003696482
GO:0019400	alditol metabolic process	14/5895	0.000584734	0.003696482
GO:0036314	response to sterol	14/5895	0.000584734	0.003696482
GO:0046823	negative regulation of nucleocytoplasmic transport	14/5895	0.000584734	0.003696482
GO:0048670	regulation of collateral sprouting	14/5895	0.000584734	0.003696482
GO:0072525	pyridine-containing compound biosynthetic process	14/5895	0.000584734	0.003696482
GO:1904062	regulation of cation transmembrane transport	119/5895	0.000587401	0.003709626
GO:0006403	RNA localization	66/5895	0.000626448	0.003952257
GO:0042157	lipoprotein metabolic process	49/5895	0.000627673	0.00395603
GO:0022408	negative regulation of cell-cell adhesion	69/5895	0.000638599	0.004020878
GO:0051289	protein homotetramerization	25/5895	0.000649324	0.004080264
GO:0055025	positive regulation of cardiac muscle tissue development	25/5895	0.000649324	0.004080264

GO:0071453	cellular response to oxygen levels	53/5895	0.000656593	0.00412141
GO:1904645	response to amyloid-beta	20/5895	0.000657183	0.00412141
GO:0046031	ADP metabolic process	40/5895	0.000663552	0.00415724
GO:0043489	RNA stabilization	26/5895	0.000676282	0.00422861
GO:1902373	negative regulation of mRNA catabolic process	26/5895	0.000676282	0.00422861
GO:0060998	regulation of dendritic spine development	39/5895	0.000678411	0.00423771
GO:0006099	tricarboxylic acid cycle	17/5895	0.000680197	0.0042446
GO:0010878	cholesterol storage	Nov-95	0.000687001	0.00427444
GO:0019359	nicotinamide nucleotide biosynthetic process	Nov-95	0.000687001	0.00427444
GO:0035459	vesicle cargo loading	Nov-95	0.000687001	0.00427444
GO:0048260	positive regulation of receptor-mediated endocytosis	27/5895	0.000697948	0.00432672
GO:0051893	regulation of focal adhesion assembly	27/5895	0.000697948	0.00432672
GO:0090109	regulation of cell-substrate junction assembly	27/5895	0.000697948	0.00432672
GO:2000177	regulation of neural precursor cell proliferation	46/5895	0.000698146	0.00432672
GO:0046928	regulation of neurotransmitter secretion	51/5895	0.000712153	0.00440919
GO:0070482	response to oxygen levels	97/5895	0.000720187	0.00445456
GO:0019079	viral genome replication	45/5895	0.000722014	0.00446149
GO:0097484	dendrite extension	21/5895	0.000723637	0.00446714

GO:0051384	response to glucocorticoid	35/5895	0.000725177	0.00447228
GO:0051236	establishment of RNA localization	59/5895	0.000733765	0.004520824
GO:0034394	protein localization to cell surface	30/5895	0.000734746	0.00452245
GO:0060042	retina morphogenesis in camera-type eye	33/5895	0.000737401	0.004534371
GO:0042306	regulation of protein import into nucleus	32/5895	0.000739668	0.004543882
GO:0048640	negative regulation of developmental growth	50/5895	0.000741024	0.004547781
GO:0042908	xenobiotic transport	15/5895	0.000757037	0.004641545
GO:0050657	nucleic acid transport	58/5895	0.000768352	0.004701778
GO:0050658	RNA transport	58/5895	0.000768352	0.004701778
GO:0051897	positive regulation of protein kinase B signaling	43/5895	0.000769728	0.004705633
GO:0015012	heparan sulfate proteoglycan biosynthetic process	13/5895	0.000777985	0.004737749
GO:0043574	peroxisomal transport	13/5895	0.000777985	0.004737749
GO:1900101	regulation of endoplasmic reticulum unfolded protein response	13/5895	0.000777985	0.004737749
GO:1900543	negative regulation of purine nucleotide metabolic process	13/5895	0.000777985	0.004737749
GO:0033344	cholesterol efflux	22/5895	0.000781859	0.004756748
GO:1900181	negative regulation of protein localization to nucleus	18/5895	0.000791075	0.004808181
GO:0014812	muscle cell migration	42/5895	0.000793325	0.004817214
GO:1903532	positive regulation of secretion by cell	126/5895	0.000796365	0.004831028

GO:0060401	cytosolic calcium ion transport	64/5895	0.000817855	0.004956623
GO:0048255	mRNA stabilization	23/5895	0.000831738	0.005031096
GO:0090342	regulation of cell aging	23/5895	0.000831738	0.005031096
GO:0034249	negative regulation of cellular amide metabolic process	60/5895	0.000836024	0.005052178
GO:0006816	calcium ion transport	135/5895	0.000841923	0.005082959
GO:0001894	tissue homeostasis	89/5895	0.000850556	0.005130172
GO:0010660	regulation of muscle cell apoptotic process	39/5895	0.000861169	0.005184271
GO:0099072	regulation of postsynaptic membrane neurotransmitter receptor levels	39/5895	0.000861169	0.005184271
GO:0009070	serine family amino acid biosynthetic process	Oct-95	0.000867897	0.005195029
GO:0010885	regulation of cholesterol storage	Oct-95	0.000867897	0.005195029
GO:0014857	regulation of skeletal muscle cell proliferation	Oct-95	0.000867897	0.005195029
GO:0016114	terpenoid biosynthetic process	Oct-95	0.000867897	0.005195029
GO:0072350	tricarboxylic acid metabolic process	Oct-95	0.000867897	0.005195029
GO:2000811	negative regulation of anoikis	Oct-95	0.000867897	0.005195029
GO:0007568	aging	86/5895	0.000871947	0.005209386
GO:1901215	negative regulation of neuron death	86/5895	0.000871947	0.005209386
GO:0021915	neural tube development	69/5895	0.0008821	0.005260083
GO:0030705	cytoskeleton-dependent intracellular transport	69/5895	0.0008821	0.005260083

GO:1900407	regulation of cellular response to oxidative stress	37/5895	0.000901899	0.005369031
GO:0070304	positive regulation of stress-activated protein kinase signaling cascade	62/5895	0.000902072	0.005369031
GO:0061001	regulation of dendritic spine morphogenesis	25/5895	0.000907337	0.005395276
GO:1903312	negative regulation of mRNA metabolic process	36/5895	0.000920159	0.005466368
GO:0010874	regulation of cholesterol efflux	16/5895	0.000922193	0.005468154
GO:0060317	cardiac epithelial to mesenchymal transition	16/5895	0.000922193	0.005468154
GO:0030516	regulation of axon extension	44/5895	0.00092639	0.005487884
GO:1903902	positive regulation of viral life cycle	26/5895	0.0009339	0.005527179
GO:0014065	phosphatidylinositol 3-kinase signaling	49/5895	0.000941602	0.00556754
GO:0030500	regulation of bone mineralization	34/5895	0.000950904	0.005617276
GO:0006893	Golgi to plasma membrane transport	27/5895	0.000953686	0.005617931
GO:0007584	response to nutrient	27/5895	0.000953686	0.005617931
GO:0010812	negative regulation of cell-substrate adhesion	27/5895	0.000953686	0.005617931
GO:0017015	regulation of transforming growth factor beta receptor signaling pathway	43/5895	0.000958551	0.005641322
GO:0006879	cellular iron ion homeostasis	28/5895	0.000967282	0.005682109
GO:1903510	mucopolysaccharide metabolic process	28/5895	0.000967282	0.005682109
GO:0060997	dendritic spine morphogenesis	31/5895	0.000976821	0.005732806
GO:0030048	actin filament-based movement	48/5895	0.000980395	0.005743091

GO:0036294	cellular response to decreased oxygen levels	48/5895	0.000980395	0.005743091
GO:1903426	regulation of reactive oxygen species biosynthetic process	42/5895	0.000990759	0.005798421
GO:0051258	protein polymerization	98/5895	0.001001278	0.005854554
GO:0010921	regulation of phosphatase activity	47/5895	0.001020134	0.005925671
GO:0048565	digestive tract development	47/5895	0.001020134	0.005925671
GO:0000423	mitophagy	14/5895	0.001020955	0.005925671
GO:0006706	steroid catabolic process	14/5895	0.001020955	0.005925671
GO:0010884	positive regulation of lipid storage	14/5895	0.001020955	0.005925671
GO:0042430	indole-containing compound metabolic process	14/5895	0.001020955	0.005925671
GO:0046426	negative regulation of receptor signaling pathway via JAK-STAT	14/5895	0.001020955	0.005925671
GO:0060004	reflex	14/5895	0.001020955	0.005925671
GO:0009135	purine nucleoside diphosphate metabolic process	41/5895	0.00102285	0.005925768
GO:0009179	purine ribonucleoside diphosphate metabolic process	41/5895	0.00102285	0.005925768
GO:0007159	leukocyte cell-cell adhesion	113/5895	0.001024902	0.005932208
GO:0034655	nucleobase-containing compound catabolic process	115/5895	0.001030825	0.005952
GO:0009067	aspartate family amino acid biosynthetic process	Dec-95	0.001032095	0.005952
GO:0010888	negative regulation of lipid storage	Dec-95	0.001032095	0.005952
GO:2000773	negative regulation of cellular senescence	Dec-95	0.001032095	0.005952

GO:0005978	glycogen biosynthetic process	21/5895	0.00105742	0.006064784
GO:0009250	glucan biosynthetic process	21/5895	0.00105742	0.006064784
GO:0051898	negative regulation of protein kinase B signaling	21/5895	0.00105742	0.006064784
GO:0097237	cellular response to toxic substance	21/5895	0.00105742	0.006064784
GO:1902895	positive regulation of pri-miRNA transcription by RNA polymerase II	21/5895	0.00105742	0.006064784
GO:1903523	negative regulation of blood circulation	21/5895	0.00105742	0.006064784
GO:0006544	glycine metabolic process	Sep-95	0.001072558	0.006123771
GO:0006895	Golgi to endosome transport	Sep-95	0.001072558	0.006123771
GO:0034497	protein localization to phagophore assembly site	Sep-95	0.001072558	0.006123771
GO:0055091	phospholipid homeostasis	Sep-95	0.001072558	0.006123771
GO:0071679	commissural neuron axon guidance	Sep-95	0.001072558	0.006123771
GO:0009101	glycoprotein biosynthetic process	92/5895	0.001075469	0.00613484
GO:0097305	response to alcohol	74/5895	0.00107748	0.006140761
GO:0051341	regulation of oxidoreductase activity	38/5895	0.001116382	0.006356727
GO:0003197	endocardial cushion development	22/5895	0.001122194	0.006372566
GO:0010718	positive regulation of epithelial to mesenchymal transition	22/5895	0.001122194	0.006372566
GO:0060964	regulation of gene silencing by miRNA	22/5895	0.001122194	0.006372566
GO:0050714	positive regulation of protein secretion	70/5895	0.001143784	0.006480621

GO:0001892	embryonic placenta development	44/5895	0.001144304	0.006480621
GO:0060078	regulation of postsynaptic membrane potential	44/5895	0.001144304	0.006480621
GO:0055072	iron ion homeostasis	37/5895	0.00114581	0.00648333
GO:0060147	regulation of posttranscriptional gene silencing	23/5895	0.001175348	0.006638557
GO:0060966	regulation of gene silencing by RNA	23/5895	0.001175348	0.006638557
GO:0016241	regulation of macroautophagy	43/5895	0.001187013	0.006686484
GO:0035601	protein deacylation	43/5895	0.001187013	0.006686484
GO:0098732	macromolecule deacylation	43/5895	0.001187013	0.006686484
GO:0048009	insulin-like growth factor receptor signaling pathway	18/5895	0.001211103	0.006816102
GO:0009395	phospholipid catabolic process	24/5895	0.001217558	0.006834142
GO:0050435	amyloid-beta metabolic process	24/5895	0.001217558	0.006834142
GO:0051055	negative regulation of lipid biosynthetic process	24/5895	0.001217558	0.006834142
GO:0009185	ribonucleoside diphosphate metabolic process	42/5895	0.001230163	0.006898752
GO:0043500	muscle adaptation	47/5895	0.001245514	0.006932396
GO:0050688	regulation of defense response to virus	33/5895	0.001245813	0.006932396
GO:0051651	maintenance of location in cell	74/5895	0.001248382	0.006932396
GO:0009119	ribonucleoside metabolic process	25/5895	0.001249642	0.006932396
GO:0050873	brown fat cell differentiation	25/5895	0.001249642	0.006932396

GO:0002089	lens morphogenesis in camera-type eye	15/5895	0.001251545	0.006932396
GO:0016577	histone demethylation	15/5895	0.001251545	0.006932396
GO:0045663	positive regulation of myoblast differentiation	15/5895	0.001251545	0.006932396
GO:0048596	embryonic camera-type eye morphogenesis	15/5895	0.001251545	0.006932396
GO:0060390	regulation of SMAD protein signal transduction	15/5895	0.001251545	0.006932396
GO:0070076	histone lysine demethylation	15/5895	0.001251545	0.006932396
GO:0071402	cellular response to lipoprotein particle stimulus	15/5895	0.001251545	0.006932396
GO:0090077	foam cell differentiation	15/5895	0.001251545	0.006932396
GO:1903671	negative regulation of sprouting angiogenesis	15/5895	0.001251545	0.006932396
GO:0010822	positive regulation of mitochondrion organization	32/5895	0.001264197	0.006990201
GO:0048844	artery morphogenesis	32/5895	0.001264197	0.006990201
GO:0000082	G1/S transition of mitotic cell cycle	65/5895	0.001269075	0.007007935
GO:0060443	mammary gland morphogenesis	26/5895	0.001272484	0.007007935
GO:0006107	oxaloacetate metabolic process	Aug-95	0.001279623	0.007007935
GO:0010898	positive regulation of triglyceride catabolic process	Aug-95	0.001279623	0.007007935
GO:0019627	urea metabolic process	Aug-95	0.001279623	0.007007935
GO:0035336	long-chain fatty-acyl-CoA metabolic process	Aug-95	0.001279623	0.007007935
GO:0038180	nerve growth factor signaling pathway	Aug-95	0.001279623	0.007007935

GO:0042762	regulation of sulfur metabolic process	Aug-95	0.001279623	0.007007935
GO:0042996	regulation of Golgi to plasma membrane protein transport	Aug-95	0.001279623	0.007007935
GO:0045945	positive regulation of transcription by RNA polymerase III	Aug-95	0.001279623	0.007007935
GO:1905168	positive regulation of double-strand break repair via homologous recombination	Aug-95	0.001279623	0.007007935
GO:0010171	body morphogenesis	27/5895	0.00128699	0.007023891
GO:0032387	negative regulation of intracellular transport	27/5895	0.00128699	0.007023891
GO:0051058	negative regulation of small GTPase mediated signal transduction	27/5895	0.00128699	0.007023891
GO:0072665	protein localization to vacuole	27/5895	0.00128699	0.007023891
GO:0006513	protein monoubiquitination	30/5895	0.001289213	0.007029943
GO:0032233	positive regulation of actin filament bundle assembly	28/5895	0.00129405	0.007046712
GO:0006354	DNA-templated transcription, elongation	29/5895	0.001294522	0.007046712
GO:0000910	cytokinesis	58/5895	0.001300755	0.007074536
GO:0010657	muscle cell apoptotic process	40/5895	0.001317081	0.007151002
GO:0035418	protein localization to synapse	40/5895	0.001317081	0.007151002
GO:0007272	ensheathment of neurons	61/5895	0.001324214	0.007177375
GO:0008366	axon ensheathment	61/5895	0.001324214	0.007177375
GO:0035850	epithelial cell differentiation involved in kidney development	19/5895	0.001330094	0.007188908
GO:0050892	intestinal absorption	19/5895	0.001330094	0.007188908

GO:0071827	plasma lipoprotein particle organization	19/5895	0.001330094	0.007188908
GO:0050870	positive regulation of T cell activation	70/5895	0.001330899	0.007188908
GO:1902107	positive regulation of leukocyte differentiation	64/5895	0.00133606	0.007210608
GO:0071241	cellular response to inorganic substance	78/5895	0.001341097	0.007231605
GO:0006085	acetyl-CoA biosynthetic process	Nov-95	0.001363517	0.007327451
GO:0007398	ectoderm development	Nov-95	0.001363517	0.007327451
GO:0010867	positive regulation of triglyceride biosynthetic process	Nov-95	0.001363517	0.007327451
GO:0036315	cellular response to sterol	Nov-95	0.001363517	0.007327451
GO:0009065	glutamine family amino acid catabolic process	13/5895	0.001376858	0.007374021
GO:0010743	regulation of macrophage derived foam cell differentiation	13/5895	0.001376858	0.007374021
GO:0034405	response to fluid shear stress	13/5895	0.001376858	0.007374021
GO:0042537	benzene-containing compound metabolic process	13/5895	0.001376858	0.007374021
GO:0031032	actomyosin structure organization	72/5895	0.001388635	0.00742799
GO:0016571	histone methylation	53/5895	0.00138929	0.00742799
GO:1903578	regulation of ATP metabolic process	38/5895	0.001403328	0.007496697
GO:0048259	regulation of receptor-mediated endocytosis	44/5895	0.001406005	0.007498296
GO:0090100	positive regulation of transmembrane receptor protein serine/threonine kinase signaling pathway	44/5895	0.001406005	0.007498296
GO:0006509	membrane protein ectodomain proteolysis	20/5895	0.001431244	0.007607175

GO:0010765	positive regulation of sodium ion transport	20/5895	0.001431244	0.007607175
GO:0030501	positive regulation of bone mineralization	20/5895	0.001431244	0.007607175
GO:2000772	regulation of cellular senescence	20/5895	0.001431244	0.007607175
GO:0002028	regulation of sodium ion transport	37/5895	0.001445497	0.007670011
GO:0008589	regulation of smoothened signaling pathway	37/5895	0.001445497	0.007670011
GO:0006282	regulation of DNA repair	48/5895	0.001449187	0.007683124
GO:0003015	heart process	79/5895	0.001459833	0.007724566
GO:0009225	nucleotide-sugar metabolic process	16/5895	0.001461675	0.007724566
GO:1902932	positive regulation of alcohol biosynthetic process	16/5895	0.001461675	0.007724566
GO:1903844	regulation of cellular response to transforming growth factor beta stimulus	43/5895	0.001461901	0.007724566
GO:0032024	positive regulation of insulin secretion	36/5895	0.001486566	0.007848323
GO:0060047	heart contraction	76/5895	0.001493717	0.007879481
GO:0051588	regulation of neurotransmitter transport	55/5895	0.001506099	0.00793816
GO:0032873	negative regulation of stress-activated MAPK cascade	21/5895	0.001514979	0.007971644
GO:0070303	negative regulation of stress-activated protein kinase signaling cascade	21/5895	0.001514979	0.007971644
GO:0097756	negative regulation of blood vessel diameter	42/5895	0.001518793	0.007978407
GO:1900006	positive regulation of dendrite development	42/5895	0.001518793	0.007978407
GO:0048284	organelle fusion	51/5895	0.001525155	0.008005159

GO:0016197	endosomal transport	78/5895	0.001541035	0.008081787
GO:0006720	isoprenoid metabolic process	34/5895	0.001563693	0.008193803
GO:0006022	aminoglycan metabolic process	41/5895	0.001576518	0.008254153
GO:0045429	positive regulation of nitric oxide biosynthetic process	22/5895	0.001582215	0.008263408
GO:0048010	vascular endothelial growth factor receptor signaling pathway	22/5895	0.001582215	0.008263408
GO:1990928	response to amino acid starvation	22/5895	0.001582215	0.008263408
GO:0046209	nitric oxide metabolic process	33/5895	0.001598743	0.00834282
GO:0043484	regulation of RNA splicing	57/5895	0.001618495	0.008438915
GO:0032088	negative regulation of NF-kappaB transcription factor activity	32/5895	0.001630664	0.008481334
GO:0060563	neuroepithelial cell differentiation	32/5895	0.001630664	0.008481334
GO:0072332	intrinsic apoptotic signaling pathway by p53 class mediator	32/5895	0.001630664	0.008481334
GO:0046470	phosphatidylcholine metabolic process	23/5895	0.001634156	0.008485505
GO:0098754	detoxification	23/5895	0.001634156	0.008485505
GO:0099633	protein localization to postsynaptic specialization membrane	17/5895	0.001647085	0.00852784
GO:0099645	neurotransmitter receptor localization to postsynaptic specialization membrane	17/5895	0.001647085	0.00852784
GO:1904646	cellular response to amyloid-beta	17/5895	0.001647085	0.00852784
GO:0050821	protein stabilization	63/5895	0.001647716	0.00852784
GO:0014743	regulation of muscle hypertrophy	31/5895	0.001658769	0.008572563

GO:00171 48	negative regulation of translation	53/5895	0.0016590 74	0.0085725 63
GO:00341 13	heterotypic cell-cell adhesion	24/5895	0.0016721 46	0.0086330 35
GO:00106 11	regulation of cardiac muscle hypertrophy	30/5895	0.0016822 88	0.0086641 28
GO:00106 33	negative regulation of epithelial cell migration	30/5895	0.0016822 88	0.0086641 28
GO:00610 45	negative regulation of wound healing	30/5895	0.0016822 88	0.0086641 28
GO:00064 76	protein deacetylation	39/5895	0.0016936 37	0.0087154 63
GO:00031 70	heart valve development	25/5895	0.0016975 8	0.0087217 81
GO:00454 28	regulation of nitric oxide biosynthetic process	29/5895	0.0017003 6	0.0087217 81
GO:00903 44	negative regulation of cell aging	14/5895	0.0017003 95	0.0087217 81
GO:19048 93	negative regulation of receptor signaling pathway via STAT	14/5895	0.0017003 95	0.0087217 81
GO:00603 48	bone development	76/5895	0.0017173 58	0.0088016 33
GO:00464 34	organophosphate catabolic process	48/5895	0.0017499 78	0.0089615 33
GO:00435 23	regulation of neuron apoptotic process	90/5895	0.0017545 65	0.0089777 37
GO:00508 90	cognition	109/589 5	0.0017833 97	0.0090729 14
GO:00706 65	positive regulation of leukocyte proliferation	55/5895	0.0017878 72	0.0090729 14
GO:00066 58	phosphatidylserine metabolic process	Oct-95	0.0017904 23	0.0090729 14
GO:00148 56	skeletal muscle cell proliferation	Oct-95	0.0017904 23	0.0090729 14
GO:00423 08	negative regulation of protein import into nucleus	Oct-95	0.0017904 23	0.0090729 14

GO:0043217	myelin maintenance	Oct-95	0.001790423	0.009072914
GO:0045820	negative regulation of glycolytic process	Oct-95	0.001790423	0.009072914
GO:0060979	vasculogenesis involved in coronary vascular morphogenesis	Oct-95	0.001790423	0.009072914
GO:1901550	regulation of endothelial cell development	Oct-95	0.001790423	0.009072914
GO:1903140	regulation of establishment of endothelial barrier	Oct-95	0.001790423	0.009072914
GO:1903541	regulation of exosomal secretion	Oct-95	0.001790423	0.009072914
GO:1904590	negative regulation of protein import	Oct-95	0.001790423	0.009072914
GO:0051348	negative regulation of transferase activity	87/5895	0.001824044	0.009235867
GO:0051402	neuron apoptotic process	98/5895	0.001836286	0.009290399
GO:0006346	DNA methylation-dependent heterochromatin assembly	Dec-95	0.001856388	0.00936207
GO:0010039	response to iron ion	Dec-95	0.001856388	0.00936207
GO:0045821	positive regulation of glycolytic process	Dec-95	0.001856388	0.00936207
GO:2000209	regulation of anoikis	Dec-95	0.001856388	0.00936207
GO:0002526	acute inflammatory response	42/5895	0.001864825	0.009396929
GO:0001938	positive regulation of endothelial cell proliferation	36/5895	0.001869258	0.009396929
GO:0014015	positive regulation of gliogenesis	36/5895	0.001869258	0.009396929
GO:0048644	muscle organ morphogenesis	36/5895	0.001869258	0.009396929
GO:0030308	negative regulation of cell growth	69/5895	0.001886624	0.009476681

GO:0007269	neurotransmitter secretion	66/5895	0.001910999	0.009583858
GO:0099643	signal release from synapse	66/5895	0.001910999	0.009583858
GO:0032874	positive regulation of stress-activated MAPK cascade	60/5895	0.001925387	0.009614666
GO:0071897	DNA biosynthetic process	60/5895	0.001925387	0.009614666
GO:0006112	energy reserve metabolic process	35/5895	0.001926286	0.009614666
GO:0031960	response to corticosteroid	35/5895	0.001926286	0.009614666
GO:0060079	excitatory postsynaptic potential	35/5895	0.001926286	0.009614666
GO:1900182	positive regulation of protein localization to nucleus	35/5895	0.001926286	0.009614666
GO:0038179	neurotrophin signaling pathway	19/5895	0.001938918	0.009662427
GO:0070570	regulation of neuron projection regeneration	19/5895	0.001938918	0.009662427
GO:0098693	regulation of synaptic vesicle cycle	53/5895	0.001973709	0.009828042
GO:0034765	regulation of ion transmembrane transport	152/5895	0.001981442	0.009844764
GO:0000380	alternative mRNA splicing, via spliceosome	34/5895	0.001981748	0.009844764
GO:0006835	dicarboxylic acid transport	34/5895	0.001981748	0.009844764
GO:0021952	central nervous system projection neuron axonogenesis	15/5895	0.001990973	0.009859534
GO:0039694	viral RNA genome replication	15/5895	0.001990973	0.009859534
GO:0060444	branching involved in mammary gland duct morphogenesis	15/5895	0.001990973	0.009859534
GO:0150117	positive regulation of cell-substrate junction organization	15/5895	0.001990973	0.009859534

GO terms associated with 8759 genes near newly opened regions

ID	Description	GeneRatio	pvalue	p.adjust
GO:0007409	axonogenesis	320/7539	5.33E-54	3.36E-50
GO:0050808	synapse organization	317/7539	4.94E-53	1.56E-49
GO:0030900	forebrain development	258/7539	2.29E-42	4.82E-39
GO:0097485	neuron projection guidance	183/7539	1.47E-39	2.32E-36
GO:0007411	axon guidance	182/7539	3.35E-39	4.22E-36
GO:0034329	cell junction assembly	268/7539	5.65E-39	5.95E-36
GO:0034765	regulation of ion transmembrane transport	296/7539	2.70E-38	2.43E-35
GO:0060562	epithelial tube morphogenesis	245/7539	2.26E-36	1.78E-33
GO:0045666	positive regulation of neuron differentiation	284/7539	4.09E-36	2.87E-33
GO:0007264	small GTPase mediated signal transduction	267/7539	1.25E-35	7.91E-33
GO:0007389	pattern specification process	285/7539	3.10E-35	1.78E-32
GO:0031589	cell-substrate adhesion	225/7539	1.05E-34	5.33E-32
GO:0010721	negative regulation of cell development	242/7539	1.10E-34	5.33E-32
GO:0001655	urogenital system development	236/7539	3.19E-34	1.44E-31
GO:0060485	mesenchyme development	190/7539	4.08E-34	1.64E-31
GO:0050807	regulation of synapse organization	188/7539	4.16E-34	1.64E-31

GO:0072001	renal system development	215/7539	6.84E-34	2.54E-31
GO:0045165	cell fate commitment	194/7539	3.31E-33	1.11E-30
GO:0051961	negative regulation of nervous system development	229/7539	3.34E-33	1.11E-30
GO:0090596	sensory organ morphogenesis	203/7539	4.72E-33	1.49E-30
GO:0050803	regulation of synapse structure or activity	192/7539	7.13E-33	2.14E-30
GO:0001667	ameboidal-type cell migration	257/7539	8.21E-33	2.25E-30
GO:0010959	regulation of metal ion transport	257/7539	8.21E-33	2.25E-30
GO:0019932	second-messenger-mediated signaling	265/7539	1.03E-32	2.72E-30
GO:1904062	regulation of cation transmembrane transport	225/7539	5.03E-32	1.27E-29
GO:0050768	negative regulation of neurogenesis	213/7539	1.25E-31	3.04E-29
GO:0048562	embryonic organ morphogenesis	208/7539	3.29E-31	7.69E-29
GO:0001822	kidney development	203/7539	4.57E-31	1.03E-28
GO:0021953	central nervous system neuron differentiation	162/7539	8.44E-31	1.84E-28
GO:0051056	regulation of small GTPase mediated signal transduction	179/7539	2.94E-30	6.19E-28
GO:0045860	positive regulation of protein kinase activity	257/7539	6.80E-30	1.39E-27
GO:0060537	muscle tissue development	270/7539	1.01E-29	2.00E-27
GO:0048880	sensory system development	249/7539	4.58E-29	8.77E-27
GO:0001654	eye development	245/7539	7.22E-29	1.34E-26

GO:0001503	ossification	234/7539	8.77E-29	1.58E-26
GO:0010769	regulation of cell morphogenesis involved in differentiation	214/7539	1.31E-28	2.30E-26
GO:0031346	positive regulation of cell projection organization	271/7539	1.45E-28	2.48E-26
GO:0150063	visual system development	246/7539	1.74E-28	2.89E-26
GO:0014706	striated muscle tissue development	257/7539	2.02E-28	3.27E-26
GO:0042391	regulation of membrane potential	258/7539	2.87E-28	4.53E-26
GO:0015672	monovalent inorganic cation transport	275/7539	3.38E-28	5.19E-26
GO:0003002	regionalization	226/7539	3.45E-28	5.19E-26
GO:0021537	telencephalon development	166/7539	5.95E-28	8.73E-26
GO:0001763	morphogenesis of a branching structure	160/7539	1.45E-27	2.09E-25
GO:1901888	regulation of cell junction assembly	147/7539	1.75E-27	2.46E-25
GO:0001764	neuron migration	133/7539	6.60E-27	9.06E-25
GO:0048762	mesenchymal cell differentiation	151/7539	7.52E-27	1.01E-24
GO:0007517	muscle organ development	241/7539	1.48E-26	1.94E-24
GO:0043583	ear development	160/7539	4.47E-26	5.76E-24
GO:0042063	gliogenesis	203/7539	5.22E-26	6.59E-24
GO:0032412	regulation of ion transmembrane transporter activity	166/7539	6.22E-26	7.70E-24
GO:0043270	positive regulation of ion transport	201/7539	6.38E-26	7.74E-24

GO:0007015	actin filament organization	247/7539	7.05E-26	8.40E-24
GO:0016358	dendrite development	188/7539	7.22E-26	8.44E-24
GO:0099173	postsynapse organization	137/7539	7.84E-26	9.00E-24
GO:0022898	regulation of transmembrane transporter activity	171/7539	8.47E-26	9.55E-24
GO:0007265	Ras protein signal transduction	208/7539	8.91E-26	9.87E-24
GO:0006813	potassium ion transport	156/7539	2.05E-25	2.23E-23
GO:0032409	regulation of transporter activity	175/7539	2.13E-25	2.28E-23
GO:0045785	positive regulation of cell adhesion	245/7539	2.23E-25	2.34E-23
GO:0010976	positive regulation of neuron projection development	217/7539	2.49E-25	2.58E-23
GO:0003012	muscle system process	233/7539	2.53E-25	2.58E-23
GO:0042692	muscle cell differentiation	228/7539	7.42E-25	7.43E-23
GO:0046578	regulation of Ras protein signal transduction	155/7539	8.04E-25	7.93E-23
GO:0045665	negative regulation of neuron differentiation	164/7539	8.75E-25	8.50E-23
GO:0007178	transmembrane receptor protein serine/threonine kinase signaling pathway	214/7539	9.38E-25	8.97E-23
GO:0072073	kidney epithelium development	107/7539	1.24E-24	1.16E-22
GO:0061138	morphogenesis of a branching epithelium	145/7539	4.92E-24	4.57E-22
GO:0006836	neurotransmitter transport	156/7539	5.17E-24	4.73E-22
GO:2001257	regulation of cation channel activity	122/7539	7.18E-24	6.36E-22

GO:0043010	camera-type eye development	213/7539	7.23E-24	6.36E-22
GO:0006816	calcium ion transport	235/7539	7.25E-24	6.36E-22
GO:0048732	gland development	257/7539	7.68E-24	6.64E-22
GO:0061351	neural precursor cell proliferation	129/7539	1.57E-23	1.34E-21
GO:0060348	bone development	144/7539	1.96E-23	1.65E-21
GO:0007611	learning or memory	180/7539	2.23E-23	1.85E-21
GO:0048638	regulation of developmental growth	229/7539	2.42E-23	1.99E-21
GO:0055074	calcium ion homeostasis	265/7539	2.82E-23	2.28E-21
GO:0048663	neuron fate commitment	68/7539	4.91E-23	3.92E-21
GO:0098742	cell-cell adhesion via plasma-membrane adhesion molecules	137/7539	5.24E-23	4.14E-21
GO:0090287	regulation of cellular response to growth factor stimulus	171/7539	5.31E-23	4.14E-21
GO:0007416	synapse assembly	128/7539	6.89E-23	5.30E-21
GO:0090130	tissue migration	179/7539	7.16E-23	5.44E-21
GO:0051271	negative regulation of cellular component movement	191/7539	9.22E-23	6.85E-21
GO:0048863	stem cell differentiation	150/7539	9.29E-23	6.85E-21
GO:0060541	respiratory system development	158/7539	9.34E-23	6.85E-21
GO:1903522	regulation of blood circulation	155/7539	1.18E-22	8.58E-21
GO:0071805	potassium ion transmembrane transport	132/7539	1.25E-22	9.00E-21

GO:0050890	cognition	195/7539	1.56E-22	1.11E-20
GO:0070838	divalent metal ion transport	252/7539	1.67E-22	1.16E-20
GO:0198738	cell-cell signaling by wnt	237/7539	1.68E-22	1.16E-20
GO:0016055	Wnt signaling pathway	236/7539	1.91E-22	1.31E-20
GO:0090132	epithelium migration	177/7539	2.52E-22	1.71E-20
GO:0040013	negative regulation of locomotion	190/7539	2.77E-22	1.86E-20
GO:0055123	digestive system development	99/7539	3.73E-22	2.48E-20
GO:0072511	divalent inorganic cation transport	252/7539	5.38E-22	3.54E-20
GO:0060560	developmental growth involved in morphogenesis	166/7539	7.65E-22	4.98E-20
GO:0010631	epithelial cell migration	175/7539	8.78E-22	5.61E-20
GO:0006874	cellular calcium ion homeostasis	254/7539	8.79E-22	5.61E-20
GO:0070588	calcium ion transmembrane transport	169/7539	9.70E-22	6.13E-20
GO:0061448	connective tissue development	166/7539	1.31E-21	8.20E-20
GO:0051216	cartilage development	130/7539	2.05E-21	1.27E-19
GO:0048705	skeletal system morphogenesis	155/7539	2.15E-21	1.32E-19
GO:0030111	regulation of Wnt signaling pathway	178/7539	2.89E-21	1.75E-19
GO:0007160	cell-matrix adhesion	136/7539	5.28E-21	3.14E-19
GO:0010810	regulation of cell-substrate adhesion	136/7539	5.28E-21	3.14E-19

GO:0061458	reproductive system development	254/7539	5.73E-21	3.38E-19
GO:0050770	regulation of axonogenesis	133/7539	6.38E-21	3.73E-19
GO:0072006	nephron development	105/7539	6.60E-21	3.82E-19
GO:0003007	heart morphogenesis	165/7539	7.09E-21	4.07E-19
GO:0048608	reproductive structure development	252/7539	7.57E-21	4.25E-19
GO:0048736	appendage development	130/7539	7.60E-21	4.25E-19
GO:0060173	limb development	130/7539	7.60E-21	4.25E-19
GO:0048839	inner ear development	136/7539	9.81E-21	5.43E-19
GO:0021700	developmental maturation	187/7539	1.12E-20	6.14E-19
GO:0006936	muscle contraction	174/7539	1.22E-20	6.64E-19
GO:0090257	regulation of muscle system process	149/7539	1.99E-20	1.07E-18
GO:0051146	striated muscle cell differentiation	182/7539	2.04E-20	1.09E-18
GO:0071900	regulation of protein serine/threonine kinase activity	236/7539	2.25E-20	1.19E-18
GO:0031644	regulation of nervous system process	121/7539	2.29E-20	1.21E-18
GO:0050673	epithelial cell proliferation	234/7539	2.96E-20	1.54E-18
GO:0032970	regulation of actin filament-based process	222/7539	3.20E-20	1.65E-18
GO:0022407	regulation of cell-cell adhesion	229/7539	4.00E-20	2.05E-18
GO:0001505	regulation of neurotransmitter levels	154/7539	6.28E-20	3.20E-18

GO:20000 27	regulation of animal organ morphogenesis	132/753 9	8.13E-20	4.11E-18
GO:00487 54	branching morphogenesis of an epithelial tube	120/753 9	9.11E-20	4.56E-18
GO:00100 01	glial cell differentiation	150/753 9	9.36E-20	4.65E-18
GO:00076 26	locomotory behavior	154/753 9	1.06E-19	5.25E-18
GO:00351 07	appendage morphogenesis	115/753 9	1.13E-19	5.51E-18
GO:00351 08	limb morphogenesis	115/753 9	1.13E-19	5.51E-18
GO:00096 11	response to wounding	255/753 9	1.25E-19	6.05E-18
GO:00430 87	regulation of GTPase activity	174/753 9	1.35E-19	6.48E-18
GO:00099 14	hormone transport	204/753 9	1.58E-19	7.50E-18
GO:00468 79	hormone secretion	200/753 9	2.68E-19	1.26E-17
GO:00424 71	ear morphogenesis	96/7539	2.95E-19	1.38E-17
GO:00140 13	regulation of gliogenesis	104/753 9	3.05E-19	1.42E-17
GO:00709 97	neuron death	222/753 9	3.29E-19	1.52E-17
GO:00163 31	morphogenesis of embryonic epithelium	118/753 9	3.74E-19	1.71E-17
GO:00506 78	regulation of epithelial cell proliferation	203/753 9	4.09E-19	1.86E-17
GO:19013 42	regulation of vasculature development	196/753 9	4.53E-19	2.04E-17
GO:00514 80	regulation of cytosolic calcium ion concentration	204/753 9	5.36E-19	2.40E-17
GO:00181 08	peptidyl-tyrosine phosphorylation	181/753 9	5.62E-19	2.50E-17

GO:0031345	negative regulation of cell projection organization	129/7539	6.14E-19	2.71E-17
GO:0018212	peptidyl-tyrosine modification	182/7539	7.69E-19	3.37E-17
GO:0030323	respiratory tube development	137/7539	9.67E-19	4.21E-17
GO:0061572	actin filament bundle organization	112/7539	9.75E-19	4.22E-17
GO:0099175	regulation of postsynapse organization	92/7539	1.33E-18	5.72E-17
GO:0030278	regulation of ossification	144/7539	1.44E-18	6.12E-17
GO:0007612	learning	113/7539	1.79E-18	7.60E-17
GO:0003205	cardiac chamber development	122/7539	1.90E-18	7.99E-17
GO:0051924	regulation of calcium ion transport	159/7539	2.05E-18	8.56E-17
GO:0048565	digestive tract development	87/7539	2.55E-18	1.06E-16
GO:0061326	renal tubule development	77/7539	2.89E-18	1.19E-16
GO:0048738	cardiac muscle tissue development	151/7539	3.13E-18	1.28E-16
GO:0060047	heart contraction	134/7539	3.86E-18	1.57E-16
GO:0006939	smooth muscle contraction	82/7539	4.55E-18	1.84E-16
GO:0008016	regulation of heart contraction	116/7539	5.33E-18	2.14E-16
GO:0051963	regulation of synapse assembly	87/7539	5.85E-18	2.34E-16
GO:0010632	regulation of epithelial cell migration	140/7539	6.17E-18	2.45E-16
GO:0035148	tube formation	113/7539	6.46E-18	2.55E-16

GO:0030324	lung development	134/7539	6.64E-18	2.60E-16
GO:1901214	regulation of neuron death	202/7539	7.45E-18	2.90E-16
GO:0032147	activation of protein kinase activity	143/7539	7.53E-18	2.92E-16
GO:0003015	heart process	138/7539	7.59E-18	2.92E-16
GO:0001823	mesonephros development	78/7539	8.15E-18	3.10E-16
GO:0051017	actin filament bundle assembly	109/7539	8.15E-18	3.10E-16
GO:0099003	vesicle-mediated transport in synapse	151/7539	8.36E-18	3.16E-16
GO:0030336	negative regulation of cell migration	159/7539	8.44E-18	3.17E-16
GO:0048588	developmental cell growth	154/7539	9.55E-18	3.55E-16
GO:0072009	nephron epithelium development	80/7539	9.56E-18	3.55E-16
GO:0019722	calcium-mediated signaling	130/7539	9.73E-18	3.59E-16
GO:0001657	ureteric bud development	76/7539	1.70E-17	6.16E-16
GO:0072163	mesonephric epithelium development	76/7539	1.70E-17	6.16E-16
GO:0072164	mesonephric tubule development	76/7539	1.70E-17	6.16E-16
GO:0071902	positive regulation of protein serine/threonine kinase activity	164/7539	1.79E-17	6.42E-16
GO:2000146	negative regulation of cell motility	164/7539	1.79E-17	6.42E-16
GO:0048015	phosphatidylinositol-mediated signaling	106/7539	1.83E-17	6.54E-16
GO:0014033	neural crest cell differentiation	69/7539	1.87E-17	6.64E-16

GO:0071695	anatomical structure maturation	156/7539	1.93E-17	6.77E-16
GO:1990778	protein localization to cell periphery	195/7539	1.93E-17	6.77E-16
GO:0032956	regulation of actin cytoskeleton organization	194/7539	2.21E-17	7.71E-16
GO:0090092	regulation of transmembrane receptor protein serine/threonine kinase signaling pathway	144/7539	3.02E-17	1.05E-15
GO:0048017	inositol lipid-mediated signaling	107/7539	3.30E-17	1.14E-15
GO:1902903	regulation of supramolecular fiber organization	197/7539	4.63E-17	1.59E-15
GO:0015837	amine transport	84/7539	6.08E-17	2.07E-15
GO:0072080	nephron tubule development	72/7539	7.33E-17	2.49E-15
GO:0007266	Rho protein signal transduction	113/7539	7.47E-17	2.52E-15
GO:0033002	muscle cell proliferation	138/7539	9.70E-17	3.26E-15
GO:0070371	ERK1 and ERK2 cascade	177/7539	9.86E-17	3.29E-15
GO:0043266	regulation of potassium ion transport	77/7539	1.08E-16	3.57E-15
GO:0030902	hindbrain development	108/7539	1.08E-16	3.57E-15
GO:0019935	cyclic-nucleotide-mediated signaling	132/7539	1.13E-16	3.69E-15
GO:0032535	regulation of cellular component size	214/7539	1.13E-16	3.69E-15
GO:0072089	stem cell proliferation	95/7539	1.14E-16	3.69E-15
GO:0045444	fat cell differentiation	145/7539	1.14E-16	3.69E-15
GO:0060070	canonical Wnt signaling pathway	159/7539	1.26E-16	4.06E-15

GO:0046883	regulation of hormone secretion	167/7539	1.62E-16	5.20E-15
GO:0003018	vascular process in circulatory system	132/7539	1.88E-16	5.99E-15
GO:0070372	regulation of ERK1 and ERK2 cascade	169/7539	1.89E-16	6.00E-15
GO:1904064	positive regulation of cation transmembrane transport	107/7539	2.13E-16	6.74E-15
GO:0035270	endocrine system development	96/7539	2.16E-16	6.79E-15
GO:0045765	regulation of angiogenesis	174/7539	2.20E-16	6.87E-15
GO:0002062	chondrocyte differentiation	77/7539	2.51E-16	7.79E-15
GO:0021915	neural tube development	118/7539	2.71E-16	8.38E-15
GO:0048864	stem cell development	67/7539	2.79E-16	8.59E-15
GO:0030198	extracellular matrix organization	166/7539	2.80E-16	8.59E-15
GO:0001894	tissue homeostasis	148/7539	3.18E-16	9.71E-15
GO:0034764	positive regulation of transmembrane transport	140/7539	3.30E-16	1.00E-14
GO:0006937	regulation of muscle contraction	100/7539	3.38E-16	1.02E-14
GO:0071559	response to transforming growth factor beta	126/7539	3.56E-16	1.07E-14
GO:0043062	extracellular structure organization	166/7539	4.23E-16	1.27E-14
GO:0048592	eye morphogenesis	112/7539	4.64E-16	1.38E-14
GO:0048639	positive regulation of developmental growth	128/7539	4.81E-16	1.42E-14
GO:0015844	monoamine transport	74/7539	5.06E-16	1.49E-14

GO:0035023	regulation of Rho protein signal transduction	91/7539	5.20E-16	1.53E-14
GO:0007162	negative regulation of cell adhesion	160/7539	6.14E-16	1.79E-14
GO:0007269	neurotransmitter secretion	115/7539	6.22E-16	1.80E-14
GO:0099643	signal release from synapse	115/7539	6.22E-16	1.80E-14
GO:0014031	mesenchymal cell development	66/7539	6.48E-16	1.87E-14
GO:0034767	positive regulation of ion transmembrane transport	114/7539	6.82E-16	1.96E-14
GO:1990138	neuron projection extension	119/7539	7.28E-16	2.08E-14
GO:0031032	actomyosin structure organization	123/7539	8.19E-16	2.33E-14
GO:0003206	cardiac chamber morphogenesis	95/7539	8.52E-16	2.41E-14
GO:0007163	establishment or maintenance of cell polarity	130/7539	1.04E-15	2.94E-14
GO:0043542	endothelial cell migration	125/7539	1.10E-15	3.10E-14
GO:0002064	epithelial cell development	145/7539	1.13E-15	3.17E-14
GO:0043405	regulation of MAP kinase activity	164/7539	1.24E-15	3.46E-14
GO:0002040	sprouting angiogenesis	83/7539	1.29E-15	3.57E-14
GO:0022612	gland morphogenesis	98/7539	1.35E-15	3.73E-14
GO:0071560	cellular response to transforming growth factor beta stimulus	123/7539	1.37E-15	3.76E-14
GO:0090288	negative regulation of cellular response to growth factor stimulus	97/7539	1.44E-15	3.94E-14
GO:0045598	regulation of fat cell differentiation	94/7539	1.72E-15	4.68E-14

GO:0007204	positive regulation of cytosolic calcium ion concentration	176/7539	1.74E-15	4.72E-14
GO:0045685	regulation of glial cell differentiation	67/7539	1.82E-15	4.91E-14
GO:0010977	negative regulation of neuron projection development	108/7539	2.05E-15	5.51E-14
GO:0045995	regulation of embryonic development	88/7539	2.29E-15	6.12E-14
GO:0045927	positive regulation of growth	168/7539	2.40E-15	6.38E-14
GO:0003279	cardiac septum development	85/7539	2.54E-15	6.72E-14
GO:2000177	regulation of neural precursor cell proliferation	80/7539	2.79E-15	7.34E-14
GO:0042472	inner ear morphogenesis	79/7539	2.80E-15	7.34E-14
GO:0051952	regulation of amine transport	79/7539	2.80E-15	7.34E-14
GO:0051588	regulation of neurotransmitter transport	96/7539	2.87E-15	7.50E-14
GO:0051048	negative regulation of secretion	129/7539	3.09E-15	8.02E-14
GO:0030326	embryonic limb morphogenesis	92/7539	3.67E-15	9.44E-14
GO:0035113	embryonic appendage morphogenesis	92/7539	3.67E-15	9.44E-14
GO:0072175	epithelial tube formation	101/7539	3.68E-15	9.44E-14
GO:0019933	cAMP-mediated signaling	116/7539	4.79E-15	1.23E-13
GO:0048813	dendrite morphogenesis	110/7539	5.16E-15	1.31E-13
GO:0060993	kidney morphogenesis	71/7539	5.47E-15	1.39E-13
GO:0009952	anterior/posterior pattern specification	132/7539	5.56E-15	1.40E-13

GO:0001952	regulation of cell-matrix adhesion	80/7539	5.81E-15	1.46E-13
GO:0042476	odontogenesis	80/7539	5.81E-15	1.46E-13
GO:1901890	positive regulation of cell junction assembly	79/7539	5.90E-15	1.47E-13
GO:0071772	response to BMP	108/7539	6.24E-15	1.54E-13
GO:0071773	cellular response to BMP stimulus	108/7539	6.24E-15	1.54E-13
GO:1903169	regulation of calcium ion transmembrane transport	101/7539	6.61E-15	1.63E-13
GO:0003151	outflow tract morphogenesis	62/7539	7.15E-15	1.75E-13
GO:0014032	neural crest cell development	62/7539	7.15E-15	1.75E-13
GO:0035265	organ growth	121/7539	7.68E-15	1.87E-13
GO:0055001	muscle cell development	120/7539	8.58E-15	2.08E-13
GO:0001838	embryonic epithelial tube formation	97/7539	9.07E-15	2.19E-13
GO:0030072	peptide hormone secretion	155/7539	9.21E-15	2.22E-13
GO:1901215	negative regulation of neuron death	140/7539	1.23E-14	2.96E-13
GO:0045778	positive regulation of ossification	72/7539	1.25E-14	3.00E-13
GO:0008306	associative learning	74/7539	1.28E-14	3.05E-13
GO:0001933	negative regulation of protein phosphorylation	213/7539	1.32E-14	3.14E-13
GO:0050773	regulation of dendrite development	116/7539	1.33E-14	3.15E-13
GO:0030100	regulation of endocytosis	127/7539	1.61E-14	3.79E-13

GO:0030177	positive regulation of Wnt signaling pathway	87/7539	1.76E-14	4.13E-13
GO:0051937	catecholamine transport	62/7539	1.81E-14	4.23E-13
GO:0001656	metanephros development	64/7539	2.11E-14	4.91E-13
GO:0044344	cellular response to fibroblast growth factor stimulus	65/7539	2.24E-14	5.20E-13
GO:0042060	wound healing	181/7539	2.30E-14	5.31E-13
GO:0046777	protein autophosphorylation	135/7539	2.32E-14	5.35E-13
GO:0007215	glutamate receptor signaling pathway	66/7539	2.36E-14	5.42E-13
GO:0001649	osteoblast differentiation	119/7539	2.56E-14	5.86E-13
GO:0043406	positive regulation of MAP kinase activity	128/7539	3.56E-14	8.12E-13
GO:0010469	regulation of signaling receptor activity	85/7539	3.75E-14	8.49E-13
GO:0015850	organic hydroxy compound transport	138/7539	3.75E-14	8.49E-13
GO:0050730	regulation of peptidyl-tyrosine phosphorylation	144/7539	3.95E-14	8.91E-13
GO:0099504	synaptic vesicle cycle	130/7539	4.35E-14	9.78E-13
GO:0042326	negative regulation of phosphorylation	230/7539	4.38E-14	9.81E-13
GO:0061333	renal tubule morphogenesis	62/7539	4.43E-14	9.89E-13
GO:0051098	regulation of binding	197/7539	4.90E-14	1.09E-12
GO:0008544	epidermis development	165/7539	5.08E-14	1.12E-12
GO:0006814	sodium ion transport	131/7539	5.96E-14	1.32E-12

GO:0048771	tissue remodeling	114/7539	7.36E-14	1.62E-12
GO:0014065	phosphatidylinositol 3-kinase signaling	83/7539	7.99E-14	1.75E-12
GO:0021675	nerve development	59/7539	8.60E-14	1.88E-12
GO:0098657	import into cell	138/7539	8.68E-14	1.89E-12
GO:0031098	stress-activated protein kinase signaling cascade	147/7539	8.85E-14	1.92E-12
GO:0051402	neuron apoptotic process	158/7539	9.19E-14	1.99E-12
GO:0001837	epithelial to mesenchymal transition	90/7539	9.26E-14	1.99E-12
GO:0021872	forebrain generation of neurons	60/7539	9.32E-14	2.00E-12
GO:0099601	regulation of neurotransmitter receptor activity	52/7539	9.43E-14	2.02E-12
GO:0032414	positive regulation of ion transmembrane transporter activity	77/7539	1.06E-13	2.26E-12
GO:0071774	response to fibroblast growth factor	65/7539	1.19E-13	2.53E-12
GO:0060538	skeletal muscle organ development	118/7539	1.20E-13	2.53E-12
GO:0035051	cardiocyte differentiation	105/7539	1.20E-13	2.53E-12
GO:0021954	central nervous system neuron development	66/7539	1.22E-13	2.56E-12
GO:0001708	cell fate specification	71/7539	1.23E-13	2.59E-12
GO:0048593	camera-type eye morphogenesis	93/7539	1.30E-13	2.71E-12
GO:0008361	regulation of cell size	121/7539	1.32E-13	2.75E-12
GO:0031214	biomineral tissue development	98/7539	1.43E-13	2.97E-12

GO:0110148	biomineralization	98/7539	1.43E-13	2.97E-12
GO:0003231	cardiac ventricle development	91/7539	1.54E-13	3.17E-12
GO:0060828	regulation of canonical Wnt signaling pathway	130/7539	1.61E-13	3.30E-12
GO:0032411	positive regulation of transporter activity	80/7539	1.80E-13	3.69E-12
GO:0050900	leukocyte migration	183/7539	1.84E-13	3.76E-12
GO:1905330	regulation of morphogenesis of an epithelium	75/7539	2.27E-13	4.62E-12
GO:0060411	cardiac septum morphogenesis	61/7539	2.35E-13	4.75E-12
GO:1901379	regulation of potassium ion transmembrane transport	61/7539	2.35E-13	4.75E-12
GO:0001755	neural crest cell migration	46/7539	2.37E-13	4.78E-12
GO:0007188	adenylate cyclase-modulating G protein-coupled receptor signaling pathway	123/7539	2.53E-13	5.08E-12
GO:0021510	spinal cord development	71/7539	2.56E-13	5.13E-12
GO:1903532	positive regulation of secretion by cell	194/7539	2.64E-13	5.28E-12
GO:0050679	positive regulation of epithelial cell proliferation	118/7539	3.00E-13	5.98E-12
GO:0060996	dendritic spine development	81/7539	3.21E-13	6.38E-12
GO:0021543	pallium development	95/7539	3.29E-13	6.52E-12
GO:0002791	regulation of peptide secretion	201/7539	3.34E-13	6.59E-12
GO:0021879	forebrain neuron differentiation	54/7539	3.38E-13	6.65E-12
GO:0048645	animal organ formation	55/7539	3.75E-13	7.34E-12

GO:0050905	neuromuscular process	87/7539	3.82E-13	7.46E-12
GO:0051403	stress-activated MAPK cascade	139/7539	3.83E-13	7.47E-12
GO:1903706	regulation of hemopoiesis	208/7539	4.07E-13	7.91E-12
GO:0072659	protein localization to plasma membrane	155/7539	4.18E-13	8.09E-12
GO:0050433	regulation of catecholamine secretion	50/7539	5.33E-13	1.03E-11
GO:0150115	cell-substrate junction organization	64/7539	5.69E-13	1.09E-11
GO:0001570	vasculogenesis	66/7539	5.70E-13	1.09E-11
GO:0010463	mesenchymal cell proliferation	45/7539	5.85E-13	1.12E-11
GO:0050432	catecholamine secretion	51/7539	6.16E-13	1.17E-11
GO:0043523	regulation of neuron apoptotic process	144/7539	6.22E-13	1.18E-11
GO:0001558	regulation of cell growth	213/7539	6.37E-13	1.21E-11
GO:0035296	regulation of tube diameter	103/7539	6.78E-13	1.28E-11
GO:0097746	regulation of blood vessel diameter	103/7539	6.78E-13	1.28E-11
GO:0043588	skin development	146/7539	6.90E-13	1.30E-11
GO:1901343	negative regulation of vasculature development	86/7539	7.41E-13	1.39E-11
GO:0030282	bone mineralization	77/7539	7.73E-13	1.44E-11
GO:0110053	regulation of actin filament organization	145/7539	7.91E-13	1.47E-11
GO:0002065	columnar/cuboidal epithelial cell differentiation	91/7539	8.32E-13	1.54E-11

GO:0010634	positive regulation of epithelial cell migration	91/7539	8.32E-13	1.54E-11
GO:0030178	negative regulation of Wnt signaling pathway	96/7539	8.65E-13	1.60E-11
GO:0055002	striated muscle cell development	109/7539	8.89E-13	1.64E-11
GO:0048665	neuron fate specification	33/7539	9.11E-13	1.67E-11
GO:0007156	homophilic cell adhesion via plasma membrane adhesion molecules	72/7539	9.98E-13	1.83E-11
GO:0035150	regulation of tube size	103/7539	1.11E-12	2.02E-11
GO:0050731	positive regulation of peptidyl-tyrosine phosphorylation	111/7539	1.11E-12	2.02E-11
GO:0048546	digestive tract morphogenesis	43/7539	1.12E-12	2.03E-11
GO:0072028	nephron morphogenesis	59/7539	1.14E-12	2.07E-11
GO:0010594	regulation of endothelial cell migration	98/7539	1.17E-12	2.11E-11
GO:0097581	lamellipodium organization	60/7539	1.18E-12	2.12E-11
GO:0030510	regulation of BMP signaling pathway	66/7539	1.19E-12	2.14E-11
GO:0048706	embryonic skeletal system development	87/7539	1.20E-12	2.15E-11
GO:0060021	roof of mouth development	65/7539	1.21E-12	2.16E-11
GO:0007044	cell-substrate junction assembly	62/7539	1.22E-12	2.16E-11
GO:0071692	protein localization to extracellular region	228/7539	1.22E-12	2.16E-11
GO:0032872	regulation of stress-activated MAPK cascade	127/7539	1.26E-12	2.23E-11
GO:0030534	adult behavior	106/7539	1.27E-12	2.23E-11

GO:1900449	regulation of glutamate receptor signaling pathway	44/7539	1.44E-12	2.52E-11
GO:0006940	regulation of smooth muscle contraction	50/7539	1.44E-12	2.52E-11
GO:0007219	Notch signaling pathway	104/7539	1.60E-12	2.80E-11
GO:0070302	regulation of stress-activated protein kinase signaling cascade	128/7539	1.66E-12	2.89E-11
GO:0030099	myeloid cell differentiation	202/7539	1.69E-12	2.94E-11
GO:0046928	regulation of neurotransmitter secretion	83/7539	1.70E-12	2.94E-11
GO:0007179	transforming growth factor beta receptor signaling pathway	99/7539	1.74E-12	3.00E-11
GO:0030509	BMP signaling pathway	98/7539	1.94E-12	3.34E-11
GO:1902105	regulation of leukocyte differentiation	159/7539	1.96E-12	3.38E-11
GO:0009306	protein secretion	224/7539	2.12E-12	3.64E-11
GO:0050920	regulation of chemotaxis	123/7539	2.13E-12	3.65E-11
GO:0048660	regulation of smooth muscle cell proliferation	97/7539	2.16E-12	3.69E-11
GO:0035592	establishment of protein localization to extracellular region	224/7539	2.76E-12	4.70E-11
GO:0048659	smooth muscle cell proliferation	99/7539	2.84E-12	4.82E-11
GO:0099084	postsynaptic specialization organization	35/7539	3.03E-12	5.13E-11
GO:0060401	cytosolic calcium ion transport	102/7539	3.25E-12	5.49E-11
GO:0006887	exocytosis	191/7539	3.33E-12	5.60E-11
GO:0060402	calcium ion transport into cytosol	93/7539	3.33E-12	5.60E-11

GO:1903531	negative regulation of secretion by cell	109/7539	3.51E-12	5.88E-11
GO:0048640	negative regulation of developmental growth	81/7539	3.58E-12	5.97E-11
GO:0070374	positive regulation of ERK1 and ERK2 cascade	119/7539	3.59E-12	5.99E-11
GO:0038127	ERBB signaling pathway	72/7539	3.72E-12	6.18E-11
GO:0007405	neuroblast proliferation	51/7539	4.08E-12	6.77E-11
GO:0045055	regulated exocytosis	138/7539	4.34E-12	7.17E-11
GO:0051047	positive regulation of secretion	211/7539	4.45E-12	7.33E-11
GO:1901861	regulation of muscle tissue development	103/7539	4.62E-12	7.60E-11
GO:0010771	negative regulation of cell morphogenesis involved in differentiation	66/7539	4.94E-12	8.10E-11
GO:0001935	endothelial cell proliferation	89/7539	5.06E-12	8.28E-11
GO:0008038	neuron recognition	40/7539	5.29E-12	8.62E-11
GO:0048041	focal adhesion assembly	56/7539	5.34E-12	8.65E-11
GO:0072078	nephron tubule morphogenesis	56/7539	5.34E-12	8.65E-11
GO:2001259	positive regulation of cation channel activity	56/7539	5.34E-12	8.65E-11
GO:0072088	nephron epithelium morphogenesis	57/7539	5.47E-12	8.83E-11
GO:0043271	negative regulation of ion transport	100/7539	6.60E-12	1.06E-10
GO:0097106	postsynaptic density organization	31/7539	6.98E-12	1.12E-10
GO:0048634	regulation of muscle organ development	103/7539	7.33E-12	1.17E-10

GO:0010811	positive regulation of cell-substrate adhesion	79/7539	7.53E-12	1.20E-10
GO:0060249	anatomical structure homeostasis	203/7539	7.88E-12	1.26E-10
GO:0030048	actin filament-based movement	78/7539	8.20E-12	1.30E-10
GO:0007272	ensheathment of neurons	98/7539	8.35E-12	1.32E-10
GO:0008366	axon ensheathment	98/7539	8.35E-12	1.32E-10
GO:0021536	diencephalon development	50/7539	9.28E-12	1.46E-10
GO:0016202	regulation of striated muscle tissue development	101/7539	9.33E-12	1.47E-10
GO:0090276	regulation of peptide hormone secretion	126/7539	1.05E-11	1.64E-10
GO:0019233	sensory perception of pain	87/7539	1.05E-11	1.64E-10
GO:0035637	multicellular organismal signaling	87/7539	1.05E-11	1.64E-10
GO:0014015	positive regulation of gliogenesis	61/7539	1.15E-11	1.80E-10
GO:0060078	regulation of postsynaptic membrane potential	72/7539	1.30E-11	2.03E-10
GO:0007519	skeletal muscle tissue development	109/7539	1.31E-11	2.03E-10
GO:0070304	positive regulation of stress-activated protein kinase signaling cascade	98/7539	1.34E-11	2.07E-10
GO:0032231	regulation of actin filament bundle assembly	70/7539	1.49E-11	2.30E-10
GO:0048709	oligodendrocyte differentiation	70/7539	1.49E-11	2.30E-10
GO:0032874	positive regulation of stress-activated MAPK cascade	97/7539	1.51E-11	2.31E-10
GO:0001508	action potential	83/7539	1.57E-11	2.41E-10

GO:0048675	axon extension	83/7539	1.57E-11	2.41E-10
GO:0007613	memory	88/7539	1.58E-11	2.41E-10
GO:1902414	protein localization to cell junction	77/7539	1.59E-11	2.41E-10
GO:0042552	myelination	96/7539	1.70E-11	2.57E-10
GO:0051928	positive regulation of calcium ion transport	82/7539	1.74E-11	2.63E-10
GO:0060349	bone morphogenesis	67/7539	1.79E-11	2.71E-10
GO:0050708	regulation of protein secretion	179/7539	1.80E-11	2.71E-10
GO:0030010	establishment of cell polarity	85/7539	2.17E-11	3.26E-10
GO:0010770	positive regulation of cell morphogenesis involved in differentiation	105/7539	2.17E-11	3.26E-10
GO:0060998	regulation of dendritic spine development	63/7539	2.18E-11	3.26E-10
GO:0010717	regulation of epithelial to mesenchymal transition	62/7539	2.26E-11	3.38E-10
GO:0072171	mesonephric tubule morphogenesis	51/7539	2.34E-11	3.48E-10
GO:0048259	regulation of receptor-mediated endocytosis	72/7539	2.39E-11	3.54E-10
GO:0016079	synaptic vesicle exocytosis	84/7539	2.41E-11	3.57E-10
GO:0008543	fibroblast growth factor receptor signaling pathway	53/7539	2.49E-11	3.68E-10
GO:1901019	regulation of calcium ion transmembrane transporter activity	56/7539	2.56E-11	3.78E-10
GO:0098727	maintenance of cell number	99/7539	2.96E-11	4.36E-10
GO:0110020	regulation of actomyosin structure organization	68/7539	3.18E-11	4.66E-10

GO:0015711	organic anion transport	217/7539	3.39E-11	4.96E-10
GO:0043491	protein kinase B signaling	108/7539	3.46E-11	5.06E-10
GO:0001841	neural tube formation	80/7539	3.62E-11	5.28E-10
GO:0001666	response to hypoxia	113/7539	4.06E-11	5.90E-10
GO:0150116	regulation of cell-substrate junction organization	46/7539	4.08E-11	5.93E-10
GO:0007187	G protein-coupled receptor signaling pathway, coupled to cyclic nucleotide second messenger	129/7539	4.47E-11	6.47E-10
GO:0043268	positive regulation of potassium ion transport	40/7539	5.01E-11	7.22E-10
GO:0048483	autonomic nervous system development	40/7539	5.01E-11	7.22E-10
GO:0060675	ureteric bud morphogenesis	50/7539	5.16E-11	7.42E-10
GO:0097553	calcium ion transmembrane import into cytosol	81/7539	5.54E-11	7.95E-10
GO:0060840	artery development	68/7539	5.87E-11	8.40E-10
GO:0035272	exocrine system development	41/7539	5.93E-11	8.46E-10
GO:0060041	retina development in camera-type eye	97/7539	5.94E-11	8.46E-10
GO:0035264	multicellular organism growth	116/7539	5.96E-11	8.47E-10
GO:1905475	regulation of protein localization to membrane	107/7539	5.97E-11	8.47E-10
GO:0062197	cellular response to chemical stress	164/7539	6.10E-11	8.64E-10
GO:0031532	actin cytoskeleton reorganization	67/7539	6.31E-11	8.89E-10
GO:0034446	substrate adhesion-dependent cell spreading	67/7539	6.31E-11	8.89E-10

GO:1903305	regulation of regulated secretory pathway	106/7539	6.80E-11	9.57E-10
GO:0043534	blood vessel endothelial cell migration	73/7539	7.01E-11	9.84E-10
GO:0048469	cell maturation	111/7539	7.89E-11	1.10E-09
GO:0043393	regulation of protein binding	122/7539	8.15E-11	1.14E-09
GO:0061387	regulation of extent of cell growth	77/7539	8.32E-11	1.16E-09
GO:0051893	regulation of focal adhesion assembly	44/7539	8.55E-11	1.19E-09
GO:0090109	regulation of cell-substrate junction assembly	44/7539	8.55E-11	1.19E-09
GO:2000181	negative regulation of blood vessel morphogenesis	76/7539	9.19E-11	1.27E-09
GO:0021846	cell proliferation in forebrain	30/7539	9.26E-11	1.28E-09
GO:0051058	negative regulation of small GTPase mediated signal transduction	45/7539	9.30E-11	1.28E-09
GO:0030073	insulin secretion	121/7539	9.35E-11	1.29E-09
GO:0022409	positive regulation of cell-cell adhesion	136/7539	9.63E-11	1.32E-09
GO:0043547	positive regulation of GTPase activity	118/7539	9.72E-11	1.33E-09
GO:0046888	negative regulation of hormone secretion	59/7539	9.99E-11	1.37E-09
GO:0042475	odontogenesis of dentin-containing tooth	58/7539	1.04E-10	1.42E-09
GO:0019827	stem cell population maintenance	96/7539	1.05E-10	1.43E-09
GO:0003281	ventricular septum development	57/7539	1.08E-10	1.46E-09
GO:0030032	lamellipodium assembly	48/7539	1.10E-10	1.49E-09

GO:0032102	negative regulation of response to external stimulus	184/7539	1.11E-10	1.50E-09
GO:0002573	myeloid leukocyte differentiation	117/7539	1.11E-10	1.50E-09
GO:2000179	positive regulation of neural precursor cell proliferation	49/7539	1.13E-10	1.52E-09
GO:0030500	regulation of bone mineralization	55/7539	1.14E-10	1.52E-09
GO:0050805	negative regulation of synaptic transmission	55/7539	1.14E-10	1.52E-09
GO:0030501	positive regulation of bone mineralization	34/7539	1.17E-10	1.57E-09
GO:0015849	organic acid transport	165/7539	1.28E-10	1.71E-09
GO:0007173	epidermal growth factor receptor signaling pathway	65/7539	1.34E-10	1.78E-09
GO:0017157	regulation of exocytosis	131/7539	1.38E-10	1.83E-09
GO:0048713	regulation of oligodendrocyte differentiation	40/7539	1.40E-10	1.86E-09
GO:0099560	synaptic membrane adhesion	28/7539	1.44E-10	1.90E-09
GO:0008217	regulation of blood pressure	112/7539	1.50E-10	1.99E-09
GO:0021761	limbic system development	63/7539	1.54E-10	2.02E-09
GO:0070167	regulation of biomineral tissue development	63/7539	1.54E-10	2.02E-09
GO:0110149	regulation of biomineralization	63/7539	1.54E-10	2.02E-09
GO:0050919	negative chemotaxis	35/7539	1.55E-10	2.04E-09
GO:0030856	regulation of epithelial cell differentiation	89/7539	1.58E-10	2.07E-09
GO:0007498	mesoderm development	70/7539	1.61E-10	2.10E-09

GO:1902904	negative regulation of supramolecular fiber organization	92/7539	1.73E-10	2.25E-09
GO:0051099	positive regulation of binding	108/7539	1.75E-10	2.27E-09
GO:0030038	contractile actin filament bundle assembly	69/7539	1.76E-10	2.27E-09
GO:0043149	stress fiber assembly	69/7539	1.76E-10	2.27E-09
GO:0001936	regulation of endothelial cell proliferation	79/7539	1.91E-10	2.46E-09
GO:0009791	post-embryonic development	79/7539	1.91E-10	2.46E-09
GO:0072091	regulation of stem cell proliferation	59/7539	1.95E-10	2.50E-09
GO:0045216	cell-cell junction organization	110/7539	1.97E-10	2.53E-09
GO:0006979	response to oxidative stress	193/7539	2.01E-10	2.58E-09
GO:0021987	cerebral cortex development	67/7539	2.08E-10	2.66E-09
GO:0034103	regulation of tissue remodeling	57/7539	2.14E-10	2.73E-09
GO:0021983	pituitary gland development	32/7539	2.15E-10	2.74E-09
GO:0014812	muscle cell migration	66/7539	2.26E-10	2.87E-09
GO:0001738	morphogenesis of a polarized epithelium	55/7539	2.31E-10	2.93E-09
GO:0021515	cell differentiation in spinal cord	46/7539	2.34E-10	2.96E-09
GO:0061035	regulation of cartilage development	49/7539	2.51E-10	3.17E-09
GO:1903670	regulation of sprouting angiogenesis	51/7539	2.51E-10	3.17E-09
GO:0099565	chemical synaptic transmission, postsynaptic	62/7539	3.06E-10	3.84E-09

GO:0030516	regulation of axon extension	69/7539	3.09E-10	3.88E-09
GO:0045933	positive regulation of muscle contraction	39/7539	3.30E-10	4.13E-09
GO:0007369	gastrulation	97/7539	3.34E-10	4.17E-09
GO:0045926	negative regulation of growth	134/7539	3.44E-10	4.29E-09
GO:0046942	carboxylic acid transport	162/7539	3.46E-10	4.31E-09
GO:0042303	molting cycle	78/7539	3.53E-10	4.37E-09
GO:0042633	hair cycle	78/7539	3.53E-10	4.37E-09
GO:0016525	negative regulation of angiogenesis	73/7539	3.60E-10	4.46E-09
GO:0035249	synaptic transmission, glutamatergic	67/7539	3.70E-10	4.57E-09
GO:0015872	dopamine transport	41/7539	4.10E-10	5.04E-09
GO:0045599	negative regulation of fat cell differentiation	41/7539	4.10E-10	5.04E-09
GO:0008593	regulation of Notch signaling pathway	57/7539	4.16E-10	5.10E-09
GO:0060079	excitatory postsynaptic potential	57/7539	4.16E-10	5.10E-09
GO:0007254	JNK cascade	107/7539	4.35E-10	5.32E-09
GO:1903034	regulation of response to wounding	101/7539	4.46E-10	5.44E-09
GO:0006869	lipid transport	174/7539	4.47E-10	5.44E-09
GO:0045667	regulation of osteoblast differentiation	84/7539	4.59E-10	5.58E-09
GO:0006941	striated muscle contraction	91/7539	4.72E-10	5.73E-09

GO:1901016	regulation of potassium ion transmembrane transporter activity	43/7539	4.74E-10	5.74E-09
GO:0050772	positive regulation of axonogenesis	64/7539	4.78E-10	5.78E-09
GO:0051090	regulation of DNA-binding transcription factor activity	194/7539	4.80E-10	5.79E-09
GO:0010464	regulation of mesenchymal cell proliferation	35/7539	4.81E-10	5.79E-09
GO:0014902	myotube differentiation	75/7539	4.90E-10	5.88E-09
GO:0060048	cardiac muscle contraction	75/7539	4.90E-10	5.88E-09
GO:0051965	positive regulation of synapse assembly	53/7539	4.96E-10	5.94E-09
GO:0071375	cellular response to peptide hormone stimulus	129/7539	5.00E-10	5.98E-09
GO:0008360	regulation of cell shape	87/7539	5.02E-10	5.99E-09
GO:0003208	cardiac ventricle morphogenesis	52/7539	5.11E-10	6.07E-09
GO:0060976	coronary vasculature development	52/7539	5.11E-10	6.07E-09
GO:0033555	multicellular organismal response to stress	63/7539	5.18E-10	6.14E-09
GO:0018209	peptidyl-serine modification	171/7539	5.18E-10	6.14E-09
GO:0007422	peripheral nervous system development	50/7539	5.33E-10	6.30E-09
GO:0036293	response to decreased oxygen levels	121/7539	5.56E-10	6.56E-09
GO:0014066	regulation of phosphatidylinositol 3-kinase signaling	62/7539	5.60E-10	6.60E-09
GO:0003179	heart valve morphogenesis	36/7539	5.80E-10	6.80E-09
GO:0003197	endocardial cushion development	36/7539	5.80E-10	6.80E-09

GO:0043524	negative regulation of neuron apoptotic process	99/7539	5.81E-10	6.81E-09
GO:0007548	sex differentiation	154/7539	5.99E-10	7.00E-09
GO:0007159	leukocyte cell-cell adhesion	166/7539	6.04E-10	7.04E-09
GO:0099072	regulation of postsynaptic membrane neurotransmitter receptor levels	61/7539	6.04E-10	7.04E-09
GO:0051896	regulation of protein kinase B signaling	92/7539	6.38E-10	7.41E-09
GO:0009799	specification of symmetry	81/7539	6.57E-10	7.63E-09
GO:0022037	metencephalon development	72/7539	6.74E-10	7.81E-09
GO:0002028	regulation of sodium ion transport	59/7539	6.97E-10	8.06E-09
GO:0048704	embryonic skeletal system morphogenesis	66/7539	7.11E-10	8.21E-09
GO:0007631	feeding behavior	76/7539	7.24E-10	8.34E-09
GO:0051494	negative regulation of cytoskeleton organization	94/7539	7.48E-10	8.60E-09
GO:0032330	regulation of chondrocyte differentiation	38/7539	7.71E-10	8.85E-09
GO:0050863	regulation of T cell activation	156/7539	8.02E-10	9.19E-09
GO:0010038	response to metal ion	137/7539	8.05E-10	9.20E-09
GO:0035418	protein localization to synapse	63/7539	9.28E-10	1.06E-08
GO:0051656	establishment of organelle localization	180/7539	9.33E-10	1.06E-08
GO:0046580	negative regulation of Ras protein signal transduction	40/7539	9.36E-10	1.06E-08
GO:0001709	cell fate determination	33/7539	9.48E-10	1.08E-08

GO:0003170	heart valve development	41/7539	1.00E-09	1.14E-08
GO:0007528	neuromuscular junction development	42/7539	1.06E-09	1.20E-08
GO:0046328	regulation of JNK cascade	100/7539	1.12E-09	1.27E-08
GO:0002090	regulation of receptor internalization	49/7539	1.12E-09	1.27E-08
GO:0007189	adenylate cyclase-activating G protein-coupled receptor signaling pathway	80/7539	1.18E-09	1.33E-08
GO:0009855	determination of bilateral symmetry	80/7539	1.18E-09	1.33E-08
GO:0048846	axon extension involved in axon guidance	30/7539	1.39E-09	1.56E-08
GO:1902284	neuron projection extension involved in neuron projection guidance	30/7539	1.39E-09	1.56E-08
GO:0014020	primary neural tube formation	70/7539	1.40E-09	1.56E-08
GO:0009953	dorsal/ventral pattern formation	64/7539	1.49E-09	1.66E-08
GO:0035050	embryonic heart tube development	55/7539	1.69E-09	1.89E-08
GO:0031099	regeneration	84/7539	1.77E-09	1.97E-08
GO:0060491	regulation of cell projection assembly	102/7539	1.83E-09	2.03E-08
GO:0006898	receptor-mediated endocytosis	122/7539	1.85E-09	2.05E-08
GO:0045137	development of primary sexual characteristics	122/7539	1.85E-09	2.05E-08
GO:1903037	regulation of leukocyte cell-cell adhesion	150/7539	1.86E-09	2.05E-08
GO:0030217	T cell differentiation	139/7539	2.04E-09	2.25E-08
GO:0046620	regulation of organ growth	71/7539	2.08E-09	2.29E-08

GO:0008344	adult locomotory behavior	66/7539	2.11E-09	2.32E-08
GO:0045687	positive regulation of glial cell differentiation	39/7539	2.12E-09	2.33E-08
GO:0009612	response to mechanical stimulus	95/7539	2.20E-09	2.40E-08
GO:0048486	parasympathetic nervous system development	20/7539	2.21E-09	2.41E-08
GO:0051492	regulation of stress fiber assembly	59/7539	2.32E-09	2.53E-08
GO:0001736	establishment of planar polarity	41/7539	2.35E-09	2.56E-08
GO:0051592	response to calcium ion	74/7539	2.41E-09	2.62E-08
GO:0003143	embryonic heart tube morphogenesis	47/7539	2.42E-09	2.62E-08
GO:0008406	gonad development	120/7539	2.45E-09	2.66E-08
GO:0050771	negative regulation of axonogenesis	46/7539	2.46E-09	2.66E-08
GO:0032835	glomerulus development	43/7539	2.47E-09	2.66E-08
GO:0046850	regulation of bone remodeling	43/7539	2.47E-09	2.66E-08
GO:0001658	branching involved in ureteric bud morphogenesis	44/7539	2.49E-09	2.68E-08
GO:0030098	lymphocyte differentiation	189/7539	2.50E-09	2.69E-08
GO:0001890	placenta development	94/7539	2.51E-09	2.69E-08
GO:1902905	positive regulation of supramolecular fiber organization	105/7539	2.53E-09	2.71E-08
GO:0048644	muscle organ morphogenesis	57/7539	2.72E-09	2.91E-08
GO:1901381	positive regulation of potassium ion transmembrane transport	33/7539	2.81E-09	2.99E-08

GO:0060326	cell chemotaxis	147/7539	2.82E-09	3.00E-08
GO:0003148	outflow tract septum morphogenesis	25/7539	2.86E-09	3.04E-08
GO:0051147	regulation of muscle cell differentiation	90/7539	2.87E-09	3.05E-08
GO:0043434	response to peptide hormone	160/7539	3.11E-09	3.30E-08
GO:0048511	rhythmic process	146/7539	3.25E-09	3.43E-08
GO:0046330	positive regulation of JNK cascade	79/7539	3.32E-09	3.50E-08
GO:1904018	positive regulation of vasculature development	108/7539	3.40E-09	3.58E-08
GO:0090101	negative regulation of transmembrane receptor protein serine/threonine kinase signaling pathway	71/7539	3.40E-09	3.58E-08
GO:0007368	determination of left/right symmetry	75/7539	3.43E-09	3.60E-08
GO:0010876	lipid localization	190/7539	3.50E-09	3.67E-08
GO:0008045	motor neuron axon guidance	29/7539	3.52E-09	3.68E-08
GO:0051495	positive regulation of cytoskeleton organization	115/7539	3.55E-09	3.71E-08
GO:0001843	neural tube closure	66/7539	3.57E-09	3.73E-08
GO:1904427	positive regulation of calcium ion transmembrane transport	53/7539	3.62E-09	3.77E-08
GO:0032869	cellular response to insulin stimulus	105/7539	3.62E-09	3.77E-08
GO:0097756	negative regulation of blood vessel diameter	65/7539	3.97E-09	4.12E-08
GO:0007157	heterophilic cell-cell adhesion via plasma membrane cell adhesion molecules	36/7539	4.13E-09	4.29E-08
GO:1990845	adaptive thermogenesis	87/7539	4.27E-09	4.42E-08

GO:0030901	midbrain development	37/7539	4.49E-09	4.63E-08
GO:0072132	mesenchyme morphogenesis	37/7539	4.49E-09	4.63E-08
GO:0070252	actin-mediated cell contraction	58/7539	4.50E-09	4.64E-08
GO:0051145	smooth muscle cell differentiation	49/7539	4.52E-09	4.65E-08
GO:0098810	neurotransmitter reuptake	26/7539	4.54E-09	4.66E-08
GO:0035725	sodium ion transmembrane transport	80/7539	4.55E-09	4.66E-08
GO:0097120	receptor localization to synapse	48/7539	4.72E-09	4.83E-08
GO:0002792	negative regulation of peptide secretion	68/7539	4.78E-09	4.87E-08
GO:0098773	skin epidermis development	68/7539	4.78E-09	4.87E-08
GO:0014909	smooth muscle cell migration	57/7539	4.90E-09	4.99E-08
GO:0120032	regulation of plasma membrane bounded cell projection assembly	100/7539	5.04E-09	5.13E-08
GO:0035567	non-canonical Wnt signaling pathway	40/7539	5.19E-09	5.26E-08
GO:0043254	regulation of protein-containing complex assembly	199/7539	5.22E-09	5.28E-08
GO:0007164	establishment of tissue polarity	41/7539	5.30E-09	5.36E-08
GO:0019226	transmission of nerve impulse	56/7539	5.33E-09	5.38E-08
GO:0010595	positive regulation of endothelial cell migration	62/7539	5.39E-09	5.43E-08
GO:0097529	myeloid leukocyte migration	112/7539	5.41E-09	5.45E-08
GO:0051261	protein depolymerization	71/7539	5.51E-09	5.54E-08

GO:0001504	neurotransmitter uptake	31/7539	5.64E-09	5.65E-08
GO:0040019	positive regulation of embryonic development	31/7539	5.64E-09	5.65E-08
GO:0055007	cardiac muscle cell differentiation	78/7539	5.86E-09	5.86E-08
GO:0060606	tube closure	66/7539	5.96E-09	5.95E-08
GO:0001659	temperature homeostasis	96/7539	6.04E-09	6.03E-08
GO:0018105	peptidyl-serine phosphorylation	157/7539	6.13E-09	6.10E-08
GO:1901652	response to peptide	184/7539	6.22E-09	6.18E-08
GO:0097061	dendritic spine organization	60/7539	6.56E-09	6.51E-08
GO:0002027	regulation of heart rate	59/7539	7.22E-09	7.15E-08
GO:0055017	cardiac muscle tissue growth	64/7539	7.40E-09	7.32E-08
GO:1901653	cellular response to peptide	144/7539	7.48E-09	7.39E-08
GO:0098698	postsynaptic specialization assembly	24/7539	7.66E-09	7.56E-08
GO:1903523	negative regulation of blood circulation	33/7539	7.73E-09	7.61E-08
GO:0048167	regulation of synaptic plasticity	170/7539	7.75E-09	7.63E-08
GO:0060563	neuroepithelial cell differentiation	50/7539	8.23E-09	8.06E-08
GO:0035282	segmentation	63/7539	8.23E-09	8.06E-08
GO:0110110	positive regulation of animal organ morphogenesis	63/7539	8.23E-09	8.06E-08
GO:0032868	response to insulin	118/7539	8.51E-09	8.32E-08

GO:0046849	bone remodeling	62/7539	9.14E-09	8.92E-08
GO:0031623	receptor internalization	74/7539	9.66E-09	9.42E-08
GO:0001942	hair follicle development	66/7539	9.81E-09	9.54E-08
GO:0050848	regulation of calcium-mediated signaling	61/7539	1.01E-08	9.80E-08
GO:0050885	neuromuscular process controlling balance	46/7539	1.01E-08	9.80E-08
GO:0021545	cranial nerve development	36/7539	1.02E-08	9.80E-08
GO:0070169	positive regulation of biomineral tissue development	36/7539	1.02E-08	9.80E-08
GO:0110151	positive regulation of biomineralization	36/7539	1.02E-08	9.80E-08
GO:2001222	regulation of neuron migration	38/7539	1.11E-08	1.07E-07
GO:0035924	cellular response to vascular endothelial growth factor stimulus	39/7539	1.14E-08	1.09E-07
GO:0021575	hindbrain morphogenesis	41/7539	1.15E-08	1.10E-07
GO:0050796	regulation of insulin secretion	99/7539	1.19E-08	1.14E-07
GO:0051668	localization within membrane	64/7539	1.23E-08	1.18E-07
GO:0090090	negative regulation of canonical Wnt signaling pathway	72/7539	1.24E-08	1.18E-07
GO:0030279	negative regulation of ossification	59/7539	1.24E-08	1.19E-07
GO:1904019	epithelial cell apoptotic process	68/7539	1.26E-08	1.20E-07
GO:0045862	positive regulation of proteolysis	161/7539	1.26E-08	1.20E-07
GO:0060415	muscle tissue morphogenesis	52/7539	1.33E-08	1.26E-07

GO:0045766	positive regulation of angiogenesis	98/7539	1.36E-08	1.29E-07
GO:0061053	somite development	58/7539	1.37E-08	1.30E-07
GO:0002685	regulation of leukocyte migration	110/7539	1.38E-08	1.31E-07
GO:0022404	molting cycle process	67/7539	1.42E-08	1.34E-07
GO:0022405	hair cycle process	67/7539	1.42E-08	1.34E-07
GO:0043535	regulation of blood vessel endothelial cell migration	57/7539	1.51E-08	1.43E-07
GO:0030307	positive regulation of cell growth	102/7539	1.56E-08	1.47E-07
GO:0042310	vasoconstriction	61/7539	1.70E-08	1.60E-07
GO:0090263	positive regulation of canonical Wnt signaling pathway	61/7539	1.70E-08	1.60E-07
GO:2000300	regulation of synaptic vesicle exocytosis	61/7539	1.70E-08	1.60E-07
GO:0048872	homeostasis of number of cells	155/7539	1.74E-08	1.63E-07
GO:0038083	peptidyl-tyrosine autophosphorylation	32/7539	1.81E-08	1.69E-07
GO:0007632	visual behavior	47/7539	1.87E-08	1.74E-07
GO:0021885	forebrain cell migration	46/7539	1.98E-08	1.84E-07
GO:0043113	receptor clustering	46/7539	1.98E-08	1.84E-07
GO:0021871	forebrain regionalization	23/7539	2.04E-08	1.89E-07
GO:0048799	animal organ maturation	23/7539	2.04E-08	1.89E-07
GO:0048168	regulation of neuronal synaptic plasticity	45/7539	2.08E-08	1.93E-07

GO:0046847	filopodium assembly	44/7539	2.18E-08	2.01E-07
GO:0060425	lung morphogenesis	44/7539	2.18E-08	2.01E-07
GO:0086003	cardiac muscle cell contraction	44/7539	2.18E-08	2.01E-07
GO:0031646	positive regulation of nervous system process	53/7539	2.19E-08	2.01E-07
GO:0099068	postsynapse assembly	27/7539	2.20E-08	2.02E-07
GO:0045446	endothelial cell differentiation	63/7539	2.26E-08	2.07E-07
GO:0048661	positive regulation of smooth muscle cell proliferation	63/7539	2.26E-08	2.07E-07
GO:0001947	heart looping	42/7539	2.35E-08	2.15E-07
GO:0099054	presynapse assembly	36/7539	2.38E-08	2.17E-07
GO:0022408	negative regulation of cell-cell adhesion	99/7539	2.38E-08	2.17E-07
GO:1901863	positive regulation of muscle tissue development	52/7539	2.39E-08	2.17E-07
GO:0010812	negative regulation of cell-substrate adhesion	41/7539	2.41E-08	2.19E-07
GO:0002042	cell migration involved in sprouting angiogenesis	38/7539	2.47E-08	2.24E-07
GO:0051153	regulation of striated muscle cell differentiation	70/7539	2.49E-08	2.26E-07
GO:0106027	neuron projection organization	62/7539	2.53E-08	2.29E-07
GO:0034599	cellular response to oxidative stress	133/7539	2.63E-08	2.38E-07
GO:0003158	endothelium development	69/7539	2.82E-08	2.54E-07
GO:0045807	positive regulation of endocytosis	69/7539	2.82E-08	2.54E-07

GO:0009100	glycoprotein metabolic process	155/7539	2.87E-08	2.58E-07
GO:0001938	positive regulation of endothelial cell proliferation	55/7539	3.17E-08	2.85E-07
GO:1903524	positive regulation of blood circulation	55/7539	3.17E-08	2.85E-07
GO:0043586	tongue development	21/7539	3.29E-08	2.95E-07
GO:1901184	regulation of ERBB signaling pathway	47/7539	3.52E-08	3.15E-07
GO:0050795	regulation of behavior	59/7539	3.53E-08	3.15E-07
GO:0060419	heart growth	67/7539	3.61E-08	3.22E-07
GO:0021549	cerebellum development	63/7539	3.66E-08	3.27E-07
GO:0014046	dopamine secretion	30/7539	3.76E-08	3.33E-07
GO:0014059	regulation of dopamine secretion	30/7539	3.76E-08	3.33E-07
GO:0031076	embryonic camera-type eye development	30/7539	3.76E-08	3.33E-07
GO:1901021	positive regulation of calcium ion transmembrane transporter activity	30/7539	3.76E-08	3.33E-07
GO:1902692	regulation of neuroblast proliferation	30/7539	3.76E-08	3.33E-07
GO:1904888	cranial skeletal system development	46/7539	3.76E-08	3.33E-07
GO:0045669	positive regulation of osteoblast differentiation	45/7539	4.01E-08	3.54E-07
GO:0098693	regulation of synaptic vesicle cycle	79/7539	4.14E-08	3.65E-07
GO:0014910	regulation of smooth muscle cell migration	52/7539	4.22E-08	3.71E-07
GO:0043949	regulation of cAMP-mediated signaling	44/7539	4.25E-08	3.73E-07

GO:2001236	regulation of extrinsic apoptotic signaling pathway	87/7539	4.36E-08	3.83E-07
GO:0045823	positive regulation of heart contraction	32/7539	4.57E-08	4.00E-07
GO:0045844	positive regulation of striated muscle tissue development	51/7539	4.62E-08	4.04E-07
GO:0048636	positive regulation of muscle organ development	51/7539	4.62E-08	4.04E-07
GO:0090278	negative regulation of peptide hormone secretion	42/7539	4.69E-08	4.10E-07
GO:0097107	postsynaptic density assembly	19/7539	4.75E-08	4.15E-07
GO:0061098	positive regulation of protein tyrosine kinase activity	33/7539	4.86E-08	4.24E-07
GO:0021517	ventral spinal cord development	40/7539	5.06E-08	4.40E-07
GO:0055024	regulation of cardiac muscle tissue development	60/7539	5.19E-08	4.50E-07
GO:0099172	presynapse organization	39/7539	5.19E-08	4.50E-07
GO:0021988	olfactory lobe development	35/7539	5.24E-08	4.53E-07
GO:0061005	cell differentiation involved in kidney development	35/7539	5.24E-08	4.53E-07
GO:0044060	regulation of endocrine process	38/7539	5.29E-08	4.57E-07
GO:0045124	regulation of bone resorption	36/7539	5.32E-08	4.58E-07
GO:0140353	lipid export from cell	36/7539	5.32E-08	4.58E-07
GO:0045619	regulation of lymphocyte differentiation	98/7539	5.37E-08	4.62E-07
GO:0048791	calcium ion-regulated exocytosis of neurotransmitter	22/7539	5.41E-08	4.65E-07
GO:0048566	embryonic digestive tract development	26/7539	5.44E-08	4.65E-07

GO:0048841	regulation of axon extension involved in axon guidance	26/7539	5.44E-08	4.65E-07
GO:1901186	positive regulation of ERBB signaling pathway	26/7539	5.44E-08	4.65E-07
GO:0021514	ventral spinal cord interneuron differentiation	17/7539	5.66E-08	4.83E-07
GO:0021783	preganglionic parasympathetic fiber development	17/7539	5.66E-08	4.83E-07
GO:0043409	negative regulation of MAPK cascade	90/7539	5.90E-08	5.03E-07
GO:0061097	regulation of protein tyrosine kinase activity	48/7539	5.98E-08	5.09E-07
GO:0006469	negative regulation of protein kinase activity	104/7539	6.10E-08	5.18E-07
GO:0007043	cell-cell junction assembly	79/7539	6.13E-08	5.21E-07
GO:2001233	regulation of apoptotic signaling pathway	185/7539	6.31E-08	5.35E-07
GO:0043279	response to alkaloid	47/7539	6.48E-08	5.48E-07
GO:0035914	skeletal muscle cell differentiation	53/7539	6.63E-08	5.59E-07
GO:1904063	negative regulation of cation transmembrane transport	53/7539	6.63E-08	5.59E-07
GO:0035116	embryonic hindlimb morphogenesis	27/7539	6.64E-08	5.59E-07
GO:0045987	positive regulation of smooth muscle contraction	27/7539	6.64E-08	5.59E-07
GO:0014068	positive regulation of phosphatidylinositol 3-kinase signaling	46/7539	6.99E-08	5.88E-07
GO:0007018	microtubule-based movement	174/7539	7.31E-08	6.13E-07
GO:0007435	salivary gland morphogenesis	28/7539	7.77E-08	6.52E-07
GO:1902668	negative regulation of axon guidance	23/7539	7.91E-08	6.62E-07

GO:1904861	excitatory synapse assembly	23/7539	7.91E-08	6.62E-07
GO:0008542	visual learning	44/7539	8.05E-08	6.71E-07
GO:0048678	response to axon injury	44/7539	8.05E-08	6.71E-07
GO:0061371	determination of heart left/right asymmetry	44/7539	8.05E-08	6.71E-07
GO:0014074	response to purine-containing compound	60/7539	8.41E-08	6.98E-07
GO:0034766	negative regulation of ion transmembrane transport	60/7539	8.41E-08	6.98E-07
GO:0048485	sympathetic nervous system development	20/7539	8.95E-08	7.43E-07
GO:0097191	extrinsic apoptotic signaling pathway	114/7539	9.16E-08	7.59E-07
GO:0007431	salivary gland development	30/7539	9.66E-08	7.99E-07
GO:0048286	lung alveolus development	40/7539	1.01E-07	8.34E-07
GO:0060986	endocrine hormone secretion	40/7539	1.01E-07	8.34E-07
GO:1905952	regulation of lipid localization	86/7539	1.03E-07	8.49E-07
GO:0051209	release of sequestered calcium ion into cytosol	62/7539	1.05E-07	8.65E-07
GO:0051897	positive regulation of protein kinase B signaling	62/7539	1.05E-07	8.65E-07
GO:0042551	neuron maturation	39/7539	1.05E-07	8.65E-07
GO:0034763	negative regulation of transmembrane transport	72/7539	1.06E-07	8.67E-07
GO:0021602	cranial nerve morphogenesis	24/7539	1.06E-07	8.69E-07
GO:0019229	regulation of vasoconstriction	48/7539	1.07E-07	8.73E-07

GO:0035136	forelimb morphogenesis	32/7539	1.09E-07	8.88E-07
GO:0046622	positive regulation of organ growth	38/7539	1.09E-07	8.88E-07
GO:0051931	regulation of sensory perception	38/7539	1.09E-07	8.88E-07
GO:2000648	positive regulation of stem cell proliferation	38/7539	1.09E-07	8.88E-07
GO:0050922	negative regulation of chemotaxis	37/7539	1.12E-07	9.09E-07
GO:0086001	cardiac muscle cell action potential	37/7539	1.12E-07	9.09E-07
GO:0001941	postsynaptic membrane organization	33/7539	1.13E-07	9.11E-07
GO:0030517	negative regulation of axon extension	33/7539	1.13E-07	9.11E-07
GO:0035137	hindlimb morphogenesis	35/7539	1.15E-07	9.27E-07
GO:0060412	ventricular septum morphogenesis	35/7539	1.15E-07	9.27E-07
GO:0060428	lung epithelium development	35/7539	1.15E-07	9.27E-07
GO:0106106	cold-induced thermogenesis	77/7539	1.18E-07	9.52E-07
GO:0120161	regulation of cold-induced thermogenesis	77/7539	1.18E-07	9.52E-07
GO:0050709	negative regulation of protein secretion	61/7539	1.19E-07	9.57E-07
GO:1903708	positive regulation of hemopoiesis	110/7539	1.20E-07	9.62E-07
GO:0001892	embryonic placenta development	64/7539	1.29E-07	1.03E-06
GO:0040036	regulation of fibroblast growth factor receptor signaling pathway	25/7539	1.34E-07	1.07E-06
GO:0007623	circadian rhythm	107/7539	1.36E-07	1.09E-06

GO:0007218	neuropeptide signaling pathway	51/7539	1.39E-07	1.11E-06
GO:0033673	negative regulation of kinase activity	111/7539	1.40E-07	1.11E-06
GO:0098739	import across plasma membrane	86/7539	1.46E-07	1.17E-06
GO:0055008	cardiac muscle tissue morphogenesis	44/7539	1.49E-07	1.18E-06
GO:0055117	regulation of cardiac muscle contraction	44/7539	1.49E-07	1.18E-06
GO:0010522	regulation of calcium ion transport into cytosol	59/7539	1.52E-07	1.20E-06
GO:0051899	membrane depolarization	50/7539	1.53E-07	1.21E-06
GO:0000187	activation of MAPK activity	66/7539	1.54E-07	1.21E-06
GO:0048013	ephrin receptor signaling pathway	26/7539	1.60E-07	1.26E-06
GO:1905606	regulation of presynapse assembly	26/7539	1.60E-07	1.26E-06
GO:0022029	telencephalon cell migration	43/7539	1.61E-07	1.27E-06
GO:0009913	epidermal cell differentiation	97/7539	1.63E-07	1.29E-06
GO:0051283	negative regulation of sequestering of calcium ion	62/7539	1.66E-07	1.30E-06
GO:0048814	regulation of dendrite morphogenesis	65/7539	1.75E-07	1.37E-06
GO:1904950	negative regulation of establishment of protein localization	87/7539	1.80E-07	1.41E-06
GO:0043500	muscle adaptation	68/7539	1.80E-07	1.41E-06
GO:0048008	platelet-derived growth factor receptor signaling pathway	41/7539	1.84E-07	1.44E-06
GO:0062237	protein localization to postsynapse	41/7539	1.84E-07	1.44E-06

GO:0015718	monocarboxylic acid transport	89/7539	1.90E-07	1.48E-06
GO:0043502	regulation of muscle adaptation	57/7539	1.93E-07	1.50E-06
GO:0006865	amino acid transport	84/7539	1.94E-07	1.51E-06
GO:0031102	neuron projection regeneration	40/7539	1.96E-07	1.52E-06
GO:0090100	positive regulation of transmembrane receptor protein serine/threonine kinase signaling pathway	64/7539	1.99E-07	1.54E-06
GO:0048843	negative regulation of axon extension involved in axon guidance	22/7539	2.02E-07	1.57E-06
GO:0038084	vascular endothelial growth factor signaling pathway	28/7539	2.04E-07	1.58E-06
GO:0072210	metanephric nephron development	28/7539	2.04E-07	1.58E-06
GO:0099174	regulation of presynapse organization	28/7539	2.04E-07	1.58E-06
GO:0048844	artery morphogenesis	47/7539	2.06E-07	1.59E-06
GO:0035019	somatic stem cell population maintenance	38/7539	2.18E-07	1.68E-06
GO:0071542	dopaminergic neuron differentiation	29/7539	2.21E-07	1.70E-06
GO:0090175	regulation of establishment of planar polarity	29/7539	2.21E-07	1.70E-06
GO:0003014	renal system process	63/7539	2.26E-07	1.74E-06
GO:0001954	positive regulation of cell-matrix adhesion	37/7539	2.27E-07	1.74E-06
GO:0021587	cerebellum morphogenesis	37/7539	2.27E-07	1.74E-06
GO:0048538	thymus development	37/7539	2.27E-07	1.74E-06
GO:0051930	regulation of sensory perception of pain	37/7539	2.27E-07	1.74E-06

GO:0014014	negative regulation of gliogenesis	36/7539	2.36E-07	1.80E-06
GO:0048854	brain morphogenesis	31/7539	2.42E-07	1.84E-06
GO:0021516	dorsal spinal cord development	19/7539	2.42E-07	1.84E-06
GO:0048536	spleen development	32/7539	2.47E-07	1.88E-06
GO:0048010	vascular endothelial growth factor receptor signaling pathway	33/7539	2.48E-07	1.88E-06
GO:0006942	regulation of striated muscle contraction	50/7539	2.60E-07	1.97E-06
GO:0042733	embryonic digit morphogenesis	44/7539	2.69E-07	2.04E-06
GO:0000302	response to reactive oxygen species	100/7539	2.70E-07	2.05E-06
GO:0043624	cellular protein complex disassembly	76/7539	2.88E-07	2.17E-06
GO:0014896	muscle hypertrophy	61/7539	2.92E-07	2.21E-06
GO:0033627	cell adhesion mediated by integrin	43/7539	2.93E-07	2.21E-06
GO:0050921	positive regulation of chemotaxis	78/7539	3.14E-07	2.36E-06
GO:0060688	regulation of morphogenesis of a branching structure	42/7539	3.18E-07	2.39E-06
GO:0002092	positive regulation of receptor internalization	24/7539	3.25E-07	2.44E-06
GO:0045742	positive regulation of epidermal growth factor receptor signaling pathway	24/7539	3.25E-07	2.44E-06
GO:0014897	striated muscle hypertrophy	60/7539	3.32E-07	2.49E-06
GO:0046631	alpha-beta T cell activation	85/7539	3.33E-07	2.50E-06
GO:0032233	positive regulation of actin filament bundle assembly	41/7539	3.43E-07	2.57E-06

GO:0016311	dephosphorylation	167/7539	3.47E-07	2.59E-06
GO:0060038	cardiac muscle cell proliferation	40/7539	3.69E-07	2.75E-06
GO:0042474	middle ear morphogenesis	20/7539	3.73E-07	2.77E-06
GO:0062009	secondary palate development	20/7539	3.73E-07	2.77E-06
GO:0040037	negative regulation of fibroblast growth factor receptor signaling pathway	17/7539	3.74E-07	2.77E-06
GO:0098962	regulation of postsynaptic neurotransmitter receptor activity	17/7539	3.74E-07	2.77E-06
GO:1905874	regulation of postsynaptic density organization	17/7539	3.74E-07	2.77E-06
GO:0021884	forebrain neuron development	25/7539	3.81E-07	2.82E-06
GO:0046887	positive regulation of hormone secretion	84/7539	3.84E-07	2.84E-06
GO:0051282	regulation of sequestering of calcium ion	62/7539	3.95E-07	2.92E-06
GO:0045776	negative regulation of blood pressure	39/7539	3.95E-07	2.92E-06
GO:1903539	protein localization to postsynaptic membrane	38/7539	4.21E-07	3.11E-06
GO:0060292	long-term synaptic depression	26/7539	4.29E-07	3.16E-06
GO:0006029	proteoglycan metabolic process	45/7539	4.33E-07	3.19E-06
GO:0010639	negative regulation of organelle organization	184/7539	4.46E-07	3.27E-06
GO:0140115	export across plasma membrane	37/7539	4.46E-07	3.27E-06
GO:0002053	positive regulation of mesenchymal cell proliferation	27/7539	4.69E-07	3.43E-06
GO:0060071	Wnt signaling pathway, planar cell polarity pathway	27/7539	4.69E-07	3.43E-06

GO:0021978	telencephalon regionalization	15/7539	4.84E-07	3.53E-06
GO:0060579	ventral spinal cord interneuron fate commitment	15/7539	4.84E-07	3.53E-06
GO:0060581	cell fate commitment involved in pattern specification	15/7539	4.84E-07	3.53E-06
GO:0061548	ganglion development	15/7539	4.84E-07	3.53E-06
GO:0055006	cardiac cell development	57/7539	4.85E-07	3.53E-06
GO:0030514	negative regulation of BMP signaling pathway	35/7539	4.91E-07	3.57E-06
GO:0048701	embryonic cranial skeleton morphogenesis	35/7539	4.91E-07	3.57E-06
GO:2000050	regulation of non-canonical Wnt signaling pathway	21/7539	5.14E-07	3.72E-06
GO:2000311	regulation of AMPA receptor activity	21/7539	5.14E-07	3.72E-06
GO:0086009	membrane repolarization	29/7539	5.20E-07	3.77E-06
GO:0021772	olfactory bulb development	32/7539	5.33E-07	3.85E-06
GO:0021696	cerebellar cortex morphogenesis	31/7539	5.36E-07	3.86E-06
GO:0050918	positive chemotaxis	31/7539	5.36E-07	3.86E-06
GO:0071526	semaphorin-plexin signaling pathway	31/7539	5.36E-07	3.86E-06
GO:0090184	positive regulation of kidney development	31/7539	5.36E-07	3.86E-06
GO:1902743	regulation of lamellipodium organization	31/7539	5.36E-07	3.86E-06
GO:0061041	regulation of wound healing	76/7539	5.92E-07	4.25E-06
GO:0051208	sequestering of calcium ion	62/7539	6.00E-07	4.31E-06

GO:0002043	blood vessel endothelial cell proliferation involved in sprouting angiogenesis	18/7539	6.50E-07	4.66E-06
GO:0048596	embryonic camera-type eye morphogenesis	22/7539	6.54E-07	4.67E-06
GO:0060039	pericardium development	22/7539	6.54E-07	4.67E-06
GO:1903671	negative regulation of sprouting angiogenesis	22/7539	6.54E-07	4.67E-06
GO:1903829	positive regulation of cellular protein localization	144/7539	6.54E-07	4.67E-06
GO:0032410	negative regulation of transporter activity	46/7539	6.70E-07	4.76E-06
GO:0045453	bone resorption	46/7539	6.70E-07	4.76E-06
GO:0071277	cellular response to calcium ion	46/7539	6.70E-07	4.76E-06
GO:0071248	cellular response to metal ion	80/7539	6.73E-07	4.78E-06
GO:0008347	glial cell migration	40/7539	6.77E-07	4.80E-06
GO:0035904	aorta development	40/7539	6.77E-07	4.80E-06
GO:0007229	integrin-mediated signaling pathway	54/7539	7.05E-07	4.99E-06
GO:0010171	body morphogenesis	39/7539	7.33E-07	5.18E-06
GO:0014743	regulation of muscle hypertrophy	45/7539	7.44E-07	5.24E-06
GO:0034109	homotypic cell-cell adhesion	45/7539	7.44E-07	5.24E-06
GO:0048747	muscle fiber development	45/7539	7.44E-07	5.24E-06
GO:0051057	positive regulation of small GTPase mediated signal transduction	45/7539	7.44E-07	5.24E-06
GO:0032368	regulation of lipid transport	69/7539	7.56E-07	5.31E-06

GO:1900006	positive regulation of dendrite development	60/7539	7.82E-07	5.49E-06
GO:1905477	positive regulation of protein localization to membrane	60/7539	7.82E-07	5.49E-06
GO:0051155	positive regulation of striated muscle cell differentiation	44/7539	8.24E-07	5.77E-06
GO:0051224	negative regulation of protein transport	83/7539	8.54E-07	5.98E-06
GO:0051590	positive regulation of neurotransmitter transport	24/7539	9.01E-07	6.29E-06
GO:0050806	positive regulation of synaptic transmission	158/7539	9.01E-07	6.29E-06
GO:0032890	regulation of organic acid transport	52/7539	9.02E-07	6.29E-06
GO:0051954	positive regulation of amine transport	35/7539	9.62E-07	6.70E-06
GO:0070977	bone maturation	19/7539	9.67E-07	6.73E-06
GO:0045637	regulation of myeloid cell differentiation	107/7539	9.83E-07	6.83E-06
GO:0030595	leukocyte chemotaxis	105/7539	9.91E-07	6.88E-06
GO:0001964	startle response	25/7539	9.96E-07	6.88E-06
GO:0003203	endocardial cushion morphogenesis	25/7539	9.96E-07	6.88E-06
GO:0003209	cardiac atrium morphogenesis	25/7539	9.96E-07	6.88E-06
GO:0048048	embryonic eye morphogenesis	25/7539	9.96E-07	6.88E-06
GO:0042058	regulation of epidermal growth factor receptor signaling pathway	42/7539	1.00E-06	6.94E-06
GO:0051149	positive regulation of muscle cell differentiation	51/7539	1.02E-06	7.03E-06
GO:0070571	negative regulation of neuron projection regeneration	16/7539	1.04E-06	7.15E-06

GO:0099150	regulation of postsynaptic specialization assembly	16/7539	1.04E-06	7.15E-06
GO:0150052	regulation of postsynapse assembly	16/7539	1.04E-06	7.15E-06
GO:0032232	negative regulation of actin filament bundle assembly	26/7539	1.07E-06	7.33E-06
GO:0048169	regulation of long-term neuronal synaptic plasticity	26/7539	1.07E-06	7.33E-06
GO:0051348	negative regulation of transferase activity	121/7539	1.10E-06	7.57E-06
GO:0051953	negative regulation of amine transport	27/7539	1.12E-06	7.67E-06
GO:0014855	striated muscle cell proliferation	46/7539	1.12E-06	7.68E-06
GO:1902667	regulation of axon guidance	31/7539	1.13E-06	7.75E-06
GO:0043112	receptor metabolic process	102/7539	1.15E-06	7.83E-06
GO:0009101	glycoprotein biosynthetic process	126/7539	1.15E-06	7.86E-06
GO:0045747	positive regulation of Notch signaling pathway	30/7539	1.15E-06	7.86E-06
GO:0003300	cardiac muscle hypertrophy	57/7539	1.16E-06	7.89E-06
GO:0010092	specification of animal organ identity	29/7539	1.16E-06	7.89E-06
GO:1901879	regulation of protein depolymerization	53/7539	1.26E-06	8.52E-06
GO:1904035	regulation of epithelial cell apoptotic process	53/7539	1.26E-06	8.52E-06
GO:0032886	regulation of microtubule-based process	120/7539	1.27E-06	8.57E-06
GO:0021521	ventral spinal cord interneuron specification	Dec-39	1.29E-06	8.70E-06
GO:0060573	cell fate specification involved in pattern specification	Dec-39	1.29E-06	8.70E-06

GO:0007413	axonal fasciculation	20/7539	1.29E-06	8.70E-06
GO:0021513	spinal cord dorsal/ventral patterning	20/7539	1.29E-06	8.70E-06
GO:0051894	positive regulation of focal adhesion assembly	20/7539	1.29E-06	8.70E-06
GO:0106030	neuron projection fasciculation	20/7539	1.29E-06	8.70E-06
GO:0008154	actin polymerization or depolymerization	103/7539	1.31E-06	8.81E-06
GO:0032092	positive regulation of protein binding	56/7539	1.32E-06	8.87E-06
GO:2001237	negative regulation of extrinsic apoptotic signaling pathway	56/7539	1.32E-06	8.87E-06
GO:0072074	kidney mesenchyme development	14/7539	1.41E-06	9.43E-06
GO:0098917	retrograde trans-synaptic signaling	14/7539	1.41E-06	9.43E-06
GO:0050954	sensory perception of mechanical stimulus	138/7539	1.45E-06	9.68E-06
GO:0002687	positive regulation of leukocyte migration	79/7539	1.50E-06	1.00E-05
GO:0002052	positive regulation of neuroblast proliferation	21/7539	1.60E-06	1.07E-05
GO:0015874	norepinephrine transport	21/7539	1.60E-06	1.07E-05
GO:0021511	spinal cord patterning	21/7539	1.60E-06	1.07E-05
GO:1901018	positive regulation of potassium ion transmembrane transporter activity	21/7539	1.60E-06	1.07E-05
GO:0031503	protein-containing complex localization	120/7539	1.61E-06	1.07E-05
GO:0046545	development of primary female sexual characteristics	63/7539	1.73E-06	1.15E-05
GO:0045986	negative regulation of smooth muscle contraction	17/7539	1.73E-06	1.15E-05

GO:0045580	regulation of T cell differentiation	80/7539	1.80E-06	1.19E-05
GO:0051258	protein polymerization	133/7539	1.80E-06	1.19E-05
GO:0051279	regulation of release of sequestered calcium ion into cytosol	46/7539	1.85E-06	1.22E-05
GO:0051966	regulation of synaptic transmission, glutamatergic	46/7539	1.85E-06	1.22E-05
GO:0060571	morphogenesis of an epithelial fold	22/7539	1.88E-06	1.24E-05
GO:0150117	positive regulation of cell-substrate junction organization	22/7539	1.88E-06	1.24E-05
GO:0017156	calcium-ion regulated exocytosis	41/7539	1.92E-06	1.26E-05
GO:0050727	regulation of inflammatory response	163/7539	1.94E-06	1.28E-05
GO:0031103	axon regeneration	34/7539	1.95E-06	1.28E-05
GO:0061383	trabecula morphogenesis	34/7539	1.95E-06	1.28E-05
GO:0001704	formation of primary germ layer	56/7539	2.01E-06	1.32E-05
GO:0010657	muscle cell apoptotic process	56/7539	2.01E-06	1.32E-05
GO:0030705	cytoskeleton-dependent intracellular transport	94/7539	2.02E-06	1.32E-05
GO:0042493	response to drug	120/7539	2.05E-06	1.34E-05
GO:0048708	astrocyte differentiation	49/7539	2.07E-06	1.35E-05
GO:0060043	regulation of cardiac muscle cell proliferation	33/7539	2.08E-06	1.35E-05
GO:1904375	regulation of protein localization to cell periphery	72/7539	2.08E-06	1.35E-05
GO:0002063	chondrocyte development	23/7539	2.11E-06	1.37E-05

GO:0021533	cell differentiation in hindbrain	23/7539	2.11E-06	1.37E-05
GO:0060074	synapse maturation	23/7539	2.11E-06	1.37E-05
GO:0061037	negative regulation of cartilage development	23/7539	2.11E-06	1.37E-05
GO:1901889	negative regulation of cell junction assembly	23/7539	2.11E-06	1.37E-05
GO:0032271	regulation of protein polymerization	107/7539	2.16E-06	1.40E-05
GO:0098659	inorganic cation import across plasma membrane	55/7539	2.30E-06	1.48E-05
GO:0099587	inorganic ion import across plasma membrane	55/7539	2.30E-06	1.48E-05
GO:0097755	positive regulation of blood vessel diameter	44/7539	2.33E-06	1.50E-05
GO:0006470	protein dephosphorylation	110/7539	2.37E-06	1.53E-05
GO:0003156	regulation of animal organ formation	30/7539	2.39E-06	1.54E-05
GO:2000826	regulation of heart morphogenesis	30/7539	2.39E-06	1.54E-05
GO:0045686	negative regulation of glial cell differentiation	25/7539	2.41E-06	1.55E-05
GO:0045746	negative regulation of Notch signaling pathway	25/7539	2.41E-06	1.55E-05
GO:0071320	cellular response to cAMP	25/7539	2.41E-06	1.55E-05
GO:0046486	glycerolipid metabolic process	158/7539	2.47E-06	1.58E-05
GO:0003230	cardiac atrium development	27/7539	2.52E-06	1.61E-05
GO:0035850	epithelial cell differentiation involved in kidney development	27/7539	2.52E-06	1.61E-05
GO:0014911	positive regulation of smooth muscle cell migration	38/7539	2.57E-06	1.64E-05

GO:0060350	endochondral bone morphogenesis	38/7539	2.57E-06	1.64E-05
GO:1903078	positive regulation of protein localization to plasma membrane	38/7539	2.57E-06	1.64E-05
GO:0002761	regulation of myeloid leukocyte differentiation	68/7539	2.58E-06	1.64E-05
GO:0060997	dendritic spine morphogenesis	43/7539	2.60E-06	1.66E-05
GO:1905954	positive regulation of lipid localization	57/7539	2.65E-06	1.68E-05
GO:0050680	negative regulation of epithelial cell proliferation	75/7539	2.65E-06	1.68E-05
GO:2000241	regulation of reproductive process	94/7539	2.67E-06	1.69E-05
GO:0070482	response to oxygen levels	130/7539	2.68E-06	1.70E-05
GO:0045787	positive regulation of cell cycle	157/7539	2.80E-06	1.77E-05
GO:0048260	positive regulation of receptor-mediated endocytosis	37/7539	2.82E-06	1.78E-05
GO:0060324	face development	37/7539	2.82E-06	1.78E-05
GO:0090183	regulation of kidney development	37/7539	2.82E-06	1.78E-05
GO:0003184	pulmonary valve morphogenesis	15/7539	2.87E-06	1.81E-05
GO:0048853	forebrain morphogenesis	15/7539	2.87E-06	1.81E-05
GO:1904889	regulation of excitatory synapse assembly	15/7539	2.87E-06	1.81E-05
GO:1905331	negative regulation of morphogenesis of an epithelium	15/7539	2.87E-06	1.81E-05
GO:0071901	negative regulation of protein serine/threonine kinase activity	62/7539	2.89E-06	1.82E-05
GO:0017015	regulation of transforming growth factor beta receptor signaling pathway	59/7539	2.99E-06	1.88E-05

GO:0031016	pancreas development	53/7539	2.99E-06	1.88E-05
GO:0003272	endocardial cushion formation	19/7539	3.22E-06	2.02E-05
GO:0031954	positive regulation of protein autophosphorylation	19/7539	3.22E-06	2.02E-05
GO:0009880	embryonic pattern specification	41/7539	3.25E-06	2.03E-05
GO:0045600	positive regulation of fat cell differentiation	41/7539	3.25E-06	2.03E-05
GO:1904377	positive regulation of protein localization to cell periphery	41/7539	3.25E-06	2.03E-05
GO:1903039	positive regulation of leukocyte cell-cell adhesion	104/7539	3.26E-06	2.03E-05
GO:0008585	female gonad development	61/7539	3.32E-06	2.07E-05
GO:0017145	stem cell division	35/7539	3.35E-06	2.08E-05
GO:0098815	modulation of excitatory postsynaptic potential	35/7539	3.35E-06	2.08E-05
GO:0018210	peptidyl-threonine modification	66/7539	3.41E-06	2.12E-05
GO:0051101	regulation of DNA binding	66/7539	3.41E-06	2.12E-05
GO:2000116	regulation of cysteine-type endopeptidase activity	109/7539	3.48E-06	2.15E-05
GO:0055025	positive regulation of cardiac muscle tissue development	34/7539	3.63E-06	2.25E-05
GO:0010256	endomembrane system organization	176/7539	3.67E-06	2.27E-05
GO:0051091	positive regulation of DNA-binding transcription factor activity	119/7539	3.74E-06	2.31E-05
GO:0042593	glucose homeostasis	124/7539	3.80E-06	2.34E-05
GO:0043281	regulation of cysteine-type endopeptidase activity involved in apoptotic process	99/7539	3.89E-06	2.40E-05

GO:0035710	CD4-positive, alpha-beta T cell activation	57/7539	3.93E-06	2.42E-05
GO:0051610	serotonin uptake	Nov-39	4.00E-06	2.46E-05
GO:0072075	metanephric mesenchyme development	Nov-39	4.00E-06	2.46E-05
GO:0014831	gastro-intestinal system smooth muscle contraction	13/7539	4.08E-06	2.50E-05
GO:0021527	spinal cord association neuron differentiation	13/7539	4.08E-06	2.50E-05
GO:0099151	regulation of postsynaptic density assembly	13/7539	4.08E-06	2.50E-05
GO:2000052	positive regulation of non-canonical Wnt signaling pathway	13/7539	4.08E-06	2.50E-05
GO:0046632	alpha-beta T cell differentiation	62/7539	4.18E-06	2.56E-05
GO:0070509	calcium ion import	50/7539	4.44E-06	2.70E-05
GO:1903035	negative regulation of response to wounding	50/7539	4.44E-06	2.70E-05
GO:0002089	lens morphogenesis in camera-type eye	21/7539	4.45E-06	2.70E-05
GO:0030878	thyroid gland development	21/7539	4.45E-06	2.70E-05
GO:0060384	innervation	21/7539	4.45E-06	2.70E-05
GO:0060445	branching involved in salivary gland morphogenesis	21/7539	4.45E-06	2.70E-05
GO:2000310	regulation of NMDA receptor activity	21/7539	4.45E-06	2.70E-05
GO:0060999	positive regulation of dendritic spine development	38/7539	4.46E-06	2.71E-05
GO:0050886	endocrine process	56/7539	4.50E-06	2.73E-05
GO:0120162	positive regulation of cold-induced thermogenesis	53/7539	4.53E-06	2.75E-05

GO:0062012	regulation of small molecule metabolic process	156/7539	4.67E-06	2.83E-05
GO:0033500	carbohydrate homeostasis	124/7539	4.75E-06	2.87E-05
GO:0017158	regulation of calcium ion-dependent exocytosis	30/7539	4.75E-06	2.87E-05
GO:0019228	neuronal action potential	30/7539	4.75E-06	2.87E-05
GO:0010611	regulation of cardiac muscle hypertrophy	42/7539	4.81E-06	2.90E-05
GO:0045123	cellular extravasation	42/7539	4.81E-06	2.90E-05
GO:0001945	lymph vessel development	22/7539	4.89E-06	2.94E-05
GO:0048714	positive regulation of oligodendrocyte differentiation	22/7539	4.89E-06	2.94E-05
GO:0014904	myotube cell development	29/7539	4.99E-06	2.99E-05
GO:0043114	regulation of vascular permeability	29/7539	4.99E-06	2.99E-05
GO:0048144	fibroblast proliferation	58/7539	5.03E-06	3.01E-05
GO:0050866	negative regulation of cell activation	99/7539	5.03E-06	3.02E-05
GO:0055013	cardiac muscle cell development	52/7539	5.18E-06	3.10E-05
GO:0010765	positive regulation of sodium ion transport	28/7539	5.19E-06	3.10E-05
GO:0060977	coronary vasculature morphogenesis	23/7539	5.19E-06	3.10E-05
GO:0070570	regulation of neuron projection regeneration	27/7539	5.35E-06	3.19E-05
GO:0099633	protein localization to postsynaptic specialization membrane	24/7539	5.38E-06	3.20E-05
GO:0099645	neurotransmitter receptor localization to postsynaptic specialization membrane	24/7539	5.38E-06	3.20E-05

GO:1902107	positive regulation of leukocyte differentiation	87/7539	5.39E-06	3.20E-05
GO:0055021	regulation of cardiac muscle tissue growth	45/7539	5.39E-06	3.20E-05
GO:0002011	morphogenesis of an epithelial sheet	36/7539	5.44E-06	3.23E-05
GO:0110111	negative regulation of animal organ morphogenesis	26/7539	5.45E-06	3.23E-05
GO:1905209	positive regulation of cardiocyte differentiation	25/7539	5.46E-06	3.24E-05
GO:1903076	regulation of protein localization to plasma membrane	60/7539	5.53E-06	3.27E-05
GO:0007586	digestion	65/7539	5.57E-06	3.29E-05
GO:0048145	regulation of fibroblast proliferation	57/7539	5.77E-06	3.41E-05
GO:0043506	regulation of JUN kinase activity	51/7539	5.92E-06	3.50E-05
GO:0002067	glandular epithelial cell differentiation	35/7539	5.99E-06	3.53E-05
GO:0055078	sodium ion homeostasis	35/7539	5.99E-06	3.53E-05
GO:0050878	regulation of body fluid levels	154/7539	5.99E-06	3.53E-05
GO:0055067	monovalent inorganic cation homeostasis	78/7539	6.03E-06	3.55E-05
GO:0015800	acidic amino acid transport	40/7539	6.05E-06	3.56E-05
GO:0001756	somitogenesis	44/7539	6.10E-06	3.58E-05
GO:0043507	positive regulation of JUN kinase activity	44/7539	6.10E-06	3.58E-05
GO:0043931	ossification involved in bone maturation	17/7539	6.34E-06	3.72E-05
GO:1903844	regulation of cellular response to transforming growth factor beta stimulus	59/7539	6.35E-06	3.72E-05

GO:0002088	lens development in camera-type eye	50/7539	6.77E-06	3.96E-05
GO:0007224	smoothened signaling pathway	77/7539	6.95E-06	4.06E-05
GO:0060271	cilium assembly	137/7539	7.23E-06	4.22E-05
GO:0022600	digestive system process	58/7539	7.30E-06	4.26E-05
GO:0007622	rhythmic behavior	38/7539	7.56E-06	4.40E-05
GO:0030858	positive regulation of epithelial cell differentiation	38/7539	7.56E-06	4.40E-05
GO:2000243	positive regulation of reproductive process	52/7539	7.77E-06	4.52E-05
GO:0035088	establishment or maintenance of apical/basal cell polarity	32/7539	7.79E-06	4.52E-05
GO:0051489	regulation of filopodium assembly	32/7539	7.79E-06	4.52E-05
GO:0061245	establishment or maintenance of bipolar cell polarity	32/7539	7.79E-06	4.52E-05
GO:0048681	negative regulation of axon regeneration	14/7539	7.88E-06	4.56E-05
GO:0055057	neuroblast division	14/7539	7.88E-06	4.56E-05
GO:0060979	vasculogenesis involved in coronary vascular morphogenesis	14/7539	7.88E-06	4.56E-05
GO:0010996	response to auditory stimulus	18/7539	7.96E-06	4.59E-05
GO:0072202	cell differentiation involved in metanephros development	18/7539	7.96E-06	4.59E-05
GO:1901626	regulation of postsynaptic membrane organization	18/7539	7.96E-06	4.59E-05
GO:0003229	ventricular cardiac muscle tissue development	37/7539	8.43E-06	4.85E-05
GO:0048512	circadian behavior	37/7539	8.43E-06	4.85E-05

GO:1905207	regulation of cardiocyte differentiation	37/7539	8.43E-06	4.85E-05
GO:0008088	axo-dendritic transport	45/7539	8.48E-06	4.87E-05
GO:0001885	endothelial cell development	41/7539	8.80E-06	5.05E-05
GO:0007492	endoderm development	41/7539	8.80E-06	5.05E-05
GO:0060420	regulation of heart growth	48/7539	8.83E-06	5.06E-05
GO:0010718	positive regulation of epithelial to mesenchymal transition	30/7539	9.07E-06	5.19E-05
GO:0070663	regulation of leukocyte proliferation	114/7539	9.17E-06	5.25E-05
GO:0036342	post-anal tail morphogenesis	19/7539	9.34E-06	5.33E-05
GO:0071880	adenylate cyclase-activating adrenergic receptor signaling pathway	19/7539	9.34E-06	5.33E-05
GO:1904738	vascular associated smooth muscle cell migration	19/7539	9.34E-06	5.33E-05
GO:1904752	regulation of vascular associated smooth muscle cell migration	19/7539	9.34E-06	5.33E-05
GO:0098900	regulation of action potential	36/7539	9.38E-06	5.34E-05
GO:0031128	developmental induction	29/7539	9.70E-06	5.52E-05
GO:0060421	positive regulation of heart growth	29/7539	9.70E-06	5.52E-05
GO:0048146	positive regulation of fibroblast proliferation	40/7539	9.92E-06	5.62E-05
GO:0071867	response to monoamine	40/7539	9.92E-06	5.62E-05
GO:0071869	response to catecholamine	40/7539	9.92E-06	5.62E-05
GO:0051259	protein complex oligomerization	103/7539	1.00E-05	5.69E-05

GO:0006109	regulation of carbohydrate metabolic process	92/7539	1.03E-05	5.80E-05
GO:0008286	insulin receptor signaling pathway	63/7539	1.04E-05	5.87E-05
GO:0010921	regulation of phosphatase activity	63/7539	1.04E-05	5.87E-05
GO:0010761	fibroblast migration	35/7539	1.04E-05	5.87E-05
GO:0014821	phasic smooth muscle contraction	20/7539	1.04E-05	5.87E-05
GO:0030201	heparan sulfate proteoglycan metabolic process	20/7539	1.04E-05	5.87E-05
GO:1903010	regulation of bone development	20/7539	1.04E-05	5.87E-05
GO:0097530	granulocyte migration	76/7539	1.08E-05	6.06E-05
GO:0021695	cerebellar cortex development	39/7539	1.12E-05	6.28E-05
GO:0030318	melanocyte differentiation	21/7539	1.12E-05	6.28E-05
GO:0045822	negative regulation of heart contraction	21/7539	1.12E-05	6.28E-05
GO:0030513	positive regulation of BMP signaling pathway	26/7539	1.13E-05	6.32E-05
GO:0031279	regulation of cyclase activity	26/7539	1.13E-05	6.32E-05
GO:0060251	regulation of glial cell proliferation	26/7539	1.13E-05	6.32E-05
GO:0090049	regulation of cell migration involved in sprouting angiogenesis	26/7539	1.13E-05	6.32E-05
GO:1901385	regulation of voltage-gated calcium channel activity	26/7539	1.13E-05	6.32E-05
GO:0044070	regulation of anion transport	67/7539	1.14E-05	6.34E-05
GO:0010660	regulation of muscle cell apoptotic process	52/7539	1.15E-05	6.42E-05

GO:0098751	bone cell development	25/7539	1.17E-05	6.47E-05
GO:0022011	myelination in peripheral nervous system	22/7539	1.17E-05	6.47E-05
GO:0032292	peripheral nervous system axon ensheathment	22/7539	1.17E-05	6.47E-05
GO:0042481	regulation of odontogenesis	22/7539	1.17E-05	6.47E-05
GO:0045932	negative regulation of muscle contraction	22/7539	1.17E-05	6.47E-05
GO:0051497	negative regulation of stress fiber assembly	22/7539	1.17E-05	6.47E-05
GO:1905314	semi-lunar valve development	22/7539	1.17E-05	6.47E-05
GO:0021520	spinal cord motor neuron cell fate specification	Dec-39	1.18E-05	6.51E-05
GO:0071679	commissural neuron axon guidance	Dec-39	1.18E-05	6.51E-05
GO:1902285	semaphorin-plexin signaling pathway involved in neuron projection guidance	Dec-39	1.18E-05	6.51E-05
GO:0019216	regulation of lipid metabolic process	156/7539	1.18E-05	6.52E-05
GO:0010591	regulation of lamellipodium assembly	24/7539	1.19E-05	6.54E-05
GO:0014047	glutamate secretion	24/7539	1.19E-05	6.54E-05
GO:0060487	lung epithelial cell differentiation	23/7539	1.19E-05	6.54E-05
GO:0060740	prostate gland epithelium morphogenesis	23/7539	1.19E-05	6.54E-05
GO:0060914	heart formation	23/7539	1.19E-05	6.54E-05
GO:0010002	cardioblast differentiation	15/7539	1.20E-05	6.60E-05
GO:0033604	negative regulation of catecholamine secretion	15/7539	1.20E-05	6.60E-05

GO:0060572	morphogenesis of an epithelial bud	15/7539	1.20E-05	6.60E-05
GO:0021766	hippocampus development	42/7539	1.24E-05	6.80E-05
GO:0051260	protein homooligomerization	83/7539	1.25E-05	6.82E-05
GO:1901216	positive regulation of neuron death	64/7539	1.26E-05	6.88E-05
GO:0007019	microtubule depolymerization	33/7539	1.27E-05	6.94E-05
GO:1900024	regulation of substrate adhesion-dependent cell spreading	33/7539	1.27E-05	6.94E-05
GO:0070507	regulation of microtubule cytoskeleton organization	94/7539	1.29E-05	7.05E-05
GO:0010927	cellular component assembly involved in morphogenesis	59/7539	1.30E-05	7.08E-05
GO:0045682	regulation of epidermis development	45/7539	1.31E-05	7.15E-05
GO:0046683	response to organophosphorus	48/7539	1.34E-05	7.27E-05
GO:0001974	blood vessel remodeling	32/7539	1.40E-05	7.60E-05
GO:0071241	cellular response to inorganic substance	104/7539	1.40E-05	7.62E-05
GO:0010633	negative regulation of epithelial cell migration	41/7539	1.41E-05	7.64E-05
GO:0046661	male sex differentiation	82/7539	1.43E-05	7.77E-05
GO:0030308	negative regulation of cell growth	93/7539	1.48E-05	8.03E-05
GO:0044782	cilium organization	148/7539	1.52E-05	8.20E-05
GO:0030166	proteoglycan biosynthetic process	31/7539	1.53E-05	8.29E-05
GO:0002695	negative regulation of leukocyte activation	89/7539	1.55E-05	8.40E-05

GO:0030512	negative regulation of transforming growth factor beta receptor signaling pathway	40/7539	1.59E-05	8.60E-05
GO:0002683	negative regulation of immune system process	192/7539	1.59E-05	8.60E-05
GO:0010763	positive regulation of fibroblast migration	16/7539	1.60E-05	8.62E-05
GO:0072189	ureter development	16/7539	1.60E-05	8.62E-05
GO:0046634	regulation of alpha-beta T cell activation	55/7539	1.60E-05	8.62E-05
GO:0046660	female sex differentiation	73/7539	1.65E-05	8.86E-05
GO:0009266	response to temperature stimulus	77/7539	1.66E-05	8.93E-05
GO:0031111	negative regulation of microtubule polymerization or depolymerization	30/7539	1.67E-05	8.95E-05
GO:0050850	positive regulation of calcium-mediated signaling	30/7539	1.67E-05	8.95E-05
GO:1990573	potassium ion import across plasma membrane	30/7539	1.67E-05	8.95E-05
GO:0060395	SMAD protein signal transduction	52/7539	1.69E-05	9.02E-05
GO:0140029	exocytic process	52/7539	1.69E-05	9.02E-05
GO:0031110	regulation of microtubule polymerization or depolymerization	49/7539	1.74E-05	9.30E-05
GO:0061515	myeloid cell development	46/7539	1.75E-05	9.35E-05
GO:0007588	excretion	35/7539	1.77E-05	9.41E-05
GO:0060443	mammary gland morphogenesis	35/7539	1.77E-05	9.41E-05
GO:0032413	negative regulation of ion transmembrane transporter activity	39/7539	1.80E-05	9.58E-05
GO:0046579	positive regulation of Ras protein signal transduction	39/7539	1.80E-05	9.58E-05

GO:0071868	cellular response to monoamine stimulus	39/7539	1.80E-05	9.58E-05
GO:0071870	cellular response to catecholamine stimulus	39/7539	1.80E-05	9.58E-05
GO:0042176	regulation of protein catabolic process	167/7539	1.81E-05	9.61E-05
GO:0003254	regulation of membrane depolarization	29/7539	1.81E-05	9.61E-05
GO:0031952	regulation of protein autophosphorylation	29/7539	1.81E-05	9.61E-05
GO:0060323	head morphogenesis	29/7539	1.81E-05	9.61E-05
GO:0070661	leukocyte proliferation	148/7539	1.82E-05	9.62E-05
GO:2001234	negative regulation of apoptotic signaling pathway	105/7539	1.94E-05	0.000102533
GO:1902547	regulation of cellular response to vascular endothelial growth factor stimulus	17/7539	1.95E-05	0.000102754
GO:0086002	cardiac muscle cell action potential involved in contraction	28/7539	1.96E-05	0.000103286
GO:1903672	positive regulation of sprouting angiogenesis	28/7539	1.96E-05	0.000103286
GO:0051591	response to cAMP	34/7539	1.97E-05	0.000103829
GO:0045639	positive regulation of myeloid cell differentiation	56/7539	1.99E-05	0.00010454
GO:0032370	positive regulation of lipid transport	45/7539	2.00E-05	0.000105332
GO:0010762	regulation of fibroblast migration	27/7539	2.10E-05	0.000110246
GO:0021955	central nervous system neuron axonogenesis	27/7539	2.10E-05	0.000110246
GO:0055023	positive regulation of cardiac muscle tissue growth	27/7539	2.10E-05	0.000110246
GO:0014848	urinary tract smooth muscle contraction	13/7539	2.15E-05	0.000112177

GO:0060442	branching involved in prostate gland morphogenesis	13/7539	2.15E-05	0.000112177
GO:0061029	eyelid development in camera-type eye	13/7539	2.15E-05	0.000112177
GO:1903587	regulation of blood vessel endothelial cell proliferation involved in sprouting angiogenesis	13/7539	2.15E-05	0.000112177
GO:2000095	regulation of Wnt signaling pathway, planar cell polarity pathway	13/7539	2.15E-05	0.000112177
GO:0046546	development of primary male sexual characteristics	69/7539	2.16E-05	0.000112901
GO:0032892	positive regulation of organic acid transport	33/7539	2.19E-05	0.000114283
GO:0051496	positive regulation of stress fiber assembly	33/7539	2.19E-05	0.000114283
GO:0061001	regulation of dendritic spine morphogenesis	33/7539	2.19E-05	0.000114283
GO:0010950	positive regulation of endopeptidase activity	77/7539	2.20E-05	0.000114381
GO:0015908	fatty acid transport	50/7539	2.23E-05	0.000115829
GO:0048741	skeletal muscle fiber development	26/7539	2.24E-05	0.000116139
GO:0085029	extracellular matrix assembly	26/7539	2.24E-05	0.000116139
GO:1901880	negative regulation of protein depolymerization	44/7539	2.29E-05	0.000118771
GO:0043388	positive regulation of DNA binding	37/7539	2.30E-05	0.000119135
GO:0099637	neurotransmitter receptor transport	37/7539	2.30E-05	0.000119135
GO:1902305	regulation of sodium ion transmembrane transport	37/7539	2.30E-05	0.000119135
GO:0001953	negative regulation of cell-matrix adhesion	25/7539	2.36E-05	0.000121905
GO:0048265	response to pain	25/7539	2.36E-05	0.000121905

GO:2000463	positive regulation of excitatory postsynaptic potential	25/7539	2.36E-05	0.000121905
GO:0021795	cerebral cortex cell migration	32/7539	2.44E-05	0.000125377
GO:0046676	negative regulation of insulin secretion	32/7539	2.44E-05	0.000125377
GO:0048662	negative regulation of smooth muscle cell proliferation	32/7539	2.44E-05	0.000125377
GO:0055010	ventricular cardiac muscle tissue morphogenesis	32/7539	2.44E-05	0.000125377
GO:0007200	phospholipase C-activating G protein-coupled receptor signaling pathway	52/7539	2.44E-05	0.000125513
GO:0009581	detection of external stimulus	66/7539	2.45E-05	0.000125788
GO:0003401	axis elongation	24/7539	2.47E-05	0.0001263
GO:0035115	embryonic forelimb morphogenesis	24/7539	2.47E-05	0.0001263
GO:0042220	response to cocaine	24/7539	2.47E-05	0.0001263
GO:0048679	regulation of axon regeneration	24/7539	2.47E-05	0.0001263
GO:0009895	negative regulation of catabolic process	135/7539	2.47E-05	0.0001263
GO:0071875	adrenergic receptor signaling pathway	20/7539	2.54E-05	0.000129525
GO:2000178	negative regulation of neural precursor cell proliferation	20/7539	2.54E-05	0.000129525
GO:0014044	Schwann cell development	23/7539	2.55E-05	0.000129726
GO:0048710	regulation of astrocyte differentiation	23/7539	2.55E-05	0.000129726
GO:0060479	lung cell differentiation	23/7539	2.55E-05	0.000129726
GO:0060512	prostate gland morphogenesis	23/7539	2.55E-05	0.000129726

GO:0006929	substrate-dependent cell migration	21/7539	2.59E-05	0.000131374
GO:0035767	endothelial cell chemotaxis	21/7539	2.59E-05	0.000131374
GO:0048521	negative regulation of behavior	21/7539	2.59E-05	0.000131374
GO:0060317	cardiac epithelial to mesenchymal transition	21/7539	2.59E-05	0.000131374
GO:0021697	cerebellar cortex formation	22/7539	2.59E-05	0.000131374
GO:0090075	relaxation of muscle	22/7539	2.59E-05	0.000131374
GO:0061337	cardiac conduction	36/7539	2.60E-05	0.000131437
GO:0070373	negative regulation of ERK1 and ERK2 cascade	43/7539	2.62E-05	0.000132402
GO:1902106	negative regulation of leukocyte differentiation	54/7539	2.63E-05	0.000133182
GO:0001819	positive regulation of cytokine production	186/7539	2.68E-05	0.000135269
GO:0046189	phenol-containing compound biosynthetic process	31/7539	2.70E-05	0.000136182
GO:0010952	positive regulation of peptidase activity	81/7539	2.81E-05	0.000141757
GO:1900076	regulation of cellular response to insulin stimulus	48/7539	2.94E-05	0.000148241
GO:0051235	maintenance of location	138/7539	2.98E-05	0.000149837
GO:0061028	establishment of endothelial barrier	30/7539	2.98E-05	0.000149837
GO:0003198	epithelial to mesenchymal transition involved in endocardial cushion formation	14/7539	3.13E-05	0.000157164
GO:0051648	vesicle localization	82/7539	3.17E-05	0.000159258
GO:0042596	fear response	34/7539	3.29E-05	0.000164951

GO:0008584	male gonad development	68/7539	3.35E-05	0.000167923
GO:0014832	urinary bladder smooth muscle contraction	Nov-39	3.38E-05	0.000168493
GO:0021561	facial nerve development	Nov-39	3.38E-05	0.000168493
GO:0021610	facial nerve morphogenesis	Nov-39	3.38E-05	0.000168493
GO:0048241	epinephrine transport	Nov-39	3.38E-05	0.000168493
GO:1902287	semaphorin-plexin signaling pathway involved in axon guidance	Nov-39	3.38E-05	0.000168493
GO:1903431	positive regulation of cell maturation	Nov-39	3.38E-05	0.000168493
GO:0007009	plasma membrane organization	57/7539	3.41E-05	0.000169851
GO:0050670	regulation of lymphocyte proliferation	104/7539	3.45E-05	0.000171898
GO:0050870	positive regulation of T cell activation	92/7539	3.52E-05	0.000175219
GO:0001569	branching involved in blood vessel morphogenesis	28/7539	3.59E-05	0.000178297
GO:0045494	photoreceptor cell maintenance	28/7539	3.59E-05	0.000178297
GO:0030199	collagen fibril organization	33/7539	3.69E-05	0.000182805
GO:0071715	icosanoid transport	33/7539	3.69E-05	0.000182805
GO:1901571	fatty acid derivative transport	33/7539	3.69E-05	0.000182805
GO:1902893	regulation of pri-miRNA transcription by RNA polymerase II	33/7539	3.69E-05	0.000182805
GO:0009798	axis specification	49/7539	3.72E-05	0.000184325
GO:0008277	regulation of G protein-coupled receptor signaling pathway	63/7539	3.76E-05	0.000185649

GO:0032944	regulation of mononuclear cell proliferation	105/7539	3.76E-05	0.000185649
GO:0010675	regulation of cellular carbohydrate metabolic process	77/7539	3.78E-05	0.000186524
GO:0044262	cellular carbohydrate metabolic process	127/7539	3.79E-05	0.000186922
GO:0009582	detection of abiotic stimulus	65/7539	3.82E-05	0.000188369
GO:0051346	negative regulation of hydrolase activity	169/7539	3.83E-05	0.000188743
GO:2001056	positive regulation of cysteine-type endopeptidase activity	69/7539	3.88E-05	0.000191015
GO:0007193	adenylate cyclase-inhibiting G protein-coupled receptor signaling pathway	40/7539	3.90E-05	0.000191797
GO:1903845	negative regulation of cellular response to transforming growth factor beta stimulus	40/7539	3.90E-05	0.000191797
GO:0045197	establishment or maintenance of epithelial cell apical/basal polarity	27/7539	3.91E-05	0.000192021
GO:0120034	positive regulation of plasma membrane bounded cell projection assembly	56/7539	3.93E-05	0.000192848
GO:0001975	response to amphetamine	15/7539	4.00E-05	0.000195018
GO:0003180	aortic valve morphogenesis	15/7539	4.00E-05	0.000195018
GO:0003215	cardiac right ventricle morphogenesis	15/7539	4.00E-05	0.000195018
GO:0035930	corticosteroid hormone secretion	15/7539	4.00E-05	0.000195018
GO:0043116	negative regulation of vascular permeability	15/7539	4.00E-05	0.000195018
GO:0060231	mesenchymal to epithelial transition	15/7539	4.00E-05	0.000195018
GO:0072578	neurotransmitter-gated ion channel clustering	15/7539	4.00E-05	0.000195018
GO:1904754	positive regulation of vascular associated smooth muscle cell migration	15/7539	4.00E-05	0.000195018

GO:0018958	phenol-containing compound metabolic process	58/7539	4.09E-05	0.000199605
GO:0014009	glial cell proliferation	32/7539	4.13E-05	0.000201134
GO:0046717	acid secretion	32/7539	4.13E-05	0.000201134
GO:0010830	regulation of myotube differentiation	36/7539	4.18E-05	0.000203294
GO:0010470	regulation of gastrulation	26/7539	4.24E-05	0.000205513
GO:0014037	Schwann cell differentiation	26/7539	4.24E-05	0.000205513
GO:0098801	regulation of renal system process	26/7539	4.24E-05	0.000205513
GO:0009416	response to light stimulus	129/7539	4.30E-05	0.000208726
GO:0008064	regulation of actin polymerization or depolymerization	87/7539	4.31E-05	0.000208958
GO:0042752	regulation of circadian rhythm	64/7539	4.40E-05	0.000213206
GO:0043954	cellular component maintenance	39/7539	4.45E-05	0.000215194
GO:0048332	mesoderm morphogenesis	39/7539	4.45E-05	0.000215194
GO:0043255	regulation of carbohydrate biosynthetic process	55/7539	4.53E-05	0.000218813
GO:0003407	neural retina development	42/7539	4.53E-05	0.000218813
GO:0031334	positive regulation of protein-containing complex assembly	105/7539	4.65E-05	0.000224205
GO:0001502	cartilage condensation	16/7539	4.70E-05	0.000225749
GO:0003177	pulmonary valve development	16/7539	4.70E-05	0.000225749
GO:0014075	response to amine	16/7539	4.70E-05	0.000225749

GO:0031290	retinal ganglion cell axon guidance	16/7539	4.70E-05	0.000225749
GO:0031641	regulation of myelination	35/7539	4.73E-05	0.000227463
GO:0030832	regulation of actin filament length	88/7539	4.78E-05	0.00022955
GO:2000273	positive regulation of signaling receptor activity	24/7539	4.87E-05	0.00023367
GO:0018107	peptidyl-threonine phosphorylation	59/7539	4.88E-05	0.000233818
GO:0006644	phospholipid metabolic process	157/7539	4.92E-05	0.000235688
GO:0042490	mechanoreceptor differentiation	47/7539	4.93E-05	0.000236048
GO:0071456	cellular response to hypoxia	52/7539	4.96E-05	0.000237127
GO:0051250	negative regulation of lymphocyte activation	75/7539	4.99E-05	0.0002387
GO:0044770	cell cycle phase transition	172/7539	5.10E-05	0.000243615
GO:0032309	icosanoid secretion	30/7539	5.15E-05	0.000245709
GO:0060441	epithelial tube branching involved in lung morphogenesis	23/7539	5.16E-05	0.000245903
GO:0032331	negative regulation of chondrocyte differentiation	17/7539	5.20E-05	0.000247572
GO:2000831	regulation of steroid hormone secretion	17/7539	5.20E-05	0.000247572
GO:0030316	osteoclast differentiation	56/7539	5.45E-05	0.000259266
GO:0060004	reflex	18/7539	5.52E-05	0.000262124
GO:0086019	cell-cell signaling involved in cardiac conduction	18/7539	5.52E-05	0.000262124
GO:0035640	exploration behavior	21/7539	5.58E-05	0.000265088

GO:0009755	hormone-mediated signaling pathway	76/7539	5.64E-05	0.000267648
GO:0001956	positive regulation of neurotransmitter secretion	19/7539	5.67E-05	0.000267936
GO:0003171	atrioventricular valve development	19/7539	5.67E-05	0.000267936
GO:0010742	macrophage derived foam cell differentiation	19/7539	5.67E-05	0.000267936
GO:0014829	vascular associated smooth muscle contraction	19/7539	5.67E-05	0.000267936
GO:0050926	regulation of positive chemotaxis	19/7539	5.67E-05	0.000267936
GO:0021952	central nervous system projection neuron axonogenesis	20/7539	5.68E-05	0.000268342
GO:0001707	mesoderm formation	37/7539	5.79E-05	0.0002729
GO:0042982	amyloid precursor protein metabolic process	37/7539	5.79E-05	0.0002729
GO:0008643	carbohydrate transport	72/7539	5.83E-05	0.000274568
GO:0043536	positive regulation of blood vessel endothelial cell migration	33/7539	6.05E-05	0.000284928
GO:0090630	activation of GTPase activity	48/7539	6.17E-05	0.000290077
GO:0035303	regulation of dephosphorylation	79/7539	6.19E-05	0.000291154
GO:0030041	actin filament polymerization	86/7539	6.28E-05	0.000294777
GO:0010259	multicellular organism aging	28/7539	6.34E-05	0.000297727
GO:0033135	regulation of peptidyl-serine phosphorylation	77/7539	6.35E-05	0.000297727
GO:0006835	dicarboxylic acid transport	45/7539	6.53E-05	0.00030599
GO:0043367	CD4-positive, alpha-beta T cell differentiation	45/7539	6.53E-05	0.00030599

GO:0045668	negative regulation of osteoblast differentiation	36/7539	6.59E-05	0.00030835
GO:1903510	mucopolysaccharide metabolic process	36/7539	6.59E-05	0.00030835
GO:0060042	retina morphogenesis in camera-type eye	42/7539	6.77E-05	0.000316312
GO:0061008	hepaticobiliary system development	63/7539	6.82E-05	0.000318361
GO:0007605	sensory perception of sound	119/7539	7.03E-05	0.000327841
GO:0043242	negative regulation of protein-containing complex disassembly	47/7539	7.11E-05	0.00033134
GO:0015695	organic cation transport	35/7539	7.51E-05	0.000349718
GO:1902115	regulation of organelle assembly	93/7539	7.67E-05	0.000356987
GO:0030850	prostate gland development	31/7539	7.69E-05	0.000357617
GO:0044058	regulation of digestive system process	31/7539	7.69E-05	0.000357617
GO:0051339	regulation of lyase activity	26/7539	7.70E-05	0.000357862
GO:0031333	negative regulation of protein-containing complex assembly	70/7539	7.72E-05	0.000358275
GO:0031109	microtubule polymerization or depolymerization	60/7539	7.79E-05	0.000361427
GO:0040034	regulation of development, heterochronic	13/7539	8.03E-05	0.000371466
GO:0043584	nose development	13/7539	8.03E-05	0.000371466
GO:0090128	regulation of synapse maturation	13/7539	8.03E-05	0.000371466
GO:0090494	dopamine uptake	13/7539	8.03E-05	0.000371466
GO:0032103	positive regulation of response to external stimulus	172/7539	8.04E-05	0.000371508

GO:0050953	sensory perception of light stimulus	77/7539	8.17E-05	0.0003771
GO:0006112	energy reserve metabolic process	46/7539	8.19E-05	0.00037797
GO:0051302	regulation of cell division	75/7539	8.38E-05	0.00038636
GO:0014742	positive regulation of muscle hypertrophy	25/7539	8.43E-05	0.000387329
GO:0015804	neutral amino acid transport	25/7539	8.43E-05	0.000387329
GO:0048520	positive regulation of behavior	25/7539	8.43E-05	0.000387329
GO:0060259	regulation of feeding behavior	25/7539	8.43E-05	0.000387329
GO:0060325	face morphogenesis	25/7539	8.43E-05	0.000387329
GO:0010524	positive regulation of calcium ion transport into cytosol	34/7539	8.54E-05	0.000391715
GO:0060711	labyrinthine layer development	34/7539	8.54E-05	0.000391715
GO:0071470	cellular response to osmotic stress	30/7539	8.65E-05	0.000396576
GO:0043280	positive regulation of cysteine-type endopeptidase activity involved in apoptotic process	61/7539	9.07E-05	0.000415327
GO:0043267	negative regulation of potassium ion transport	24/7539	9.17E-05	0.000419629
GO:1903170	negative regulation of calcium ion transmembrane transport	24/7539	9.17E-05	0.000419629
GO:0021559	trigeminal nerve development	Oct-39	9.62E-05	0.000438725
GO:0051458	corticotropin secretion	Oct-39	9.62E-05	0.000438725
GO:0071415	cellular response to purine-containing compound	Oct-39	9.62E-05	0.000438725
GO:0072102	glomerulus morphogenesis	Oct-39	9.62E-05	0.000438725

GO:0010517	regulation of phospholipase activity	33/7539	9.70E-05	0.000441813
GO:0061614	pri-miRNA transcription by RNA polymerase II	33/7539	9.70E-05	0.000441813
GO:0090659	walking behavior	29/7539	9.71E-05	0.000441813
GO:0003128	heart field specification	14/7539	9.86E-05	0.000447039
GO:0031281	positive regulation of cyclase activity	14/7539	9.86E-05	0.000447039
GO:0051580	regulation of neurotransmitter uptake	14/7539	9.86E-05	0.000447039
GO:0086011	membrane repolarization during action potential	14/7539	9.86E-05	0.000447039
GO:2000846	regulation of corticosteroid hormone secretion	14/7539	9.86E-05	0.000447039
GO:0010453	regulation of cell fate commitment	23/7539	9.92E-05	0.000448385
GO:0035886	vascular associated smooth muscle cell differentiation	23/7539	9.92E-05	0.000448385
GO:0061384	heart trabecula morphogenesis	23/7539	9.92E-05	0.000448385
GO:0071604	transforming growth factor beta production	23/7539	9.92E-05	0.000448385
GO:0030833	regulation of actin filament polymerization	79/7539	0.000101206	0.000457021
GO:0072593	reactive oxygen species metabolic process	125/7539	0.000101206	0.000457021
GO:0021782	glial cell development	58/7539	0.000103702	0.000467958
GO:2001235	positive regulation of apoptotic signaling pathway	84/7539	0.000103904	0.000468534
GO:0044772	mitotic cell cycle phase transition	156/7539	0.000104288	0.000469927
GO:0019934	cGMP-mediated signaling	22/7539	0.000106334	0.000477444

GO:0035329	hippo signaling	22/7539	0.000106334	0.000477444
GO:0043950	positive regulation of cAMP-mediated signaling	22/7539	0.000106334	0.000477444
GO:0071634	regulation of transforming growth factor beta production	22/7539	0.000106334	0.000477444
GO:0090162	establishment of epithelial cell polarity	22/7539	0.000106334	0.000477444
GO:0048545	response to steroid hormone	97/7539	0.000106942	0.000479832
GO:0007628	adult walking behavior	28/7539	0.000108674	0.000486911
GO:2001239	regulation of extrinsic apoptotic signaling pathway in absence of ligand	28/7539	0.000108674	0.000486911
GO:0001895	retina homeostasis	32/7539	0.000110154	0.000492845
GO:0043647	inositol phosphate metabolic process	32/7539	0.000110154	0.000492845
GO:0001696	gastric acid secretion	15/7539	0.00011186	0.000498356
GO:0048557	embryonic digestive tract morphogenesis	15/7539	0.00011186	0.000498356
GO:0072079	nephron tubule formation	15/7539	0.00011186	0.000498356
GO:0072087	renal vesicle development	15/7539	0.00011186	0.000498356
GO:0072243	metanephric nephron epithelium development	15/7539	0.00011186	0.000498356
GO:1900746	regulation of vascular endothelial growth factor signaling pathway	15/7539	0.00011186	0.000498356
GO:0051651	maintenance of location in cell	95/7539	0.000112538	0.000501023
GO:0032958	inositol phosphate biosynthetic process	21/7539	0.000112965	0.000502218
GO:0051150	regulation of smooth muscle cell differentiation	21/7539	0.000112965	0.000502218

GO:0002262	myeloid cell homeostasis	85/7539	0.000114131	0.000507044
GO:0031330	negative regulation of cellular catabolic process	109/7539	0.000114479	0.000508231
GO:0006809	nitric oxide biosynthetic process	41/7539	0.0001146 6	0.000508678
GO:0098656	anion transmembrane transport	98/7539	0.000115585	0.000512421
GO:0046651	lymphocyte proliferation	134/7539	0.000116273	0.000515108
GO:0010922	positive regulation of phosphatase activity	20/7539	0.000118645	0.000524513
GO:0035162	embryonic hemopoiesis	20/7539	0.000118645	0.000524513
GO:0071711	basement membrane organization	20/7539	0.000118645	0.000524513
GO:0010970	transport along microtubule	76/7539	0.000119899	0.000529689
GO:0015012	heparan sulfate proteoglycan biosynthetic process	16/7539	0.0001202 9	0.000530299
GO:0032148	activation of protein kinase B activity	16/7539	0.0001202 9	0.000530299
GO:0048532	anatomical structure arrangement	16/7539	0.0001202 9	0.000530299
GO:0002688	regulation of leukocyte chemotaxis	59/7539	0.000120586	0.000531235
GO:0001889	liver development	61/7539	0.000120772	0.000531682
GO:0032943	mononuclear cell proliferation	135/7539	0.000122302	0.000538043
GO:0006837	serotonin transport	19/7539	0.000122871	0.000539419
GO:0061217	regulation of mesonephros development	19/7539	0.000122871	0.000539419
GO:0090077	foam cell differentiation	19/7539	0.000122871	0.000539419

GO:0003181	atrioventricular valve morphogenesis	17/7539	0.000124431	0.000543254
GO:0044331	cell-cell adhesion mediated by cadherin	17/7539	0.000124431	0.000543254
GO:0072207	metanephric epithelium development	17/7539	0.000124431	0.000543254
GO:0052547	regulation of peptidase activity	172/7539	0.000124819	0.000543254
GO:0002209	behavioral defense response	31/7539	0.000124917	0.000543254
GO:0034113	heterotypic cell-cell adhesion	31/7539	0.000124917	0.000543254
GO:2000649	regulation of sodium ion transmembrane transporter activity	31/7539	0.000124917	0.000543254
GO:0030325	adrenal gland development	18/7539	0.000125035	0.000543254
GO:0034104	negative regulation of tissue remodeling	18/7539	0.000125035	0.000543254
GO:0035929	steroid hormone secretion	18/7539	0.000125035	0.000543254
GO:0050927	positive regulation of positive chemotaxis	18/7539	0.000125035	0.000543254
GO:0090280	positive regulation of calcium ion import	18/7539	0.000125035	0.000543254
GO:0120033	negative regulation of plasma membrane bounded cell projection assembly	18/7539	0.000125035	0.000543254
GO:0140058	neuron projection arborization	18/7539	0.000125035	0.000543254
GO:2000738	positive regulation of stem cell differentiation	18/7539	0.000125035	0.000543254
GO:0034101	erythrocyte homeostasis	70/7539	0.000130201	0.000565309
GO:0046626	regulation of insulin receptor signaling pathway	40/7539	0.000131963	0.000572563
GO:0046636	negative regulation of alpha-beta T cell activation	26/7539	0.00013508	0.000585689

GO:0009896	positive regulation of catabolic process	187/7539	0.000136545	0.000591631
GO:1903038	negative regulation of leukocyte cell-cell adhesion	64/7539	0.000137307	0.000594527
GO:0042491	inner ear auditory receptor cell differentiation	30/7539	0.000141479	0.000611749
GO:1904036	negative regulation of epithelial cell apoptotic process	30/7539	0.000141479	0.000611749
GO:0009991	response to extracellular stimulus	156/7539	0.000141718	0.0006122
GO:0062013	positive regulation of small molecule metabolic process	73/7539	0.000141874	0.0006122
GO:1903707	negative regulation of hemopoiesis	73/7539	0.000141874	0.0006122
GO:0002793	positive regulation of peptide secretion	101/7539	0.000144232	0.000621952
GO:0060603	mammary gland duct morphogenesis	25/7539	0.000149845	0.000644832
GO:1900744	regulation of p38MAPK cascade	25/7539	0.000149845	0.000644832
GO:1905332	positive regulation of morphogenesis of an epithelium	25/7539	0.000149845	0.000644832
GO:0007183	SMAD protein complex assembly	Nov-39	0.000154698	0.00066121
GO:0021604	cranial nerve structural organization	Nov-39	0.000154698	0.00066121
GO:0031223	auditory behavior	Nov-39	0.000154698	0.00066121
GO:0035630	bone mineralization involved in bone maturation	Nov-39	0.000154698	0.00066121
GO:0045161	neuronal ion channel clustering	Nov-39	0.000154698	0.00066121
GO:0045906	negative regulation of vasoconstriction	Nov-39	0.000154698	0.00066121
GO:0060601	lateral sprouting from an epithelium	Nov-39	0.000154698	0.00066121

GO:0070831	basement membrane assembly	Nov-39	0.000154698	0.00066121
GO:0072216	positive regulation of metanephros development	Nov-39	0.000154698	0.00066121
GO:2000833	positive regulation of steroid hormone secretion	Nov-39	0.000154698	0.00066121
GO:0006650	glycerophospholipid metabolic process	111/7539	0.000157316	0.000671946
GO:0052548	regulation of endopeptidase activity	144/7539	0.000158605	0.000676992
GO:0010613	positive regulation of cardiac muscle hypertrophy	24/7539	0.000165545	0.000706141
GO:0030522	intracellular receptor signaling pathway	92/7539	0.000166468	0.000709595
GO:0002066	columnar/cuboidal epithelial cell development	41/7539	0.000166706	0.000710131
GO:0034614	cellular response to reactive oxygen species	70/7539	0.000167642	0.000713638
GO:0001678	cellular glucose homeostasis	75/7539	0.0001749	0.000744031
GO:0030168	platelet activation	43/7539	0.000179433	0.000762802
GO:0003382	epithelial cell morphogenesis	28/7539	0.000180669	0.000766506
GO:0038066	p38MAPK cascade	28/7539	0.000180669	0.000766506
GO:1903115	regulation of actin filament-based movement	28/7539	0.000180669	0.000766506
GO:0002691	regulation of cellular extravasation	23/7539	0.00018202	0.000771719
GO:1903409	reactive oxygen species biosynthetic process	60/7539	0.000184084	0.00077995
GO:0030216	keratinocyte differentiation	56/7539	0.000185119	0.000783807
GO:0035176	social behavior	31/7539	0.00019806	0.000837478

GO:0045604	regulation of epidermal cell differentiation	31/7539	0.00019806	0.000837478
GO:0043297	apical junction assembly	47/7539	0.000198472	0.000838116
GO:0022411	cellular component disassembly	157/7539	0.000198595	0.000838116
GO:0010719	negative regulation of epithelial to mesenchymal transition	22/7539	0.000199008	0.000838116
GO:0086091	regulation of heart rate by cardiac conduction	22/7539	0.000199008	0.000838116
GO:1990089	response to nerve growth factor	22/7539	0.000199008	0.000838116
GO:1990090	cellular response to nerve growth factor stimulus	22/7539	0.000199008	0.000838116
GO:0007601	visual perception	74/7539	0.000200503	0.000843851
GO:0034394	protein localization to cell surface	37/7539	0.000201121	0.000845888
GO:0030218	erythrocyte differentiation	65/7539	0.000202452	0.000850916
GO:0032222	regulation of synaptic transmission, cholinergic	Dec-39	0.000203748	0.00085272
GO:0034698	response to gonadotropin	Dec-39	0.000203748	0.00085272
GO:0042428	serotonin metabolic process	Dec-39	0.000203748	0.00085272
GO:0086014	atrial cardiac muscle cell action potential	Dec-39	0.000203748	0.00085272
GO:0086026	atrial cardiac muscle cell to AV node cell signaling	Dec-39	0.000203748	0.00085272
GO:0086066	atrial cardiac muscle cell to AV node cell communication	Dec-39	0.000203748	0.00085272
GO:0051926	negative regulation of calcium ion transport	34/7539	0.000203961	0.00085272
GO:0098930	axonal transport	34/7539	0.000203961	0.00085272

GO:0050728	negative regulation of inflammatory response	72/7539	0.000207697	0.000867763
GO:0071496	cellular response to external stimulus	119/7539	0.000208544	0.000870728
GO:0043244	regulation of protein-containing complex disassembly	61/7539	0.000209782	0.000875316
GO:0050764	regulation of phagocytosis	55/7539	0.000213595	0.000890639
GO:0022410	circadian sleep/wake cycle process	21/7539	0.000216124	0.000899401
GO:0034260	negative regulation of GTPase activity	21/7539	0.000216124	0.000899401
GO:1902745	positive regulation of lamellipodium organization	21/7539	0.000216124	0.000899401
GO:0043433	negative regulation of DNA-binding transcription factor activity	75/7539	0.000221248	0.000920119
GO:0001662	behavioral fear response	30/7539	0.000225675	0.000935449
GO:0010596	negative regulation of endothelial cell migration	30/7539	0.000225675	0.000935449
GO:0043666	regulation of phosphoprotein phosphatase activity	30/7539	0.000225675	0.000935449
GO:0051955	regulation of amino acid transport	30/7539	0.000225675	0.000935449
GO:0097720	calcineurin-mediated signaling	30/7539	0.000225675	0.000935449
GO:0021522	spinal cord motor neuron differentiation	26/7539	0.000229001	0.000947367
GO:0033628	regulation of cell adhesion mediated by integrin	26/7539	0.000229001	0.000947367
GO:1902895	positive regulation of pri-miRNA transcription by RNA polymerase II	26/7539	0.000229001	0.000947367
GO:0071383	cellular response to steroid hormone stimulus	73/7539	0.0002298<br>4	0.000950217
GO:0001963	synaptic transmission, dopaminergic	20/7539	0.000232821	0.0009569

GO:0002026	regulation of the force of heart contraction	20/7539	0.000232821	0.0009569
GO:0042634	regulation of hair cycle	20/7539	0.000232821	0.0009569
GO:0044319	wound healing, spreading of cells	20/7539	0.000232821	0.0009569
GO:0048535	lymph node development	20/7539	0.000232821	0.0009569
GO:0055075	potassium ion homeostasis	20/7539	0.000232821	0.0009569
GO:0061036	positive regulation of cartilage development	20/7539	0.000232821	0.0009569
GO:0086010	membrane depolarization during action potential	20/7539	0.000232821	0.0009569
GO:0090505	epiboly involved in wound healing	20/7539	0.000232821	0.0009569
GO:0002690	positive regulation of leukocyte chemotaxis	48/7539	0.000236721	0.000972298
GO:0032984	protein-containing complex disassembly	94/7539	0.000236967	0.000972675
GO:0051781	positive regulation of cell division	41/7539	0.000239084	0.000974475
GO:0021854	hypothalamus development	13/7539	0.000239731	0.000974475
GO:0035313	wound healing, spreading of epidermal cells	13/7539	0.000239731	0.000974475
GO:0036035	osteoclast development	13/7539	0.000239731	0.000974475
GO:0051589	negative regulation of neurotransmitter transport	13/7539	0.000239731	0.000974475
GO:0051895	negative regulation of focal adhesion assembly	13/7539	0.000239731	0.000974475
GO:0055012	ventricular cardiac muscle cell differentiation	13/7539	0.000239731	0.000974475
GO:0072010	glomerular epithelium development	13/7539	0.000239731	0.000974475

GO:0090493	catecholamine uptake	13/7539	0.000239731	0.000974475
GO:0097152	mesenchymal cell apoptotic process	13/7539	0.000239731	0.000974475
GO:0098814	spontaneous synaptic transmission	13/7539	0.000239731	0.000974475
GO:0150118	negative regulation of cell-substrate junction organization	13/7539	0.000239731	0.000974475
GO:1901160	primary amino compound metabolic process	13/7539	0.000239731	0.000974475
GO:2000252	negative regulation of feeding behavior	13/7539	0.000239731	0.000974475
GO:2000369	regulation of clathrin-dependent endocytosis	13/7539	0.000239731	0.000974475
GO:0045732	positive regulation of protein catabolic process	97/7539	0.000239875	0.000974475
GO:0071621	granulocyte chemotaxis	60/7539	0.000241641	0.000981018
GO:0051100	negative regulation of binding	76/7539	0.000243312	0.000987166
GO:0003085	negative regulation of systemic arterial blood pressure	19/7539	0.000248347	0.00100501
GO:0045761	regulation of adenylate cyclase activity	19/7539	0.000248347	0.00100501
GO:0061311	cell surface receptor signaling pathway involved in heart development	19/7539	0.000248347	0.00100501
GO:0099622	cardiac muscle cell membrane repolarization	19/7539	0.000248347	0.00100501
GO:0046530	photoreceptor cell differentiation	43/7539	0.000253496	0.00102519
GO:0015698	inorganic anion transport	67/7539	0.000254116	0.00102704
GO:0048806	genitalia development	29/7539	0.0002569	0.001037628
GO:0003338	metanephros morphogenesis	18/7539	0.000261701	0.001052305

GO:0033688	regulation of osteoblast proliferation	18/7539	0.000261701	0.001052305
GO:0042044	fluid transport	18/7539	0.000261701	0.001052305
GO:0060045	positive regulation of cardiac muscle cell proliferation	18/7539	0.000261701	0.001052305
GO:0061437	renal system vasculature development	18/7539	0.000261701	0.001052305
GO:0061440	kidney vasculature development	18/7539	0.000261701	0.001052305
GO:1905809	negative regulation of synapse organization	18/7539	0.000261701	0.001052305
GO:0014061	regulation of norepinephrine secretion	14/7539	0.000262644	0.001053411
GO:0048243	norepinephrine secretion	14/7539	0.000262644	0.001053411
GO:0051349	positive regulation of lyase activity	14/7539	0.000262644	0.001053411
GO:1905276	regulation of epithelial tube formation	14/7539	0.000262644	0.001053411
GO:0000082	G1/S transition of mitotic cell cycle	82/7539	0.000264995	0.001062169
GO:2000514	regulation of CD4-positive, alpha-beta T cell activation	35/7539	0.000266271	0.001066605
GO:0001754	eye photoreceptor cell differentiation	32/7539	0.000268024	0.001070231
GO:0010656	negative regulation of muscle cell apoptotic process	32/7539	0.000268024	0.001070231
GO:0035315	hair cell differentiation	32/7539	0.000268024	0.001070231
GO:0048016	inositol phosphate-mediated signaling	32/7539	0.000268024	0.001070231
GO:0051703	intraspecies interaction between organisms	32/7539	0.000268024	0.001070231
GO:0014048	regulation of glutamate secretion	17/7539	0.000271586	0.001076155

GO:0032878	regulation of establishment or maintenance of cell polarity	17/7539	0.000271586	0.001076155
GO:0035024	negative regulation of Rho protein signal transduction	17/7539	0.000271586	0.001076155
GO:0048266	behavioral response to pain	17/7539	0.000271586	0.001076155
GO:0061213	positive regulation of mesonephros development	17/7539	0.000271586	0.001076155
GO:0098743	cell aggregation	17/7539	0.000271586	0.001076155
GO:0007270	neuron-neuron synaptic transmission	Sep-39	0.000271894	0.001076155
GO:0021612	facial nerve structural organization	Sep-39	0.000271894	0.001076155
GO:0050957	equilibrioception	Sep-39	0.000271894	0.001076155
GO:0051583	dopamine uptake involved in synaptic transmission	Sep-39	0.000271894	0.001076155
GO:0051934	catecholamine uptake involved in synaptic transmission	Sep-39	0.000271894	0.001076155
GO:0097119	postsynaptic density protein 95 clustering	Sep-39	0.000271894	0.001076155
GO:0098598	learned vocalization behavior or vocal learning	Sep-39	0.000271894	0.001076155
GO:1903011	negative regulation of bone development	Sep-39	0.000271894	0.001076155
GO:0033138	positive regulation of peptidyl-serine phosphorylation	61/7539	0.000273625	0.001082328
GO:0051481	negative regulation of cytosolic calcium ion concentration	15/7539	0.000274129	0.001083641
GO:0002313	mature B cell differentiation involved in immune response	16/7539	0.000276379	0.001087767
GO:0003176	aortic valve development	16/7539	0.000276379	0.001087767
GO:0034405	response to fluid shear stress	16/7539	0.000276379	0.001087767

GO:0036303	lymph vessel morphogenesis	16/7539	0.000276379	0.001087767
GO:0098703	calcium ion import across plasma membrane	16/7539	0.000276379	0.001087767
GO:0101023	vascular endothelial cell proliferation	16/7539	0.000276379	0.001087767
GO:1905562	regulation of vascular endothelial cell proliferation	16/7539	0.000276379	0.001087767
GO:0003073	regulation of systemic arterial blood pressure	59/7539	0.000278355	0.00109418
GO:1990266	neutrophil migration	59/7539	0.000278355	0.00109418
GO:0030431	sleep	24/7539	0.000287412	0.001126978
GO:0038179	neurotrophin signaling pathway	24/7539	0.000287412	0.001126978
GO:0086004	regulation of cardiac muscle cell contraction	24/7539	0.000287412	0.001126978
GO:0099590	neurotransmitter receptor internalization	24/7539	0.000287412	0.001126978
GO:0060113	inner ear receptor cell differentiation	42/7539	0.000292869	0.001147661
GO:0071322	cellular response to carbohydrate stimulus	71/7539	0.000301961	0.001182555
GO:0021799	cerebral cortex radially oriented cell migration	23/7539	0.000320465	0.001250367
GO:0031114	regulation of microtubule depolymerization	23/7539	0.000320465	0.001250367
GO:0035335	peptidyl-tyrosine dephosphorylation	23/7539	0.000320465	0.001250367
GO:0045684	positive regulation of epidermis development	23/7539	0.000320465	0.001250367
GO:0086065	cell communication involved in cardiac conduction	23/7539	0.000320465	0.001250367
GO:2000036	regulation of stem cell population maintenance	23/7539	0.000320465	0.001250367

GO:0050868	negative regulation of T cell activation	58/7539	0.000320664	0.001250372
GO:0051222	positive regulation of protein transport	143/7539	0.000327573	0.001276523
GO:0042098	T cell proliferation	93/7539	0.000327787	0.001276568
GO:0050974	detection of mechanical stimulus involved in sensory perception	27/7539	0.000331798	0.001291395
GO:0038034	signal transduction in absence of ligand	41/7539	0.000338414	0.001314711
GO:0097192	extrinsic apoptotic signaling pathway in absence of ligand	41/7539	0.000338414	0.001314711
GO:1901862	negative regulation of muscle tissue development	41/7539	0.000338414	0.001314711
GO:0046635	positive regulation of alpha-beta T cell activation	36/7539	0.000339808	0.001318506
GO:0048641	regulation of skeletal muscle tissue development	36/7539	0.000339808	0.001318506
GO:0036475	neuron death in response to oxidative stress	22/7539	0.000355924	0.001378496
GO:0042745	circadian sleep/wake cycle	22/7539	0.000355924	0.001378496
GO:2000191	regulation of fatty acid transport	22/7539	0.000355924	0.001378496
GO:0009314	response to radiation	162/7539	0.000361877	0.001400691
GO:1903825	organic acid transmembrane transport	59/7539	0.000362627	0.001402734
GO:0045786	negative regulation of cell cycle	180/7539	0.000364914	0.001410717
GO:1903793	positive regulation of anion transport	38/7539	0.00036808	0.00142191
GO:0009743	response to carbohydrate	89/7539	0.00036826	0.00142191
GO:0071300	cellular response to retinoic acid	26/7539	0.00037632	0.001452145

GO:0061180	mammary gland epithelium development	40/7539	0.000391109	0.001508292
GO:0032623	interleukin-2 production	35/7539	0.000391888	0.001509449
GO:2000736	regulation of stem cell differentiation	35/7539	0.000391888	0.001509449
GO:0021591	ventricular system development	21/7539	0.000393477	0.001513724
GO:0043537	negative regulation of blood vessel endothelial cell migration	21/7539	0.000393477	0.001513724
GO:0030042	actin filament depolymerization	32/7539	0.000405307	0.001555494
GO:0010906	regulation of glucose metabolic process	60/7539	0.000407825	0.001555494
GO:0003157	endocardium development	Oct-39	0.000408277	0.001555494
GO:0003207	cardiac chamber formation	Oct-39	0.000408277	0.001555494
GO:0032341	aldosterone metabolic process	Oct-39	0.000408277	0.001555494
GO:0035933	glucocorticoid secretion	Oct-39	0.000408277	0.001555494
GO:0043587	tongue morphogenesis	Oct-39	0.000408277	0.001555494
GO:0044557	relaxation of smooth muscle	Oct-39	0.000408277	0.001555494
GO:0060073	micturition	Oct-39	0.000408277	0.001555494
GO:0060453	regulation of gastric acid secretion	Oct-39	0.000408277	0.001555494
GO:0060872	semicircular canal development	Oct-39	0.000408277	0.001555494
GO:0070444	oligodendrocyte progenitor proliferation	Oct-39	0.000408277	0.001555494
GO:0070445	regulation of oligodendrocyte progenitor proliferation	Oct-39	0.000408277	0.001555494

GO:0072044	collecting duct development	Oct-39	0.000408277	0.001555494
GO:0086013	membrane repolarization during cardiac muscle cell action potential	Oct-39	0.000408277	0.001555494
GO:1902459	positive regulation of stem cell population maintenance	Oct-39	0.000408277	0.001555494
GO:2000058	regulation of ubiquitin-dependent protein catabolic process	72/7539	0.000417347	0.001589092
GO:0044843	cell cycle G1/S phase transition	88/7539	0.000418453	0.001592342
GO:0043407	negative regulation of MAP kinase activity	37/7539	0.000425134	0.00161679
GO:0097484	dendrite extension	25/7539	0.000426128	0.001617648
GO:1902742	apoptotic process involved in development	25/7539	0.000426128	0.001617648
GO:2001258	negative regulation of cation channel activity	25/7539	0.000426128	0.001617648
GO:0008589	regulation of smoothened signaling pathway	46/7539	0.000431144	0.001633967
GO:0048384	retinoic acid receptor signaling pathway	20/7539	0.000432581	0.001633967
GO:0048668	collateral sprouting	20/7539	0.000432581	0.001633967
GO:0051491	positive regulation of filopodium assembly	20/7539	0.000432581	0.001633967
GO:0060306	regulation of membrane repolarization	20/7539	0.000432581	0.001633967
GO:0090504	epiboly	20/7539	0.000432581	0.001633967
GO:1903859	regulation of dendrite extension	20/7539	0.000432581	0.001633967
GO:2000515	negative regulation of CD4-positive, alpha-beta T cell activation	20/7539	0.000432581	0.001633967
GO:0001818	negative regulation of cytokine production	110/7539	0.000432757	0.001633967

GO:0036473	cell death in response to oxidative stress	48/7539	0.000436093	0.00164558
GO:0044264	cellular polysaccharide metabolic process	50/7539	0.000437584	0.001650218
GO:0042445	hormone metabolic process	86/7539	0.000443325	0.001670874
GO:0045670	regulation of osteoclast differentiation	39/7539	0.000452094	0.001702905
GO:0034332	adherens junction organization	28/7539	0.000460298	0.001732773
GO:0007585	respiratory gaseous exchange by respiratory system	31/7539	0.000466096	0.001753556
GO:0036294	cellular response to decreased oxygen levels	59/7539	0.000469166	0.001764053
GO:0002335	mature B cell differentiation	19/7539	0.000472378	0.00177191
GO:0030947	regulation of vascular endothelial growth factor receptor signaling pathway	19/7539	0.000472378	0.00177191
GO:0043951	negative regulation of cAMP-mediated signaling	19/7539	0.000472378	0.00177191
GO:0050802	circadian sleep/wake cycle, sleep	19/7539	0.000472378	0.00177191
GO:0046209	nitric oxide metabolic process	41/7539	0.000473	0.001773188
GO:0002274	myeloid leukocyte activation	99/7539	0.000477973	0.001790768
GO:0042542	response to hydrogen peroxide	57/7539	0.000480652	0.001799736
GO:0006970	response to osmotic stress	43/7539	0.000488254	0.001827119
GO:0120193	tight junction organization	45/7539	0.000498354	0.00186381
GO:0010882	regulation of cardiac muscle contraction by calcium ion signaling	Nov-39	0.000509542	0.001895545
GO:0021903	rostrocaudal neural tube patterning	Nov-39	0.000509542	0.001895545

GO:0021984	adenohypophysis development	Nov-39	0.000509542	0.001895545
GO:0035791	platelet-derived growth factor receptor-beta signaling pathway	Nov-39	0.000509542	0.001895545
GO:0048505	regulation of timing of cell differentiation	Nov-39	0.000509542	0.001895545
GO:0048755	branching morphogenesis of a nerve	Nov-39	0.000509542	0.001895545
GO:0071371	cellular response to gonadotropin stimulus	Nov-39	0.000509542	0.001895545
GO:0099550	trans-synaptic signaling, modulating synaptic transmission	Nov-39	0.000509542	0.001895545
GO:1901841	regulation of high voltage-gated calcium channel activity	Nov-39	0.000509542	0.001895545
GO:0010460	positive regulation of heart rate	18/7539	0.000511595	0.001897591
GO:0045992	negative regulation of embryonic development	18/7539	0.000511595	0.001897591
GO:0060037	pharyngeal system development	18/7539	0.000511595	0.001897591
GO:0086005	ventricular cardiac muscle cell action potential	18/7539	0.000511595	0.001897591
GO:1900745	positive regulation of p38MAPK cascade	18/7539	0.000511595	0.001897591
GO:0030865	cortical cytoskeleton organization	33/7539	0.000521287	0.001929006
GO:0031018	endocrine pancreas development	33/7539	0.000521287	0.001929006
GO:0050854	regulation of antigen receptor-mediated signaling pathway	33/7539	0.000521287	0.001929006
GO:0050982	detection of mechanical stimulus	33/7539	0.000521287	0.001929006
GO:0007029	endoplasmic reticulum organization	38/7539	0.000522688	0.001933057
GO:0070527	platelet aggregation	27/7539	0.000526108	0.001944566

GO:2000351	regulation of endothelial cell apoptotic process	30/7539	0.000535899	0.001979598
GO:0090277	positive regulation of peptide hormone secretion	58/7539	0.000539743	0.001991466
GO:1905039	carboxylic acid transmembrane transport	58/7539	0.000539743	0.001991466
GO:0007292	female gamete generation	70/7539	0.00054625	0.002011667
GO:0051650	establishment of vesicle localization	70/7539	0.00054625	0.002011667
GO:0048635	negative regulation of muscle organ development	40/7539	0.000547032	0.002011667
GO:0060191	regulation of lipase activity	40/7539	0.000547032	0.002011667
GO:0140056	organelle localization by membrane tethering	40/7539	0.000547032	0.002011667
GO:0021801	cerebral cortex radial glia guided migration	17/7539	0.000548405	0.002011667
GO:0022030	telencephalon glial cell migration	17/7539	0.000548405	0.002011667
GO:0032303	regulation of icosanoid secretion	17/7539	0.000548405	0.002011667
GO:0072012	glomerulus vasculature development	17/7539	0.000548405	0.002011667
GO:0090189	regulation of branching involved in ureteric bud morphogenesis	17/7539	0.000548405	0.002011667
GO:0016050	vesicle organization	115/7539	0.000552889	0.002026938
GO:0005976	polysaccharide metabolic process	54/7539	0.000565096	0.002069286
GO:0043401	steroid hormone mediated signaling pathway	54/7539	0.000565096	0.002069286
GO:0030239	myofibril assembly	35/7539	0.000567395	0.002075297
GO:1903307	positive regulation of regulated secretory pathway	35/7539	0.000567395	0.002075297

GO:0007638	mechanosensory behavior	Dec-39	0.000574038	0.002088704
GO:0010454	negative regulation of cell fate commitment	Dec-39	0.000574038	0.002088704
GO:0035641	locomotory exploration behavior	Dec-39	0.000574038	0.002088704
GO:0048715	negative regulation of oligodendrocyte differentiation	Dec-39	0.000574038	0.002088704
GO:0071599	otic vesicle development	Dec-39	0.000574038	0.002088704
GO:0090051	negative regulation of cell migration involved in sprouting angiogenesis	Dec-39	0.000574038	0.002088704
GO:0097094	craniofacial suture morphogenesis	Dec-39	0.000574038	0.002088704
GO:1901386	negative regulation of voltage-gated calcium channel activity	Dec-39	0.000574038	0.002088704
GO:2001053	regulation of mesenchymal cell apoptotic process	Dec-39	0.000574038	0.002088704
GO:0030903	notochord development	16/7539	0.000580273	0.002107748
GO:0048670	regulation of collateral sprouting	16/7539	0.000580273	0.002107748
GO:1902656	calcium ion import into cytosol	16/7539	0.000580273	0.002107748
GO:0009408	response to heat	46/7539	0.000582214	0.002113579
GO:0045931	positive regulation of mitotic cell cycle	76/7539	0.000584409	0.002120328
GO:0032881	regulation of polysaccharide metabolic process	26/7539	0.00060083	0.002178656
GO:0072678	T cell migration	32/7539	0.000601256	0.00217895
GO:0048857	neural nucleus development	15/7539	0.000603784	0.002179353
GO:0051770	positive regulation of nitric-oxide synthase biosynthetic process	15/7539	0.000603784	0.002179353

GO:0060749	mammary gland alveolus development	15/7539	0.000603784	0.002179353
GO:0061377	mammary gland lobule development	15/7539	0.000603784	0.002179353
GO:0072215	regulation of metanephros development	15/7539	0.000603784	0.002179353
GO:0072273	metanephric nephron morphogenesis	15/7539	0.000603784	0.002179353
GO:2001026	regulation of endothelial cell chemotaxis	15/7539	0.000603784	0.002179353
GO:0003214	cardiac left ventricle morphogenesis	13/7539	0.000606747	0.002182561
GO:0009886	post-embryonic animal morphogenesis	13/7539	0.000606747	0.002182561
GO:0035633	maintenance of blood-brain barrier	13/7539	0.000606747	0.002182561
GO:0042487	regulation of odontogenesis of dentin-containing tooth	13/7539	0.000606747	0.002182561
GO:0060841	venous blood vessel development	13/7539	0.000606747	0.002182561
GO:0072077	renal vesicle morphogenesis	13/7539	0.000606747	0.002182561
GO:0030866	cortical actin cytoskeleton organization	22/7539	0.000610984	0.002194051
GO:0051647	nucleus localization	22/7539	0.000610984	0.002194051
GO:0070873	regulation of glycogen metabolic process	22/7539	0.000610984	0.002194051
GO:0099111	microtubule-based transport	85/7539	0.000613134	0.002195341
GO:0006883	cellular sodium ion homeostasis	14/7539	0.000614473	0.002195341
GO:0021535	cell migration in hindbrain	14/7539	0.000614473	0.002195341
GO:0032095	regulation of response to food	14/7539	0.000614473	0.002195341

GO:0032252	secretory granule localization	14/7539	0.000614473	0.002195341
GO:0046851	negative regulation of bone remodeling	14/7539	0.000614473	0.002195341
GO:0071625	vocalization behavior	14/7539	0.000614473	0.002195341
GO:1903909	regulation of receptor clustering	14/7539	0.000614473	0.002195341
GO:2000114	regulation of establishment of cell polarity	14/7539	0.000614473	0.002195341
GO:0045843	negative regulation of striated muscle tissue development	39/7539	0.00063279	0.002259505
GO:0045621	positive regulation of lymphocyte differentiation	55/7539	0.000637194	0.002273944
GO:0060389	pathway-restricted SMAD protein phosphorylation	34/7539	0.00065564	0.002338451
GO:0035773	insulin secretion involved in cellular response to glucose stimulus	45/7539	0.000672893	0.00239863
GO:1901615	organic hydroxy compound metabolic process	195/7539	0.00067546	0.00240642
GO:0007026	negative regulation of microtubule depolymerization	21/7539	0.000685181	0.002432606
GO:0009187	cyclic nucleotide metabolic process	21/7539	0.000685181	0.002432606
GO:0016339	calcium-dependent cell-cell adhesion via plasma membrane cell adhesion molecules	21/7539	0.000685181	0.002432606
GO:0034110	regulation of homotypic cell-cell adhesion	21/7539	0.000685181	0.002432606
GO:1903203	regulation of oxidative stress-induced neuron death	21/7539	0.000685181	0.002432606
GO:0032350	regulation of hormone metabolic process	25/7539	0.000685507	0.002432606
GO:0046621	negative regulation of organ growth	25/7539	0.000685507	0.002432606
GO:1901617	organic hydroxy compound biosynthetic process	96/7539	0.000689455	0.00244524

GO:0072577	endothelial cell apoptotic process	31/7539	0.000693513	0.00245825
GO:0032891	negative regulation of organic acid transport	20/7539	0.000765591	0.00270917
GO:1901099	negative regulation of signal transduction in absence of ligand	20/7539	0.000765591	0.00270917
GO:2001240	negative regulation of extrinsic apoptotic signaling pathway in absence of ligand	20/7539	0.000765591	0.00270917
GO:0006476	protein deacetylation	48/7539	0.000773656	0.00273618
GO:0072348	sulfur compound transport	24/7539	0.000781241	0.0027599
GO:0072583	clathrin-dependent endocytosis	24/7539	0.000781241	0.0027599
GO:0051783	regulation of nuclear division	83/7539	0.000790057	0.00278950
GO:0050873	brown fat cell differentiation	30/7539	0.000799941	0.0028228
GO:0002763	positive regulation of myeloid leukocyte differentiation	35/7539	0.000808774	0.00285080
GO:0046637	regulation of alpha-beta T cell differentiation	35/7539	0.000808774	0.00285080
GO:0014888	striated muscle adaptation	27/7539	0.000813256	0.00285862
GO:0033173	calcineurin-NFAT signaling cascade	27/7539	0.000813256	0.00285862
GO:0042755	eating behavior	27/7539	0.000813256	0.00285862
GO:0043550	regulation of lipid kinase activity	27/7539	0.000813256	0.00285862
GO:0090102	cochlea development	27/7539	0.000813256	0.00285862
GO:0001958	endochondral ossification	19/7539	0.000851678	0.00298536
GO:0007097	nuclear migration	19/7539	0.000851678	0.00298536

GO:0033687	osteoblast proliferation	19/7539	0.000851678	0.002985364
GO:0036075	replacement ossification	19/7539	0.000851678	0.002985364
GO:0044062	regulation of excretion	19/7539	0.000851678	0.002985364
GO:1903036	positive regulation of response to wounding	39/7539	0.000874471	0.00306356
GO:0032640	tumor necrosis factor production	58/7539	0.000884043	0.003095376
GO:0034105	positive regulation of tissue remodeling	23/7539	0.000889175	0.003109894
GO:0043551	regulation of phosphatidylinositol 3-kinase activity	23/7539	0.000889175	0.003109894
GO:0003333	amino acid transmembrane transport	41/7539	0.00089138	0.003112434
GO:0032272	negative regulation of protein polymerization	41/7539	0.00089138	0.003112434
GO:0061178	regulation of insulin secretion involved in cellular response to glucose stimulus	41/7539	0.00089138	0.003112434
GO:0045913	positive regulation of carbohydrate metabolic process	43/7539	0.000899323	0.003138433
GO:0019221	cytokine-mediated signaling pathway	148/7539	0.000903813	0.003152361
GO:1904705	regulation of vascular associated smooth muscle cell proliferation	29/7539	0.000922707	0.003214707
GO:1990874	vascular associated smooth muscle cell proliferation	29/7539	0.000922707	0.003214707
GO:0048278	vesicle docking	34/7539	0.000935878	0.003258796
GO:0007214	gamma-aminobutyric acid signaling pathway	18/7539	0.000942365	0.003275962
GO:0045187	regulation of circadian sleep/wake cycle, sleep	18/7539	0.000942365	0.003275962
GO:0048643	positive regulation of skeletal muscle tissue development	18/7539	0.000942365	0.003275962

GO:1904951	positive regulation of establishment of protein localization	146/7539	0.000972468	0.003376588
GO:0035601	protein deacylation	52/7539	0.000972915	0.003376588
GO:0098732	macromolecule deacylation	52/7539	0.000972915	0.003376588
GO:0043473	pigmentation	50/7539	0.000996922	0.003458004
GO:0010831	positive regulation of myotube differentiation	22/7539	0.001010448	0.003497236
GO:0045601	regulation of endothelial cell differentiation	22/7539	0.001010448	0.003497236
GO:0051968	positive regulation of synaptic transmission, glutamatergic	22/7539	0.001010448	0.003497236
GO:0060674	placenta blood vessel development	22/7539	0.001010448	0.003497236
GO:0010659	cardiac muscle cell apoptotic process	31/7539	0.001012598	0.003502758
GO:0016048	detection of temperature stimulus	17/7539	0.001035801	0.003557679
GO:0032528	microvillus organization	17/7539	0.001035801	0.003557679
GO:0042753	positive regulation of circadian rhythm	17/7539	0.001035801	0.003557679
GO:0045780	positive regulation of bone resorption	17/7539	0.001035801	0.003557679
GO:0046852	positive regulation of bone remodeling	17/7539	0.001035801	0.003557679
GO:0046885	regulation of hormone biosynthetic process	17/7539	0.001035801	0.003557679
GO:0048745	smooth muscle tissue development	17/7539	0.001035801	0.003557679
GO:0051767	nitric-oxide synthase biosynthetic process	17/7539	0.001035801	0.003557679
GO:0051769	regulation of nitric-oxide synthase biosynthetic process	17/7539	0.001035801	0.003557679

GO:0060343	trabecula formation	17/7539	0.001035801	0.003557679
GO:1900120	regulation of receptor binding	17/7539	0.001035801	0.003557679
GO:1904994	regulation of leukocyte adhesion to vascular endothelial cell	17/7539	0.001035801	0.003557679
GO:2000727	positive regulation of cardiac muscle cell differentiation	17/7539	0.001035801	0.003557679
GO:2001057	reactive nitrogen species metabolic process	42/7539	0.001039991	0.003570127
GO:0003139	secondary heart field specification	Sep-39	0.0010626	0.00362605
GO:0032342	aldosterone biosynthetic process	Sep-39	0.0010626	0.00362605
GO:0035881	amacrine cell differentiation	Sep-39	0.0010626	0.00362605
GO:0060013	righting reflex	Sep-39	0.0010626	0.00362605
GO:0060174	limb bud formation	Sep-39	0.0010626	0.00362605
GO:0060600	dichotomous subdivision of an epithelial terminal unit	Sep-39	0.0010626	0.00362605
GO:0060900	embryonic camera-type eye formation	Sep-39	0.0010626	0.00362605
GO:0072172	mesonephric tubule formation	Sep-39	0.0010626	0.00362605
GO:1902548	negative regulation of cellular response to vascular endothelial growth factor stimulus	Sep-39	0.0010626	0.00362605
GO:1904058	positive regulation of sensory perception of pain	Sep-39	0.0010626	0.00362605
GO:2000849	regulation of glucocorticoid secretion	Sep-39	0.0010626	0.00362605
GO:0002448	mast cell mediated immunity	28/7539	0.001064305	0.003627947
GO:0030857	negative regulation of epithelial cell differentiation	28/7539	0.001064305	0.003627947

GO:0015749	monosaccharide transmembrane transport	53/7539	0.001087945	0.003706529
GO:0006638	neutral lipid metabolic process	63/7539	0.001122017	0.003818487
GO:0071453	cellular response to oxygen levels	63/7539	0.001122017	0.003818487
GO:0071312	cellular response to alkaloid	16/7539	0.00112905	0.003838285
GO:2000193	positive regulation of fatty acid transport	16/7539	0.00112905	0.003838285
GO:0031069	hair follicle morphogenesis	21/7539	0.001146142	0.003885925
GO:0035909	aorta morphogenesis	21/7539	0.001146142	0.003885925
GO:0048009	insulin-like growth factor receptor signaling pathway	21/7539	0.001146142	0.003885925
GO:0060351	cartilage development involved in endochondral bone morphogenesis	21/7539	0.001146142	0.003885925
GO:2000171	negative regulation of dendrite development	21/7539	0.001146142	0.003885925
GO:0031331	positive regulation of cellular catabolic process	157/7539	0.001160772	0.003933415
GO:0099518	vesicle cytoskeletal trafficking	37/7539	0.00117199	0.003969298
GO:0032675	regulation of interleukin-6 production	69/7539	0.001188926	0.004024499
GO:0070830	bicellular tight junction assembly	41/7539	0.001202916	0.004069672
GO:0030879	mammary gland development	72/7539	0.001209258	0.004088937
GO:0003283	atrial septum development	15/7539	0.001217669	0.00409981
GO:0010743	regulation of macrophage derived foam cell differentiation	15/7539	0.001217669	0.00409981
GO:0021692	cerebellar Purkinje cell layer morphogenesis	15/7539	0.001217669	0.00409981

GO:0032098	regulation of appetite	15/7539	0.001217669	0.00409981
GO:0051969	regulation of transmission of nerve impulse	15/7539	0.001217669	0.00409981
GO:0090190	positive regulation of branching involved in ureteric bud morphogenesis	15/7539	0.001217669	0.00409981
GO:0097062	dendritic spine maintenance	15/7539	0.001217669	0.00409981
GO:0099149	regulation of postsynaptic neurotransmitter receptor internalization	15/7539	0.001217669	0.00409981
GO:0002762	negative regulation of myeloid leukocyte differentiation	27/7539	0.001227599	0.00413104
GO:0040018	positive regulation of multicellular organism growth	24/7539	0.001229805	0.004131857
GO:0055026	negative regulation of cardiac muscle tissue development	24/7539	0.001229805	0.004131857
GO:1903792	negative regulation of anion transport	24/7539	0.001229805	0.004131857
GO:0008645	hexose transmembrane transport	52/7539	0.00125174	0.004180083
GO:0008212	mineralocorticoid metabolic process	Oct-39	0.001253542	0.004180083
GO:0021670	lateral ventricle development	Oct-39	0.001253542	0.004180083
GO:0021859	pyramidal neuron differentiation	Oct-39	0.001253542	0.004180083
GO:0021877	forebrain neuron fate commitment	Oct-39	0.001253542	0.004180083
GO:0032486	Rap protein signal transduction	Oct-39	0.001253542	0.004180083
GO:0035810	positive regulation of urine volume	Oct-39	0.001253542	0.004180083
GO:0055119	relaxation of cardiac muscle	Oct-39	0.001253542	0.004180083
GO:0060452	positive regulation of cardiac muscle contraction	Oct-39	0.001253542	0.004180083

GO:0061140	lung secretory cell differentiation	Oct-39	0.001253542	0.004180083
GO:0072160	nephron tubule epithelial cell differentiation	Oct-39	0.001253542	0.004180083
GO:1900025	negative regulation of substrate adhesion-dependent cell spreading	Oct-39	0.001253542	0.004180083
GO:2001054	negative regulation of mesenchymal cell apoptotic process	Oct-39	0.001253542	0.004180083
GO:0010658	striated muscle cell apoptotic process	32/7539	0.001254091	0.004180083
GO:0060393	regulation of pathway-restricted SMAD protein phosphorylation	32/7539	0.001254091	0.004180083
GO:0007249	I-kappaB kinase/NF-kappaB signaling	91/7539	0.001262115	0.004204609
GO:0006639	acylglycerol metabolic process	62/7539	0.001283679	0.004274189
GO:0050714	positive regulation of protein secretion	85/7539	0.00129414	0.004285601
GO:0007190	activation of adenylate cyclase activity	14/7539	0.001295162	0.004285601
GO:0032305	positive regulation of icosanoid secretion	14/7539	0.001295162	0.004285601
GO:0032332	positive regulation of chondrocyte differentiation	14/7539	0.001295162	0.004285601
GO:0033605	positive regulation of catecholamine secretion	14/7539	0.001295162	0.004285601
GO:0051900	regulation of mitochondrial depolarization	14/7539	0.001295162	0.004285601
GO:0060055	angiogenesis involved in wound healing	14/7539	0.001295162	0.004285601
GO:0060456	positive regulation of digestive system process	14/7539	0.001295162	0.004285601
GO:0072170	metanephric tubule development	14/7539	0.001295162	0.004285601
GO:0090036	regulation of protein kinase C signaling	14/7539	0.001295162	0.004285601

GO:2001224	positive regulation of neuron migration	14/7539	0.001295162	0.004285601
GO:0098868	bone growth	20/7539	0.001297182	0.004285601
GO:0099563	modification of synaptic structure	20/7539	0.001297182	0.004285601
GO:1900274	regulation of phospholipase C activity	20/7539	0.001297182	0.004285601
GO:0032635	interleukin-6 production	73/7539	0.001297289	0.004285601
GO:0042129	regulation of T cell proliferation	76/7539	0.001305994	0.004312101
GO:0032680	regulation of tumor necrosis factor production	55/7539	0.001338834	0.004404289
GO:0003188	heart valve formation	Nov-39	0.001350475	0.004404289
GO:0014049	positive regulation of glutamate secretion	Nov-39	0.001350475	0.004404289
GO:0019755	one-carbon compound transport	Nov-39	0.001350475	0.004404289
GO:0045760	positive regulation of action potential	Nov-39	0.001350475	0.004404289
GO:0061307	cardiac neural crest cell differentiation involved in heart development	Nov-39	0.001350475	0.004404289
GO:0061318	renal filtration cell differentiation	Nov-39	0.001350475	0.004404289
GO:0061430	bone trabecula morphogenesis	Nov-39	0.001350475	0.004404289
GO:0072112	glomerular visceral epithelial cell differentiation	Nov-39	0.001350475	0.004404289
GO:0072283	metanephric renal vesicle morphogenesis	Nov-39	0.001350475	0.004404289
GO:0072311	glomerular epithelial cell differentiation	Nov-39	0.001350475	0.004404289
GO:1903818	positive regulation of voltage-gated potassium channel activity	Nov-39	0.001350475	0.004404289

GO:2000811	negative regulation of anoikis	Nov-39	0.001350475	0.004404289
GO:0021924	cell proliferation in external granule layer	13/7539	0.001352305	0.004404289
GO:0021930	cerebellar granule cell precursor proliferation	13/7539	0.001352305	0.004404289
GO:0032352	positive regulation of hormone metabolic process	13/7539	0.001352305	0.004404289
GO:0045956	positive regulation of calcium ion-dependent exocytosis	13/7539	0.001352305	0.004404289
GO:0048484	enteric nervous system development	13/7539	0.001352305	0.004404289
GO:0060044	negative regulation of cardiac muscle cell proliferation	13/7539	0.001352305	0.004404289
GO:0060712	spongiotrophoblast layer development	13/7539	0.001352305	0.004404289
GO:0071605	monocyte chemotactic protein-1 production	13/7539	0.001352305	0.004404289
GO:0071637	regulation of monocyte chemotactic protein-1 production	13/7539	0.001352305	0.004404289
GO:0097623	potassium ion export across plasma membrane	13/7539	0.001352305	0.004404289
GO:1900451	positive regulation of glutamate receptor signaling pathway	13/7539	0.001352305	0.004404289
GO:2000251	positive regulation of actin cytoskeleton reorganization	13/7539	0.001352305	0.004404289
GO:0033619	membrane protein proteolysis	29/7539	0.001354146	0.004404289
GO:0035306	positive regulation of dephosphorylation	29/7539	0.001354146	0.004404289
GO:0042531	positive regulation of tyrosine phosphorylation of STAT protein	29/7539	0.001354146	0.004404289
GO:0045773	positive regulation of axon extension	29/7539	0.001354146	0.004404289
GO:0061045	negative regulation of wound healing	36/7539	0.001357347	0.004412426

GO:0043900	regulation of multi-organism process	44/7539	0.001375523	0.004446802
GO:0002024	diet induced thermogenesis	Dec-39	0.001376374	0.004446802
GO:0010752	regulation of cGMP-mediated signaling	Dec-39	0.001376374	0.004446802
GO:0032516	positive regulation of phosphoprotein phosphatase activity	Dec-39	0.001376374	0.004446802
GO:0032957	inositol trisphosphate metabolic process	Dec-39	0.001376374	0.004446802
GO:0045793	positive regulation of cell size	Dec-39	0.001376374	0.004446802
GO:0045989	positive regulation of striated muscle contraction	Dec-39	0.001376374	0.004446802
GO:0060413	atrial septum morphogenesis	Dec-39	0.001376374	0.004446802
GO:0060973	cell migration involved in heart development	Dec-39	0.001376374	0.004446802
GO:0072234	metanephric nephron tubule development	Dec-39	0.001376374	0.004446802
GO:1900119	positive regulation of execution phase of apoptosis	Dec-39	0.001376374	0.004446802
GO:1900452	regulation of long-term synaptic depression	Dec-39	0.001376374	0.004446802
GO:0035304	regulation of protein dephosphorylation	42/7539	0.0013888 4	0.004484782
GO:0034219	carbohydrate transmembrane transport	53/7539	0.001390765	0.004488702
GO:0002686	negative regulation of leukocyte migration	23/7539	0.001409931	0.004539469
GO:0048730	epidermis morphogenesis	23/7539	0.001409931	0.004539469
GO:0051281	positive regulation of release of sequestered calcium ion into cytosol	23/7539	0.001409931	0.004539469
GO:1902991	regulation of amyloid precursor protein catabolic process	23/7539	0.001409931	0.004539469

GO:0032609	interferon-gamma production	58/7539	0.001410809	0.004539469
GO:0071706	tumor necrosis factor superfamily cytokine production	58/7539	0.001410809	0.004539469
GO:0090279	regulation of calcium ion import	26/7539	0.001415874	0.004553446
GO:0031645	negative regulation of nervous system process	19/7539	0.001464204	0.004694525
GO:0042749	regulation of circadian sleep/wake cycle	19/7539	0.001464204	0.004694525
GO:0060561	apoptotic process involved in morphogenesis	19/7539	0.001464204	0.004694525
GO:0060669	embryonic placenta morphogenesis	19/7539	0.001464204	0.004694525
GO:1902473	regulation of protein localization to synapse	19/7539	0.001464204	0.004694525
GO:2000352	negative regulation of endothelial cell apoptotic process	19/7539	0.001464204	0.004694525
GO:0090068	positive regulation of cell cycle process	105/7539	0.001484661	0.004757697
GO:0045921	positive regulation of exocytosis	49/7539	0.001486136	0.004760009
GO:0034121	regulation of toll-like receptor signaling pathway	33/7539	0.001524167	0.004876871
GO:0042246	tissue regeneration	33/7539	0.001524167	0.004876871
GO:1902882	regulation of response to oxidative stress	47/7539	0.001527256	0.004884278
GO:0007088	regulation of mitotic nuclear division	70/7539	0.001565957	0.005005512
GO:0032388	positive regulation of intracellular transport	79/7539	0.001574749	0.005031068
GO:0030203	glycosaminoglycan metabolic process	41/7539	0.001605813	0.005127718
GO:0032885	regulation of polysaccharide biosynthetic process	22/7539	0.001615172	0.005149788

GO:0050931	pigment cell differentiation	22/7539	0.001615172	0.005149788
GO:1900026	positive regulation of substrate adhesion-dependent cell spreading	22/7539	0.001615172	0.005149788
GO:0032386	regulation of intracellular transport	132/7539	0.001625529	0.005180195
GO:0010518	positive regulation of phospholipase activity	25/7539	0.001632895	0.00519842
GO:1901985	positive regulation of protein acetylation	25/7539	0.001632895	0.00519842
GO:0071326	cellular response to monosaccharide stimulus	65/7539	0.001634312	0.005200309
GO:0001759	organ induction	18/7539	0.001647344	0.005228598
GO:0005979	regulation of glycogen biosynthetic process	18/7539	0.001647344	0.005228598
GO:0010962	regulation of glucan biosynthetic process	18/7539	0.001647344	0.005228598
GO:0050951	sensory perception of temperature stimulus	18/7539	0.001647344	0.005228598
GO:0051957	positive regulation of amino acid transport	18/7539	0.001647344	0.005228598
GO:0032663	regulation of interleukin-2 production	30/7539	0.001682342	0.005336998
GO:0032760	positive regulation of tumor necrosis factor production	32/7539	0.00176646	0.005601037
GO:0031668	cellular response to extracellular stimulus	91/7539	0.001770183	0.005610022
GO:0051606	detection of stimulus	102/7539	0.001786608	0.005659235
GO:0006643	membrane lipid metabolic process	75/7539	0.001791325	0.005671331
GO:0008089	anterograde axonal transport	27/7539	0.001812246	0.005731818
GO:0043303	mast cell degranulation	27/7539	0.001812246	0.005731818

GO:0010827	regulation of glucose transmembrane transport	42/7539	0.001835791	0.005794673
GO:0031960	response to corticosteroid	42/7539	0.001835791	0.005794673
GO:0032526	response to retinoic acid	42/7539	0.001835791	0.005794673
GO:0050672	negative regulation of lymphocyte proliferation	42/7539	0.001835791	0.005794673
GO:1904659	glucose transmembrane transport	51/7539	0.001837429	0.005796946
GO:0045737	positive regulation of cyclin-dependent protein serine/threonine kinase activity	17/7539	0.001845938	0.005811657
GO:0051446	positive regulation of meiotic cell cycle	17/7539	0.001845938	0.005811657
GO:1901020	negative regulation of calcium ion transmembrane transporter activity	17/7539	0.001845938	0.005811657
GO:1903861	positive regulation of dendrite extension	17/7539	0.001845938	0.005811657
GO:0001782	B cell homeostasis	21/7539	0.001848536	0.005811657
GO:0009954	proximal/distal pattern formation	21/7539	0.001848536	0.005811657
GO:0021680	cerebellar Purkinje cell layer development	21/7539	0.001848536	0.005811657
GO:0072384	organelle transport along microtubule	40/7539	0.001857063	0.005835558
GO:0005977	glycogen metabolic process	38/7539	0.001864021	0.005845786
GO:0006073	cellular glucan metabolic process	38/7539	0.001864021	0.005845786
GO:0008625	extrinsic apoptotic signaling pathway via death domain receptors	38/7539	0.001864021	0.005845786
GO:0044042	glucan metabolic process	38/7539	0.001864021	0.005845786
GO:0006024	glycosaminoglycan biosynthetic process	24/7539	0.001882962	0.005899326

GO:0070849	response to epidermal growth factor	24/7539	0.001882962	0.005899326
GO:0043122	regulation of I-kappaB kinase/NF-kappaB signaling	79/7539	0.001888732	0.005914466
GO:0045620	negative regulation of lymphocyte differentiation	29/7539	0.001949404	0.006095388
GO:0051932	synaptic transmission, GABAergic	29/7539	0.001949404	0.006095388
GO:0071675	regulation of mononuclear cell migration	29/7539	0.001949404	0.006095388
GO:0032755	positive regulation of interleukin-6 production	47/7539	0.001971608	0.006161762
GO:0042698	ovulation cycle	31/7539	0.002047963	0.006394036
GO:0009713	catechol-containing compound biosynthetic process	16/7539	0.002058084	0.006394036
GO:0010592	positive regulation of lamellipodium assembly	16/7539	0.002058084	0.006394036
GO:0032104	regulation of response to extracellular stimulus	16/7539	0.002058084	0.006394036
GO:0032107	regulation of response to nutrient levels	16/7539	0.002058084	0.006394036
GO:0042423	catecholamine biosynthetic process	16/7539	0.002058084	0.006394036
GO:0060065	uterus development	16/7539	0.002058084	0.006394036
GO:0070168	negative regulation of biomineral tissue development	16/7539	0.002058084	0.006394036
GO:0098901	regulation of cardiac muscle cell action potential	16/7539	0.002058084	0.006394036
GO:0110150	negative regulation of biomineralization	16/7539	0.002058084	0.006394036
GO:1901380	negative regulation of potassium ion transmembrane transport	16/7539	0.002058084	0.006394036
GO:2000679	positive regulation of transcription regulatory region DNA binding	16/7539	0.002058084	0.006394036

GO:0040014	regulation of multicellular organism growth	43/7539	0.00208118	0.006462613
GO:0021532	neural tube patterning	26/7539	0.002097095	0.00650563
GO:0032941	secretion by tissue	26/7539	0.002097095	0.00650563
GO:0032094	response to food	20/7539	0.002113183	0.006545891
GO:0045879	negative regulation of smoothened signaling pathway	20/7539	0.002113183	0.006545891
GO:0055083	monovalent inorganic anion homeostasis	20/7539	0.002113183	0.006545891
GO:0120192	tight junction assembly	41/7539	0.00212134	0.006567934
GO:1903555	regulation of tumor necrosis factor superfamily cytokine production	55/7539	0.002122789	0.006569199
GO:0006140	regulation of nucleotide metabolic process	39/7539	0.002148085	0.006640969
GO:0043525	positive regulation of neuron apoptotic process	39/7539	0.002148085	0.006640969
GO:0000271	polysaccharide biosynthetic process	37/7539	0.00215811	0.006668697
GO:0006509	membrane protein ectodomain proteolysis	23/7539	0.002170977	0.006701896
GO:0016601	Rac protein signal transduction	23/7539	0.002170977	0.006701896
GO:0042177	negative regulation of protein catabolic process	58/7539	0.00219632	0.006776818
GO:0046834	lipid phosphorylation	28/7539	0.002259589	0.006968629
GO:0008090	retrograde axonal transport	15/7539	0.002279994	0.007011011
GO:0015732	prostaglandin transport	15/7539	0.002279994	0.007011011
GO:0021889	olfactory bulb interneuron differentiation	15/7539	0.002279994	0.007011011

GO:0030728	ovulation	15/7539	0.002279994	0.007011011
GO:0097150	neuronal stem cell population maintenance	15/7539	0.002279994	0.007011011
GO:0099623	regulation of cardiac muscle cell membrane repolarization	15/7539	0.002279994	0.007011011
GO:0071331	cellular response to hexose stimulus	64/7539	0.002284564	0.007021643
GO:0016051	carbohydrate biosynthetic process	83/7539	0.002338766	0.007184734
GO:0070664	negative regulation of leukocyte proliferation	44/7539	0.002341496	0.007189621
GO:0032945	negative regulation of mononuclear cell proliferation	42/7539	0.002402677	0.00737031
GO:0071674	mononuclear cell migration	42/7539	0.002402677	0.00737031
GO:0000186	activation of MAPKK activity	19/7539	0.002412322	0.007385542
GO:0045616	regulation of keratinocyte differentiation	19/7539	0.002412322	0.007385542
GO:0098884	postsynaptic neurotransmitter receptor internalization	19/7539	0.002412322	0.007385542
GO:0140239	postsynaptic endocytosis	19/7539	0.002412322	0.007385542
GO:0031667	response to nutrient levels	137/7539	0.002419618	0.007404289
GO:0043902	positive regulation of multi-organism process	25/7539	0.002427188	0.007413081
GO:0045214	sarcomere organization	25/7539	0.002427188	0.007413081
GO:0045581	negative regulation of T cell differentiation	25/7539	0.002427188	0.007413081
GO:0120163	negative regulation of cold-induced thermogenesis	25/7539	0.002427188	0.007413081
GO:1903557	positive regulation of tumor necrosis factor superfamily cytokine production	32/7539	0.002449698	0.007477219

GO:0022406	membrane docking	40/7539	0.002451742	0.007477219
GO:0051384	response to glucocorticoid	40/7539	0.002451742	0.007477219
GO:0045428	regulation of nitric oxide biosynthetic process	34/7539	0.002489156	0.00758766
GO:0031649	heat generation	14/7539	0.002505073	0.007610456
GO:0048339	paraxial mesoderm development	14/7539	0.002505073	0.007610456
GO:0051797	regulation of hair follicle development	14/7539	0.002505073	0.007610456
GO:0060602	branch elongation of an epithelium	14/7539	0.002505073	0.007610456
GO:0072111	cell proliferation involved in kidney development	14/7539	0.002505073	0.007610456
GO:0099625	ventricular cardiac muscle cell membrane repolarization	14/7539	0.002505073	0.007610456
GO:1903514	release of sequestered calcium ion into cytosol by endoplasmic reticulum	14/7539	0.002505073	0.007610456
GO:0001776	leukocyte homeostasis	52/7539	0.002551893	0.007748965
GO:0071333	cellular response to glucose stimulus	63/7539	0.002601626	0.007896185
GO:0007599	hemostasis	72/7539	0.002606866	0.007908288
GO:0070665	positive regulation of leukocyte proliferation	66/7539	0.002618429	0.007939549
GO:0002279	mast cell activation involved in immune response	27/7539	0.002620022	0.007940567
GO:0045930	negative regulation of mitotic cell cycle	97/7539	0.002694201	0.008095877
GO:0006665	sphingolipid metabolic process	58/7539	0.002715979	0.008095877
GO:0002315	marginal zone B cell differentiation	Aug-39	0.002720131	0.008095877

GO:0003211	cardiac ventricle formation	Aug-39	0.002720131	0.008095877
GO:0010692	regulation of alkaline phosphatase activity	Aug-39	0.002720131	0.008095877
GO:0010745	negative regulation of macrophage derived foam cell differentiation	Aug-39	0.002720131	0.008095877
GO:0022027	interkinetic nuclear migration	Aug-39	0.002720131	0.008095877
GO:0032224	positive regulation of synaptic transmission, cholinergic	Aug-39	0.002720131	0.008095877
GO:0032354	response to follicle-stimulating hormone	Aug-39	0.002720131	0.008095877
GO:0032650	regulation of interleukin-1 alpha production	Aug-39	0.002720131	0.008095877
GO:0032836	glomerular basement membrane development	Aug-39	0.002720131	0.008095877
GO:0045607	regulation of inner ear auditory receptor cell differentiation	Aug-39	0.002720131	0.008095877
GO:0045631	regulation of mechanoreceptor differentiation	Aug-39	0.002720131	0.008095877
GO:0045762	positive regulation of adenylate cyclase activity	Aug-39	0.002720131	0.008095877
GO:0060297	regulation of sarcomere organization	Aug-39	0.002720131	0.008095877
GO:0060379	cardiac muscle cell myoblast differentiation	Aug-39	0.002720131	0.008095877
GO:0060484	lung-associated mesenchyme development	Aug-39	0.002720131	0.008095877
GO:0060513	prostatic bud formation	Aug-39	0.002720131	0.008095877
GO:0060710	chorio-allantoic fusion	Aug-39	0.002720131	0.008095877
GO:0072017	distal tubule development	Aug-39	0.002720131	0.008095877
GO:0072070	loop of Henle development	Aug-39	0.002720131	0.008095877

GO:0072203	cell proliferation involved in metanephros development	Aug-39	0.002720131	0.008095877
GO:0098953	receptor diffusion trapping	Aug-39	0.002720131	0.008095877
GO:0098970	postsynaptic neurotransmitter receptor diffusion trapping	Aug-39	0.002720131	0.008095877
GO:0099541	trans-synaptic signaling by lipid	Aug-39	0.002720131	0.008095877
GO:0099542	trans-synaptic signaling by endocannabinoid	Aug-39	0.002720131	0.008095877
GO:0099628	neurotransmitter receptor diffusion trapping	Aug-39	0.002720131	0.008095877
GO:1902959	regulation of aspartic-type endopeptidase activity involved in amyloid precursor protein catabolic process	Aug-39	0.002720131	0.008095877
GO:1905962	glutamatergic neuron differentiation	Aug-39	0.002720131	0.008095877
GO:1990264	peptidyl-tyrosine dephosphorylation involved in inactivation of protein kinase activity	Aug-39	0.002720131	0.008095877
GO:2000543	positive regulation of gastrulation	Aug-39	0.002720131	0.008095877
GO:2000807	regulation of synaptic vesicle clustering	Aug-39	0.002720131	0.008095877
GO:2000980	regulation of inner ear receptor cell differentiation	Aug-39	0.002720131	0.008095877
GO:0001946	lymphangiogenesis	13/7539	0.002722564	0.008095877
GO:0021534	cell proliferation in hindbrain	13/7539	0.002722564	0.008095877
GO:0032310	prostaglandin secretion	13/7539	0.002722564	0.008095877
GO:0035584	calcium-mediated signaling using intracellular calcium source	13/7539	0.002722564	0.008095877
GO:0048172	regulation of short-term neuronal synaptic plasticity	13/7539	0.002722564	0.008095877
GO:0060252	positive regulation of glial cell proliferation	13/7539	0.002722564	0.008095877

GO:0090030	regulation of steroid hormone biosynthetic process	13/7539	0.002722564	0.008095877
GO:0043276	anoikis	18/7539	0.002749035	0.008163057
GO:1904031	positive regulation of cyclin-dependent protein kinase activity	18/7539	0.002749035	0.008163057
GO:1905523	positive regulation of macrophage migration	18/7539	0.002749035	0.008163057
GO:0032964	collagen biosynthetic process	29/7539	0.002755734	0.008179101
GO:1900180	regulation of protein localization to nucleus	61/7539	0.002761304	0.008191782
GO:0044728	DNA methylation or demethylation	41/7539	0.00277422	0.008226233
GO:0061756	leukocyte adhesion to vascular endothelial cell	24/7539	0.002809788	0.008327789
GO:0032228	regulation of synaptic transmission, GABAergic	21/7539	0.002883821	0.00853519
GO:0070528	protein kinase C signaling	21/7539	0.002883821	0.00853519
GO:2000249	regulation of actin cytoskeleton reorganization	21/7539	0.002883821	0.00853519
GO:0042116	macrophage activation	46/7539	0.002904729	0.008589063
GO:0006833	water transport	Dec-39	0.002915629	0.008589063
GO:0010919	regulation of inositol phosphate biosynthetic process	Dec-39	0.002915629	0.008589063
GO:0021542	dentate gyrus development	Dec-39	0.002915629	0.008589063
GO:0021702	cerebellar Purkinje cell differentiation	Dec-39	0.002915629	0.008589063
GO:0032793	positive regulation of CREB transcription factor activity	Dec-39	0.002915629	0.008589063
GO:0045779	negative regulation of bone resorption	Dec-39	0.002915629	0.008589063

GO:0060947	cardiac vascular smooth muscle cell differentiation	Dec-39	0.002915629	0.008589063
GO:0071696	ectodermal placode development	Dec-39	0.002915629	0.008589063
GO:1901881	positive regulation of protein depolymerization	Dec-39	0.002915629	0.008589063
GO:0007596	blood coagulation	71/7539	0.002952306	0.008693053
GO:0010821	regulation of mitochondrion organization	62/7539	0.002961974	0.008713393
GO:0046488	phosphatidylinositol metabolic process	62/7539	0.002961974	0.008713393
GO:0048285	organelle fission	181/7539	0.00300882	0.00882236
GO:0003129	heart induction	Sep-39	0.003025568	0.00882236
GO:0003337	mesenchymal to epithelial transition involved in metanephros morphogenesis	Sep-39	0.003025568	0.00882236
GO:0006705	mineralocorticoid biosynthetic process	Sep-39	0.003025568	0.00882236
GO:0010459	negative regulation of heart rate	Sep-39	0.003025568	0.00882236
GO:0014745	negative regulation of muscle adaptation	Sep-39	0.003025568	0.00882236
GO:0021860	pyramidal neuron development	Sep-39	0.003025568	0.00882236
GO:0030949	positive regulation of vascular endothelial growth factor receptor signaling pathway	Sep-39	0.003025568	0.00882236
GO:0043455	regulation of secondary metabolic process	Sep-39	0.003025568	0.00882236
GO:0048021	regulation of melanin biosynthetic process	Sep-39	0.003025568	0.00882236
GO:0051798	positive regulation of hair follicle development	Sep-39	0.003025568	0.00882236
GO:0060433	bronchus development	Sep-39	0.003025568	0.00882236

GO:0060525	prostate glandular acinus development	Sep-39	0.003025568	0.00882236
GO:0072176	nephric duct development	Sep-39	0.003025568	0.00882236
GO:0097113	AMPA glutamate receptor clustering	Sep-39	0.003025568	0.00882236
GO:1900376	regulation of secondary metabolite biosynthetic process	Sep-39	0.003025568	0.00882236
GO:1903998	regulation of eating behavior	Sep-39	0.003025568	0.00882236
GO:2000302	positive regulation of synaptic vesicle exocytosis	Sep-39	0.003025568	0.00882236
GO:2001135	regulation of endocytic recycling	Sep-39	0.003025568	0.00882236
GO:0045646	regulation of erythrocyte differentiation	26/7539	0.003039048	0.008857577
GO:0014733	regulation of skeletal muscle adaptation	Nov-39	0.003058638	0.008865555
GO:0030322	stabilization of membrane potential	Nov-39	0.003058638	0.008865555
GO:0031000	response to caffeine	Nov-39	0.003058638	0.008865555
GO:0035590	purinergic nucleotide receptor signaling pathway	Nov-39	0.003058638	0.008865555
GO:0060026	convergent extension	Nov-39	0.003058638	0.008865555
GO:0071731	response to nitric oxide	Nov-39	0.003058638	0.008865555
GO:0072673	lamellipodium morphogenesis	Nov-39	0.003058638	0.008865555
GO:0090136	epithelial cell-cell adhesion	Nov-39	0.003058638	0.008865555
GO:1901077	regulation of relaxation of muscle	Nov-39	0.003058638	0.008865555
GO:1901550	regulation of endothelial cell development	Nov-39	0.003058638	0.008865555

GO:1903140	regulation of establishment of endothelial barrier	Nov-39	0.003058638	0.008865555
GO:2000647	negative regulation of stem cell proliferation	Nov-39	0.003058638	0.008865555
GO:0002224	toll-like receptor signaling pathway	49/7539	0.003067102	0.008881934
GO:0032091	negative regulation of protein binding	49/7539	0.003067102	0.008881934
GO:0032273	positive regulation of protein polymerization	57/7539	0.003101074	0.008958884
GO:0014820	tonic smooth muscle contraction	Oct-39	0.003113542	0.008958884
GO:0019896	axonal transport of mitochondrion	Oct-39	0.003113542	0.008958884
GO:0021936	regulation of cerebellar granule cell precursor proliferation	Oct-39	0.003113542	0.008958884
GO:0032328	alanine transport	Oct-39	0.003113542	0.008958884
GO:0032536	regulation of cell projection size	Oct-39	0.003113542	0.008958884
GO:0034309	primary alcohol biosynthetic process	Oct-39	0.003113542	0.008958884
GO:0035815	positive regulation of renal sodium excretion	Oct-39	0.003113542	0.008958884
GO:0038065	collagen-activated signaling pathway	Oct-39	0.003113542	0.008958884
GO:0048672	positive regulation of collateral sprouting	Oct-39	0.003113542	0.008958884
GO:0061308	cardiac neural crest cell development involved in heart development	Oct-39	0.003113542	0.008958884
GO:0072224	metanephric glomerulus development	Oct-39	0.003113542	0.008958884
GO:0090179	planar cell polarity pathway involved in neural tube closure	Oct-39	0.003113542	0.008958884
GO:1905063	regulation of vascular associated smooth muscle cell differentiation	Oct-39	0.003113542	0.008958884

GO:0050817	coagulation	72/7539	0.003124728	0.00898245
GO:0071356	cellular response to tumor necrosis factor	72/7539	0.003124728	0.00898245
GO:0060444	branching involved in mammary gland duct morphogenesis	17/7539	0.003126001	0.00898245
GO:0030183	B cell differentiation	66/7539	0.003170252	0.009105461
GO:0009636	response to toxic substance	52/7539	0.003191024	0.009160953
GO:0010665	regulation of cardiac muscle cell apoptotic process	28/7539	0.003198566	0.00917426
GO:0030834	regulation of actin filament depolymerization	28/7539	0.003198566	0.00917426
GO:0006821	chloride transport	47/7539	0.003206479	0.009188606
GO:0072676	lymphocyte migration	47/7539	0.003206479	0.009188606
GO:0003341	cilium movement	76/7539	0.003247803	0.009297527
GO:0003298	physiological muscle hypertrophy	23/7539	0.003253325	0.009297527
GO:0003301	physiological cardiac muscle hypertrophy	23/7539	0.003253325	0.009297527
GO:0045880	positive regulation of smoothened signaling pathway	23/7539	0.003253325	0.009297527
GO:0061049	cell growth involved in cardiac muscle cell development	23/7539	0.003253325	0.009297527
GO:1903053	regulation of extracellular matrix organization	23/7539	0.003253325	0.009297527
GO:1900542	regulation of purine nucleotide metabolic process	38/7539	0.003276802	0.009360386
GO:0090303	positive regulation of wound healing	30/7539	0.003298052	0.009404065
GO:1905517	macrophage migration	30/7539	0.003298052	0.009404065

GO:2000401	regulation of lymphocyte migration	30/7539	0.003298052	0.009404065
GO:2001238	positive regulation of extrinsic apoptotic signaling pathway	30/7539	0.003298052	0.009404065
GO:0010573	vascular endothelial growth factor production	20/7539	0.003321985	0.00946376
GO:0034142	toll-like receptor 4 signaling pathway	20/7539	0.003321985	0.00946376
GO:0007260	tyrosine phosphorylation of STAT protein	36/7539	0.003329157	0.009475639
GO:1903725	regulation of phospholipid metabolic process	36/7539	0.003329157	0.009475639
GO:0006305	DNA alkylation	34/7539	0.003354905	0.009524828
GO:0006306	DNA methylation	34/7539	0.003354905	0.009524828
GO:0030888	regulation of B cell proliferation	34/7539	0.003354905	0.009524828
GO:1990868	response to chemokine	34/7539	0.003354905	0.009524828
GO:1990869	cellular response to chemokine	34/7539	0.003354905	0.009524828
GO:0032963	collagen metabolic process	50/7539	0.003355491	0.009524828
GO:0033077	T cell differentiation in thymus	43/7539	0.003472271	0.009847462
GO:0045445	myoblast differentiation	43/7539	0.003472271	0.009847462
GO:0001578	microtubule bundle formation	48/7539	0.003520833	0.0099807

GO terms associated with 1189 Rep_early_depleted_vs_Hep genes

ID	Description	GeneRatio	pvalue	p.adjust
GO:0016054	organic acid catabolic process	79/913	4.26E-61	1.94E-57
GO:0046395	carboxylic acid catabolic process	78/913	3.47E-60	7.91E-57
GO:0044282	small molecule catabolic process	92/913	1.33E-59	2.02E-56
GO:0006631	fatty acid metabolic process	99/913	1.41E-54	1.61E-51
GO:0006520	cellular amino acid metabolic process	69/913	9.32E-44	8.51E-41
GO:1901605	alpha-amino acid metabolic process	59/913	1.16E-42	8.83E-40
GO:0072329	monocarboxylic acid catabolic process	44/913	1.24E-34	8.11E-32
GO:0009063	cellular amino acid catabolic process	38/913	4.44E-32	2.53E-29
GO:0046394	carboxylic acid biosynthetic process	61/913	5.34E-32	2.71E-29
GO:0016053	organic acid biosynthetic process	61/913	6.53E-32	2.88E-29
GO:0016042	lipid catabolic process	64/913	6.95E-32	2.88E-29
GO:0009062	fatty acid catabolic process	37/913	1.31E-29	4.97E-27
GO:0001676	long-chain fatty acid metabolic process	40/913	2.08E-29	7.32E-27
GO:0006790	sulfur compound metabolic process	61/913	2.65E-29	8.63E-27
GO:0044242	cellular lipid catabolic process	51/913	4.89E-29	1.49E-26
GO:1901606	alpha-amino acid catabolic process	33/913	5.38E-29	1.54E-26

GO:0033559	unsaturated fatty acid metabolic process	37/913	1.01E-24	2.70E-22
GO:0008202	steroid metabolic process	56/913	2.20E-24	5.58E-22
GO:0019395	fatty acid oxidation	34/913	4.58E-24	1.10E-21
GO:0120254	olefinic compound metabolic process	39/913	1.89E-23	4.31E-21
GO:0034440	lipid oxidation	34/913	5.54E-23	1.20E-20
GO:0006635	fatty acid beta-oxidation	27/913	1.49E-21	3.09E-19
GO:0019369	arachidonic acid metabolic process	27/913	3.31E-21	6.56E-19
GO:0006690	icosanoid metabolic process	34/913	1.56E-20	2.97E-18
GO:0030258	lipid modification	41/913	1.96E-20	3.58E-18
GO:0033865	nucleoside bisphosphate metabolic process	30/913	5.00E-20	8.15E-18
GO:0033875	ribonucleoside bisphosphate metabolic process	30/913	5.00E-20	8.15E-18
GO:0034032	purine nucleoside bisphosphate metabolic process	30/913	5.00E-20	8.15E-18
GO:0043648	dicarboxylic acid metabolic process	27/913	6.22E-20	9.78E-18
GO:0042180	cellular ketone metabolic process	41/913	5.84E-19	8.88E-17
GO:0006575	cellular modified amino acid metabolic process	37/913	5.88E-18	8.66E-16
GO:0009410	response to xenobiotic stimulus	46/913	1.87E-17	2.66E-15
GO:0006637	acyl-CoA metabolic process	25/913	2.18E-17	2.93E-15
GO:0035383	thioester metabolic process	25/913	2.18E-17	2.93E-15

GO:0072330	monocarboxylic acid biosynthetic process	36/913	9.32E-17	1.21E-14
GO:0006805	xenobiotic metabolic process	28/913	1.95E-16	2.47E-14
GO:0072521	purine-containing compound metabolic process	48/913	1.29E-15	1.59E-13
GO:0071466	cellular response to xenobiotic stimulus	32/913	2.16E-15	2.60E-13
GO:0009117	nucleotide metabolic process	51/913	3.02E-15	3.53E-13
GO:0006753	nucleoside phosphate metabolic process	51/913	6.19E-15	7.07E-13
GO:0009259	ribonucleotide metabolic process	45/913	7.12E-15	7.92E-13
GO:0009150	purine ribonucleotide metabolic process	43/913	2.13E-14	2.32E-12
GO:0019693	ribose phosphate metabolic process	45/913	2.35E-14	2.49E-12
GO:0009074	aromatic amino acid family catabolic process	11/913	1.15E-13	1.19E-11
GO:0006639	acylglycerol metabolic process	27/913	1.46E-13	1.49E-11
GO:0006638	neutral lipid metabolic process	27/913	2.06E-13	2.04E-11
GO:0006163	purine nucleotide metabolic process	43/913	2.25E-13	2.18E-11
GO:0006066	alcohol metabolic process	41/913	4.26E-13	4.05E-11
GO:0009064	glutamine family amino acid metabolic process	18/913	5.95E-13	5.54E-11
GO:0015711	organic anion transport	41/913	9.09E-13	8.30E-11
GO:0042537	benzene-containing compound metabolic process	12/913	1.82E-12	1.63E-10
GO:0008206	bile acid metabolic process	15/913	1.93E-12	1.69E-10

GO:0016125	sterol metabolic process	26/913	3.14E-12	2.70E-10
GO:0051791	medium-chain fatty acid metabolic process	12/913	3.39E-12	2.87E-10
GO:0009072	aromatic amino acid family metabolic process	13/913	3.63E-12	3.01E-10
GO:0006694	steroid biosynthetic process	27/913	4.64E-12	3.78E-10
GO:0008203	cholesterol metabolic process	24/913	3.30E-11	2.64E-09
GO:1902652	secondary alcohol metabolic process	25/913	3.55E-11	2.79E-09
GO:0043651	linoleic acid metabolic process	12/913	4.88E-11	3.75E-09
GO:0006641	triglyceride metabolic process	21/913	4.93E-11	3.75E-09
GO:0046942	carboxylic acid transport	34/913	5.66E-11	4.24E-09
GO:0006820	anion transport	46/913	1.14E-10	8.36E-09
GO:0006957	complement activation, alternative pathway	9/913	1.31E-10	9.46E-09
GO:0006536	glutamate metabolic process	11/913	2.50E-10	1.78E-08
GO:1901617	organic hydroxy compound biosynthetic process	29/913	4.26E-10	2.99E-08
GO:0006749	glutathione metabolic process	17/913	5.41E-10	3.74E-08
GO:0019373	epoxygenase P450 pathway	12/913	5.92E-10	4.03E-08
GO:0006699	bile acid biosynthetic process	11/913	6.25E-10	4.19E-08
GO:0008652	cellular amino acid biosynthetic process	15/913	6.74E-10	4.46E-08
GO:0009069	serine family amino acid metabolic process	12/913	8.51E-10	5.55E-08

GO:0006869	lipid transport	38/913	1.08E-09	6.92E-08
GO:0009636	response to toxic substance	20/913	1.70E-09	1.08E-07
GO:1901607	alpha-amino acid biosynthetic process	14/913	2.02E-09	1.26E-07
GO:0015849	organic acid transport	33/913	3.54E-09	2.19E-07
GO:0010876	lipid localization	40/913	5.26E-09	3.20E-07
GO:0046486	glycerolipid metabolic process	36/913	6.66E-09	4.00E-07
GO:0019216	regulation of lipid metabolic process	36/913	7.14E-09	4.23E-07
GO:0046890	regulation of lipid biosynthetic process	25/913	7.69E-09	4.50E-07
GO:0034754	cellular hormone metabolic process	19/913	1.04E-08	5.98E-07
GO:0042402	cellular biogenic amine catabolic process	9/913	1.06E-08	6.06E-07
GO:0042445	hormone metabolic process	26/913	1.12E-08	6.25E-07
GO:0062012	regulation of small molecule metabolic process	35/913	1.12E-08	6.25E-07
GO:0015850	organic hydroxy compound transport	28/913	1.29E-08	7.11E-07
GO:0035458	cellular response to interferon-beta	13/913	1.60E-08	8.68E-07
GO:0009310	amine catabolic process	9/913	1.70E-08	9.11E-07
GO:0043650	dicarboxylic acid biosynthetic process	7/913	2.16E-08	1.15E-06
GO:1901361	organic cyclic compound catabolic process	40/913	2.83E-08	1.49E-06
GO:0006730	one-carbon metabolic process	11/913	3.04E-08	1.57E-06

GO:0000096	sulfur amino acid metabolic process	10/913	3.33E-08	1.71E-06
GO:1901568	fatty acid derivative metabolic process	11/913	4.03E-08	2.04E-06
GO:0006568	tryptophan metabolic process	7/913	4.55E-08	2.26E-06
GO:0006586	indolalkylamine metabolic process	7/913	4.55E-08	2.26E-06
GO:0006576	cellular biogenic amine metabolic process	17/913	5.21E-08	2.56E-06
GO:0000038	very long-chain fatty acid metabolic process	10/913	8.68E-08	4.12E-06
GO:0035456	response to interferon-beta	13/913	8.78E-08	4.12E-06
GO:0006633	fatty acid biosynthetic process	20/913	8.84E-08	4.12E-06
GO:0009083	branched-chain amino acid catabolic process	8/913	8.85E-08	4.12E-06
GO:0050667	homocysteine metabolic process	7/913	8.86E-08	4.12E-06
GO:0009308	amine metabolic process	18/913	9.87E-08	4.55E-06
GO:0042430	indole-containing compound metabolic process	9/913	1.24E-07	5.67E-06
GO:1901616	organic hydroxy compound catabolic process	13/913	1.55E-07	7.02E-06
GO:0046185	aldehyde catabolic process	7/913	1.61E-07	7.23E-06
GO:0006695	cholesterol biosynthetic process	12/913	1.81E-07	7.96E-06
GO:1902653	secondary alcohol biosynthetic process	12/913	1.81E-07	7.96E-06
GO:0006544	glycine metabolic process	7/913	2.79E-07	1.21E-05
GO:0044106	cellular amine metabolic process	17/913	2.84E-07	1.22E-05

GO:0006766	vitamin metabolic process	14/913	3.77E-07	1.61E-05
GO:0097305	response to alcohol	24/913	3.90E-07	1.65E-05
GO:1905039	carboxylic acid transmembrane transport	18/913	3.99E-07	1.67E-05
GO:0033539	fatty acid beta-oxidation using acyl-CoA dehydrogenase	6/913	4.01E-07	1.67E-05
GO:0015718	monocarboxylic acid transport	17/913	4.07E-07	1.67E-05
GO:0010565	regulation of cellular ketone metabolic process	19/913	4.16E-07	1.69E-05
GO:1903825	organic acid transmembrane transport	18/913	4.45E-07	1.80E-05
GO:0019439	aromatic compound catabolic process	36/913	4.60E-07	1.83E-05
GO:0009081	branched-chain amino acid metabolic process	8/913	4.62E-07	1.83E-05
GO:0046165	alcohol biosynthetic process	18/913	5.52E-07	2.17E-05
GO:0016126	sterol biosynthetic process	12/913	5.75E-07	2.24E-05
GO:0036315	cellular response to sterol	8/913	6.60E-07	2.55E-05
GO:0006956	complement activation	21/913	9.65E-07	3.70E-05
GO:0006525	arginine metabolic process	7/913	1.13E-06	4.30E-05
GO:0044272	sulfur compound biosynthetic process	14/913	1.35E-06	5.10E-05
GO:0006091	generation of precursor metabolites and energy	34/913	1.61E-06	6.02E-05
GO:0046503	glycerolipid catabolic process	12/913	1.87E-06	6.94E-05
GO:0045834	positive regulation of lipid metabolic process	20/913	1.92E-06	7.08E-05

GO:0046459	short-chain fatty acid metabolic process	6/913	2.40E-06	8.78E-05
GO:0002920	regulation of humoral immune response	10/913	2.60E-06	9.42E-05
GO:0055088	lipid homeostasis	19/913	3.52E-06	0.000126612
GO:0042182	ketone catabolic process	6/913	3.90E-06	0.000139037
GO:0015721	bile acid and bile salt transport	8/913	4.07E-06	0.000142748
GO:0036314	response to sterol	8/913	4.07E-06	0.000142748
GO:1990845	adaptive thermogenesis	18/913	5.00E-06	0.000174254
GO:0010875	positive regulation of cholesterol efflux	8/913	5.27E-06	0.000182253
GO:0001562	response to protozoan	9/913	5.39E-06	0.000184972
GO:0006081	cellular aldehyde metabolic process	11/913	5.71E-06	0.000194365
GO:0062013	positive regulation of small molecule metabolic process	18/913	7.02E-06	0.000237427
GO:0006721	terpenoid metabolic process	11/913	7.73E-06	0.000259556
GO:0046461	neutral lipid catabolic process	9/913	8.10E-06	0.000267798
GO:0046464	acylglycerol catabolic process	9/913	8.10E-06	0.000267798
GO:0035337	fatty-acyl-CoA metabolic process	6/913	9.13E-06	0.000297943
GO:0044273	sulfur compound catabolic process	7/913	9.14E-06	0.000297943
GO:0006084	acetyl-CoA metabolic process	8/913	1.08E-05	0.000350945
GO:0035384	thioester biosynthetic process	7/913	1.22E-05	0.00038826

GO:0071616	acyl-CoA biosynthetic process	7/913	1.22E-05	0.00038826
GO:0006558	L-phenylalanine metabolic process	5/913	1.24E-05	0.000391037
GO:1902221	erythrose 4-phosphate/phosphoenolpyruvate family amino acid metabolic process	5/913	1.24E-05	0.000391037
GO:0006959	humoral immune response	31/913	1.25E-05	0.000391037
GO:0090205	positive regulation of cholesterol metabolic process	6/913	1.33E-05	0.000409678
GO:1901570	fatty acid derivative biosynthetic process	6/913	1.33E-05	0.000409678
GO:0033866	nucleoside bisphosphate biosynthetic process	8/913	1.36E-05	0.000409678
GO:0034030	ribonucleoside bisphosphate biosynthetic process	8/913	1.36E-05	0.000409678
GO:0034033	purine nucleoside bisphosphate biosynthetic process	8/913	1.36E-05	0.000409678
GO:0090407	organophosphate biosynthetic process	34/913	1.39E-05	0.000417932
GO:0006720	isoprenoid metabolic process	12/913	1.91E-05	0.000568621
GO:0050873	brown fat cell differentiation	10/913	2.02E-05	0.000599963
GO:0042832	defense response to protozoan	8/913	2.07E-05	0.000609822
GO:0071397	cellular response to cholesterol	6/913	2.64E-05	0.000771191
GO:0006835	dicarboxylic acid transport	12/913	2.70E-05	0.00078463
GO:0046456	icosanoid biosynthetic process	9/913	2.85E-05	0.000823178
GO:0009071	serine family amino acid catabolic process	5/913	3.29E-05	0.000938207
GO:0009084	glutamine family amino acid biosynthetic process	5/913	3.29E-05	0.000938207

GO:0046889	positive regulation of lipid biosynthetic process	13/913	3.46E-05	0.000980715
GO:0090181	regulation of cholesterol metabolic process	8/913	3.72E-05	0.001048121
GO:0006692	prostanoid metabolic process	9/913	4.58E-05	0.001273477
GO:0006693	prostaglandin metabolic process	9/913	4.58E-05	0.001273477
GO:0055006	cardiac cell development	13/913	4.64E-05	0.001283969
GO:0015918	sterol transport	11/913	4.88E-05	0.001333416
GO:0034308	primary alcohol metabolic process	11/913	4.88E-05	0.001333416
GO:0032782	bile acid secretion	5/913	4.98E-05	0.001353651
GO:0006644	phospholipid metabolic process	27/913	5.11E-05	0.001379551
GO:0010874	regulation of cholesterol efflux	8/913	5.34E-05	0.001426739
GO:0050994	regulation of lipid catabolic process	10/913	5.35E-05	0.001426739
GO:0009065	glutamine family amino acid catabolic process	6/913	6.33E-05	0.001666602
GO:0019835	cytolysis	6/913	6.33E-05	0.001666602
GO:0042632	cholesterol homeostasis	12/913	6.35E-05	0.001666602
GO:0009112	nucleobase metabolic process	7/913	6.51E-05	0.001698118
GO:0055092	sterol homeostasis	12/913	7.02E-05	0.001821613
GO:0006570	tyrosine metabolic process	5/913	7.28E-05	0.001855987
GO:0006577	amino-acid betaine metabolic process	5/913	7.28E-05	0.001855987

GO:0015936	coenzyme A metabolic process	5/913	7.28E-05	0.001855987
GO:0045940	positive regulation of steroid metabolic process	8/913	7.50E-05	0.001902825
GO:0106106	cold-induced thermogenesis	15/913	7.61E-05	0.001919271
GO:0019218	regulation of steroid metabolic process	13/913	8.85E-05	0.002218452
GO:0019217	regulation of fatty acid metabolic process	12/913	9.42E-05	0.002348313
GO:0042908	xenobiotic transport	7/913	9.70E-05	0.002405842
GO:0009070	serine family amino acid biosynthetic process	5/913	0.000103119	0.002530301
GO:1902001	fatty acid transmembrane transport	5/913	0.000103119	0.002530301
GO:0008209	androgen metabolic process	6/913	0.000105177	0.002553338
GO:0030449	regulation of complement activation	6/913	0.000105177	0.002553338
GO:0016101	diterpenoid metabolic process	9/913	0.000107378	0.002592978
GO:0072593	reactive oxygen species metabolic process	20/913	0.000117022	0.002810996
GO:0046434	organophosphate catabolic process	15/913	0.000119433	0.002853891
GO:0030301	cholesterol transport	10/913	0.000124863	0.002968096
GO:0070723	response to cholesterol	6/913	0.00013308	0.003147043
GO:0045833	negative regulation of lipid metabolic process	12/913	0.00013669	0.003215732
GO:0007031	peroxisome organization	7/913	0.000140541	0.003272589
GO:0009066	aspartate family amino acid metabolic process	7/913	0.000140541	0.003272589

GO:0006103	2-oxoglutarate metabolic process	5/913	0.000142281	0.003296292
GO:0015740	C4-dicarboxylate transport	6/913	0.000166539	0.003838815
GO:0042060	wound healing	26/913	0.00017387	0.003987646
GO:0033344	cholesterol efflux	9/913	0.000178707	0.004064092
GO:0000050	urea cycle	4/913	0.000180502	0.004064092
GO:0051775	response to redox state	4/913	0.000180502	0.004064092
GO:0019722	calcium-mediated signaling	17/913	0.000180765	0.004064092
GO:0009165	nucleotide biosynthetic process	18/913	0.000184233	0.004121752
GO:0090207	regulation of triglyceride metabolic process	8/913	0.000187507	0.004174543
GO:0019432	triglyceride biosynthetic process	7/913	0.000198719	0.004381425
GO:0033238	regulation of cellular amine metabolic process	7/913	0.000198719	0.004381425
GO:0046460	neutral lipid biosynthetic process	8/913	0.000215508	0.004706118
GO:0046463	acylglycerol biosynthetic process	8/913	0.000215508	0.004706118
GO:0050850	positive regulation of calcium-mediated signaling	7/913	0.00023434	0.005072444
GO:0120161	regulation of cold-induced thermogenesis	14/913	0.000234506	0.005072444
GO:1901293	nucleoside phosphate biosynthetic process	18/913	0.000244735	0.005268728
GO:0007159	leukocyte cell-cell adhesion	26/913	0.00025215	0.005359184
GO:0043691	reverse cholesterol transport	5/913	0.000253634	0.005359184

GO:0044539	long-chain fatty acid import into cell	5/913	0.000253634	0.005359184
GO:1904251	regulation of bile acid metabolic process	5/913	0.000253634	0.005359184
GO:0006107	oxaloacetate metabolic process	4/913	0.000276532	0.005762986
GO:0019627	urea metabolic process	4/913	0.000276532	0.005762986
GO:0071941	nitrogen cycle metabolic process	4/913	0.000276532	0.005762986
GO:0002455	humoral immune response mediated by circulating immunoglobulin	16/913	0.000285879	0.005930682
GO:0001655	urogenital system development	26/913	0.000295986	0.006112572
GO:0097306	cellular response to alcohol	14/913	0.000310302	0.006379358
GO:0045444	fat cell differentiation	20/913	0.000315516	0.00645746
GO:0006089	lactate metabolic process	5/913	0.000329392	0.006651961
GO:0006541	glutamine metabolic process	5/913	0.000329392	0.006651961
GO:0140354	lipid import into cell	5/913	0.000329392	0.006651961
GO:0006958	complement activation, classical pathway	15/913	0.000331712	0.006669317
GO:0001659	temperature homeostasis	16/913	0.000342511	0.006856233
GO:0042558	pteridine-containing compound metabolic process	6/913	0.000371843	0.007410881
GO:0019932	second-messenger-mediated signaling	22/913	0.000384983	0.00763941
GO:0006534	cysteine metabolic process	4/913	0.000404407	0.007887662
GO:0015911	long-chain fatty acid import across plasma membrane	4/913	0.000404407	0.007887662

GO:0034310	primary alcohol catabolic process	4/913	0.000404407	0.007887662
GO:0052695	cellular glucuronidation	4/913	0.000404407	0.007887662
GO:0098754	detoxification	8/913	0.000411726	0.007996244
GO:0008207	C21-steroid hormone metabolic process	7/913	0.000431569	0.008310884
GO:0070232	regulation of T cell apoptotic process	7/913	0.000431569	0.008310884
GO:0010574	regulation of vascular endothelial growth factor production	6/913	0.000445483	0.008542797
GO:1901652	response to peptide	27/913	0.000455759	0.008703279
GO:0001523	retinoid metabolic process	8/913	0.000464508	0.008801396
GO:0055013	cardiac muscle cell development	11/913	0.000464754	0.008801396
GO:0034341	response to interferon-gamma	13/913	0.000492565	0.009289524
GO:0006826	iron ion transport	8/913	0.000522645	0.009805435
GO:0002253	activation of immune response	28/913	0.000524217	0.009805435
GO:0010039	response to iron ion	5/913	0.00053082	0.009808359
GO:0035634	response to stilbenoid	5/913	0.00053082	0.009808359
GO:0071404	cellular response to low-density lipoprotein particle stimulus	5/913	0.00053082	0.009808359
GO:0043434	response to peptide hormone	24/913	0.0005331	0.009810756

GO terms associated with 3140 Rep_intermed_depleted_vs_Hep genes

ID	Description	GeneRatio	pvalue	p.adjust
GO:0044282	small molecule catabolic process	184/2440	9.90E-108	5.63E-104
GO:0006631	fatty acid metabolic process	211/2440	2.09E-106	5.94E-103
GO:0016054	organic acid catabolic process	143/2440	1.72E-97	3.26E-94
GO:0046395	carboxylic acid catabolic process	141/2440	1.11E-95	1.58E-92
GO:0046394	carboxylic acid biosynthetic process	142/2440	8.66E-73	9.85E-70
GO:0016053	organic acid biosynthetic process	142/2440	1.53E-72	1.45E-69
GO:0006520	cellular amino acid metabolic process	132/2440	1.18E-71	9.60E-69
GO:0008202	steroid metabolic process	144/2440	4.59E-65	3.26E-62
GO:0006091	generation of precursor metabolites and energy	161/2440	4.40E-62	2.78E-59
GO:0006790	sulfur compound metabolic process	138/2440	1.51E-61	8.58E-59
GO:1901605	alpha-amino acid metabolic process	101/2440	4.11E-59	2.13E-56
GO:0072521	purine-containing compound metabolic process	143/2440	2.02E-54	9.60E-52
GO:0072329	monocarboxylic acid catabolic process	79/2440	2.94E-54	1.29E-51
GO:0006753	nucleoside phosphate metabolic process	154/2440	1.36E-53	5.54E-51
GO:0016042	lipid catabolic process	129/2440	2.47E-53	9.37E-51
GO:0009117	nucleotide metabolic process	152/2440	3.35E-53	1.19E-50

GO:0072330	monocarboxylic acid biosynthetic process	101/2440	1.89E-52	6.33E-50
GO:0015980	energy derivation by oxidation of organic compounds	123/2440	3.40E-51	1.07E-48
GO:0009259	ribonucleotide metabolic process	133/2440	9.37E-51	2.81E-48
GO:0006066	alcohol metabolic process	129/2440	1.44E-50	3.90E-48
GO:0009150	purine ribonucleotide metabolic process	129/2440	1.44E-50	3.90E-48
GO:0045333	cellular respiration	102/2440	7.30E-50	1.89E-47
GO:0019693	ribose phosphate metabolic process	134/2440	1.14E-49	2.82E-47
GO:0044242	cellular lipid catabolic process	102/2440	1.23E-49	2.92E-47
GO:0006163	purine nucleotide metabolic process	132/2440	3.73E-49	8.48E-47
GO:0009063	cellular amino acid catabolic process	66/2440	1.39E-48	3.05E-46
GO:0009060	aerobic respiration	86/2440	4.83E-48	1.02E-45
GO:0016125	sterol metabolic process	82/2440	8.14E-47	1.65E-44
GO:0009062	fatty acid catabolic process	66/2440	6.53E-46	1.28E-43
GO:1902652	secondary alcohol metabolic process	82/2440	1.13E-45	2.14E-43
GO:0008203	cholesterol metabolic process	78/2440	4.98E-45	9.15E-43
GO:0033865	nucleoside bisphosphate metabolic process	66/2440	1.99E-42	3.34E-40
GO:0033875	ribonucleoside bisphosphate metabolic process	66/2440	1.99E-42	3.34E-40
GO:0034032	purine nucleoside bisphosphate metabolic process	66/2440	1.99E-42	3.34E-40

GO:0006637	acyl-CoA metabolic process	59/2440	3.70E-42	5.85E-40
GO:0035383	thioester metabolic process	59/2440	3.70E-42	5.85E-40
GO:0043648	dicarboxylic acid metabolic process	58/2440	1.09E-41	1.67E-39
GO:0042180	cellular ketone metabolic process	95/2440	1.10E-40	1.64E-38
GO:0046034	ATP metabolic process	99/2440	2.48E-39	3.61E-37
GO:0010876	lipid localization	133/2440	3.66E-39	5.21E-37
GO:0006805	xenobiotic metabolic process	67/2440	5.07E-39	7.04E-37
GO:1901606	alpha-amino acid catabolic process	53/2440	1.80E-38	2.45E-36
GO:0006635	fatty acid beta-oxidation	52/2440	5.22E-38	6.91E-36
GO:0019395	fatty acid oxidation	63/2440	7.14E-38	9.24E-36
GO:0034440	lipid oxidation	63/2440	1.70E-35	2.15E-33
GO:0006869	lipid transport	117/2440	3.77E-35	4.67E-33
GO:0019216	regulation of lipid metabolic process	115/2440	1.00E-34	1.22E-32
GO:0120254	olefinic compound metabolic process	72/2440	1.57E-34	1.86E-32
GO:0071466	cellular response to xenobiotic stimulus	76/2440	1.69E-34	1.97E-32
GO:0006694	steroid biosynthetic process	73/2440	5.54E-34	6.31E-32
GO:0046890	regulation of lipid biosynthetic process	80/2440	1.12E-33	1.26E-31
GO:0006119	oxidative phosphorylation	60/2440	1.17E-33	1.28E-31

GO:0055088	lipid homeostasis	74/2440	1.77E-33	1.91E-31
GO:0006633	fatty acid biosynthetic process	68/2440	1.10E-32	1.16E-30
GO:0033559	unsaturated fatty acid metabolic process	63/2440	2.21E-32	2.29E-30
GO:0062012	regulation of small molecule metabolic process	109/2440	6.24E-32	6.34E-30
GO:0009410	response to xenobiotic stimulus	101/2440	6.53E-32	6.52E-30
GO:0001676	long-chain fatty acid metabolic process	60/2440	1.48E-31	1.45E-29
GO:0030258	lipid modification	79/2440	1.24E-30	1.20E-28
GO:0090407	organophosphate biosynthetic process	125/2440	1.30E-30	1.23E-28
GO:0016126	sterol biosynthetic process	41/2440	6.54E-29	6.11E-27
GO:0006695	cholesterol biosynthetic process	39/2440	7.78E-29	7.03E-27
GO:1902653	secondary alcohol biosynthetic process	39/2440	7.78E-29	7.03E-27
GO:0006641	triglyceride metabolic process	53/2440	1.96E-27	1.74E-25
GO:1901617	organic hydroxy compound biosynthetic process	78/2440	3.61E-27	3.16E-25
GO:0022904	respiratory electron transport chain	48/2440	7.13E-27	6.15E-25
GO:0045834	positive regulation of lipid metabolic process	68/2440	1.05E-26	8.96E-25
GO:0006639	acylglycerol metabolic process	60/2440	1.44E-26	1.21E-24
GO:0022900	electron transport chain	50/2440	2.30E-26	1.90E-24
GO:0010565	regulation of cellular ketone metabolic process	61/2440	2.52E-26	2.05E-24

GO:0006575	cellular modified amino acid metabolic process	71/2440	2.72E-26	2.18E-24
GO:0006638	neutral lipid metabolic process	60/2440	3.43E-26	2.71E-24
GO:0019369	arachidonic acid metabolic process	43/2440	5.19E-26	4.04E-24
GO:0006084	acetyl-CoA metabolic process	28/2440	3.07E-24	2.36E-22
GO:0006690	icosanoid metabolic process	56/2440	5.30E-24	4.02E-22
GO:0046889	positive regulation of lipid biosynthetic process	48/2440	1.06E-23	7.93E-22
GO:0033108	mitochondrial respiratory chain complex assembly	44/2440	5.19E-23	3.84E-21
GO:0015711	organic anion transport	91/2440	2.03E-22	1.48E-20
GO:0042775	mitochondrial ATP synthesis coupled electron transport	37/2440	2.62E-22	1.89E-20
GO:0019646	aerobic electron transport chain	33/2440	2.99E-22	2.12E-20
GO:0042773	ATP synthesis coupled electron transport	37/2440	1.01E-21	7.11E-20
GO:0046165	alcohol biosynthetic process	53/2440	1.20E-21	8.27E-20
GO:0055092	sterol homeostasis	44/2440	1.21E-21	8.27E-20
GO:0042632	cholesterol homeostasis	43/2440	6.44E-21	4.36E-19
GO:0015918	sterol transport	39/2440	6.63E-21	4.44E-19
GO:0046486	glycerolipid metabolic process	92/2440	1.55E-20	1.02E-18
GO:1901293	nucleoside phosphate biosynthetic process	66/2440	2.16E-20	1.41E-18
GO:0030301	cholesterol transport	37/2440	2.28E-20	1.47E-18

GO:0009165	nucleotide biosynthetic process	65/2440	2.66E-20	1.70E-18
GO:0042445	hormone metabolic process	66/2440	3.69E-20	2.34E-18
GO:0019217	regulation of fatty acid metabolic process	43/2440	4.28E-20	2.68E-18
GO:0062013	positive regulation of small molecule metabolic process	56/2440	6.69E-20	4.14E-18
GO:0015850	organic hydroxy compound transport	71/2440	8.47E-20	5.19E-18
GO:0090181	regulation of cholesterol metabolic process	27/2440	1.07E-19	6.45E-18
GO:0009152	purine ribonucleotide biosynthetic process	52/2440	2.71E-19	1.63E-17
GO:0009636	response to toxic substance	46/2440	4.83E-19	2.86E-17
GO:0072522	purine-containing compound biosynthetic process	56/2440	5.82E-19	3.41E-17
GO:0046942	carboxylic acid transport	75/2440	6.77E-19	3.90E-17
GO:0008206	bile acid metabolic process	27/2440	6.78E-19	3.90E-17
GO:0009069	serine family amino acid metabolic process	25/2440	9.45E-19	5.38E-17
GO:0010257	NADH dehydrogenase complex assembly	31/2440	1.30E-18	7.25E-17
GO:0032981	mitochondrial respiratory chain complex I assembly	31/2440	1.30E-18	7.25E-17
GO:0006720	isoprenoid metabolic process	38/2440	1.44E-18	7.93E-17
GO:1905952	regulation of lipid localization	54/2440	1.45E-18	7.93E-17
GO:0015849	organic acid transport	79/2440	2.40E-18	1.30E-16
GO:0034754	cellular hormone metabolic process	45/2440	3.19E-18	1.71E-16

GO:0050994	regulation of lipid catabolic process	33/2440	4.86E-18	2.58E-16
GO:0006164	purine nucleotide biosynthetic process	53/2440	1.02E-17	5.37E-16
GO:0009260	ribonucleotide biosynthetic process	52/2440	1.20E-17	6.29E-16
GO:0006820	anion transport	103/2440	1.23E-17	6.36E-16
GO:0007031	peroxisome organization	24/2440	1.99E-17	1.02E-15
GO:0090207	regulation of triglyceride metabolic process	28/2440	2.33E-17	1.19E-15
GO:0006721	terpenoid metabolic process	32/2440	2.82E-17	1.42E-15
GO:0046390	ribose phosphate biosynthetic process	53/2440	3.22E-17	1.61E-15
GO:0006699	bile acid biosynthetic process	21/2440	6.19E-17	3.06E-15
GO:0042537	benzene-containing compound metabolic process	19/2440	1.12E-16	5.48E-15
GO:0008652	cellular amino acid biosynthetic process	30/2440	1.75E-16	8.52E-15
GO:0006099	tricarboxylic acid cycle	22/2440	1.82E-16	8.79E-15
GO:0006766	vitamin metabolic process	35/2440	1.98E-16	9.45E-15
GO:0006090	pyruvate metabolic process	42/2440	2.66E-16	1.26E-14
GO:0042304	regulation of fatty acid biosynthetic process	26/2440	5.05E-16	2.38E-14
GO:0019218	regulation of steroid metabolic process	41/2440	8.02E-16	3.74E-14
GO:1901361	organic cyclic compound catabolic process	96/2440	9.91E-16	4.59E-14
GO:0098754	detoxification	28/2440	1.08E-15	4.94E-14

GO:0006644	phospholipid metabolic process	80/2440	2.54E-15	1.16E-13
GO:0006749	glutathione metabolic process	33/2440	3.15E-15	1.42E-13
GO:0043691	reverse cholesterol transport	16/2440	5.06E-15	2.27E-13
GO:0015748	organophosphate ester transport	41/2440	8.03E-15	3.57E-13
GO:0006081	cellular aldehyde metabolic process	29/2440	8.32E-15	3.67E-13
GO:0045940	positive regulation of steroid metabolic process	24/2440	1.08E-14	4.73E-13
GO:1901607	alpha-amino acid biosynthetic process	27/2440	1.10E-14	4.80E-13
GO:0009083	branched-chain amino acid catabolic process	16/2440	2.33E-14	1.00E-12
GO:0034976	response to endoplasmic reticulum stress	62/2440	2.81E-14	1.20E-12
GO:0097006	regulation of plasma lipoprotein particle levels	29/2440	3.55E-14	1.51E-12
GO:0033344	cholesterol efflux	28/2440	4.44E-14	1.87E-12
GO:0005996	monosaccharide metabolic process	64/2440	4.54E-14	1.90E-12
GO:0006536	glutamate metabolic process	18/2440	1.05E-13	4.36E-12
GO:0033013	tetrapyrrole metabolic process	26/2440	1.06E-13	4.36E-12
GO:0009081	branched-chain amino acid metabolic process	17/2440	1.09E-13	4.47E-12
GO:0071827	plasma lipoprotein particle organization	22/2440	1.18E-13	4.80E-12
GO:0009072	aromatic amino acid family metabolic process	19/2440	2.07E-13	8.36E-12
GO:1990748	cellular detoxification	22/2440	2.28E-13	9.14E-12

GO:0009064	glutamine family amino acid metabolic process	27/2440	2.33E-13	9.26E-12
GO:0019439	aromatic compound catabolic process	87/2440	2.71E-13	1.07E-11
GO:0046434	organophosphate catabolic process	44/2440	3.01E-13	1.18E-11
GO:0044272	sulfur compound biosynthetic process	33/2440	3.41E-13	1.33E-11
GO:0006778	porphyrin-containing compound metabolic process	22/2440	4.29E-13	1.66E-11
GO:0016101	diterpenoid metabolic process	26/2440	4.70E-13	1.81E-11
GO:0051604	protein maturation	72/2440	5.22E-13	2.00E-11
GO:0019373	epoxygenase P450 pathway	20/2440	6.45E-13	2.38E-11
GO:0033866	nucleoside bisphosphate biosynthetic process	20/2440	6.45E-13	2.38E-11
GO:0034030	ribonucleoside bisphosphate biosynthetic process	20/2440	6.45E-13	2.38E-11
GO:0034033	purine nucleoside bisphosphate biosynthetic process	20/2440	6.45E-13	2.38E-11
GO:0042168	heme metabolic process	20/2440	6.45E-13	2.38E-11
GO:0006120	mitochondrial electron transport, NADH to ubiquinone	18/2440	6.58E-13	2.41E-11
GO:0035384	thioester biosynthetic process	17/2440	8.35E-13	3.03E-11
GO:0071616	acyl-CoA biosynthetic process	17/2440	8.35E-13	3.03E-11
GO:0019318	hexose metabolic process	59/2440	1.14E-12	4.10E-11
GO:0097237	cellular response to toxic substance	23/2440	1.31E-12	4.70E-11
GO:0071825	protein-lipid complex subunit organization	22/2440	1.42E-12	5.06E-11

GO:0090208	positive regulation of triglyceride metabolic process	18/2440	1.51E-12	5.35E-11
GO:0001523	retinoid metabolic process	25/2440	1.53E-12	5.37E-11
GO:0006605	protein targeting	62/2440	1.66E-12	5.79E-11
GO:0090205	positive regulation of cholesterol metabolic process	14/2440	2.08E-12	7.21E-11
GO:0016052	carbohydrate catabolic process	40/2440	3.55E-12	1.22E-10
GO:0019915	lipid storage	31/2440	4.34E-12	1.49E-10
GO:0009145	purine nucleoside triphosphate biosynthetic process	27/2440	4.81E-12	1.63E-10
GO:0046503	glycerolipid catabolic process	27/2440	4.81E-12	1.63E-10
GO:0006544	glycine metabolic process	13/2440	4.90E-12	1.64E-10
GO:0009074	aromatic amino acid family catabolic process	13/2440	4.90E-12	1.64E-10
GO:0072593	reactive oxygen species metabolic process	56/2440	5.11E-12	1.70E-10
GO:0051791	medium-chain fatty acid metabolic process	16/2440	6.50E-12	2.15E-10
GO:0015986	proton motive force-driven ATP synthesis	15/2440	7.62E-12	2.49E-10
GO:0043574	peroxisomal transport	15/2440	7.62E-12	2.49E-10
GO:0050817	coagulation	45/2440	9.11E-12	2.96E-10
GO:0051235	maintenance of location	69/2440	9.45E-12	3.06E-10
GO:1905039	carboxylic acid transmembrane transport	39/2440	9.55E-12	3.07E-10
GO:1903825	organic acid transmembrane transport	39/2440	1.22E-11	3.90E-10

GO:0007596	blood coagulation	44/2440	1.86E-11	5.92E-10
GO:0009206	purine ribonucleoside triphosphate biosynthetic process	26/2440	2.22E-11	7.03E-10
GO:0015718	monocarboxylic acid transport	36/2440	2.53E-11	7.94E-10
GO:0015914	phospholipid transport	30/2440	3.39E-11	1.06E-09
GO:0007599	hemostasis	44/2440	3.46E-11	1.08E-09
GO:0034368	protein-lipid complex remodeling	16/2440	3.52E-11	1.08E-09
GO:0034369	plasma lipoprotein particle remodeling	16/2440	3.52E-11	1.08E-09
GO:0044262	cellular carbohydrate metabolic process	61/2440	3.86E-11	1.18E-09
GO:0034308	primary alcohol metabolic process	28/2440	4.24E-11	1.29E-09
GO:0140053	mitochondrial gene expression	33/2440	4.87E-11	1.47E-09
GO:0010866	regulation of triglyceride biosynthetic process	15/2440	4.96E-11	1.49E-09
GO:0019432	triglyceride biosynthetic process	19/2440	5.21E-11	1.55E-09
GO:0046460	neutral lipid biosynthetic process	22/2440	5.21E-11	1.55E-09
GO:0046463	acylglycerol biosynthetic process	22/2440	5.21E-11	1.55E-09
GO:0000038	very long-chain fatty acid metabolic process	18/2440	5.25E-11	1.55E-09
GO:0035966	response to topologically incorrect protein	38/2440	6.57E-11	1.93E-09
GO:0045923	positive regulation of fatty acid metabolic process	21/2440	6.60E-11	1.93E-09
GO:0009201	ribonucleoside triphosphate biosynthetic process	26/2440	6.79E-11	1.97E-09

GO:0006006	glucose metabolic process	51/2440	7.35E-11	2.12E-09
GO:0042178	xenobiotic catabolic process	16/2440	7.56E-11	2.17E-09
GO:0042157	lipoprotein metabolic process	37/2440	8.58E-11	2.45E-09
GO:0032368	regulation of lipid transport	35/2440	8.81E-11	2.51E-09
GO:0032371	regulation of sterol transport	17/2440	9.21E-11	2.60E-09
GO:0032374	regulation of cholesterol transport	17/2440	9.21E-11	2.60E-09
GO:0006754	ATP biosynthetic process	23/2440	9.34E-11	2.61E-09
GO:0071806	protein transmembrane transport	23/2440	9.34E-11	2.61E-09
GO:0009071	serine family amino acid catabolic process	Nov-40	1.04E-10	2.89E-09
GO:0034375	high-density lipoprotein particle remodeling	Nov-40	1.04E-10	2.89E-09
GO:0009144	purine nucleoside triphosphate metabolic process	30/2440	1.14E-10	3.13E-09
GO:0065002	intracellular protein transmembrane transport	22/2440	1.27E-10	3.48E-09
GO:0008654	phospholipid biosynthetic process	50/2440	1.43E-10	3.90E-09
GO:0034367	protein-containing complex remodeling	16/2440	1.55E-10	4.19E-09
GO:0042572	retinol metabolic process	16/2440	1.55E-10	4.19E-09
GO:1990542	mitochondrial transmembrane transport	27/2440	1.76E-10	4.71E-09
GO:0009308	amine metabolic process	35/2440	1.83E-10	4.90E-09
GO:0006085	acetyl-CoA biosynthetic process	13/2440	1.86E-10	4.93E-09

GO:0042730	fibrinolysis	13/2440	1.86E-10	4.93E-09
GO:0006107	oxaloacetate metabolic process	Oct-40	1.89E-10	4.97E-09
GO:0033539	fatty acid beta-oxidation using acyl-CoA dehydrogenase	Oct-40	1.89E-10	4.97E-09
GO:0070972	protein localization to endoplasmic reticulum	26/2440	1.94E-10	5.06E-09
GO:0006783	heme biosynthetic process	15/2440	2.49E-10	6.46E-09
GO:0009205	purine ribonucleoside triphosphate metabolic process	28/2440	2.87E-10	7.42E-09
GO:0009116	nucleoside metabolic process	22/2440	2.96E-10	7.62E-09
GO:0046470	phosphatidylcholine metabolic process	23/2440	3.10E-10	7.93E-09
GO:0043467	regulation of generation of precursor metabolites and energy	38/2440	3.11E-10	7.93E-09
GO:0009142	nucleoside triphosphate biosynthetic process	27/2440	3.32E-10	8.44E-09
GO:0031998	regulation of fatty acid beta-oxidation	14/2440	3.69E-10	9.33E-09
GO:1901657	glycosyl compound metabolic process	26/2440	3.76E-10	9.47E-09
GO:1901616	organic hydroxy compound catabolic process	24/2440	4.42E-10	1.11E-08
GO:0050810	regulation of steroid biosynthetic process	27/2440	4.53E-10	1.13E-08
GO:0045833	negative regulation of lipid metabolic process	31/2440	4.87E-10	1.21E-08
GO:0045540	regulation of cholesterol biosynthetic process	13/2440	4.90E-10	1.21E-08
GO:0106118	regulation of sterol biosynthetic process	13/2440	4.90E-10	1.21E-08
GO:0045723	positive regulation of fatty acid biosynthetic process	15/2440	5.15E-10	1.26E-08

GO:0030195	negative regulation of blood coagulation	20/2440	5.45E-10	1.33E-08
GO:0006103	2-oxoglutarate metabolic process	Dec-40	5.48E-10	1.33E-08
GO:0042776	proton motive force-driven mitochondrial ATP synthesis	Dec-40	5.48E-10	1.33E-08
GO:0042908	xenobiotic transport	17/2440	5.77E-10	1.39E-08
GO:0006779	porphyrin-containing compound biosynthetic process	16/2440	5.84E-10	1.40E-08
GO:0033014	tetrapyrrole biosynthetic process	16/2440	5.84E-10	1.40E-08
GO:0046364	monosaccharide biosynthetic process	28/2440	6.93E-10	1.65E-08
GO:0046461	neutral lipid catabolic process	19/2440	7.04E-10	1.66E-08
GO:0046464	acylglycerol catabolic process	19/2440	7.04E-10	1.66E-08
GO:0006576	cellular biogenic amine metabolic process	31/2440	8.13E-10	1.91E-08
GO:1900047	negative regulation of hemostasis	20/2440	8.47E-10	1.99E-08
GO:0016051	carbohydrate biosynthetic process	45/2440	8.77E-10	2.04E-08
GO:0016485	protein processing	56/2440	8.80E-10	2.04E-08
GO:0009199	ribonucleoside triphosphate metabolic process	28/2440	9.20E-10	2.13E-08
GO:0010883	regulation of lipid storage	21/2440	9.35E-10	2.15E-08
GO:0044106	cellular amine metabolic process	33/2440	1.01E-09	2.32E-08
GO:0000096	sulfur amino acid metabolic process	16/2440	1.08E-09	2.45E-08
GO:0010875	positive regulation of cholesterol efflux	16/2440	1.08E-09	2.45E-08

GO:1901292	nucleoside phosphate catabolic process	25/2440	1.11E-09	2.52E-08
GO:1901136	carbohydrate derivative catabolic process	37/2440	1.19E-09	2.68E-08
GO:0050819	negative regulation of coagulation	20/2440	1.30E-09	2.92E-08
GO:0042060	wound healing	68/2440	1.47E-09	3.29E-08
GO:0006625	protein targeting to peroxisome	Dec-40	1.52E-09	3.36E-08
GO:0072662	protein localization to peroxisome	Dec-40	1.52E-09	3.36E-08
GO:0072663	establishment of protein localization to peroxisome	Dec-40	1.52E-09	3.36E-08
GO:0006957	complement activation, alternative pathway	Nov-40	1.56E-09	3.42E-08
GO:0046185	aldehyde catabolic process	Nov-40	1.56E-09	3.42E-08
GO:0044273	sulfur compound catabolic process	14/2440	1.71E-09	3.74E-08
GO:0009066	aspartate family amino acid metabolic process	17/2440	1.71E-09	3.74E-08
GO:0050818	regulation of coagulation	24/2440	1.72E-09	3.75E-08
GO:0009112	nucleobase metabolic process	16/2440	1.92E-09	4.15E-08
GO:0050996	positive regulation of lipid catabolic process	16/2440	1.92E-09	4.15E-08
GO:0006986	response to unfolded protein	32/2440	2.10E-09	4.51E-08
GO:0030193	regulation of blood coagulation	23/2440	2.66E-09	5.66E-08
GO:0042440	pigment metabolic process	23/2440	2.66E-09	5.66E-08
GO:0009164	nucleoside catabolic process	13/2440	2.67E-09	5.66E-08

GO:0034377	plasma lipoprotein particle assembly	13/2440	2.67E-09	5.66E-08
GO:0034381	plasma lipoprotein particle clearance	17/2440	2.86E-09	5.98E-08
GO:0055090	acylglycerol homeostasis	17/2440	2.86E-09	5.98E-08
GO:0070328	triglyceride homeostasis	17/2440	2.86E-09	5.98E-08
GO:1905953	negative regulation of lipid localization	20/2440	2.94E-09	6.14E-08
GO:0034656	nucleobase-containing small molecule catabolic process	14/2440	3.41E-09	7.09E-08
GO:0009991	response to extracellular stimulus	71/2440	3.60E-09	7.46E-08
GO:0015919	peroxisomal membrane transport	Dec-40	3.79E-09	7.82E-08
GO:0009141	nucleoside triphosphate metabolic process	31/2440	4.37E-09	8.98E-08
GO:0006767	water-soluble vitamin metabolic process	17/2440	4.67E-09	9.55E-08
GO:0006979	response to oxidative stress	71/2440	4.96E-09	1.01E-07
GO:0006879	cellular iron ion homeostasis	23/2440	5.10E-09	1.03E-07
GO:1900046	regulation of hemostasis	23/2440	5.10E-09	1.03E-07
GO:0008299	isoprenoid biosynthetic process	16/2440	5.68E-09	1.15E-07
GO:0009162	deoxyribonucleoside monophosphate metabolic process	15/2440	6.42E-09	1.29E-07
GO:0015721	bile acid and bile salt transport	15/2440	6.42E-09	1.29E-07
GO:1905954	positive regulation of lipid localization	28/2440	7.57E-09	1.51E-07
GO:0009166	nucleotide catabolic process	22/2440	7.93E-09	1.58E-07

GO:0010038	response to metal ion	56/2440	8.34E-09	1.65E-07
GO:0006144	purine nucleobase metabolic process	Dec-40	8.75E-09	1.72E-07
GO:0009159	deoxyribonucleoside monophosphate catabolic process	Dec-40	8.75E-09	1.72E-07
GO:0031638	zymogen activation	28/2440	9.67E-09	1.89E-07
GO:0055072	iron ion homeostasis	27/2440	9.68E-09	1.89E-07
GO:0034384	high-density lipoprotein particle clearance	Sep-40	1.04E-08	2.02E-07
GO:0019433	triglyceride catabolic process	15/2440	1.11E-08	2.16E-07
GO:0046320	regulation of fatty acid oxidation	17/2440	1.18E-08	2.27E-07
GO:1901568	fatty acid derivative metabolic process	17/2440	1.18E-08	2.27E-07
GO:0072594	establishment of protein localization to organelle	72/2440	1.23E-08	2.36E-07
GO:0050878	regulation of body fluid levels	65/2440	1.87E-08	3.58E-07
GO:0046164	alcohol catabolic process	18/2440	1.96E-08	3.75E-07
GO:0051346	negative regulation of hydrolase activity	64/2440	2.10E-08	3.98E-07
GO:0045861	negative regulation of proteolysis	63/2440	2.11E-08	3.98E-07
GO:0052548	regulation of endopeptidase activity	63/2440	2.11E-08	3.98E-07
GO:0009125	nucleoside monophosphate catabolic process	13/2440	2.19E-08	4.12E-07
GO:0030449	regulation of complement activation	13/2440	2.19E-08	4.12E-07
GO:0043434	response to peptide hormone	63/2440	2.35E-08	4.40E-07

GO:1901661	quinone metabolic process	16/2440	2.43E-08	4.54E-07
GO:0045017	glycerolipid biosynthetic process	44/2440	2.48E-08	4.61E-07
GO:0062014	negative regulation of small molecule metabolic process	27/2440	2.58E-08	4.77E-07
GO:0010874	regulation of cholesterol efflux	17/2440	2.77E-08	5.12E-07
GO:0010867	positive regulation of triglyceride biosynthetic process	Nov-40	2.85E-08	5.26E-07
GO:0009119	ribonucleoside metabolic process	15/2440	3.10E-08	5.70E-07
GO:0046031	ADP metabolic process	27/2440	3.26E-08	5.96E-07
GO:0006826	iron ion transport	20/2440	3.66E-08	6.67E-07
GO:0046700	heterocycle catabolic process	71/2440	3.70E-08	6.73E-07
GO:0043651	linoleic acid metabolic process	14/2440	3.71E-08	6.73E-07
GO:0010039	response to iron ion	Dec-40	3.82E-08	6.91E-07
GO:0043650	dicarboxylic acid biosynthetic process	Sep-40	3.84E-08	6.91E-07
GO:0065005	protein-lipid complex assembly	13/2440	4.04E-08	7.26E-07
GO:0002920	regulation of humoral immune response	18/2440	4.21E-08	7.51E-07
GO:0009262	deoxyribonucleotide metabolic process	18/2440	4.21E-08	7.51E-07
GO:0007006	mitochondrial membrane organization	28/2440	4.84E-08	8.59E-07
GO:0036503	ERAD pathway	28/2440	4.84E-08	8.59E-07
GO:0042278	purine nucleoside metabolic process	15/2440	5.00E-08	8.84E-07

GO:0008637	apoptotic mitochondrial changes	30/2440	5.02E-08	8.85E-07
GO:0019319	hexose biosynthetic process	24/2440	5.49E-08	9.64E-07
GO:0006730	one-carbon metabolic process	16/2440	5.83E-08	1.02E-06
GO:0006739	NADP metabolic process	16/2440	5.83E-08	1.02E-06
GO:0009261	ribonucleotide catabolic process	17/2440	6.16E-08	1.07E-06
GO:0045922	negative regulation of fatty acid metabolic process	14/2440	6.24E-08	1.08E-06
GO:0016114	terpenoid biosynthetic process	Nov-40	6.25E-08	1.08E-06
GO:0052547	regulation of peptidase activity	72/2440	6.29E-08	1.08E-06
GO:0006096	glycolytic process	25/2440	7.01E-08	1.21E-06
GO:0009264	deoxyribonucleotide catabolic process	13/2440	7.19E-08	1.23E-06
GO:0031639	plasminogen activation	13/2440	7.19E-08	1.23E-06
GO:0072525	pyridine-containing compound biosynthetic process	Dec-40	7.37E-08	1.26E-06
GO:0044270	cellular nitrogen compound catabolic process	70/2440	7.43E-08	1.26E-06
GO:0035967	cellular response to topologically incorrect protein	28/2440	7.44E-08	1.26E-06
GO:1901652	response to peptide	69/2440	7.69E-08	1.30E-06
GO:0072524	pyridine-containing compound metabolic process	15/2440	7.91E-08	1.33E-06
GO:0009123	nucleoside monophosphate metabolic process	21/2440	7.92E-08	1.33E-06
GO:0009135	purine nucleoside diphosphate metabolic process	27/2440	8.03E-08	1.34E-06

GO:0009179	purine ribonucleoside diphosphate metabolic process	27/2440	8.03E-08	1.34E-06
GO:0006757	ATP generation from ADP	25/2440	8.89E-08	1.48E-06
GO:0072523	purine-containing compound catabolic process	17/2440	9.00E-08	1.49E-06
GO:0009070	serine family amino acid biosynthetic process	Oct-40	9.26E-08	1.52E-06
GO:0055091	phospholipid homeostasis	Oct-40	9.26E-08	1.52E-06
GO:0072350	tricarboxylic acid metabolic process	Oct-40	9.26E-08	1.52E-06
GO:0009132	nucleoside diphosphate metabolic process	31/2440	9.74E-08	1.60E-06
GO:0006165	nucleoside diphosphate phosphorylation	27/2440	9.98E-08	1.63E-06
GO:0031667	response to nutrient levels	63/2440	1.00E-07	1.63E-06
GO:0000050	urea cycle	Aug-40	1.01E-07	1.63E-06
GO:0110095	cellular detoxification of aldehyde	Aug-40	1.01E-07	1.63E-06
GO:0006888	endoplasmic reticulum to Golgi vesicle-mediated transport	29/2440	1.02E-07	1.65E-06
GO:0006063	uronic acid metabolic process	Sep-40	1.15E-07	1.84E-06
GO:0009120	deoxyribonucleoside metabolic process	Sep-40	1.15E-07	1.84E-06
GO:0019585	glucuronate metabolic process	Sep-40	1.15E-07	1.84E-06
GO:0070508	cholesterol import	Sep-40	1.15E-07	1.84E-06
GO:0098656	anion transmembrane transport	41/2440	1.22E-07	1.94E-06
GO:0055076	transition metal ion homeostasis	32/2440	1.22E-07	1.94E-06

GO:0042573	retinoic acid metabolic process	13/2440	1.24E-07	1.96E-06
GO:0046128	purine ribonucleoside metabolic process	13/2440	1.24E-07	1.96E-06
GO:0009394	2'-deoxyribonucleotide metabolic process	17/2440	1.30E-07	2.04E-06
GO:0019692	deoxyribose phosphate metabolic process	17/2440	1.30E-07	2.04E-06
GO:0032527	protein exit from endoplasmic reticulum	16/2440	1.31E-07	2.06E-06
GO:0010878	cholesterol storage	Dec-40	1.36E-07	2.13E-06
GO:0010906	regulation of glucose metabolic process	31/2440	1.41E-07	2.20E-06
GO:0006839	mitochondrial transport	34/2440	1.50E-07	2.33E-06
GO:0046939	nucleotide phosphorylation	27/2440	1.88E-07	2.91E-06
GO:0032543	mitochondrial translation	22/2440	1.90E-07	2.94E-06
GO:0010896	regulation of triglyceride catabolic process	Oct-40	2.08E-07	3.19E-06
GO:0046415	urate metabolic process	Oct-40	2.08E-07	3.19E-06
GO:0045047	protein targeting to ER	16/2440	2.79E-07	4.28E-06
GO:1990845	adaptive thermogenesis	35/2440	2.82E-07	4.31E-06
GO:0032782	bile acid secretion	Sep-40	2.98E-07	4.54E-06
GO:0050667	homocysteine metabolic process	Sep-40	2.98E-07	4.54E-06
GO:0010823	negative regulation of mitochondrion organization	18/2440	3.20E-07	4.86E-06
GO:0010675	regulation of cellular carbohydrate metabolic process	35/2440	3.29E-07	4.98E-06

GO:0010951	negative regulation of endopeptidase activity	38/2440	3.35E-07	5.04E-06
GO:1901658	glycosyl compound catabolic process	13/2440	3.37E-07	5.04E-06
GO:0006122	mitochondrial electron transport, ubiquinol to cytochrome c	Aug-40	3.42E-07	5.04E-06
GO:0006558	L-phenylalanine metabolic process	Aug-40	3.42E-07	5.04E-06
GO:0009812	flavonoid metabolic process	Aug-40	3.42E-07	5.04E-06
GO:0019627	urea metabolic process	Aug-40	3.42E-07	5.04E-06
GO:0045542	positive regulation of cholesterol biosynthetic process	Aug-40	3.42E-07	5.04E-06
GO:0071941	nitrogen cycle metabolic process	Aug-40	3.42E-07	5.04E-06
GO:0106120	positive regulation of sterol biosynthetic process	Aug-40	3.42E-07	5.04E-06
GO:1902221	erythrose 4-phosphate/phosphoenolpyruvate family amino acid metabolic process	Aug-40	3.42E-07	5.04E-06
GO:0051259	protein complex oligomerization	46/2440	3.47E-07	5.10E-06
GO:0032102	negative regulation of response to external stimulus	65/2440	3.58E-07	5.25E-06
GO:0010888	negative regulation of lipid storage	Dec-40	4.14E-07	6.04E-06
GO:0046386	deoxyribose phosphate catabolic process	Dec-40	4.14E-07	6.04E-06
GO:0046916	cellular transition metal ion homeostasis	27/2440	4.17E-07	6.07E-06
GO:0051004	regulation of lipoprotein lipase activity	Oct-40	4.32E-07	6.25E-06
GO:0072378	blood coagulation, fibrin clot formation	Oct-40	4.32E-07	6.25E-06
GO:0009158	ribonucleoside monophosphate catabolic process	Nov-40	4.59E-07	6.62E-06

GO:0010885	regulation of cholesterol storage	Nov-40	4.59E-07	6.62E-06
GO:2000377	regulation of reactive oxygen species metabolic process	34/2440	4.61E-07	6.62E-06
GO:0051260	protein homooligomerization	37/2440	4.71E-07	6.75E-06
GO:0051289	protein homotetramerization	17/2440	5.04E-07	7.21E-06
GO:0006094	gluconeogenesis	22/2440	5.09E-07	7.26E-06
GO:0036314	response to sterol	13/2440	5.35E-07	7.58E-06
GO:0042558	pteridine-containing compound metabolic process	13/2440	5.35E-07	7.58E-06
GO:0045454	cell redox homeostasis	13/2440	5.35E-07	7.58E-06
GO:0006109	regulation of carbohydrate metabolic process	39/2440	5.48E-07	7.74E-06
GO:0019748	secondary metabolic process	18/2440	5.85E-07	8.24E-06
GO:0006953	acute-phase response	15/2440	5.99E-07	8.42E-06
GO:0106106	cold-induced thermogenesis	32/2440	6.60E-07	9.25E-06
GO:0006570	tyrosine metabolic process	Sep-40	6.89E-07	9.55E-06
GO:0010887	negative regulation of cholesterol storage	Sep-40	6.89E-07	9.55E-06
GO:0033700	phospholipid efflux	Sep-40	6.89E-07	9.55E-06
GO:0110096	cellular response to aldehyde	Sep-40	6.89E-07	9.55E-06
GO:0046148	pigment biosynthetic process	17/2440	6.90E-07	9.55E-06
GO:0009185	ribonucleoside diphosphate metabolic process	27/2440	7.37E-07	1.02E-05

GO:0097305	response to alcohol	43/2440	7.46E-07	1.03E-05
GO:0006956	complement activation	38/2440	7.70E-07	1.06E-05
GO:0051055	negative regulation of lipid biosynthetic process	18/2440	7.81E-07	1.07E-05
GO:0009065	glutamine family amino acid catabolic process	Nov-40	8.12E-07	1.11E-05
GO:0007007	inner mitochondrial membrane organization	13/2440	8.31E-07	1.13E-05
GO:0006525	arginine metabolic process	Oct-40	8.42E-07	1.14E-05
GO:0006613	cotranslational protein targeting to membrane	Oct-40	8.42E-07	1.14E-05
GO:0019363	pyridine nucleotide biosynthetic process	Oct-40	8.42E-07	1.14E-05
GO:1904251	regulation of bile acid metabolic process	Oct-40	8.42E-07	1.14E-05
GO:0007030	Golgi organization	30/2440	9.33E-07	1.26E-05
GO:0034620	cellular response to unfolded protein	23/2440	9.43E-07	1.26E-05
GO:0006086	acetyl-CoA biosynthetic process from pyruvate	Aug-40	9.48E-07	1.26E-05
GO:0006152	purine nucleoside catabolic process	Aug-40	9.48E-07	1.26E-05
GO:0009435	NAD biosynthetic process	Aug-40	9.48E-07	1.26E-05
GO:0010898	positive regulation of triglyceride catabolic process	Aug-40	9.48E-07	1.26E-05
GO:0052695	cellular glucuronidation	Aug-40	9.48E-07	1.26E-05
GO:0071281	cellular response to iron ion	Aug-40	9.48E-07	1.26E-05
GO:0051262	protein tetramerization	22/2440	1.01E-06	1.34E-05

GO:0010821	regulation of mitochondrion organization	31/2440	1.08E-06	1.43E-05
GO:0072599	establishment of protein localization to endoplasmic reticulum	16/2440	1.09E-06	1.43E-05
GO:0042430	indole-containing compound metabolic process	Dec-40	1.11E-06	1.46E-05
GO:0006835	dicarboxylic acid transport	23/2440	1.17E-06	1.53E-05
GO:0050892	intestinal absorption	15/2440	1.22E-06	1.59E-05
GO:0006692	prostanoid metabolic process	17/2440	1.26E-06	1.64E-05
GO:0006693	prostaglandin metabolic process	17/2440	1.26E-06	1.64E-05
GO:0006457	protein folding	34/2440	1.30E-06	1.69E-05
GO:0044042	glucan metabolic process	21/2440	1.34E-06	1.74E-05
GO:0072376	protein activation cascade	Nov-40	1.38E-06	1.79E-05
GO:0015909	long-chain fatty acid transport	19/2440	1.40E-06	1.81E-05
GO:0019359	nicotinamide nucleotide biosynthetic process	Sep-40	1.46E-06	1.85E-05
GO:0034433	steroid esterification	Sep-40	1.46E-06	1.85E-05
GO:0034434	sterol esterification	Sep-40	1.46E-06	1.85E-05
GO:0034435	cholesterol esterification	Sep-40	1.46E-06	1.85E-05
GO:0042454	ribonucleoside catabolic process	Sep-40	1.46E-06	1.85E-05
GO:0045717	negative regulation of fatty acid biosynthetic process	Sep-40	1.46E-06	1.85E-05
GO:0070857	regulation of bile acid biosynthetic process	Sep-40	1.46E-06	1.85E-05

GO:0120161	regulation of cold-induced thermogenesis	31/2440	1.48E-06	1.87E-05
GO:0006650	glycerophospholipid metabolic process	48/2440	1.52E-06	1.92E-05
GO:0009110	vitamin biosynthetic process	Oct-40	1.55E-06	1.96E-05
GO:0015908	fatty acid transport	24/2440	1.58E-06	1.99E-05
GO:2000378	negative regulation of reactive oxygen species metabolic process	17/2440	1.68E-06	2.11E-05
GO:0010466	negative regulation of peptidase activity	46/2440	1.71E-06	2.15E-05
GO:0008053	mitochondrial fusion	Dec-40	1.75E-06	2.18E-05
GO:0010893	positive regulation of steroid biosynthetic process	Dec-40	1.75E-06	2.18E-05
GO:1903008	organelle disassembly	26/2440	1.76E-06	2.19E-05
GO:0061045	negative regulation of wound healing	20/2440	1.77E-06	2.20E-05
GO:0018958	phenol-containing compound metabolic process	28/2440	1.82E-06	2.25E-05
GO:0006568	tryptophan metabolic process	Aug-40	2.28E-06	2.79E-05
GO:0006586	indolalkylamine metabolic process	Aug-40	2.28E-06	2.79E-05
GO:0010872	regulation of cholesterol esterification	Aug-40	2.28E-06	2.79E-05
GO:0043649	dicarboxylic acid catabolic process	Aug-40	2.28E-06	2.79E-05
GO:0008209	androgen metabolic process	Nov-40	2.28E-06	2.79E-05
GO:0018904	ether metabolic process	Nov-40	2.28E-06	2.79E-05
GO:0036315	cellular response to sterol	Nov-40	2.28E-06	2.79E-05

GO:0006195	purine nucleotide catabolic process	14/2440	2.63E-06	3.20E-05
GO:0006959	humoral immune response	64/2440	2.71E-06	3.30E-05
GO:0044743	protein transmembrane import into intracellular organelle	13/2440	2.77E-06	3.36E-05
GO:0035337	fatty-acyl-CoA metabolic process	Sep-40	2.86E-06	3.46E-05
GO:0042407	cristae formation	Sep-40	2.86E-06	3.46E-05
GO:0001659	temperature homeostasis	36/2440	2.92E-06	3.52E-05
GO:0010873	positive regulation of cholesterol esterification	Jul-40	2.95E-06	3.52E-05
GO:0034372	very-low-density lipoprotein particle remodeling	Jul-40	2.95E-06	3.52E-05
GO:0034380	high-density lipoprotein particle assembly	Jul-40	2.95E-06	3.52E-05
GO:0046055	dGMP catabolic process	Jul-40	2.95E-06	3.52E-05
GO:0046130	purine ribonucleoside catabolic process	Jul-40	2.95E-06	3.52E-05
GO:0048193	Golgi vesicle transport	47/2440	3.18E-06	3.79E-05
GO:0051341	regulation of oxidoreductase activity	23/2440	3.19E-06	3.79E-05
GO:0032370	positive regulation of lipid transport	20/2440	3.50E-06	4.15E-05
GO:0030970	retrograde protein transport, ER to cytosol	Nov-40	3.65E-06	4.31E-05
GO:0070723	response to cholesterol	Nov-40	3.65E-06	4.31E-05
GO:1903513	endoplasmic reticulum to cytosol transport	Nov-40	3.65E-06	4.31E-05
GO:0016236	macroautophagy	43/2440	3.71E-06	4.36E-05

GO:0031668	cellular response to extracellular stimulus	42/2440	3.72E-06	4.36E-05
GO:0009395	phospholipid catabolic process	17/2440	3.80E-06	4.44E-05
GO:0042743	hydrogen peroxide metabolic process	17/2440	3.80E-06	4.44E-05
GO:0009154	purine ribonucleotide catabolic process	13/2440	4.00E-06	4.67E-05
GO:0005977	glycogen metabolic process	20/2440	4.36E-06	5.06E-05
GO:0006073	cellular glucan metabolic process	20/2440	4.36E-06	5.06E-05
GO:0035634	response to stilbenoid	Oct-40	4.64E-06	5.37E-05
GO:0042402	cellular biogenic amine catabolic process	Oct-40	4.64E-06	5.37E-05
GO:1901264	carbohydrate derivative transport	18/2440	4.87E-06	5.62E-05
GO:0030433	ubiquitin-dependent ERAD pathway	21/2440	4.90E-06	5.64E-05
GO:0010635	regulation of mitochondrial fusion	Aug-40	4.93E-06	5.64E-05
GO:0034756	regulation of iron ion transport	Aug-40	4.93E-06	5.64E-05
GO:0046459	short-chain fatty acid metabolic process	Aug-40	4.93E-06	5.64E-05
GO:1903725	regulation of phospholipid metabolic process	14/2440	5.09E-06	5.82E-05
GO:0002921	negative regulation of humoral immune response	Sep-40	5.29E-06	6.02E-05
GO:0006614	SRP-dependent cotranslational protein targeting to membrane	Sep-40	5.29E-06	6.02E-05
GO:0006706	steroid catabolic process	Nov-40	5.69E-06	6.43E-05
GO:0019362	pyridine nucleotide metabolic process	Nov-40	5.69E-06	6.43E-05

GO:0006775	fat-soluble vitamin metabolic process	13/2440	5.69E-06	6.43E-05
GO:0071402	cellular response to lipoprotein particle stimulus	13/2440	5.69E-06	6.43E-05
GO:0042593	glucose homeostasis	48/2440	6.19E-06	6.98E-05
GO:0009161	ribonucleoside monophosphate metabolic process	16/2440	6.24E-06	7.02E-05
GO:1903035	negative regulation of response to wounding	22/2440	6.43E-06	7.22E-05
GO:0033500	carbohydrate homeostasis	48/2440	6.81E-06	7.63E-05
GO:0009746	response to hexose	36/2440	6.95E-06	7.77E-05
GO:0009172	purine deoxyribonucleoside monophosphate catabolic process	Jul-40	7.52E-06	8.35E-05
GO:0030300	regulation of intestinal cholesterol absorption	Jul-40	7.52E-06	8.35E-05
GO:0034370	triglyceride-rich lipoprotein particle remodeling	Jul-40	7.52E-06	8.35E-05
GO:0051006	positive regulation of lipoprotein lipase activity	Jul-40	7.52E-06	8.35E-05
GO:0009151	purine deoxyribonucleotide metabolic process	Oct-40	7.59E-06	8.38E-05
GO:0009310	amine catabolic process	Oct-40	7.59E-06	8.38E-05
GO:0019400	alditol metabolic process	Oct-40	7.59E-06	8.38E-05
GO:0009743	response to carbohydrate	38/2440	8.03E-06	8.84E-05
GO:0050873	brown fat cell differentiation	17/2440	8.08E-06	8.88E-05
GO:0050995	negative regulation of lipid catabolic process	Nov-40	8.66E-06	9.50E-05
GO:1904478	regulation of intestinal absorption	Sep-40	9.29E-06	0.000101693

GO:0030968	endoplasmic reticulum unfolded protein response	18/2440	9.73E-06	0.000105916
GO:0009170	purine deoxyribonucleoside monophosphate metabolic process	Aug-40	9.77E-06	0.000105916
GO:0032373	positive regulation of sterol transport	Aug-40	9.77E-06	0.000105916
GO:0032376	positive regulation of cholesterol transport	Aug-40	9.77E-06	0.000105916
GO:0046085	adenosine metabolic process	Aug-40	9.77E-06	0.000105916
GO:0034284	response to monosaccharide	36/2440	9.90E-06	0.000106995
GO:0001818	negative regulation of cytokine production	48/2440	9.91E-06	0.000106995
GO:1902930	regulation of alcohol biosynthetic process	15/2440	1.02E-05	0.000110207
GO:0006612	protein targeting to membrane	26/2440	1.09E-05	0.000117604
GO:0051180	vitamin transport	13/2440	1.11E-05	0.000118685
GO:1905897	regulation of response to endoplasmic reticulum stress	19/2440	1.11E-05	0.000119125
GO:0019751	polyol metabolic process	25/2440	1.12E-05	0.000120196
GO:0042158	lipoprotein biosynthetic process	23/2440	1.14E-05	0.000121267
GO:0070585	protein localization to mitochondrion	23/2440	1.14E-05	0.000121267
GO:0000041	transition metal ion transport	24/2440	1.14E-05	0.000121267
GO:0009895	negative regulation of catabolic process	50/2440	1.19E-05	0.000126769
GO:0008210	estrogen metabolic process	Oct-40	1.20E-05	0.000127249
GO:0046496	nicotinamide nucleotide metabolic process	Oct-40	1.20E-05	0.000127249

GO:0055094	response to lipoprotein particle	Dec-40	1.24E-05	0.000130524
GO:0006626	protein targeting to mitochondrion	19/2440	1.36E-05	0.000143879
GO:0006071	glycerol metabolic process	Sep-40	1.56E-05	0.000163428
GO:0009067	aspartate family amino acid biosynthetic process	Sep-40	1.56E-05	0.000163428
GO:0071397	cellular response to cholesterol	Sep-40	1.56E-05	0.000163428
GO:0098856	intestinal lipid absorption	Sep-40	1.56E-05	0.000163428
GO:0001867	complement activation, lectin pathway	Jul-40	1.67E-05	0.000173467
GO:0006000	fructose metabolic process	Jul-40	1.67E-05	0.000173467
GO:0006534	cysteine metabolic process	Jul-40	1.67E-05	0.000173467
GO:0016558	protein import into peroxisome matrix	Jul-40	1.67E-05	0.000173467
GO:0061365	positive regulation of triglyceride lipase activity	Jul-40	1.67E-05	0.000173467
GO:0044275	cellular carbohydrate catabolic process	Dec-40	1.74E-05	0.000179355
GO:1903715	regulation of aerobic respiration	Dec-40	1.74E-05	0.000179355
GO:0009128	purine nucleoside monophosphate catabolic process	Aug-40	1.81E-05	0.000186122
GO:0030299	intestinal cholesterol absorption	Aug-40	1.81E-05	0.000186122
GO:0090114	COPII-coated vesicle budding	Oct-40	1.85E-05	0.00019032
GO:1903578	regulation of ATP metabolic process	21/2440	1.86E-05	0.000190424
GO:0046033	AMP metabolic process	Nov-40	1.88E-05	0.000192342

GO:0030148	sphingolipid biosynthetic process	22/2440	1.89E-05	0.000192432
GO:0044264	cellular polysaccharide metabolic process	22/2440	1.89E-05	0.000192432
GO:0006636	unsaturated fatty acid biosynthetic process	13/2440	2.04E-05	0.000207834
GO:0010907	positive regulation of glucose metabolic process	14/2440	2.20E-05	0.000223302
GO:0032868	response to insulin	41/2440	2.21E-05	0.000224561
GO:0090199	regulation of release of cytochrome c from mitochondria	15/2440	2.23E-05	0.000225421
GO:0006914	autophagy	66/2440	2.24E-05	0.000225925
GO:0061919	process utilizing autophagic mechanism	66/2440	2.24E-05	0.000225925
GO:0006662	glycerol ether metabolic process	Sep-40	2.53E-05	0.0002542
GO:0046653	tetrahydrofolate metabolic process	Sep-40	2.53E-05	0.0002542
GO:0006665	sphingolipid metabolic process	28/2440	2.64E-05	0.000264787
GO:0001889	liver development	26/2440	2.64E-05	0.000264787
GO:0009126	purine nucleoside monophosphate metabolic process	13/2440	2.73E-05	0.000272875
GO:0006760	folic acid-containing compound metabolic process	Oct-40	2.78E-05	0.000277784
GO:0090150	establishment of protein localization to membrane	42/2440	2.86E-05	0.000285007
GO:0009749	response to glucose	34/2440	3.04E-05	0.000302573
GO:0006112	energy reserve metabolic process	20/2440	3.09E-05	0.000306776
GO:0035459	vesicle cargo loading	Aug-40	3.16E-05	0.000313494

GO:0051651	maintenance of location in cell	38/2440	3.22E-05	0.000318391
GO:0000255	allantoin metabolic process	Jul-40	3.36E-05	0.000330203
GO:0034374	low-density lipoprotein particle remodeling	Jul-40	3.36E-05	0.000330203
GO:0046054	dGMP metabolic process	Jul-40	3.36E-05	0.000330203
GO:1904729	regulation of intestinal lipid absorption	Jul-40	3.36E-05	0.000330203
GO:1903409	reactive oxygen species biosynthetic process	16/2440	3.39E-05	0.000332866
GO:0006984	ER-nucleus signaling pathway	13/2440	3.61E-05	0.000353479
GO:0050766	positive regulation of phagocytosis	20/2440	3.68E-05	0.00036005
GO:0006656	phosphatidylcholine biosynthetic process	Sep-40	3.96E-05	0.000384924
GO:0071404	cellular response to low-density lipoprotein particle stimulus	Sep-40	3.96E-05	0.000384924
GO:1903427	negative regulation of reactive oxygen species biosynthetic process	Sep-40	3.96E-05	0.000384924
GO:0061008	hepaticobiliary system development	26/2440	4.00E-05	0.000388645
GO:0034637	cellular carbohydrate biosynthetic process	18/2440	4.12E-05	0.00039907
GO:0005976	polysaccharide metabolic process	23/2440	4.12E-05	0.00039907
GO:0043457	regulation of cellular respiration	16/2440	4.21E-05	0.000407315
GO:0045913	positive regulation of carbohydrate metabolic process	19/2440	4.30E-05	0.000414973
GO:0072655	establishment of protein localization to mitochondrion	21/2440	4.36E-05	0.00041965
GO:0005978	glycogen biosynthetic process	13/2440	4.73E-05	0.000453642

GO:0009250	glucan biosynthetic process	13/2440	4.73E-05	0.000453642
GO:0050764	regulation of phagocytosis	24/2440	5.25E-05	0.000502762
GO:0002526	acute inflammatory response	23/2440	5.55E-05	0.00053076
GO:0006707	cholesterol catabolic process	Jun-40	5.70E-05	0.000542078
GO:0016127	sterol catabolic process	Jun-40	5.70E-05	0.000542078
GO:0046053	dAMP metabolic process	Jun-40	5.70E-05	0.000542078
GO:1903027	regulation of opsonization	Jun-40	5.70E-05	0.000542078
GO:0009167	purine ribonucleoside monophosphate metabolic process	Dec-40	5.91E-05	0.000560766
GO:0010984	regulation of lipoprotein particle clearance	Sep-40	6.01E-05	0.000568301
GO:0042219	cellular modified amino acid catabolic process	Sep-40	6.01E-05	0.000568301
GO:0046456	icosanoid biosynthetic process	14/2440	6.02E-05	0.000568301
GO:0007568	aging	19/2440	6.12E-05	0.000576751
GO:0034599	cellular response to oxidative stress	43/2440	6.17E-05	0.00058003
GO:0002138	retinoic acid biosynthetic process	Jul-40	6.23E-05	0.000580876
GO:0016102	diterpenoid biosynthetic process	Jul-40	6.23E-05	0.000580876
GO:0030497	fatty acid elongation	Jul-40	6.23E-05	0.000580876
GO:0042559	pteridine-containing compound biosynthetic process	Jul-40	6.23E-05	0.000580876
GO:0090110	COPII-coated vesicle cargo loading	Jul-40	6.23E-05	0.000580876

GO:0042886	amide transport	52/2440	6.37E-05	0.000593147
GO:0031348	negative regulation of defense response	39/2440	6.74E-05	0.00062708
GO:0002697	regulation of immune effector process	61/2440	7.26E-05	0.000674518
GO:0035592	establishment of protein localization to extracellular region	57/2440	7.45E-05	0.000690319
GO:1903573	negative regulation of response to endoplasmic reticulum stress	Dec-40	7.80E-05	0.000721636
GO:0035461	vitamin transmembrane transport	Aug-40	8.42E-05	0.000777009
GO:0044241	lipid digestion	Aug-40	8.42E-05	0.000777009
GO:0050820	positive regulation of coagulation	Sep-40	8.90E-05	0.000819374
GO:0006643	membrane lipid metabolic process	32/2440	9.08E-05	0.000834734
GO:0001836	release of cytochrome c from mitochondria	16/2440	9.53E-05	0.000875275
GO:0032365	intracellular lipid transport	Dec-40	0.000101788	0.000932973
GO:0009155	purine deoxyribonucleotide catabolic process	Jul-40	0.000108173	0.000978889
GO:0009169	purine ribonucleoside monophosphate catabolic process	Jul-40	0.000108173	0.000978889
GO:0015936	coenzyme A metabolic process	Jul-40	0.000108173	0.000978889
GO:0032933	SREBP signaling pathway	Jul-40	0.000108173	0.000978889
GO:0034638	phosphatidylcholine catabolic process	Jul-40	0.000108173	0.000978889
GO:0042182	ketone catabolic process	Jul-40	0.000108173	0.000978889
GO:0042574	retinal metabolic process	Jul-40	0.000108173	0.000978889

GO:0055089	fatty acid homeostasis	Jul-40	0.000108173	0.000978889
GO:0071692	protein localization to extracellular region	57/2440	0.000111932	0.001011295
GO:0097193	intrinsic apoptotic signaling pathway	46/2440	0.000117784	0.001062486
GO:0061041	regulation of wound healing	25/2440	0.000123007	0.001107844
GO:0002082	regulation of oxidative phosphorylation	Sep-40	0.000128584	0.001155757
GO:0009306	protein secretion	56/2440	0.000128936	0.001155757
GO:0034655	nucleobase-containing compound catabolic process	56/2440	0.000128936	0.001155757
GO:0050665	hydrogen peroxide biosynthetic process	Aug-40	0.000129964	0.001163135
GO:0003333	amino acid transmembrane transport	20/2440	0.000134349	0.001200494
GO:0071496	cellular response to external stimulus	45/2440	0.000135538	0.001209222
GO:0007584	response to nutrient	16/2440	0.000139279	0.001240655
GO:0008643	carbohydrate transport	28/2440	0.000144502	0.001285169
GO:0017038	protein import	37/2440	0.000151651	0.001346644
GO:0002683	negative regulation of immune system process	61/2440	0.000156382	0.001385116
GO:0032869	cellular response to insulin stimulus	34/2440	0.000156609	0.001385116
GO:0062125	regulation of mitochondrial gene expression	Oct-40	0.000156714	0.001385116
GO:0006584	catecholamine metabolic process	15/2440	0.000160021	0.001405618
GO:0009712	catechol-containing compound metabolic process	15/2440	0.000160021	0.001405618

GO:0010676	positive regulation of cellular carbohydrate metabolic process	15/2440	0.000160021	0.001405618
GO:0015807	L-amino acid transport	15/2440	0.000160021	0.001405618
GO:0009309	amine biosynthetic process	Dec-40	0.000168564	0.001475885
GO:0042401	cellular biogenic amine biosynthetic process	Dec-40	0.000168564	0.001475885
GO:0000002	mitochondrial genome maintenance	Nov-40	0.000168798	0.001475885
GO:0071375	cellular response to peptide hormone stimulus	41/2440	0.00017266	0.001507331
GO:0007586	digestion	25/2440	0.000177483	0.001546589
GO:0042159	lipoprotein catabolic process	Jul-40	0.000178244	0.001546589
GO:0071501	cellular response to sterol depletion	Jul-40	0.000178244	0.001546589
GO:1902001	fatty acid transmembrane transport	Jul-40	0.000178244	0.001546589
GO:0120162	positive regulation of cold-induced thermogenesis	20/2440	0.000180529	0.001564007
GO:0072527	pyrimidine-containing compound metabolic process	14/2440	0.000180801	0.001564007
GO:0046321	positive regulation of fatty acid oxidation	Aug-40	0.000194386	0.001676436
GO:0071071	regulation of phospholipid biosynthetic process	Aug-40	0.000194386	0.001676436
GO:0060191	regulation of lipase activity	18/2440	0.000196735	0.001694125
GO:0010822	positive regulation of mitochondrion organization	17/2440	0.000200495	0.00172389
GO:2000112	regulation of cellular macromolecule biosynthetic process	64/2440	0.000211058	0.001811977
GO:0034248	regulation of cellular amide metabolic process	61/2440	0.000212074	0.001817957

GO:0008207	C21-steroid hormone metabolic process	Dec-40	0.000214052	0.001831364
GO:0006213	pyrimidine nucleoside metabolic process	Jun-40	0.000216212	0.001831364
GO:0006686	sphingomyelin biosynthetic process	Jun-40	0.000216212	0.001831364
GO:0009068	aspartate family amino acid catabolic process	Jun-40	0.000216212	0.001831364
GO:0032000	positive regulation of fatty acid beta-oxidation	Jun-40	0.000216212	0.001831364
GO:0034310	primary alcohol catabolic process	Jun-40	0.000216212	0.001831364
GO:0035437	maintenance of protein localization in endoplasmic reticulum	Jun-40	0.000216212	0.001831364
GO:0090209	negative regulation of triglyceride metabolic process	Jun-40	0.000216212	0.001831364
GO:0033692	cellular polysaccharide biosynthetic process	15/2440	0.000233457	0.0019745
GO:0062197	cellular response to chemical stress	48/2440	0.000248605	0.002099492
GO:0006862	nucleotide transport	Sep-40	0.000252481	0.002125916
GO:0015740	C4-dicarboxylate transport	Sep-40	0.000252481	0.002125916
GO:0031669	cellular response to nutrient levels	33/2440	0.000259499	0.002181788
GO:0043030	regulation of macrophage activation	14/2440	0.000269051	0.002255429
GO:0043470	regulation of carbohydrate catabolic process	14/2440	0.000269051	0.002255429
GO:0006991	response to sterol depletion	Jul-40	0.000280885	0.002344274
GO:0009176	pyrimidine deoxyribonucleoside monophosphate metabolic process	Jul-40	0.000280885	0.002344274
GO:1901569	fatty acid derivative catabolic process	Jul-40	0.000280885	0.002344274

GO:0034755	iron ion transmembrane transport	Aug-40	0.000282858	0.002353843
GO:0150104	transport across blood-brain barrier	Aug-40	0.000282858	0.002353843
GO:0031330	negative regulation of cellular catabolic process	38/2440	0.000284631	0.002365137
GO:0051881	regulation of mitochondrial membrane potential	18/2440	0.000311799	0.002587115
GO:0046467	membrane lipid biosynthetic process	24/2440	0.00031558	0.002614676
GO:0097035	regulation of membrane lipid distribution	14/2440	0.000325559	0.002689526
GO:1902475	L-alpha-amino acid transmembrane transport	14/2440	0.000325559	0.002689526
GO:1901653	cellular response to peptide	45/2440	0.000330732	0.002728295
GO:0042417	dopamine metabolic process	Dec-40	0.000336881	0.002775005
GO:0009100	glycoprotein metabolic process	47/2440	0.000352687	0.002901005
GO:0017004	cytochrome complex assembly	Oct-40	0.000366199	0.003003463
GO:0046475	glycerophospholipid catabolic process	Oct-40	0.000366199	0.003003463
GO:0009084	glutamine family amino acid biosynthetic process	Jun-40	0.000372745	0.003043998
GO:0034982	mitochondrial protein processing	Jun-40	0.000372745	0.003043998
GO:0035999	tetrahydrofolate interconversion	Jun-40	0.000372745	0.003043998
GO:0003018	vascular process in circulatory system	36/2440	0.000376318	0.003068769
GO:1900542	regulation of purine nucleotide metabolic process	17/2440	0.000379536	0.003090583
GO:0022600	digestive system process	22/2440	0.000388598	0.003159854

GO:0010232	vascular transport	Aug-40	0.000401656	0.003252102
GO:0019835	cytolysis	Aug-40	0.000401656	0.003252102
GO:0090201	negative regulation of release of cytochrome c from mitochondria	Aug-40	0.000401656	0.003252102
GO:0000266	mitochondrial fission	Dec-40	0.000417863	0.003378517
GO:0006865	amino acid transport	27/2440	0.000424474	0.003420895
GO:0002455	humoral immune response mediated by circulating immunoglobulin	30/2440	0.000424627	0.003420895
GO:0042953	lipoprotein transport	Jul-40	0.000426109	0.003420895
GO:1901570	fatty acid derivative biosynthetic process	Jul-40	0.000426109	0.003420895
GO:1901663	quinone biosynthetic process	Jul-40	0.000426109	0.003420895
GO:0006140	regulation of nucleotide metabolic process	17/2440	0.000441537	0.003539759
GO:0006497	protein lipidation	19/2440	0.000447597	0.003583291
GO:2001234	negative regulation of apoptotic signaling pathway	37/2440	0.00045189	0.003612584
GO:1902932	positive regulation of alcohol biosynthetic process	Sep-40	0.000461921	0.003687591
GO:0050673	epithelial cell proliferation	62/2440	0.000463401	0.003694226
GO:0006801	superoxide metabolic process	15/2440	0.000470485	0.003745454
GO:0000959	mitochondrial RNA metabolic process	Dec-40	0.000514665	0.004091446
GO:0003044	regulation of systemic arterial blood pressure mediated by a chemical signal	13/2440	0.000548084	0.004351035
GO:1900407	regulation of cellular response to oxidative stress	18/2440	0.000553199	0.004385525

GO:0002698	negative regulation of immune effector process	23/2440	0.000556061	0.004402086
GO:0034383	low-density lipoprotein particle clearance	Aug-40	0.000558003	0.004405207
GO:0098869	cellular oxidant detoxification	Aug-40	0.000558003	0.004405207
GO:0072595	maintenance of protein localization in organelle	Nov-40	0.000569533	0.004490001
GO:0031331	positive regulation of cellular catabolic process	60/2440	0.00057759	0.004547223
GO:0006067	ethanol metabolic process	Jun-40	0.000605616	0.004715685
GO:0006465	signal peptide processing	Jun-40	0.000605616	0.004715685
GO:0006555	methionine metabolic process	Jun-40	0.000605616	0.004715685
GO:0008228	opsonization	Jun-40	0.000605616	0.004715685
GO:0045916	negative regulation of complement activation	Jun-40	0.000605616	0.004715685
GO:0046073	dTMP metabolic process	Jun-40	0.000605616	0.004715685
GO:0070207	protein homotrimerization	Jun-40	0.000605616	0.004715685
GO:2001140	positive regulation of phospholipid transport	Jun-40	0.000605616	0.004715685
GO:0097242	amyloid-beta clearance	Oct-40	0.000607039	0.00472031
GO:0032369	negative regulation of lipid transport	Sep-40	0.000610671	0.00473561
GO:0043032	positive regulation of macrophage activation	Sep-40	0.000610671	0.00473561
GO:0050708	regulation of protein secretion	43/2440	0.000623645	0.00480837
GO:0044539	long-chain fatty acid import into cell	Jul-40	0.000625541	0.00480837

GO:0044872	lipoprotein localization	Jul-40	0.000625541	0.00480837
GO:0045821	positive regulation of glycolytic process	Jul-40	0.000625541	0.00480837
GO:0046485	ether lipid metabolic process	Jul-40	0.000625541	0.00480837
GO:0050746	regulation of lipoprotein metabolic process	Jul-40	0.000625541	0.00480837
GO:0061912	selective autophagy	16/2440	0.000625967	0.00480837
GO:2001233	regulation of apoptotic signaling pathway	54/2440	0.000635641	0.004876103
GO:0045088	regulation of innate immune response	36/2440	0.000658555	0.005045083
GO:0042446	hormone biosynthetic process	14/2440	0.000664332	0.005070264
GO:0046173	polyol biosynthetic process	14/2440	0.000664332	0.005070264
GO:1902882	regulation of response to oxidative stress	19/2440	0.000664515	0.005070264
GO:0071453	cellular response to oxygen levels	25/2440	0.000669305	0.005099981
GO:0007029	endoplasmic reticulum organization	18/2440	0.000725074	0.00551754
GO:1903034	regulation of response to wounding	28/2440	0.00073347	0.005573981
GO:0051224	negative regulation of protein transport	24/2440	0.000758744	0.005685141
GO:0006196	AMP catabolic process	May-40	0.00076	0.005685141
GO:0006591	ornithine metabolic process	May-40	0.00076	0.005685141
GO:0007597	blood coagulation, intrinsic pathway	May-40	0.00076	0.005685141
GO:0009131	pyrimidine nucleoside monophosphate catabolic process	May-40	0.00076	0.005685141

GO:0009178	pyrimidine deoxyribonucleoside monophosphate catabolic process	May-40	0.00076	0.005685141
GO:0048840	otolith development	May-40	0.00076	0.005685141
GO:0051775	response to redox state	May-40	0.00076	0.005685141
GO:0150172	regulation of phosphatidylcholine metabolic process	May-40	0.00076	0.005685141
GO:0010884	positive regulation of lipid storage	Aug-40	0.000760083	0.005685141
GO:0015813	L-glutamate transmembrane transport	Aug-40	0.000760083	0.005685141
GO:0046037	GMP metabolic process	Aug-40	0.000760083	0.005685141
GO:0070873	regulation of glycogen metabolic process	Oct-40	0.000769351	0.005746907
GO:0044070	regulation of anion transport	21/2440	0.000780394	0.005821759
GO:0070059	intrinsic apoptotic signaling pathway in response to endoplasmic reticulum stress	14/2440	0.000784977	0.005848281
GO:0051353	positive regulation of oxidoreductase activity	13/2440	0.000788518	0.00586699
GO:0000422	autophagy of mitochondrion	16/2440	0.000840479	0.006237298
GO:0061726	mitochondrion disassembly	16/2440	0.000840479	0.006237298
GO:1901998	toxin transport	Nov-40	0.000871804	0.006461338
GO:0000271	polysaccharide biosynthetic process	15/2440	0.000888566	0.006577003
GO:0006089	lactate metabolic process	Jul-40	0.000892391	0.006588181
GO:0140354	lipid import into cell	Jul-40	0.000892391	0.006588181
GO:0000097	sulfur amino acid biosynthetic process	Jun-40	0.000937245	0.006839482

GO:0006577	amino-acid betaine metabolic process	Jun-40	0.000937245	0.006839482
GO:0006596	polyamine biosynthetic process	Jun-40	0.000937245	0.006839482
GO:0036498	IRE1-mediated unfolded protein response	Jun-40	0.000937245	0.006839482
GO:0046322	negative regulation of fatty acid oxidation	Jun-40	0.000937245	0.006839482
GO:0060330	regulation of response to interferon-gamma	Jun-40	0.000937245	0.006839482
GO:0060334	regulation of interferon-gamma-mediated signaling pathway	Jun-40	0.000937245	0.006839482
GO:0061709	reticulophagy	Jun-40	0.000937245	0.006839482
GO:2001138	regulation of phospholipid transport	Jun-40	0.000937245	0.006839482
GO:2001242	regulation of intrinsic apoptotic signaling pathway	28/2440	0.000962846	0.00701668
GO:0032692	negative regulation of interleukin-1 production	Oct-40	0.000965722	0.00701668
GO:0000045	autophagosome assembly	19/2440	0.000966458	0.00701668
GO:0032479	regulation of type I interferon production	19/2440	0.000966458	0.00701668
GO:1905037	autophagosome organization	20/2440	0.000978511	0.007095142
GO:0015833	peptide transport	44/2440	0.000981705	0.007109245
GO:0050687	negative regulation of defense response to virus	Aug-40	0.001017039	0.007337121
GO:0070129	regulation of mitochondrial translation	Aug-40	0.001017039	0.007337121
GO:0097164	ammonium ion metabolic process	Aug-40	0.001017039	0.007337121
GO:0006734	NADH metabolic process	Sep-40	0.001025544	0.00738911

GO:1902600	proton transmembrane transport	17/2440	0.001028361	0.007400036
GO:0098586	cellular response to virus	15/2440	0.001032144	0.007417886
GO:0042594	response to starvation	29/2440	0.001088716	0.00781459
GO:0001678	cellular glucose homeostasis	27/2440	0.001104759	0.00791976
GO:0006900	vesicle budding from membrane	13/2440	0.001111834	0.007960451
GO:0007162	negative regulation of cell adhesion	43/2440	0.001132046	0.008094979
GO:0016486	peptide hormone processing	Jul-40	0.001241393	0.00883251
GO:0030194	positive regulation of blood coagulation	Jul-40	0.001241393	0.00883251
GO:0060333	interferon-gamma-mediated signaling pathway	Jul-40	0.001241393	0.00883251
GO:1900048	positive regulation of hemostasis	Jul-40	0.001241393	0.00883251
GO:1904950	negative regulation of establishment of protein localization	24/2440	0.001252695	0.008901794
GO:0090087	regulation of peptide transport	35/2440	0.001285422	0.009122967
GO:1903037	regulation of leukocyte cell-cell adhesion	46/2440	0.001291785	0.009154327
GO:0006068	ethanol catabolic process	May-40	0.001296273	0.009154327
GO:1905288	vascular associated smooth muscle cell apoptotic process	May-40	0.001296273	0.009154327
GO:1905459	regulation of vascular associated smooth muscle cell apoptotic process	May-40	0.001296273	0.009154327
GO:0050999	regulation of nitric-oxide synthase activity	Sep-40	0.001305288	0.009195173
GO:0120178	steroid hormone biosynthetic process	Sep-40	0.001305288	0.009195173

GO:0006515	protein quality control for misfolded or incompletely synthesized proteins	Aug-40	0.001338959	0.00940908
GO:0031100	animal organ regeneration	Aug-40	0.001338959	0.00940908
GO:0090276	regulation of peptide hormone secretion	34/2440	0.001379471	0.009681809
GO:0046500	S-adenosylmethionine metabolic process	Jun-40	0.001392645	0.009762236
GO:0043161	proteasome-mediated ubiquitin-dependent protein catabolic process	55/2440	0.0014227	0.009960653

GO terms associated with 3926 Rep_late_depleted_vs_Hep genes

ID	Description	GeneRatio	pvalue	p.adjust
GO:0006631	fatty acid metabolic process	245/3028	1.90E-121	1.11E-117
GO:0044282	small molecule catabolic process	200/3028	9.91E-109	2.89E-105
GO:0016054	organic acid catabolic process	156/3028	5.36E-101	1.04E-97
GO:0046395	carboxylic acid catabolic process	154/3028	2.64E-99	3.86E-96
GO:0006520	cellular amino acid metabolic process	151/3028	8.89E-80	1.04E-76
GO:0016053	organic acid biosynthetic process	160/3028	8.53E-78	8.30E-75
GO:0046394	carboxylic acid biosynthetic process	159/3028	4.59E-77	3.83E-74
GO:0006790	sulfur compound metabolic process	165/3028	5.46E-74	3.99E-71
GO:0006091	generation of precursor metabolites and energy	192/3028	1.48E-73	9.62E-71
GO:0008202	steroid metabolic process	167/3028	2.36E-73	1.38E-70
GO:1901605	alpha-amino acid metabolic process	113/3028	2.55E-63	1.35E-60
GO:0015980	energy derivation by oxidation of organic compounds	146/3028	2.88E-60	1.40E-57
GO:0072330	monocarboxylic acid biosynthetic process	116/3028	1.40E-58	6.30E-56
GO:0006753	nucleoside phosphate metabolic process	177/3028	5.91E-58	2.46E-55
GO:0009117	nucleotide metabolic process	175/3028	8.68E-58	3.38E-55
GO:0016042	lipid catabolic process	147/3028	2.02E-57	7.30E-55

GO:0072521	purine-containing compound metabolic process	162/3028	2.13E-57	7.30E-55
GO:0072329	monocarboxylic acid catabolic process	87/3028	2.65E-57	8.61E-55
GO:0006066	alcohol metabolic process	150/3028	1.15E-56	3.53E-54
GO:0045333	cellular respiration	118/3028	3.15E-56	9.19E-54
GO:0009060	aerobic respiration	99/3028	3.01E-54	8.37E-52
GO:0044242	cellular lipid catabolic process	116/3028	5.58E-54	1.48E-51
GO:0009259	ribonucleotide metabolic process	151/3028	8.37E-54	2.13E-51
GO:0019693	ribose phosphate metabolic process	153/3028	3.44E-53	8.38E-51
GO:0009150	purine ribonucleotide metabolic process	145/3028	1.50E-52	3.50E-50
GO:0016125	sterol metabolic process	94/3028	1.63E-52	3.67E-50
GO:0006163	purine nucleotide metabolic process	150/3028	4.29E-52	9.28E-50
GO:0009063	cellular amino acid catabolic process	71/3028	2.43E-49	5.06E-47
GO:0009062	fatty acid catabolic process	73/3028	5.92E-49	1.19E-46
GO:1902652	secondary alcohol metabolic process	92/3028	6.59E-49	1.28E-46
GO:0042180	cellular ketone metabolic process	113/3028	3.99E-48	7.52E-46
GO:0008203	cholesterol metabolic process	87/3028	8.15E-48	1.49E-45
GO:0006805	xenobiotic metabolic process	79/3028	2.31E-46	4.08E-44
GO:0046034	ATP metabolic process	116/3028	8.31E-45	1.43E-42

GO:0019216	regulation of lipid metabolic process	141/3028	3.58E-43	5.97E-41
GO:0033865	nucleoside bisphosphate metabolic process	71/3028	2.28E-42	3.50E-40
GO:0033875	ribonucleoside bisphosphate metabolic process	71/3028	2.28E-42	3.50E-40
GO:0034032	purine nucleoside bisphosphate metabolic process	71/3028	2.28E-42	3.50E-40
GO:0033108	mitochondrial respiratory chain complex assembly	65/3028	3.11E-42	4.66E-40
GO:0006637	acyl-CoA metabolic process	63/3028	5.29E-42	7.53E-40
GO:0035383	thioester metabolic process	63/3028	5.29E-42	7.53E-40
GO:0071466	cellular response to xenobiotic stimulus	91/3028	1.81E-41	2.52E-39
GO:0006635	fatty acid beta-oxidation	58/3028	1.98E-41	2.69E-39
GO:0006119	oxidative phosphorylation	72/3028	3.13E-41	4.15E-39
GO:0019395	fatty acid oxidation	71/3028	3.37E-41	4.38E-39
GO:0010876	lipid localization	152/3028	3.78E-41	4.80E-39
GO:0043648	dicarboxylic acid metabolic process	61/3028	2.53E-40	3.15E-38
GO:1901606	alpha-amino acid catabolic process	57/3028	6.08E-39	7.39E-37
GO:0009410	response to xenobiotic stimulus	122/3028	2.35E-38	2.80E-36
GO:0034440	lipid oxidation	71/3028	2.40E-38	2.80E-36
GO:0006633	fatty acid biosynthetic process	80/3028	5.28E-38	6.05E-36
GO:0062012	regulation of small molecule metabolic process	131/3028	8.45E-38	9.49E-36

GO:0090407	organophosphate biosynthetic process	152/3028	5.25E-37	5.78E-35
GO:0055088	lipid homeostasis	85/3028	7.28E-37	7.88E-35
GO:0006575	cellular modified amino acid metabolic process	91/3028	4.20E-36	4.39E-34
GO:0046890	regulation of lipid biosynthetic process	91/3028	4.20E-36	4.39E-34
GO:0006694	steroid biosynthetic process	82/3028	8.47E-36	8.68E-34
GO:0006869	lipid transport	131/3028	3.37E-35	3.39E-33
GO:0120254	olefinic compound metabolic process	79/3028	9.80E-35	9.70E-33
GO:0030258	lipid modification	92/3028	2.63E-34	2.56E-32
GO:0033559	unsaturated fatty acid metabolic process	70/3028	1.16E-33	1.11E-31
GO:0016126	sterol biosynthetic process	47/3028	4.94E-33	4.66E-31
GO:0006695	cholesterol biosynthetic process	44/3028	4.09E-32	3.73E-30
GO:1902653	secondary alcohol biosynthetic process	44/3028	4.09E-32	3.73E-30
GO:0022900	electron transport chain	60/3028	4.86E-32	4.36E-30
GO:0140053	mitochondrial gene expression	61/3028	1.61E-31	1.42E-29
GO:0001676	long-chain fatty acid metabolic process	65/3028	3.22E-31	2.81E-29
GO:0006641	triglyceride metabolic process	61/3028	1.28E-30	1.10E-28
GO:0045834	positive regulation of lipid metabolic process	80/3028	1.46E-30	1.24E-28
GO:0010257	NADH dehydrogenase complex assembly	43/3028	3.87E-30	3.18E-28

GO:0032981	mitochondrial respiratory chain complex I assembly	43/3028	3.87E-30	3.18E-28
GO:0022904	respiratory electron transport chain	55/3028	6.36E-30	5.16E-28
GO:0010565	regulation of cellular ketone metabolic process	71/3028	1.24E-29	9.91E-28
GO:1901617	organic hydroxy compound biosynthetic process	89/3028	8.23E-29	6.49E-27
GO:0006639	acylglycerol metabolic process	68/3028	2.45E-28	1.91E-26
GO:0006638	neutral lipid metabolic process	68/3028	6.74E-28	5.18E-26
GO:0042775	mitochondrial ATP synthesis coupled electron transport	44/3028	7.75E-27	5.88E-25
GO:0006690	icosanoid metabolic process	65/3028	8.02E-27	6.01E-25
GO:0042773	ATP synthesis coupled electron transport	44/3028	4.65E-26	3.44E-24
GO:0019646	aerobic electron transport chain	38/3028	2.05E-25	1.49E-23
GO:0046165	alcohol biosynthetic process	63/3028	2.54E-25	1.83E-23
GO:0009165	nucleotide biosynthetic process	80/3028	2.67E-25	1.90E-23
GO:0046486	glycerolipid metabolic process	113/3028	2.92E-25	2.05E-23
GO:1901293	nucleoside phosphate biosynthetic process	81/3028	3.04E-25	2.11E-23
GO:0046889	positive regulation of lipid biosynthetic process	54/3028	4.07E-25	2.80E-23
GO:0019369	arachidonic acid metabolic process	45/3028	2.22E-24	1.51E-22
GO:0055092	sterol homeostasis	51/3028	2.45E-24	1.64E-22
GO:0015711	organic anion transport	106/3028	3.00E-24	1.99E-22

GO:0042632	cholesterol homeostasis	50/3028	1.22E-23	8.01E-22
GO:0019217	regulation of fatty acid metabolic process	51/3028	1.47E-23	9.51E-22
GO:0006749	glutathione metabolic process	45/3028	1.93E-23	1.24E-21
GO:0062013	positive regulation of small molecule metabolic process	67/3028	2.13E-23	1.35E-21
GO:0006084	acetyl-CoA metabolic process	29/3028	3.21E-23	2.02E-21
GO:0034976	response to endoplasmic reticulum stress	85/3028	8.86E-23	5.50E-21
GO:1905952	regulation of lipid localization	65/3028	4.65E-22	2.86E-20
GO:0032543	mitochondrial translation	42/3028	7.49E-22	4.56E-20
GO:0009152	purine ribonucleotide biosynthetic process	61/3028	8.95E-22	5.39E-20
GO:0015850	organic hydroxy compound transport	83/3028	9.11E-22	5.43E-20
GO:0006720	isoprenoid metabolic process	45/3028	9.78E-22	5.77E-20
GO:0072522	purine-containing compound biosynthetic process	66/3028	1.71E-21	9.98E-20
GO:0046390	ribose phosphate biosynthetic process	65/3028	3.03E-21	1.75E-19
GO:0009260	ribonucleotide biosynthetic process	63/3028	3.09E-21	1.77E-19
GO:0050994	regulation of lipid catabolic process	39/3028	3.46E-21	1.96E-19
GO:0006164	purine nucleotide biosynthetic process	64/3028	3.71E-21	2.08E-19
GO:0090181	regulation of cholesterol metabolic process	30/3028	3.83E-21	2.13E-19
GO:0009636	response to toxic substance	53/3028	7.72E-21	4.25E-19

GO:0046942	carboxylic acid transport	88/3028	8.30E-21	4.53E-19
GO:0009069	serine family amino acid metabolic process	28/3028	1.72E-20	9.30E-19
GO:0015849	organic acid transport	93/3028	2.96E-20	1.59E-18
GO:0042445	hormone metabolic process	74/3028	3.08E-20	1.64E-18
GO:0042168	heme metabolic process	27/3028	1.25E-19	6.57E-18
GO:0015918	sterol transport	41/3028	1.78E-19	9.28E-18
GO:0033013	tetrapyrrole metabolic process	34/3028	2.10E-19	1.09E-17
GO:0090207	regulation of triglyceride metabolic process	32/3028	2.71E-19	1.39E-17
GO:0030301	cholesterol transport	39/3028	3.90E-19	1.98E-17
GO:0006644	phospholipid metabolic process	99/3028	4.71E-19	2.37E-17
GO:0006778	porphyrin-containing compound metabolic process	29/3028	6.64E-19	3.31E-17
GO:0006820	anion transport	120/3028	1.70E-18	8.40E-17
GO:0098754	detoxification	33/3028	2.57E-18	1.26E-16
GO:0097006	regulation of plasma lipoprotein particle levels	36/3028	4.15E-18	2.02E-16
GO:0007031	peroxisome organization	26/3028	8.82E-18	4.23E-16
GO:0006605	protein targeting	81/3028	8.83E-18	4.23E-16
GO:0008206	bile acid metabolic process	28/3028	1.11E-17	5.27E-16
GO:0034754	cellular hormone metabolic process	49/3028	1.18E-17	5.58E-16

GO:0009064	glutamine family amino acid metabolic process	34/3028	1.59E-17	7.41E-16
GO:0006721	terpenoid metabolic process	35/3028	2.11E-17	9.80E-16
GO:0071827	plasma lipoprotein particle organization	27/3028	2.75E-17	1.27E-15
GO:0019218	regulation of steroid metabolic process	47/3028	5.38E-17	2.46E-15
GO:0042178	xenobiotic catabolic process	22/3028	5.58E-17	2.53E-15
GO:0006081	cellular aldehyde metabolic process	34/3028	5.74E-17	2.56E-15
GO:0042304	regulation of fatty acid biosynthetic process	29/3028	5.75E-17	2.56E-15
GO:1990542	mitochondrial transmembrane transport	38/3028	5.91E-17	2.62E-15
GO:0006766	vitamin metabolic process	39/3028	6.21E-17	2.73E-15
GO:1990748	cellular detoxification	27/3028	6.94E-17	3.02E-15
GO:0008652	cellular amino acid biosynthetic process	33/3028	8.25E-17	3.57E-15
GO:0044272	sulfur compound biosynthetic process	41/3028	9.98E-17	4.29E-15
GO:0006120	mitochondrial electron transport, NADH to ubiquinone	22/3028	2.08E-16	8.87E-15
GO:0072593	reactive oxygen species metabolic process	72/3028	2.30E-16	9.73E-15
GO:0006536	glutamate metabolic process	21/3028	4.20E-16	1.77E-14
GO:0055090	acylglycerol homeostasis	25/3028	4.34E-16	1.80E-14
GO:0070328	triglyceride homeostasis	25/3028	4.34E-16	1.80E-14
GO:0051604	protein maturation	89/3028	4.72E-16	1.94E-14

GO:0071806	protein transmembrane transport	31/3028	6.05E-16	2.47E-14
GO:0006090	pyruvate metabolic process	46/3028	6.68E-16	2.71E-14
GO:0015748	organophosphate ester transport	47/3028	7.98E-16	3.21E-14
GO:0045940	positive regulation of steroid metabolic process	27/3028	8.79E-16	3.49E-14
GO:0071825	protein-lipid complex subunit organization	27/3028	8.79E-16	3.49E-14
GO:0005996	monosaccharide metabolic process	76/3028	9.20E-16	3.63E-14
GO:0097237	cellular response to toxic substance	28/3028	1.51E-15	5.94E-14
GO:0009144	purine nucleoside triphosphate metabolic process	40/3028	1.63E-15	6.34E-14
GO:1901607	alpha-amino acid biosynthetic process	30/3028	3.11E-15	1.20E-13
GO:0006783	heme biosynthetic process	20/3028	3.13E-15	1.20E-13
GO:0043467	regulation of generation of precursor metabolites and energy	51/3028	3.35E-15	1.28E-13
GO:0065002	intracellular protein transmembrane transport	29/3028	4.44E-15	1.69E-13
GO:0006699	bile acid biosynthetic process	21/3028	4.94E-15	1.86E-13
GO:0042537	benzene-containing compound metabolic process	19/3028	6.15E-15	2.30E-13
GO:0019915	lipid storage	38/3028	7.37E-15	2.74E-13
GO:1901361	organic cyclic compound catabolic process	108/3028	8.51E-15	3.14E-13
GO:0009145	purine nucleoside triphosphate biosynthetic process	33/3028	8.62E-15	3.17E-13
GO:0006099	tricarboxylic acid cycle	22/3028	1.69E-14	6.19E-13

GO:0019373	epoxygenase P450 pathway	23/3028	1.71E-14	6.20E-13
GO:0009083	branched-chain amino acid catabolic process	17/3028	1.86E-14	6.72E-13
GO:0009141	nucleoside triphosphate metabolic process	43/3028	2.34E-14	8.39E-13
GO:0035966	response to topologically incorrect protein	48/3028	3.61E-14	1.29E-12
GO:0009206	purine ribonucleoside triphosphate biosynthetic process	32/3028	3.92E-14	1.39E-12
GO:0006779	porphyrin-containing compound biosynthetic process	21/3028	4.14E-14	1.44E-12
GO:0009072	aromatic amino acid family metabolic process	21/3028	4.14E-14	1.44E-12
GO:0033014	tetrapyrrole biosynthetic process	21/3028	4.14E-14	1.44E-12
GO:0031998	regulation of fatty acid beta-oxidation	18/3028	4.67E-14	1.61E-12
GO:0046503	glycerolipid catabolic process	32/3028	6.49E-14	2.23E-12
GO:0006979	response to oxidative stress	95/3028	6.91E-14	2.36E-12
GO:0035384	thioester biosynthetic process	19/3028	7.73E-14	2.61E-12
GO:0071616	acyl-CoA biosynthetic process	19/3028	7.73E-14	2.61E-12
GO:0045923	positive regulation of fatty acid metabolic process	26/3028	8.84E-14	2.97E-12
GO:0034367	protein-containing complex remodeling	20/3028	9.95E-14	3.32E-12
GO:0046434	organophosphate catabolic process	50/3028	1.21E-13	4.02E-12
GO:0043691	reverse cholesterol transport	16/3028	1.52E-13	5.00E-12
GO:0016101	diterpenoid metabolic process	29/3028	1.60E-13	5.25E-12

GO:0009081	branched-chain amino acid metabolic process	18/3028	1.68E-13	5.49E-12
GO:0044262	cellular carbohydrate metabolic process	75/3028	1.70E-13	5.51E-12
GO:0033344	cholesterol efflux	30/3028	1.71E-13	5.52E-12
GO:0009201	ribonucleoside triphosphate biosynthetic process	32/3028	1.72E-13	5.52E-12
GO:0036503	ERAD pathway	40/3028	1.87E-13	5.98E-12
GO:0019432	triglyceride biosynthetic process	23/3028	2.03E-13	6.46E-12
GO:0034368	protein-lipid complex remodeling	19/3028	2.35E-13	7.38E-12
GO:0034369	plasma lipoprotein particle remodeling	19/3028	2.35E-13	7.38E-12
GO:0033866	nucleoside bisphosphate biosynthetic process	22/3028	2.43E-13	7.50E-12
GO:0034030	ribonucleoside bisphosphate biosynthetic process	22/3028	2.43E-13	7.50E-12
GO:0034033	purine nucleoside bisphosphate biosynthetic process	22/3028	2.43E-13	7.50E-12
GO:0050996	positive regulation of lipid catabolic process	21/3028	2.68E-13	8.23E-12
GO:0090208	positive regulation of triglyceride metabolic process	20/3028	2.69E-13	8.23E-12
GO:0015986	proton motive force-driven ATP synthesis	17/3028	3.50E-13	1.06E-11
GO:0043574	peroxisomal transport	17/3028	3.50E-13	1.06E-11
GO:0009142	nucleoside triphosphate biosynthetic process	34/3028	3.60E-13	1.08E-11
GO:1905039	carboxylic acid transmembrane transport	46/3028	3.92E-13	1.17E-11
GO:0015914	phospholipid transport	36/3028	4.06E-13	1.21E-11

GO:0019318	hexose metabolic process	68/3028	4.22E-13	1.25E-11
GO:0001523	retinoid metabolic process	28/3028	4.35E-13	1.28E-11
GO:0006754	ATP biosynthetic process	28/3028	4.35E-13	1.28E-11
GO:0009205	purine ribonucleoside triphosphate metabolic process	35/3028	4.76E-13	1.39E-11
GO:0015718	monocarboxylic acid transport	43/3028	4.88E-13	1.42E-11
GO:1903825	organic acid transmembrane transport	46/3028	5.27E-13	1.52E-11
GO:0046461	neutral lipid catabolic process	24/3028	6.49E-13	1.86E-11
GO:0046464	acylglycerol catabolic process	24/3028	6.49E-13	1.86E-11
GO:0042440	pigment metabolic process	30/3028	7.69E-13	2.19E-11
GO:0019439	aromatic compound catabolic process	99/3028	8.16E-13	2.31E-11
GO:0000959	mitochondrial RNA metabolic process	25/3028	8.65E-13	2.44E-11
GO:0007006	mitochondrial membrane organization	39/3028	9.58E-13	2.69E-11
GO:0008654	phospholipid biosynthetic process	61/3028	1.41E-12	3.95E-11
GO:0000038	very long-chain fatty acid metabolic process	21/3028	1.42E-12	3.96E-11
GO:0006839	mitochondrial transport	48/3028	1.55E-12	4.28E-11
GO:1901657	glycosyl compound metabolic process	32/3028	1.64E-12	4.48E-11
GO:0000096	sulfur amino acid metabolic process	20/3028	1.64E-12	4.48E-11
GO:0007007	inner mitochondrial membrane organization	20/3028	1.64E-12	4.48E-11

GO:0072594	establishment of protein localization to organelle	94/3028	2.25E-12	6.10E-11
GO:0009199	ribonucleoside triphosphate metabolic process	35/3028	2.26E-12	6.12E-11
GO:0042157	lipoprotein metabolic process	44/3028	3.02E-12	8.14E-11
GO:0010883	regulation of lipid storage	26/3028	3.22E-12	8.62E-11
GO:0045833	negative regulation of lipid metabolic process	38/3028	3.37E-12	9.00E-11
GO:0010866	regulation of triglyceride biosynthetic process	17/3028	3.74E-12	9.92E-11
GO:0009991	response to extracellular stimulus	90/3028	5.03E-12	1.33E-10
GO:0006085	acetyl-CoA biosynthetic process	15/3028	5.24E-12	1.38E-10
GO:0032368	regulation of lipid transport	41/3028	7.28E-12	1.90E-10
GO:0016485	protein processing	69/3028	7.30E-12	1.90E-10
GO:1905954	positive regulation of lipid localization	36/3028	7.67E-12	1.99E-10
GO:0034377	plasma lipoprotein particle assembly	16/3028	8.56E-12	2.21E-10
GO:0046460	neutral lipid biosynthetic process	25/3028	8.76E-12	2.24E-10
GO:0046463	acylglycerol biosynthetic process	25/3028	8.76E-12	2.24E-10
GO:0046320	regulation of fatty acid oxidation	22/3028	9.26E-12	2.36E-10
GO:0016052	carbohydrate catabolic process	44/3028	1.21E-11	3.08E-10
GO:0034381	plasma lipoprotein particle clearance	21/3028	1.29E-11	3.25E-10
GO:1901661	quinone metabolic process	21/3028	1.29E-11	3.25E-10

GO:0034370	triglyceride-rich lipoprotein particle remodeling	Nov-28	1.70E-11	4.26E-10
GO:0008299	isoprenoid biosynthetic process	20/3028	1.72E-11	4.30E-10
GO:0045540	regulation of cholesterol biosynthetic process	15/3028	1.89E-11	4.68E-10
GO:0106118	regulation of sterol biosynthetic process	15/3028	1.89E-11	4.68E-10
GO:1901136	carbohydrate derivative catabolic process	45/3028	1.94E-11	4.79E-10
GO:0009071	serine family amino acid catabolic process	Dec-28	2.09E-11	5.13E-10
GO:0019433	triglyceride catabolic process	19/3028	2.18E-11	5.29E-10
GO:0032371	regulation of sterol transport	19/3028	2.18E-11	5.29E-10
GO:0032374	regulation of cholesterol transport	19/3028	2.18E-11	5.29E-10
GO:0006006	glucose metabolic process	59/3028	2.66E-11	6.41E-10
GO:0006986	response to unfolded protein	39/3028	3.15E-11	7.57E-10
GO:0006625	protein targeting to peroxisome	14/3028	3.96E-11	9.32E-10
GO:0051004	regulation of lipoprotein lipase activity	14/3028	3.96E-11	9.32E-10
GO:0072662	protein localization to peroxisome	14/3028	3.96E-11	9.32E-10
GO:0072663	establishment of protein localization to peroxisome	14/3028	3.96E-11	9.32E-10
GO:0090205	positive regulation of cholesterol metabolic process	14/3028	3.96E-11	9.32E-10
GO:0034308	primary alcohol metabolic process	31/3028	4.67E-11	1.10E-09
GO:0043434	response to peptide hormone	80/3028	5.76E-11	1.34E-09

GO:0009116	nucleoside metabolic process	25/3028	6.71E-11	1.56E-09
GO:0006544	glycine metabolic process	13/3028	7.68E-11	1.77E-09
GO:0009074	aromatic amino acid family catabolic process	13/3028	7.68E-11	1.77E-09
GO:0051881	regulation of mitochondrial membrane potential	32/3028	7.76E-11	1.79E-09
GO:0017004	cytochrome complex assembly	19/3028	9.47E-11	2.17E-09
GO:0006888	endoplasmic reticulum to Golgi vesicle-mediated transport	38/3028	1.01E-10	2.30E-09
GO:0009262	deoxyribonucleotide metabolic process	23/3028	1.10E-10	2.50E-09
GO:2000377	regulation of reactive oxygen species metabolic process	46/3028	1.25E-10	2.84E-09
GO:0062014	negative regulation of small molecule metabolic process	34/3028	1.28E-10	2.89E-09
GO:0015919	peroxisomal membrane transport	14/3028	1.36E-10	3.05E-09
GO:0030433	ubiquitin-dependent ERAD pathway	31/3028	1.38E-10	3.10E-09
GO:0070972	protein localization to endoplasmic reticulum	29/3028	1.46E-10	3.25E-09
GO:1901292	nucleoside phosphate catabolic process	29/3028	1.46E-10	3.25E-09
GO:0051346	negative regulation of hydrolase activity	80/3028	1.55E-10	3.44E-09
GO:0032527	protein exit from endoplasmic reticulum	21/3028	1.59E-10	3.50E-09
GO:0034372	very-low-density lipoprotein particle remodeling	Oct-28	1.62E-10	3.56E-09
GO:0035634	response to stilbenoid	15/3028	1.69E-10	3.70E-09
GO:0051791	medium-chain fatty acid metabolic process	16/3028	1.72E-10	3.75E-09

GO:0035967	cellular response to topologically incorrect protein	36/3028	1.82E-10	3.95E-09
GO:0044743	protein transmembrane import into intracellular organelle	19/3028	1.87E-10	4.04E-09
GO:0042407	cristae formation	13/3028	2.95E-10	6.33E-09
GO:0042776	proton motive force-driven mitochondrial ATP synthesis	13/3028	2.95E-10	6.33E-09
GO:0009394	2'-deoxyribonucleotide metabolic process	22/3028	2.99E-10	6.38E-09
GO:0019692	deoxyribose phosphate metabolic process	22/3028	2.99E-10	6.38E-09
GO:0010038	response to metal ion	68/3028	3.38E-10	7.17E-09
GO:0065005	protein-lipid complex assembly	16/3028	4.03E-10	8.53E-09
GO:0006879	cellular iron ion homeostasis	27/3028	4.55E-10	9.60E-09
GO:0042060	wound healing	80/3028	4.62E-10	9.71E-09
GO:0050810	regulation of steroid biosynthetic process	30/3028	4.93E-10	1.03E-08
GO:0009112	nucleobase metabolic process	18/3028	5.08E-10	1.06E-08
GO:0009166	nucleotide catabolic process	26/3028	5.40E-10	1.12E-08
GO:0031668	cellular response to extracellular stimulus	58/3028	5.95E-10	1.23E-08
GO:0046185	aldehyde catabolic process	Dec-28	5.97E-10	1.23E-08
GO:0046470	phosphatidylcholine metabolic process	25/3028	6.26E-10	1.29E-08
GO:0009066	aspartate family amino acid metabolic process	19/3028	6.60E-10	1.35E-08
GO:0098656	anion transmembrane transport	52/3028	6.71E-10	1.37E-08

GO:0055072	iron ion homeostasis	32/3028	7.71E-10	1.57E-08
GO:0048193	Golgi vesicle transport	64/3028	8.54E-10	1.73E-08
GO:0045723	positive regulation of fatty acid biosynthetic process	16/3028	8.92E-10	1.80E-08
GO:0031667	response to nutrient levels	79/3028	9.62E-10	1.94E-08
GO:0007599	hemostasis	47/3028	9.74E-10	1.95E-08
GO:0050817	coagulation	47/3028	9.74E-10	1.95E-08
GO:1901652	response to peptide	86/3028	1.03E-09	2.05E-08
GO:0010878	cholesterol storage	15/3028	1.06E-09	2.08E-08
GO:0016226	iron-sulfur cluster assembly	15/3028	1.06E-09	2.08E-08
GO:0031163	metallo-sulfur cluster assembly	15/3028	1.06E-09	2.08E-08
GO:0006626	protein targeting to mitochondrion	28/3028	1.12E-09	2.20E-08
GO:0015721	bile acid and bile salt transport	17/3028	1.39E-09	2.71E-08
GO:0045922	negative regulation of fatty acid metabolic process	17/3028	1.39E-09	2.71E-08
GO:0072655	establishment of protein localization to mitochondrion	32/3028	1.41E-09	2.75E-08
GO:0055076	transition metal ion homeostasis	40/3028	1.51E-09	2.94E-08
GO:0033539	fatty acid beta-oxidation using acyl-CoA dehydrogenase	Oct-28	1.61E-09	3.12E-08
GO:0006826	iron ion transport	24/3028	1.65E-09	3.18E-08
GO:0007596	blood coagulation	46/3028	1.69E-09	3.26E-08

GO:0045017	glycerolipid biosynthetic process	53/3028	1.72E-09	3.28E-08
GO:0042908	xenobiotic transport	18/3028	1.81E-09	3.45E-08
GO:0070585	protein localization to mitochondrion	33/3028	1.87E-09	3.56E-08
GO:2000378	negative regulation of reactive oxygen species metabolic process	23/3028	1.88E-09	3.56E-08
GO:0006767	water-soluble vitamin metabolic process	19/3028	2.09E-09	3.95E-08
GO:0090150	establishment of protein localization to membrane	60/3028	2.15E-09	4.06E-08
GO:0006650	glycerophospholipid metabolic process	63/3028	2.61E-09	4.90E-08
GO:0042730	fibrinolysis	13/3028	2.74E-09	5.07E-08
GO:1904478	regulation of intestinal absorption	13/3028	2.74E-09	5.07E-08
GO:0009164	nucleoside catabolic process	14/3028	2.74E-09	5.07E-08
GO:0010885	regulation of cholesterol storage	14/3028	2.74E-09	5.07E-08
GO:1903427	negative regulation of reactive oxygen species biosynthetic process	14/3028	2.74E-09	5.07E-08
GO:0009132	nucleoside diphosphate metabolic process	38/3028	2.97E-09	5.47E-08
GO:0046148	pigment biosynthetic process	22/3028	3.24E-09	5.94E-08
GO:0006739	NADP metabolic process	19/3028	3.59E-09	6.57E-08
GO:0042572	retinol metabolic process	16/3028	3.78E-09	6.90E-08
GO:0009308	amine metabolic process	37/3028	4.15E-09	7.54E-08
GO:0051341	regulation of oxidoreductase activity	31/3028	4.55E-09	8.24E-08

GO:0046364	monosaccharide biosynthetic process	30/3028	4.56E-09	8.24E-08
GO:0030970	retrograde protein transport, ER to cytosol	15/3028	5.08E-09	9.10E-08
GO:0034656	nucleobase-containing small molecule catabolic process	15/3028	5.08E-09	9.10E-08
GO:1903513	endoplasmic reticulum to cytosol transport	15/3028	5.08E-09	9.10E-08
GO:0046916	cellular transition metal ion homeostasis	34/3028	5.28E-09	9.43E-08
GO:1903578	regulation of ATP metabolic process	30/3028	6.12E-09	1.09E-07
GO:0072525	pyridine-containing compound biosynthetic process	14/3028	6.33E-09	1.12E-07
GO:0006103	2-oxoglutarate metabolic process	Dec-28	6.63E-09	1.17E-07
GO:0010896	regulation of triglyceride catabolic process	Dec-28	6.63E-09	1.17E-07
GO:1903008	organelle disassembly	34/3028	6.82E-09	1.20E-07
GO:0051235	maintenance of location	72/3028	6.83E-09	1.20E-07
GO:0006692	prostanoid metabolic process	22/3028	7.57E-09	1.32E-07
GO:0006693	prostaglandin metabolic process	22/3028	7.57E-09	1.32E-07
GO:0006109	regulation of carbohydrate metabolic process	49/3028	8.39E-09	1.46E-07
GO:0006086	acetyl-CoA biosynthetic process from pyruvate	Oct-28	8.76E-09	1.51E-07
GO:0006534	cysteine metabolic process	Oct-28	8.76E-09	1.51E-07
GO:0032000	positive regulation of fatty acid beta-oxidation	Oct-28	8.76E-09	1.51E-07
GO:0072523	purine-containing compound catabolic process	20/3028	9.46E-09	1.62E-07

GO:0009264	deoxyribonucleotide catabolic process	15/3028	1.03E-08	1.77E-07
GO:0044042	glucan metabolic process	27/3028	1.06E-08	1.81E-07
GO:0008637	apoptotic mitochondrial changes	35/3028	1.31E-08	2.23E-07
GO:0009162	deoxyribonucleoside monophosphate metabolic process	16/3028	1.36E-08	2.29E-07
GO:0042558	pteridine-containing compound metabolic process	16/3028	1.36E-08	2.29E-07
GO:0045454	cell redox homeostasis	16/3028	1.36E-08	2.29E-07
GO:0062125	regulation of mitochondrial gene expression	16/3028	1.36E-08	2.29E-07
GO:0010873	positive regulation of cholesterol esterification	Sep-28	1.40E-08	2.34E-07
GO:0110095	cellular detoxification of aldehyde	Sep-28	1.40E-08	2.34E-07
GO:1903715	regulation of aerobic respiration	17/3028	1.57E-08	2.62E-07
GO:0050892	intestinal absorption	19/3028	1.60E-08	2.67E-07
GO:0010906	regulation of glucose metabolic process	37/3028	1.65E-08	2.74E-07
GO:0044106	cellular amine metabolic process	35/3028	1.65E-08	2.74E-07
GO:0010867	positive regulation of triglyceride biosynthetic process	Dec-28	1.80E-08	2.96E-07
GO:0006457	protein folding	43/3028	1.93E-08	3.18E-07
GO:1905953	negative regulation of lipid localization	21/3028	1.99E-08	3.27E-07
GO:0042573	retinoic acid metabolic process	15/3028	2.00E-08	3.28E-07
GO:0045861	negative regulation of proteolysis	73/3028	2.19E-08	3.58E-07

GO:0030195	negative regulation of blood coagulation	20/3028	2.29E-08	3.73E-07
GO:0050818	regulation of coagulation	25/3028	2.48E-08	4.03E-07
GO:0072524	pyridine-containing compound metabolic process	17/3028	2.68E-08	4.33E-07
GO:0006730	one-carbon metabolic process	18/3028	2.69E-08	4.34E-07
GO:0044270	cellular nitrogen compound catabolic process	83/3028	2.69E-08	4.34E-07
GO:0046939	nucleotide phosphorylation	32/3028	2.79E-08	4.48E-07
GO:0008535	respiratory chain complex IV assembly	14/3028	2.81E-08	4.49E-07
GO:0044273	sulfur compound catabolic process	14/3028	2.81E-08	4.49E-07
GO:0016051	carbohydrate biosynthetic process	48/3028	2.83E-08	4.51E-07
GO:0046700	heterocycle catabolic process	83/3028	3.00E-08	4.77E-07
GO:0030193	regulation of blood coagulation	24/3028	3.21E-08	5.06E-07
GO:1901616	organic hydroxy compound catabolic process	24/3028	3.21E-08	5.06E-07
GO:0034375	high-density lipoprotein particle remodeling	Oct-28	3.43E-08	5.38E-07
GO:0034982	mitochondrial protein processing	Oct-28	3.43E-08	5.38E-07
GO:0043649	dicarboxylic acid catabolic process	Oct-28	3.43E-08	5.38E-07
GO:1900047	negative regulation of hemostasis	20/3028	3.49E-08	5.45E-07
GO:0005977	glycogen metabolic process	26/3028	3.54E-08	5.50E-07
GO:0006073	cellular glucan metabolic process	26/3028	3.54E-08	5.50E-07

GO:0006576	cellular biogenic amine metabolic process	32/3028	3.57E-08	5.53E-07
GO:0051055	negative regulation of lipid biosynthetic process	22/3028	3.59E-08	5.55E-07
GO:0010039	response to iron ion	13/3028	3.70E-08	5.70E-07
GO:0008053	mitochondrial fusion	15/3028	3.75E-08	5.76E-07
GO:0009135	purine nucleoside diphosphate metabolic process	31/3028	3.88E-08	5.94E-07
GO:0009179	purine ribonucleoside diphosphate metabolic process	31/3028	3.88E-08	5.94E-07
GO:0009261	ribonucleotide catabolic process	19/3028	3.96E-08	6.05E-07
GO:0009185	ribonucleoside diphosphate metabolic process	33/3028	4.11E-08	6.26E-07
GO:0016236	macroautophagy	55/3028	4.16E-08	6.31E-07
GO:0034248	regulation of cellular amide metabolic process	86/3028	4.17E-08	6.31E-07
GO:0006953	acute-phase response	18/3028	4.32E-08	6.51E-07
GO:1901568	fatty acid derivative metabolic process	18/3028	4.32E-08	6.51E-07
GO:0006525	arginine metabolic process	Dec-28	4.41E-08	6.58E-07
GO:0016114	terpenoid biosynthetic process	Dec-28	4.41E-08	6.58E-07
GO:0019363	pyridine nucleotide biosynthetic process	Dec-28	4.41E-08	6.58E-07
GO:0034433	steroid esterification	Nov-28	4.48E-08	6.63E-07
GO:0034434	sterol esterification	Nov-28	4.48E-08	6.63E-07
GO:0034435	cholesterol esterification	Nov-28	4.48E-08	6.63E-07

GO:0045717	negative regulation of fatty acid biosynthetic process	Nov-28	4.48E-08	6.63E-07
GO:0052548	regulation of endopeptidase activity	72/3028	4.89E-08	7.22E-07
GO:0006165	nucleoside diphosphate phosphorylation	31/3028	4.98E-08	7.32E-07
GO:0050819	negative regulation of coagulation	20/3028	5.24E-08	7.69E-07
GO:0046031	ADP metabolic process	30/3028	5.39E-08	7.89E-07
GO:0046386	deoxyribose phosphate catabolic process	14/3028	5.50E-08	8.04E-07
GO:0034620	cellular response to unfolded protein	28/3028	6.08E-08	8.86E-07
GO:1900046	regulation of hemostasis	24/3028	6.16E-08	8.96E-07
GO:0006914	autophagy	87/3028	6.25E-08	9.04E-07
GO:0061919	process utilizing autophagic mechanism	87/3028	6.25E-08	9.04E-07
GO:0052547	regulation of peptidase activity	84/3028	6.30E-08	9.08E-07
GO:0060191	regulation of lipase activity	27/3028	6.31E-08	9.08E-07
GO:0010823	negative regulation of mitochondrion organization	21/3028	6.40E-08	9.18E-07
GO:0043651	linoleic acid metabolic process	15/3028	6.76E-08	9.66E-07
GO:1901658	glycosyl compound catabolic process	15/3028	6.76E-08	9.66E-07
GO:1903725	regulation of phospholipid metabolic process	18/3028	6.82E-08	9.72E-07
GO:0006558	L-phenylalanine metabolic process	Sep-28	6.98E-08	9.75E-07
GO:0019627	urea metabolic process	Sep-28	6.98E-08	9.75E-07

GO:0030300	regulation of intestinal cholesterol absorption	Sep-28	6.98E-08	9.75E-07
GO:0034384	high-density lipoprotein particle clearance	Sep-28	6.98E-08	9.75E-07
GO:0051006	positive regulation of lipoprotein lipase activity	Sep-28	6.98E-08	9.75E-07
GO:0071941	nitrogen cycle metabolic process	Sep-28	6.98E-08	9.75E-07
GO:1902221	erythrose 4-phosphate/phosphoenolpyruvate family amino acid metabolic process	Sep-28	6.98E-08	9.75E-07
GO:0043161	proteasome-mediated ubiquitin-dependent protein catabolic process	82/3028	6.98E-08	9.75E-07
GO:0071496	cellular response to external stimulus	63/3028	7.29E-08	1.02E-06
GO:0009065	glutamine family amino acid catabolic process	13/3028	7.68E-08	1.07E-06
GO:2000112	regulation of cellular macromolecule biosynthetic process	89/3028	8.28E-08	1.15E-06
GO:0006144	purine nucleobase metabolic process	Dec-28	9.95E-08	1.38E-06
GO:1903409	reactive oxygen species biosynthetic process	22/3028	1.03E-07	1.43E-06
GO:0032782	bile acid secretion	Oct-28	1.09E-07	1.49E-06
GO:0050667	homocysteine metabolic process	Oct-28	1.09E-07	1.49E-06
GO:0051180	vitamin transport	17/3028	1.17E-07	1.60E-06
GO:0010675	regulation of cellular carbohydrate metabolic process	41/3028	1.18E-07	1.62E-06
GO:0032868	response to insulin	54/3028	1.21E-07	1.65E-06
GO:0042278	purine nucleoside metabolic process	16/3028	1.22E-07	1.66E-06
GO:0006112	energy reserve metabolic process	27/3028	1.43E-07	1.94E-06

GO:0043457	regulation of cellular respiration	22/3028	1.44E-07	1.96E-06
GO:0009123	nucleoside monophosphate metabolic process	23/3028	1.52E-07	2.06E-06
GO:0045047	protein targeting to ER	18/3028	1.61E-07	2.18E-06
GO:0000041	transition metal ion transport	31/3028	1.63E-07	2.19E-06
GO:0006195	purine nucleotide catabolic process	17/3028	1.83E-07	2.46E-06
GO:1902600	proton transmembrane transport	27/3028	1.85E-07	2.48E-06
GO:0010821	regulation of mitochondrion organization	37/3028	1.94E-07	2.59E-06
GO:0019319	hexose biosynthetic process	26/3028	1.96E-07	2.62E-06
GO:0010875	positive regulation of cholesterol efflux	15/3028	2.01E-07	2.67E-06
GO:0046321	positive regulation of fatty acid oxidation	Dec-28	2.10E-07	2.79E-06
GO:0010898	positive regulation of triglyceride catabolic process	Sep-28	2.53E-07	3.33E-06
GO:0016558	protein import into peroxisome matrix	Sep-28	2.53E-07	3.33E-06
GO:0043650	dicarboxylic acid biosynthetic process	Sep-28	2.53E-07	3.33E-06
GO:0061365	positive regulation of triglyceride lipase activity	Sep-28	2.53E-07	3.33E-06
GO:0031638	zymogen activation	29/3028	2.55E-07	3.35E-06
GO:0002082	regulation of oxidative phosphorylation	13/3028	2.85E-07	3.72E-06
GO:0010884	positive regulation of lipid storage	13/3028	2.85E-07	3.72E-06
GO:0030449	regulation of complement activation	13/3028	2.85E-07	3.72E-06

GO:0033500	carbohydrate homeostasis	60/3028	2.88E-07	3.74E-06
GO:0006957	complement activation, alternative pathway	Oct-28	2.95E-07	3.83E-06
GO:0110096	cellular response to aldehyde	Oct-28	2.95E-07	3.83E-06
GO:0006096	glycolytic process	27/3028	3.08E-07	3.98E-06
GO:0006094	gluconeogenesis	25/3028	3.53E-07	4.55E-06
GO:1905897	regulation of response to endoplasmic reticulum stress	24/3028	3.68E-07	4.73E-06
GO:0007030	Golgi organization	35/3028	3.94E-07	5.05E-06
GO:0006757	ATP generation from ADP	27/3028	3.94E-07	5.05E-06
GO:0009895	negative regulation of catabolic process	63/3028	4.01E-07	5.13E-06
GO:1900542	regulation of purine nucleotide metabolic process	25/3028	4.59E-07	5.86E-06
GO:0070129	regulation of mitochondrial translation	13/3028	5.15E-07	6.55E-06
GO:0031669	cellular response to nutrient levels	46/3028	5.20E-07	6.61E-06
GO:0046164	alcohol catabolic process	18/3028	5.25E-07	6.66E-06
GO:0009119	ribonucleoside metabolic process	15/3028	5.37E-07	6.79E-06
GO:0000050	urea cycle	Aug-28	5.46E-07	6.87E-06
GO:0034380	high-density lipoprotein particle assembly	Aug-28	5.46E-07	6.87E-06
GO:0050878	regulation of body fluid levels	71/3028	5.50E-07	6.91E-06
GO:0044264	cellular polysaccharide metabolic process	28/3028	5.72E-07	7.16E-06

GO:0006613	cotranslational protein targeting to membrane	Nov-28	5.74E-07	7.16E-06
GO:0044241	lipid digestion	Nov-28	5.74E-07	7.16E-06
GO:0042593	glucose homeostasis	59/3028	5.75E-07	7.17E-06
GO:0006665	sphingolipid metabolic process	36/3028	5.81E-07	7.21E-06
GO:0006140	regulation of nucleotide metabolic process	25/3028	5.95E-07	7.38E-06
GO:0010951	negative regulation of endopeptidase activity	43/3028	5.98E-07	7.40E-06
GO:0042886	amide transport	68/3028	6.04E-07	7.46E-06
GO:0019748	secondary metabolic process	20/3028	6.51E-07	8.02E-06
GO:0009070	serine family amino acid biosynthetic process	Oct-28	7.13E-07	8.72E-06
GO:0019359	nicotinamide nucleotide biosynthetic process	Oct-28	7.13E-07	8.72E-06
GO:0030299	intestinal cholesterol absorption	Oct-28	7.13E-07	8.72E-06
GO:0017038	protein import	50/3028	7.14E-07	8.72E-06
GO:0031331	positive regulation of cellular catabolic process	83/3028	7.21E-07	8.79E-06
GO:0019751	polyol metabolic process	31/3028	7.35E-07	8.95E-06
GO:0006063	uronic acid metabolic process	Sep-28	7.44E-07	8.97E-06
GO:0010872	regulation of cholesterol esterification	Sep-28	7.44E-07	8.97E-06
GO:0019585	glucuronate metabolic process	Sep-28	7.44E-07	8.97E-06
GO:0070508	cholesterol import	Sep-28	7.44E-07	8.97E-06

GO:1904729	regulation of intestinal lipid absorption	Sep-28	7.44E-07	8.97E-06
GO:0072599	establishment of protein localization to endoplasmic reticulum	18/3028	7.58E-07	9.11E-06
GO:0009151	purine deoxyribonucleotide metabolic process	Dec-28	7.89E-07	9.46E-06
GO:1990845	adaptive thermogenesis	39/3028	7.98E-07	9.56E-06
GO:0032869	cellular response to insulin stimulus	46/3028	8.04E-07	9.61E-06
GO:0042743	hydrogen peroxide metabolic process	20/3028	8.96E-07	1.07E-05
GO:0019362	pyridine nucleotide metabolic process	13/3028	8.97E-07	1.07E-05
GO:0031639	plasminogen activation	13/3028	8.97E-07	1.07E-05
GO:0007584	response to nutrient	22/3028	9.04E-07	1.07E-05
GO:0010466	negative regulation of peptidase activity	54/3028	9.99E-07	1.18E-05
GO:0042398	cellular modified amino acid biosynthetic process	16/3028	1.13E-06	1.33E-05
GO:0006390	mitochondrial transcription	Nov-28	1.15E-06	1.35E-05
GO:0009159	deoxyribonucleoside monophosphate catabolic process	Nov-28	1.15E-06	1.35E-05
GO:0098856	intestinal lipid absorption	Nov-28	1.15E-06	1.35E-05
GO:0106106	cold-induced thermogenesis	36/3028	1.18E-06	1.38E-05
GO:0009154	purine ribonucleotide catabolic process	15/3028	1.31E-06	1.53E-05
GO:0032102	negative regulation of response to external stimulus	74/3028	1.33E-06	1.55E-05
GO:0098869	cellular oxidant detoxification	Dec-28	1.43E-06	1.66E-05

GO:0042430	indole-containing compound metabolic process	13/3028	1.51E-06	1.75E-05
GO:0050995	negative regulation of lipid catabolic process	13/3028	1.51E-06	1.75E-05
GO:0046415	urate metabolic process	Oct-28	1.57E-06	1.81E-05
GO:1901264	carbohydrate derivative transport	21/3028	1.63E-06	1.88E-05
GO:0006399	tRNA metabolic process	39/3028	1.78E-06	2.05E-05
GO:0006107	oxaloacetate metabolic process	Aug-28	1.82E-06	2.07E-05
GO:0006122	mitochondrial electron transport, ubiquinol to cytochrome c	Aug-28	1.82E-06	2.07E-05
GO:0009812	flavonoid metabolic process	Aug-28	1.82E-06	2.07E-05
GO:0045542	positive regulation of cholesterol biosynthetic process	Aug-28	1.82E-06	2.07E-05
GO:0106120	positive regulation of sterol biosynthetic process	Aug-28	1.82E-06	2.07E-05
GO:0010635	regulation of mitochondrial fusion	Sep-28	1.89E-06	2.13E-05
GO:0030497	fatty acid elongation	Sep-28	1.89E-06	2.13E-05
GO:0034756	regulation of iron ion transport	Sep-28	1.89E-06	2.13E-05
GO:0042559	pteridine-containing compound biosynthetic process	Sep-28	1.89E-06	2.13E-05
GO:0046459	short-chain fatty acid metabolic process	Sep-28	1.89E-06	2.13E-05
GO:0032370	positive regulation of lipid transport	23/3028	1.91E-06	2.16E-05
GO:0030148	sphingolipid biosynthetic process	27/3028	1.97E-06	2.22E-05
GO:0006775	fat-soluble vitamin metabolic process	15/3028	1.99E-06	2.24E-05

GO:0051289	protein homotetramerization	18/3028	2.12E-06	2.38E-05
GO:0033617	mitochondrial cytochrome c oxidase assembly	Nov-28	2.19E-06	2.45E-05
GO:0046653	tetrahydrofolate metabolic process	Nov-28	2.19E-06	2.45E-05
GO:0005976	polysaccharide metabolic process	29/3028	2.23E-06	2.49E-05
GO:0120161	regulation of cold-induced thermogenesis	35/3028	2.35E-06	2.62E-05
GO:0010893	positive regulation of steroid biosynthetic process	13/3028	2.48E-06	2.74E-05
GO:0008209	androgen metabolic process	Dec-28	2.48E-06	2.74E-05
GO:0009125	nucleoside monophosphate catabolic process	Dec-28	2.48E-06	2.74E-05
GO:0000266	mitochondrial fission	17/3028	2.75E-06	3.04E-05
GO:0006612	protein targeting to membrane	31/3028	2.85E-06	3.15E-05
GO:0034284	response to monosaccharide	43/3028	2.99E-06	3.29E-05
GO:0006614	SRP-dependent cotranslational protein targeting to membrane	Oct-28	3.19E-06	3.50E-05
GO:0072378	blood coagulation, fibrin clot formation	Oct-28	3.19E-06	3.50E-05
GO:0009743	response to carbohydrate	45/3028	3.40E-06	3.72E-05
GO:0006984	ER-nucleus signaling pathway	16/3028	3.50E-06	3.81E-05
GO:0010874	regulation of cholesterol efflux	16/3028	3.50E-06	3.81E-05
GO:0043039	tRNA aminoacylation	16/3028	3.50E-06	3.81E-05
GO:0030968	endoplasmic reticulum unfolded protein response	21/3028	3.69E-06	4.01E-05

GO:0042594	response to starvation	41/3028	3.90E-06	4.23E-05
GO:0006835	dicarboxylic acid transport	25/3028	3.96E-06	4.27E-05
GO:0006656	phosphatidylcholine biosynthetic process	Nov-28	3.96E-06	4.27E-05
GO:0009158	ribonucleoside monophosphate catabolic process	Nov-28	3.96E-06	4.27E-05
GO:0034599	cellular response to oxidative stress	54/3028	4.13E-06	4.44E-05
GO:0006760	folic acid-containing compound metabolic process	Dec-28	4.17E-06	4.46E-05
GO:0010888	negative regulation of lipid storage	Dec-28	4.17E-06	4.46E-05
GO:0006570	tyrosine metabolic process	Sep-28	4.28E-06	4.51E-05
GO:0010887	negative regulation of cholesterol storage	Sep-28	4.28E-06	4.51E-05
GO:0010889	regulation of sequestering of triglyceride	Sep-28	4.28E-06	4.51E-05
GO:0032373	positive regulation of sterol transport	Sep-28	4.28E-06	4.51E-05
GO:0032376	positive regulation of cholesterol transport	Sep-28	4.28E-06	4.51E-05
GO:0032933	SREBP signaling pathway	Sep-28	4.28E-06	4.51E-05
GO:0033700	phospholipid efflux	Sep-28	4.28E-06	4.51E-05
GO:0042182	ketone catabolic process	Sep-28	4.28E-06	4.51E-05
GO:0055089	fatty acid homeostasis	Sep-28	4.28E-06	4.51E-05
GO:0001659	temperature homeostasis	41/3028	4.49E-06	4.73E-05
GO:0006417	regulation of translation	71/3028	4.70E-06	4.94E-05

GO:0009746	response to hexose	42/3028	4.82E-06	5.06E-05
GO:0018958	phenol-containing compound metabolic process	31/3028	4.90E-06	5.12E-05
GO:0006152	purine nucleoside catabolic process	Aug-28	4.94E-06	5.12E-05
GO:0009435	NAD biosynthetic process	Aug-28	4.94E-06	5.12E-05
GO:0052695	cellular glucuronidation	Aug-28	4.94E-06	5.12E-05
GO:0071281	cellular response to iron ion	Aug-28	4.94E-06	5.12E-05
GO:0071712	ER-associated misfolded protein catabolic process	Aug-28	4.94E-06	5.12E-05
GO:0090209	negative regulation of triglyceride metabolic process	Aug-28	4.94E-06	5.12E-05
GO:0043038	amino acid activation	16/3028	4.97E-06	5.13E-05
GO:0071375	cellular response to peptide hormone stimulus	53/3028	5.09E-06	5.25E-05
GO:0044275	cellular carbohydrate catabolic process	14/3028	5.28E-06	5.44E-05
GO:0009161	ribonucleoside monophosphate metabolic process	18/3028	5.42E-06	5.57E-05
GO:1903362	regulation of cellular protein catabolic process	50/3028	6.01E-06	6.17E-05
GO:1904251	regulation of bile acid metabolic process	Oct-28	6.10E-06	6.25E-05
GO:0006418	tRNA aminoacylation for protein translation	15/3028	6.30E-06	6.44E-05
GO:1903573	negative regulation of response to endoplasmic reticulum stress	15/3028	6.30E-06	6.44E-05
GO:0006515	protein quality control for misfolded or incompletely synthesized proteins	Dec-28	6.78E-06	6.90E-05
GO:0000302	response to reactive oxygen species	41/3028	6.78E-06	6.90E-05

GO:0010984	regulation of lipoprotein particle clearance	Nov-28	6.87E-06	6.97E-05
GO:0019400	alditol metabolic process	Nov-28	6.87E-06	6.97E-05
GO:1902930	regulation of alcohol biosynthetic process	17/3028	7.26E-06	7.33E-05
GO:1903426	regulation of reactive oxygen species biosynthetic process	17/3028	7.26E-06	7.33E-05
GO:0051205	protein insertion into membrane	18/3028	7.26E-06	7.33E-05
GO:0035592	establishment of protein localization to extracellular region	71/3028	7.36E-06	7.41E-05
GO:0042176	regulation of protein catabolic process	70/3028	7.53E-06	7.57E-05
GO:0097193	intrinsic apoptotic signaling pathway	58/3028	8.06E-06	8.09E-05
GO:0006643	membrane lipid metabolic process	40/3028	8.33E-06	8.35E-05
GO:0042159	lipoprotein catabolic process	Sep-28	8.86E-06	8.79E-05
GO:0042454	ribonucleoside catabolic process	Sep-28	8.86E-06	8.79E-05
GO:0055091	phospholipid homeostasis	Sep-28	8.86E-06	8.79E-05
GO:0070857	regulation of bile acid biosynthetic process	Sep-28	8.86E-06	8.79E-05
GO:0071501	cellular response to sterol depletion	Sep-28	8.86E-06	8.79E-05
GO:0072350	tricarboxylic acid metabolic process	Sep-28	8.86E-06	8.79E-05
GO:0061912	selective autophagy	22/3028	9.00E-06	8.91E-05
GO:0072527	pyrimidine-containing compound metabolic process	18/3028	9.66E-06	9.54E-05
GO:0048545	response to steroid hormone	46/3028	9.72E-06	9.59E-05

GO:0046128	purine ribonucleoside metabolic process	Dec-28	1.07E-05	0.000105678
GO:0006071	glycerol metabolic process	Oct-28	1.11E-05	0.00010831
GO:0009067	aspartate family amino acid biosynthetic process	Oct-28	1.11E-05	0.00010831
GO:0009110	vitamin biosynthetic process	Oct-28	1.11E-05	0.00010831
GO:0003018	vascular process in circulatory system	47/3028	1.12E-05	0.000110063
GO:0046496	nicotinamide nucleotide metabolic process	Nov-28	1.15E-05	0.000111974
GO:0072376	protein activation cascade	Nov-28	1.15E-05	0.000111974
GO:0006568	tryptophan metabolic process	Aug-28	1.17E-05	0.000113094
GO:0006586	indolalkylamine metabolic process	Aug-28	1.17E-05	0.000113094
GO:0009120	deoxyribonucleoside metabolic process	Aug-28	1.17E-05	0.000113094
GO:0051260	protein homooligomerization	39/3028	1.17E-05	0.000113507
GO:0071692	protein localization to extracellular region	71/3028	1.24E-05	0.000119928
GO:0061045	negative regulation of wound healing	21/3028	1.27E-05	0.000122624
GO:0051353	positive regulation of oxidoreductase activity	18/3028	1.27E-05	0.000122624
GO:0009306	protein secretion	70/3028	1.27E-05	0.000122624
GO:0031647	regulation of protein stability	58/3028	1.33E-05	0.000127763
GO:0003333	amino acid transmembrane transport	25/3028	1.37E-05	0.000131611
GO:0042158	lipoprotein biosynthetic process	26/3028	1.41E-05	0.000134926

GO:0009395	phospholipid catabolic process	18/3028	1.67E-05	0.000159274
GO:0000963	mitochondrial RNA processing	Sep-28	1.71E-05	0.000162124
GO:0006991	response to sterol depletion	Sep-28	1.71E-05	0.000162124
GO:0035337	fatty-acyl-CoA metabolic process	Sep-28	1.71E-05	0.000162124
GO:0035459	vesicle cargo loading	Sep-28	1.71E-05	0.000162124
GO:0090199	regulation of release of cytochrome c from mitochondria	17/3028	1.75E-05	0.000165989
GO:0009749	response to glucose	40/3028	1.83E-05	0.000173616
GO:0018904	ether metabolic process	Nov-28	1.86E-05	0.00017482
GO:0036315	cellular response to sterol	Nov-28	1.86E-05	0.00017482
GO:0090114	COPII-coated vesicle budding	Nov-28	1.86E-05	0.00017482
GO:0060193	positive regulation of lipase activity	19/3028	1.99E-05	0.000186944
GO:0015908	fatty acid transport	25/3028	2.02E-05	0.000189226
GO:0006865	amino acid transport	35/3028	2.02E-05	0.000189719
GO:0055094	response to lipoprotein particle	13/3028	2.04E-05	0.00019139
GO:0009267	cellular response to starvation	36/3028	2.17E-05	0.000203251
GO:0051259	protein complex oligomerization	48/3028	2.25E-05	0.000209685
GO:0002920	regulation of humoral immune response	16/3028	2.39E-05	0.000222915
GO:0008207	C21-steroid hormone metabolic process	15/3028	2.40E-05	0.000222936

GO:0002138	retinoic acid biosynthetic process	Aug-28	2.47E-05	0.000228449
GO:0016102	diterpenoid biosynthetic process	Aug-28	2.47E-05	0.000228449
GO:0090110	COPII-coated vesicle cargo loading	Aug-28	2.47E-05	0.000228449
GO:0010155	regulation of proton transport	Dec-28	2.50E-05	0.000230598
GO:0031330	negative regulation of cellular catabolic process	48/3028	2.50E-05	0.00023107
GO:1901653	cellular response to peptide	57/3028	2.64E-05	0.000243427
GO:0030730	sequestering of triglyceride	Sep-28	3.09E-05	0.000283775
GO:0060192	negative regulation of lipase activity	Sep-28	3.09E-05	0.000283775
GO:1901663	quinone biosynthetic process	Sep-28	3.09E-05	0.000283775
GO:1904732	regulation of electron transfer activity	Jul-28	3.16E-05	0.000288653
GO:0009219	pyrimidine deoxyribonucleotide metabolic process	Oct-28	3.17E-05	0.000288653
GO:0034755	iron ion transmembrane transport	Oct-28	3.17E-05	0.000288653
GO:0042402	cellular biogenic amine catabolic process	Oct-28	3.17E-05	0.000288653
GO:0071404	cellular response to low-density lipoprotein particle stimulus	Oct-28	3.17E-05	0.000288653
GO:0001836	release of cytochrome c from mitochondria	19/3028	3.21E-05	0.000291124
GO:0015909	long-chain fatty acid transport	19/3028	3.21E-05	0.000291124
GO:0006890	retrograde vesicle-mediated transport, Golgi to endoplasmic reticulum	15/3028	3.25E-05	0.000294092
GO:0051262	protein tetramerization	22/3028	3.28E-05	0.000296925

GO:0061008	hepaticobiliary system development	30/3028	3.59E-05	0.000324343
GO:0036314	response to sterol	Dec-28	3.68E-05	0.000331405
GO:0072337	modified amino acid transport	Dec-28	3.68E-05	0.000331405
GO:0043090	amino acid import	17/3028	3.90E-05	0.000350908
GO:0061041	regulation of wound healing	30/3028	4.18E-05	0.000374882
GO:0006706	steroid catabolic process	Nov-28	4.45E-05	0.000397709
GO:0015740	C4-dicarboxylate transport	Nov-28	4.45E-05	0.000397709
GO:0015807	L-amino acid transport	18/3028	4.54E-05	0.000405483
GO:0009155	purine deoxyribonucleotide catabolic process	Aug-28	4.80E-05	0.000426041
GO:0009170	purine deoxyribonucleoside monophosphate metabolic process	Aug-28	4.80E-05	0.000426041
GO:0015936	coenzyme A metabolic process	Aug-28	4.80E-05	0.000426041
GO:0042574	retinal metabolic process	Aug-28	4.80E-05	0.000426041
GO:0000045	autophagosome assembly	25/3028	4.97E-05	0.000440174
GO:0015833	peptide transport	57/3028	5.03E-05	0.000445388
GO:0006801	superoxide metabolic process	19/3028	5.06E-05	0.000447246
GO:0009310	amine catabolic process	Oct-28	5.08E-05	0.00044726
GO:0070861	regulation of protein exit from endoplasmic reticulum	Oct-28	5.08E-05	0.00044726
GO:0090201	negative regulation of release of cytochrome c from mitochondria	Oct-28	5.08E-05	0.00044726

GO:0006743	ubiquinone metabolic process	Sep-28	5.33E-05	0.000466826
GO:0006851	mitochondrial calcium ion transmembrane transport	Sep-28	5.33E-05	0.000466826
GO:0035461	vitamin transmembrane transport	Sep-28	5.33E-05	0.000466826
GO:0001885	endothelial cell development	20/3028	5.46E-05	0.00047753
GO:0062197	cellular response to chemical stress	59/3028	5.72E-05	0.000499062
GO:0010907	positive regulation of glucose metabolic process	15/3028	5.78E-05	0.000503405
GO:0030808	regulation of nucleotide biosynthetic process	13/3028	5.80E-05	0.000503405
GO:0071402	cellular response to lipoprotein particle stimulus	13/3028	5.80E-05	0.000503405
GO:1900371	regulation of purine nucleotide biosynthetic process	13/3028	5.80E-05	0.000503405
GO:0001889	liver development	29/3028	5.94E-05	0.000514365
GO:1903035	negative regulation of response to wounding	23/3028	5.99E-05	0.00051722
GO:2001057	reactive nitrogen species metabolic process	23/3028	5.99E-05	0.00051722
GO:0043470	regulation of carbohydrate catabolic process	17/3028	6.42E-05	0.000553508
GO:0007586	digestion	30/3028	6.49E-05	0.000559005
GO:0034637	cellular carbohydrate biosynthetic process	20/3028	6.72E-05	0.000577909
GO:1905037	autophagosome organization	26/3028	6.77E-05	0.000581522
GO:0006686	sphingomyelin biosynthetic process	Jul-28	6.90E-05	0.00058888
GO:0009068	aspartate family amino acid catabolic process	Jul-28	6.90E-05	0.00058888

GO:0009215	purine deoxyribonucleoside triphosphate metabolic process	Jul-28	6.90E-05	0.00058888
GO:0043248	proteasome assembly	Jul-28	6.90E-05	0.00058888
GO:0034614	cellular response to reactive oxygen species	31/3028	7.00E-05	0.000597282
GO:0007029	endoplasmic reticulum organization	23/3028	7.17E-05	0.000610501
GO:0070059	intrinsic apoptotic signaling pathway in response to endoplasmic reticulum stress	18/3028	7.20E-05	0.000612447
GO:0009100	glycoprotein metabolic process	58/3028	7.60E-05	0.000645586
GO:0008210	estrogen metabolic process	Oct-28	7.90E-05	0.000666476
GO:0034383	low-density lipoprotein particle clearance	Oct-28	7.90E-05	0.000666476
GO:0050820	positive regulation of coagulation	Oct-28	7.90E-05	0.000666476
GO:0051788	response to misfolded protein	Oct-28	7.90E-05	0.000666476
GO:0002526	acute inflammatory response	26/3028	7.95E-05	0.000669688
GO:0097035	regulation of membrane lipid distribution	17/3028	8.14E-05	0.000683816
GO:1902475	L-alpha-amino acid transmembrane transport	17/3028	8.14E-05	0.000683816
GO:0045913	positive regulation of carbohydrate metabolic process	21/3028	8.48E-05	0.000711427
GO:0071397	cellular response to cholesterol	Sep-28	8.79E-05	0.000736467
GO:0022600	digestive system process	27/3028	8.88E-05	0.000743319
GO:0008643	carbohydrate transport	33/3028	9.00E-05	0.000752135
GO:0031348	negative regulation of defense response	45/3028	9.69E-05	0.000808976

GO:0030522	intracellular receptor signaling pathway	42/3028	9.75E-05	0.000812498
GO:0010822	positive regulation of mitochondrion organization	20/3028	0.000100242	0.000834061
GO:0051384	response to glucocorticoid	21/3028	0.000102362	0.000850494
GO:0051222	positive regulation of protein transport	56/3028	0.000103956	0.000862512
GO:0005978	glycogen biosynthetic process	14/3028	0.000105166	0.000870081
GO:0009250	glucan biosynthetic process	14/3028	0.000105166	0.000870081
GO:0046467	membrane lipid biosynthetic process	29/3028	0.000105903	0.000874938
GO:0009167	purine ribonucleoside monophosphate metabolic process	13/3028	0.000108133	0.000889581
GO:0045981	positive regulation of nucleotide metabolic process	13/3028	0.000108133	0.000889581
GO:1900544	positive regulation of purine nucleotide metabolic process	13/3028	0.000108133	0.000889581
GO:0003044	regulation of systemic arterial blood pressure mediated by a chemical signal	16/3028	0.000115323	0.000947401
GO:0090151	establishment of protein localization to mitochondrial membrane	Oct-28	0.000119228	0.000978103
GO:0031960	response to corticosteroid	22/3028	0.0001227	0.001003766
GO:0046209	nitric oxide metabolic process	22/3028	0.0001227	0.001003766
GO:1903034	regulation of response to wounding	35/3028	0.000124053	0.00101342
GO:0050873	brown fat cell differentiation	17/3028	0.000128318	0.001046792
GO:0051224	negative regulation of protein transport	30/3028	0.000129796	0.001057378
GO:0002793	positive regulation of peptide secretion	28/3028	0.00013021	0.001059272

GO:2001233	regulation of apoptotic signaling pathway	67/3028	0.000132244	0.00107432
GO:0009084	glutamine family amino acid biosynthetic process	Jul-28	0.000135814	0.001095705
GO:0033240	positive regulation of cellular amine metabolic process	Jul-28	0.000135814	0.001095705
GO:0035999	tetrahydrofolate interconversion	Jul-28	0.000135814	0.001095705
GO:0042761	very long-chain fatty acid biosynthetic process	Jul-28	0.000135814	0.001095705
GO:0046054	dGMP metabolic process	Jul-28	0.000135814	0.001095705
GO:0034655	nucleobase-containing compound catabolic process	66/3028	0.00013779	0.001110111
GO:0009225	nucleotide-sugar metabolic process	Nov-28	0.000138215	0.001110476
GO:0046033	AMP metabolic process	Nov-28	0.000138215	0.001110476
GO:0006662	glycerol ether metabolic process	Sep-28	0.000139463	0.001115892
GO:0051204	protein insertion into mitochondrial membrane	Sep-28	0.000139463	0.001115892
GO:0071071	regulation of phospholipid biosynthetic process	Sep-28	0.000139463	0.001115892
GO:1903580	positive regulation of ATP metabolic process	13/3028	0.000144875	0.001157609
GO:0000422	autophagy of mitochondrion	20/3028	0.000146937	0.001170886
GO:0061726	mitochondrion disassembly	20/3028	0.000146937	0.001170886
GO:0006744	ubiquinone biosynthetic process	Aug-28	0.000149398	0.001180831
GO:0009176	pyrimidine deoxyribonucleoside monophosphate metabolic process	Aug-28	0.000149398	0.001180831
GO:0010918	positive regulation of mitochondrial membrane potential	Aug-28	0.000149398	0.001180831

GO:0019430	removal of superoxide radicals	Aug-28	0.000149398	0.001180831
GO:0032048	cardiolipin metabolic process	Aug-28	0.000149398	0.001180831
GO:1901569	fatty acid derivative catabolic process	Aug-28	0.000149398	0.001180831
GO:0120162	positive regulation of cold-induced thermogenesis	23/3028	0.00016815	0.001327252
GO:0045446	endothelial cell differentiation	26/3028	0.000170228	0.001341837
GO:0043171	peptide catabolic process	Oct-28	0.000175551	0.00137822
GO:0070723	response to cholesterol	Oct-28	0.000175551	0.00137822
GO:0097164	ammonium ion metabolic process	Oct-28	0.000175551	0.00137822
GO:2001242	regulation of intrinsic apoptotic signaling pathway	35/3028	0.000176139	0.001380978
GO:0044058	regulation of digestive system process	16/3028	0.000183139	0.001433935
GO:0090277	positive regulation of peptide hormone secretion	27/3028	0.00018443	0.001442107
GO:0006220	pyrimidine nucleotide metabolic process	13/3028	0.000191863	0.001498226
GO:0008300	isoprenoid catabolic process	Jun-28	0.000193402	0.001500217
GO:0046053	dAMP metabolic process	Jun-28	0.000193402	0.001500217
GO:0046055	dGMP catabolic process	Jun-28	0.000193402	0.001500217
GO:0046130	purine ribonucleoside catabolic process	Jun-28	0.000193402	0.001500217
GO:1904959	regulation of cytochrome-c oxidase activity	Jun-28	0.000193402	0.001500217
GO:0046173	polyol biosynthetic process	17/3028	0.00019744	0.001529504

GO:0097242	amyloid-beta clearance	Dec-28	0.000198006	0.001531862
GO:0045765	regulation of angiogenesis	53/3028	0.000205183	0.001585284
GO:0006684	sphingomyelin metabolic process	Sep-28	0.000214049	0.001647248
GO:0071218	cellular response to misfolded protein	Sep-28	0.000214049	0.001647248
GO:0072529	pyrimidine-containing compound catabolic process	Sep-28	0.000214049	0.001647248
GO:0045444	fat cell differentiation	47/3028	0.000221628	0.00170333
GO:1903829	positive regulation of protein localization	77/3028	0.000233784	0.001794389
GO:0050673	epithelial cell proliferation	75/3028	0.000241814	0.001853587
GO:0035456	response to interferon-beta	17/3028	0.000242799	0.001858704
GO:0035296	regulation of tube diameter	34/3028	0.000243899	0.001859361
GO:0097746	blood vessel diameter maintenance	34/3028	0.000243899	0.001859361
GO:0009200	deoxyribonucleoside triphosphate metabolic process	Aug-28	0.000244159	0.001859361
GO:0042953	lipoprotein transport	Aug-28	0.000244159	0.001859361
GO:0006206	pyrimidine nucleobase metabolic process	Jul-28	0.000246949	0.001863604
GO:0006465	signal peptide processing	Jul-28	0.000246949	0.001863604
GO:0046073	dTMP metabolic process	Jul-28	0.000246949	0.001863604
GO:0090153	regulation of sphingolipid biosynthetic process	Jul-28	0.000246949	0.001863604
GO:0097428	protein maturation by iron-sulfur cluster transfer	Jul-28	0.000246949	0.001863604

GO:1905038	regulation of membrane lipid metabolic process	Jul-28	0.000246949	0.001863604
GO:2001140	positive regulation of phospholipid transport	Jul-28	0.000246949	0.001863604
GO:1904950	negative regulation of establishment of protein localization	30/3028	0.00024774	0.001867159
GO:0007033	vacuole organization	36/3028	0.000249076	0.001874813
GO:0009126	purine nucleoside monophosphate metabolic process	13/3028	0.000251334	0.001889375
GO:0006862	nucleotide transport	Oct-28	0.000252663	0.001896919
GO:0003158	endothelium development	28/3028	0.00025751	0.00193083
GO:0000002	mitochondrial genome maintenance	Dec-28	0.000265208	0.001986003
GO:0035150	regulation of tube size	34/3028	0.000273005	0.002041767
GO:0006900	vesicle budding from membrane	16/3028	0.000283202	0.002115325
GO:1901342	regulation of vasculature development	53/3028	0.000284865	0.002125027
GO:0000305	response to oxygen radical	Sep-28	0.000318967	0.002367324
GO:0009129	pyrimidine nucleoside monophosphate metabolic process	Sep-28	0.000318967	0.002367324
GO:0020027	hemoglobin metabolic process	Sep-28	0.000318967	0.002367324
GO:0042219	cellular modified amino acid catabolic process	Sep-28	0.000318967	0.002367324
GO:0009309	amine biosynthetic process	13/3028	0.000325869	0.002412423
GO:0042401	cellular biogenic amine biosynthetic process	13/3028	0.000325869	0.002412423
GO:0002790	peptide secretion	51/3028	0.000330737	0.002445357

GO:1904951	positive regulation of establishment of protein localization	56/3028	0.000376164	0.002777717
GO:0009251	glucan catabolic process	Aug-28	0.000382959	0.002796078
GO:0030150	protein import into mitochondrial matrix	Aug-28	0.000382959	0.002796078
GO:0042448	progesterone metabolic process	Aug-28	0.000382959	0.002796078
GO:0044872	lipoprotein localization	Aug-28	0.000382959	0.002796078
GO:0045821	positive regulation of glycolytic process	Aug-28	0.000382959	0.002796078
GO:0050746	regulation of lipoprotein metabolic process	Aug-28	0.000382959	0.002796078
GO:0071450	cellular response to oxygen radical	Aug-28	0.000382959	0.002796078
GO:0071451	cellular response to superoxide	Aug-28	0.000382959	0.002796078
GO:1902236	negative regulation of endoplasmic reticulum stress-induced intrinsic apoptotic signaling pathway	Aug-28	0.000382959	0.002796078
GO:0006497	protein lipidation	22/3028	0.000385766	0.002813057
GO:0009133	nucleoside diphosphate biosynthetic process	Jun-28	0.000387547	0.002819004
GO:0009172	purine deoxyribonucleoside monophosphate catabolic process	Jun-28	0.000387547	0.002819004
GO:0006111	regulation of gluconeogenesis	15/3028	0.000406533	0.002953435
GO:0006577	amino-acid betaine metabolic process	Jul-28	0.000421034	0.003032382
GO:0009169	purine ribonucleoside monophosphate catabolic process	Jul-28	0.000421034	0.003032382
GO:0036498	IRE1-mediated unfolded protein response	Jul-28	0.000421034	0.003032382
GO:0046085	adenosine metabolic process	Jul-28	0.000421034	0.003032382

GO:0046322	negative regulation of fatty acid oxidation	Jul-28	0.000421034	0.003032382
GO:0061709	reticulophagy	Jul-28	0.000421034	0.003032382
GO:2001138	regulation of phospholipid transport	Jul-28	0.000421034	0.003032382
GO:0098586	cellular response to virus	18/3028	0.000438133	0.003151643
GO:0050728	negative regulation of inflammatory response	29/3028	0.000442286	0.003177603
GO:1903828	negative regulation of protein localization	40/3028	0.000487305	0.003496739
GO:0009218	pyrimidine ribonucleotide metabolic process	Oct-28	0.000492792	0.003531775
GO:0035458	cellular response to interferon-beta	15/3028	0.000501912	0.00359273
GO:0006584	catecholamine metabolic process	16/3028	0.000520456	0.003711821
GO:0009712	catechol-containing compound metabolic process	16/3028	0.000520456	0.003711821
GO:0010676	positive regulation of cellular carbohydrate metabolic process	16/3028	0.000520456	0.003711821
GO:0071326	cellular response to monosaccharide stimulus	30/3028	0.000569866	0.004056267
GO:0050821	protein stabilization	36/3028	0.000570141	0.004056267
GO:0030072	peptide hormone secretion	49/3028	0.000575065	0.004086323
GO:0006089	lactate metabolic process	Aug-28	0.000579677	0.004104112
GO:0006541	glutamine metabolic process	Aug-28	0.000579677	0.004104112
GO:0050665	hydrogen peroxide biosynthetic process	Aug-28	0.000579677	0.004104112
GO:0046456	icosanoid biosynthetic process	14/3028	0.00058406	0.004130137

GO:2001234	negative regulation of apoptotic signaling pathway	43/3028	0.000593602	0.004192542
GO:1903050	regulation of proteolysis involved in cellular protein catabolic process	39/3028	0.000598474	0.004221841
GO:0050727	regulation of inflammatory response	57/3028	0.000602396	0.004244386
GO:0002697	regulation of immune effector process	68/3028	0.0006085	0.004282228
GO:0006487	protein N-linked glycosylation	16/3028	0.000630419	0.004431137
GO:0046513	ceramide biosynthetic process	17/3028	0.000631242	0.004431589
GO:0071456	cellular response to hypoxia	23/3028	0.000646809	0.0045354 3
GO:0046037	GMP metabolic process	Sep-28	0.000656326	0.004596646
GO:0098657	import into cell	42/3028	0.000665844	0.004657721
GO:0032369	negative regulation of lipid transport	Oct-28	0.0006700 8	0.004670569
GO:0039528	cytoplasmic pattern recognition receptor signaling pathway in response to virus	Oct-28	0.0006700 8	0.004670569
GO:0042744	hydrogen peroxide catabolic process	Oct-28	0.0006700 8	0.004670569
GO:0042417	dopamine metabolic process	13/3028	0.000671415	0.004674299
GO:0009128	purine nucleoside monophosphate catabolic process	Jul-28	0.000680741	0.004716734
GO:0019184	nonribosomal peptide biosynthetic process	Jul-28	0.000680741	0.004716734
GO:0044804	autophagy of nucleus	Jul-28	0.000680741	0.004716734
GO:1902001	fatty acid transmembrane transport	Jul-28	0.000680741	0.004716734
GO:2001243	negative regulation of intrinsic apoptotic signaling pathway	22/3028	0.000694555	0.004806747

GO:0071453	cellular response to oxygen levels	29/3028	0.000705196	0.004807141
GO:0001867	complement activation, lectin pathway	Jun-28	0.000706134	0.004807141
GO:0006000	fructose metabolic process	Jun-28	0.000706134	0.004807141
GO:0006123	mitochondrial electron transport, cytochrome c to oxygen	Jun-28	0.000706134	0.004807141
GO:0006213	pyrimidine nucleoside metabolic process	Jun-28	0.000706134	0.004807141
GO:0009223	pyrimidine deoxyribonucleotide catabolic process	Jun-28	0.000706134	0.004807141
GO:0033212	iron import into cell	Jun-28	0.000706134	0.004807141
GO:0034310	primary alcohol catabolic process	Jun-28	0.000706134	0.004807141
GO:0035437	maintenance of protein localization in endoplasmic reticulum	Jun-28	0.000706134	0.004807141
GO:0046689	response to mercury ion	Jun-28	0.000706134	0.004807141
GO:0048312	intracellular distribution of mitochondria	Jun-28	0.000706134	0.004807141
GO:0070863	positive regulation of protein exit from endoplasmic reticulum	Jun-28	0.000706134	0.004807141
GO:1904152	regulation of retrograde protein transport, ER to cytosol	Jun-28	0.000706134	0.004807141
GO:2000303	regulation of ceramide biosynthetic process	Jun-28	0.000706134	0.004807141
GO:0061028	establishment of endothelial barrier	14/3028	0.000722836	0.004915114
GO:0043030	regulation of macrophage activation	15/3028	0.000750861	0.005099746
GO:0006636	unsaturated fatty acid biosynthetic process	Dec-28	0.000759225	0.005147296
GO:0033692	cellular polysaccharide biosynthetic process	16/3028	0.000759625	0.005147296

GO:0061136	regulation of proteasomal protein catabolic process	34/3028	0.000784247	0.005307983
GO:0001678	cellular glucose homeostasis	32/3028	0.000795934	0.005380844
GO:0034219	carbohydrate transmembrane transport	26/3028	0.000839353	0.005658084
GO:0006110	regulation of glycolytic process	13/3028	0.000839849	0.005658084
GO:0072348	sulfur compound transport	13/3028	0.000839849	0.005658084
GO:0043255	regulation of carbohydrate biosynthetic process	23/3028	0.000847718	0.005704515
GO:0030194	positive regulation of blood coagulation	Aug-28	0.000850578	0.005710604
GO:1900048	positive regulation of hemostasis	Aug-28	0.000850578	0.005710604
GO:0009755	hormone-mediated signaling pathway	31/3028	0.000885981	0.005941461
GO:0051923	sulfation	Oct-28	0.000896794	0.006007077
GO:0039529	RIG-I signaling pathway	Sep-28	0.000910854	0.006080344
GO:0045838	positive regulation of membrane potential	Sep-28	0.000910854	0.006080344
GO:0050687	negative regulation of defense response to virus	Sep-28	0.000910854	0.006080344
GO:0001818	negative regulation of cytokine production	49/3028	0.00091408	0.006094912
GO:0050708	regulation of protein secretion	50/3028	0.000944524	0.006290721
GO:0070085	glycosylation	38/3028	0.000965037	0.006420023
GO:0006809	nitric oxide biosynthetic process	19/3028	0.000968537	0.006428665
GO:0007568	aging	19/3028	0.000968537	0.006428665

GO:0050714	positive regulation of protein secretion	31/3028	0.000983128	0.006518105
GO:0044070	regulation of anion transport	24/3028	0.001007924	0.006674928
GO:2000379	positive regulation of reactive oxygen species metabolic process	18/3028	0.001017601	0.006731379
GO:0070131	positive regulation of mitochondrial translation	Jul-28	0.001052681	0.006955551
GO:0001666	response to hypoxia	38/3028	0.001054663	0.006960777
GO:0032024	positive regulation of insulin secretion	20/3028	0.001057853	0.006966085
GO:1900407	regulation of cellular response to oxidative stress	20/3028	0.001057853	0.006966085
GO:0070873	regulation of glycogen metabolic process	Nov-28	0.001081787	0.007115674
GO:0071331	cellular response to hexose stimulus	29/3028	0.001095923	0.007200547
GO:0002791	regulation of peptide secretion	41/3028	0.001142658	0.007499177
GO:0006734	NADH metabolic process	Oct-28	0.001182757	0.007744938
GO:1905898	positive regulation of response to endoplasmic reticulum stress	Oct-28	0.001182757	0.007744938
GO:0000255	allantoin metabolic process	Jun-28	0.001194979	0.007781352
GO:0009437	carnitine metabolic process	Jun-28	0.001194979	0.007781352
GO:0034374	low-density lipoprotein particle remodeling	Jun-28	0.001194979	0.007781352
GO:1901142	insulin metabolic process	Jun-28	0.001194979	0.007781352
GO:1901678	iron coordination entity transport	Jun-28	0.001194979	0.007781352
GO:0042181	ketone biosynthetic process	Dec-28	0.001208772	0.007845991

GO:0048771	tissue remodeling	36/3028	0.001213882	0.007845991
GO:0000303	response to superoxide	Aug-28	0.001214309	0.007845991
GO:0015865	purine nucleotide transport	Aug-28	0.001214309	0.007845991
GO:0034312	diol biosynthetic process	Aug-28	0.001214309	0.007845991
GO:0071398	cellular response to fatty acid	Aug-28	0.001214309	0.007845991
GO:0150104	transport across blood-brain barrier	Aug-28	0.001214309	0.007845991
GO:0070584	mitochondrion morphogenesis	Sep-28	0.00123995	0.008002817
GO:0043124	negative regulation of I-kappaB kinase/NF-kappaB signaling	13/3028	0.001284	0.008268845
GO:0089718	amino acid import across plasma membrane	13/3028	0.001284	0.008268845
GO:0046474	glycerophospholipid biosynthetic process	31/3028	0.00133245	0.008571413
GO:0071322	cellular response to carbohydrate stimulus	30/3028	0.001345632	0.008646684
GO:0032692	negative regulation of interleukin-1 production	Nov-28	0.001379589	0.008855142
GO:0050796	regulation of insulin secretion	34/3028	0.001393151	0.008930934
GO:0090276	regulation of peptide hormone secretion	40/3028	0.001394455	0.008930934
GO:0090087	regulation of peptide transport	41/3028	0.001459797	0.009339184
GO:1902882	regulation of response to oxidative stress	21/3028	0.00148486	0.009489132
GO:0046326	positive regulation of glucose import	Dec-28	0.001505154	0.009608311
GO:0032890	regulation of organic acid transport	19/3028	0.001508729	0.009620616

GO:0050999	regulation of nitric-oxide synthase activity	Oct-28	0.001538901	0.009802312
GO:0002921	negative regulation of humoral immune response	Jul-28	0.001567195	0.009928399
GO:0005980	glycogen catabolic process	Jul-28	0.001567195	0.009928399
GO:0016137	glycoside metabolic process	Jul-28	0.001567195	0.009928399
GO:1901570	fatty acid derivative biosynthetic process	Jul-28	0.001567195	0.009928399
GO:1903727	positive regulation of phospholipid metabolic process	Jul-28	0.001567195	0.009928399

GO terms associated with 2355 Rep_early_enriched_vs_Hep genes

ID	Description	GeneRatio	pvalue	p.adjust
GO:0030198	extracellular matrix organization	94/1966	1.36E-35	4.77E-32
GO:0043062	extracellular structure organization	94/1966	1.82E-35	4.77E-32
GO:0045229	external encapsulating structure organization	94/1966	2.42E-35	4.77E-32
GO:0031589	cell-substrate adhesion	93/1966	1.50E-28	2.23E-25
GO:0000280	nuclear division	96/1966	2.52E-24	2.99E-21
GO:0140014	mitotic nuclear division	72/1966	3.85E-23	3.80E-20
GO:0048285	organelle fission	99/1966	2.96E-22	2.50E-19
GO:0007059	chromosome segregation	77/1966	4.25E-21	3.15E-18
GO:0007015	actin filament organization	91/1966	1.54E-20	1.01E-17
GO:0001667	ameboidal-type cell migration	86/1966	3.04E-18	1.80E-15
GO:0007178	transmembrane receptor protein serine/threonine kinase signaling pathway	78/1966	6.78E-18	3.65E-15
GO:0033674	positive regulation of kinase activity	83/1966	9.92E-18	4.89E-15
GO:0007346	regulation of mitotic cell cycle	88/1966	1.22E-17	5.55E-15
GO:0098813	nuclear chromosome segregation	63/1966	1.32E-17	5.57E-15
GO:0045785	positive regulation of cell adhesion	88/1966	1.82E-17	7.20E-15
GO:0000070	mitotic sister chromatid segregation	46/1966	2.81E-17	1.04E-14

GO:0030199	collagen fibril organization	27/1966	1.58E-16	5.49E-14
GO:0060485	mesenchyme development	64/1966	1.77E-16	5.81E-14
GO:0007409	axonogenesis	87/1966	2.38E-16	7.41E-14
GO:0007160	cell-matrix adhesion	55/1966	3.26E-16	9.67E-14
GO:0048762	mesenchymal cell differentiation	56/1966	3.68E-16	1.04E-13
GO:0007264	small GTPase mediated signal transduction	79/1966	6.50E-16	1.70E-13
GO:0032970	regulation of actin filament-based process	77/1966	6.62E-16	1.70E-13
GO:0042060	wound healing	72/1966	9.05E-16	2.23E-13
GO:0045860	positive regulation of protein kinase activity	70/1966	9.92E-16	2.31E-13
GO:0031346	positive regulation of cell projection organization	80/1966	1.02E-15	2.31E-13
GO:0000819	sister chromatid segregation	48/1966	1.24E-15	2.71E-13
GO:0061448	connective tissue development	62/1966	1.30E-15	2.75E-13
GO:0001655	urogenital system development	73/1966	1.75E-15	3.58E-13
GO:0045787	positive regulation of cell cycle	69/1966	2.18E-15	4.30E-13
GO:0032956	regulation of actin cytoskeleton organization	70/1966	3.21E-15	6.14E-13
GO:0090092	regulation of transmembrane receptor protein serine/threonine kinase signaling pathway	56/1966	5.03E-15	9.26E-13
GO:0085029	extracellular matrix assembly	22/1966	5.17E-15	9.26E-13
GO:0060562	epithelial tube morphogenesis	73/1966	5.32E-15	9.26E-13

GO:0010810	regulation of cell-substrate adhesion	52/1966	6.46E-15	1.09E-12
GO:0018108	peptidyl-tyrosine phosphorylation	64/1966	1.02E-14	1.68E-12
GO:0090287	regulation of cellular response to growth factor stimulus	61/1966	1.09E-14	1.74E-12
GO:0007265	Ras protein signal transduction	66/1966	1.27E-14	1.97E-12
GO:0043254	regulation of protein-containing complex assembly	76/1966	1.52E-14	2.31E-12
GO:0018212	peptidyl-tyrosine modification	64/1966	1.60E-14	2.37E-12
GO:1902903	regulation of supramolecular fiber organization	71/1966	2.11E-14	3.04E-12
GO:0001763	morphogenesis of a branching structure	54/1966	2.25E-14	3.12E-12
GO:0034329	cell junction assembly	77/1966	2.27E-14	3.12E-12
GO:0010634	positive regulation of epithelial cell migration	41/1966	3.43E-14	4.61E-12
GO:0050767	regulation of neurogenesis	78/1966	4.22E-14	5.48E-12
GO:0090130	tissue migration	62/1966	4.25E-14	5.48E-12
GO:0044772	mitotic cell cycle phase transition	74/1966	4.52E-14	5.58E-12
GO:0032963	collagen metabolic process	36/1966	4.52E-14	5.58E-12
GO:0061138	morphogenesis of a branching epithelium	51/1966	4.66E-14	5.63E-12
GO:0007179	transforming growth factor beta receptor signaling pathway	45/1966	5.21E-14	6.17E-12
GO:1901342	regulation of vasculature development	61/1966	5.96E-14	6.93E-12
GO:0022604	regulation of cell morphogenesis	64/1966	7.97E-14	8.99E-12

GO:0010631	epithelial cell migration	61/1966	8.04E-14	8.99E-12
GO:0071560	cellular response to transforming growth factor beta stimulus	50/1966	9.04E-14	9.92E-12
GO:0090132	epithelium migration	61/1966	1.08E-13	1.16E-11
GO:0070371	ERK1 and ERK2 cascade	65/1966	1.14E-13	1.20E-11
GO:1902850	microtubule cytoskeleton organization involved in mitosis	39/1966	1.31E-13	1.36E-11
GO:0070372	regulation of ERK1 and ERK2 cascade	62/1966	1.59E-13	1.62E-11
GO:0071559	response to transforming growth factor beta	50/1966	1.85E-13	1.86E-11
GO:0072001	renal system development	64/1966	2.12E-13	2.09E-11
GO:0010632	regulation of epithelial cell migration	51/1966	3.23E-13	3.14E-11
GO:0045765	regulation of angiogenesis	59/1966	3.84E-13	3.66E-11
GO:0007411	axon guidance	53/1966	3.90E-13	3.66E-11
GO:0097485	neuron projection guidance	53/1966	4.58E-13	4.24E-11
GO:0032964	collagen biosynthetic process	23/1966	7.39E-13	6.73E-11
GO:0001822	kidney development	61/1966	9.07E-13	8.14E-11
GO:0007266	Rho protein signal transduction	36/1966	1.56E-12	1.38E-10
GO:0090068	positive regulation of cell cycle process	53/1966	1.61E-12	1.40E-10
GO:0051495	positive regulation of cytoskeleton organization	45/1966	2.06E-12	1.77E-10
GO:1902905	positive regulation of supramolecular fiber organization	42/1966	2.36E-12	2.00E-10

GO:0010959	regulation of metal ion transport	73/1966	2.84E-12	2.37E-10
GO:0007051	spindle organization	42/1966	3.46E-12	2.82E-10
GO:0007052	mitotic spindle organization	33/1966	3.48E-12	2.82E-10
GO:0051056	regulation of small GTPase mediated signal transduction	48/1966	4.44E-12	3.56E-10
GO:0090101	negative regulation of transmembrane receptor protein serine/threonine kinase signaling pathway	34/1966	4.72E-12	3.72E-10
GO:0071900	regulation of protein serine/threonine kinase activity	62/1966	5.20E-12	4.05E-10
GO:0043087	regulation of GTPase activity	61/1966	6.52E-12	5.02E-10
GO:1901990	regulation of mitotic cell cycle phase transition	57/1966	7.20E-12	5.46E-10
GO:1903034	regulation of response to wounding	40/1966	7.38E-12	5.53E-10
GO:1901987	regulation of cell cycle phase transition	67/1966	8.68E-12	6.42E-10
GO:0051962	positive regulation of nervous system development	62/1966	1.24E-11	9.04E-10
GO:0048754	branching morphogenesis of an epithelial tube	42/1966	1.25E-11	9.04E-10
GO:0051983	regulation of chromosome segregation	28/1966	1.47E-11	1.05E-09
GO:0046578	regulation of Ras protein signal transduction	43/1966	1.56E-11	1.10E-09
GO:1903829	positive regulation of protein localization	76/1966	1.69E-11	1.18E-09
GO:0051090	regulation of DNA-binding transcription factor activity	70/1966	1.97E-11	1.36E-09
GO:0051656	establishment of organelle localization	69/1966	2.04E-11	1.38E-09
GO:0051258	protein polymerization	52/1966	2.05E-11	1.38E-09

GO:0010639	negative regulation of organelle organization	63/1966	2.26E-11	1.50E-09
GO:0051017	actin filament bundle assembly	39/1966	2.54E-11	1.67E-09
GO:0032535	regulation of cellular component size	65/1966	2.79E-11	1.81E-09
GO:0001503	ossification	68/1966	2.90E-11	1.87E-09
GO:1903046	meiotic cell cycle process	45/1966	3.10E-11	1.96E-09
GO:0051216	cartilage development	44/1966	3.11E-11	1.96E-09
GO:0040013	negative regulation of locomotion	59/1966	3.16E-11	1.97E-09
GO:0045930	negative regulation of mitotic cell cycle	46/1966	3.58E-11	2.18E-09
GO:0050920	regulation of chemotaxis	46/1966	3.58E-11	2.18E-09
GO:0061572	actin filament bundle organization	39/1966	4.43E-11	2.67E-09
GO:0050900	leukocyte migration	63/1966	5.06E-11	3.03E-09
GO:0050730	regulation of peptidyl-tyrosine phosphorylation	50/1966	5.40E-11	3.20E-09
GO:0010811	positive regulation of cell-substrate adhesion	34/1966	5.49E-11	3.22E-09
GO:0048608	reproductive structure development	74/1966	5.63E-11	3.26E-09
GO:0140694	non-membrane-bounded organelle assembly	63/1966	5.66E-11	3.26E-09
GO:0001837	epithelial to mesenchymal transition	36/1966	5.95E-11	3.39E-09
GO:0050673	epithelial cell proliferation	73/1966	6.57E-11	3.71E-09
GO:0110053	regulation of actin filament organization	51/1966	6.65E-11	3.71E-09

GO:1901991	negative regulation of mitotic cell cycle phase transition	38/1966	7.15E-11	3.96E-09
GO:0030512	negative regulation of transforming growth factor beta receptor signaling pathway	24/1966	7.77E-11	4.26E-09
GO:0002685	regulation of leukocyte migration	45/1966	7.93E-11	4.31E-09
GO:0061458	reproductive system development	74/1966	8.31E-11	4.47E-09
GO:0002687	positive regulation of leukocyte migration	36/1966	8.77E-11	4.64E-09
GO:0008360	regulation of cell shape	36/1966	8.77E-11	4.64E-09
GO:0050727	regulation of inflammatory response	59/1966	1.18E-10	6.17E-09
GO:0048251	elastic fiber assembly	Oct-66	1.24E-10	6.46E-09
GO:0050808	synapse organization	74/1966	1.47E-10	7.60E-09
GO:0042692	muscle cell differentiation	68/1966	1.72E-10	8.77E-09
GO:0003007	heart morphogenesis	51/1966	1.93E-10	9.77E-09
GO:0007162	negative regulation of cell adhesion	54/1966	2.67E-10	1.34E-08
GO:0010720	positive regulation of cell development	61/1966	3.11E-10	1.55E-08
GO:0060537	muscle tissue development	73/1966	3.34E-10	1.65E-08
GO:0050769	positive regulation of neurogenesis	52/1966	3.75E-10	1.83E-08
GO:0051304	chromosome separation	27/1966	4.02E-10	1.94E-08
GO:0060560	developmental growth involved in morphogenesis	50/1966	4.04E-10	1.94E-08
GO:0017015	regulation of transforming growth factor beta receptor signaling pathway	30/1966	4.26E-10	2.03E-08

GO:0051783	regulation of nuclear division	33/1966	4.41E-10	2.09E-08
GO:0022407	regulation of cell-cell adhesion	70/1966	4.93E-10	2.32E-08
GO:0034765	regulation of ion transmembrane transport	73/1966	5.27E-10	2.46E-08
GO:0071674	mononuclear cell migration	38/1966	5.75E-10	2.66E-08
GO:0045216	cell-cell junction organization	42/1966	6.24E-10	2.87E-08
GO:0050731	positive regulation of peptidyl-tyrosine phosphorylation	39/1966	6.76E-10	3.08E-08
GO:0051310	metaphase plate congression	21/1966	6.96E-10	3.15E-08
GO:0050678	regulation of epithelial cell proliferation	63/1966	7.38E-10	3.30E-08
GO:0048588	developmental cell growth	49/1966	7.41E-10	3.30E-08
GO:1903844	regulation of cellular response to transforming growth factor beta stimulus	30/1966	8.06E-10	3.56E-08
GO:0030177	positive regulation of Wnt signaling pathway	32/1966	8.61E-10	3.78E-08
GO:0140013	meiotic nuclear division	40/1966	9.05E-10	3.94E-08
GO:0032965	regulation of collagen biosynthetic process	18/1966	9.62E-10	4.16E-08
GO:0001570	vasculogenesis	26/1966	1.00E-09	4.30E-08
GO:0048732	gland development	70/1966	1.04E-09	4.41E-08
GO:0043542	endothelial cell migration	43/1966	1.06E-09	4.47E-08
GO:0071772	response to BMP	38/1966	1.10E-09	4.59E-08
GO:0071773	cellular response to BMP stimulus	38/1966	1.10E-09	4.59E-08

GO:0061041	regulation of wound healing	31/1966	1.14E-09	4.71E-08
GO:0003012	muscle system process	65/1966	1.32E-09	5.43E-08
GO:0022612	gland morphogenesis	34/1966	1.44E-09	5.89E-08
GO:0030323	respiratory tube development	44/1966	1.49E-09	6.06E-08
GO:0001558	regulation of cell growth	68/1966	1.57E-09	6.34E-08
GO:1901343	negative regulation of vasculature development	28/1966	1.63E-09	6.54E-08
GO:0010712	regulation of collagen metabolic process	19/1966	1.67E-09	6.64E-08
GO:0008154	actin polymerization or depolymerization	39/1966	1.72E-09	6.77E-08
GO:0050921	positive regulation of chemotaxis	33/1966	1.96E-09	7.68E-08
GO:0007611	learning or memory	53/1966	2.19E-09	8.55E-08
GO:0001952	regulation of cell-matrix adhesion	30/1966	2.22E-09	8.59E-08
GO:0042063	gliogenesis	56/1966	2.26E-09	8.70E-08
GO:0051271	negative regulation of cellular component movement	52/1966	2.50E-09	9.54E-08
GO:0032271	regulation of protein polymerization	40/1966	2.56E-09	9.73E-08
GO:0003018	vascular process in circulatory system	43/1966	2.77E-09	1.05E-07
GO:0070997	neuron death	65/1966	2.81E-09	1.05E-07
GO:0030041	actin filament polymerization	35/1966	2.92E-09	1.08E-07
GO:0032103	positive regulation of response to external stimulus	67/1966	2.93E-09	1.08E-07

GO:0030324	lung development	43/1966	3.17E-09	1.16E-07
GO:0010717	regulation of epithelial to mesenchymal transition	25/1966	3.17E-09	1.16E-07
GO:0010595	positive regulation of endothelial cell migration	27/1966	3.20E-09	1.16E-07
GO:1903706	regulation of hemopoiesis	61/1966	3.63E-09	1.31E-07
GO:0062197	cellular response to chemical stress	54/1966	3.70E-09	1.33E-07
GO:0016055	Wnt signaling pathway	66/1966	3.78E-09	1.35E-07
GO:0007088	regulation of mitotic nuclear division	27/1966	3.97E-09	1.40E-07
GO:1905818	regulation of chromosome separation	22/1966	3.98E-09	1.40E-07
GO:0071604	transforming growth factor beta production	16/1966	4.16E-09	1.46E-07
GO:1901988	negative regulation of cell cycle phase transition	44/1966	4.29E-09	1.50E-07
GO:0198738	cell-cell signaling by wnt	66/1966	4.52E-09	1.57E-07
GO:0006814	sodium ion transport	44/1966	4.88E-09	1.68E-07
GO:0098742	cell-cell adhesion via plasma-membrane adhesion molecules	40/1966	5.21E-09	1.78E-07
GO:0031334	positive regulation of protein-containing complex assembly	39/1966	5.55E-09	1.89E-07
GO:0060541	respiratory system development	46/1966	5.76E-09	1.95E-07
GO:0050000	chromosome localization	23/1966	5.83E-09	1.96E-07
GO:2000181	negative regulation of blood vessel morphogenesis	27/1966	6.05E-09	2.02E-07
GO:0035023	regulation of Rho protein signal transduction	24/1966	6.21E-09	2.06E-07

GO:0010594	regulation of endothelial cell migration	35/1966	6.51E-09	2.15E-07
GO:0010934	macrophage cytokine production	Nov-66	6.58E-09	2.16E-07
GO:0001818	negative regulation of cytokine production	49/1966	7.22E-09	2.36E-07
GO:0010976	positive regulation of neuron projection development	41/1966	7.27E-09	2.37E-07
GO:0043491	protein kinase B signaling	39/1966	7.36E-09	2.38E-07
GO:0044839	cell cycle G2/M phase transition	32/1966	7.60E-09	2.45E-07
GO:0043405	regulation of MAP kinase activity	38/1966	7.82E-09	2.50E-07
GO:0000086	G2/M transition of mitotic cell cycle	30/1966	8.32E-09	2.63E-07
GO:0007093	mitotic cell cycle checkpoint signaling	30/1966	8.32E-09	2.63E-07
GO:1904019	epithelial cell apoptotic process	28/1966	8.46E-09	2.67E-07
GO:0009314	response to radiation	63/1966	8.88E-09	2.77E-07
GO:0030509	BMP signaling pathway	35/1966	8.88E-09	2.77E-07
GO:0048638	regulation of developmental growth	60/1966	9.12E-09	2.83E-07
GO:0070374	positive regulation of ERK1 and ERK2 cascade	41/1966	9.48E-09	2.92E-07
GO:0061640	cytoskeleton-dependent cytokinesis	24/1966	9.91E-09	3.02E-07
GO:1904035	regulation of epithelial cell apoptotic process	24/1966	9.91E-09	3.02E-07
GO:0032102	negative regulation of response to external stimulus	60/1966	1.00E-08	3.04E-07
GO:0030866	cortical actin cytoskeleton organization	15/1966	1.06E-08	3.19E-07

GO:0030010	establishment of cell polarity	32/1966	1.07E-08	3.20E-07
GO:0002062	chondrocyte differentiation	27/1966	1.11E-08	3.33E-07
GO:0050890	cognition	55/1966	1.50E-08	4.46E-07
GO:0048659	smooth muscle cell proliferation	36/1966	1.55E-08	4.59E-07
GO:0043588	skin development	51/1966	1.63E-08	4.81E-07
GO:1990778	protein localization to cell periphery	56/1966	1.72E-08	5.04E-07
GO:0016525	negative regulation of angiogenesis	26/1966	1.78E-08	5.21E-07
GO:0051303	establishment of chromosome localization	22/1966	1.87E-08	5.43E-07
GO:0033002	muscle cell proliferation	44/1966	1.89E-08	5.45E-07
GO:0031345	negative regulation of cell projection organization	39/1966	1.91E-08	5.48E-07
GO:2000146	negative regulation of cell motility	49/1966	1.95E-08	5.58E-07
GO:0045786	negative regulation of cell cycle	56/1966	2.08E-08	5.93E-07
GO:1901214	regulation of neuron death	58/1966	2.20E-08	6.24E-07
GO:0046847	filopodium assembly	21/1966	2.22E-08	6.27E-07
GO:0030510	regulation of BMP signaling pathway	25/1966	2.32E-08	6.52E-07
GO:0050679	positive regulation of epithelial cell proliferation	39/1966	2.48E-08	6.93E-07
GO:0021700	developmental maturation	54/1966	2.59E-08	7.19E-07
GO:0048144	fibroblast proliferation	26/1966	2.66E-08	7.35E-07

GO:0010935	regulation of macrophage cytokine production	Oct-66	2.67E-08	7.35E-07
GO:0007517	muscle organ development	55/1966	2.68E-08	7.35E-07
GO:0030111	regulation of Wnt signaling pathway	50/1966	2.86E-08	7.81E-07
GO:0014812	muscle cell migration	27/1966	2.93E-08	7.95E-07
GO:0051346	negative regulation of hydrolase activity	55/1966	2.95E-08	7.98E-07
GO:0001656	metanephros development	23/1966	3.06E-08	8.24E-07
GO:0045807	positive regulation of endocytosis	28/1966	3.13E-08	8.39E-07
GO:0048146	positive regulation of fibroblast proliferation	20/1966	3.35E-08	8.94E-07
GO:0030100	regulation of endocytosis	41/1966	3.37E-08	8.94E-07
GO:0014909	smooth muscle cell migration	25/1966	3.49E-08	9.24E-07
GO:0030336	negative regulation of cell migration	47/1966	3.55E-08	9.34E-07
GO:1901888	regulation of cell junction assembly	39/1966	3.65E-08	9.57E-07
GO:0030048	actin filament-based movement	28/1966	3.74E-08	9.76E-07
GO:0048771	tissue remodeling	37/1966	3.84E-08	9.97E-07
GO:0031532	actin cytoskeleton reorganization	26/1966	3.91E-08	1.01E-06
GO:0032967	positive regulation of collagen biosynthetic process	13/1966	4.30E-08	1.11E-06
GO:0061035	regulation of cartilage development	20/1966	4.34E-08	1.11E-06
GO:0060070	canonical Wnt signaling pathway	48/1966	4.71E-08	1.20E-06

GO:0001764	neuron migration	36/1966	4.75E-08	1.21E-06
GO:0006936	muscle contraction	49/1966	5.00E-08	1.27E-06
GO:0051383	kinetochore organization	Nov-66	5.07E-08	1.28E-06
GO:0051924	regulation of calcium ion transport	46/1966	5.63E-08	1.41E-06
GO:0051145	smooth muscle cell differentiation	21/1966	5.93E-08	1.48E-06
GO:0031099	regeneration	31/1966	6.25E-08	1.56E-06
GO:1901215	negative regulation of neuron death	43/1966	6.77E-08	1.67E-06
GO:0016358	dendrite development	49/1966	6.77E-08	1.67E-06
GO:0010948	negative regulation of cell cycle process	46/1966	6.96E-08	1.71E-06
GO:0008064	regulation of actin polymerization or depolymerization	32/1966	7.02E-08	1.72E-06
GO:0072006	nephron development	31/1966	7.29E-08	1.78E-06
GO:0002011	morphogenesis of an epithelial sheet	18/1966	7.34E-08	1.78E-06
GO:0014706	striated muscle tissue development	46/1966	7.74E-08	1.87E-06
GO:0003205	cardiac chamber development	36/1966	8.10E-08	1.95E-06
GO:0071675	regulation of mononuclear cell migration	26/1966	8.23E-08	1.97E-06
GO:0045931	positive regulation of mitotic cell cycle	28/1966	8.83E-08	2.11E-06
GO:0071634	regulation of transforming growth factor beta production	14/1966	9.19E-08	2.19E-06
GO:0051961	negative regulation of nervous system development	32/1966	9.41E-08	2.23E-06

GO:0045444	fat cell differentiation	43/1966	9.45E-08	2.23E-06
GO:0007224	smoothened signaling pathway	31/1966	9.86E-08	2.32E-06
GO:0006979	response to oxidative stress	58/1966	9.96E-08	2.33E-06
GO:0090288	negative regulation of cellular response to growth factor stimulus	24/1966	1.02E-07	2.37E-06
GO:0051896	regulation of protein kinase B signaling	33/1966	1.02E-07	2.38E-06
GO:0050919	negative chemotaxis	15/1966	1.06E-07	2.46E-06
GO:0006869	lipid transport	56/1966	1.08E-07	2.48E-06
GO:0030832	regulation of actin filament length	32/1966	1.09E-07	2.50E-06
GO:0007163	establishment or maintenance of cell polarity	39/1966	1.11E-07	2.53E-06
GO:1904062	regulation of cation transmembrane transport	55/1966	1.17E-07	2.66E-06
GO:0043271	negative regulation of ion transport	33/1966	1.18E-07	2.66E-06
GO:0001654	eye development	59/1966	1.18E-07	2.66E-06
GO:0003206	cardiac chamber morphogenesis	30/1966	1.19E-07	2.66E-06
GO:0031333	negative regulation of protein-containing complex assembly	30/1966	1.19E-07	2.66E-06
GO:0030865	cortical cytoskeleton organization	18/1966	1.27E-07	2.84E-06
GO:0007613	memory	31/1966	1.32E-07	2.94E-06
GO:0070507	regulation of microtubule cytoskeleton organization	31/1966	1.32E-07	2.94E-06
GO:0007229	integrin-mediated signaling pathway	23/1966	1.34E-07	2.96E-06

GO:1990138	neuron projection extension	36/1966	1.35E-07	2.98E-06
GO:0031032	actomyosin structure organization	37/1966	1.38E-07	3.04E-06
GO:0051321	meiotic cell cycle	50/1966	1.39E-07	3.04E-06
GO:0150063	visual system development	59/1966	1.51E-07	3.28E-06
GO:0010977	negative regulation of neuron projection development	31/1966	1.53E-07	3.32E-06
GO:0051984	positive regulation of chromosome segregation	Nov-66	1.58E-07	3.41E-06
GO:0010714	positive regulation of collagen metabolic process	13/1966	1.59E-07	3.43E-06
GO:0008347	glial cell migration	18/1966	1.66E-07	3.55E-06
GO:0030833	regulation of actin filament polymerization	29/1966	1.66E-07	3.55E-06
GO:0010721	negative regulation of cell development	36/1966	1.74E-07	3.71E-06
GO:0097191	extrinsic apoptotic signaling pathway	40/1966	1.75E-07	3.72E-06
GO:0050768	negative regulation of neurogenesis	31/1966	1.77E-07	3.73E-06
GO:0048660	regulation of smooth muscle cell proliferation	33/1966	1.77E-07	3.73E-06
GO:0007018	microtubule-based movement	59/1966	1.78E-07	3.73E-06
GO:0072697	protein localization to cell cortex	Aug-66	1.79E-07	3.74E-06
GO:0048661	positive regulation of smooth muscle cell proliferation	24/1966	1.80E-07	3.75E-06
GO:0010563	negative regulation of phosphorus metabolic process	62/1966	1.84E-07	3.80E-06
GO:0045936	negative regulation of phosphate metabolic process	62/1966	1.84E-07	3.80E-06

GO:0060249	anatomical structure homeostasis	49/1966	1.97E-07	4.07E-06
GO:0010389	regulation of G2/M transition of mitotic cell cycle	23/1966	1.99E-07	4.09E-06
GO:0001666	response to hypoxia	37/1966	2.00E-07	4.09E-06
GO:0048880	sensory system development	59/1966	2.09E-07	4.26E-06
GO:0090263	positive regulation of canonical Wnt signaling pathway	24/1966	2.16E-07	4.40E-06
GO:0008608	attachment of spindle microtubules to kinetochore	13/1966	2.38E-07	4.82E-06
GO:0009914	hormone transport	54/1966	2.54E-07	5.12E-06
GO:0006310	DNA recombination	48/1966	2.54E-07	5.12E-06
GO:0048145	regulation of fibroblast proliferation	24/1966	2.60E-07	5.21E-06
GO:0072659	protein localization to plasma membrane	46/1966	2.61E-07	5.22E-06
GO:0045766	positive regulation of angiogenesis	33/1966	2.64E-07	5.25E-06
GO:1904018	positive regulation of vasculature development	33/1966	2.64E-07	5.25E-06
GO:0034508	centromere complex assembly	Dec-66	2.67E-07	5.30E-06
GO:0051225	spindle assembly	25/1966	2.72E-07	5.36E-06
GO:0034599	cellular response to oxidative stress	43/1966	2.75E-07	5.40E-06
GO:0045926	negative regulation of growth	42/1966	2.86E-07	5.61E-06
GO:0048678	response to axon injury	19/1966	2.90E-07	5.66E-06
GO:0034446	substrate adhesion-dependent cell spreading	24/1966	3.11E-07	6.05E-06

GO:004 8675	axon extension	28/196 6	3.16E-07	6.13E-06
GO:001 4910	regulation of smooth muscle cell migration	22/196 6	3.20E-07	6.20E-06
GO:003 5924	cellular response to vascular endothelial growth factor stimulus	17/196 6	3.23E-07	6.23E-06
GO:190 3532	positive regulation of secretion by cell	52/196 6	3.26E-07	6.27E-06
GO:006 0326	cell chemotaxis	47/196 6	3.28E-07	6.28E-06
GO:003 2680	regulation of tumor necrosis factor production	33/196 6	3.43E-07	6.55E-06
GO:000 3231	cardiac ventricle development	29/196 6	3.51E-07	6.69E-06
GO:000 0281	mitotic cytokinesis	18/196 6	3.53E-07	6.69E-06
GO:003 0193	regulation of blood coagulation	18/196 6	3.53E-07	6.69E-06
GO:000 1894	tissue homeostasis	44/196 6	3.55E-07	6.69E-06
GO:000 3208	cardiac ventricle morphogenesis	20/196 6	3.61E-07	6.78E-06
GO:006 0828	regulation of canonical Wnt signaling pathway	40/196 6	3.79E-07	7.10E-06
GO:000 6820	anion transport	65/196 6	3.87E-07	7.20E-06
GO:007 1174	mitotic spindle checkpoint signaling	14/196 6	3.88E-07	7.20E-06
GO:000 7080	mitotic metaphase plate congression	15/196 6	3.89E-07	7.20E-06
GO:001 0718	positive regulation of epithelial to mesenchymal transition	15/196 6	3.89E-07	7.20E-06
GO:000 2274	myeloid leukocyte activation	42/196 6	3.91E-07	7.21E-06
GO:006 1082	myeloid leukocyte cytokine production	Dec-66	4.09E-07	7.53E-06

GO:0071692	protein localization to extracellular region	56/1966	4.13E-07	7.58E-06
GO:0007091	metaphase/anaphase transition of mitotic cell cycle	17/1966	4.20E-07	7.67E-06
GO:0090307	mitotic spindle assembly	18/1966	4.50E-07	8.20E-06
GO:0051302	regulation of cell division	34/1966	4.52E-07	8.21E-06
GO:0051098	regulation of binding	55/1966	4.53E-07	8.21E-06
GO:0043433	negative regulation of DNA-binding transcription factor activity	31/1966	4.67E-07	8.43E-06
GO:0002718	regulation of cytokine production involved in immune response	22/1966	4.71E-07	8.47E-06
GO:0051402	neuron apoptotic process	47/1966	4.76E-07	8.54E-06
GO:0010876	lipid localization	60/1966	4.84E-07	8.66E-06
GO:0051146	striated muscle cell differentiation	48/1966	4.86E-07	8.66E-06
GO:1903555	regulation of tumor necrosis factor superfamily cytokine production	33/1966	5.03E-07	8.94E-06
GO:0071526	semaphorin-plexin signaling pathway	15/1966	5.26E-07	9.32E-06
GO:0006816	calcium ion transport	59/1966	5.36E-07	9.47E-06
GO:0010965	regulation of mitotic sister chromatid separation	17/1966	5.41E-07	9.54E-06
GO:0070252	actin-mediated cell contraction	22/1966	5.68E-07	9.93E-06
GO:0033045	regulation of sister chromatid segregation	18/1966	5.70E-07	9.93E-06
GO:0051926	negative regulation of calcium ion transport	18/1966	5.70E-07	9.93E-06
GO:1900046	regulation of hemostasis	18/1966	5.70E-07	9.93E-06

GO:1903036	positive regulation of response to wounding	19/1966	5.72E-07	9.93E-06
GO:0042326	negative regulation of phosphorylation	55/1966	5.78E-07	1.00E-05
GO:0071902	positive regulation of protein serine/threonine kinase activity	35/1966	5.80E-07	1.00E-05
GO:0052547	regulation of peptidase activity	59/1966	6.72E-07	1.16E-05
GO:0002690	positive regulation of leukocyte chemotaxis	22/1966	6.84E-07	1.17E-05
GO:1905517	macrophage migration	17/1966	6.95E-07	1.19E-05
GO:0061097	regulation of protein tyrosine kinase activity	19/1966	7.10E-07	1.21E-05
GO:0032640	tumor necrosis factor production	33/1966	7.29E-07	1.24E-05
GO:1905475	regulation of protein localization to membrane	34/1966	7.34E-07	1.24E-05
GO:0003014	renal system process	25/1966	7.36E-07	1.24E-05
GO:0002367	cytokine production involved in immune response	24/1966	7.36E-07	1.24E-05
GO:0061098	positive regulation of protein tyrosine kinase activity	14/1966	7.41E-07	1.25E-05
GO:1902105	regulation of leukocyte differentiation	47/1966	7.51E-07	1.26E-05
GO:0048562	embryonic organ morphogenesis	48/1966	7.60E-07	1.27E-05
GO:0050878	regulation of body fluid levels	52/1966	7.61E-07	1.27E-05
GO:0018209	peptidyl-serine modification	51/1966	8.32E-07	1.38E-05
GO:0006898	receptor-mediated endocytosis	41/1966	8.33E-07	1.38E-05
GO:0002697	regulation of immune effector process	58/1966	8.66E-07	1.43E-05

GO:0060840	artery development	24/1966	8.69E-07	1.43E-05
GO:0044784	metaphase/anaphase transition of cell cycle	17/1966	8.86E-07	1.46E-05
GO:0000075	cell cycle checkpoint signaling	31/1966	8.98E-07	1.47E-05
GO:0050818	regulation of coagulation	18/1966	9.02E-07	1.48E-05
GO:0060271	cilium assembly	50/1966	9.08E-07	1.48E-05
GO:0060348	bone development	40/1966	9.69E-07	1.58E-05
GO:0032147	activation of protein kinase activity	22/1966	9.82E-07	1.59E-05
GO:0001569	branching involved in blood vessel morphogenesis	14/1966	1.01E-06	1.62E-05
GO:0031577	spindle checkpoint signaling	14/1966	1.01E-06	1.62E-05
GO:1905521	regulation of macrophage migration	14/1966	1.01E-06	1.62E-05
GO:0032886	regulation of microtubule-based process	41/1966	1.02E-06	1.63E-05
GO:0050770	regulation of axonogenesis	32/1966	1.04E-06	1.66E-05
GO:0071706	tumor necrosis factor superfamily cytokine production	33/1966	1.05E-06	1.67E-05
GO:0032273	positive regulation of protein polymerization	21/1966	1.11E-06	1.77E-05
GO:0022409	positive regulation of cell-cell adhesion	44/1966	1.12E-06	1.77E-05
GO:0032835	glomerulus development	17/1966	1.12E-06	1.77E-05
GO:0051306	mitotic sister chromatid separation	17/1966	1.12E-06	1.77E-05
GO:0045664	regulation of neuron differentiation	38/1966	1.17E-06	1.85E-05

GO:0006022	aminoglycan metabolic process	23/1966	1.20E-06	1.89E-05
GO:0045839	negative regulation of mitotic nuclear division	15/1966	1.23E-06	1.93E-05
GO:0050729	positive regulation of inflammatory response	27/1966	1.24E-06	1.94E-05
GO:0014031	mesenchymal cell development	21/1966	1.33E-06	2.07E-05
GO:0072073	kidney epithelium development	28/1966	1.33E-06	2.07E-05
GO:0071695	anatomical structure maturation	44/1966	1.35E-06	2.09E-05
GO:0044843	cell cycle G1/S phase transition	37/1966	1.36E-06	2.09E-05
GO:0051985	negative regulation of chromosome segregation	14/1966	1.36E-06	2.09E-05
GO:0007094	mitotic spindle assembly checkpoint signaling	13/1966	1.40E-06	2.15E-05
GO:0071173	spindle assembly checkpoint signaling	13/1966	1.40E-06	2.15E-05
GO:1902749	regulation of cell cycle G2/M phase transition	23/1966	1.42E-06	2.17E-05
GO:0036293	response to decreased oxygen levels	38/1966	1.44E-06	2.20E-05
GO:0048015	phosphatidylinositol-mediated signaling	30/1966	1.45E-06	2.20E-05
GO:2001236	regulation of extrinsic apoptotic signaling pathway	30/1966	1.45E-06	2.20E-05
GO:0051222	positive regulation of protein transport	46/1966	1.51E-06	2.28E-05
GO:0061982	meiosis I cell cycle process	26/1966	1.52E-06	2.30E-05
GO:0046879	hormone secretion	51/1966	1.60E-06	2.41E-05
GO:2000351	regulation of endothelial cell apoptotic process	15/1966	1.61E-06	2.42E-05

GO:0001933	negative regulation of protein phosphorylation	49/1966	1.64E-06	2.45E-05
GO:0002064	epithelial cell development	37/1966	1.67E-06	2.50E-05
GO:0043409	negative regulation of MAPK cascade	32/1966	1.68E-06	2.51E-05
GO:0043523	regulation of neuron apoptotic process	42/1966	1.70E-06	2.53E-05
GO:1903510	mucopolysaccharide metabolic process	18/1966	1.73E-06	2.57E-05
GO:0033044	regulation of chromosome organization	34/1966	1.84E-06	2.73E-05
GO:0002028	regulation of sodium ion transport	21/1966	1.91E-06	2.82E-05
GO:0030516	regulation of axon extension	23/1966	1.96E-06	2.89E-05
GO:0014015	positive regulation of gliogenesis	19/1966	1.99E-06	2.92E-05
GO:0007043	cell-cell junction assembly	28/1966	1.99E-06	2.92E-05
GO:2001233	regulation of apoptotic signaling pathway	54/1966	2.05E-06	2.99E-05
GO:0043547	positive regulation of GTPase activity	39/1966	2.05E-06	2.99E-05
GO:0042391	regulation of membrane potential	60/1966	2.06E-06	3.00E-05
GO:0048017	inositol lipid-mediated signaling	30/1966	2.11E-06	3.07E-05
GO:0000910	cytokinesis	31/1966	2.13E-06	3.09E-05
GO:0043406	positive regulation of MAP kinase activity	25/1966	2.15E-06	3.10E-05
GO:0060324	face development	16/1966	2.22E-06	3.19E-05
GO:0001935	endothelial cell proliferation	31/1966	2.40E-06	3.45E-05

GO:004 4782	cilium organization	52/196 6	2.51E-06	3.59E-05
GO:004 5841	negative regulation of mitotic metaphase/anaphase transition	13/196 6	2.61E-06	3.74E-05
GO:000 9306	protein secretion	53/196 6	2.63E-06	3.75E-05
GO:009 7529	myeloid leukocyte migration	36/196 6	2.64E-06	3.75E-05
GO:003 4501	protein localization to kinetochore	Sep-66	2.65E-06	3.75E-05
GO:190 3083	protein localization to condensed chromosome	Sep-66	2.65E-06	3.75E-05
GO:005 2548	regulation of endopeptidase activity	49/196 6	2.66E-06	3.76E-05
GO:190 1652	response to peptide	55/196 6	2.67E-06	3.76E-05
GO:000 7416	synapse assembly	32/196 6	2.68E-06	3.76E-05
GO:003 4763	negative regulation of transmembrane transport	26/196 6	2.68E-06	3.76E-05
GO:190 1992	positive regulation of mitotic cell cycle phase transition	21/196 6	2.70E-06	3.78E-05
GO:005 1784	negative regulation of nuclear division	16/196 6	2.80E-06	3.91E-05
GO:003 5592	establishment of protein localization to extracellular region	53/196 6	2.84E-06	3.96E-05
GO:001 8105	peptidyl-serine phosphorylation	47/196 6	2.96E-06	4.11E-05
GO:003 1214	biomineral tissue development	30/196 6	3.04E-06	4.22E-05
GO:000 1649	osteoblast differentiation	35/196 6	3.06E-06	4.23E-05
GO:006 0976	coronary vasculature development	18/196 6	3.20E-06	4.42E-05
GO:003 0308	negative regulation of cell growth	33/196 6	3.28E-06	4.51E-05

GO:0048705	skeletal system morphogenesis	39/1966	3.32E-06	4.56E-05
GO:0042634	regulation of hair cycle	Nov-66	3.43E-06	4.68E-05
GO:1905523	positive regulation of macrophage migration	Nov-66	3.43E-06	4.68E-05
GO:0051653	spindle localization	15/1966	3.44E-06	4.69E-05
GO:0072577	endothelial cell apoptotic process	15/1966	3.44E-06	4.69E-05
GO:0002040	sprouting angiogenesis	24/1966	3.51E-06	4.73E-05
GO:0051261	protein depolymerization	24/1966	3.51E-06	4.73E-05
GO:0033046	negative regulation of sister chromatid segregation	13/1966	3.51E-06	4.73E-05
GO:0033048	negative regulation of mitotic sister chromatid segregation	13/1966	3.51E-06	4.73E-05
GO:2000816	negative regulation of mitotic sister chromatid separation	13/1966	3.51E-06	4.73E-05
GO:0010466	negative regulation of peptidase activity	39/1966	3.64E-06	4.89E-05
GO:0048846	axon extension involved in axon guidance	Dec-66	3.66E-06	4.89E-05
GO:1902284	neuron projection extension involved in neuron projection guidance	Dec-66	3.66E-06	4.89E-05
GO:0072009	nephron epithelium development	23/1966	3.67E-06	4.89E-05
GO:0010951	negative regulation of endopeptidase activity	31/1966	3.82E-06	5.08E-05
GO:0045927	positive regulation of growth	45/1966	3.85E-06	5.11E-05
GO:0061180	mammary gland epithelium development	18/1966	3.90E-06	5.17E-05
GO:0000727	double-strand break repair via break-induced replication	Jul-66	3.97E-06	5.25E-05

GO:0034614	cellular response to reactive oxygen species	26/1966	4.03E-06	5.32E-05
GO:1903053	regulation of extracellular matrix organization	14/1966	4.10E-06	5.40E-05
GO:0070293	renal absorption	Oct-66	4.12E-06	5.40E-05
GO:0071459	protein localization to chromosome, centromeric region	Oct-66	4.12E-06	5.40E-05
GO:0043010	camera-type eye development	50/1966	4.16E-06	5.43E-05
GO:0050866	negative regulation of cell activation	35/1966	4.17E-06	5.43E-05
GO:0060415	muscle tissue morphogenesis	19/1966	4.23E-06	5.51E-05
GO:0051897	positive regulation of protein kinase B signaling	23/1966	4.26E-06	5.53E-05
GO:0014911	positive regulation of smooth muscle cell migration	16/1966	4.39E-06	5.69E-05
GO:0030203	glycosaminoglycan metabolic process	20/1966	4.41E-06	5.70E-05
GO:0048644	muscle organ morphogenesis	20/1966	4.41E-06	5.70E-05
GO:0032872	regulation of stress-activated MAPK cascade	33/1966	4.53E-06	5.83E-05
GO:0072593	reactive oxygen species metabolic process	37/1966	4.55E-06	5.85E-05
GO:1902100	negative regulation of metaphase/anaphase transition of cell cycle	13/1966	4.68E-06	6.00E-05
GO:0035265	organ growth	34/1966	4.83E-06	6.18E-05
GO:0010038	response to metal ion	42/1966	4.98E-06	6.36E-05
GO:0090257	regulation of muscle system process	38/1966	5.16E-06	6.55E-05
GO:0032922	circadian regulation of gene expression	17/1966	5.17E-06	6.55E-05

GO:0046579	positive regulation of Ras protein signal transduction	17/1966	5.17E-06	6.55E-05
GO:0048864	stem cell development	20/1966	5.24E-06	6.63E-05
GO:1903522	regulation of blood circulation	39/1966	5.28E-06	6.66E-05
GO:0051293	establishment of spindle localization	14/1966	5.30E-06	6.68E-05
GO:0043524	negative regulation of neuron apoptotic process	31/1966	5.35E-06	6.72E-05
GO:0043393	regulation of protein binding	34/1966	5.35E-06	6.72E-05
GO:0031109	microtubule polymerization or depolymerization	24/1966	5.39E-06	6.75E-05
GO:0045862	positive regulation of proteolysis	48/1966	5.49E-06	6.86E-05
GO:0030071	regulation of mitotic metaphase/anaphase transition	15/1966	5.54E-06	6.90E-05
GO:1904951	positive regulation of establishment of protein localization	46/1966	5.71E-06	7.10E-05
GO:0000302	response to reactive oxygen species	31/1966	5.98E-06	7.41E-05
GO:0098739	import across plasma membrane	31/1966	5.98E-06	7.41E-05
GO:1905477	positive regulation of protein localization to membrane	22/1966	5.99E-06	7.41E-05
GO:0050766	positive regulation of phagocytosis	19/1966	6.06E-06	7.46E-05
GO:2001251	negative regulation of chromosome organization	19/1966	6.06E-06	7.46E-05
GO:0030204	chondroitin sulfate metabolic process	Oct-66	6.14E-06	7.55E-05
GO:1905819	negative regulation of chromosome separation	13/1966	6.17E-06	7.56E-05
GO:0044344	cellular response to fibroblast growth factor stimulus	20/1966	6.19E-06	7.56E-05

GO:0070302	regulation of stress-activated protein kinase signaling cascade	33/1966	6.21E-06	7.56E-05
GO:2000241	regulation of reproductive process	33/1966	6.21E-06	7.56E-05
GO:0031098	stress-activated protein kinase signaling cascade	38/1966	6.21E-06	7.56E-05
GO:1905330	regulation of morphogenesis of an epithelium	17/1966	6.31E-06	7.66E-05
GO:0060100	positive regulation of phagocytosis, engulfment	Aug-66	6.44E-06	7.79E-05
GO:1905155	positive regulation of membrane invagination	Aug-66	6.44E-06	7.79E-05
GO:0048639	positive regulation of developmental growth	34/1966	6.56E-06	7.91E-05
GO:1901989	positive regulation of cell cycle phase transition	23/1966	6.62E-06	7.97E-05
GO:0032506	cytokinetic process	Dec-66	6.79E-06	8.14E-05
GO:0061005	cell differentiation involved in kidney development	14/1966	6.80E-06	8.14E-05
GO:0043270	positive regulation of ion transport	45/1966	6.80E-06	8.14E-05
GO:0048844	artery morphogenesis	18/1966	6.89E-06	8.22E-05
GO:0048841	regulation of axon extension involved in axon guidance	Nov-66	6.89E-06	8.22E-05
GO:0006941	striated muscle contraction	29/1966	6.95E-06	8.26E-05
GO:0098657	import into cell	36/1966	7.11E-06	8.44E-05
GO:2001237	negative regulation of extrinsic apoptotic signaling pathway	21/1966	7.21E-06	8.51E-05
GO:0014032	neural crest cell development	19/1966	7.21E-06	8.51E-05
GO:0033627	cell adhesion mediated by integrin	19/1966	7.21E-06	8.51E-05

GO:0051781	positive regulation of cell division	20/1966	7.31E-06	8.60E-05
GO:0008544	epidermis development	48/1966	7.44E-06	8.75E-05
GO:0032874	positive regulation of stress-activated MAPK cascade	25/1966	7.48E-06	8.77E-05
GO:0048738	cardiac muscle tissue development	39/1966	7.56E-06	8.85E-05
GO:0030856	regulation of epithelial cell differentiation	27/1966	7.84E-06	9.16E-05
GO:0033047	regulation of mitotic sister chromatid segregation	13/1966	8.06E-06	9.40E-05
GO:0043589	skin morphogenesis	Jul-66	8.10E-06	9.43E-05
GO:0050803	regulation of synapse structure or activity	39/1966	8.26E-06	9.59E-05
GO:0016331	morphogenesis of embryonic epithelium	30/1966	8.54E-06	9.85E-05
GO:0035051	cardiocyte differentiation	30/1966	8.54E-06	9.85E-05
GO:0110148	biomineralization	30/1966	8.54E-06	9.85E-05
GO:0071774	response to fibroblast growth factor	20/1966	8.60E-06	9.91E-05
GO:1904036	negative regulation of epithelial cell apoptotic process	14/1966	8.66E-06	9.96E-05
GO:0002548	monocyte chemotaxis	15/1966	8.69E-06	9.98E-05
GO:0001936	regulation of endothelial cell proliferation	28/1966	8.84E-06	0.000101191
GO:0007009	plasma membrane organization	27/1966	8.85E-06	0.000101191
GO:0042886	amide transport	47/1966	8.89E-06	0.000101434
GO:0002683	negative regulation of immune system process	56/1966	8.93E-06	0.000101721

GO:0051091	positive regulation of DNA-binding transcription factor activity	39/1966	9.02E-06	0.000102527
GO:0022411	cellular component disassembly	54/1966	9.17E-06	0.000103989
GO:0046777	protein autophosphorylation	36/1966	9.44E-06	0.000106847
GO:0090504	epiboly	Nov-66	9.56E-06	0.000108024
GO:0051047	positive regulation of secretion	53/1966	9.58E-06	0.000108024
GO:0070304	positive regulation of stress-activated protein kinase signaling cascade	25/1966	9.70E-06	0.000109167
GO:0002700	regulation of production of molecular mediator of immune response	29/1966	9.77E-06	0.000109544
GO:0050807	regulation of synapse organization	38/1966	9.77E-06	0.000109544
GO:0035725	sodium ion transmembrane transport	26/1966	9.91E-06	0.000110912
GO:0030099	myeloid cell differentiation	55/1966	1.00E-05	0.000111852
GO:0014033	neural crest cell differentiation	20/1966	1.01E-05	0.000112523
GO:0050708	regulation of protein secretion	42/1966	1.06E-05	0.000117901
GO:0007612	learning	30/1966	1.06E-05	0.00011791
GO:2000027	regulation of animal organ morphogenesis	24/1966	1.07E-05	0.000118157
GO:0022898	regulation of transmembrane transporter activity	40/1966	1.07E-05	0.000118843
GO:1902099	regulation of metaphase/anaphase transition of cell cycle	15/1966	1.08E-05	0.000119384
GO:0001890	placenta development	29/1966	1.09E-05	0.000120393
GO:0032330	regulation of chondrocyte differentiation	14/1966	1.10E-05	0.000120654

GO:0030900	forebrain development	51/1966	1.12E-05	0.000122552
GO:0061326	renal tubule development	21/1966	1.14E-05	0.00012505
GO:0097581	lamellipodium organization	19/1966	1.20E-05	0.000130749
GO:1903035	negative regulation of response to wounding	19/1966	1.20E-05	0.000130749
GO:0035886	vascular associated smooth muscle cell differentiation	Dec-66	1.21E-05	0.000131689
GO:2001234	negative regulation of apoptotic signaling pathway	36/1966	1.24E-05	0.00013542
GO:0038127	ERBB signaling pathway	22/1966	1.25E-05	0.000135489
GO:0010971	positive regulation of G2/M transition of mitotic cell cycle	Oct-66	1.29E-05	0.000139426
GO:0002688	regulation of leukocyte chemotaxis	23/1966	1.33E-05	0.00014334
GO:0048259	regulation of receptor-mediated endocytosis	23/1966	1.33E-05	0.00014334
GO:0060491	regulation of cell projection assembly	32/1966	1.33E-05	0.00014334
GO:0031102	neuron projection regeneration	15/1966	1.34E-05	0.000143856
GO:0030517	negative regulation of axon extension	13/1966	1.34E-05	0.000144469
GO:0006029	proteoglycan metabolic process	17/1966	1.35E-05	0.000144719
GO:0006260	DNA replication	39/1966	1.39E-05	0.000148509
GO:1902806	regulation of cell cycle G1/S phase transition	28/1966	1.40E-05	0.000149084
GO:0061351	neural precursor cell proliferation	31/1966	1.40E-05	0.000149084
GO:0060047	heart contraction	35/1966	1.47E-05	0.00015615

GO:0032409	regulation of transporter activity	41/1966	1.48E-05	0.000157645
GO:0051403	stress-activated MAPK cascade	36/1966	1.49E-05	0.000158184
GO:0035904	aorta development	16/1966	1.51E-05	0.000159688
GO:0050771	negative regulation of axonogenesis	16/1966	1.51E-05	0.000159688
GO:0006750	glutathione biosynthetic process	Jul-66	1.52E-05	0.000160366
GO:0051764	actin crosslink formation	Jul-66	1.52E-05	0.000160366
GO:0051988	regulation of attachment of spindle microtubules to kinetochore	Jul-66	1.52E-05	0.000160366
GO:0032760	positive regulation of tumor necrosis factor production	21/1966	1.53E-05	0.000160714
GO:0006874	cellular calcium ion homeostasis	60/1966	1.54E-05	0.000160928
GO:0007596	blood coagulation	28/1966	1.56E-05	0.000163192
GO:0019932	second-messenger-mediated signaling	42/1966	1.58E-05	0.000165126
GO:0038084	vascular endothelial growth factor signaling pathway	Dec-66	1.59E-05	0.000165346
GO:2000379	positive regulation of reactive oxygen species metabolic process	17/1966	1.62E-05	0.000168203
GO:0042176	regulation of protein catabolic process	50/1966	1.64E-05	0.00017047
GO:0048260	positive regulation of receptor-mediated endocytosis	15/1966	1.64E-05	0.00017047
GO:0050764	regulation of phagocytosis	22/1966	1.65E-05	0.000170604
GO:0008589	regulation of smoothened signaling pathway	19/1966	1.65E-05	0.000170604
GO:0051899	membrane depolarization	19/1966	1.65E-05	0.000170604

GO:0071897	DNA biosynthetic process	29/1966	1.69E-05	0.000173691
GO:0048640	negative regulation of developmental growth	23/1966	1.73E-05	0.00017779
GO:0061387	regulation of extent of cell growth	23/1966	1.73E-05	0.00017779
GO:0008406	gonad development	35/1966	1.76E-05	0.000179968
GO:0048863	stem cell differentiation	35/1966	1.76E-05	0.000179968
GO:0032570	response to progesterone	Aug-66	1.77E-05	0.000180729
GO:0003015	heart process	36/1966	1.78E-05	0.000181536
GO:0031623	receptor internalization	24/1966	1.78E-05	0.000181566
GO:0009416	response to light stimulus	43/1966	1.81E-05	0.000183644
GO:0034121	regulation of toll-like receptor signaling pathway	16/1966	1.83E-05	0.000185247
GO:0045132	meiotic chromosome segregation	20/1966	1.87E-05	0.000189025
GO:0051057	positive regulation of small GTPase mediated signal transduction	17/1966	1.93E-05	0.000194989
GO:1901879	regulation of protein depolymerization	19/1966	1.94E-05	0.000195325
GO:0007098	centrosome cycle	23/1966	1.97E-05	0.000198437
GO:0014013	regulation of gliogenesis	23/1966	1.97E-05	0.000198437
GO:0030879	mammary gland development	27/1966	1.99E-05	0.000200192
GO:1903557	positive regulation of tumor necrosis factor superfamily cytokine production	21/1966	2.04E-05	0.000204144
GO:0045880	positive regulation of smoothened signaling pathway	Dec-66	2.06E-05	0.000206446

GO:0019748	secondary metabolic process	14/1966	2.14E-05	0.000213539
GO:0030514	negative regulation of BMP signaling pathway	14/1966	2.14E-05	0.000213539
GO:0090100	positive regulation of transmembrane receptor protein serine/threonine kinase signaling pathway	22/1966	2.16E-05	0.000214278
GO:1902414	protein localization to cell junction	22/1966	2.16E-05	0.000214278
GO:0007599	hemostasis	28/1966	2.16E-05	0.000214278
GO:0050817	coagulation	28/1966	2.16E-05	0.000214278
GO:0032675	regulation of interleukin-6 production	27/1966	2.23E-05	0.00022039
GO:0007044	cell-substrate junction assembly	19/1966	2.26E-05	0.00022316
GO:0051092	positive regulation of NF-kappaB transcription factor activity	26/1966	2.27E-05	0.000224168
GO:0006261	DNA-templated DNA replication	25/1966	2.30E-05	0.00022589
GO:0003151	outflow tract morphogenesis	18/1966	2.31E-05	0.000226868
GO:0034114	regulation of heterotypic cell-cell adhesion	Sep-66	2.36E-05	0.000231151
GO:0022617	extracellular matrix disassembly	Nov-66	2.36E-05	0.000231272
GO:0009636	response to toxic substance	22/1966	2.47E-05	0.000241414
GO:0032412	regulation of ion transmembrane transporter activity	38/1966	2.50E-05	0.000243944
GO:0021952	central nervous system projection neuron axonogenesis	Oct-66	2.51E-05	0.000244021
GO:0071711	basement membrane organization	Oct-66	2.51E-05	0.000244021
GO:0045137	development of primary sexual characteristics	35/1966	2.51E-05	0.000244021

GO:0045598	regulation of fat cell differentiation	25/1966	2.58E-05	0.000250333
GO:0021955	central nervous system neuron axonogenesis	Dec-66	2.66E-05	0.000257615
GO:0045732	positive regulation of protein catabolic process	33/1966	2.68E-05	0.000259054
GO:0003215	cardiac right ventricle morphogenesis	Aug-66	2.77E-05	0.000267498
GO:0042542	response to hydrogen peroxide	22/1966	2.82E-05	0.000271255
GO:0007127	meiosis I	23/1966	2.89E-05	0.000277884
GO:0008584	male gonad development	24/1966	2.92E-05	0.000279009
GO:0009855	determination of bilateral symmetry	24/1966	2.92E-05	0.000279009
GO:1904375	regulation of protein localization to cell periphery	24/1966	2.92E-05	0.000279009
GO:0048511	rhythmic process	41/1966	3.00E-05	0.000286951
GO:0032231	regulation of actin filament bundle assembly	21/1966	3.08E-05	0.000293877
GO:0007292	female gamete generation	27/1966	3.09E-05	0.000294313
GO:0060674	placenta blood vessel development	Nov-66	3.12E-05	0.000296672
GO:0000725	recombinational repair	26/1966	3.19E-05	0.000302494
GO:0001578	microtubule bundle formation	22/1966	3.21E-05	0.000303348
GO:0002224	toll-like receptor signaling pathway	22/1966	3.21E-05	0.000303348
GO:0051147	regulation of muscle cell differentiation	25/1966	3.25E-05	0.000307397
GO:0009799	specification of symmetry	24/1966	3.29E-05	0.000309326

GO:0046546	development of primary male sexual characteristics	24/1966	3.29E-05	0.000309326
GO:0090596	sensory organ morphogenesis	42/1966	3.39E-05	0.000319024
GO:0030261	chromosome condensation	Dec-66	3.41E-05	0.000319666
GO:1902751	positive regulation of cell cycle G2/M phase transition	Oct-66	3.43E-05	0.000320894
GO:0044771	meiotic cell cycle phase transition	Jun-66	3.43E-05	0.000320894
GO:0048872	homeostasis of number of cells	47/1966	3.46E-05	0.000323525
GO:0030595	leukocyte chemotaxis	33/1966	3.52E-05	0.000326823
GO:0055001	muscle cell development	33/1966	3.52E-05	0.000326823
GO:0030038	contractile actin filament bundle assembly	21/1966	3.52E-05	0.000326823
GO:0043149	stress fiber assembly	21/1966	3.52E-05	0.000326823
GO:0071214	cellular response to abiotic stimulus	43/1966	3.53E-05	0.000326823
GO:0104004	cellular response to environmental stimulus	43/1966	3.53E-05	0.000326823
GO:0007159	leukocyte cell-cell adhesion	48/1966	3.54E-05	0.000326823
GO:0001938	positive regulation of endothelial cell proliferation	19/1966	3.55E-05	0.000327245
GO:0060350	endochondral bone morphogenesis	15/1966	3.59E-05	0.000330926
GO:0015833	peptide transport	42/1966	3.65E-05	0.000335979
GO:0031023	microtubule organizing center organization	24/1966	3.70E-05	0.000339355
GO:0014068	positive regulation of phosphatidylinositol 3-kinase signaling	16/1966	3.76E-05	0.000345031

GO:0070373	negative regulation of ERK1 and ERK2 cascade	17/1966	3.79E-05	0.000347122
GO:0010001	glial cell differentiation	36/1966	3.84E-05	0.000351256
GO:0009991	response to extracellular stimulus	50/1966	3.90E-05	0.000355434
GO:0014009	glial cell proliferation	14/1966	3.98E-05	0.000362082
GO:0060443	mammary gland morphogenesis	14/1966	3.98E-05	0.000362082
GO:0015711	organic anion transport	45/1966	4.05E-05	0.000367483
GO:0050728	negative regulation of inflammatory response	24/1966	4.15E-05	0.00037657
GO:0060099	regulation of phagocytosis, engulfment	Aug-66	4.21E-05	0.000381413
GO:0032233	positive regulation of actin filament bundle assembly	15/1966	4.32E-05	0.000390143
GO:0035050	embryonic heart tube development	18/1966	4.32E-05	0.000390143
GO:0019184	nonribosomal peptide biosynthetic process	Jul-66	4.50E-05	0.000405568
GO:0002063	chondrocyte development	Oct-66	4.62E-05	0.000413108
GO:0006270	DNA replication initiation	Oct-66	4.62E-05	0.000413108
GO:0044319	wound healing, spreading of cells	Oct-66	4.62E-05	0.000413108
GO:0060740	prostate gland epithelium morphogenesis	Oct-66	4.62E-05	0.000413108
GO:0090505	epiboly involved in wound healing	Oct-66	4.62E-05	0.000413108
GO:1901653	cellular response to peptide	41/1966	4.70E-05	0.000420131
GO:0031954	positive regulation of protein autophosphorylation	Sep-66	4.77E-05	0.000424526

GO:0048843	negative regulation of axon extension involved in axon guidance	Sep-66	4.77E-05	0.000424526
GO:0072012	glomerulus vasculature development	Sep-66	4.77E-05	0.000424526
GO:0010812	negative regulation of cell-substrate adhesion	14/1966	4.84E-05	0.000430087
GO:0060349	bone morphogenesis	20/1966	5.03E-05	0.000445726
GO:0019221	cytokine-mediated signaling pathway	51/1966	5.05E-05	0.000447333
GO:0071677	positive regulation of mononuclear cell migration	15/1966	5.18E-05	0.000457574
GO:0006023	aminoglycan biosynthetic process	13/1966	5.26E-05	0.000462459
GO:0031103	axon regeneration	13/1966	5.26E-05	0.000462459
GO:0048483	autonomic nervous system development	13/1966	5.26E-05	0.000462459
GO:0060351	cartilage development involved in endochondral bone morphogenesis	Nov-66	5.28E-05	0.000463112
GO:1905332	positive regulation of morphogenesis of an epithelium	Nov-66	5.28E-05	0.000463112
GO:0002090	regulation of receptor internalization	16/1966	5.29E-05	0.000463112
GO:0051348	negative regulation of transferase activity	36/1966	5.32E-05	0.000465638
GO:0042116	macrophage activation	19/1966	5.45E-05	0.000474169
GO:0072080	nephron tubule development	19/1966	5.45E-05	0.000474169
GO:0150115	cell-substrate junction organization	19/1966	5.45E-05	0.000474169
GO:0007219	Notch signaling pathway	28/1966	5.46E-05	0.000474169
GO:0050714	positive regulation of protein secretion	26/1966	5.46E-05	0.000474169

GO:0001838	embryonic epithelial tube formation	25/1966	5.67E-05	0.000491902
GO:0032635	interleukin-6 production	27/1966	5.77E-05	0.000499672
GO:0032386	regulation of intracellular transport	43/1966	5.80E-05	0.000501747
GO:0060759	regulation of response to cytokine stimulus	24/1966	5.84E-05	0.000504133
GO:0010171	body morphogenesis	14/1966	5.87E-05	0.000505031
GO:0051058	negative regulation of small GTPase mediated signal transduction	14/1966	5.87E-05	0.000505031
GO:0008361	regulation of cell size	31/1966	5.88E-05	0.000505581
GO:0008306	associative learning	21/1966	5.92E-05	0.00050767
GO:0048041	focal adhesion assembly	17/1966	6.10E-05	0.000522407
GO:0002092	positive regulation of receptor internalization	Oct-66	6.14E-05	0.000523491
GO:0050654	chondroitin sulfate proteoglycan metabolic process	Oct-66	6.14E-05	0.000523491
GO:0060512	prostate gland morphogenesis	Oct-66	6.14E-05	0.000523491
GO:0099560	synaptic membrane adhesion	Oct-66	6.14E-05	0.000523491
GO:0031579	membrane raft organization	Aug-66	6.22E-05	0.000526505
GO:0060080	inhibitory postsynaptic potential	Aug-66	6.22E-05	0.000526505
GO:0150104	transport across blood-brain barrier	Aug-66	6.22E-05	0.000526505
GO:1905153	regulation of membrane invagination	Aug-66	6.22E-05	0.000526505
GO:2001224	positive regulation of neuron migration	Aug-66	6.22E-05	0.000526505

GO:0014066	regulation of phosphatidylinositol 3-kinase signaling	19/1966	6.26E-05	0.000528841
GO:0120032	regulation of plasma membrane bounded cell projection assembly	30/1966	6.35E-05	0.000535477
GO:0030838	positive regulation of actin filament polymerization	13/1966	6.46E-05	0.000540556
GO:0045687	positive regulation of glial cell differentiation	13/1966	6.46E-05	0.000540556
GO:0046580	negative regulation of Ras protein signal transduction	13/1966	6.46E-05	0.000540556
GO:2001222	regulation of neuron migration	13/1966	6.46E-05	0.000540556
GO:0060433	bronchus development	Jun-66	6.46E-05	0.000540556
GO:0060907	positive regulation of macrophage cytokine production	Jun-66	6.46E-05	0.000540556
GO:0001843	neural tube closure	20/1966	6.55E-05	0.000547108
GO:0033622	integrin activation	Sep-66	6.61E-05	0.000549857
GO:0061437	renal system vasculature development	Sep-66	6.61E-05	0.000549857
GO:0061440	kidney vasculature development	Sep-66	6.61E-05	0.000549857
GO:0072175	epithelial tube formation	26/1966	6.72E-05	0.000558108
GO:0060996	dendritic spine development	22/1966	6.74E-05	0.000558787
GO:0060603	mammary gland duct morphogenesis	Nov-66	6.78E-05	0.000561287
GO:0030195	negative regulation of blood coagulation	Dec-66	6.82E-05	0.00056215
GO:0048010	vascular endothelial growth factor receptor signaling pathway	Dec-66	6.82E-05	0.00056215
GO:0048246	macrophage chemotaxis	Dec-66	6.82E-05	0.00056215

GO:000 0082	G1/S transition of mitotic cell cycle	30/196 6	6.95E-05	0.00057 2649
GO:000 3413	chondrocyte differentiation involved in endochondral bone morphogenesis	Jul-66	7.20E-05	0.00059 0404
GO:005 1382	kinetochore assembly	Jul-66	7.20E-05	0.00059 0404
GO:009 0136	epithelial cell-cell adhesion	Jul-66	7.20E-05	0.00059 0404
GO:000 6302	double-strand break repair	36/196 6	7.30E-05	0.00059 8153
GO:200 0134	negative regulation of G1/S transition of mitotic cell cycle	15/196 6	7.34E-05	0.00060 0553
GO:006 0606	tube closure	20/196 6	7.45E-05	0.00060 8932
GO:002 1782	glial cell development	21/196 6	7.58E-05	0.00061 7713
GO:003 2612	interleukin-1 production	21/196 6	7.58E-05	0.00061 7713
GO:000 9913	epidermal cell differentiation	33/196 6	7.64E-05	0.00062 1665
GO:003 3157	regulation of intracellular protein transport	32/196 6	7.68E-05	0.00062 3053
GO:004 8545	response to steroid hormone	32/196 6	7.68E-05	0.00062 3053
GO:003 1110	regulation of microtubule polymerization or depolymerization	18/196 6	7.76E-05	0.00062 836
GO:190 2904	negative regulation of supramolecular fiber organization	27/196 6	7.78E-05	0.00062 9088
GO:190 3131	mononuclear cell differentiation	57/196 6	7.85E-05	0.00063 3988
GO:004 6942	carboxylic acid transport	38/196 6	8.06E-05	0.00064 905
GO:009 0162	establishment of epithelial cell polarity	Oct-66	8.08E-05	0.00064 905
GO:190 1099	negative regulation of signal transduction in absence of ligand	Oct-66	8.08E-05	0.00064 905

GO:2001240	negative regulation of extrinsic apoptotic signaling pathway in absence of ligand	Oct-66	8.08E-05	0.00064905
GO:0071248	cellular response to metal ion	26/1966	8.23E-05	0.000659938
GO:0001738	morphogenesis of a polarized epithelium	17/1966	8.25E-05	0.000659938
GO:0021675	nerve development	17/1966	8.25E-05	0.000659938
GO:0015849	organic acid transport	41/1966	8.33E-05	0.000665572
GO:0070588	calcium ion transmembrane transport	39/1966	8.38E-05	0.000668911
GO:1900047	negative regulation of hemostasis	Dec-66	8.47E-05	0.000674158
GO:2001239	regulation of extrinsic apoptotic signaling pathway in absence of ligand	Dec-66	8.47E-05	0.000674158
GO:0060393	regulation of pathway-restricted SMAD protein phosphorylation	14/1966	8.49E-05	0.000674598
GO:0007368	determination of left/right symmetry	22/1966	8.52E-05	0.000674598
GO:0043244	regulation of protein-containing complex disassembly	22/1966	8.52E-05	0.000674598
GO:0060048	cardiac muscle contraction	22/1966	8.52E-05	0.000674598
GO:0007369	gastrulation	27/1966	8.57E-05	0.000677699
GO:0007143	female meiotic nuclear division	Nov-66	8.62E-05	0.000680532
GO:0055008	cardiac muscle tissue morphogenesis	15/1966	8.69E-05	0.000685643
GO:1902107	positive regulation of leukocyte differentiation	28/1966	8.82E-05	0.00069393
GO:1903708	positive regulation of hemopoiesis	28/1966	8.82E-05	0.00069393
GO:0010232	vascular transport	Aug-66	8.97E-05	0.000703714

GO:0071496	cellular response to external stimulus	39/1966	9.01E-05	0.000703714
GO:0003148	outflow tract septum morphogenesis	Sep-66	9.01E-05	0.000703714
GO:0060444	branching involved in mammary gland duct morphogenesis	Sep-66	9.01E-05	0.000703714
GO:0097205	renal filtration	Sep-66	9.01E-05	0.000703714
GO:0061333	renal tubule morphogenesis	17/1966	9.55E-05	0.000745359
GO:0010463	mesenchymal cell proliferation	13/1966	9.58E-05	0.000745773
GO:0051489	regulation of filopodium assembly	13/1966	9.58E-05	0.000745773
GO:0120034	positive regulation of plasma membrane bounded cell projection assembly	20/1966	9.60E-05	0.000746295
GO:0070482	response to oxygen levels	39/1966	9.67E-05	0.000750892
GO:0008543	fibroblast growth factor receptor signaling pathway	16/1966	0.000100274	0.000776243
GO:1902807	negative regulation of cell cycle G1/S phase transition	16/1966	0.000100274	0.000776243
GO:0001954	positive regulation of cell-matrix adhesion	14/1966	0.000101491	0.000784633
GO:1904063	negative regulation of cation transmembrane transport	18/1966	0.000102427	0.00079084
GO:0043583	ear development	34/1966	0.000104133	0.000802962
GO:0003254	regulation of membrane depolarization	Dec-66	0.000104576	0.000804283
GO:0050819	negative regulation of coagulation	Dec-66	0.000104576	0.000804283
GO:0002053	positive regulation of mesenchymal cell proliferation	Oct-66	0.000105038	0.000806793
GO:0034766	negative regulation of ion transmembrane transport	19/1966	0.000106715	0.000818608

GO:0032652	regulation of interleukin-1 production	20/1966	0.000108692	0.000830757
GO:0043534	blood vessel endothelial cell migration	20/1966	0.000108692	0.000830757
GO:0050892	intestinal absorption	Nov-66	0.000108719	0.000830757
GO:0070661	leukocyte proliferation	45/1966	0.000109177	0.000833179
GO:0046209	nitric oxide metabolic process	17/1966	0.000110378	0.000841261
GO:0046661	male sex differentiation	26/1966	0.000110771	0.000843166
GO:0032060	bleb assembly	Jun-66	0.000113075	0.000854117
GO:0032905	transforming growth factor beta1 production	Jun-66	0.000113075	0.000854117
GO:0051315	attachment of mitotic spindle microtubules to kinetochore	Jun-66	0.000113075	0.000854117
GO:0071635	negative regulation of transforming growth factor beta production	Jun-66	0.000113075	0.000854117
GO:0097084	vascular associated smooth muscle cell development	Jun-66	0.000113075	0.000854117
GO:1905048	regulation of metallopeptidase activity	Jun-66	0.000113075	0.000854117
GO:0016311	dephosphorylation	43/1966	0.000113717	0.000857878
GO:0035148	tube formation	27/1966	0.000114135	0.000859931
GO:0002573	myeloid leukocyte differentiation	33/1966	0.000114832	0.000864089
GO:0090303	positive regulation of wound healing	13/1966	0.000115873	0.000870809
GO:0038034	signal transduction in absence of ligand	16/1966	0.000116782	0.000875418
GO:0097192	extrinsic apoptotic signaling pathway in absence of ligand	16/1966	0.000116782	0.000875418

GO:0048286	lung alveolus development	14/1966	0.000120806	0.000901853
GO:0060688	regulation of morphogenesis of a branching structure	14/1966	0.000120806	0.000901853
GO:0002029	desensitization of G protein-coupled receptor signaling pathway	Sep-66	0.000120917	0.000901853
GO:0022401	negative adaptation of signaling pathway	Sep-66	0.000120917	0.000901853
GO:0035296	regulation of tube diameter	26/1966	0.000122037	0.000907922
GO:0097746	blood vessel diameter maintenance	26/1966	0.000122037	0.000907922
GO:0006939	smooth muscle contraction	20/1966	0.00012281	0.000912525
GO:0043242	negative regulation of protein-containing complex disassembly	17/1966	0.000127212	0.000942863
GO:0060420	regulation of heart growth	17/1966	0.000127212	0.000942863
GO:0051099	positive regulation of binding	28/1966	0.000127446	0.000943418
GO:0006937	regulation of muscle contraction	25/1966	0.000129766	0.000959391
GO:0014065	phosphatidylinositol 3-kinase signaling	22/1966	0.00013391	0.000988798
GO:0035150	regulation of tube size	26/1966	0.000134316	0.000989324
GO:1903169	regulation of calcium ion transmembrane transport	26/1966	0.000134316	0.000989324
GO:0035909	aorta morphogenesis	Oct-66	0.000135191	0.00099933
GO:0045746	negative regulation of Notch signaling pathway	Oct-66	0.000135191	0.00099933
GO:0010464	regulation of mesenchymal cell proliferation	Nov-66	0.000136085	0.000998631
GO:0042310	vasoconstriction	19/1966	0.000137664	0.001008966

GO:0007528	neuromuscular junction development	13/1966	0.000139422	0.001020589
GO:2001057	reactive nitrogen species metabolic process	17/1966	0.00014625	0.001069252
GO:0032611	interleukin-1 beta production	18/1966	0.000152854	0.001114779
GO:0062207	regulation of pattern recognition receptor signaling pathway	18/1966	0.000152854	0.001114779
GO:0030282	bone mineralization	21/1966	0.000153635	0.001119099
GO:0014020	primary neural tube formation	20/1966	0.00015598	0.001134785
GO:0060191	regulation of lipase activity	16/1966	0.000157039	0.001141084
GO:0002699	positive regulation of immune effector process	39/1966	0.000157494	0.001142987
GO:1903364	positive regulation of cellular protein catabolic process	23/1966	0.000159589	0.001156359
GO:0035767	endothelial cell chemotaxis	Sep-66	0.000160117	0.001156359
GO:0061036	positive regulation of cartilage development	Sep-66	0.000160117	0.001156359
GO:2000352	negative regulation of endothelial cell apoptotic process	Sep-66	0.000160117	0.001156359
GO:0051494	negative regulation of cytoskeleton organization	26/1966	0.000162221	0.001170124
GO:0002263	cell activation involved in immune response	41/1966	0.000163389	0.00117712
GO:0051054	positive regulation of DNA metabolic process	38/1966	0.000164631	0.0011844
GO:0021783	preganglionic parasympathetic fiber development	Jul-66	0.000165187	0.0011844
GO:0033623	regulation of integrin activation	Jul-66	0.000165187	0.0011844
GO:0080164	regulation of nitric oxide metabolic process	15/1966	0.00016552	0.0011844

GO:0044272	sulfur compound biosynthetic process	17/1966	0.000167733	0.001199657
GO:0070301	cellular response to hydrogen peroxide	17/1966	0.000167733	0.001199657
GO:0051480	regulation of cytosolic calcium ion concentration	47/1966	0.000168282	0.001202129
GO:0050918	positive chemotaxis	Nov-66	0.000169093	0.001204003
GO:0006801	superoxide metabolic process	14/1966	0.000169154	0.001204003
GO:0060389	pathway-restricted SMAD protein phosphorylation	14/1966	0.000169154	0.001204003
GO:0042476	odontogenesis	21/1966	0.000171887	0.001220717
GO:0051294	establishment of spindle orientation	Oct-66	0.000172327	0.001220717
GO:0072210	metanephric nephron development	Oct-66	0.000172327	0.001220717
GO:2000758	positive regulation of peptidyl-lysine acetylation	Oct-66	0.000172327	0.001220717
GO:0009100	glycoprotein metabolic process	41/1966	0.000174369	0.001233709
GO:0010759	positive regulation of macrophage chemotaxis	Aug-66	0.000175025	0.00123354
GO:0035988	chondrocyte proliferation	Aug-66	0.000175025	0.00123354
GO:0002221	pattern recognition receptor signaling pathway	26/1966	0.000178018	0.001253629
GO:0021537	telencephalon development	34/1966	0.000178031	0.001253629
GO:0021953	central nervous system neuron differentiation	29/1966	0.000181215	0.001272389
GO:0034103	regulation of tissue remodeling	16/1966	0.000181352	0.001272389
GO:0072088	nephron epithelium morphogenesis	16/1966	0.000181352	0.001272389

GO:0008016	regulation of heart contraction	28/1966	0.000181555	0.001272389
GO:0030072	peptide hormone secretion	37/1966	0.000184135	0.001288944
GO:0000724	double-strand break repair via homologous recombination	24/1966	0.000185137	0.001294432
GO:1902969	mitotic DNA replication	Jun-66	0.000186446	0.001300511
GO:2000052	positive regulation of non-canonical Wnt signaling pathway	Jun-66	0.000186446	0.001300511
GO:0030850	prostate gland development	Dec-66	0.000189931	0.001323262
GO:0021954	central nervous system neuron development	17/1966	0.000191919	0.001334694
GO:0003279	cardiac septum development	21/1966	0.000192023	0.001334694
GO:0051963	regulation of synapse assembly	20/1966	0.000196795	0.001366257
GO:0022408	negative regulation of cell-cell adhesion	29/1966	0.000197093	0.001366728
GO:0099565	chemical synaptic transmission, postsynaptic	18/1966	0.000197506	0.00136799
GO:0003143	embryonic heart tube morphogenesis	14/1966	0.000199034	0.001374064
GO:1904377	positive regulation of protein localization to cell periphery	14/1966	0.000199034	0.001374064
GO:0032731	positive regulation of interleukin-1 beta production	13/1966	0.000199079	0.001374064
GO:0007389	pattern specification process	55/1966	0.00020088	0.00138488
GO:0031349	positive regulation of defense response	38/1966	0.000202072	0.001391479
GO:0014902	myotube differentiation	22/1966	0.000205812	0.001415584
GO:0031952	regulation of protein autophosphorylation	Nov-66	0.000208647	0.001429978

GO:0060323	head morphogenesis	Nov-66	0.000208647	0.001429978
GO:1903170	negative regulation of calcium ion transmembrane transport	Nov-66	0.000208647	0.001429978
GO:0019229	regulation of vasoconstriction	16/1966	0.000208871	0.001429978
GO:0023058	adaptation of signaling pathway	Sep-66	0.00020936	0.001431673
GO:0060402	calcium ion transport into cytosol	26/1966	0.000213768	0.00146013
GO:0051592	response to calcium ion	21/1966	0.000214206	0.001461439
GO:1904645	response to amyloid-beta	Oct-66	0.00021768	0.001483431
GO:0032651	regulation of interleukin-1 beta production	17/1966	0.000219089	0.001491315
GO:0060419	heart growth	20/1966	0.000220513	0.001499284
GO:0007173	epidermal growth factor receptor signaling pathway	18/1966	0.000223827	0.00152007
GO:2000377	regulation of reactive oxygen species metabolic process	24/1966	0.000224929	0.001525807
GO:0045861	negative regulation of proteolysis	43/1966	0.000227558	0.001541878
GO:0002703	regulation of leukocyte mediated immunity	38/1966	0.00023111	0.001564156
GO:0060711	labyrinthine layer development	13/1966	0.00023633	0.001597655
GO:0002031	G protein-coupled receptor internalization	Jul-66	0.000239201	0.001611031
GO:0010763	positive regulation of fibroblast migration	Jul-66	0.000239201	0.001611031
GO:0072010	glomerular epithelium development	Jul-66	0.000239201	0.001611031
GO:0035418	protein localization to synapse	16/1966	0.000239941	0.001611031

GO:0072028	nephron morphogenesis	16/1966	0.000239941	0.001611031
GO:1902893	regulation of miRNA transcription	16/1966	0.000239941	0.001611031
GO:0050863	regulation of T cell activation	42/1966	0.000241209	0.001617713
GO:0046330	positive regulation of JNK cascade	18/1966	0.000253155	0.001695911
GO:0045619	regulation of lymphocyte differentiation	28/1966	0.000255245	0.00170753
GO:0006024	glycosaminoglycan biosynthetic process	Nov-66	0.000255755	0.00170753
GO:1903524	positive regulation of blood circulation	Nov-66	0.000255755	0.00170753
GO:0045637	regulation of myeloid cell differentiation	30/1966	0.00026863	0.001791474
GO:0006790	sulfur compound metabolic process	40/1966	0.00027004	0.001798849
GO:0033260	nuclear DNA replication	Sep-66	0.000270552	0.001800236
GO:0042398	cellular modified amino acid biosynthetic process	Oct-66	0.000272631	0.001810004
GO:0060325	face morphogenesis	Oct-66	0.000272631	0.001810004
GO:0034113	heterotypic cell-cell adhesion	Dec-66	0.000275123	0.001823395
GO:0015748	organophosphate ester transport	20/1966	0.000275572	0.001823395
GO:1901890	positive regulation of cell junction assembly	20/1966	0.000275572	0.001823395
GO:0001765	membrane raft assembly	May-66	0.000277256	0.001830443
GO:0099640	axo-dendritic protein transport	May-66	0.000277256	0.001830443
GO:0001755	neural crest cell migration	13/1966	0.000279383	0.001840386

GO:0045744	negative regulation of G protein-coupled receptor signaling pathway	13/1966	0.000279383	0.001840386
GO:0046883	regulation of hormone secretion	38/1966	0.000281703	0.001853606
GO:0070555	response to interleukin-1	17/1966	0.000283609	0.001864077
GO:0035264	multicellular organism growth	30/1966	0.000290511	0.001907322
GO:0007076	mitotic chromosome condensation	Jun-66	0.000292812	0.001918179
GO:1904177	regulation of adipose tissue development	Jun-66	0.000292812	0.001918179
GO:0001841	neural tube formation	21/1966	0.000294842	0.001929343
GO:1904888	cranial skeletal system development	15/1966	0.00029836	0.001950208
GO:0050680	negative regulation of epithelial cell proliferation	25/1966	0.00030321	0.001979725
GO:0015850	organic hydroxy compound transport	33/1966	0.000307641	0.002006445
GO:0003179	heart valve morphogenesis	Nov-66	0.000311527	0.002029552
GO:0061614	miRNA transcription	16/1966	0.000314257	0.00204509
GO:0002790	peptide secretion	37/1966	0.000316464	0.00205719
GO:0045428	regulation of nitric oxide biosynthetic process	14/1966	0.000317451	0.002061121
GO:0062009	secondary palate development	Aug-66	0.000317765	0.002061121
GO:0001657	ureteric bud development	18/1966	0.00032199	0.002079414
GO:0072163	mesonephric epithelium development	18/1966	0.00032199	0.002079414
GO:0072164	mesonephric tubule development	18/1966	0.00032199	0.002079414

GO:2000243	positive regulation of reproductive process	18/1966	0.00032199	0.002079414
GO:0030834	regulation of actin filament depolymerization	Dec-66	0.000328649	0.002115163
GO:0043536	positive regulation of blood vessel endothelial cell migration	Dec-66	0.000328649	0.002115163
GO:0051893	regulation of focal adhesion assembly	13/1966	0.000328954	0.002115163
GO:0090109	regulation of cell-substrate junction assembly	13/1966	0.000328954	0.002115163
GO:0006268	DNA unwinding involved in DNA replication	Jul-66	0.00033774	0.002164608
GO:0051797	regulation of hair follicle development	Jul-66	0.00033774	0.002164608
GO:0090594	inflammatory response to wounding	Jul-66	0.00033774	0.002164608
GO:0035850	epithelial cell differentiation involved in kidney development	Oct-66	0.000338717	0.002166177
GO:2000463	positive regulation of excitatory postsynaptic potential	Oct-66	0.000338717	0.002166177
GO:0018958	phenol-containing compound metabolic process	20/1966	0.000342293	0.002186552
GO:0006749	glutathione metabolic process	15/1966	0.00034338	0.002186552
GO:0032088	negative regulation of NF-kappaB transcription factor activity	15/1966	0.00034338	0.002186552
GO:1901880	negative regulation of protein depolymerization	15/1966	0.00034338	0.002186552
GO:1903037	regulation of leukocyte cell-cell adhesion	41/1966	0.000347129	0.002208052
GO:0097553	calcium ion transmembrane import into cytosol	23/1966	0.00035262	0.002240572
GO:0001892	embryonic placenta development	19/1966	0.000354395	0.002249438
GO:0008593	regulation of Notch signaling pathway	16/1966	0.000358339	0.00227204

GO:0072676	lymphocyte migration	18/1966	0.000362123	0.002293574
GO:0006672	ceramide metabolic process	17/1966	0.000363996	0.002302975
GO:0000079	regulation of cyclin-dependent protein serine/threonine kinase activity	14/1966	0.000368431	0.002326066
GO:0061045	negative regulation of wound healing	14/1966	0.000368431	0.002326066
GO:1901985	positive regulation of protein acetylation	Nov-66	0.000377188	0.002376588
GO:2000045	regulation of G1/S transition of mitotic cell cycle	22/1966	0.000377236	0.002376588
GO:0051101	regulation of DNA binding	20/1966	0.000380645	0.002395512
GO:0048008	platelet-derived growth factor receptor signaling pathway	13/1966	0.00038582	0.002425504
GO:0019722	calcium-mediated signaling	27/1966	0.00039017	0.002448591
GO:0035567	non-canonical Wnt signaling pathway	Dec-66	0.000390733	0.002448591
GO:0140353	lipid export from cell	Dec-66	0.000390733	0.002448591
GO:0033673	negative regulation of kinase activity	30/1966	0.000394688	0.002470763
GO:0060078	regulation of postsynaptic membrane potential	19/1966	0.000395825	0.002475263
GO:0001959	regulation of cytokine-mediated signaling pathway	21/1966	0.000400901	0.002496462
GO:0030278	regulation of ossification	21/1966	0.000400901	0.002496462
GO:0042303	molting cycle	21/1966	0.000400901	0.002496462
GO:0042633	hair cycle	21/1966	0.000400901	0.002496462
GO:0032755	positive regulation of interleukin-6 production	18/1966	0.000406521	0.002528802

GO:0099173	postsynapse organization	28/1966	0.000415592	0.002575806
GO:0018149	peptide cross-linking	Oct-66	0.000417632	0.002575806
GO:0090175	regulation of establishment of planar polarity	Oct-66	0.000417632	0.002575806
GO:0090224	regulation of spindle organization	Oct-66	0.000417632	0.002575806
GO:0110110	positive regulation of animal organ morphogenesis	Oct-66	0.000417632	0.002575806
GO:2000249	regulation of actin cytoskeleton reorganization	Oct-66	0.000417632	0.002575806
GO:0003094	glomerular filtration	Aug-66	0.000418427	0.002575806
GO:0032148	activation of protein kinase B activity	Aug-66	0.000418427	0.002575806
GO:0090077	foam cell differentiation	Aug-66	0.000418427	0.002575806
GO:1903055	positive regulation of extracellular matrix organization	Aug-66	0.000418427	0.002575806
GO:0048813	dendrite morphogenesis	26/1966	0.000428615	0.002635779
GO:0045580	regulation of T cell differentiation	24/1966	0.000430043	0.002641819
GO:0035025	positive regulation of Rho protein signal transduction	Sep-66	0.000437585	0.002685367
GO:0022600	digestive system process	19/1966	0.000441395	0.002698987
GO:0045666	positive regulation of neuron differentiation	19/1966	0.000441395	0.002698987
GO:0038065	collagen-activated signaling pathway	Jun-66	0.000441503	0.002698987
GO:0009410	response to xenobiotic stimulus	39/1966	0.000441628	0.002698987
GO:0032984	protein-containing complex disassembly	31/1966	0.000442367	0.002700718

GO:0002720	positive regulation of cytokine production involved in immune response	13/1966	0.000450823	0.002745738
GO:0055021	regulation of cardiac muscle tissue growth	15/1966	0.000451132	0.002745738
GO:0072078	nephron tubule morphogenesis	15/1966	0.000451132	0.002745738
GO:0001823	mesonephros development	18/1966	0.000455554	0.002766966
GO:1901800	positive regulation of proteasomal protein catabolic process	18/1966	0.000455554	0.002766966
GO:0007249	I-kappaB kinase/NF-kappaB signaling	30/1966	0.000458223	0.002780327
GO:0021915	neural tube development	27/1966	0.000458888	0.002781509
GO:0032465	regulation of cytokinesis	17/1966	0.000463356	0.002795025
GO:0060021	roof of mouth development	17/1966	0.000463356	0.002795025
GO:0090630	activation of GTPase activity	17/1966	0.000463356	0.002795025
GO:0010743	regulation of macrophage derived foam cell differentiation	Jul-66	0.000466309	0.002795025
GO:0035634	response to stilbenoid	Jul-66	0.000466309	0.002795025
GO:0048486	parasympathetic nervous system development	Jul-66	0.000466309	0.002795025
GO:0060438	trachea development	Jul-66	0.000466309	0.002795025
GO:0060602	branch elongation of an epithelium	Jul-66	0.000466309	0.002795025
GO:0060749	mammary gland alveolus development	Jul-66	0.000466309	0.002795025
GO:0061377	mammary gland lobule development	Jul-66	0.000466309	0.002795025
GO:0090026	positive regulation of monocyte chemotaxis	Jul-66	0.000466309	0.002795025

GO:2000278	regulation of DNA biosynthetic process	20/1966	0.000468701	0.002806519
GO:2000116	regulation of cysteine-type endopeptidase activity	31/1966	0.000475455	0.002844085
GO:0032908	regulation of transforming growth factor beta1 production	May-66	0.000479718	0.00285804
GO:0034115	negative regulation of heterotypic cell-cell adhesion	May-66	0.000479718	0.00285804
GO:0061517	macrophage proliferation	May-66	0.000479718	0.00285804
GO:0070587	regulation of cell-cell adhesion involved in gastrulation	May-66	0.000479718	0.00285804
GO:0030307	positive regulation of cell growth	28/1966	0.000485994	0.002892518
GO:2000177	regulation of neural precursor cell proliferation	19/1966	0.000491444	0.00292011
GO:0051149	positive regulation of muscle cell differentiation	14/1966	0.000491616	0.00292011
GO:0002366	leukocyte activation involved in immune response	39/1966	0.000498557	0.002958372
GO:0065004	protein-DNA complex assembly	22/1966	0.000502458	0.002978534
GO:1902106	negative regulation of leukocyte differentiation	18/1966	0.000509614	0.003016573
GO:0008217	regulation of blood pressure	29/1966	0.000509894	0.003016573
GO:0045823	positive regulation of heart contraction	Oct-66	0.000511242	0.003021532
GO:0006809	nitric oxide biosynthetic process	15/1966	0.000515065	0.003032024
GO:0032272	negative regulation of protein polymerization	15/1966	0.000515065	0.003032024
GO:0043154	negative regulation of cysteine-type endopeptidase activity involved in apoptotic process	15/1966	0.000515065	0.003032024
GO:0072089	stem cell proliferation	15/1966	0.000515065	0.003032024

GO:0002698	negative regulation of immune effector process	20/1966	0.000519006	0.003052189
GO:0060395	SMAD protein signal transduction	17/1966	0.000521242	0.003062294
GO:0043535	regulation of blood vessel endothelial cell migration	16/1966	0.000523964	0.00307204
GO:0150076	neuroinflammatory response	13/1966	0.000524874	0.00307204
GO:1990874	vascular associated smooth muscle cell proliferation	13/1966	0.000524874	0.00307204
GO:0043281	regulation of cysteine-type endopeptidase activity involved in apoptotic process	28/1966	0.000524975	0.00307204
GO:0007548	sex differentiation	38/1966	0.000529472	0.003095293
GO:0045332	phospholipid translocation	Nov-66	0.00054366	0.003175104
GO:0045599	negative regulation of fat cell differentiation	Dec-66	0.000544907	0.003179249
GO:0034122	negative regulation of toll-like receptor signaling pathway	Sep-66	0.000548434	0.003187268
GO:0040001	establishment of mitotic spindle localization	Sep-66	0.000548434	0.003187268
GO:0051491	positive regulation of filopodium assembly	Sep-66	0.000548434	0.003187268
GO:1904037	positive regulation of epithelial cell apoptotic process	Sep-66	0.000548434	0.003187268
GO:1904029	regulation of cyclin-dependent protein kinase activity	14/1966	0.000565323	0.003282197
GO:0055017	cardiac muscle tissue growth	18/1966	0.000569118	0.003300998
GO:0060401	cytosolic calcium ion transport	27/1966	0.000581906	0.003371868
GO:0060993	kidney morphogenesis	17/1966	0.000585236	0.003387845
GO:0048469	cell maturation	29/1966	0.000591607	0.003414094

GO:1903828	negative regulation of protein localization	29/1966	0.000591607	0.003414094
GO:0006970	response to osmotic stress	16/1966	0.000592076	0.003414094
GO:0045103	intermediate filament-based process	16/1966	0.000592076	0.003414094
GO:0032508	DNA duplex unwinding	Oct-66	0.000621582	0.00358075
GO:0051235	maintenance of location	40/1966	0.000628472	0.003612306
GO:0002695	negative regulation of leukocyte activation	27/1966	0.00062889	0.003612306
GO:0098656	anion transmembrane transport	27/1966	0.00062889	0.003612306
GO:0035089	establishment of apical/basal cell polarity	Jul-66	0.000631064	0.003614275
GO:0036119	response to platelet-derived growth factor	Jul-66	0.000631064	0.003614275
GO:1902894	negative regulation of miRNA transcription	Jul-66	0.000631064	0.003614275
GO:1903707	negative regulation of hemopoiesis	18/1966	0.00063451	0.003630499
GO:0030042	actin filament depolymerization	Dec-66	0.000639389	0.003644335
GO:0044380	protein localization to cytoskeleton	Dec-66	0.000639389	0.003644335
GO:0050922	negative regulation of chemotaxis	Dec-66	0.000639389	0.003644335
GO:0098815	modulation of excitatory postsynaptic potential	Dec-66	0.000639389	0.003644335
GO:0061308	cardiac neural crest cell development involved in heart development	Jun-66	0.00064306	0.003661732
GO:0030857	negative regulation of epithelial cell differentiation	Nov-66	0.000647516	0.003676501
GO:0090279	regulation of calcium ion import	Nov-66	0.000647516	0.003676501

GO:1902041	regulation of extrinsic apoptotic signaling pathway via death domain receptors	Nov-66	0.000647516	0.003676501
GO:0051153	regulation of striated muscle cell differentiation	17/1966	0.000655856	0.003720286
GO:0007204	positive regulation of cytosolic calcium ion concentration	41/1966	0.00065937	0.00373664
GO:0046328	regulation of JNK cascade	22/1966	0.000662431	0.003750397
GO:0046785	microtubule polymerization	15/1966	0.000666372	0.003769107
GO:0045685	regulation of glial cell differentiation	16/1966	0.000667618	0.00377255
GO:0006469	negative regulation of protein kinase activity	27/1966	0.00067916	0.003834116
GO:0090313	regulation of protein targeting to membrane	Sep-66	0.000681262	0.003842318
GO:0090025	regulation of monocyte chemotaxis	Aug-66	0.000697186	0.003928387
GO:0003158	endothelium development	20/1966	0.000699002	0.003934877
GO:0045668	negative regulation of osteoblast differentiation	13/1966	0.000704113	0.003952379
GO:0060425	lung morphogenesis	13/1966	0.000704113	0.003952379
GO:0150116	regulation of cell-substrate junction organization	13/1966	0.000704113	0.003952379
GO:0042471	ear morphogenesis	21/1966	0.000716147	0.004016117
GO:0034612	response to tumor necrosis factor	27/1966	0.000732908	0.004106225
GO:0051651	maintenance of location in cell	29/1966	0.000735725	0.004118115
GO:0032677	regulation of interleukin-8 production	14/1966	0.000741137	0.00414058
GO:0043627	response to estrogen	14/1966	0.000741137	0.00414058

GO:0046620	regulation of organ growth	19/1966	0.000743914	0.004152176
GO:0042551	neuron maturation	Dec-66	0.000747221	0.004166704
GO:0060411	cardiac septum morphogenesis	15/1966	0.000755212	0.004207305
GO:0002819	regulation of adaptive immune response	30/1966	0.000757332	0.004215153
GO:0002042	cell migration involved in sprouting angiogenesis	Nov-66	0.00076736	0.004262951
GO:0034204	lipid translocation	Nov-66	0.00076736	0.004262951
GO:0000018	regulation of DNA recombination	20/1966	0.000769907	0.004269093
GO:0010212	response to ionizing radiation	20/1966	0.000769907	0.004269093
GO:0002246	wound healing involved in inflammatory response	May-66	0.000776213	0.004276031
GO:0007183	SMAD protein complex assembly	May-66	0.000776213	0.004276031
GO:0021561	facial nerve development	May-66	0.000776213	0.004276031
GO:0021610	facial nerve morphogenesis	May-66	0.000776213	0.004276031
GO:0038063	collagen-activated tyrosine kinase receptor signaling pathway	May-66	0.000776213	0.004276031
GO:0060623	regulation of chromosome condensation	May-66	0.000776213	0.004276031
GO:0070586	cell-cell adhesion involved in gastrulation	May-66	0.000776213	0.004276031
GO:0035107	appendage morphogenesis	24/1966	0.00078348	0.004308051
GO:0035108	limb morphogenesis	24/1966	0.00078348	0.004308051
GO:0006665	sphingolipid metabolic process	21/1966	0.000785526	0.004315294

GO:0042098	T cell proliferation	29/1966	0.00079016	0.004336727
GO:0042593	glucose homeostasis	35/1966	0.000808294	0.004432144
GO:0006949	syncytium formation	13/1966	0.00081148	0.004445501
GO:0002702	positive regulation of production of molecular mediator of immune response	19/1966	0.000822165	0.004499872
GO:1903039	positive regulation of leukocyte cell-cell adhesion	31/1966	0.000828839	0.00453221
GO:0002693	positive regulation of cellular extravasation	Jul-66	0.00083888	0.004563624
GO:0030903	notochord development	Jul-66	0.00083888	0.004563624
GO:0001953	negative regulation of cell-matrix adhesion	Sep-66	0.000839208	0.004563624
GO:0044786	cell cycle DNA replication	Sep-66	0.000839208	0.004563624
GO:0060236	regulation of mitotic spindle organization	Sep-66	0.000839208	0.004563624
GO:0098868	bone growth	Sep-66	0.000839208	0.004563624
GO:0032637	interleukin-8 production	14/1966	0.000845087	0.004582972
GO:0070192	chromosome organization involved in meiotic cell cycle	14/1966	0.000845087	0.004582972
GO:0071277	cellular response to calcium ion	14/1966	0.000845087	0.004582972
GO:0006942	regulation of striated muscle contraction	15/1966	0.000853899	0.004626522
GO:0033500	carbohydrate homeostasis	35/1966	0.000859448	0.00465233
GO:0010972	negative regulation of G2/M transition of mitotic cell cycle	Dec-66	0.000869834	0.004692473
GO:0060612	adipose tissue development	Dec-66	0.000869834	0.004692473

GO:1903078	positive regulation of protein localization to plasma membrane	Dec-66	0.000869834	0.004692473
GO:0098659	inorganic cation import across plasma membrane	18/1966	0.000870825	0.004692473
GO:0099587	inorganic ion import across plasma membrane	18/1966	0.000870825	0.004692473
GO:0035066	positive regulation of histone acetylation	Aug-66	0.000883992	0.004741851
GO:0043552	positive regulation of phosphatidylinositol 3-kinase activity	Aug-66	0.000883992	0.004741851
GO:0061037	negative regulation of cartilage development	Aug-66	0.000883992	0.004741851
GO:0086010	membrane depolarization during action potential	Aug-66	0.000883992	0.004741851
GO:1903319	positive regulation of protein maturation	Aug-66	0.000883992	0.004741851
GO:0030279	negative regulation of ossification	Oct-66	0.000901481	0.00482012
GO:0055023	positive regulation of cardiac muscle tissue growth	Oct-66	0.000901481	0.00482012
GO:0007019	microtubule depolymerization	Nov-66	0.000905034	0.00482012
GO:0035088	establishment or maintenance of apical/basal cell polarity	Nov-66	0.000905034	0.00482012
GO:0035272	exocrine system development	Nov-66	0.000905034	0.00482012
GO:0061245	establishment or maintenance of bipolar cell polarity	Nov-66	0.000905034	0.00482012
GO:1903052	positive regulation of proteolysis involved in cellular protein catabolic process	19/1966	0.000907376	0.00482012
GO:0007614	short-term memory	Jun-66	0.000909165	0.00482012
GO:0060192	negative regulation of lipase activity	Jun-66	0.000909165	0.00482012
GO:0061307	cardiac neural crest cell differentiation involved in heart development	Jun-66	0.000909165	0.00482012

GO:0061318	renal filtration cell differentiation	Jun-66	0.000909165	0.00482012
GO:0072112	glomerular visceral epithelial cell differentiation	Jun-66	0.000909165	0.00482012
GO:0072311	glomerular epithelial cell differentiation	Jun-66	0.000909165	0.00482012
GO:0008277	regulation of G protein-coupled receptor signaling pathway	20/1966	0.000930468	0.004928652
GO:1902895	positive regulation of miRNA transcription	13/1966	0.000932257	0.004933714
GO:0033135	regulation of peptidyl-serine phosphorylation	23/1966	0.000936208	0.004950198
GO:0032091	negative regulation of protein binding	16/1966	0.00094545	0.004994607
GO:2000573	positive regulation of DNA biosynthetic process	14/1966	0.000961059	0.005072543
GO:0010517	regulation of phospholipase activity	Dec-66	0.001008764	0.005314858
GO:0031424	keratinization	Dec-66	0.001008764	0.005314858
GO:0048839	inner ear development	28/1966	0.001018809	0.00536301
GO:0050773	regulation of dendrite development	20/1966	0.001020993	0.005369732
GO:0030705	cytoskeleton-dependent intracellular transport	26/1966	0.001022179	0.0053712
GO:0002931	response to ischemia	Sep-66	0.001025666	0.005379976
GO:0043304	regulation of mast cell degranulation	Sep-66	0.001025666	0.005379976
GO:0010469	regulation of signaling receptor activity	21/1966	0.001029557	0.005395609
GO:0034605	cellular response to heat	Nov-66	0.001062511	0.005563387
GO:0007405	neuroblast proliferation	13/1966	0.001067723	0.005585737

GO:0030835	negative regulation of actin filament depolymerization	Oct-66	0.001076002	0.005619121
GO:0043114	regulation of vascular permeability	Oct-66	0.001076002	0.005619121
GO:0045665	negative regulation of neuron differentiation	15/1966	0.001084275	0.005657336
GO:0002822	regulation of adaptive immune response based on somatic recombination of immune receptors built from immunoglobulin superfamily domains	28/1966	0.001093026	0.005693294
GO:0002719	negative regulation of cytokine production involved in immune response	Jul-66	0.001096935	0.005693294
GO:0007413	axonal fasciculation	Jul-66	0.001096935	0.005693294
GO:0061162	establishment of monopolar cell polarity	Jul-66	0.001096935	0.005693294
GO:0106030	neuron projection fasciculation	Jul-66	0.001096935	0.005693294
GO:1905820	positive regulation of chromosome separation	Jul-66	0.001096935	0.005693294
GO:0000132	establishment of mitotic spindle orientation	Aug-66	0.00110887	0.005735134
GO:0034260	negative regulation of GTPase activity	Aug-66	0.00110887	0.005735134
GO:0051150	regulation of smooth muscle cell differentiation	Aug-66	0.00110887	0.005735134
GO:0060977	coronary vasculature morphogenesis	Aug-66	0.00110887	0.005735134
GO:0045667	regulation of osteoblast differentiation	22/1966	0.001118516	0.005779975
GO:0071901	negative regulation of protein serine/threonine kinase activity	17/1966	0.00112868	0.005827414
GO:1902750	negative regulation of cell cycle G2/M phase transition	Dec-66	0.001165643	0.00600778
GO:1904705	regulation of vascular associated smooth muscle cell proliferation	Dec-66	0.001165643	0.00600778
GO:0007623	circadian rhythm	28/1966	0.001171891	0.00603473

GO:0051209	release of sequestered calcium ion into cytosol	18/1966	0.001178639	0.006064207
GO:0033625	positive regulation of integrin activation	May-66	0.001190675	0.00609436
GO:0044557	relaxation of smooth muscle	May-66	0.001190675	0.00609436
GO:0051639	actin filament network formation	May-66	0.001190675	0.00609436
GO:0060394	negative regulation of pathway-restricted SMAD protein phosphorylation	May-66	0.001190675	0.00609436
GO:0072393	microtubule anchoring at microtubule organizing center	May-66	0.001190675	0.00609436
GO:1901678	iron coordination entity transport	May-66	0.001190675	0.00609436
GO:0051100	negative regulation of binding	23/1966	0.001196951	0.006121197
GO:0015914	phospholipid transport	15/1966	0.00121782	0.006213696
GO:0032436	positive regulation of proteasomal ubiquitin-dependent protein catabolic process	15/1966	0.00121782	0.006213696
GO:0032732	positive regulation of interleukin-1 production	13/1966	0.001219236	0.006213696
GO:0060761	negative regulation of response to cytokine stimulus	13/1966	0.001219236	0.006213696
GO:0034341	response to interferon-gamma	20/1966	0.001224861	0.006236998
GO:0055007	cardiac muscle cell differentiation	21/1966	0.001226203	0.006238466
GO:0044774	mitotic DNA integrity checkpoint signaling	14/1966	0.001233424	0.006264441
GO:0051279	regulation of release of sequestered calcium ion into cytosol	14/1966	0.001233424	0.006264441
GO:0001974	blood vessel remodeling	Nov-66	0.0012419	0.0062913
GO:0035019	somatic stem cell population maintenance	Nov-66	0.0012419	0.0062913

GO:0072698	protein localization to microtubule cytoskeleton	Nov-66	0.0012419	0.0062913
GO:0003214	cardiac left ventricle morphogenesis	Jun-66	0.001252539	0.00630207
GO:0010544	negative regulation of platelet activation	Jun-66	0.001252539	0.00630207
GO:0035313	wound healing, spreading of epidermal cells	Jun-66	0.001252539	0.00630207
GO:0040037	negative regulation of fibroblast growth factor receptor signaling pathway	Jun-66	0.001252539	0.00630207
GO:0045198	establishment of epithelial cell apical/basal polarity	Jun-66	0.001252539	0.00630207
GO:0045837	negative regulation of membrane potential	Jun-66	0.001252539	0.00630207
GO:0071636	positive regulation of transforming growth factor beta production	Jun-66	0.001252539	0.00630207
GO:1905709	negative regulation of membrane permeability	Jun-66	0.001252539	0.00630207
GO:0042445	hormone metabolic process	28/1966	0.001255644	0.006312327
GO:0001941	postsynaptic membrane organization	Oct-66	0.001277179	0.006415146
GO:0009266	response to temperature stimulus	23/1966	0.001296726	0.006507237
GO:0018107	peptidyl-threonine phosphorylation	18/1966	0.001299909	0.006507237
GO:0051283	negative regulation of sequestering of calcium ion	18/1966	0.001299909	0.006507237
GO:0098773	skin epidermis development	18/1966	0.001299909	0.006507237
GO:0050772	positive regulation of axonogenesis	16/1966	0.001315693	0.00658069
GO:0043122	regulation of I-kappaB kinase/NF-kappaB signaling	25/1966	0.001322487	0.006609088
GO:0051048	negative regulation of secretion	26/1966	0.001370299	0.00684225

GO:0033598	mammary gland epithelial cell proliferation	Aug-66	0.001377132	0.006847501
GO:0040036	regulation of fibroblast growth factor receptor signaling pathway	Aug-66	0.001377132	0.006847501
GO:0048566	embryonic digestive tract development	Aug-66	0.001377132	0.006847501
GO:1904646	cellular response to amyloid-beta	Aug-66	0.001377132	0.006847501
GO:1905314	semi-lunar valve development	Aug-66	0.001377132	0.006847501
GO:0010522	regulation of calcium ion transport into cytosol	17/1966	0.001385936	0.0068796
GO:0030032	lamellipodium assembly	13/1966	0.001388234	0.0068796
GO:0032623	interleukin-2 production	13/1966	0.001388234	0.0068796
GO:0060675	ureteric bud morphogenesis	13/1966	0.001388234	0.0068796
GO:0008625	extrinsic apoptotic signaling pathway via death domain receptors	14/1966	0.00139215	0.00688748
GO:0033209	tumor necrosis factor-mediated signaling pathway	14/1966	0.00139215	0.00688748
GO:1902115	regulation of organelle assembly	27/1966	0.001408858	0.006958096
GO:0006582	melanin metabolic process	Jul-66	0.001413473	0.006958096
GO:0010742	macrophage derived foam cell differentiation	Jul-66	0.001413473	0.006958096
GO:0051307	meiotic chromosome separation	Jul-66	0.001413473	0.006958096
GO:0061339	establishment or maintenance of monopolar cell polarity	Jul-66	0.001413473	0.006958096
GO:0071276	cellular response to cadmium ion	Jul-66	0.001413473	0.006958096
GO:0009749	response to glucose	25/1966	0.001424073	0.007004451

GO:0098754	detoxification	Nov-66	0.00144544	0.007097755
GO:1903533	regulation of protein targeting	Nov-66	0.00144544	0.007097755
GO:0010657	muscle cell apoptotic process	16/1966	0.001463498	0.007174535
GO:0045185	maintenance of protein location	16/1966	0.001463498	0.007174535
GO:0006575	cellular modified amino acid metabolic process	26/1966	0.001471822	0.00720937
GO:0051693	actin filament capping	Sep-66	0.001498981	0.007336334
GO:0033173	calcineurin-NFAT signaling cascade	Oct-66	0.001507946	0.007368035
GO:0060421	positive regulation of heart growth	Oct-66	0.001507946	0.007368035
GO:0030168	platelet activation	15/1966	0.001526649	0.007441001
GO:0045104	intermediate filament cytoskeleton organization	15/1966	0.001526649	0.007441001
GO:0048814	regulation of dendrite morphogenesis	15/1966	0.001526649	0.007441001
GO:0048708	astrocyte differentiation	14/1966	0.001567567	0.007634156
GO:0010769	regulation of cell morphogenesis involved in differentiation	18/1966	0.00157442	0.007638675
GO:0034754	cellular hormone metabolic process	18/1966	0.00157442	0.007638675
GO:0045621	positive regulation of lymphocyte differentiation	18/1966	0.00157442	0.007638675
GO:0051282	regulation of sequestering of calcium ion	18/1966	0.00157442	0.007638675
GO:0002886	regulation of myeloid leukocyte mediated immunity	13/1966	0.001576235	0.007638675
GO:0072171	mesonephric tubule morphogenesis	13/1966	0.001576235	0.007638675

GO:0048736	appendage development	26/1966	0.001579753	0.007643217
GO:0060173	limb development	26/1966	0.001579753	0.007643217
GO:0015698	inorganic anion transport	21/1966	0.001581066	0.007643327
GO:1903531	negative regulation of secretion by cell	23/1966	0.001640008	0.007921798
GO:0031668	cellular response to extracellular stimulus	29/1966	0.001663616	0.008029287
GO:0001523	retinoid metabolic process	Nov-66	0.001675504	0.008080079
GO:0060294	cilium movement involved in cell motility	22/1966	0.001683591	0.008095129
GO:0010804	negative regulation of tumor necrosis factor-mediated signaling pathway	Jun-66	0.001686827	0.008095129
GO:0033599	regulation of mammary gland epithelial cell proliferation	Jun-66	0.001686827	0.008095129
GO:0036120	cellular response to platelet-derived growth factor stimulus	Jun-66	0.001686827	0.008095129
GO:1902430	negative regulation of amyloid-beta formation	Jun-66	0.001686827	0.008095129
GO:1904754	positive regulation of vascular associated smooth muscle cell migration	Jun-66	0.001686827	0.008095129
GO:0045639	positive regulation of myeloid cell differentiation	17/1966	0.001691052	0.008108836
GO:0010758	regulation of macrophage chemotaxis	Aug-66	0.001694454	0.008112011
GO:1901186	positive regulation of ERBB signaling pathway	Aug-66	0.001694454	0.008112011
GO:0120193	tight junction organization	15/1966	0.0017041	0.008145019
GO:2000117	negative regulation of cysteine-type endopeptidase activity	15/1966	0.0017041	0.008145019
GO:0032368	regulation of lipid transport	18/1966	0.001729095	0.008257823

GO:0002237	response to molecule of bacterial origin	44/1966	0.001744959	0.008297345
GO:0030206	chondroitin sulfate biosynthetic process	May-66	0.001748579	0.008297345
GO:0035791	platelet-derived growth factor receptor-beta signaling pathway	May-66	0.001748579	0.008297345
GO:0060670	branching involved in labyrinthine layer morphogenesis	May-66	0.001748579	0.008297345
GO:0061309	cardiac neural crest cell development involved in outflow tract morphogenesis	May-66	0.001748579	0.008297345
GO:0072224	metanephric glomerulus development	May-66	0.001748579	0.008297345
GO:0098840	protein transport along microtubule	May-66	0.001748579	0.008297345
GO:0099118	microtubule-based protein transport	May-66	0.001748579	0.008297345
GO:0042440	pigment metabolic process	Dec-66	0.001761838	0.008353567
GO:0009746	response to hexose	25/1966	0.00177007	0.008378841
GO:0010862	positive regulation of pathway-restricted SMAD protein phosphorylation	Oct-66	0.001771413	0.008378841
GO:0090102	cochlea development	Oct-66	0.001771413	0.008378841
GO:1901184	regulation of ERBB signaling pathway	13/1966	0.001784835	0.008435591
GO:0001774	microglial cell activation	Sep-66	0.001793894	0.008439068
GO:0010803	regulation of tumor necrosis factor-mediated signaling pathway	Sep-66	0.001793894	0.008439068
GO:0033006	regulation of mast cell activation involved in immune response	Sep-66	0.001793894	0.008439068
GO:0045066	regulatory T cell differentiation	Sep-66	0.001793894	0.008439068
GO:0007274	neuromuscular synaptic transmission	Jul-66	0.001796972	0.008439068

GO:0031100	animal organ regeneration	Jul-66	0.001796972	0.008439068
GO:0032703	negative regulation of interleukin-2 production	Jul-66	0.001796972	0.008439068
GO:0070841	inclusion body assembly	Jul-66	0.001796972	0.008439068
GO:0010770	positive regulation of cell morphogenesis involved in differentiation	16/1966	0.001801229	0.008452361
GO:0071356	cellular response to tumor necrosis factor	24/1966	0.00184242	0.008638805
GO:0009101	glycoprotein biosynthetic process	32/1966	0.001888283	0.008846845
GO:0071241	cellular response to inorganic substance	29/1966	0.001890237	0.008848999
GO:0015718	monocarboxylic acid transport	18/1966	0.00189639	0.00886379
GO:0051208	sequestering of calcium ion	18/1966	0.00189639	0.00886379
GO:0003170	heart valve development	Nov-66	0.0019346	0.008999764
GO:0043388	positive regulation of DNA binding	Nov-66	0.0019346	0.008999764
GO:0050775	positive regulation of dendrite morphogenesis	Nov-66	0.0019346	0.008999764
GO:0051155	positive regulation of striated muscle cell differentiation	Nov-66	0.0019346	0.008999764
GO:0051445	regulation of meiotic cell cycle	Nov-66	0.0019346	0.008999764
GO:0097035	regulation of membrane lipid distribution	Nov-66	0.0019346	0.008999764
GO:0070665	positive regulation of leukocyte proliferation	22/1966	0.001969706	0.009155887
GO:0006643	membrane lipid metabolic process	24/1966	0.001981619	0.009204038
GO:0000768	syncytium formation by plasma membrane fusion	Dec-66	0.002008885	0.009301497

GO:0030837	negative regulation of actin filament polymerization	Dec-66	0.002008885	0.009301497
GO:0044458	motile cilium assembly	Dec-66	0.002008885	0.009301497
GO:0140253	cell-cell fusion	Dec-66	0.002008885	0.009301497
GO:1903076	regulation of protein localization to plasma membrane	17/1966	0.002050783	0.009488075
GO:0001702	gastrulation with mouth forming second	Aug-66	0.00206686	0.009506716
GO:0003401	axis elongation	Aug-66	0.00206686	0.009506716
GO:0010667	negative regulation of cardiac muscle cell apoptotic process	Aug-66	0.00206686	0.009506716
GO:0090218	positive regulation of lipid kinase activity	Aug-66	0.00206686	0.009506716
GO:1904738	vascular associated smooth muscle cell migration	Aug-66	0.00206686	0.009506716
GO:0006687	glycosphingolipid metabolic process	Oct-66	0.002070865	0.009506716
GO:0007520	myoblast fusion	Oct-66	0.002070865	0.009506716
GO:0032392	DNA geometric change	Oct-66	0.002070865	0.009506716
GO:0044818	mitotic G2/M transition checkpoint	Oct-66	0.002070865	0.009506716
GO:1904407	positive regulation of nitric oxide metabolic process	Oct-66	0.002070865	0.009506716
GO:0032388	positive regulation of intracellular transport	24/1966	0.002129685	0.009763609
GO:0032924	activin receptor signaling pathway	Sep-66	0.00213342	0.009763609
GO:0048873	homeostasis of number of cells within a tissue	Sep-66	0.00213342	0.009763609
GO:0086002	cardiac muscle cell action potential involved in contraction	Sep-66	0.00213342	0.009763609

GO:0031348	negative regulation of defense response	29/1966	0.002142909	0.009799463
GO:0097193	intrinsic apoptotic signaling pathway	35/1966	0.002161352	0.009876179
GO:0034284	response to monosaccharide	25/1966	0.002185648	0.009979498

GO terms associated with 4473 Rep_intermed_enriched_vs_Hep genes

ID	Description	Gene Ratio	pvalue	p.adjust
GO:0007015	actin filament organization	172/3805	5.01E-40	1.82E-36
GO:0044782	cilium organization	155/3805	5.82E-40	1.82E-36
GO:0060271	cilium assembly	140/3805	1.79E-35	3.73E-32
GO:0032970	regulation of actin filament-based process	153/3805	8.91E-35	1.39E-31
GO:1902903	regulation of supramolecular fiber organization	146/3805	3.01E-34	3.76E-31
GO:0050808	synapse organization	168/3805	6.23E-33	6.49E-30
GO:0034329	cell junction assembly	155/3805	2.60E-32	2.32E-29
GO:0001667	ameboidal-type cell migration	155/3805	6.27E-31	4.90E-28
GO:0030198	extracellular matrix organization	123/3805	7.90E-31	5.48E-28
GO:0043062	extracellular structure organization	123/3805	1.13E-30	7.05E-28
GO:0045229	external encapsulating structure organization	123/3805	1.61E-30	9.13E-28
GO:0007264	small GTPase mediated signal transduction	149/3805	3.28E-30	1.71E-27
GO:0007409	axonogenesis	162/3805	7.66E-30	3.68E-27
GO:0032956	regulation of actin cytoskeleton organization	133/3805	1.54E-29	6.89E-27
GO:0060562	epithelial tube morphogenesis	138/3805	1.35E-28	5.63E-26
GO:0048762	mesenchymal cell differentiation	99/3805	6.13E-27	2.39E-24

GO:0060485	mesenchyme development	112/3805	2.27E-26	8.33E-24
GO:0007018	microtubule-based movement	140/3805	5.87E-26	2.04E-23
GO:0045216	cell-cell junction organization	90/3805	2.66E-25	8.74E-23
GO:0001655	urogenital system development	130/3805	3.46E-25	1.08E-22
GO:0031032	actomyosin structure organization	89/3805	3.85E-25	1.15E-22
GO:0001822	kidney development	117/3805	4.53E-25	1.29E-22
GO:0072001	renal system development	120/3805	7.67E-25	2.08E-22
GO:0031346	positive regulation of cell projection organization	141/3805	1.34E-24	3.50E-22
GO:0033674	positive regulation of kinase activity	139/3805	1.89E-24	4.71E-22
GO:0007178	transmembrane receptor protein serine/threonine kinase signaling pathway	129/3805	3.42E-24	8.20E-22
GO:0010639	negative regulation of organelle organization	124/3805	1.24E-23	2.76E-21
GO:0031589	cell-substrate adhesion	124/3805	1.24E-23	2.76E-21
GO:0071900	regulation of protein serine/threonine kinase activity	119/3805	1.95E-23	4.21E-21
GO:0007265	Ras protein signal transduction	116/3805	5.16E-23	1.07E-20
GO:0043254	regulation of protein-containing complex assembly	134/3805	1.33E-22	2.68E-20
GO:0045860	positive regulation of protein kinase activity	118/3805	3.32E-22	6.48E-20
GO:0090287	regulation of cellular response to growth factor stimulus	105/3805	3.82E-22	7.22E-20
GO:0022604	regulation of cell morphogenesis	113/3805	7.02E-22	1.29E-19

GO:0060560	developmental growth involved in morphogenesis	100/3805	9.13E-22	1.63E-19
GO:0016358	dendrite development	108/3805	1.08E-21	1.83E-19
GO:0090130	tissue migration	108/3805	1.08E-21	1.83E-19
GO:0010631	epithelial cell migration	107/3805	1.24E-21	2.04E-19
GO:0050767	regulation of neurogenesis	137/3805	1.89E-21	3.03E-19
GO:0090132	epithelium migration	107/3805	2.16E-21	3.38E-19
GO:0051056	regulation of small GTPase mediated signal transduction	88/3805	2.67E-21	4.07E-19
GO:0110053	regulation of actin filament organization	98/3805	2.81E-21	4.18E-19
GO:0045785	positive regulation of cell adhesion	142/3805	3.29E-21	4.79E-19
GO:0071559	response to transforming growth factor beta	87/3805	3.99E-21	5.66E-19
GO:0071560	cellular response to transforming growth factor beta stimulus	86/3805	4.24E-21	5.89E-19
GO:0001837	epithelial to mesenchymal transition	68/3805	5.83E-21	7.92E-19
GO:0042692	muscle cell differentiation	132/3805	9.41E-21	1.25E-18
GO:0198738	cell-cell signaling by wnt	135/3805	1.03E-20	1.34E-18
GO:0061572	actin filament bundle organization	73/3805	1.18E-20	1.50E-18
GO:0040013	negative regulation of locomotion	111/3805	1.32E-20	1.65E-18
GO:0051017	actin filament bundle assembly	72/3805	1.70E-20	2.09E-18
GO:0032535	regulation of cellular component size	122/3805	1.89E-20	2.26E-18

GO:0016055	Wnt signaling pathway	134/3805	1.92E-20	2.26E-18
GO:0003007	heart morphogenesis	98/3805	2.92E-20	3.38E-18
GO:0070371	ERK1 and ERK2 cascade	112/3805	4.00E-20	4.54E-18
GO:0007163	establishment or maintenance of cell polarity	85/3805	4.68E-20	5.17E-18
GO:0043087	regulation of GTPase activity	111/3805	4.71E-20	5.17E-18
GO:0001558	regulation of cell growth	135/3805	5.63E-20	6.07E-18
GO:0030010	establishment of cell polarity	65/3805	3.31E-19	3.51E-17
GO:0051258	protein polymerization	94/3805	4.72E-19	4.91E-17
GO:0010632	regulation of epithelial cell migration	86/3805	5.25E-19	5.38E-17
GO:0097485	neuron projection guidance	90/3805	5.98E-19	6.02E-17
GO:1902905	positive regulation of supramolecular fiber organization	72/3805	7.59E-19	7.52E-17
GO:0000280	nuclear division	129/3805	9.60E-19	9.37E-17
GO:1901342	regulation of vasculature development	101/3805	1.08E-18	1.04E-16
GO:0070372	regulation of ERK1 and ERK2 cascade	104/3805	1.40E-18	1.33E-16
GO:0010563	negative regulation of phosphorus metabolic process	131/3805	1.45E-18	1.33E-16
GO:0045936	negative regulation of phosphate metabolic process	131/3805	1.45E-18	1.33E-16
GO:0001763	morphogenesis of a branching structure	87/3805	1.54E-18	1.39E-16
GO:0007411	axon guidance	89/3805	1.60E-18	1.43E-16

GO:0003012	muscle system process	125/3805	2.60E-18	2.28E-16
GO:0090092	regulation of transmembrane receptor protein serine/threonine kinase signaling pathway	88/3805	3.22E-18	2.79E-16
GO:0007224	smoothened signaling pathway	65/3805	3.65E-18	3.12E-16
GO:0045765	regulation of angiogenesis	99/3805	4.09E-18	3.45E-16
GO:0051495	positive regulation of cytoskeleton organization	76/3805	4.72E-18	3.93E-16
GO:0051271	negative regulation of cellular component movement	100/3805	7.31E-18	6.01E-16
GO:0071902	positive regulation of protein serine/threonine kinase activity	76/3805	8.99E-18	7.29E-16
GO:1902904	negative regulation of supramolecular fiber organization	69/3805	1.02E-17	8.20E-16
GO:0060537	muscle tissue development	136/3805	1.14E-17	9.04E-16
GO:0022407	regulation of cell-cell adhesion	131/3805	1.25E-17	9.74E-16
GO:0032271	regulation of protein polymerization	76/3805	1.69E-17	1.26E-15
GO:0099111	microtubule-based transport	76/3805	1.69E-17	1.26E-15
GO:0007389	pattern specification process	135/3805	1.69E-17	1.26E-15
GO:0001578	microtubule bundle formation	55/3805	1.70E-17	1.26E-15
GO:0001654	eye development	121/3805	2.38E-17	1.75E-15
GO:0048588	developmental cell growth	91/3805	2.41E-17	1.75E-15
GO:0031345	negative regulation of cell projection organization	77/3805	2.84E-17	2.03E-15
GO:0034765	regulation of ion transmembrane transport	136/3805	2.86E-17	2.03E-15

GO:0007179	transforming growth factor beta receptor signaling pathway	70/3805	3.81E-17	2.67E-15
GO:0150063	visual system development	121/3805	4.34E-17	3.01E-15
GO:0060070	canonical Wnt signaling pathway	96/3805	6.20E-17	4.25E-15
GO:0051090	regulation of DNA-binding transcription factor activity	123/3805	6.36E-17	4.32E-15
GO:0061448	connective tissue development	94/3805	6.80E-17	4.57E-15
GO:0006936	muscle contraction	98/3805	7.07E-17	4.70E-15
GO:0046578	regulation of Ras protein signal transduction	73/3805	7.86E-17	5.12E-15
GO:0048638	regulation of developmental growth	116/3805	7.86E-17	5.12E-15
GO:0032886	regulation of microtubule-based process	88/3805	8.35E-17	5.37E-15
GO:0051494	negative regulation of cytoskeleton organization	67/3805	9.04E-17	5.76E-15
GO:0048880	sensory system development	121/3805	9.53E-17	6.01E-15
GO:0007346	regulation of mitotic cell cycle	131/3805	9.71E-17	6.06E-15
GO:0030336	negative regulation of cell migration	93/3805	1.03E-16	6.35E-15
GO:0045926	negative regulation of growth	87/3805	1.26E-16	7.73E-15
GO:0050673	epithelial cell proliferation	130/3805	1.43E-16	8.69E-15
GO:0010959	regulation of metal ion transport	124/3805	1.63E-16	9.80E-15
GO:2000146	negative regulation of cell motility	95/3805	2.43E-16	1.44E-14
GO:0045787	positive regulation of cell cycle	105/3805	3.60E-16	2.12E-14

GO:0050678	regulation of epithelial cell proliferation	115/3805	3.88E-16	2.27E-14
GO:0007611	learning or memory	98/3805	4.48E-16	2.58E-14
GO:0048285	organelle fission	133/3805	4.51E-16	2.58E-14
GO:1901888	regulation of cell junction assembly	76/3805	4.55E-16	2.58E-14
GO:0007162	negative regulation of cell adhesion	96/3805	5.13E-16	2.89E-14
GO:0010976	positive regulation of neuron projection development	77/3805	5.31E-16	2.96E-14
GO:0061138	morphogenesis of a branching epithelium	78/3805	6.13E-16	3.39E-14
GO:0070997	neuron death	121/3805	6.39E-16	3.50E-14
GO:0018108	peptidyl-tyrosine phosphorylation	98/3805	7.00E-16	3.80E-14
GO:0030900	forebrain development	113/3805	7.06E-16	3.80E-14
GO:0043405	regulation of MAP kinase activity	71/3805	8.24E-16	4.40E-14
GO:0050890	cognition	105/3805	8.35E-16	4.42E-14
GO:0042326	negative regulation of phosphorylation	113/3805	1.04E-15	5.48E-14
GO:0018212	peptidyl-tyrosine modification	98/3805	1.36E-15	7.06E-14
GO:0030111	regulation of Wnt signaling pathway	96/3805	1.58E-15	8.16E-14
GO:0007160	cell-matrix adhesion	78/3805	1.82E-15	9.30E-14
GO:0050803	regulation of synapse structure or activity	86/3805	1.84E-15	9.34E-14
GO:0060541	respiratory system development	85/3805	2.17E-15	1.09E-13

GO:0042060	wound healing	107/3805	2.35E-15	1.16E-13
GO:0043010	camera-type eye development	107/3805	2.35E-15	1.16E-13
GO:0050807	regulation of synapse organization	84/3805	2.57E-15	1.26E-13
GO:0070507	regulation of microtubule cytoskeleton organization	61/3805	2.74E-15	1.34E-13
GO:0051261	protein depolymerization	52/3805	3.36E-15	1.63E-13
GO:1901879	regulation of protein depolymerization	44/3805	3.91E-15	1.88E-13
GO:0043542	endothelial cell migration	76/3805	4.15E-15	1.98E-13
GO:0010721	negative regulation of cell development	71/3805	4.75E-15	2.25E-13
GO:1903829	positive regulation of protein localization	129/3805	4.86E-15	2.28E-13
GO:0051961	negative regulation of nervous system development	62/3805	4.95E-15	2.31E-13
GO:0007043	cell-cell junction assembly	59/3805	6.09E-15	2.80E-13
GO:0008154	actin polymerization or depolymerization	69/3805	6.10E-15	2.80E-13
GO:0050770	regulation of axonogenesis	66/3805	6.39E-15	2.91E-13
GO:0140014	mitotic nuclear division	87/3805	6.46E-15	2.92E-13
GO:0030323	respiratory tube development	78/3805	6.73E-15	3.03E-13
GO:0002064	epithelial cell development	77/3805	7.86E-15	3.51E-13
GO:0010634	positive regulation of epithelial cell migration	59/3805	8.54E-15	3.78E-13
GO:0048017	inositol lipid-mediated signaling	63/3805	8.62E-15	3.79E-13

GO:0030324	lung development	77/3805	1.02E-14	4.44E-13
GO:0010810	regulation of cell-substrate adhesion	75/3805	1.07E-14	4.62E-13
GO:0003205	cardiac chamber development	69/3805	1.08E-14	4.67E-13
GO:0008360	regulation of cell shape	59/3805	1.19E-14	5.10E-13
GO:0048015	phosphatidylinositol-mediated signaling	62/3805	1.29E-14	5.50E-13
GO:0048732	gland development	125/3805	1.34E-14	5.64E-13
GO:0051146	striated muscle cell differentiation	96/3805	1.36E-14	5.69E-13
GO:0010720	positive regulation of cell development	106/3805	1.37E-14	5.70E-13
GO:0050768	negative regulation of neurogenesis	60/3805	2.12E-14	8.78E-13
GO:0030705	cytoskeleton-dependent intracellular transport	68/3805	2.17E-14	8.93E-13
GO:0043406	positive regulation of MAP kinase activity	52/3805	2.30E-14	9.39E-13
GO:0030177	positive regulation of Wnt signaling pathway	54/3805	3.12E-14	1.27E-12
GO:0051216	cartilage development	71/3805	3.27E-14	1.32E-12
GO:0030308	negative regulation of cell growth	69/3805	3.32E-14	1.32E-12
GO:1990138	neuron projection extension	69/3805	3.32E-14	1.32E-12
GO:0035148	tube formation	64/3805	3.56E-14	1.41E-12
GO:0001764	neuron migration	67/3805	4.35E-14	1.71E-12
GO:0090257	regulation of muscle system process	80/3805	4.45E-14	1.74E-12

GO:0031109	microtubule polymerization or depolymerization	51/3805	4.90E-14	1.89E-12
GO:0048640	negative regulation of developmental growth	51/3805	4.90E-14	1.89E-12
GO:0048608	reproductive structure development	125/3805	4.97E-14	1.90E-12
GO:0001894	tissue homeostasis	86/3805	5.93E-14	2.26E-12
GO:0051098	regulation of binding	108/3805	7.00E-14	2.65E-12
GO:0030048	actin filament-based movement	51/3805	7.07E-14	2.66E-12
GO:0021915	neural tube development	67/3805	7.58E-14	2.83E-12
GO:0001933	negative regulation of protein phosphorylation	99/3805	8.29E-14	3.08E-12
GO:0016331	morphogenesis of embryonic epithelium	64/3805	8.51E-14	3.14E-12
GO:0061458	reproductive system development	125/3805	9.42E-14	3.46E-12
GO:0061351	neural precursor cell proliferation	67/3805	9.96E-14	3.64E-12
GO:0060249	anatomical structure homeostasis	94/3805	1.03E-13	3.74E-12
GO:0001838	embryonic epithelial tube formation	57/3805	1.35E-13	4.89E-12
GO:1990778	protein localization to cell periphery	102/3805	1.40E-13	5.01E-12
GO:0014706	striated muscle tissue development	86/3805	1.41E-13	5.04E-12
GO:1904062	regulation of cation transmembrane transport	104/3805	1.60E-13	5.68E-12
GO:0010594	regulation of endothelial cell migration	61/3805	1.64E-13	5.78E-12
GO:1901214	regulation of neuron death	106/3805	1.81E-13	6.33E-12

GO:0046777	protein autophosphorylation	76/3805	1.81E-13	6.33E-12
GO:0030856	regulation of epithelial cell differentiation	57/3805	1.86E-13	6.44E-12
GO:0043244	regulation of protein-containing complex disassembly	51/3805	2.06E-13	7.11E-12
GO:1903844	regulation of cellular response to transforming growth factor beta stimulus	49/3805	2.21E-13	7.60E-12
GO:0050920	regulation of chemotaxis	73/3805	2.38E-13	8.12E-12
GO:0010970	transport along microtubule	59/3805	2.77E-13	9.36E-12
GO:0072175	epithelial tube formation	59/3805	2.77E-13	9.36E-12
GO:0001503	ossification	111/3805	3.02E-13	1.01E-11
GO:0017015	regulation of transforming growth factor beta receptor signaling pathway	48/3805	3.26E-13	1.09E-11
GO:0007517	muscle organ development	100/3805	3.65E-13	1.21E-11
GO:0007266	Rho protein signal transduction	52/3805	3.85E-13	1.27E-11
GO:0140694	non-membrane-bounded organelle assembly	103/3805	4.04E-13	1.33E-11
GO:1903706	regulation of hemopoiesis	107/3805	4.20E-13	1.37E-11
GO:0030041	actin filament polymerization	59/3805	4.99E-13	1.62E-11
GO:0035082	axoneme assembly	40/3805	5.77E-13	1.86E-11
GO:0043242	negative regulation of protein-containing complex disassembly	40/3805	5.77E-13	1.86E-11
GO:0051403	stress-activated MAPK cascade	76/3805	5.80E-13	1.86E-11
GO:0048754	branching morphogenesis of an epithelial tube	64/3805	5.90E-13	1.88E-11

GO:0030199	collagen fibril organization	31/3805	6.18E-13	1.96E-11
GO:0010717	regulation of epithelial to mesenchymal transition	41/3805	6.27E-13	1.98E-11
GO:0031098	stress-activated protein kinase signaling cascade	78/3805	6.32E-13	1.98E-11
GO:0051962	positive regulation of nervous system development	98/3805	7.94E-13	2.48E-11
GO:2001233	regulation of apoptotic signaling pathway	107/3805	8.29E-13	2.57E-11
GO:1901987	regulation of cell cycle phase transition	106/3805	8.63E-13	2.67E-11
GO:0031110	regulation of microtubule polymerization or depolymerization	41/3805	9.54E-13	2.94E-11
GO:0050679	positive regulation of epithelial cell proliferation	69/3805	9.66E-13	2.96E-11
GO:0042063	gliogenesis	96/3805	9.99E-13	3.03E-11
GO:1901880	negative regulation of protein depolymerization	37/3805	1.00E-12	3.03E-11
GO:0030038	contractile actin filament bundle assembly	46/3805	1.02E-12	3.07E-11
GO:0043149	stress fiber assembly	46/3805	1.02E-12	3.07E-11
GO:0007051	spindle organization	62/3805	1.03E-12	3.08E-11
GO:0006816	calcium ion transport	113/3805	1.20E-12	3.57E-11
GO:0043393	regulation of protein binding	69/3805	1.23E-12	3.65E-11
GO:0050730	regulation of peptidyl-tyrosine phosphorylation	79/3805	1.26E-12	3.70E-11
GO:0009799	specification of symmetry	52/3805	1.42E-12	4.16E-11
GO:0008361	regulation of cell size	68/3805	1.46E-12	4.25E-11

GO:0032409	regulation of transporter activity	85/3805	2.03E-12	5.90E-11
GO:0050900	leukocyte migration	101/3805	2.08E-12	6.01E-11
GO:0071675	regulation of mononuclear cell migration	46/3805	2.10E-12	6.06E-11
GO:0060828	regulation of canonical Wnt signaling pathway	75/3805	2.14E-12	6.12E-11
GO:0003206	cardiac chamber morphogenesis	54/3805	2.16E-12	6.15E-11
GO:0014909	smooth muscle cell migration	43/3805	2.23E-12	6.35E-11
GO:0033002	muscle cell proliferation	77/3805	2.27E-12	6.42E-11
GO:1902850	microtubule cytoskeleton organization involved in mitosis	53/3805	2.40E-12	6.75E-11
GO:0099173	postsynapse organization	66/3805	2.60E-12	7.27E-11
GO:0010977	negative regulation of neuron projection development	56/3805	3.06E-12	8.53E-11
GO:0032231	regulation of actin filament bundle assembly	45/3805	3.12E-12	8.64E-11
GO:0014910	regulation of smooth muscle cell migration	40/3805	3.13E-12	8.64E-11
GO:0014065	phosphatidylinositol 3-kinase signaling	50/3805	3.19E-12	8.79E-11
GO:0002685	regulation of leukocyte migration	70/3805	3.38E-12	9.26E-11
GO:0044772	mitotic cell cycle phase transition	108/3805	3.46E-12	9.42E-11
GO:0007368	determination of left/right symmetry	49/3805	3.47E-12	9.42E-11
GO:0009855	determination of bilateral symmetry	51/3805	3.99E-12	1.08E-10
GO:0001738	morphogenesis of a polarized epithelium	38/3805	4.25E-12	1.14E-10

GO:0001843	neural tube closure	44/3805	4.61E-12	1.24E-10
GO:0051402	neuron apoptotic process	88/3805	5.15E-12	1.37E-10
GO:0001952	regulation of cell-matrix adhesion	48/3805	5.19E-12	1.38E-10
GO:0030099	myeloid cell differentiation	111/3805	5.49E-12	1.45E-10
GO:0050771	negative regulation of axonogenesis	33/3805	5.64E-12	1.49E-10
GO:0007052	mitotic spindle organization	46/3805	5.96E-12	1.56E-10
GO:0060606	tube closure	44/3805	6.59E-12	1.72E-10
GO:0022898	regulation of transmembrane transporter activity	81/3805	6.68E-12	1.74E-10
GO:0006941	striated muscle contraction	58/3805	7.03E-12	1.82E-10
GO:0008064	regulation of actin polymerization or depolymerization	56/3805	7.11E-12	1.83E-10
GO:0031333	negative regulation of protein-containing complex assembly	53/3805	7.89E-12	2.02E-10
GO:0072073	kidney epithelium development	53/3805	7.89E-12	2.02E-10
GO:0007416	synapse assembly	62/3805	8.22E-12	2.09E-10
GO:0009314	response to radiation	109/3805	8.39E-12	2.13E-10
GO:0003341	cilium movement	67/3805	9.15E-12	2.31E-10
GO:0072006	nephron development	54/3805	9.27E-12	2.33E-10
GO:0043547	positive regulation of GTPase activity	75/3805	9.61E-12	2.41E-10
GO:0070374	positive regulation of ERK1 and ERK2 cascade	69/3805	1.01E-11	2.51E-10

GO:0008589	regulation of smoothened signaling pathway	39/3805	1.01E-11	2.51E-10
GO:0048660	regulation of smooth muscle cell proliferation	59/3805	1.02E-11	2.54E-10
GO:0072659	protein localization to plasma membrane	84/3805	1.16E-11	2.87E-10
GO:0090068	positive regulation of cell cycle process	77/3805	1.21E-11	2.97E-10
GO:0014020	primary neural tube formation	45/3805	1.25E-11	3.05E-10
GO:0098742	cell-cell adhesion via plasma-membrane adhesion molecules	66/3805	1.38E-11	3.36E-10
GO:0110020	regulation of actomyosin structure organization	43/3805	1.39E-11	3.38E-10
GO:0061387	regulation of extent of cell growth	47/3805	1.49E-11	3.60E-10
GO:0001938	positive regulation of endothelial cell proliferation	40/3805	1.50E-11	3.62E-10
GO:0032102	negative regulation of response to external stimulus	103/3805	1.60E-11	3.84E-10
GO:0030832	regulation of actin filament length	56/3805	1.61E-11	3.84E-10
GO:0014812	muscle cell migration	45/3805	1.74E-11	4.15E-10
GO:0042073	intraciliary transport	26/3805	1.80E-11	4.28E-10
GO:0006937	regulation of muscle contraction	55/3805	1.86E-11	4.39E-10
GO:0060840	artery development	44/3805	1.86E-11	4.39E-10
GO:0048813	dendrite morphogenesis	60/3805	1.87E-11	4.40E-10
GO:0003018	vascular process in circulatory system	70/3805	2.02E-11	4.71E-10
GO:0048675	axon extension	50/3805	2.02E-11	4.71E-10

GO:0090263	positive regulation of canonical Wnt signaling pathway	42/3805	2.06E-11	4.79E-10
GO:0003231	cardiac ventricle development	52/3805	2.12E-11	4.89E-10
GO:0071674	mononuclear cell migration	59/3805	2.20E-11	5.08E-10
GO:0048659	smooth muscle cell proliferation	60/3805	2.40E-11	5.51E-10
GO:0032872	regulation of stress-activated MAPK cascade	64/3805	2.48E-11	5.66E-10
GO:0001841	neural tube formation	48/3805	2.52E-11	5.74E-10
GO:0043588	skin development	87/3805	2.71E-11	6.15E-10
GO:0030516	regulation of axon extension	43/3805	2.78E-11	6.28E-10
GO:0003279	cardiac septum development	47/3805	2.79E-11	6.28E-10
GO:0060491	regulation of cell projection assembly	64/3805	3.12E-11	7.01E-10
GO:0097191	extrinsic apoptotic signaling pathway	71/3805	3.16E-11	7.06E-10
GO:0002028	regulation of sodium ion transport	39/3805	3.21E-11	7.17E-10
GO:0022612	gland morphogenesis	53/3805	3.23E-11	7.18E-10
GO:0051091	positive regulation of DNA-binding transcription factor activity	77/3805	3.26E-11	7.22E-10
GO:0055001	muscle cell development	68/3805	3.65E-11	8.05E-10
GO:0050769	positive regulation of neurogenesis	82/3805	3.69E-11	8.11E-10
GO:0043523	regulation of neuron apoptotic process	79/3805	3.88E-11	8.50E-10
GO:0060348	bone development	74/3805	3.99E-11	8.71E-10

GO:0042391	regulation of membrane potential	114/3805	4.12E-11	8.97E-10
GO:0010927	cellular component assembly involved in morphogenesis	45/3805	4.60E-11	9.98E-10
GO:0070252	actin-mediated cell contraction	39/3805	4.66E-11	1.01E-09
GO:0032412	regulation of ion transmembrane transporter activity	77/3805	4.80E-11	1.03E-09
GO:0070302	regulation of stress-activated protein kinase signaling cascade	64/3805	4.92E-11	1.06E-09
GO:0062197	cellular response to chemical stress	89/3805	5.06E-11	1.08E-09
GO:0022411	cellular component disassembly	106/3805	5.30E-11	1.13E-09
GO:0051656	establishment of organelle localization	106/3805	5.30E-11	1.13E-09
GO:0002062	chondrocyte differentiation	43/3805	5.42E-11	1.15E-09
GO:0048738	cardiac muscle tissue development	76/3805	5.87E-11	1.24E-09
GO:0031334	positive regulation of protein-containing complex assembly	63/3805	5.90E-11	1.24E-09
GO:0003015	heart process	72/3805	5.91E-11	1.24E-09
GO:0048661	positive regulation of smooth muscle cell proliferation	41/3805	6.17E-11	1.29E-09
GO:0030833	regulation of actin filament polymerization	50/3805	6.41E-11	1.33E-09
GO:0018209	peptidyl-serine modification	94/3805	6.66E-11	1.38E-09
GO:0048562	embryonic organ morphogenesis	88/3805	7.40E-11	1.53E-09
GO:1903034	regulation of response to wounding	56/3805	7.59E-11	1.56E-09
GO:0090101	negative regulation of transmembrane receptor protein serine/threonine kinase signaling pathway	46/3805	7.75E-11	1.59E-09

GO:2000181	negative regulation of blood vessel morphogenesis	42/3805	8.12E-11	1.66E-09
GO:0051924	regulation of calcium ion transport	79/3805	8.19E-11	1.67E-09
GO:0018105	peptidyl-serine phosphorylation	89/3805	8.40E-11	1.71E-09
GO:0007044	cell-substrate junction assembly	38/3805	9.92E-11	2.01E-09
GO:0051348	negative regulation of transferase activity	74/3805	1.06E-10	2.15E-09
GO:0006814	sodium ion transport	71/3805	1.08E-10	2.17E-09
GO:0002687	positive regulation of leukocyte migration	52/3805	1.09E-10	2.20E-09
GO:1901343	negative regulation of vasculature development	42/3805	1.13E-10	2.26E-09
GO:0010595	positive regulation of endothelial cell migration	41/3805	1.21E-10	2.42E-09
GO:0032984	protein-containing complex disassembly	69/3805	1.31E-10	2.60E-09
GO:1905475	regulation of protein localization to membrane	61/3805	1.33E-10	2.64E-09
GO:0001570	vasculogenesis	38/3805	1.42E-10	2.81E-09
GO:0032386	regulation of intracellular transport	88/3805	1.44E-10	2.85E-09
GO:0031532	actin cytoskeleton reorganization	42/3805	1.56E-10	3.06E-09
GO:0016525	negative regulation of angiogenesis	41/3805	1.69E-10	3.31E-09
GO:0007612	learning	58/3805	1.81E-10	3.52E-09
GO:0071772	response to BMP	58/3805	1.81E-10	3.52E-09
GO:0071773	cellular response to BMP stimulus	58/3805	1.81E-10	3.52E-09

GO:0007528	neuromuscular junction development	28/3805	1.85E-10	3.58E-09
GO:1901215	negative regulation of neuron death	73/3805	1.91E-10	3.69E-09
GO:0150115	cell-substrate junction organization	39/3805	1.93E-10	3.71E-09
GO:0021700	developmental maturation	91/3805	2.03E-10	3.88E-09
GO:0120193	tight junction organization	37/3805	2.11E-10	4.03E-09
GO:0001935	endothelial cell proliferation	57/3805	2.16E-10	4.11E-09
GO:0022409	positive regulation of cell-cell adhesion	80/3805	2.34E-10	4.45E-09
GO:2000027	regulation of animal organ morphogenesis	46/3805	2.51E-10	4.76E-09
GO:0007219	Notch signaling pathway	57/3805	2.73E-10	5.15E-09
GO:0030239	myofibril assembly	31/3805	2.80E-10	5.26E-09
GO:0030512	negative regulation of transforming growth factor beta receptor signaling pathway	32/3805	2.99E-10	5.61E-09
GO:0035023	regulation of Rho protein signal transduction	36/3805	3.12E-10	5.83E-09
GO:0003002	regionalization	96/3805	3.28E-10	6.11E-09
GO:1901990	regulation of mitotic cell cycle phase transition	82/3805	3.64E-10	6.77E-09
GO:0030509	BMP signaling pathway	55/3805	3.87E-10	7.18E-09
GO:0032233	positive regulation of actin filament bundle assembly	30/3805	3.96E-10	7.30E-09
GO:0044458	motile cilium assembly	30/3805	3.96E-10	7.30E-09
GO:0120032	regulation of plasma membrane bounded cell projection assembly	61/3805	4.02E-10	7.38E-09

GO:0035567	non-canonical Wnt signaling pathway	27/3805	4.11E-10	7.54E-09
GO:0044344	cellular response to fibroblast growth factor stimulus	37/3805	4.28E-10	7.79E-09
GO:0001936	regulation of endothelial cell proliferation	53/3805	4.28E-10	7.79E-09
GO:0055002	striated muscle cell development	31/3805	4.29E-10	7.79E-09
GO:0045930	negative regulation of mitotic cell cycle	66/3805	4.32E-10	7.82E-09
GO:1902105	regulation of leukocyte differentiation	84/3805	4.65E-10	8.39E-09
GO:0032272	negative regulation of protein polymerization	34/3805	4.66E-10	8.39E-09
GO:1903522	regulation of blood circulation	73/3805	4.87E-10	8.73E-09
GO:0045786	negative regulation of cell cycle	93/3805	5.32E-10	9.53E-09
GO:0032835	glomerulus development	29/3805	5.58E-10	9.95E-09
GO:0061640	cytoskeleton-dependent cytokinesis	36/3805	6.37E-10	1.13E-08
GO:0030100	regulation of endocytosis	67/3805	6.40E-10	1.14E-08
GO:0003014	renal system process	43/3805	6.49E-10	1.15E-08
GO:0060048	cardiac muscle contraction	45/3805	6.69E-10	1.18E-08
GO:0050919	negative chemotaxis	23/3805	6.79E-10	1.19E-08
GO:0021537	telencephalon development	71/3805	7.33E-10	1.29E-08
GO:0048771	tissue remodeling	60/3805	7.46E-10	1.30E-08
GO:0060047	heart contraction	67/3805	7.77E-10	1.36E-08

GO:0071774	response to fibroblast growth factor	37/3805	8.48E-10	1.47E-08
GO:0043409	negative regulation of MAPK cascade	57/3805	8.50E-10	1.47E-08
GO:0031099	regeneration	50/3805	9.02E-10	1.56E-08
GO:0031503	protein-containing complex localization	60/3805	9.22E-10	1.59E-08
GO:0045927	positive regulation of growth	83/3805	9.34E-10	1.61E-08
GO:0046785	microtubule polymerization	34/3805	9.80E-10	1.68E-08
GO:0043524	negative regulation of neuron apoptotic process	57/3805	1.06E-09	1.81E-08
GO:0014066	regulation of phosphatidylinositol 3-kinase signaling	38/3805	1.09E-09	1.86E-08
GO:0090162	establishment of epithelial cell polarity	20/3805	1.09E-09	1.86E-08
GO:0008406	gonad development	67/3805	1.14E-09	1.93E-08
GO:0051492	regulation of stress fiber assembly	37/3805	1.18E-09	2.00E-08
GO:0006874	cellular calcium ion homeostasis	115/3805	1.19E-09	2.01E-08
GO:0007059	chromosome segregation	86/3805	1.27E-09	2.14E-08
GO:0032874	positive regulation of stress-activated MAPK cascade	46/3805	1.31E-09	2.20E-08
GO:0060996	dendritic spine development	44/3805	1.33E-09	2.23E-08
GO:0043297	apical junction assembly	35/3805	1.34E-09	2.24E-08
GO:0045664	regulation of neuron differentiation	67/3805	1.38E-09	2.29E-08
GO:1903046	meiotic cell cycle process	63/3805	1.42E-09	2.36E-08

GO:0090288	negative regulation of cellular response to growth factor stimulus	38/3805	1.50E-09	2.49E-08
GO:0071526	semaphorin-plexin signaling pathway	24/3805	1.63E-09	2.69E-08
GO:0050731	positive regulation of peptidyl-tyrosine phosphorylation	57/3805	1.63E-09	2.69E-08
GO:0014911	positive regulation of smooth muscle cell migration	28/3805	1.91E-09	3.13E-08
GO:0045766	positive regulation of angiogenesis	55/3805	1.91E-09	3.13E-08
GO:1904018	positive regulation of vasculature development	55/3805	1.91E-09	3.13E-08
GO:0034599	cellular response to oxidative stress	73/3805	2.02E-09	3.29E-08
GO:0022408	negative regulation of cell-cell adhesion	60/3805	2.11E-09	3.42E-08
GO:0006979	response to oxidative stress	98/3805	2.11E-09	3.42E-08
GO:0061035	regulation of cartilage development	30/3805	2.12E-09	3.43E-08
GO:0051145	smooth muscle cell differentiation	32/3805	2.14E-09	3.45E-08
GO:0070304	positive regulation of stress-activated protein kinase signaling cascade	46/3805	2.20E-09	3.55E-08
GO:0032964	collagen biosynthetic process	26/3805	2.40E-09	3.85E-08
GO:0045137	development of primary sexual characteristics	67/3805	2.40E-09	3.85E-08
GO:0008544	epidermis development	89/3805	2.60E-09	4.15E-08
GO:0050921	positive regulation of chemotaxis	48/3805	2.63E-09	4.19E-08
GO:1904035	regulation of epithelial cell apoptotic process	35/3805	2.65E-09	4.21E-08
GO:2001236	regulation of extrinsic apoptotic signaling pathway	52/3805	2.66E-09	4.21E-08

GO:0085029	extracellular matrix assembly	22/3805	2.72E-09	4.30E-08
GO:0034332	adherens junction organization	24/3805	2.74E-09	4.33E-08
GO:0010934	macrophage cytokine production	14/3805	2.90E-09	4.57E-08
GO:0007613	memory	50/3805	3.01E-09	4.73E-08
GO:0032963	collagen metabolic process	42/3805	3.05E-09	4.78E-08
GO:0048844	artery morphogenesis	32/3805	3.09E-09	4.82E-08
GO:0061512	protein localization to cilium	30/3805	3.15E-09	4.90E-08
GO:0007019	microtubule depolymerization	25/3805	3.32E-09	5.15E-08
GO:0098813	nuclear chromosome segregation	73/3805	3.36E-09	5.21E-08
GO:0001666	response to hypoxia	61/3805	3.82E-09	5.91E-08
GO:0051058	negative regulation of small GTPase mediated signal transduction	27/3805	4.19E-09	6.46E-08
GO:0002573	myeloid leukocyte differentiation	66/3805	4.25E-09	6.54E-08
GO:0009416	response to light stimulus	81/3805	4.28E-09	6.56E-08
GO:0002274	myeloid leukocyte activation	71/3805	4.31E-09	6.60E-08
GO:0048286	lung alveolus development	28/3805	4.45E-09	6.79E-08
GO:0033673	negative regulation of kinase activity	63/3805	4.52E-09	6.89E-08
GO:0045637	regulation of myeloid cell differentiation	62/3805	4.59E-09	6.97E-08
GO:0014033	neural crest cell differentiation	36/3805	4.66E-09	7.07E-08

GO:0021591	ventricular system development	19/3805	4.77E-09	7.22E-08
GO:0014031	mesenchymal cell development	35/3805	5.11E-09	7.70E-08
GO:0010935	regulation of macrophage cytokine production	13/3805	5.16E-09	7.76E-08
GO:0086005	ventricular cardiac muscle cell action potential	17/3805	5.48E-09	8.23E-08
GO:0007159	leukocyte cell-cell adhesion	92/3805	5.67E-09	8.50E-08
GO:0006469	negative regulation of protein kinase activity	58/3805	5.72E-09	8.53E-08
GO:0030510	regulation of BMP signaling pathway	37/3805	5.72E-09	8.53E-08
GO:0060411	cardiac septum morphogenesis	33/3805	5.94E-09	8.83E-08
GO:0030517	negative regulation of axon extension	23/3805	6.12E-09	9.08E-08
GO:0035725	sodium ion transmembrane transport	47/3805	6.44E-09	9.54E-08
GO:0050878	regulation of body fluid levels	90/3805	6.56E-09	9.69E-08
GO:0007422	peripheral nervous system development	30/3805	6.74E-09	9.90E-08
GO:0014068	positive regulation of phosphatidylinositol 3-kinase signaling	30/3805	6.74E-09	9.90E-08
GO:0090596	sensory organ morphogenesis	80/3805	7.17E-09	1.05E-07
GO:0048863	stem cell differentiation	65/3805	7.49E-09	1.10E-07
GO:0035050	embryonic heart tube development	34/3805	7.67E-09	1.12E-07
GO:0006942	regulation of striated muscle contraction	33/3805	8.30E-09	1.21E-07
GO:0035721	intraciliary retrograde transport	Dec-05	8.55E-09	1.24E-07

GO:2001234	negative regulation of apoptotic signaling pathway	66/3805	8.67E-09	1.26E-07
GO:0080164	regulation of nitric oxide metabolic process	30/3805	9.73E-09	1.41E-07
GO:0003158	endothelium development	43/3805	9.84E-09	1.42E-07
GO:0030837	negative regulation of actin filament polymerization	28/3805	9.93E-09	1.43E-07
GO:0045428	regulation of nitric oxide biosynthetic process	29/3805	9.93E-09	1.43E-07
GO:1903131	mononuclear cell differentiation	111/3805	1.02E-08	1.46E-07
GO:0098739	import across plasma membrane	55/3805	1.03E-08	1.48E-07
GO:0090175	regulation of establishment of planar polarity	21/3805	1.06E-08	1.52E-07
GO:0031023	microtubule organizing center organization	45/3805	1.16E-08	1.64E-07
GO:0010971	positive regulation of G2/M transition of mitotic cell cycle	17/3805	1.16E-08	1.65E-07
GO:0008306	associative learning	40/3805	1.21E-08	1.70E-07
GO:2000177	regulation of neural precursor cell proliferation	40/3805	1.21E-08	1.70E-07
GO:0007229	integrin-mediated signaling pathway	35/3805	1.30E-08	1.84E-07
GO:0010001	glial cell differentiation	68/3805	1.34E-08	1.89E-07
GO:0043534	blood vessel endothelial cell migration	39/3805	1.39E-08	1.95E-07
GO:0060976	coronary vasculature development	30/3805	1.39E-08	1.95E-07
GO:0050727	regulation of inflammatory response	85/3805	1.43E-08	1.99E-07
GO:0032103	positive regulation of response to external stimulus	104/3805	1.48E-08	2.06E-07

GO:1903555	regulation of tumor necrosis factor superfamily cytokine production	54/3805	1.54E-08	2.15E-07
GO:1902414	protein localization to cell junction	40/3805	1.57E-08	2.18E-07
GO:1904019	epithelial cell apoptotic process	40/3805	1.57E-08	2.18E-07
GO:0010811	positive regulation of cell-substrate adhesion	44/3805	1.74E-08	2.41E-07
GO:0006939	smooth muscle contraction	39/3805	1.82E-08	2.52E-07
GO:0035107	appendage morphogenesis	51/3805	1.84E-08	2.52E-07
GO:0035108	limb morphogenesis	51/3805	1.84E-08	2.52E-07
GO:0007369	gastrulation	52/3805	1.86E-08	2.55E-07
GO:0048705	skeletal system morphogenesis	68/3805	1.87E-08	2.56E-07
GO:0001736	establishment of planar polarity	26/3805	2.11E-08	2.87E-07
GO:0060688	regulation of morphogenesis of a branching structure	27/3805	2.14E-08	2.92E-07
GO:0001656	metanephros development	33/3805	2.18E-08	2.96E-07
GO:0007254	JNK cascade	52/3805	2.29E-08	3.10E-07
GO:0042542	response to hydrogen peroxide	40/3805	2.64E-08	3.57E-07
GO:2001237	negative regulation of extrinsic apoptotic signaling pathway	36/3805	2.78E-08	3.75E-07
GO:2001222	regulation of neuron migration	24/3805	2.88E-08	3.87E-07
GO:0001818	negative regulation of cytokine production	74/3805	2.97E-08	3.98E-07
GO:1903828	negative regulation of protein localization	60/3805	3.01E-08	4.04E-07

GO:0031113	regulation of microtubule polymerization	25/3805	3.05E-08	4.06E-07
GO:0048846	axon extension involved in axon guidance	19/3805	3.05E-08	4.06E-07
GO:0060071	Wnt signaling pathway, planar cell polarity pathway	19/3805	3.05E-08	4.06E-07
GO:1902284	neuron projection extension involved in neuron projection guidance	19/3805	3.05E-08	4.06E-07
GO:0048146	positive regulation of fibroblast proliferation	28/3805	3.07E-08	4.07E-07
GO:0046328	regulation of JNK cascade	46/3805	3.08E-08	4.07E-07
GO:0043270	positive regulation of ion transport	80/3805	3.09E-08	4.09E-07
GO:0061041	regulation of wound healing	42/3805	3.13E-08	4.12E-07
GO:0007164	establishment of tissue polarity	26/3805	3.14E-08	4.12E-07
GO:0030865	cortical cytoskeleton organization	26/3805	3.14E-08	4.12E-07
GO:2001235	positive regulation of apoptotic signaling pathway	47/3805	3.18E-08	4.17E-07
GO:0140013	meiotic nuclear division	56/3805	3.31E-08	4.32E-07
GO:0008016	regulation of heart contraction	55/3805	3.36E-08	4.38E-07
GO:0071214	cellular response to abiotic stimulus	80/3805	3.56E-08	4.63E-07
GO:0104004	cellular response to environmental stimulus	80/3805	3.56E-08	4.63E-07
GO:0032411	positive regulation of transporter activity	42/3805	3.98E-08	5.15E-07
GO:0034767	positive regulation of ion transmembrane transport	54/3805	4.11E-08	5.32E-07
GO:0046847	filopodium assembly	29/3805	4.17E-08	5.37E-07

GO:1905515	non-motile cilium assembly	29/3805	4.17E-08	5.37E-07
GO:0007405	neuroblast proliferation	28/3805	4.40E-08	5.65E-07
GO:0030834	regulation of actin filament depolymerization	24/3805	4.40E-08	5.65E-07
GO:0014032	neural crest cell development	32/3805	4.48E-08	5.74E-07
GO:0098657	import into cell	63/3805	4.53E-08	5.79E-07
GO:0061082	myeloid leukocyte cytokine production	17/3805	4.56E-08	5.81E-07
GO:0030042	actin filament depolymerization	25/3805	4.58E-08	5.81E-07
GO:0035924	cellular response to vascular endothelial growth factor stimulus	25/3805	4.58E-08	5.81E-07
GO:0071706	tumor necrosis factor superfamily cytokine production	54/3805	4.97E-08	6.30E-07
GO:0030866	cortical actin cytoskeleton organization	19/3805	5.35E-08	6.75E-07
GO:0031114	regulation of microtubule depolymerization	19/3805	5.35E-08	6.75E-07
GO:0070588	calcium ion transmembrane transport	74/3805	5.37E-08	6.76E-07
GO:0032273	positive regulation of protein polymerization	33/3805	5.42E-08	6.81E-07
GO:0048144	fibroblast proliferation	37/3805	5.46E-08	6.85E-07
GO:0048259	regulation of receptor-mediated endocytosis	40/3805	5.60E-08	7.01E-07
GO:0031111	negative regulation of microtubule polymerization or depolymerization	22/3805	5.77E-08	7.20E-07
GO:0045446	endothelial cell differentiation	38/3805	6.06E-08	7.53E-07
GO:0072009	nephron epithelium development	38/3805	6.06E-08	7.53E-07

GO:0002088	lens development in camera-type eye	32/3805	6.08E-08	7.55E-07
GO:0032680	regulation of tumor necrosis factor production	52/3805	6.16E-08	7.64E-07
GO:0048678	response to axon injury	28/3805	6.24E-08	7.70E-07
GO:1905330	regulation of morphogenesis of an epithelium	28/3805	6.24E-08	7.70E-07
GO:0071677	positive regulation of mononuclear cell migration	27/3805	6.54E-08	8.06E-07
GO:0034764	positive regulation of transmembrane transport	65/3805	6.73E-08	8.28E-07
GO:0048041	focal adhesion assembly	31/3805	6.77E-08	8.30E-07
GO:0045862	positive regulation of proteolysis	84/3805	6.82E-08	8.35E-07
GO:0051147	regulation of muscle cell differentiation	45/3805	7.17E-08	8.77E-07
GO:0048864	stem cell development	33/3805	7.25E-08	8.85E-07
GO:0036293	response to decreased oxygen levels	63/3805	7.44E-08	9.05E-07
GO:0048145	regulation of fibroblast proliferation	36/3805	8.27E-08	1.01E-06
GO:0060326	cell chemotaxis	76/3805	8.29E-08	1.01E-06
GO:1902751	positive regulation of cell cycle G2/M phase transition	17/3805	8.52E-08	1.03E-06
GO:0045214	sarcomere organization	22/3805	9.01E-08	1.09E-06
GO:0018210	peptidyl-threonine modification	40/3805	9.06E-08	1.09E-06
GO:0045807	positive regulation of endocytosis	40/3805	9.06E-08	1.09E-06
GO:0050863	regulation of T cell activation	82/3805	9.10E-08	1.09E-06

GO:0000910	cytokinesis	51/3805	9.14E-08	1.10E-06
GO:0035088	establishment or maintenance of apical/basal cell polarity	23/3805	9.63E-08	1.15E-06
GO:0061245	establishment or maintenance of bipolar cell polarity	23/3805	9.63E-08	1.15E-06
GO:0006898	receptor-mediated endocytosis	67/3805	9.64E-08	1.15E-06
GO:0098900	regulation of action potential	26/3805	9.72E-08	1.16E-06
GO:0043491	protein kinase B signaling	56/3805	9.85E-08	1.17E-06
GO:0051496	positive regulation of stress fiber assembly	24/3805	9.93E-08	1.18E-06
GO:0051222	positive regulation of protein transport	77/3805	1.02E-07	1.20E-06
GO:0001823	mesonephros development	36/3805	1.07E-07	1.27E-06
GO:1903035	negative regulation of response to wounding	32/3805	1.10E-07	1.29E-06
GO:0045880	positive regulation of smoothened signaling pathway	20/3805	1.12E-07	1.31E-06
GO:0086002	cardiac muscle cell action potential involved in contraction	20/3805	1.12E-07	1.31E-06
GO:0043583	ear development	64/3805	1.14E-07	1.34E-06
GO:0007098	centrosome cycle	40/3805	1.15E-07	1.34E-06
GO:0010948	negative regulation of cell cycle process	71/3805	1.17E-07	1.37E-06
GO:0030307	positive regulation of cell growth	56/3805	1.17E-07	1.37E-06
GO:0045931	positive regulation of mitotic cell cycle	41/3805	1.21E-07	1.40E-06
GO:0048736	appendage development	55/3805	1.21E-07	1.41E-06

GO:0060173	limb development	55/3805	1.21E-07	1.41E-06
GO:0003281	ventricular septum development	31/3805	1.24E-07	1.43E-06
GO:0120192	tight junction assembly	31/3805	1.24E-07	1.43E-06
GO:0010038	response to metal ion	72/3805	1.26E-07	1.46E-06
GO:0018107	peptidyl-threonine phosphorylation	38/3805	1.28E-07	1.47E-06
GO:0055117	regulation of cardiac muscle contraction	27/3805	1.31E-07	1.51E-06
GO:0002040	sprouting angiogenesis	39/3805	1.37E-07	1.57E-06
GO:1903169	regulation of calcium ion transmembrane transport	49/3805	1.37E-07	1.57E-06
GO:0006809	nitric oxide biosynthetic process	30/3805	1.39E-07	1.58E-06
GO:0000281	mitotic cytokinesis	26/3805	1.39E-07	1.58E-06
GO:1902305	regulation of sodium ion transmembrane transport	26/3805	1.39E-07	1.58E-06
GO:1901988	negative regulation of cell cycle phase transition	63/3805	1.41E-07	1.60E-06
GO:0010712	regulation of collagen metabolic process	23/3805	1.45E-07	1.64E-06
GO:0046580	negative regulation of Ras protein signal transduction	23/3805	1.45E-07	1.64E-06
GO:0070301	cellular response to hydrogen peroxide	32/3805	1.46E-07	1.65E-06
GO:1903532	positive regulation of secretion by cell	84/3805	1.47E-07	1.66E-06
GO:0034763	negative regulation of transmembrane transport	42/3805	1.57E-07	1.77E-06
GO:0001657	ureteric bud development	35/3805	1.63E-07	1.82E-06

GO:0072163	mesonephric epithelium development	35/3805	1.63E-07	1.82E-06
GO:0072164	mesonephric tubule development	35/3805	1.63E-07	1.82E-06
GO:0090100	positive regulation of transmembrane receptor protein serine/threonine kinase signaling pathway	38/3805	1.63E-07	1.82E-06
GO:0019221	cytokine-mediated signaling pathway	94/3805	1.64E-07	1.83E-06
GO:0043433	negative regulation of DNA-binding transcription factor activity	48/3805	1.68E-07	1.87E-06
GO:0032414	positive regulation of ion transmembrane transporter activity	39/3805	1.73E-07	1.92E-06
GO:1905477	positive regulation of protein localization to membrane	36/3805	1.79E-07	1.98E-06
GO:0001649	osteoblast differentiation	58/3805	1.81E-07	2.00E-06
GO:0000302	response to reactive oxygen species	52/3805	1.89E-07	2.09E-06
GO:0032640	tumor necrosis factor production	52/3805	1.89E-07	2.09E-06
GO:0019932	second-messenger-mediated signaling	74/3805	1.91E-07	2.11E-06
GO:0038127	ERBB signaling pathway	37/3805	1.94E-07	2.12E-06
GO:0044843	cell cycle G1/S phase transition	60/3805	1.94E-07	2.12E-06
GO:0060041	retina development in camera-type eye	50/3805	1.98E-07	2.17E-06
GO:0010718	positive regulation of epithelial to mesenchymal transition	21/3805	1.99E-07	2.17E-06
GO:0001539	cilium or flagellum-dependent cell motility	48/3805	2.03E-07	2.22E-06
GO:0060285	cilium-dependent cell motility	48/3805	2.03E-07	2.22E-06
GO:1903037	regulation of leukocyte cell-cell adhesion	80/3805	2.04E-07	2.22E-06

GO:0070286	axonemal dynein complex assembly	18/3805	2.05E-07	2.22E-06
GO:0070306	lens fiber cell differentiation	18/3805	2.05E-07	2.22E-06
GO:0000070	mitotic sister chromatid segregation	46/3805	2.06E-07	2.22E-06
GO:0021782	glial cell development	38/3805	2.07E-07	2.23E-06
GO:0003208	cardiac ventricle morphogenesis	29/3805	2.10E-07	2.26E-06
GO:0008543	fibroblast growth factor receptor signaling pathway	29/3805	2.10E-07	2.26E-06
GO:0046209	nitric oxide metabolic process	31/3805	2.22E-07	2.38E-06
GO:0048872	homeostasis of number of cells	85/3805	2.23E-07	2.39E-06
GO:1903707	negative regulation of hemopoiesis	36/3805	2.29E-07	2.46E-06
GO:0043271	negative regulation of ion transport	49/3805	2.43E-07	2.59E-06
GO:0071604	transforming growth factor beta production	19/3805	2.49E-07	2.66E-06
GO:0051092	positive regulation of NF-kappaB transcription factor activity	45/3805	2.51E-07	2.67E-06
GO:0071695	anatomical structure maturation	72/3805	2.54E-07	2.71E-06
GO:0010714	positive regulation of collagen metabolic process	17/3805	2.70E-07	2.87E-06
GO:0003143	embryonic heart tube morphogenesis	26/3805	2.76E-07	2.93E-06
GO:0006887	exocytosis	92/3805	2.76E-07	2.93E-06
GO:0048008	platelet-derived growth factor receptor signaling pathway	25/3805	2.94E-07	3.11E-06
GO:0050866	negative regulation of cell activation	58/3805	2.95E-07	3.12E-06

GO:0050680	negative regulation of epithelial cell proliferation	48/3805	2.98E-07	3.14E-06
GO:0034614	cellular response to reactive oxygen species	42/3805	3.00E-07	3.14E-06
GO:0051783	regulation of nuclear division	42/3805	3.00E-07	3.14E-06
GO:0007009	plasma membrane organization	45/3805	3.06E-07	3.21E-06
GO:0048639	positive regulation of developmental growth	57/3805	3.10E-07	3.25E-06
GO:0045666	positive regulation of neuron differentiation	37/3805	3.12E-07	3.26E-06
GO:2000351	regulation of endothelial cell apoptotic process	22/3805	3.15E-07	3.29E-06
GO:0031644	regulation of nervous system process	52/3805	3.22E-07	3.35E-06
GO:0006820	anion transport	106/3805	3.43E-07	3.57E-06
GO:0032967	positive regulation of collagen biosynthetic process	16/3805	3.47E-07	3.58E-06
GO:1901889	negative regulation of cell junction assembly	16/3805	3.47E-07	3.58E-06
GO:0007088	regulation of mitotic nuclear division	35/3805	3.47E-07	3.58E-06
GO:0034446	substrate adhesion-dependent cell spreading	35/3805	3.47E-07	3.58E-06
GO:0045444	fat cell differentiation	65/3805	3.61E-07	3.72E-06
GO:0070373	negative regulation of ERK1 and ERK2 cascade	29/3805	3.82E-07	3.93E-06
GO:0035904	aorta development	26/3805	3.84E-07	3.95E-06
GO:2001057	reactive nitrogen species metabolic process	31/3805	3.88E-07	3.98E-06
GO:0000819	sister chromatid segregation	51/3805	3.97E-07	4.07E-06

GO:0038084	vascular endothelial growth factor signaling pathway	19/3805	3.98E-07	4.07E-06
GO:1904951	positive regulation of establishment of protein localization	78/3805	4.00E-07	4.08E-06
GO:0030098	lymphocyte differentiation	97/3805	4.25E-07	4.33E-06
GO:0051057	positive regulation of small GTPase mediated signal transduction	28/3805	4.30E-07	4.38E-06
GO:0050708	regulation of protein secretion	72/3805	4.37E-07	4.44E-06
GO:1902106	negative regulation of leukocyte differentiation	35/3805	4.43E-07	4.49E-06
GO:0003151	outflow tract morphogenesis	30/3805	4.46E-07	4.51E-06
GO:0035265	organ growth	56/3805	4.51E-07	4.56E-06
GO:0003401	axis elongation	17/3805	4.61E-07	4.64E-06
GO:0007026	negative regulation of microtubule depolymerization	17/3805	4.61E-07	4.64E-06
GO:0032330	regulation of chondrocyte differentiation	22/3805	4.65E-07	4.68E-06
GO:0002683	negative regulation of immune system process	97/3805	4.73E-07	4.75E-06
GO:0010657	muscle cell apoptotic process	33/3805	4.83E-07	4.84E-06
GO:0002688	regulation of leukocyte chemotaxis	38/3805	5.17E-07	5.17E-06
GO:0050818	regulation of coagulation	26/3805	5.30E-07	5.30E-06
GO:0008584	male gonad development	41/3805	5.53E-07	5.50E-06
GO:1904375	regulation of protein localization to cell periphery	41/3805	5.53E-07	5.50E-06
GO:0007548	sex differentiation	74/3805	5.53E-07	5.50E-06

GO:0002931	response to ischemia	18/3805	5.56E-07	5.51E-06
GO:1904645	response to amyloid-beta	18/3805	5.56E-07	5.51E-06
GO:0051480	regulation of cytosolic calcium ion concentration	88/3805	5.56E-07	5.51E-06
GO:0044839	cell cycle G2/M phase transition	43/3805	5.57E-07	5.51E-06
GO:0030193	regulation of blood coagulation	25/3805	5.79E-07	5.71E-06
GO:0033627	cell adhesion mediated by integrin	30/3805	5.88E-07	5.79E-06
GO:0051150	regulation of smooth muscle cell differentiation	16/3805	6.08E-07	5.98E-06
GO:0048839	inner ear development	56/3805	6.21E-07	6.10E-06
GO:0045744	negative regulation of G protein-coupled receptor signaling pathway	24/3805	6.22E-07	6.10E-06
GO:0051099	positive regulation of binding	51/3805	6.67E-07	6.53E-06
GO:0030835	negative regulation of actin filament depolymerization	20/3805	6.68E-07	6.53E-06
GO:0061326	renal tubule development	34/3805	6.70E-07	6.54E-06
GO:0035637	multicellular organismal signaling	44/3805	6.73E-07	6.56E-06
GO:0046546	development of primary male sexual characteristics	41/3805	6.79E-07	6.60E-06
GO:0034113	heterotypic cell-cell adhesion	22/3805	6.79E-07	6.60E-06
GO:0001508	action potential	42/3805	6.81E-07	6.60E-06
GO:0010633	negative regulation of epithelial cell migration	26/3805	7.26E-07	7.02E-06
GO:0046579	positive regulation of Ras protein signal transduction	26/3805	7.26E-07	7.02E-06

GO:0001890	placenta development	48/3805	7.42E-07	7.16E-06
GO:0035296	regulation of tube diameter	47/3805	7.64E-07	7.35E-06
GO:0097746	blood vessel diameter maintenance	47/3805	7.64E-07	7.35E-06
GO:0007173	epidermal growth factor receptor signaling pathway	33/3805	7.95E-07	7.64E-06
GO:0061337	cardiac conduction	25/3805	8.01E-07	7.68E-06
GO:0006261	DNA-templated DNA replication	42/3805	8.30E-07	7.95E-06
GO:0051346	negative regulation of hydrolase activity	82/3805	8.46E-07	8.09E-06
GO:0071901	negative regulation of protein serine/threonine kinase activity	34/3805	8.51E-07	8.13E-06
GO:0002697	regulation of immune effector process	94/3805	8.60E-07	8.20E-06
GO:0051893	regulation of focal adhesion assembly	24/3805	8.71E-07	8.28E-06
GO:0090109	regulation of cell-substrate junction assembly	24/3805	8.71E-07	8.28E-06
GO:0061097	regulation of protein tyrosine kinase activity	27/3805	8.78E-07	8.33E-06
GO:0045055	regulated exocytosis	68/3805	8.87E-07	8.39E-06
GO:0045165	cell fate commitment	68/3805	8.87E-07	8.39E-06
GO:0060415	muscle tissue morphogenesis	29/3805	8.93E-07	8.44E-06
GO:0035150	regulation of tube size	47/3805	9.14E-07	8.62E-06
GO:0050922	negative regulation of chemotaxis	23/3805	9.32E-07	8.78E-06
GO:0010389	regulation of G2/M transition of mitotic cell cycle	32/3805	9.42E-07	8.86E-06

GO:0098840	protein transport along microtubule	Oct-05	9.60E-07	8.99E-06
GO:0099118	microtubule-based protein transport	Oct-05	9.60E-07	8.99E-06
GO:2000052	positive regulation of non-canonical Wnt signaling pathway	Oct-05	9.60E-07	8.99E-06
GO:0021955	central nervous system neuron axonogenesis	19/3805	9.64E-07	9.02E-06
GO:0030858	positive regulation of epithelial cell differentiation	22/3805	9.79E-07	9.12E-06
GO:0072577	endothelial cell apoptotic process	22/3805	9.79E-07	9.12E-06
GO:2000649	regulation of sodium ion transmembrane transporter activity	22/3805	9.79E-07	9.12E-06
GO:0000079	regulation of cyclin-dependent protein serine/threonine kinase activity	26/3805	9.87E-07	9.16E-06
GO:0043954	cellular component maintenance	26/3805	9.87E-07	9.16E-06
GO:0032965	regulation of collagen biosynthetic process	20/3805	1.00E-06	9.28E-06
GO:0045197	establishment or maintenance of epithelial cell apical/basal polarity	20/3805	1.00E-06	9.28E-06
GO:0030857	negative regulation of epithelial cell differentiation	21/3805	1.01E-06	9.29E-06
GO:0055123	digestive system development	40/3805	1.01E-06	9.34E-06
GO:0046330	positive regulation of JNK cascade	33/3805	1.01E-06	9.34E-06
GO:0035051	cardiocyte differentiation	49/3805	1.02E-06	9.34E-06
GO:0070830	bicellular tight junction assembly	28/3805	1.03E-06	9.44E-06
GO:0048841	regulation of axon extension involved in axon guidance	16/3805	1.04E-06	9.49E-06
GO:1904646	cellular response to amyloid-beta	16/3805	1.04E-06	9.49E-06

GO:1900046	regulation of hemostasis	25/3805	1.10E-06	1.00E-05
GO:0035264	multicellular organism growth	56/3805	1.16E-06	1.06E-05
GO:0032611	interleukin-1 beta production	32/3805	1.20E-06	1.10E-05
GO:0090136	epithelial cell-cell adhesion	Nov-05	1.20E-06	1.10E-05
GO:0014902	myotube differentiation	40/3805	1.24E-06	1.13E-05
GO:0035329	hippo signaling	17/3805	1.24E-06	1.13E-05
GO:0086091	regulation of heart rate by cardiac conduction	17/3805	1.24E-06	1.13E-05
GO:0097581	lamellipodium organization	30/3805	1.31E-06	1.18E-05
GO:0002011	morphogenesis of an epithelial sheet	23/3805	1.31E-06	1.18E-05
GO:1902749	regulation of cell cycle G2/M phase transition	34/3805	1.36E-06	1.22E-05
GO:0051693	actin filament capping	18/3805	1.38E-06	1.25E-05
GO:0098659	inorganic cation import across plasma membrane	35/3805	1.41E-06	1.27E-05
GO:0099587	inorganic ion import across plasma membrane	35/3805	1.41E-06	1.27E-05
GO:0032388	positive regulation of intracellular transport	49/3805	1.42E-06	1.28E-05
GO:0030217	T cell differentiation	70/3805	1.45E-06	1.30E-05
GO:0051963	regulation of synapse assembly	36/3805	1.46E-06	1.30E-05
GO:0060412	ventricular septum morphogenesis	21/3805	1.46E-06	1.30E-05
GO:0086003	cardiac muscle cell contraction	25/3805	1.50E-06	1.33E-05

GO:0071692	protein localization to extracellular region	88/3805	1.50E-06	1.34E-05
GO:0000086	G2/M transition of mitotic cell cycle	39/3805	1.51E-06	1.35E-05
GO:0042116	macrophage activation	32/3805	1.53E-06	1.36E-05
GO:0072080	nephron tubule development	32/3805	1.53E-06	1.36E-05
GO:0043535	regulation of blood vessel endothelial cell migration	30/3805	1.69E-06	1.49E-05
GO:1903557	positive regulation of tumor necrosis factor superfamily cytokine production	34/3805	1.70E-06	1.51E-05
GO:0048843	negative regulation of axon extension involved in axon guidance	14/3805	1.76E-06	1.56E-05
GO:0072089	stem cell proliferation	28/3805	1.78E-06	1.57E-05
GO:0051149	positive regulation of muscle cell differentiation	26/3805	1.78E-06	1.57E-05
GO:0060433	bronchus development	Sep-05	1.83E-06	1.61E-05
GO:0009913	epidermal cell differentiation	58/3805	1.84E-06	1.61E-05
GO:0043281	regulation of cysteine-type endopeptidase activity involved in apoptotic process	53/3805	1.87E-06	1.64E-05
GO:0060993	kidney morphogenesis	32/3805	1.95E-06	1.71E-05
GO:0031112	positive regulation of microtubule polymerization or depolymerization	17/3805	1.97E-06	1.72E-05
GO:0060674	placenta blood vessel development	17/3805	1.97E-06	1.72E-05
GO:0071634	regulation of transforming growth factor beta production	17/3805	1.97E-06	1.72E-05
GO:0010761	fibroblast migration	22/3805	1.97E-06	1.72E-05
GO:0060294	cilium movement involved in cell motility	44/3805	2.04E-06	1.78E-05

GO:0072676	lymphocyte migration	33/3805	2.05E-06	1.78E-05
GO:0033157	regulation of intracellular protein transport	56/3805	2.10E-06	1.82E-05
GO:0009914	hormone transport	83/3805	2.14E-06	1.85E-05
GO:0021954	central nervous system neuron development	30/3805	2.16E-06	1.87E-05
GO:0048644	muscle organ morphogenesis	30/3805	2.16E-06	1.87E-05
GO:1903115	regulation of actin filament-based movement	19/3805	2.18E-06	1.88E-05
GO:0034103	regulation of tissue remodeling	28/3805	2.32E-06	2.00E-05
GO:0002690	positive regulation of leukocyte chemotaxis	31/3805	2.32E-06	2.00E-05
GO:0042634	regulation of hair cycle	15/3805	2.33E-06	2.00E-05
GO:0071248	cellular response to metal ion	45/3805	2.35E-06	2.02E-05
GO:1904029	regulation of cyclin-dependent protein kinase activity	26/3805	2.37E-06	2.03E-05
GO:0030595	leukocyte chemotaxis	56/3805	2.43E-06	2.08E-05
GO:0045619	regulation of lymphocyte differentiation	51/3805	2.46E-06	2.10E-05
GO:0001755	neural crest cell migration	23/3805	2.51E-06	2.14E-05
GO:0010658	striated muscle cell apoptotic process	23/3805	2.51E-06	2.14E-05
GO:2000401	regulation of lymphocyte migration	23/3805	2.51E-06	2.14E-05
GO:0002065	columnar/cuboidal epithelial cell differentiation	33/3805	2.57E-06	2.18E-05
GO:0106027	neuron projection organization	33/3805	2.57E-06	2.18E-05

GO:0016311	dephosphorylation	77/3805	2.63E-06	2.23E-05
GO:0061430	bone trabecula morphogenesis	Nov-05	2.73E-06	2.31E-05
GO:0002224	toll-like receptor signaling pathway	36/3805	2.75E-06	2.32E-05
GO:1901890	positive regulation of cell junction assembly	36/3805	2.75E-06	2.32E-05
GO:0032651	regulation of interleukin-1 beta production	30/3805	2.77E-06	2.33E-05
GO:0014044	Schwann cell development	16/3805	2.77E-06	2.33E-05
GO:1904064	positive regulation of cation transmembrane transport	45/3805	2.79E-06	2.35E-05
GO:1901653	cellular response to peptide	71/3805	2.80E-06	2.35E-05
GO:0007249	I-kappaB kinase/NF-kappaB signaling	56/3805	2.81E-06	2.36E-05
GO:0048592	eye morphogenesis	48/3805	2.88E-06	2.41E-05
GO:0048593	camera-type eye morphogenesis	42/3805	3.10E-06	2.59E-05
GO:0061484	hematopoietic stem cell homeostasis	14/3805	3.10E-06	2.59E-05
GO:0045332	phospholipid translocation	20/3805	3.12E-06	2.60E-05
GO:0002090	regulation of receptor internalization	26/3805	3.13E-06	2.61E-05
GO:0031214	biomineral tissue development	46/3805	3.17E-06	2.64E-05
GO:0034331	cell junction maintenance	18/3805	3.20E-06	2.66E-05
GO:0032760	positive regulation of tumor necrosis factor production	33/3805	3.21E-06	2.66E-05
GO:0052547	regulation of peptidase activity	93/3805	3.22E-06	2.67E-05

GO:1902746	regulation of lens fiber cell differentiation	Aug-05	3.22E-06	2.67E-05
GO:0002367	cytokine production involved in immune response	34/3805	3.29E-06	2.72E-05
GO:1904705	regulation of vascular associated smooth muscle cell proliferation	23/3805	3.44E-06	2.84E-05
GO:1901652	response to peptide	89/3805	3.49E-06	2.88E-05
GO:0038034	signal transduction in absence of ligand	27/3805	3.53E-06	2.90E-05
GO:0097192	extrinsic apoptotic signaling pathway in absence of ligand	27/3805	3.53E-06	2.90E-05
GO:0006836	neurotransmitter transport	58/3805	3.71E-06	3.05E-05
GO:0097061	dendritic spine organization	31/3805	3.71E-06	3.05E-05
GO:0010659	cardiac muscle cell apoptotic process	22/3805	3.80E-06	3.11E-05
GO:0014009	glial cell proliferation	22/3805	3.80E-06	3.11E-05
GO:0034612	response to tumor necrosis factor	51/3805	3.89E-06	3.18E-05
GO:0008277	regulation of G protein-coupled receptor signaling pathway	38/3805	4.07E-06	3.31E-05
GO:0030178	negative regulation of Wnt signaling pathway	44/3805	4.09E-06	3.33E-05
GO:2000379	positive regulation of reactive oxygen species metabolic process	26/3805	4.11E-06	3.33E-05
GO:1901989	positive regulation of cell cycle phase transition	35/3805	4.11E-06	3.33E-05
GO:0150116	regulation of cell-substrate junction organization	24/3805	4.12E-06	3.33E-05
GO:1904377	positive regulation of protein localization to cell periphery	24/3805	4.12E-06	3.33E-05
GO:0048565	digestive tract development	36/3805	4.12E-06	3.34E-05

GO:0001505	regulation of neurotransmitter levels	60/3805	4.18E-06	3.37E-05
GO:0099003	vesicle-mediated transport in synapse	60/3805	4.18E-06	3.37E-05
GO:0010719	negative regulation of epithelial to mesenchymal transition	16/3805	4.38E-06	3.53E-05
GO:0009612	response to mechanical stimulus	46/3805	4.41E-06	3.55E-05
GO:0061005	cell differentiation involved in kidney development	20/3805	4.45E-06	3.57E-05
GO:0002718	regulation of cytokine production involved in immune response	30/3805	4.46E-06	3.58E-05
GO:0006260	DNA replication	64/3805	4.47E-06	3.58E-05
GO:0009306	protein secretion	85/3805	4.60E-06	3.68E-05
GO:0052548	regulation of endopeptidase activity	78/3805	4.69E-06	3.75E-05
GO:0051047	positive regulation of secretion	88/3805	4.69E-06	3.75E-05
GO:0030278	regulation of ossification	38/3805	4.92E-06	3.92E-05
GO:0061008	hepaticobiliary system development	37/3805	4.99E-06	3.97E-05
GO:0032612	interleukin-1 production	35/3805	5.04E-06	4.01E-05
GO:0035592	establishment of protein localization to extracellular region	85/3805	5.10E-06	4.05E-05
GO:0006949	syncytium formation	24/3805	5.48E-06	4.35E-05
GO:0032091	negative regulation of protein binding	30/3805	5.62E-06	4.45E-05
GO:0007269	neurotransmitter secretion	47/3805	5.75E-06	4.52E-05
GO:0071356	cellular response to tumor necrosis factor	47/3805	5.75E-06	4.52E-05

GO:0099643	signal release from synapse	47/3805	5.75E-06	4.52E-05
GO:0110148	biomineralization	47/3805	5.75E-06	4.52E-05
GO:0048538	thymus development	21/3805	5.76E-06	4.52E-05
GO:0061383	trabecula morphogenesis	21/3805	5.76E-06	4.52E-05
GO:0086001	cardiac muscle cell action potential	21/3805	5.76E-06	4.52E-05
GO:0030326	embryonic limb morphogenesis	39/3805	5.79E-06	4.54E-05
GO:0035113	embryonic appendage morphogenesis	39/3805	5.79E-06	4.54E-05
GO:0099565	chemical synaptic transmission, postsynaptic	31/3805	5.84E-06	4.57E-05
GO:0008088	axo-dendritic transport	27/3805	5.90E-06	4.60E-05
GO:0034109	homotypic cell-cell adhesion	27/3805	5.90E-06	4.60E-05
GO:0046879	hormone secretion	80/3805	5.98E-06	4.66E-05
GO:2000116	regulation of cysteine-type endopeptidase activity	57/3805	6.02E-06	4.69E-05
GO:0097193	intrinsic apoptotic signaling pathway	69/3805	6.02E-06	4.69E-05
GO:0021953	central nervous system neuron differentiation	51/3805	6.08E-06	4.73E-05
GO:0008585	female gonad development	34/3805	6.16E-06	4.78E-05
GO:0002063	chondrocyte development	15/3805	6.20E-06	4.80E-05
GO:0006270	DNA replication initiation	15/3805	6.20E-06	4.80E-05
GO:0033260	nuclear DNA replication	15/3805	6.20E-06	4.80E-05

GO:0007492	endoderm development	25/3805	6.26E-06	4.82E-05
GO:0034204	lipid translocation	20/3805	6.26E-06	4.82E-05
GO:1904036	negative regulation of epithelial cell apoptotic process	20/3805	6.26E-06	4.82E-05
GO:0030203	glycosaminoglycan metabolic process	29/3805	6.74E-06	5.18E-05
GO:0051899	membrane depolarization	29/3805	6.74E-06	5.18E-05
GO:0035909	aorta morphogenesis	16/3805	6.77E-06	5.20E-05
GO:1902692	regulation of neuroblast proliferation	17/3805	7.00E-06	5.36E-05
GO:0097529	myeloid leukocyte migration	55/3805	7.00E-06	5.36E-05
GO:1901992	positive regulation of mitotic cell cycle phase transition	30/3805	7.05E-06	5.39E-05
GO:0001947	heart looping	22/3805	7.08E-06	5.40E-05
GO:2001257	regulation of cation channel activity	46/3805	7.11E-06	5.43E-05
GO:0070661	leukocyte proliferation	79/3805	7.29E-06	5.56E-05
GO:0001889	liver development	36/3805	7.42E-06	5.64E-05
GO:0042310	vasoconstriction	32/3805	7.43E-06	5.64E-05
GO:0051896	regulation of protein kinase B signaling	45/3805	7.52E-06	5.70E-05
GO:0030316	osteoclast differentiation	34/3805	7.53E-06	5.71E-05
GO:1901991	negative regulation of mitotic cell cycle phase transition	44/3805	7.92E-06	6.00E-05
GO:0050905	neuromuscular process	49/3805	8.04E-06	6.08E-05

GO:1903039	positive regulation of leukocyte cell-cell adhesion	58/3805	8.27E-06	6.25E-05
GO:0051321	meiotic cell cycle	73/3805	8.29E-06	6.25E-05
GO:0150076	neuroinflammatory response	23/3805	8.35E-06	6.28E-05
GO:1990874	vascular associated smooth muscle cell proliferation	23/3805	8.35E-06	6.28E-05
GO:0002700	regulation of production of molecular mediator of immune response	45/3805	8.80E-06	6.62E-05
GO:0016202	regulation of striated muscle tissue development	Dec-05	9.06E-06	6.80E-05
GO:0006022	aminoglycan metabolic process	32/3805	9.16E-06	6.87E-05
GO:0120034	positive regulation of plasma membrane bounded cell projection assembly	33/3805	9.21E-06	6.90E-05
GO:0007596	blood coagulation	44/3805	9.30E-06	6.95E-05
GO:0046661	male sex differentiation	44/3805	9.30E-06	6.95E-05
GO:0072678	T cell migration	22/3805	9.52E-06	7.11E-05
GO:0031116	positive regulation of microtubule polymerization	15/3805	9.75E-06	7.26E-05
GO:0090504	epiboly	15/3805	9.75E-06	7.26E-05
GO:0045580	regulation of T cell differentiation	43/3805	9.80E-06	7.29E-05
GO:0006858	extracellular transport	18/3805	1.00E-05	7.42E-05
GO:0031952	regulation of protein autophosphorylation	18/3805	1.00E-05	7.42E-05
GO:0001953	negative regulation of cell-matrix adhesion	16/3805	1.03E-05	7.60E-05
GO:0044786	cell cycle DNA replication	16/3805	1.03E-05	7.60E-05

GO:0030902	hindbrain development	45/3805	1.03E-05	7.60E-05
GO:0014037	Schwann cell differentiation	17/3805	1.03E-05	7.60E-05
GO:2000249	regulation of actin cytoskeleton reorganization	17/3805	1.03E-05	7.60E-05
GO:0061982	meiosis I cell cycle process	37/3805	1.05E-05	7.76E-05
GO:0043113	receptor clustering	25/3805	1.06E-05	7.78E-05
GO:0007204	positive regulation of cytosolic calcium ion concentration	76/3805	1.07E-05	7.88E-05
GO:0050772	positive regulation of axonogenesis	30/3805	1.10E-05	8.03E-05
GO:0090630	activation of GTPase activity	30/3805	1.10E-05	8.03E-05
GO:0000768	syncytium formation by plasma membrane fusion	23/3805	1.10E-05	8.07E-05
GO:0061371	determination of heart left/right asymmetry	23/3805	1.10E-05	8.07E-05
GO:0140253	cell-cell fusion	23/3805	1.10E-05	8.07E-05
GO:0010763	positive regulation of fibroblast migration	Nov-05	1.11E-05	8.10E-05
GO:0045019	negative regulation of nitric oxide biosynthetic process	Nov-05	1.11E-05	8.10E-05
GO:0072010	glomerular epithelium development	Nov-05	1.11E-05	8.10E-05
GO:1904406	negative regulation of nitric oxide metabolic process	Nov-05	1.11E-05	8.10E-05
GO:0046545	development of primary female sexual characteristics	34/3805	1.12E-05	8.12E-05
GO:0032652	regulation of interleukin-1 production	33/3805	1.13E-05	8.16E-05
GO:0007274	neuromuscular synaptic transmission	13/3805	1.19E-05	8.61E-05

GO:0031954	positive regulation of protein autophosphorylation	13/3805	1.19E-05	8.61E-05
GO:0008542	visual learning	24/3805	1.24E-05	8.96E-05
GO:0043506	regulation of JUN kinase activity	24/3805	1.24E-05	8.96E-05
GO:0061045	negative regulation of wound healing	24/3805	1.24E-05	8.96E-05
GO:0031623	receptor internalization	37/3805	1.26E-05	9.06E-05
GO:0042303	molting cycle	37/3805	1.26E-05	9.06E-05
GO:0042633	hair cycle	37/3805	1.26E-05	9.06E-05
GO:0050773	regulation of dendrite development	37/3805	1.26E-05	9.06E-05
GO:0008347	glial cell migration	22/3805	1.27E-05	9.11E-05
GO:0003184	pulmonary valve morphogenesis	Oct-05	1.27E-05	9.12E-05
GO:2001044	regulation of integrin-mediated signaling pathway	Oct-05	1.27E-05	9.12E-05
GO:0010457	centriole-centriole cohesion	Sep-05	1.29E-05	9.21E-05
GO:0048755	branching morphogenesis of a nerve	Sep-05	1.29E-05	9.21E-05
GO:1902969	mitotic DNA replication	Sep-05	1.29E-05	9.21E-05
GO:0051293	establishment of spindle localization	19/3805	1.32E-05	9.39E-05
GO:0010660	regulation of muscle cell apoptotic process	29/3805	1.33E-05	9.45E-05
GO:0007292	female gamete generation	43/3805	1.35E-05	9.57E-05
GO:0051100	negative regulation of binding	43/3805	1.35E-05	9.57E-05

GO:0010769	regulation of cell morphogenesis involved in differentiation	34/3805	1.35E-05	9.60E-05
GO:0043502	regulation of muscle adaptation	30/3805	1.36E-05	9.60E-05
GO:0060395	SMAD protein signal transduction	30/3805	1.36E-05	9.60E-05
GO:0006310	DNA recombination	70/3805	1.36E-05	9.61E-05
GO:0001892	embryonic placenta development	33/3805	1.37E-05	9.66E-05
GO:0051225	spindle assembly	33/3805	1.37E-05	9.66E-05
GO:0007156	homophilic cell adhesion via plasma membrane adhesion molecules	32/3805	1.38E-05	9.70E-05
GO:0001945	lymph vessel development	14/3805	1.39E-05	9.71E-05
GO:0021952	central nervous system projection neuron axonogenesis	14/3805	1.39E-05	9.71E-05
GO:0061036	positive regulation of cartilage development	14/3805	1.39E-05	9.71E-05
GO:0061037	negative regulation of cartilage development	14/3805	1.39E-05	9.71E-05
GO:0086010	membrane depolarization during action potential	14/3805	1.39E-05	9.71E-05
GO:2000352	negative regulation of endothelial cell apoptotic process	14/3805	1.39E-05	9.71E-05
GO:1902115	regulation of organelle assembly	51/3805	1.42E-05	9.96E-05
GO:0060443	mammary gland morphogenesis	21/3805	1.45E-05	0.000101053
GO:0098815	modulation of excitatory postsynaptic potential	21/3805	1.45E-05	0.000101053
GO:0007599	hemostasis	44/3805	1.49E-05	0.000103396
GO:0050817	coagulation	44/3805	1.49E-05	0.000103396

GO:0003351	epithelial cilium movement involved in extracellular fluid movement	17/3805	1.50E-05	0.000103841
GO:0010765	positive regulation of sodium ion transport	17/3805	1.50E-05	0.000103841
GO:0042176	regulation of protein catabolic process	82/3805	1.50E-05	0.000104314
GO:0002761	regulation of myeloid leukocyte differentiation	37/3805	1.51E-05	0.000104364
GO:0010842	retina layer formation	Dec-05	1.59E-05	0.000110103
GO:0099625	ventricular cardiac muscle cell membrane repolarization	Dec-05	1.59E-05	0.000110103
GO:0045732	positive regulation of protein catabolic process	53/3805	1.60E-05	0.000110449
GO:0140029	exocytic process	28/3805	1.61E-05	0.00011073
GO:0001885	endothelial cell development	24/3805	1.61E-05	0.00011073
GO:1903510	mucopolysaccharide metabolic process	24/3805	1.61E-05	0.00011073
GO:0035019	somatic stem cell population maintenance	20/3805	1.64E-05	0.000112258
GO:0051653	spindle localization	20/3805	1.64E-05	0.000112258
GO:0030317	flagellated sperm motility	39/3805	1.65E-05	0.000113273
GO:0051897	positive regulation of protein kinase B signaling	33/3805	1.66E-05	0.000113958
GO:0045581	negative regulation of T cell differentiation	19/3805	1.82E-05	0.000124435
GO:0061028	establishment of endothelial barrier	19/3805	1.82E-05	0.000124435
GO:1902806	regulation of cell cycle G1/S phase transition	43/3805	1.84E-05	0.000125646
GO:0042246	tissue regeneration	23/3805	1.89E-05	0.00012903

GO:0043500	muscle adaptation	35/3805	1.92E-05	0.000130774
GO:0045620	negative regulation of lymphocyte differentiation	21/3805	1.94E-05	0.00013179
GO:0061333	renal tubule morphogenesis	27/3805	1.94E-05	0.00013179
GO:1901019	regulation of calcium ion transmembrane transporter activity	27/3805	1.94E-05	0.00013179
GO:0032675	regulation of interleukin-6 production	42/3805	1.95E-05	0.000132426
GO:0046620	regulation of organ growth	34/3805	1.97E-05	0.000133556
GO:0001941	postsynaptic membrane organization	18/3805	1.99E-05	0.00013466
GO:0003179	heart valve morphogenesis	18/3805	1.99E-05	0.00013466
GO:0048010	vascular endothelial growth factor receptor signaling pathway	18/3805	1.99E-05	0.00013466
GO:0006970	response to osmotic stress	28/3805	2.00E-05	0.000135082
GO:0002221	pattern recognition receptor signaling pathway	44/3805	2.01E-05	0.000135511
GO:0050764	regulation of phagocytosis	33/3805	2.01E-05	0.000135511
GO:0010770	positive regulation of cell morphogenesis involved in differentiation	30/3805	2.06E-05	0.000138097
GO:0046849	bone remodeling	30/3805	2.06E-05	0.000138097
GO:0051153	regulation of striated muscle cell differentiation	30/3805	2.06E-05	0.000138097
GO:0051797	regulation of hair follicle development	Nov-05	2.06E-05	0.000138097
GO:0002263	cell activation involved in immune response	71/3805	2.12E-05	0.000141635
GO:0010464	regulation of mesenchymal cell proliferation	17/3805	2.14E-05	0.000142911

GO:0061098	positive regulation of protein tyrosine kinase activity	17/3805	2.14E-05	0.000142911
GO:0022011	myelination in peripheral nervous system	14/3805	2.17E-05	0.00014426
GO:0032292	peripheral nervous system axon ensheathment	14/3805	2.17E-05	0.00014426
GO:0034260	negative regulation of GTPase activity	14/3805	2.17E-05	0.00014426
GO:0048668	collateral sprouting	14/3805	2.17E-05	0.00014426
GO:1905332	positive regulation of morphogenesis of an epithelium	16/3805	2.23E-05	0.000148491
GO:1900180	regulation of protein localization to nucleus	39/3805	2.30E-05	0.000152601
GO:0072088	nephron epithelium morphogenesis	26/3805	2.34E-05	0.000155163
GO:0098773	skin epidermis development	33/3805	2.43E-05	0.000160851
GO:0033077	T cell differentiation in thymus	28/3805	2.48E-05	0.000164408
GO:0000082	G1/S transition of mitotic cell cycle	49/3805	2.50E-05	0.000164991
GO:0060349	bone morphogenesis	31/3805	2.51E-05	0.000165635
GO:0032092	positive regulation of protein binding	29/3805	2.52E-05	0.000165933
GO:0031102	neuron projection regeneration	21/3805	2.57E-05	0.00016912
GO:0060401	cytosolic calcium ion transport	48/3805	2.69E-05	0.000177247
GO:0061162	establishment of monopolar cell polarity	Dec-05	2.70E-05	0.000177247
GO:0000727	double-strand break repair via break-induced replication	Aug-05	2.77E-05	0.000180935
GO:0048251	elastic fiber assembly	Aug-05	2.77E-05	0.000180935

GO:0060907	positive regulation of macrophage cytokine production	Aug-05	2.77E-05	0.000180935
GO:0072697	protein localization to cell cortex	Aug-05	2.77E-05	0.000180935
GO:0098911	regulation of ventricular cardiac muscle cell action potential	Aug-05	2.77E-05	0.000180935
GO:0021543	pallium development	41/3805	2.82E-05	0.000184465
GO:0010469	regulation of signaling receptor activity	38/3805	2.85E-05	0.000185416
GO:0001977	renal system process involved in regulation of blood volume	Sep-05	2.85E-05	0.000185416
GO:0099550	trans-synaptic signaling, modulating synaptic transmission	Sep-05	2.85E-05	0.000185416
GO:2000095	regulation of Wnt signaling pathway, planar cell polarity pathway	Sep-05	2.85E-05	0.000185416
GO:0060350	endochondral bone morphogenesis	22/3805	2.88E-05	0.000187494
GO:0019722	calcium-mediated signaling	47/3805	2.90E-05	0.000188324
GO:0051302	regulation of cell division	47/3805	2.90E-05	0.000188324
GO:0019229	regulation of vasoconstriction	26/3805	2.94E-05	0.000190241
GO:0001942	hair follicle development	32/3805	2.99E-05	0.000193487
GO:0051054	positive regulation of DNA metabolic process	65/3805	2.99E-05	0.000193487
GO:0086009	membrane repolarization	17/3805	3.02E-05	0.000194656
GO:1902991	regulation of amyloid precursor protein catabolic process	17/3805	3.02E-05	0.000194656
GO:0021549	cerebellum development	31/3805	3.04E-05	0.000196258
GO:0002695	negative regulation of leukocyte activation	48/3805	3.09E-05	0.00019863

GO:0032147	activation of protein kinase activity	29/3805	3.09E-05	0.00019863
GO:0097205	renal filtration	13/3805	3.11E-05	0.000199912
GO:0043122	regulation of I-kappaB kinase/NF-kappaB signaling	46/3805	3.13E-05	0.00020054
GO:0009581	detection of external stimulus	36/3805	3.13E-05	0.00020054
GO:0048168	regulation of neuronal synaptic plasticity	23/3805	3.16E-05	0.000201511
GO:0055008	cardiac muscle tissue morphogenesis	23/3805	3.16E-05	0.000201511
GO:1990868	response to chemokine	23/3805	3.16E-05	0.000201511
GO:1990869	cellular response to chemokine	23/3805	3.16E-05	0.000201511
GO:0097722	sperm motility	39/3805	3.17E-05	0.000202274
GO:0035850	epithelial cell differentiation involved in kidney development	16/3805	3.22E-05	0.000204833
GO:0060603	mammary gland duct morphogenesis	16/3805	3.22E-05	0.000204833
GO:0007127	meiosis I	35/3805	3.26E-05	0.000207397
GO:0040036	regulation of fibroblast growth factor receptor signaling pathway	14/3805	3.31E-05	0.000209873
GO:0044319	wound healing, spreading of cells	14/3805	3.31E-05	0.000209873
GO:0090505	epiboly involved in wound healing	14/3805	3.31E-05	0.000209873
GO:0060441	epithelial tube branching involved in lung morphogenesis	15/3805	3.33E-05	0.000210909
GO:0048260	positive regulation of receptor-mediated endocytosis	21/3805	3.37E-05	0.000213385
GO:0007632	visual behavior	24/3805	3.38E-05	0.000213385

GO:0060419	heart growth	33/3805	3.50E-05	0.000221084
GO:0050729	positive regulation of inflammatory response	37/3805	3.52E-05	0.000222344
GO:0002244	hematopoietic progenitor cell differentiation	42/3805	3.58E-05	0.000225872
GO:0045217	cell-cell junction maintenance	Nov-05	3.63E-05	0.000228013
GO:0060307	regulation of ventricular cardiac muscle cell membrane repolarization	Nov-05	3.63E-05	0.000228013
GO:0060602	branch elongation of an epithelium	Nov-05	3.63E-05	0.000228013
GO:0072028	nephron morphogenesis	26/3805	3.67E-05	0.000229895
GO:1902893	regulation of miRNA transcription	26/3805	3.67E-05	0.000229895
GO:0060079	excitatory postsynaptic potential	27/3805	3.74E-05	0.00023422
GO:0034766	negative regulation of ion transmembrane transport	30/3805	3.74E-05	0.00023422
GO:0045185	maintenance of protein location	29/3805	3.78E-05	0.000235892
GO:0070555	response to interleukin-1	28/3805	3.78E-05	0.000235892
GO:0050819	negative regulation of coagulation	18/3805	3.79E-05	0.000236206
GO:1903053	regulation of extracellular matrix organization	18/3805	3.79E-05	0.000236206
GO:0031647	regulation of protein stability	67/3805	3.80E-05	0.000236657
GO:0010662	regulation of striated muscle cell apoptotic process	20/3805	3.92E-05	0.000243521
GO:0097035	regulation of membrane lipid distribution	20/3805	3.92E-05	0.000243521
GO:0055007	cardiac muscle cell differentiation	38/3805	3.93E-05	0.000243857

GO:0030282	bone mineralization	34/3805	4.03E-05	0.000250043
GO:0060997	dendritic spine morphogenesis	23/3805	4.04E-05	0.000250043
GO:0070482	response to oxygen levels	65/3805	4.13E-05	0.00025559
GO:0045494	photoreceptor cell maintenance	17/3805	4.20E-05	0.000259676
GO:0099504	synaptic vesicle cycle	52/3805	4.26E-05	0.000263194
GO:0061180	mammary gland epithelium development	24/3805	4.27E-05	0.000263536
GO:0030859	polarized epithelial cell differentiation	Dec-05	4.41E-05	0.000270272
GO:0045737	positive regulation of cyclin-dependent protein serine/threonine kinase activity	Dec-05	4.41E-05	0.000270272
GO:0061339	establishment or maintenance of monopolar cell polarity	Dec-05	4.41E-05	0.000270272
GO:0070293	renal absorption	Dec-05	4.41E-05	0.000270272
GO:0099623	regulation of cardiac muscle cell membrane repolarization	Dec-05	4.41E-05	0.000270272
GO:2000050	regulation of non-canonical Wnt signaling pathway	Dec-05	4.41E-05	0.000270272
GO:0072078	nephron tubule morphogenesis	25/3805	4.44E-05	0.000272271
GO:0071478	cellular response to radiation	43/3805	4.47E-05	0.000273362
GO:0098727	maintenance of cell number	43/3805	4.47E-05	0.000273362
GO:2001242	regulation of intrinsic apoptotic signaling pathway	43/3805	4.47E-05	0.000273362
GO:0010596	negative regulation of endothelial cell migration	19/3805	4.51E-05	0.000275402
GO:0021675	nerve development	26/3805	4.56E-05	0.000277855

GO:0030168	platelet activation	27/3805	4.62E-05	0.000281231
GO:0000212	meiotic spindle organization	Oct-05	4.70E-05	0.000285496
GO:0040037	negative regulation of fibroblast growth factor receptor signaling pathway	Oct-05	4.70E-05	0.000285496
GO:0045198	establishment of epithelial cell apical/basal polarity	Oct-05	4.70E-05	0.000285496
GO:0055003	cardiac myofibril assembly	Oct-05	4.70E-05	0.000285496
GO:0002366	leukocyte activation involved in immune response	69/3805	4.73E-05	0.000287134
GO:0051926	negative regulation of calcium ion transport	22/3805	4.81E-05	0.000291163
GO:0002029	desensitization of G protein-coupled receptor signaling pathway	13/3805	4.83E-05	0.000291592
GO:0006929	substrate-dependent cell migration	13/3805	4.83E-05	0.000291592
GO:0022401	negative adaptation of signaling pathway	13/3805	4.83E-05	0.000291592
GO:0051928	positive regulation of calcium ion transport	37/3805	4.87E-05	0.00029369
GO:0060306	regulation of membrane repolarization	14/3805	4.94E-05	0.00029809
GO:0015748	organophosphate ester transport	33/3805	4.99E-05	0.00030035
GO:0030534	adult behavior	45/3805	5.10E-05	0.000306649
GO:0008038	neuron recognition	18/3805	5.14E-05	0.000308826
GO:0044380	protein localization to cytoskeleton	20/3805	5.15E-05	0.000309458
GO:0022404	molting cycle process	32/3805	5.17E-05	0.000309929
GO:0022405	hair cycle process	32/3805	5.17E-05	0.000309929

GO:0007519	skeletal muscle tissue development	48/3805	5.24E-05	0.000313718
GO:0050821	protein stabilization	46/3805	5.39E-05	0.000322734
GO:0045638	negative regulation of myeloid cell differentiation	26/3805	5.63E-05	0.000336432
GO:0061614	miRNA transcription	26/3805	5.63E-05	0.000336432
GO:0003229	ventricular cardiac muscle tissue development	21/3805	5.70E-05	0.000339656
GO:0048706	embryonic skeletal system development	37/3805	5.70E-05	0.000339656
GO:0048103	somatic stem cell division	Sep-05	5.75E-05	0.000341909
GO:0090128	regulation of synapse maturation	Sep-05	5.75E-05	0.000341909
GO:0003149	membranous septum morphogenesis	Jul-05	5.79E-05	0.000344024
GO:0099640	axo-dendritic protein transport	Jul-05	5.79E-05	0.000344024
GO:0060402	calcium ion transport into cytosol	43/3805	5.93E-05	0.000351575
GO:0010665	regulation of cardiac muscle cell apoptotic process	19/3805	5.99E-05	0.000353788
GO:0043507	positive regulation of JUN kinase activity	19/3805	5.99E-05	0.000353788
GO:0043536	positive regulation of blood vessel endothelial cell migration	19/3805	5.99E-05	0.000353788
GO:0072698	protein localization to microtubule cytoskeleton	19/3805	5.99E-05	0.000353788
GO:0035089	establishment of apical/basal cell polarity	Nov-05	6.12E-05	0.000361226
GO:1902992	negative regulation of amyloid precursor protein catabolic process	Nov-05	6.12E-05	0.000361226
GO:0007498	mesoderm development	32/3805	6.17E-05	0.000363076

GO:0042472	inner ear morphogenesis	32/3805	6.17E-05	0.000363076
GO:0003093	regulation of glomerular filtration	Aug-05	6.36E-05	0.000372621
GO:0043589	skin morphogenesis	Aug-05	6.36E-05	0.000372621
GO:0072393	microtubule anchoring at microtubule organizing center	Aug-05	6.36E-05	0.000372621
GO:1903651	positive regulation of cytoplasmic transport	Aug-05	6.36E-05	0.000372621
GO:0007093	mitotic cell cycle checkpoint signaling	35/3805	6.37E-05	0.000372621
GO:0009582	detection of abiotic stimulus	35/3805	6.37E-05	0.000372621
GO:0045601	regulation of endothelial cell differentiation	16/3805	6.38E-05	0.00037307
GO:1903076	regulation of protein localization to plasma membrane	31/3805	6.39E-05	0.000373322
GO:0051048	negative regulation of secretion	47/3805	6.47E-05	0.000377631
GO:1903038	negative regulation of leukocyte cell-cell adhesion	37/3805	6.66E-05	0.000388323
GO:0051592	response to calcium ion	34/3805	6.70E-05	0.000390221
GO:0010656	negative regulation of muscle cell apoptotic process	20/3805	6.72E-05	0.000390525
GO:0140115	export across plasma membrane	20/3805	6.72E-05	0.000390525
GO:0071347	cellular response to interleukin-1	24/3805	6.72E-05	0.000390525
GO:0055081	anion homeostasis	18/3805	6.89E-05	0.000400121
GO:0061053	somite development	27/3805	6.93E-05	0.000401563
GO:0046883	regulation of hormone secretion	65/3805	6.94E-05	0.000401563

GO:0008593	regulation of Notch signaling pathway	26/3805	6.94E-05	0.000401563
GO:0021846	cell proliferation in forebrain	14/3805	7.24E-05	0.000418066
GO:1901099	negative regulation of signal transduction in absence of ligand	14/3805	7.24E-05	0.000418066
GO:2001240	negative regulation of extrinsic apoptotic signaling pathway in absence of ligand	14/3805	7.24E-05	0.000418066
GO:0071711	basement membrane organization	13/3805	7.31E-05	0.000420851
GO:0099622	cardiac muscle cell membrane repolarization	13/3805	7.31E-05	0.000420851
GO:1905523	positive regulation of macrophage migration	13/3805	7.31E-05	0.000420851
GO:0032635	interleukin-6 production	42/3805	7.34E-05	0.000422023
GO:0016079	synaptic vesicle exocytosis	35/3805	7.48E-05	0.000429613
GO:0042098	T cell proliferation	51/3805	7.56E-05	0.000434277
GO:1902895	positive regulation of miRNA transcription	22/3805	7.80E-05	0.000447279
GO:0010799	regulation of peptidyl-threonine phosphorylation	17/3805	7.85E-05	0.000448746
GO:0030195	negative regulation of blood coagulation	17/3805	7.85E-05	0.000448746
GO:0033628	regulation of cell adhesion mediated by integrin	17/3805	7.85E-05	0.000448746
GO:0010463	mesenchymal cell proliferation	19/3805	7.88E-05	0.000449692
GO:0030514	negative regulation of BMP signaling pathway	19/3805	7.88E-05	0.000449692
GO:0032642	regulation of chemokine production	30/3805	7.90E-05	0.000450358
GO:0051250	negative regulation of lymphocyte activation	41/3805	7.90E-05	0.000450358

GO:0042471	ear morphogenesis	36/3805	8.26E-05	0.000470081
GO:0002031	G protein-coupled receptor internalization	Oct-05	8.29E-05	0.000470848
GO:1902430	negative regulation of amyloid-beta formation	Oct-05	8.29E-05	0.000470848
GO:1904754	positive regulation of vascular associated smooth muscle cell migration	Oct-05	8.29E-05	0.000470848
GO:0003407	neural retina development	24/3805	8.37E-05	0.000474628
GO:0002699	positive regulation of immune effector process	65/3805	8.48E-05	0.000480911
GO:0030879	mammary gland development	40/3805	8.50E-05	0.000481314
GO:0031122	cytoplasmic microtubule organization	20/3805	8.70E-05	0.000491741
GO:1903078	positive regulation of protein localization to plasma membrane	20/3805	8.70E-05	0.000491741
GO:2000377	regulation of reactive oxygen species metabolic process	39/3805	9.13E-05	0.000515544
GO:0038066	p38MAPK cascade	18/3805	9.16E-05	0.000515782
GO:0072091	regulation of stem cell proliferation	18/3805	9.16E-05	0.000515782
GO:0097720	calcineurin-mediated signaling	18/3805	9.16E-05	0.000515782
GO:0072593	reactive oxygen species metabolic process	54/3805	9.20E-05	0.000517907
GO:0050868	negative regulation of T cell activation	34/3805	9.27E-05	0.000521178
GO:0021987	cerebral cortex development	30/3805	9.44E-05	0.000529608
GO:0055017	cardiac muscle tissue growth	30/3805	9.44E-05	0.000529608
GO:0070849	response to epidermal growth factor	15/3805	9.69E-05	0.000543468

GO:0097553	calcium ion transmembrane import into cytosol	38/3805	9.79E-05	0.000548387
GO:2000179	positive regulation of neural precursor cell proliferation	22/3805	9.84E-05	0.000550798
GO:0032331	negative regulation of chondrocyte differentiation	Nov-05	9.96E-05	0.000555361
GO:0036303	lymph vessel morphogenesis	Nov-05	9.96E-05	0.000555361
GO:0051984	positive regulation of chromosome segregation	Nov-05	9.96E-05	0.000555361
GO:0098901	regulation of cardiac muscle cell action potential	Nov-05	9.96E-05	0.000555361
GO:0001895	retina homeostasis	19/3805	0.000102743	0.000572462
GO:0060042	retina morphogenesis in camera-type eye	24/3805	0.000103615	0.000576808
GO:0008045	motor neuron axon guidance	14/3805	0.000104164	0.000578783
GO:0120316	sperm flagellum assembly	14/3805	0.000104164	0.000578783
GO:0035418	protein localization to synapse	25/3805	0.000104248	0.000578783
GO:0050870	positive regulation of T cell activation	50/3805	0.000104835	0.000581525
GO:0033173	calcineurin-NFAT signaling cascade	17/3805	0.000105447	0.000583366
GO:1900047	negative regulation of hemostasis	17/3805	0.000105447	0.000583366
GO:2001239	regulation of extrinsic apoptotic signaling pathway in absence of ligand	17/3805	0.000105447	0.000583366
GO:1902107	positive regulation of leukocyte differentiation	44/3805	0.000107154	0.00059165
GO:1903708	positive regulation of hemopoiesis	44/3805	0.000107154	0.00059165
GO:0003094	glomerular filtration	Dec-05	0.000107458	0.00059165

GO:0048634	regulation of muscle organ development	Dec-05	0.000107458	0.00059165
GO:0032602	chemokine production	31/3805	0.000107767	0.00059165
GO:0060078	regulation of postsynaptic membrane potential	31/3805	0.000107767	0.00059165
GO:0045655	regulation of monocyte differentiation	Sep-05	0.000107797	0.00059165
GO:0051895	negative regulation of focal adhesion assembly	Sep-05	0.000107797	0.00059165
GO:0150118	negative regulation of cell-substrate junction organization	Sep-05	0.000107797	0.00059165
GO:0023058	adaptation of signaling pathway	13/3805	0.000108288	0.000593304
GO:0060074	synapse maturation	13/3805	0.000108288	0.000593304
GO:0090090	negative regulation of canonical Wnt signaling pathway	34/3805	0.000108653	0.000594783
GO:0090316	positive regulation of intracellular protein transport	36/3805	0.000111981	0.000612461
GO:0060538	skeletal muscle organ development	49/3805	0.000114575	0.000626101
GO:0014013	regulation of gliogenesis	33/3805	0.000114975	0.000627741
GO:0017157	regulation of exocytosis	54/3805	0.000115121	0.000627988
GO:0007585	respiratory gaseous exchange by respiratory system	21/3805	0.000118636	0.000646031
GO:0090307	mitotic spindle assembly	21/3805	0.000118636	0.000646031
GO:0019827	stem cell population maintenance	41/3805	0.000119452	0.000649912
GO:0001569	branching involved in blood vessel morphogenesis	16/3805	0.000119936	0.000651976
GO:0031103	axon regeneration	18/3805	0.000120534	0.000653522

GO:0035272	exocrine system development	18/3805	0.000120534	0.000653522
GO:0045604	regulation of epidermal cell differentiation	18/3805	0.000120534	0.000653522
GO:1904063	negative regulation of cation transmembrane transport	27/3805	0.000123784	0.00067056
GO:0044728	DNA methylation or demethylation	26/3805	0.000126165	0.000682868
GO:0032677	regulation of interleukin-8 production	23/3805	0.000126409	0.000683593
GO:0031397	negative regulation of protein ubiquitination	25/3805	0.000127539	0.000688755
GO:0008625	extrinsic apoptotic signaling pathway via death domain receptors	24/3805	0.000127694	0.000688755
GO:0033209	tumor necrosis factor-mediated signaling pathway	24/3805	0.000127694	0.000688755
GO:0051764	actin crosslink formation	Aug-05	0.000131071	0.000705751
GO:0086069	bundle of His cell to Purkinje myocyte communication	Aug-05	0.000131071	0.000705751
GO:0051155	positive regulation of striated muscle cell differentiation	19/3805	0.000132933	0.00071516
GO:0035886	vascular associated smooth muscle cell differentiation	15/3805	0.000134291	0.00072122
GO:0086065	cell communication involved in cardiac conduction	15/3805	0.000134291	0.00072122
GO:0009100	glycoprotein metabolic process	68/3805	0.000137158	0.000735989
GO:0001946	lymphangiogenesis	Oct-05	0.000139573	0.000747022
GO:0003177	pulmonary valve development	Oct-05	0.000139573	0.000747022
GO:0007252	I-kappaB phosphorylation	Oct-05	0.000139573	0.000747022
GO:0021532	neural tube patterning	17/3805	0.000140247	0.000749987

GO:0010968	regulation of microtubule nucleation	Jul-05	0.000141076	0.000752488
GO:0061517	macrophage proliferation	Jul-05	0.000141076	0.000752488
GO:0072102	glomerulus morphogenesis	Jul-05	0.000141076	0.000752488
GO:0051310	metaphase plate congression	20/3805	0.000142561	0.000758865
GO:0002702	positive regulation of production of molecular mediator of immune response	32/3805	0.000142637	0.000758865
GO:0022037	metencephalon development	32/3805	0.000142637	0.000758865
GO:0010952	positive regulation of peptidase activity	42/3805	0.000143754	0.00076416
GO:0022617	extracellular matrix disassembly	14/3805	0.000147302	0.000781023
GO:0071364	cellular response to epidermal growth factor stimulus	14/3805	0.000147302	0.000781023
GO:1904707	positive regulation of vascular associated smooth muscle cell proliferation	14/3805	0.000147302	0.000781023
GO:0009791	post-embryonic development	34/3805	0.000148157	0.000784892
GO:0006940	regulation of smooth muscle contraction	21/3805	0.000149512	0.000790426
GO:0060425	lung morphogenesis	21/3805	0.000149512	0.000790426
GO:0032943	mononuclear cell proliferation	69/3805	0.000149581	0.000790426
GO:0060759	regulation of response to cytokine stimulus	36/3805	0.000150537	0.000794805
GO:0051983	regulation of chromosome segregation	26/3805	0.000152734	0.000805721
GO:0030032	lamellipodium assembly	22/3805	0.000153992	0.000811673
GO:0035914	skeletal muscle cell differentiation	25/3805	0.000155351	0.000817456

GO:1904427	positive regulation of calcium ion transmembrane transport	25/3805	0.000155351	0.000817456
GO:0000075	cell cycle checkpoint signaling	41/3805	0.000155951	0.000819922
GO:0032637	interleukin-8 production	23/3805	0.000156265	0.000820134
GO:0071277	cellular response to calcium ion	23/3805	0.000156265	0.000820134
GO:0034114	regulation of heterotypic cell-cell adhesion	Nov-05	0.000156647	0.000820134
GO:0044331	cell-cell adhesion mediated by cadherin	Nov-05	0.000156647	0.000820134
GO:0055083	monovalent inorganic anion homeostasis	Nov-05	0.000156647	0.000820134
GO:1905314	semi-lunar valve development	13/3805	0.000157162	0.000822139
GO:1901216	positive regulation of neuron death	33/3805	0.000157639	0.000823943
GO:1990845	adaptive thermogenesis	39/3805	0.000159869	0.000834902
GO:0060444	branching involved in mammary gland duct morphogenesis	Dec-05	0.00016135	0.000840525
GO:1903649	regulation of cytoplasmic transport	Dec-05	0.00016135	0.000840525
GO:1904031	positive regulation of cyclin-dependent protein kinase activity	Dec-05	0.00016135	0.000840525
GO:0048536	spleen development	16/3805	0.000161507	0.000840643
GO:0030216	keratinocyte differentiation	37/3805	0.000161809	0.000841513
GO:0055006	cardiac cell development	29/3805	0.000165516	0.000860076
GO:0002548	monocyte chemotaxis	19/3805	0.000170658	0.000883856
GO:0048278	vesicle docking	19/3805	0.000170658	0.000883856

GO:0060632	regulation of microtubule-based movement	19/3805	0.000170658	0.000883856
GO:2001238	positive regulation of extrinsic apoptotic signaling pathway	19/3805	0.000170658	0.000883856
GO:2000241	regulation of reproductive process	47/3805	0.000173484	0.00089775
GO:0070663	regulation of leukocyte proliferation	57/3805	0.00018267	0.000944499
GO:0050714	positive regulation of protein secretion	39/3805	0.000183044	0.000945143
GO:0001774	microglial cell activation	15/3805	0.000183551	0.000945143
GO:0006904	vesicle docking involved in exocytosis	15/3805	0.000183551	0.000945143
GO:0007143	female meiotic nuclear division	15/3805	0.000183551	0.000945143
GO:1900744	regulation of p38MAPK cascade	15/3805	0.000183551	0.000945143
GO:1904407	positive regulation of nitric oxide metabolic process	17/3805	0.000184685	0.000950199
GO:0071897	DNA biosynthetic process	42/3805	0.000185933	0.000955831
GO:0010827	regulation of glucose transmembrane transport	25/3805	0.00018842	0.000967027
GO:1901379	regulation of potassium ion transmembrane transport	25/3805	0.00018842	0.000967027
GO:0035633	maintenance of blood-brain barrier	Sep-05	0.000190315	0.000973551
GO:0061318	renal filtration cell differentiation	Sep-05	0.000190315	0.000973551
GO:0072112	glomerular visceral epithelial cell differentiation	Sep-05	0.000190315	0.000973551
GO:0072311	glomerular epithelial cell differentiation	Sep-05	0.000190315	0.000973551
GO:0043280	positive regulation of cysteine-type endopeptidase activity involved in apoptotic process	32/3805	0.000195682	0.00100019

GO:0099175	regulation of postsynapse organization	29/3805	0.000196127	0.001001644
GO:0009411	response to UV	36/3805	0.000200633	0.001022151
GO:0045598	regulation of fat cell differentiation	36/3805	0.000200633	0.001022151
GO:2001056	positive regulation of cysteine-type endopeptidase activity	36/3805	0.000200633	0.001022151
GO:0048016	inositol phosphate-mediated signaling	18/3805	0.000203346	0.001035123
GO:0060236	regulation of mitotic spindle organization	14/3805	0.000205045	0.001041227
GO:0072210	metanephric nephron development	14/3805	0.000205045	0.001041227
GO:0086004	regulation of cardiac muscle cell contraction	14/3805	0.000205045	0.001041227
GO:0009952	anterior/posterior pattern specification	51/3805	0.000211643	0.001073861
GO:0062207	regulation of pattern recognition receptor signaling pathway	27/3805	0.000213643	0.001083129
GO:0007281	germ cell development	76/3805	0.000214021	0.001084164
GO:0003016	respiratory system process	16/3805	0.000215068	0.001085946
GO:0043114	regulation of vascular permeability	16/3805	0.000215068	0.001085946
GO:1903524	positive regulation of blood circulation	16/3805	0.000215068	0.001085946
GO:2000404	regulation of T cell migration	16/3805	0.000215068	0.001085946
GO:0010812	negative regulation of cell-substrate adhesion	19/3805	0.000217455	0.001096225
GO:0032731	positive regulation of interleukin-1 beta production	19/3805	0.000217455	0.001096225
GO:0070509	calcium ion import	26/3805	0.00022121	0.001114256

GO:0048013	ephrin receptor signaling pathway	13/3805	0.000223845	0.001124808
GO:0099560	synaptic membrane adhesion	13/3805	0.000223845	0.001124808
GO:1903959	regulation of anion transmembrane transport	13/3805	0.000223845	0.001124808
GO:0031579	membrane raft organization	Oct-05	0.000225646	0.001129313
GO:0046325	negative regulation of glucose import	Oct-05	0.000225646	0.001129313
GO:0051383	kinetochore organization	Oct-05	0.000225646	0.001129313
GO:0061081	positive regulation of myeloid leukocyte cytokine production involved in immune response	Oct-05	0.000225646	0.001129313
GO:2000810	regulation of bicellular tight junction assembly	Oct-05	0.000225646	0.001129313
GO:0010876	lipid localization	86/3805	0.000226644	0.001133402
GO:0001836	release of cytochrome c from mitochondria	20/3805	0.00022726	0.00113557
GO:0060420	regulation of heart growth	25/3805	0.00022758	0.001136265
GO:0071346	cellular response to interferon-gamma	29/3805	0.00023168	0.001154249
GO:0034341	response to interferon-gamma	34/3805	0.000231737	0.001154249
GO:0051588	regulation of neurotransmitter transport	34/3805	0.000231737	0.001154249
GO:0010921	regulation of phosphatase activity	24/3805	0.000232393	0.001156594
GO:0021885	forebrain cell migration	21/3805	0.000233166	0.001157676
GO:0034121	regulation of toll-like receptor signaling pathway	21/3805	0.000233166	0.001157676
GO:2001244	positive regulation of intrinsic apoptotic signaling pathway	21/3805	0.000233166	0.001157676

GO:0006305	DNA alkylation	22/3805	0.000235658	0.001167263
GO:0006306	DNA methylation	22/3805	0.000235658	0.001167263
GO:1903036	positive regulation of response to wounding	22/3805	0.000235658	0.001167263
GO:0002819	regulation of adaptive immune response	51/3805	0.000235875	0.001167414
GO:0060563	neuroepithelial cell differentiation	Dec-05	0.000236735	0.001169815
GO:0090025	regulation of monocyte chemotaxis	Dec-05	0.000236735	0.001169815
GO:0006582	melanin metabolic process	Nov-05	0.000239219	0.001181155
GO:0045839	negative regulation of mitotic nuclear division	17/3805	0.000240909	0.001186687
GO:0090279	regulation of calcium ion import	17/3805	0.000240909	0.001186687
GO:1902041	regulation of extrinsic apoptotic signaling pathway via death domain receptors	17/3805	0.000240909	0.001186687
GO:0015833	peptide transport	65/3805	0.000242253	0.001192363
GO:1903320	regulation of protein modification by small protein conjugation or removal	54/3805	0.000243434	0.001197235
GO:0030072	peptide hormone secretion	60/3805	0.000247533	0.001214908
GO:0030501	positive regulation of bone mineralization	15/3805	0.000247708	0.001214908
GO:0032924	activin receptor signaling pathway	15/3805	0.000247708	0.001214908
GO:0043455	regulation of secondary metabolic process	Aug-05	0.000248298	0.001214908
GO:0048021	regulation of melanin biosynthetic process	Aug-05	0.000248298	0.001214908
GO:1900376	regulation of secondary metabolite biosynthetic process	Aug-05	0.000248298	0.001214908

GO:0000018	regulation of DNA recombination	33/3805	0.000248389	0.001214908
GO:1903531	negative regulation of secretion by cell	40/3805	0.000249935	0.001221516
GO:0019748	secondary metabolic process	18/3805	0.000260845	0.001273835
GO:0098656	anion transmembrane transport	45/3805	0.000262601	0.001281411
GO:0045576	mast cell activation	26/3805	0.000264707	0.001289673
GO:0051781	positive regulation of cell division	26/3805	0.000264707	0.001289673
GO:0006470	protein dephosphorylation	53/3805	0.000267871	0.001304071
GO:0018205	peptidyl-lysine modification	77/3805	0.000269109	0.001309076
GO:0035282	segmentation	29/3805	0.000272847	0.001325197
GO:0045639	positive regulation of myeloid cell differentiation	29/3805	0.000272847	0.001325197
GO:0010171	body morphogenesis	19/3805	0.0002751	0.001331994
GO:0046850	regulation of bone remodeling	19/3805	0.0002751	0.001331994
GO:0051784	negative regulation of nuclear division	19/3805	0.0002751	0.001331994
GO:0060711	labyrinthine layer development	19/3805	0.0002751	0.001331994
GO:0002822	regulation of adaptive immune response based on somatic recombination of immune receptors built from immunoglobulin superfamily domains	48/3805	0.000280157	0.00135438
GO:0031396	regulation of protein ubiquitination	48/3805	0.000280157	0.00135438
GO:2000403	positive regulation of lymphocyte migration	14/3805	0.000281251	0.00135862
GO:0045123	cellular extravasation	23/3805	0.000286547	0.001383133

GO:0006029	proteoglycan metabolic process	22/3805	0.000289231	0.001395008
GO:0006869	lipid transport	76/3805	0.000296727	0.001430061
GO:0042886	amide transport	70/3805	0.000299546	0.001439571
GO:0033262	regulation of nuclear cell cycle DNA replication	Jul-05	0.000299853	0.001439571
GO:0038063	collagen-activated tyrosine kinase receptor signaling pathway	Jul-05	0.000299853	0.001439571
GO:0051798	positive regulation of hair follicle development	Jul-05	0.000299853	0.001439571
GO:0072176	nephric duct development	Jul-05	0.000299853	0.001439571
GO:0001704	formation of primary germ layer	27/3805	0.000301998	0.001448753
GO:0046660	female sex differentiation	36/3805	0.00030394	0.001456952
GO:0046651	lymphocyte proliferation	67/3805	0.000310705	0.001488237
GO:0002042	cell migration involved in sprouting angiogenesis	17/3805	0.000311426	0.00148827
GO:0045907	positive regulation of vasoconstriction	17/3805	0.000311426	0.00148827
GO:0070169	positive regulation of biomineral tissue development	17/3805	0.000311426	0.00148827
GO:0007020	microtubule nucleation	13/3805	0.000313323	0.001493903
GO:0035025	positive regulation of Rho protein signal transduction	13/3805	0.000313323	0.001493903
GO:1904738	vascular associated smooth muscle cell migration	13/3805	0.000313323	0.001493903
GO:0010544	negative regulation of platelet activation	Sep-05	0.000319267	0.001517612
GO:0022038	corpus callosum development	Sep-05	0.000319267	0.001517612

GO:0071636	positive regulation of transforming growth factor beta production	Sep-05	0.000319267	0.001517612
GO:1900117	regulation of execution phase of apoptosis	Sep-05	0.000319267	0.001517612
GO:0042552	myelination	40/3805	0.000321637	0.001527712
GO:0048814	regulation of dendrite morphogenesis	25/3805	0.000328035	0.001555736
GO:0070828	heterochromatin organization	25/3805	0.000328035	0.001555736
GO:0017145	stem cell division	15/3805	0.000330306	0.001564132
GO:1900026	positive regulation of substrate adhesion-dependent cell spreading	15/3805	0.000330306	0.001564132
GO:0032755	positive regulation of interleukin-6 production	28/3805	0.000338937	0.001602572
GO:0050886	endocrine process	28/3805	0.000338937	0.001602572
GO:0007288	sperm axoneme assembly	Dec-05	0.000340079	0.001605543
GO:1904752	regulation of vascular associated smooth muscle cell migration	Dec-05	0.000340079	0.001605543
GO:0010517	regulation of phospholipase activity	19/3805	0.000345631	0.00162929
GO:0043407	negative regulation of MAP kinase activity	19/3805	0.000345631	0.00162929
GO:0055021	regulation of cardiac muscle tissue growth	23/3805	0.000347441	0.001636588
GO:0048469	cell maturation	48/3805	0.000348696	0.001641261
GO:0009266	response to temperature stimulus	39/3805	0.0003506	0.001647739
GO:0010950	positive regulation of endopeptidase activity	39/3805	0.0003506	0.001647739
GO:0022029	telencephalon cell migration	20/3805	0.000353133	0.001658396

GO:0006346	DNA methylation-dependent heterochromatin assembly	Nov-05	0.000355714	0.00165929
GO:0010829	negative regulation of glucose transmembrane transport	Nov-05	0.000355714	0.00165929
GO:0030204	chondroitin sulfate metabolic process	Nov-05	0.000355714	0.00165929
GO:0051016	barbed-end actin filament capping	Nov-05	0.000355714	0.00165929
GO:0060716	labyrinthine layer blood vessel development	Nov-05	0.000355714	0.00165929
GO:0062009	secondary palate development	Nov-05	0.000355714	0.00165929
GO:0072012	glomerulus vasculature development	Nov-05	0.000355714	0.00165929
GO:0072567	chemokine (C-X-C motif) ligand 2 production	Nov-05	0.000355714	0.00165929
GO:2000341	regulation of chemokine (C-X-C motif) ligand 2 production	Nov-05	0.000355714	0.00165929
GO:0055013	cardiac muscle cell development	27/3805	0.000357238	0.001663913
GO:0070167	regulation of biomineral tissue development	27/3805	0.000357238	0.001663913
GO:0045429	positive regulation of nitric oxide biosynthetic process	16/3805	0.000369642	0.001719126
GO:0061900	glial cell activation	16/3805	0.000369642	0.001719126
GO:1903321	negative regulation of protein modification by small protein conjugation or removal	26/3805	0.000374922	0.001742383
GO:0060351	cartilage development involved in endochondral bone morphogenesis	14/3805	0.000380501	0.001765684
GO:1903670	regulation of sprouting angiogenesis	14/3805	0.000380501	0.001765684
GO:0071496	cellular response to external stimulus	61/3805	0.000392673	0.001820813
GO:0006023	aminoglycan biosynthetic process	17/3805	0.000399133	0.001841202

GO:0044060	regulation of endocrine process	17/3805	0.000399133	0.001841202
GO:0045773	positive regulation of axon extension	17/3805	0.000399133	0.001841202
GO:0048483	autonomic nervous system development	17/3805	0.000399133	0.001841202
GO:0048701	embryonic cranial skeleton morphogenesis	17/3805	0.000399133	0.001841202
GO:0070527	platelet aggregation	17/3805	0.000399133	0.001841202
GO:0110151	positive regulation of biomineralization	17/3805	0.000399133	0.001841202
GO:0071805	potassium ion transmembrane transport	45/3805	0.000414198	0.001909283
GO:0003170	heart valve development	18/3805	0.000419381	0.001924693
GO:0045071	negative regulation of viral genome replication	18/3805	0.000419381	0.001924693
GO:0045599	negative regulation of fat cell differentiation	18/3805	0.000419381	0.001924693
GO:0045778	positive regulation of ossification	18/3805	0.000419381	0.001924693
GO:0032722	positive regulation of chemokine production	23/3805	0.00041939	0.001924693
GO:0050000	chromosome localization	23/3805	0.00041939	0.001924693
GO:0031349	positive regulation of defense response	61/3805	0.000429743	0.00197076
GO:0019233	sensory perception of pain	35/3805	0.000430739	0.001971774
GO:0002053	positive regulation of mesenchymal cell proliferation	13/3805	0.000431543	0.001971774
GO:0034122	negative regulation of toll-like receptor signaling pathway	13/3805	0.000431543	0.001971774
GO:0048169	regulation of long-term neuronal synaptic plasticity	13/3805	0.000431543	0.001971774

GO:0120033	negative regulation of plasma membrane bounded cell projection assembly	13/3805	0.000431543	0.001971774
GO:0033135	regulation of peptidyl-serine phosphorylation	38/3805	0.000433211	0.001977949
GO:0034394	protein localization to cell surface	21/3805	0.000435155	0.001983927
GO:0060675	ureteric bud morphogenesis	21/3805	0.000435155	0.001983927
GO:1905521	regulation of macrophage migration	15/3805	0.000435491	0.001984008
GO:0038065	collagen-activated signaling pathway	Aug-05	0.000439118	0.001996165
GO:0055057	neuroblast division	Aug-05	0.000439118	0.001996165
GO:0060026	convergent extension	Aug-05	0.000439118	0.001996165
GO:0009798	axis specification	26/3805	0.000443846	0.002014724
GO:0051304	chromosome separation	26/3805	0.000443846	0.002014724
GO:0007272	ensheathment of neurons	40/3805	0.000463794	0.002102219
GO:0008366	axon ensheathment	40/3805	0.000463794	0.002102219
GO:0045685	regulation of glial cell differentiation	25/3805	0.000465615	0.002108943
GO:0008344	adult locomotory behavior	28/3805	0.000466307	0.002110545
GO:0050728	negative regulation of inflammatory response	34/3805	0.000466958	0.002111964
GO:0032873	negative regulation of stress-activated MAPK cascade	16/3805	0.000477593	0.00215227
GO:0035305	negative regulation of dephosphorylation	16/3805	0.000477593	0.00215227
GO:0043618	regulation of transcription from RNA polymerase II promoter in response to stress	16/3805	0.000477593	0.00215227

GO:0070303	negative regulation of stress-activated protein kinase signaling cascade	16/3805	0.000477593	0.00215227
GO:0090102	cochlea development	16/3805	0.000477593	0.00215227
GO:0030511	positive regulation of transforming growth factor beta receptor signaling pathway	Dec-05	0.000479154	0.002154639
GO:0060977	coronary vasculature morphogenesis	Dec-05	0.000479154	0.002154639
GO:1903846	positive regulation of cellular response to transforming growth factor beta stimulus	Dec-05	0.000479154	0.002154639
GO:0051651	maintenance of location in cell	48/3805	0.000479831	0.002156134
GO:0046888	negative regulation of hormone secretion	24/3805	0.000485918	0.002181916
GO:0006302	double-strand break repair	55/3805	0.000488175	0.002189276
GO:0006813	potassium ion transport	51/3805	0.000488258	0.002189276
GO:0006304	DNA modification	27/3805	0.000495024	0.002216431
GO:0110149	regulation of biomineralization	27/3805	0.000495024	0.002216431
GO:0015711	organic anion transport	69/3805	0.000500816	0.002240756
GO:0060998	regulation of dendritic spine development	23/3805	0.000504036	0.002253551
GO:0030838	positive regulation of actin filament polymerization	17/3805	0.000507354	0.002262299
GO:0043620	regulation of DNA-templated transcription in response to stress	17/3805	0.000507354	0.002262299
GO:0055010	ventricular cardiac muscle tissue morphogenesis	17/3805	0.000507354	0.002262299
GO:0010762	regulation of fibroblast migration	14/3805	0.000508166	0.002262299
GO:0010939	regulation of necrotic cell death	14/3805	0.000508166	0.002262299

GO:2000463	positive regulation of excitatory postsynaptic potential	14/3805	0.000508166	0.002262299
GO:0034349	glial cell apoptotic process	Sep-05	0.00051264	0.002277348
GO:0034501	protein localization to kinetochore	Sep-05	0.00051264	0.002277348
GO:1903083	protein localization to condensed chromosome	Sep-05	0.00051264	0.002277348
GO:0060445	branching involved in salivary gland morphogenesis	Nov-05	0.00051635	0.002285701
GO:0061437	renal system vasculature development	Nov-05	0.00051635	0.002285701
GO:0061440	kidney vasculature development	Nov-05	0.00051635	0.002285701
GO:1903055	positive regulation of extracellular matrix organization	Nov-05	0.00051635	0.002285701
GO:2000178	negative regulation of neural precursor cell proliferation	Nov-05	0.00051635	0.002285701
GO:0002790	peptide secretion	60/3805	0.000518545	0.002293019
GO:0051965	positive regulation of synapse assembly	22/3805	0.000519105	0.002293019
GO:1904888	cranial skeletal system development	22/3805	0.000519105	0.002293019
GO:0060021	roof of mouth development	26/3805	0.000523649	0.002311458
GO:0042987	amyloid precursor protein catabolic process	18/3805	0.000525977	0.002318457
GO:1900024	regulation of substrate adhesion-dependent cell spreading	18/3805	0.000525977	0.002318457
GO:0008217	regulation of blood pressure	47/3805	0.00052811	0.002326218
GO:0042982	amyloid precursor protein metabolic process	21/3805	0.0005301	0.002331695
GO:0072171	mesonephric tubule morphogenesis	21/3805	0.0005301	0.002331695

GO:0003283	atrial septum development	Oct-05	0.000532481	0.002333949
GO:0034405	response to fluid shear stress	Oct-05	0.000532481	0.002333949
GO:0042438	melanin biosynthetic process	Oct-05	0.000532481	0.002333949
GO:0048670	regulation of collateral sprouting	Oct-05	0.000532481	0.002333949
GO:0055064	chloride ion homeostasis	Oct-05	0.000532481	0.002333949
GO:0001954	positive regulation of cell-matrix adhesion	19/3805	0.000534954	0.002340431
GO:0032615	interleukin-12 production	19/3805	0.000534954	0.002340431
GO:0048662	negative regulation of smooth muscle cell proliferation	20/3805	0.000535833	0.002340431
GO:0097194	execution phase of apoptosis	20/3805	0.000535833	0.002340431
GO:1902017	regulation of cilium assembly	20/3805	0.000535833	0.002340431
GO:0043266	regulation of potassium ion transport	28/3805	0.000544516	0.002376692
GO:0051235	maintenance of location	67/3805	0.00056161	0.002449593
GO:0002269	leukocyte activation involved in inflammatory response	15/3805	0.000568063	0.002476011
GO:0044557	relaxation of smooth muscle	Jul-05	0.000575532	0.002503323
GO:0051639	actin filament network formation	Jul-05	0.000575532	0.002503323
GO:0090557	establishment of endothelial intestinal barrier	Jul-05	0.000575532	0.002503323
GO:0035270	endocrine system development	33/3805	0.000579009	0.002516694
GO:0006821	chloride transport	27/3805	0.000579969	0.002519116

GO:0010923	negative regulation of phosphatase activity	13/3805	0.000585507	0.002536113
GO:0045746	negative regulation of Notch signaling pathway	13/3805	0.000585507	0.002536113
GO:0051647	nucleus localization	13/3805	0.000585507	0.002536113
GO:0090313	regulation of protein targeting to membrane	13/3805	0.000585507	0.002536113
GO:0042476	odontogenesis	31/3805	0.000590553	0.00255562
GO:0038061	NIK/NF-kappaB signaling	29/3805	0.000591871	0.002560129
GO:0071241	cellular response to inorganic substance	50/3805	0.00059457	0.002570026
GO:1904950	negative regulation of establishment of protein localization	34/3805	0.000608889	0.002630099
GO:0007520	myoblast fusion	16/3805	0.000611494	0.002634063
GO:0008089	anterograde axonal transport	16/3805	0.000611494	0.002634063
GO:0043268	positive regulation of potassium ion transport	16/3805	0.000611494	0.002634063
GO:0045933	positive regulation of muscle contraction	16/3805	0.000611494	0.002634063
GO:0009615	response to virus	66/3805	0.000619608	0.002667175
GO:0045670	regulation of osteoclast differentiation	22/3805	0.000624954	0.002686485
GO:1902807	negative regulation of cell cycle G1/S phase transition	22/3805	0.000624954	0.002686485
GO:0016570	histone modification	91/3805	0.000627225	0.002694394
GO:0015698	inorganic anion transport	35/3805	0.000636424	0.002732031
GO:0061001	regulation of dendritic spine morphogenesis	17/3805	0.00063987	0.002744938

GO:0140056	organelle localization by membrane tethering	21/3805	0.000642524	0.002750655
GO:1901184	regulation of ERBB signaling pathway	21/3805	0.000642524	0.002750655
GO:1905818	regulation of chromosome separation	21/3805	0.000642524	0.002750655
GO:0002285	lymphocyte activation involved in immune response	47/3805	0.000650093	0.002781153
GO:0042733	embryonic digit morphogenesis	20/3805	0.000654526	0.002798202
GO:0045682	regulation of epidermis development	18/3805	0.000655126	0.002798849
GO:0002720	positive regulation of cytokine production involved in immune response	19/3805	0.00065934	0.002813006
GO:0050891	multicellular organismal water homeostasis	19/3805	0.00065934	0.002813006
GO:0045667	regulation of osteoblast differentiation	36/3805	0.000661591	0.002817706
GO:0051224	negative regulation of protein transport	33/3805	0.00066616	0.002817706
GO:0033687	osteoblast proliferation	Dec-05	0.000663149	0.002817706
GO:0051642	centrosome localization	Dec-05	0.000663149	0.002817706
GO:0060740	prostate gland epithelium morphogenesis	Dec-05	0.000663149	0.002817706
GO:2000108	positive regulation of leukocyte apoptotic process	Dec-05	0.000663149	0.002817706
GO:0090224	regulation of spindle organization	14/3805	0.000670474	0.002844959
GO:0110110	positive regulation of animal organ morphogenesis	14/3805	0.000670474	0.002844959
GO:0002237	response to molecule of bacterial origin	77/3805	0.000678004	0.002874959
GO:0033138	positive regulation of peptidyl-serine phosphorylation	31/3805	0.000678894	0.002876778

GO:1900182	positive regulation of protein localization to nucleus	24/3805	0.000685994	0.00289681
GO:0001765	membrane raft assembly	Jun-05	0.000688259	0.00289681
GO:0006971	hypotonic response	Jun-05	0.000688259	0.00289681
GO:0010216	maintenance of DNA methylation	Jun-05	0.000688259	0.00289681
GO:0021957	corticospinal tract morphogenesis	Jun-05	0.000688259	0.00289681
GO:0036363	transforming growth factor beta activation	Jun-05	0.000688259	0.00289681
GO:0038130	ERBB4 signaling pathway	Jun-05	0.000688259	0.00289681
GO:0048671	negative regulation of collateral sprouting	Jun-05	0.000688259	0.00289681
GO:0090306	meiotic spindle assembly	Jun-05	0.000688259	0.00289681
GO:1900119	positive regulation of execution phase of apoptosis	Jun-05	0.000688259	0.00289681
GO:0007626	locomotory behavior	54/3805	0.000713515	0.003001086
GO:0045995	regulation of embryonic development	23/3805	0.000718926	0.00302181
GO:0003148	outflow tract septum morphogenesis	Nov-05	0.000733295	0.003071198
GO:0006024	glycosaminoglycan biosynthetic process	15/3805	0.00073353	0.003071198
GO:0071300	cellular response to retinoic acid	15/3805	0.00073353	0.003071198
GO:0051014	actin filament severing	Aug-05	0.000733626	0.003071198
GO:0061308	cardiac neural crest cell development involved in heart development	Aug-05	0.000733626	0.003071198
GO:1902285	semaphorin-plexin signaling pathway involved in neuron projection guidance	Aug-05	0.000733626	0.003071198

GO:0014015	positive regulation of gliogenesis	22/3805	0.000749036	0.003133611
GO:0060428	lung epithelium development	16/3805	0.000776198	0.003245071
GO:0007413	axonal fasciculation	Oct-05	0.000783037	0.003253181
GO:0010759	positive regulation of macrophage chemotaxis	Oct-05	0.000783037	0.003253181
GO:0035024	negative regulation of Rho protein signal transduction	Oct-05	0.000783037	0.003253181
GO:0035988	chondrocyte proliferation	Oct-05	0.000783037	0.003253181
GO:0062098	regulation of programmed necrotic cell death	Oct-05	0.000783037	0.003253181
GO:0106030	neuron projection fasciculation	Oct-05	0.000783037	0.003253181
GO:0150105	protein localization to cell-cell junction	Oct-05	0.000783037	0.003253181
GO:0051294	establishment of spindle orientation	13/3805	0.000783347	0.003253181
GO:1901385	regulation of voltage-gated calcium channel activity	13/3805	0.000783347	0.003253181
GO:1902003	regulation of amyloid-beta formation	13/3805	0.000783347	0.003253181
GO:0032332	positive regulation of chondrocyte differentiation	Sep-05	0.00079244	0.003280042
GO:0035994	response to muscle stretch	Sep-05	0.00079244	0.003280042
GO:0045606	positive regulation of epidermal cell differentiation	Sep-05	0.00079244	0.003280042
GO:0090594	inflammatory response to wounding	Sep-05	0.00079244	0.003280042
GO:1903909	regulation of receptor clustering	Sep-05	0.00079244	0.003280042
GO:1903317	regulation of protein maturation	20/3805	0.000795247	0.003289479

GO:0006493	protein O-linked glycosylation	17/3805	0.000800956	0.003304341
GO:0031529	ruffle organization	17/3805	0.000800956	0.003304341
GO:1901861	regulation of muscle tissue development	17/3805	0.000800956	0.003304341
GO:1903533	regulation of protein targeting	17/3805	0.000800956	0.003304341
GO:0021575	hindbrain morphogenesis	18/3805	0.000810592	0.003337478
GO:0050982	detection of mechanical stimulus	18/3805	0.000810592	0.003337478
GO:0070098	chemokine-mediated signaling pathway	18/3805	0.000810592	0.003337478
GO:0001959	regulation of cytokine-mediated signaling pathway	32/3805	0.000820248	0.003375012
GO:0006865	amino acid transport	36/3805	0.00084481	0.003473786
GO:0050766	positive regulation of phagocytosis	23/3805	0.000853419	0.003506877
GO:0032944	regulation of mononuclear cell proliferation	51/3805	0.000873801	0.003588273
GO:0045823	positive regulation of heart contraction	14/3805	0.000874568	0.003589061
GO:0002092	positive regulation of receptor internalization	Dec-05	0.000902769	0.003692662
GO:0048799	animal organ maturation	Dec-05	0.000902769	0.003692662
GO:0060512	prostate gland morphogenesis	Dec-05	0.000902769	0.003692662
GO:0061842	microtubule organizing center localization	Dec-05	0.000902769	0.003692662
GO:2000406	positive regulation of T cell migration	Dec-05	0.000902769	0.003692662
GO:1901800	positive regulation of proteasomal protein catabolic process	27/3805	0.000915961	0.003744169

GO:0006919	activation of cysteine-type endopeptidase activity involved in apoptotic process	21/3805	0.000930414	0.003800761
GO:0003382	epithelial cell morphogenesis	15/3805	0.000938146	0.003827343
GO:0043403	skeletal muscle tissue regeneration	15/3805	0.000938146	0.003827343
GO:0001541	ovarian follicle development	20/3805	0.00096124	0.003916442
GO:0032732	positive regulation of interleukin-1 production	20/3805	0.00096124	0.003916442
GO:0043123	positive regulation of I-kappaB kinase/NF-kappaB signaling	30/3805	0.000966676	0.003933459
GO:0051101	regulation of DNA binding	30/3805	0.000966676	0.003933459
GO:0030522	intracellular receptor signaling pathway	46/3805	0.000970626	0.00394696
GO:0007131	reciprocal meiotic recombination	16/3805	0.000977181	0.003965869
GO:0021795	cerebral cortex cell migration	16/3805	0.000977181	0.003965869
GO:0140527	reciprocal homologous recombination	16/3805	0.000977181	0.003965869
GO:0060389	pathway-restricted SMAD protein phosphorylation	19/3805	0.000984167	0.003991626
GO:0043666	regulation of phosphoprotein phosphatase activity	17/3805	0.000995405	0.004034098
GO:0051785	positive regulation of nuclear division	18/3805	0.000996576	0.004034098
GO:1905517	macrophage migration	18/3805	0.000996576	0.004034098
GO:0034644	cellular response to UV	23/3805	0.001009133	0.004082284
GO:0006750	glutathione biosynthetic process	Jul-05	0.001019993	0.004092283
GO:0007144	female meiosis I	Jul-05	0.001019993	0.004092283

GO:0010359	regulation of anion channel activity	Jul-05	0.001019993	0.004092283
GO:0016081	synaptic vesicle docking	Jul-05	0.001019993	0.004092283
GO:0035791	platelet-derived growth factor receptor-beta signaling pathway	Jul-05	0.001019993	0.004092283
GO:0051152	positive regulation of smooth muscle cell differentiation	Jul-05	0.001019993	0.004092283
GO:0060346	bone trabecula formation	Jul-05	0.001019993	0.004092283
GO:1905063	regulation of vascular associated smooth muscle cell differentiation	Jul-05	0.001019993	0.004092283
GO:2000105	positive regulation of DNA-templated DNA replication	Jul-05	0.001019993	0.004092283
GO:0030947	regulation of vascular endothelial growth factor receptor signaling pathway	Nov-05	0.001020778	0.004092283
GO:0045932	negative regulation of muscle contraction	Nov-05	0.001020778	0.004092283
GO:0048745	smooth muscle tissue development	Nov-05	0.001020778	0.004092283
GO:0060343	trabecula formation	Nov-05	0.001020778	0.004092283
GO:0140058	neuron projection arborization	Nov-05	0.001020778	0.004092283
GO:0010522	regulation of calcium ion transport into cytosol	27/3805	0.001060492	0.004248771
GO:1903825	organic acid transmembrane transport	32/3805	0.001063215	0.00425695
GO:0048545	response to steroid hormone	47/3805	0.001070952	0.00428518
GO:0042770	signal transduction in response to DNA damage	37/3805	0.001095905	0.004382216
GO:0071621	granulocyte chemotaxis	30/3805	0.001105714	0.004418611
GO:0044550	secondary metabolite biosynthetic process	Oct-05	0.001122923	0.004481648

GO:0071459	protein localization to chromosome, centromeric region	Oct-05	0.001122923	0.004481648
GO:0099084	postsynaptic specialization organization	14/3805	0.001128557	0.004498383
GO:1901021	positive regulation of calcium ion transmembrane transporter activity	14/3805	0.001128557	0.004498383
GO:1904659	glucose transmembrane transport	28/3805	0.001133418	0.004514877
GO:0046631	alpha-beta T cell activation	40/3805	0.001149753	0.004577027
GO:0032623	interleukin-2 production	20/3805	0.001156086	0.004599309
GO:0010212	response to ionizing radiation	31/3805	0.001158776	0.004604145
GO:0035303	regulation of dephosphorylation	31/3805	0.001158776	0.004604145
GO:0035330	regulation of hippo signaling	Aug-05	0.001168275	0.004615461
GO:0036035	osteoclast development	Aug-05	0.001168275	0.004615461
GO:0036159	inner dynein arm assembly	Aug-05	0.001168275	0.004615461
GO:0044406	adhesion of symbiont to host	Aug-05	0.001168275	0.004615461
GO:0061307	cardiac neural crest cell differentiation involved in heart development	Aug-05	0.001168275	0.004615461
GO:0062099	negative regulation of programmed necrotic cell death	Aug-05	0.001168275	0.004615461
GO:0070486	leukocyte aggregation	Aug-05	0.001168275	0.004615461
GO:1901881	positive regulation of protein depolymerization	Aug-05	0.001168275	0.004615461
GO:1904424	regulation of GTP binding	Aug-05	0.001168275	0.004615461
GO:0090276	regulation of peptide hormone secretion	48/3805	0.001175147	0.004639676

GO:0017121	plasma membrane phospholipid scrambling	Sep-05	0.001184837	0.004654396
GO:0044851	hair cycle phase	Sep-05	0.001184837	0.004654396
GO:0048486	parasympathetic nervous system development	Sep-05	0.001184837	0.004654396
GO:0051770	positive regulation of nitric-oxide synthase biosynthetic process	Sep-05	0.001184837	0.004654396
GO:0060438	trachea development	Sep-05	0.001184837	0.004654396
GO:0072111	cell proliferation involved in kidney development	Sep-05	0.001184837	0.004654396
GO:0090026	positive regulation of monocyte chemotaxis	Sep-05	0.001184837	0.004654396
GO:2001224	positive regulation of neuron migration	Sep-05	0.001184837	0.004654396
GO:1990573	potassium ion import across plasma membrane	15/3805	0.001188957	0.004667645
GO:0021695	cerebellar cortex development	19/3805	0.001192378	0.004672265
GO:0070613	regulation of protein processing	19/3805	0.001192378	0.004672265
GO:0099601	regulation of neurotransmitter receptor activity	19/3805	0.001192378	0.004672265
GO:0008608	attachment of spindle microtubules to kinetochore	Dec-05	0.001210306	0.004727687
GO:0010667	negative regulation of cardiac muscle cell apoptotic process	Dec-05	0.001210306	0.004727687
GO:0043243	positive regulation of protein-containing complex disassembly	Dec-05	0.001210306	0.004727687
GO:1990089	response to nerve growth factor	Dec-05	0.001210306	0.004727687
GO:1990090	cellular response to nerve growth factor stimulus	Dec-05	0.001210306	0.004727687
GO:0050435	amyloid-beta metabolic process	18/3805	0.001217743	0.004750795

GO:0060393	regulation of pathway-restricted SMAD protein phosphorylation	18/3805	0.001217743	0.004750795
GO:0035825	homologous recombination	16/3805	0.001220569	0.004755881
GO:0045912	negative regulation of carbohydrate metabolic process	16/3805	0.001220569	0.004755881
GO:0050775	positive regulation of dendrite morphogenesis	17/3805	0.001228558	0.004781043
GO:0071622	regulation of granulocyte chemotaxis	17/3805	0.001228558	0.004781043
GO:2000045	regulation of G1/S transition of mitotic cell cycle	33/3805	0.001249513	0.004859563
GO:0030500	regulation of bone mineralization	22/3805	0.001257168	0.004886291
GO:0034219	carbohydrate transmembrane transport	30/3805	0.001261823	0.004901335
GO:0008645	hexose transmembrane transport	28/3805	0.001301899	0.005050721
GO:0031058	positive regulation of histone modification	28/3805	0.001301899	0.005050721
GO:0008643	carbohydrate transport	35/3805	0.001320493	0.005119679
GO:0009880	embryonic pattern specification	21/3805	0.001322875	0.005125731
GO:0009101	glycoprotein biosynthetic process	54/3805	0.00133238	0.005159358
GO:0002676	regulation of chronic inflammatory response	Jun-05	0.001344878	0.005172482
GO:0009415	response to water	Jun-05	0.001344878	0.005172482
GO:0034115	negative regulation of heterotypic cell-cell adhesion	Jun-05	0.001344878	0.005172482
GO:0034454	microtubule anchoring at centrosome	Jun-05	0.001344878	0.005172482
GO:0038180	nerve growth factor signaling pathway	Jun-05	0.001344878	0.005172482

GO:0045602	negative regulation of endothelial cell differentiation	Jun-05	0.001344878	0.005172482
GO:0070587	regulation of cell-cell adhesion involved in gastrulation	Jun-05	0.001344878	0.005172482
GO:0072178	nephric duct morphogenesis	Jun-05	0.001344878	0.005172482
GO:1902513	regulation of organelle transport along microtubule	Jun-05	0.001344878	0.005172482
GO:1903564	regulation of protein localization to cilium	Jun-05	0.001344878	0.005172482
GO:2001204	regulation of osteoclast development	Jun-05	0.001344878	0.005172482
GO:0032232	negative regulation of actin filament bundle assembly	13/3805	0.001349232	0.005179658
GO:0034110	regulation of homotypic cell-cell adhesion	13/3805	0.001349232	0.005179658
GO:0106056	regulation of calcineurin-mediated signaling	13/3805	0.001349232	0.005179658
GO:0010611	regulation of cardiac muscle hypertrophy	20/3805	0.001383718	0.005299019
GO:0016445	somatic diversification of immunoglobulins	20/3805	0.001383718	0.005299019
GO:0048645	animal organ formation	20/3805	0.001383718	0.005299019
GO:0071479	cellular response to ionizing radiation	20/3805	0.001383718	0.005299019
GO:0007097	nuclear migration	Nov-05	0.001395162	0.005320025
GO:0010453	regulation of cell fate commitment	Nov-05	0.001395162	0.005320025
GO:0048535	lymph node development	Nov-05	0.001395162	0.005320025
GO:1903319	positive regulation of protein maturation	Nov-05	0.001395162	0.005320025
GO:2000648	positive regulation of stem cell proliferation	Nov-05	0.001395162	0.005320025

GO:0015914	phospholipid transport	23/3805	0.001395165	0.005320025
GO:0045814	negative regulation of gene expression, epigenetic	23/3805	0.001395165	0.005320025
GO:0002456	T cell mediated immunity	33/3805	0.001410557	0.005372157
GO:1903364	positive regulation of cellular protein catabolic process	33/3805	0.001410557	0.005372157
GO:0036473	cell death in response to oxidative stress	25/3805	0.001420448	0.005406532
GO:0001659	temperature homeostasis	40/3805	0.001421725	0.005408098
GO:0010569	regulation of double-strand break repair via homologous recombination	19/3805	0.001436972	0.005456127
GO:0046324	regulation of glucose import	19/3805	0.001436972	0.005456127
GO:0050885	neuromuscular process controlling balance	19/3805	0.001436972	0.005456127
GO:0009743	response to carbohydrate	44/3805	0.001439575	0.005462688
GO:0002703	regulation of leukocyte mediated immunity	59/3805	0.001452758	0.005509367
GO:0032613	interleukin-10 production	18/3805	0.001479237	0.005596192
GO:0060038	cardiac muscle cell proliferation	18/3805	0.001479237	0.005596192
GO:0060999	positive regulation of dendritic spine development	18/3805	0.001479237	0.005596192
GO:0098930	axonal transport	18/3805	0.001479237	0.005596192
GO:0043525	positive regulation of neuron apoptotic process	22/3805	0.001481966	0.0055965
GO:0002821	positive regulation of adaptive immune response	35/3805	0.001482006	0.0055965
GO:0097530	granulocyte migration	35/3805	0.001482006	0.0055965

GO:0022600	digestive system process	28/3805	0.001491614	0.005629377
GO:0003197	endocardial cushion development	15/3805	0.001493827	0.005630925
GO:0003254	regulation of membrane depolarization	15/3805	0.001493827	0.005630925
GO:0032757	positive regulation of interleukin-8 production	17/3805	0.001506323	0.005674602
GO:0002762	negative regulation of myeloid leukocyte differentiation	16/3805	0.001513171	0.005696965
GO:0033555	multicellular organismal response to stress	26/3805	0.001524005	0.005734298
GO:0031668	cellular response to extracellular stimulus	48/3805	0.001553244	0.005840795
GO:0022406	membrane docking	21/3805	0.001567148	0.005882454
GO:0032088	negative regulation of NF-kappaB transcription factor activity	21/3805	0.001567148	0.005882454
GO:0050709	negative regulation of protein secretion	21/3805	0.001567148	0.005882454
GO:0003071	renal system process involved in regulation of systemic arterial blood pressure	Oct-05	0.001574223	0.005894842
GO:0032528	microvillus organization	Oct-05	0.001574223	0.005894842
GO:0034453	microtubule anchoring	Oct-05	0.001574223	0.005894842
GO:0035809	regulation of urine volume	Oct-05	0.001574223	0.005894842
GO:0040001	establishment of mitotic spindle localization	Dec-05	0.001599688	0.005983024
GO:1904037	positive regulation of epithelial cell apoptotic process	Dec-05	0.001599688	0.005983024
GO:0010466	negative regulation of peptidase activity	52/3805	0.001625362	0.006071776
GO:2001020	regulation of response to DNA damage stimulus	52/3805	0.001625362	0.006071776

GO:0061515	myeloid cell development	23/3805	0.001631555	0.00609127
GO:0021510	spinal cord development	25/3805	0.00164441	0.006128273
GO:0021761	limbic system development	25/3805	0.00164441	0.006128273
GO:0045132	meiotic chromosome segregation	25/3805	0.00164441	0.006128273
GO:0000725	recombinational repair	35/3805	0.001660421	0.006184253
GO:0003091	renal water homeostasis	Jul-05	0.001695225	0.006280177
GO:0030320	cellular monovalent inorganic anion homeostasis	Jul-05	0.001695225	0.006280177
GO:0032530	regulation of microvillus organization	Jul-05	0.001695225	0.006280177
GO:0034350	regulation of glial cell apoptotic process	Jul-05	0.001695225	0.006280177
GO:0061577	calcium ion transmembrane transport via high voltage-gated calcium channel	Jul-05	0.001695225	0.006280177
GO:0070307	lens fiber cell development	Jul-05	0.001695225	0.006280177
GO:0086014	atrial cardiac muscle cell action potential	Jul-05	0.001695225	0.006280177
GO:0086026	atrial cardiac muscle cell to AV node cell signaling	Jul-05	0.001695225	0.006280177
GO:0086066	atrial cardiac muscle cell to AV node cell communication	Jul-05	0.001695225	0.006280177
GO:0008637	apoptotic mitochondrial changes	28/3805	0.001704706	0.006311555
GO:0002115	store-operated calcium entry	Sep-05	0.001720163	0.006341203
GO:0010232	vascular transport	Sep-05	0.001720163	0.006341203
GO:0021535	cell migration in hindbrain	Sep-05	0.001720163	0.006341203

GO:0033630	positive regulation of cell adhesion mediated by integrin	Sep-05	0.001720163	0.006341203
GO:0072202	cell differentiation involved in metanephros development	Sep-05	0.001720163	0.006341203
GO:1902894	negative regulation of miRNA transcription	Sep-05	0.001720163	0.006341203
GO:0002763	positive regulation of myeloid leukocyte differentiation	19/3805	0.001722866	0.006341203
GO:0017156	calcium-ion regulated exocytosis	19/3805	0.001722866	0.006341203
GO:0042058	regulation of epidermal growth factor receptor signaling pathway	19/3805	0.001722866	0.006341203
GO:2000134	negative regulation of G1/S transition of mitotic cell cycle	19/3805	0.001722866	0.006341203
GO:0003230	cardiac atrium development	13/3805	0.001739727	0.006391962
GO:0007431	salivary gland development	13/3805	0.001739727	0.006391962
GO:0030513	positive regulation of BMP signaling pathway	13/3805	0.001739727	0.006391962
GO:0033044	regulation of chromosome organization	43/3805	0.001751986	0.006433219
GO:1903052	positive regulation of proteolysis involved in cellular protein catabolic process	29/3805	0.001781771	0.006530269
GO:0021783	preganglionic parasympathetic fiber development	Aug-05	0.001785735	0.006530269
GO:0032570	response to progesterone	Aug-05	0.001785735	0.006530269
GO:0033623	regulation of integrin activation	Aug-05	0.001785735	0.006530269
GO:0035313	wound healing, spreading of epidermal cells	Aug-05	0.001785735	0.006530269
GO:0086011	membrane repolarization during action potential	Aug-05	0.001785735	0.006530269
GO:0090660	cerebrospinal fluid circulation	Aug-05	0.001785735	0.006530269

GO:0030279	negative regulation of ossification	14/3805	0.001823707	0.006661328
GO:1903170	negative regulation of calcium ion transmembrane transport	14/3805	0.001823707	0.006661328
GO:0033003	regulation of mast cell activation	17/3805	0.001835194	0.006691547
GO:0042551	neuron maturation	17/3805	0.001835194	0.006691547
GO:0060324	face development	17/3805	0.001835194	0.006691547
GO:0050670	regulation of lymphocyte proliferation	49/3805	0.001840784	0.006708013
GO:0014855	striated muscle cell proliferation	21/3805	0.001848792	0.006725427
GO:0051279	regulation of release of sequestered calcium ion into cytosol	21/3805	0.001848792	0.006725427
GO:0051303	establishment of chromosome localization	21/3805	0.001848792	0.006725427
GO:0071322	cellular response to carbohydrate stimulus	35/3805	0.001857172	0.006751976
GO:0002791	regulation of peptide secretion	48/3805	0.00186101	0.006759511
GO:0006687	glycosphingolipid metabolic process	15/3805	0.001861466	0.006759511
GO:0071470	cellular response to osmotic stress	16/3805	0.001862491	0.006759511
GO:0000132	establishment of mitotic spindle orientation	Nov-05	0.001874976	0.006773336
GO:0003416	endochondral bone growth	Nov-05	0.001874976	0.006773336
GO:0021904	dorsal/ventral neural tube patterning	Nov-05	0.001874976	0.006773336
GO:0045830	positive regulation of isotype switching	Nov-05	0.001874976	0.006773336
GO:0055022	negative regulation of cardiac muscle tissue growth	Nov-05	0.001874976	0.006773336

GO:0060390	regulation of SMAD protein signal transduction	Nov-05	0.001874976	0.006773336
GO:0061117	negative regulation of heart growth	Nov-05	0.001874976	0.006773336
GO:0098703	calcium ion import across plasma membrane	Nov-05	0.001874976	0.006773336
GO:0016573	histone acetylation	36/3805	0.001883008	0.006798421
GO:1905039	carboxylic acid transmembrane transport	31/3805	0.001910183	0.006892551
GO:0045861	negative regulation of proteolysis	67/3805	0.001917983	0.006916698
GO:1903305	regulation of regulated secretory pathway	39/3805	0.001928815	0.006951746
GO:0015749	monosaccharide transmembrane transport	28/3805	0.001943471	0.007000529
GO:0046942	carboxylic acid transport	56/3805	0.001954421	0.007035915
GO:0006665	sphingolipid metabolic process	32/3805	0.001962147	0.007059662
GO:2001251	negative regulation of chromosome organization	22/3805	0.002035829	0.007320545
GO:0015849	organic acid transport	61/3805	0.002057465	0.00739409
GO:0006884	cell volume homeostasis	Dec-05	0.002086495	0.007485495
GO:0007435	salivary gland morphogenesis	Dec-05	0.002086495	0.007485495
GO:0090075	relaxation of muscle	Dec-05	0.002086495	0.007485495
GO:0031497	chromatin assembly	36/3805	0.002097673	0.007516961
GO:0055067	monovalent inorganic cation homeostasis	36/3805	0.002097673	0.007516961
GO:0006643	membrane lipid metabolic process	39/3805	0.002135505	0.007648143

GO:0002052	positive regulation of neuroblast proliferation	Oct-05	0.00216193	0.007711831
GO:0007176	regulation of epidermal growth factor-activated receptor activity	Oct-05	0.00216193	0.007711831
GO:0033622	integrin activation	Oct-05	0.00216193	0.007711831
GO:0033688	regulation of osteoblast proliferation	Oct-05	0.00216193	0.007711831
GO:0038083	peptidyl-tyrosine autophosphorylation	Oct-05	0.00216193	0.007711831
GO:0051767	nitric-oxide synthase biosynthetic process	Oct-05	0.00216193	0.007711831
GO:0051769	regulation of nitric-oxide synthase biosynthetic process	Oct-05	0.00216193	0.007711831
GO:0046928	regulation of neurotransmitter secretion	28/3805	0.002210359	0.007880083
GO:0071174	mitotic spindle checkpoint signaling	13/3805	0.002219048	0.007897533
GO:0098751	bone cell development	13/3805	0.002219048	0.007897533
GO:1900181	negative regulation of protein localization to nucleus	13/3805	0.002219048	0.007897533
GO:0031348	negative regulation of defense response	48/3805	0.002220726	0.007899006
GO:0002067	glandular epithelial cell differentiation	17/3805	0.002222266	0.007899984
GO:0051353	positive regulation of oxidoreductase activity	16/3805	0.002276748	0.008079869
GO:0051489	regulation of filopodium assembly	16/3805	0.002276748	0.008079869
GO:0140353	lipid export from cell	16/3805	0.002276748	0.008079869
GO:0010543	regulation of platelet activation	14/3805	0.002286206	0.008108825
GO:0060043	regulation of cardiac muscle cell proliferation	15/3805	0.002301439	0.008153594

GO:006 1756	leukocyte adhesion to vascular endothelial cell	15/38 05	0.00230 1439	0.00815 3594
GO:007 1482	cellular response to light stimulus	26/38 05	0.00230 5365	0.00816 2875
GO:001 4743	regulation of muscle hypertrophy	20/38 05	0.00230 8162	0.00816 3521
GO:004 5453	bone resorption	20/38 05	0.00230 8162	0.00816 3521
GO:001 0951	negative regulation of endopeptidase activity	39/38 05	0.00236 1092	0.00834 6
GO:004 6323	glucose import	22/38 05	0.00237 2953	0.00837 6733
GO:000 2246	wound healing involved in inflammatory response	Jun-05	0.00238 9904	0.00837 6733
GO:000 7183	SMAD protein complex assembly	Jun-05	0.00238 9904	0.00837 6733
GO:001 6188	synaptic vesicle maturation	Jun-05	0.00238 9904	0.00837 6733
GO:002 1561	facial nerve development	Jun-05	0.00238 9904	0.00837 6733
GO:002 1610	facial nerve morphogenesis	Jun-05	0.00238 9904	0.00837 6733
GO:003 3275	actin-myosin filament sliding	Jun-05	0.00238 9904	0.00837 6733
GO:003 5331	negative regulation of hippo signaling	Jun-05	0.00238 9904	0.00837 6733
GO:003 6005	response to macrophage colony-stimulating factor	Jun-05	0.00238 9904	0.00837 6733
GO:003 6006	cellular response to macrophage colony-stimulating factor stimulus	Jun-05	0.00238 9904	0.00837 6733
GO:006 0430	lung saccule development	Jun-05	0.00238 9904	0.00837 6733
GO:007 0316	regulation of G0 to G1 transition	Jun-05	0.00238 9904	0.00837 6733
GO:007 0586	cell-cell adhesion involved in gastrulation	Jun-05	0.00238 9904	0.00837 6733

GO:0090399	replicative senescence	Jun-05	0.002389904	0.008376733
GO:1903960	negative regulation of anion transmembrane transport	Jun-05	0.002389904	0.008376733
GO:0090087	regulation of peptide transport	48/3805	0.002422245	0.008485329
GO:0002693	positive regulation of cellular extravasation	Sep-05	0.002432772	0.008493623
GO:0030903	notochord development	Sep-05	0.002432772	0.008493623
GO:0032515	negative regulation of phosphoprotein phosphatase activity	Sep-05	0.002432772	0.008493623
GO:0048485	sympathetic nervous system development	Sep-05	0.002432772	0.008493623
GO:1902547	regulation of cellular response to vascular endothelial growth factor stimulus	Sep-05	0.002432772	0.008493623
GO:1990776	response to angiotensin	Sep-05	0.002432772	0.008493623
GO:0060761	negative regulation of response to cytokine stimulus	19/3805	0.002440394	0.008515475
GO:0120161	regulation of cold-induced thermogenesis	32/3805	0.002473156	0.008624976
GO:0030224	monocyte differentiation	Nov-05	0.002480889	0.008632665
GO:0044030	regulation of DNA methylation	Nov-05	0.002480889	0.008632665
GO:0060669	embryonic placenta morphogenesis	Nov-05	0.002480889	0.008632665
GO:1902656	calcium ion import into cytosol	Nov-05	0.002480889	0.008632665
GO:0002824	positive regulation of adaptive immune response based on somatic recombination of immune receptors built from immunoglobulin superfamily domains	33/3805	0.002514728	0.008745542
GO:0031507	heterochromatin assembly	21/3805	0.00254223	0.008821539
GO:0043154	negative regulation of cysteine-type endopeptidase activity involved in apoptotic process	21/3805	0.00254223	0.008821539

GO:0048708	astrocyte differentiation	21/3805	0.00254223	0.008821539
GO:2001259	positive regulation of cation channel activity	21/3805	0.00254223	0.008821539
GO:0000724	double-strand break repair via homologous recombination	34/3805	0.002547837	0.008836086
GO:2000278	regulation of DNA biosynthetic process	29/3805	0.002590318	0.008978428
GO:0002027	regulation of heart rate	26/3805	0.002632172	0.009096112
GO:0002544	chronic inflammatory response	Aug-05	0.002634465	0.009096112
GO:0003215	cardiac right ventricle morphogenesis	Aug-05	0.002634465	0.009096112
GO:0010766	negative regulation of sodium ion transport	Aug-05	0.002634465	0.009096112
GO:0036120	cellular response to platelet-derived growth factor stimulus	Aug-05	0.002634465	0.009096112
GO:0072087	renal vesicle development	Aug-05	0.002634465	0.009096112
GO:2001135	regulation of endocytic recycling	Aug-05	0.002634465	0.009096112
GO:0002467	germinal center formation	Jul-05	0.002672198	0.009175673
GO:0019184	nonribosomal peptide biosynthetic process	Jul-05	0.002672198	0.009175673
GO:0042048	olfactory behavior	Jul-05	0.002672198	0.009175673
GO:0072673	lamellipodium morphogenesis	Jul-05	0.002672198	0.009175673
GO:0086103	G protein-coupled receptor signaling pathway involved in heart process	Jul-05	0.002672198	0.009175673
GO:1901722	regulation of cell proliferation involved in kidney development	Jul-05	0.002672198	0.009175673
GO:1902043	positive regulation of extrinsic apoptotic signaling pathway via death domain receptors	Jul-05	0.002672198	0.009175673

GO:1902287	semaphorin-plexin signaling pathway involved in axon guidance	Jul-05	0.002672198	0.009175673
GO:2001212	regulation of vasculogenesis	Jul-05	0.002672198	0.009175673
GO:2001256	regulation of store-operated calcium entry	Jul-05	0.002672198	0.009175673
GO:0032655	regulation of interleukin-12 production	17/3805	0.002675238	0.009181065
GO:0010664	negative regulation of striated muscle cell apoptotic process	Dec-05	0.002687948	0.009194371
GO:0010737	protein kinase A signaling	Dec-05	0.002687948	0.009194371
GO:0046329	negative regulation of JNK cascade	Dec-05	0.002687948	0.009194371
GO:0070528	protein kinase C signaling	Dec-05	0.002687948	0.009194371
GO:0097106	postsynaptic density organization	Dec-05	0.002687948	0.009194371
GO:0098868	bone growth	Dec-05	0.002687948	0.009194371
GO:0021587	cerebellum morphogenesis	16/3805	0.002764879	0.009442009
GO:0042311	vasodilation	16/3805	0.002764879	0.009442009
GO:0043030	regulation of macrophage activation	16/3805	0.002764879	0.009442009
GO:0098801	regulation of renal system process	13/3805	0.002801655	0.009557149
GO:1902116	negative regulation of organelle assembly	13/3805	0.002801655	0.009557149
GO:0046189	phenol-containing compound biosynthetic process	15/3805	0.002824172	0.0096287
GO:0034205	amyloid-beta formation	14/3805	0.002841289	0.009676495
GO:0048246	macrophage chemotaxis	14/3805	0.002841289	0.009676495

GO:0003300	cardiac muscle hypertrophy	25/3805	0.002868013	0.009762185
GO:0006475	internal protein amino acid acetylation	37/3805	0.002879856	0.009791823
GO:0018393	internal peptidyl-lysine acetylation	37/3805	0.002879856	0.009791823
GO:0030104	water homeostasis	19/3805	0.002884064	0.009800795
GO:0010954	positive regulation of protein processing	Oct-05	0.002913843	0.009885858
GO:0033081	regulation of T cell differentiation in thymus	Oct-05	0.002913843	0.009885858
GO:0090330	regulation of platelet aggregation	Oct-05	0.002913843	0.009885858
GO:0002698	negative regulation of immune effector process	29/3805	0.002921554	0.009906639

GO terms associated with 5864 Rep_late_enriched_vs_Hep genes

ID	Description	Gene Ratio	pvalue	p.adjust
GO:0044782	cilium organization	193/4924	4.80E-50	3.02E-46
GO:0007015	actin filament organization	209/4924	1.78E-46	5.63E-43
GO:0060271	cilium assembly	175/4924	8.91E-45	1.87E-41
GO:0032970	regulation of actin filament-based process	184/4924	4.42E-39	6.96E-36
GO:1902903	regulation of supramolecular fiber organization	175/4924	2.99E-38	3.77E-35
GO:0007264	small GTPase mediated signal transduction	186/4924	2.35E-37	2.47E-34
GO:0034329	cell junction assembly	187/4924	2.70E-36	2.43E-33
GO:0007018	microtubule-based movement	182/4924	7.14E-36	5.63E-33
GO:0032956	regulation of actin cytoskeleton organization	162/4924	2.78E-34	1.94E-31
GO:0060562	epithelial tube morphogenesis	169/4924	1.74E-33	1.10E-30
GO:0050808	synapse organization	196/4924	3.25E-33	1.86E-30
GO:0007409	axonogenesis	192/4924	2.62E-31	1.38E-28
GO:0030198	extracellular matrix organization	142/4924	3.07E-31	1.49E-28
GO:0043062	extracellular structure organization	142/4924	4.65E-31	2.09E-28
GO:0045229	external encapsulating structure organization	142/4924	7.02E-31	2.95E-28
GO:0001667	ameboidal-type cell migration	178/4924	8.43E-30	3.32E-27

GO:0031346	positive regulation of cell projection organization	175/4924	1.64E-29	6.10E-27
GO:0010639	negative regulation of organelle organization	155/4924	4.08E-29	1.43E-26
GO:0007265	Ras protein signal transduction	145/4924	2.07E-28	6.88E-26
GO:0031589	cell-substrate adhesion	153/4924	5.02E-28	1.58E-25
GO:0000280	nuclear division	172/4924	1.01E-27	3.04E-25
GO:0045216	cell-cell junction organization	106/4924	2.01E-27	5.77E-25
GO:0050767	regulation of neurogenesis	174/4924	2.15E-27	5.90E-25
GO:0110053	regulation of actin filament organization	123/4924	1.12E-26	2.94E-24
GO:0061572	actin filament bundle organization	92/4924	1.38E-26	3.48E-24
GO:0001655	urogenital system development	154/4924	1.61E-26	3.91E-24
GO:0007178	transmembrane receptor protein serine/threonine kinase signaling pathway	154/4924	5.83E-26	1.36E-23
GO:0051017	actin filament bundle assembly	90/4924	7.66E-26	1.72E-23
GO:0140014	mitotic nuclear division	123/4924	8.24E-26	1.79E-23
GO:0072001	renal system development	141/4924	9.60E-26	2.02E-23
GO:0001578	microtubule bundle formation	73/4924	1.25E-25	2.55E-23
GO:0043254	regulation of protein-containing complex assembly	163/4924	2.20E-25	4.33E-23
GO:0001822	kidney development	136/4924	2.71E-25	5.17E-23
GO:0032535	regulation of cellular component size	153/4924	3.51E-25	6.51E-23

GO:0032886	regulation of microtubule-based process	118/4924	4.34E-25	7.83E-23
GO:0045785	positive regulation of cell adhesion	176/4924	5.40E-25	9.46E-23
GO:0048762	mesenchymal cell differentiation	110/4924	7.09E-25	1.21E-22
GO:0099111	microtubule-based transport	100/4924	7.27E-25	1.21E-22
GO:0031032	actomyosin structure organization	101/4924	1.03E-24	1.66E-22
GO:0060485	mesenchyme development	126/4924	1.41E-24	2.22E-22
GO:0051258	protein polymerization	119/4924	2.54E-24	3.90E-22
GO:0043087	regulation of GTPase activity	137/4924	1.22E-23	1.84E-21
GO:0048285	organelle fission	177/4924	1.32E-23	1.93E-21
GO:0051056	regulation of small GTPase mediated signal transduction	105/4924	1.91E-23	2.73E-21
GO:0040013	negative regulation of locomotion	135/4924	2.62E-23	3.67E-21
GO:0007346	regulation of mitotic cell cycle	171/4924	4.36E-23	5.98E-21
GO:0051494	negative regulation of cytoskeleton organization	87/4924	4.86E-23	6.51E-21
GO:0016358	dendrite development	128/4924	6.15E-23	7.91E-21
GO:0090130	tissue migration	128/4924	6.15E-23	7.91E-21
GO:0090287	regulation of cellular response to growth factor stimulus	123/4924	8.90E-23	1.12E-20
GO:0033674	positive regulation of kinase activity	159/4924	9.32E-23	1.15E-20
GO:0090132	epithelium migration	127/4924	1.06E-22	1.29E-20

GO:0022604	regulation of cell morphogenesis	133/4924	1.46E-22	1.74E-20
GO:0007162	negative regulation of cell adhesion	126/4924	1.83E-22	2.09E-20
GO:0010631	epithelial cell migration	126/4924	1.83E-22	2.09E-20
GO:0001558	regulation of cell growth	165/4924	1.86E-22	2.09E-20
GO:1902904	negative regulation of supramolecular fiber organization	86/4924	6.07E-22	6.72E-20
GO:0007163	establishment or maintenance of cell polarity	101/4924	9.53E-22	1.04E-19
GO:0032271	regulation of protein polymerization	95/4924	1.11E-21	1.19E-19
GO:0198738	cell-cell signaling by wnt	161/4924	1.38E-21	1.45E-19
GO:0016055	Wnt signaling pathway	160/4924	2.26E-21	2.34E-19
GO:0060560	developmental growth involved in morphogenesis	115/4924	2.60E-21	2.63E-19
GO:0001763	morphogenesis of a branching structure	106/4924	2.63E-21	2.63E-19
GO:0060541	respiratory system development	111/4924	2.93E-21	2.89E-19
GO:0022407	regulation of cell-cell adhesion	164/4924	3.03E-21	2.94E-19
GO:0071560	cellular response to transforming growth factor beta stimulus	99/4924	4.41E-21	4.21E-19
GO:0071559	response to transforming growth factor beta	100/4924	5.45E-21	5.13E-19
GO:0045860	positive regulation of protein kinase activity	135/4924	6.50E-21	6.03E-19
GO:0044772	mitotic cell cycle phase transition	151/4924	7.03E-21	6.42E-19
GO:0097485	neuron projection guidance	108/4924	7.61E-21	6.86E-19

GO:0051271	negative regulation of cellular component movement	123/4924	8.90E-21	7.90E-19
GO:0001654	eye development	151/4924	9.14E-21	8.00E-19
GO:0001837	epithelial to mesenchymal transition	77/4924	9.99E-21	8.63E-19
GO:0007224	smoothened signaling pathway	78/4924	1.50E-20	1.28E-18
GO:0007411	axon guidance	107/4924	1.89E-20	1.59E-18
GO:0150063	visual system development	151/4924	1.99E-20	1.65E-18
GO:0048880	sensory system development	152/4924	2.02E-20	1.66E-18
GO:0051090	regulation of DNA-binding transcription factor activity	154/4924	2.07E-20	1.68E-18
GO:0070507	regulation of microtubule cytoskeleton organization	78/4924	3.95E-20	3.15E-18
GO:0030010	establishment of cell polarity	75/4924	8.61E-20	6.79E-18
GO:0071900	regulation of protein serine/threonine kinase activity	131/4924	9.21E-20	7.17E-18
GO:0090092	regulation of transmembrane receptor protein serine/threonine kinase signaling pathway	105/4924	1.14E-19	8.79E-18
GO:2000146	negative regulation of cell motility	118/4924	1.75E-19	1.33E-17
GO:0007389	pattern specification process	165/4924	2.20E-19	1.65E-17
GO:0030900	forebrain development	142/4924	2.25E-19	1.67E-17
GO:0061138	morphogenesis of a branching epithelium	97/4924	2.80E-19	2.05E-17
GO:0030336	negative regulation of cell migration	114/4924	3.14E-19	2.28E-17
GO:0008154	actin polymerization or depolymerization	87/4924	8.29E-19	5.86E-17

GO:0030705	cytoskeleton-dependent intracellular transport	87/4924	8.29E-19	5.86E-17
GO:0003007	heart morphogenesis	111/4924	8.36E-19	5.86E-17
GO:0048588	developmental cell growth	109/4924	9.50E-19	6.58E-17
GO:0034765	regulation of ion transmembrane transport	165/4924	1.32E-18	8.95E-17
GO:1901987	regulation of cell cycle phase transition	141/4924	1.32E-18	8.95E-17
GO:0045787	positive regulation of cell cycle	129/4924	1.76E-18	1.18E-16
GO:0007059	chromosome segregation	125/4924	2.01E-18	1.33E-16
GO:0048608	reproductive structure development	161/4924	2.26E-18	1.48E-16
GO:0031109	microtubule polymerization or depolymerization	65/4924	2.33E-18	1.52E-16
GO:0046578	regulation of Ras protein signal transduction	87/4924	2.66E-18	1.70E-16
GO:0031345	negative regulation of cell projection organization	91/4924	2.67E-18	1.70E-16
GO:0051495	positive regulation of cytoskeleton organization	88/4924	3.24E-18	2.04E-16
GO:0007160	cell-matrix adhesion	96/4924	4.08E-18	2.54E-16
GO:0042692	muscle cell differentiation	149/4924	4.36E-18	2.70E-16
GO:0007043	cell-cell junction assembly	73/4924	4.62E-18	2.83E-16
GO:0061458	reproductive system development	161/4924	5.44E-18	3.30E-16
GO:0007179	transforming growth factor beta receptor signaling pathway	82/4924	6.42E-18	3.86E-16
GO:0030323	respiratory tube development	97/4924	6.55E-18	3.89E-16

GO:0035082	axoneme assembly	52/4924	6.96E-18	4.10E-16
GO:0030324	lung development	96/4924	8.07E-18	4.71E-16
GO:0050678	regulation of epithelial cell proliferation	140/4924	9.41E-18	5.44E-16
GO:0042063	gliogenesis	126/4924	1.04E-17	5.99E-16
GO:0010970	transport along microtubule	76/4924	1.13E-17	6.40E-16
GO:0051261	protein depolymerization	63/4924	1.26E-17	7.07E-16
GO:0010632	regulation of epithelial cell migration	97/4924	1.28E-17	7.13E-16
GO:0007051	spindle organization	81/4924	1.68E-17	9.30E-16
GO:0010721	negative regulation of cell development	87/4924	1.73E-17	9.46E-16
GO:0045926	negative regulation of growth	103/4924	2.35E-17	1.28E-15
GO:0010959	regulation of metal ion transport	149/4924	2.71E-17	1.46E-15
GO:1901342	regulation of vasculature development	115/4924	2.79E-17	1.48E-15
GO:1901990	regulation of mitotic cell cycle phase transition	115/4924	2.79E-17	1.48E-15
GO:1902905	positive regulation of supramolecular fiber organization	80/4924	2.96E-17	1.56E-15
GO:1990778	protein localization to cell periphery	130/4924	3.11E-17	1.62E-15
GO:0043010	camera-type eye development	131/4924	3.13E-17	1.62E-15
GO:0048732	gland development	156/4924	3.53E-17	1.81E-15
GO:0060491	regulation of cell projection assembly	87/4924	3.56E-17	1.81E-15

GO:0090068	positive regulation of cell cycle process	103/4924	4.33E-17	2.19E-15
GO:0051961	negative regulation of nervous system development	75/4924	4.60E-17	2.30E-15
GO:0010810	regulation of cell-substrate adhesion	92/4924	5.13E-17	2.55E-15
GO:0061351	neural precursor cell proliferation	84/4924	6.39E-17	3.15E-15
GO:0030111	regulation of Wnt signaling pathway	116/4924	6.61E-17	3.23E-15
GO:0061448	connective tissue development	110/4924	6.78E-17	3.29E-15
GO:1902850	microtubule cytoskeleton organization involved in mitosis	69/4924	7.30E-17	3.51E-15
GO:0042060	wound healing	130/4924	8.36E-17	3.99E-15
GO:0050673	epithelial cell proliferation	155/4924	8.54E-17	4.05E-15
GO:0010976	positive regulation of neuron projection development	91/4924	8.85E-17	4.17E-15
GO:0098813	nuclear chromosome segregation	105/4924	9.08E-17	4.24E-15
GO:0000819	sister chromatid segregation	81/4924	1.13E-16	5.25E-15
GO:1903829	positive regulation of protein localization	158/4924	1.44E-16	6.59E-15
GO:0050768	negative regulation of neurogenesis	73/4924	1.44E-16	6.59E-15
GO:0031023	microtubule organizing center organization	67/4924	1.49E-16	6.74E-15
GO:0051098	regulation of binding	135/4924	1.67E-16	7.52E-15
GO:0140694	non-membrane-bounded organelle assembly	131/4924	1.72E-16	7.70E-15
GO:1901888	regulation of cell junction assembly	89/4924	1.89E-16	8.37E-15

GO:0007611	learning or memory	116/4924	1.93E-16	8.49E-15
GO:0008360	regulation of cell shape	71/4924	1.98E-16	8.68E-15
GO:0010720	positive regulation of cell development	130/4924	2.19E-16	9.54E-15
GO:0045765	regulation of angiogenesis	112/4924	2.23E-16	9.65E-15
GO:0000070	mitotic sister chromatid segregation	72/4924	2.55E-16	1.10E-14
GO:0044839	cell cycle G2/M phase transition	69/4924	2.69E-16	1.14E-14
GO:0051962	positive regulation of nervous system development	125/4924	2.80E-16	1.19E-14
GO:0060070	canonical Wnt signaling pathway	111/4924	2.83E-16	1.19E-14
GO:0098742	cell-cell adhesion via plasma-membrane adhesion molecules	87/4924	2.87E-16	1.19E-14
GO:0042073	intraciliary transport	34/4924	2.88E-16	1.19E-14
GO:0035148	tube formation	78/4924	2.89E-16	1.19E-14
GO:0030308	negative regulation of cell growth	84/4924	3.78E-16	1.55E-14
GO:0050807	regulation of synapse organization	100/4924	3.83E-16	1.55E-14
GO:0030038	contractile actin filament bundle assembly	58/4924	3.85E-16	1.55E-14
GO:0043149	stress fiber assembly	58/4924	3.85E-16	1.55E-14
GO:0030041	actin filament polymerization	74/4924	4.05E-16	1.62E-14
GO:0009799	specification of symmetry	66/4924	4.13E-16	1.64E-14
GO:0050803	regulation of synapse structure or activity	102/4924	4.34E-16	1.71E-14

GO:0010717	regulation of epithelial to mesenchymal transition	51/4924	5.07E-16	1.97E-14
GO:1901879	regulation of protein depolymerization	51/4924	5.07E-16	1.97E-14
GO:0120032	regulation of plasma membrane bounded cell projection assembly	84/4924	5.34E-16	2.07E-14
GO:0051091	positive regulation of DNA-binding transcription factor activity	102/4924	5.79E-16	2.22E-14
GO:0050890	cognition	124/4924	7.41E-16	2.83E-14
GO:0048754	branching morphogenesis of an epithelial tube	80/4924	8.47E-16	3.22E-14
GO:0016331	morphogenesis of embryonic epithelium	78/4924	8.75E-16	3.29E-14
GO:0050770	regulation of axonogenesis	78/4924	8.75E-16	3.29E-14
GO:0031110	regulation of microtubule polymerization or depolymerization	51/4924	9.03E-16	3.37E-14
GO:0018209	peptidyl-serine modification	125/4924	9.38E-16	3.48E-14
GO:0030832	regulation of actin filament length	73/4924	1.05E-15	3.88E-14
GO:0009855	determination of bilateral symmetry	65/4924	1.14E-15	4.16E-14
GO:0032231	regulation of actin filament bundle assembly	57/4924	1.14E-15	4.16E-14
GO:0070371	ERK1 and ERK2 cascade	121/4924	1.20E-15	4.36E-14
GO:0008064	regulation of actin polymerization or depolymerization	72/4924	1.26E-15	4.53E-14
GO:0007368	determination of left/right symmetry	62/4924	1.71E-15	6.13E-14
GO:0030177	positive regulation of Wnt signaling pathway	64/4924	2.02E-15	7.20E-14
GO:0001838	embryonic epithelial tube formation	69/4924	2.11E-15	7.49E-14

GO:0002064	epithelial cell development	91/4924	2.18E-15	7.67E-14
GO:0000086	G2/M transition of mitotic cell cycle	63/4924	2.33E-15	8.16E-14
GO:0018108	peptidyl-tyrosine phosphorylation	114/4924	2.43E-15	8.47E-14
GO:0046777	protein autophosphorylation	93/4924	2.54E-15	8.80E-14
GO:0001894	tissue homeostasis	104/4924	3.34E-15	1.15E-13
GO:0001764	neuron migration	80/4924	3.41E-15	1.16E-13
GO:0051656	establishment of organelle localization	139/4924	3.41E-15	1.16E-13
GO:0010563	negative regulation of phosphorus metabolic process	146/4924	3.99E-15	1.35E-13
GO:0045936	negative regulation of phosphate metabolic process	146/4924	3.99E-15	1.35E-13
GO:1990138	neuron projection extension	82/4924	4.38E-15	1.47E-13
GO:0007052	mitotic spindle organization	58/4924	4.40E-15	1.47E-13
GO:0018212	peptidyl-tyrosine modification	114/4924	5.12E-15	1.70E-13
GO:0018105	peptidyl-serine phosphorylation	117/4924	5.14E-15	1.70E-13
GO:0007266	Rho protein signal transduction	63/4924	5.52E-15	1.81E-13
GO:0001503	ossification	137/4924	5.54E-15	1.81E-13
GO:0045786	negative regulation of cell cycle	125/4924	6.01E-15	1.95E-13
GO:2001233	regulation of apoptotic signaling pathway	133/4924	7.75E-15	2.51E-13
GO:0043542	endothelial cell migration	88/4924	7.86E-15	2.53E-13

GO:0003341	cilium movement	85/4924	8.27E-15	2.65E-13
GO:0007098	centrosome cycle	60/4924	8.48E-15	2.70E-13
GO:0072175	epithelial tube formation	71/4924	1.01E-14	3.19E-13
GO:0070372	regulation of ERK1 and ERK2 cascade	113/4924	1.06E-14	3.35E-13
GO:0071902	positive regulation of protein serine/threonine kinase activity	82/4924	1.16E-14	3.65E-13
GO:0031503	protein-containing complex localization	81/4924	1.44E-14	4.51E-13
GO:0060249	anatomical structure homeostasis	113/4924	1.72E-14	5.35E-13
GO:0048638	regulation of developmental growth	131/4924	1.93E-14	5.97E-13
GO:0021915	neural tube development	79/4924	2.22E-14	6.84E-13
GO:0050769	positive regulation of neurogenesis	105/4924	2.73E-14	8.35E-13
GO:0072659	protein localization to plasma membrane	106/4924	2.75E-14	8.39E-13
GO:0060537	muscle tissue development	152/4924	3.08E-14	9.33E-13
GO:0051403	stress-activated MAPK cascade	92/4924	3.25E-14	9.79E-13
GO:0014020	primary neural tube formation	56/4924	3.51E-14	1.05E-12
GO:0043547	positive regulation of GTPase activity	94/4924	3.54E-14	1.06E-12
GO:0043393	regulation of protein binding	84/4924	3.55E-14	1.06E-12
GO:0001952	regulation of cell-matrix adhesion	59/4924	3.59E-14	1.06E-12
GO:0030833	regulation of actin filament polymerization	64/4924	3.62E-14	1.07E-12

GO:0003205	cardiac chamber development	79/4924	4.24E-14	1.24E-12
GO:0043405	regulation of MAP kinase activity	79/4924	4.24E-14	1.24E-12
GO:0045930	negative regulation of mitotic cell cycle	87/4924	4.38E-14	1.27E-12
GO:1903706	regulation of hemopoiesis	130/4924	4.62E-14	1.34E-12
GO:0001738	morphogenesis of a polarized epithelium	46/4924	5.06E-14	1.46E-12
GO:0048562	embryonic organ morphogenesis	113/4924	5.59E-14	1.60E-12
GO:0031098	stress-activated protein kinase signaling cascade	94/4924	6.10E-14	1.74E-12
GO:1903844	regulation of cellular response to transforming growth factor beta stimulus	57/4924	7.40E-14	2.10E-12
GO:0051216	cartilage development	82/4924	7.54E-14	2.13E-12
GO:0003012	muscle system process	136/4924	8.19E-14	2.30E-12
GO:0017015	regulation of transforming growth factor beta receptor signaling pathway	56/4924	8.53E-14	2.39E-12
GO:0050900	leukocyte migration	124/4924	8.56E-14	2.39E-12
GO:0042391	regulation of membrane potential	145/4924	9.42E-14	2.62E-12
GO:0031333	negative regulation of protein-containing complex assembly	65/4924	9.52E-14	2.62E-12
GO:0072073	kidney epithelium development	65/4924	9.52E-14	2.62E-12
GO:0010634	positive regulation of epithelial cell migration	66/4924	1.14E-13	3.13E-12
GO:0001843	neural tube closure	53/4924	1.27E-13	3.47E-12
GO:0006260	DNA replication	98/4924	1.47E-13	3.98E-12

GO:0030048	actin filament-based movement	58/4924	1.48E-13	3.99E-12
GO:1901880	negative regulation of protein depolymerization	43/4924	1.65E-13	4.44E-12
GO:0042326	negative regulation of phosphorylation	128/4924	1.71E-13	4.58E-12
GO:0022409	positive regulation of cell-cell adhesion	103/4924	1.94E-13	5.17E-12
GO:0010389	regulation of G2/M transition of mitotic cell cycle	49/4924	1.97E-13	5.25E-12
GO:0060606	tube closure	53/4924	2.00E-13	5.29E-12
GO:0006816	calcium ion transport	137/4924	2.50E-13	6.59E-12
GO:0043242	negative regulation of protein-containing complex disassembly	46/4924	2.52E-13	6.60E-12
GO:1904062	regulation of cation transmembrane transport	123/4924	2.52E-13	6.60E-12
GO:0001841	neural tube formation	59/4924	2.81E-13	7.31E-12
GO:0051146	striated muscle cell differentiation	110/4924	2.89E-13	7.50E-12
GO:1905515	non-motile cilium assembly	41/4924	2.95E-13	7.62E-12
GO:0032102	negative regulation of response to external stimulus	128/4924	3.12E-13	8.03E-12
GO:0010971	positive regulation of G2/M transition of mitotic cell cycle	23/4924	3.52E-13	9.03E-12
GO:0110020	regulation of actomyosin structure organization	52/4924	3.56E-13	9.05E-12
GO:1902749	regulation of cell cycle G2/M phase transition	52/4924	3.56E-13	9.05E-12
GO:0007159	leukocyte cell-cell adhesion	123/4924	3.81E-13	9.64E-12
GO:1903034	regulation of response to wounding	70/4924	4.08E-13	1.03E-11

GO:0021537	telencephalon development	92/4924	4.78E-13	1.20E-11
GO:0006936	muscle contraction	106/4924	4.85E-13	1.21E-11
GO:0044458	motile cilium assembly	38/4924	4.92E-13	1.22E-11
GO:0022408	negative regulation of cell-cell adhesion	79/4924	4.92E-13	1.22E-11
GO:0050730	regulation of peptidyl-tyrosine phosphorylation	94/4924	4.93E-13	1.22E-11
GO:0043244	regulation of protein-containing complex disassembly	58/4924	4.95E-13	1.22E-11
GO:0030856	regulation of epithelial cell differentiation	65/4924	5.82E-13	1.42E-11
GO:0072006	nephron development	65/4924	5.82E-13	1.42E-11
GO:0035567	non-canonical Wnt signaling pathway	34/4924	6.27E-13	1.53E-11
GO:1902751	positive regulation of cell cycle G2/M phase transition	24/4924	9.28E-13	2.25E-11
GO:0008361	regulation of cell size	80/4924	9.32E-13	2.25E-11
GO:0010977	negative regulation of neuron projection development	66/4924	9.46E-13	2.28E-11
GO:0097191	extrinsic apoptotic signaling pathway	87/4924	1.21E-12	2.91E-11
GO:0002685	regulation of leukocyte migration	83/4924	1.41E-12	3.36E-11
GO:0001933	negative regulation of protein phosphorylation	114/4924	1.48E-12	3.51E-11
GO:0061387	regulation of extent of cell growth	56/4924	1.54E-12	3.65E-11
GO:0010001	glial cell differentiation	91/4924	1.68E-12	3.94E-11
GO:0033002	muscle cell proliferation	91/4924	1.68E-12	3.94E-11

GO:0032386	regulation of intracellular transport	110/4924	2.07E-12	4.84E-11
GO:1903131	mononuclear cell differentiation	147/4924	2.07E-12	4.84E-11
GO:0048813	dendrite morphogenesis	72/4924	2.41E-12	5.61E-11
GO:0051492	regulation of stress fiber assembly	47/4924	2.61E-12	6.06E-11
GO:2000027	regulation of animal organ morphogenesis	57/4924	2.76E-12	6.38E-11
GO:0050920	regulation of chemotaxis	83/4924	3.14E-12	7.24E-11
GO:0048017	inositol lipid-mediated signaling	68/4924	3.17E-12	7.26E-11
GO:0043406	positive regulation of MAP kinase activity	56/4924	3.33E-12	7.60E-11
GO:0014706	striated muscle tissue development	98/4924	3.38E-12	7.69E-11
GO:0030516	regulation of axon extension	51/4924	3.55E-12	8.06E-11
GO:1902105	regulation of leukocyte differentiation	106/4924	3.57E-12	8.06E-11
GO:0007019	microtubule depolymerization	32/4924	3.85E-12	8.67E-11
GO:0048015	phosphatidylinositol-mediated signaling	67/4924	3.95E-12	8.85E-11
GO:0008544	epidermis development	115/4924	3.96E-12	8.85E-11
GO:0051054	positive regulation of DNA metabolic process	100/4924	4.10E-12	9.14E-11
GO:0070997	neuron death	133/4924	4.13E-12	9.16E-11
GO:0003206	cardiac chamber morphogenesis	62/4924	4.29E-12	9.48E-11
GO:0010594	regulation of endothelial cell migration	68/4924	4.34E-12	9.53E-11

GO:0032272	negative regulation of protein polymerization	42/4924	4.34E-12	9.53E-11
GO:0007416	synapse assembly	73/4924	4.69E-12	1.03E-10
GO:0007422	peripheral nervous system development	39/4924	5.16E-12	1.13E-10
GO:0120193	tight junction organization	45/4924	5.26E-12	1.14E-10
GO:0090257	regulation of muscle system process	89/4924	5.70E-12	1.23E-10
GO:0010948	negative regulation of cell cycle process	97/4924	6.79E-12	1.47E-10
GO:0043588	skin development	105/4924	6.97E-12	1.50E-10
GO:0060828	regulation of canonical Wnt signaling pathway	87/4924	7.22E-12	1.55E-10
GO:0090101	negative regulation of transmembrane receptor protein serine/threonine kinase signaling pathway	55/4924	8.49E-12	1.82E-10
GO:0048660	regulation of smooth muscle cell proliferation	69/4924	8.68E-12	1.85E-10
GO:0090596	sensory organ morphogenesis	104/4924	8.88E-12	1.89E-10
GO:0030098	lymphocyte differentiation	134/4924	9.39E-12	1.99E-10
GO:0099173	postsynapse organization	76/4924	9.60E-12	2.03E-10
GO:1903555	regulation of tumor necrosis factor superfamily cytokine production	71/4924	9.93E-12	2.09E-10
GO:0060840	artery development	51/4924	1.20E-11	2.50E-10
GO:0009314	response to radiation	130/4924	1.20E-11	2.50E-10
GO:0010927	cellular component assembly involved in morphogenesis	53/4924	1.23E-11	2.57E-10
GO:0022411	cellular component disassembly	129/4924	1.27E-11	2.64E-10

GO:0006261	DNA-templated DNA replication	60/4924	1.28E-11	2.65E-10
GO:0098657	import into cell	84/4924	1.49E-11	3.06E-10
GO:0003002	regionalization	119/4924	1.55E-11	3.18E-10
GO:0031099	regeneration	62/4924	1.62E-11	3.31E-10
GO:0050679	positive regulation of epithelial cell proliferation	78/4924	1.75E-11	3.57E-10
GO:1903046	meiotic cell cycle process	79/4924	1.79E-11	3.64E-10
GO:0071706	tumor necrosis factor superfamily cytokine production	72/4924	1.86E-11	3.77E-10
GO:0048675	axon extension	58/4924	1.94E-11	3.93E-10
GO:0050863	regulation of T cell activation	110/4924	1.97E-11	3.97E-10
GO:0002028	regulation of sodium ion transport	45/4924	2.04E-11	4.10E-10
GO:0031334	positive regulation of protein-containing complex assembly	75/4924	2.09E-11	4.19E-10
GO:0071674	mononuclear cell migration	69/4924	2.10E-11	4.20E-10
GO:0022612	gland morphogenesis	62/4924	2.23E-11	4.39E-10
GO:0051092	positive regulation of NF-kappaB transcription factor activity	62/4924	2.23E-11	4.39E-10
GO:0071772	response to BMP	70/4924	2.23E-11	4.39E-10
GO:0071773	cellular response to BMP stimulus	70/4924	2.23E-11	4.39E-10
GO:0098739	import across plasma membrane	71/4924	2.35E-11	4.61E-10
GO:0030509	BMP signaling pathway	67/4924	2.48E-11	4.86E-10

GO:0030512	negative regulation of transforming growth factor beta receptor signaling pathway	38/4924	2.69E-11	5.25E-10
GO:0032872	regulation of stress-activated MAPK cascade	75/4924	2.74E-11	5.32E-10
GO:0006887	exocytosis	125/4924	2.74E-11	5.32E-10
GO:0090263	positive regulation of canonical Wnt signaling pathway	48/4924	2.98E-11	5.76E-10
GO:0032984	protein-containing complex disassembly	83/4924	3.09E-11	5.96E-10
GO:0002062	chondrocyte differentiation	50/4924	3.12E-11	6.00E-10
GO:1905475	regulation of protein localization to membrane	73/4924	3.36E-11	6.44E-10
GO:0007156	homophilic cell adhesion via plasma membrane adhesion molecules	49/4924	3.73E-11	7.09E-10
GO:2000181	negative regulation of blood vessel morphogenesis	49/4924	3.73E-11	7.09E-10
GO:0032680	regulation of tumor necrosis factor production	69/4924	3.74E-11	7.09E-10
GO:1903037	regulation of leukocyte cell-cell adhesion	108/4924	3.88E-11	7.34E-10
GO:0006814	sodium ion transport	85/4924	3.92E-11	7.40E-10
GO:0046847	filopodium assembly	38/4924	4.49E-11	8.45E-10
GO:0048678	response to axon injury	37/4924	4.82E-11	9.02E-10
GO:0061512	protein localization to cilium	37/4924	4.82E-11	9.02E-10
GO:0032409	regulation of transporter activity	97/4924	4.85E-11	9.04E-10
GO:0046785	microtubule polymerization	41/4924	5.39E-11	1.00E-09
GO:1901343	negative regulation of vasculature development	49/4924	5.50E-11	1.02E-09

GO:0007044	cell-substrate junction assembly	44/4924	5.61E-11	1.04E-09
GO:0070302	regulation of stress-activated protein kinase signaling cascade	75/4924	6.03E-11	1.11E-09
GO:0003015	heart process	85/4924	6.27E-11	1.15E-09
GO:0061640	cytoskeleton-dependent cytokinesis	43/4924	6.49E-11	1.19E-09
GO:0032640	tumor necrosis factor production	70/4924	6.82E-11	1.25E-09
GO:0048640	negative regulation of developmental growth	53/4924	7.65E-11	1.39E-09
GO:0048661	positive regulation of smooth muscle cell proliferation	47/4924	7.84E-11	1.42E-09
GO:0043583	ear development	85/4924	7.90E-11	1.43E-09
GO:0050727	regulation of inflammatory response	109/4924	7.98E-11	1.44E-09
GO:1901988	negative regulation of cell cycle phase transition	84/4924	7.99E-11	1.44E-09
GO:0007219	Notch signaling pathway	68/4924	8.27E-11	1.49E-09
GO:0022898	regulation of transmembrane transporter activity	93/4924	8.36E-11	1.50E-09
GO:0048659	smooth muscle cell proliferation	69/4924	8.62E-11	1.54E-09
GO:0050731	positive regulation of peptidyl-tyrosine phosphorylation	70/4924	8.95E-11	1.59E-09
GO:0001736	establishment of planar polarity	33/4924	9.74E-11	1.73E-09
GO:0090175	regulation of establishment of planar polarity	26/4924	1.02E-10	1.80E-09
GO:0045137	development of primary sexual characteristics	83/4924	1.02E-10	1.80E-09
GO:0008406	gonad development	82/4924	1.03E-10	1.81E-09

GO:0043297	apical junction assembly	42/4924	1.16E-10	2.03E-09
GO:0062197	cellular response to chemical stress	105/4924	1.17E-10	2.06E-09
GO:2001236	regulation of extrinsic apoptotic signaling pathway	64/4924	1.19E-10	2.08E-09
GO:1901214	regulation of neuron death	118/4924	1.20E-10	2.09E-09
GO:1901215	negative regulation of neuron death	87/4924	1.20E-10	2.09E-09
GO:0061035	regulation of cartilage development	36/4924	1.45E-10	2.51E-09
GO:0014910	regulation of smooth muscle cell migration	43/4924	1.52E-10	2.63E-09
GO:0060996	dendritic spine development	53/4924	1.53E-10	2.63E-09
GO:0051924	regulation of calcium ion transport	93/4924	1.58E-10	2.71E-09
GO:2001234	negative regulation of apoptotic signaling pathway	83/4924	1.61E-10	2.77E-09
GO:0150115	cell-substrate junction organization	45/4924	1.65E-10	2.81E-09
GO:0021700	developmental maturation	109/4924	1.68E-10	2.87E-09
GO:0010595	positive regulation of endothelial cell migration	47/4924	1.70E-10	2.89E-09
GO:0007164	establishment of tissue polarity	33/4924	1.71E-10	2.90E-09
GO:0006310	DNA recombination	101/4924	1.72E-10	2.90E-09
GO:1903035	negative regulation of response to wounding	42/4924	1.78E-10	3.00E-09
GO:2000177	regulation of neural precursor cell proliferation	50/4924	1.99E-10	3.35E-09
GO:0014909	smooth muscle cell migration	46/4924	2.04E-10	3.42E-09

GO:0085029	extracellular matrix assembly	26/4924	2.15E-10	3.60E-09
GO:0035721	intraciliary retrograde transport	14/4924	2.25E-10	3.75E-09
GO:0007613	memory	61/4924	2.37E-10	3.95E-09
GO:0007088	regulation of mitotic nuclear division	47/4924	2.48E-10	4.10E-09
GO:0016525	negative regulation of angiogenesis	47/4924	2.48E-10	4.10E-09
GO:0050771	negative regulation of axonogenesis	35/4924	2.60E-10	4.29E-09
GO:0032874	positive regulation of stress-activated MAPK cascade	55/4924	2.68E-10	4.41E-09
GO:0031111	negative regulation of microtubule polymerization or depolymerization	28/4924	2.80E-10	4.60E-09
GO:0010935	regulation of macrophage cytokine production	15/4924	2.92E-10	4.78E-09
GO:0010934	macrophage cytokine production	16/4924	2.97E-10	4.86E-09
GO:0045664	regulation of neuron differentiation	81/4924	3.30E-10	5.37E-09
GO:0030100	regulation of endocytosis	80/4924	3.34E-10	5.43E-09
GO:0050866	negative regulation of cell activation	77/4924	3.44E-10	5.58E-09
GO:0071675	regulation of mononuclear cell migration	49/4924	3.47E-10	5.60E-09
GO:1903522	regulation of blood circulation	87/4924	3.58E-10	5.78E-09
GO:0060348	bone development	85/4924	3.78E-10	6.09E-09
GO:0000910	cytokinesis	66/4924	3.95E-10	6.34E-09
GO:0008589	regulation of smoothened signaling pathway	42/4924	4.09E-10	6.54E-09

GO:1901991	negative regulation of mitotic cell cycle phase transition	63/4924	4.65E-10	7.43E-09
GO:0030837	negative regulation of actin filament polymerization	34/4924	4.66E-10	7.43E-09
GO:0035023	regulation of Rho protein signal transduction	41/4924	4.81E-10	7.63E-09
GO:0032835	glomerulus development	33/4924	5.01E-10	7.94E-09
GO:0070304	positive regulation of stress-activated protein kinase signaling cascade	55/4924	5.04E-10	7.97E-09
GO:0070252	actin-mediated cell contraction	43/4924	5.14E-10	8.11E-09
GO:0048144	fibroblast proliferation	47/4924	5.16E-10	8.12E-09
GO:0007612	learning	67/4924	5.28E-10	8.28E-09
GO:0034332	adherens junction organization	28/4924	5.33E-10	8.34E-09
GO:0007528	neuromuscular junction development	31/4924	5.50E-10	8.57E-09
GO:0030199	collagen fibril organization	31/4924	5.50E-10	8.57E-09
GO:0048839	inner ear development	75/4924	5.59E-10	8.68E-09
GO:0006937	regulation of muscle contraction	61/4924	5.62E-10	8.71E-09
GO:0002687	positive regulation of leukocyte migration	59/4924	6.73E-10	1.04E-08
GO:0014812	muscle cell migration	49/4924	6.94E-10	1.07E-08
GO:0035725	sodium ion transmembrane transport	57/4924	7.96E-10	1.22E-08
GO:0032412	regulation of ion transmembrane transporter activity	88/4924	7.99E-10	1.23E-08
GO:0051225	spindle assembly	48/4924	8.54E-10	1.31E-08

GO:0044843	cell cycle G1/S phase transition	78/4924	8.55E-10	1.31E-08
GO:0045055	regulated exocytosis	91/4924	8.68E-10	1.32E-08
GO:0051099	positive regulation of binding	68/4924	8.96E-10	1.36E-08
GO:0006022	aminoglycan metabolic process	46/4924	9.01E-10	1.37E-08
GO:0045619	regulation of lymphocyte differentiation	70/4924	9.09E-10	1.38E-08
GO:0051783	regulation of nuclear division	55/4924	9.31E-10	1.40E-08
GO:0032103	positive regulation of response to external stimulus	130/4924	9.91E-10	1.49E-08
GO:0007517	muscle organ development	109/4924	9.98E-10	1.50E-08
GO:0060047	heart contraction	79/4924	1.05E-09	1.57E-08
GO:0031532	actin cytoskeleton reorganization	47/4924	1.05E-09	1.57E-08
GO:0006941	striated muscle contraction	63/4924	1.05E-09	1.57E-08
GO:0060071	Wnt signaling pathway, planar cell polarity pathway	23/4924	1.10E-09	1.62E-08
GO:0070286	axonemal dynein complex assembly	23/4924	1.10E-09	1.62E-08
GO:0001657	ureteric bud development	45/4924	1.10E-09	1.62E-08
GO:0072163	mesonephric epithelium development	45/4924	1.10E-09	1.62E-08
GO:0072164	mesonephric tubule development	45/4924	1.10E-09	1.62E-08
GO:0051402	neuron apoptotic process	98/4924	1.11E-09	1.62E-08
GO:0140013	meiotic nuclear division	70/4924	1.16E-09	1.70E-08

GO:0030217	T cell differentiation	94/4924	1.36E-09	1.98E-08
GO:0014911	positive regulation of smooth muscle cell migration	32/4924	1.51E-09	2.20E-08
GO:1903039	positive regulation of leukocyte cell-cell adhesion	81/4924	1.53E-09	2.23E-08
GO:0061326	renal tubule development	45/4924	1.57E-09	2.28E-08
GO:0030099	myeloid cell differentiation	125/4924	1.67E-09	2.42E-08
GO:0003279	cardiac septum development	51/4924	1.67E-09	2.42E-08
GO:0055001	muscle cell development	76/4924	1.74E-09	2.51E-08
GO:2001222	regulation of neuron migration	29/4924	1.77E-09	2.55E-08
GO:0045931	positive regulation of mitotic cell cycle	52/4924	1.83E-09	2.63E-08
GO:0014044	Schwann cell development	22/4924	1.87E-09	2.67E-08
GO:0048738	cardiac muscle tissue development	86/4924	1.94E-09	2.78E-08
GO:0043523	regulation of neuron apoptotic process	89/4924	2.08E-09	2.97E-08
GO:0007249	I-kappaB kinase/NF-kappaB signaling	76/4924	2.17E-09	3.09E-08
GO:0001818	negative regulation of cytokine production	92/4924	2.19E-09	3.11E-08
GO:0007292	female gamete generation	61/4924	2.21E-09	3.13E-08
GO:0048145	regulation of fibroblast proliferation	45/4924	2.24E-09	3.16E-08
GO:0120192	tight junction assembly	39/4924	2.27E-09	3.21E-08
GO:0031114	regulation of microtubule depolymerization	23/4924	2.32E-09	3.27E-08

GO:0008016	regulation of heart contraction	68/4924	2.40E-09	3.37E-08
GO:0031102	neuron projection regeneration	31/4924	2.71E-09	3.79E-08
GO:0001938	positive regulation of endothelial cell proliferation	42/4924	2.84E-09	3.97E-08
GO:0018210	peptidyl-threonine modification	50/4924	2.86E-09	3.99E-08
GO:0072009	nephron epithelium development	47/4924	2.90E-09	4.04E-08
GO:0031113	regulation of microtubule polymerization	30/4924	2.91E-09	4.04E-08
GO:0002274	myeloid leukocyte activation	85/4924	3.02E-09	4.19E-08
GO:0043524	negative regulation of neuron apoptotic process	66/4924	3.05E-09	4.22E-08
GO:0045444	fat cell differentiation	84/4924	3.15E-09	4.34E-08
GO:0006270	DNA replication initiation	21/4924	3.16E-09	4.34E-08
GO:0001823	mesonephros development	45/4924	3.16E-09	4.34E-08
GO:0050921	positive regulation of chemotaxis	56/4924	3.17E-09	4.35E-08
GO:1901989	positive regulation of cell cycle phase transition	48/4924	3.24E-09	4.43E-08
GO:0003018	vascular process in circulatory system	77/4924	3.27E-09	4.46E-08
GO:0060993	kidney morphogenesis	43/4924	3.35E-09	4.56E-08
GO:0014065	phosphatidylinositol 3-kinase signaling	52/4924	3.36E-09	4.56E-08
GO:0002040	sprouting angiogenesis	49/4924	3.57E-09	4.84E-08
GO:0010811	positive regulation of cell-substrate adhesion	53/4924	3.59E-09	4.86E-08

GO:0050878	regulation of body fluid levels	109/4924	3.82E-09	5.15E-08
GO:0048705	skeletal system morphogenesis	83/4924	4.01E-09	5.40E-08
GO:0048286	lung alveolus development	32/4924	4.03E-09	5.41E-08
GO:0006874	cellular calcium ion homeostasis	137/4924	4.33E-09	5.81E-08
GO:0048259	regulation of receptor-mediated endocytosis	49/4924	4.88E-09	6.53E-08
GO:2001020	regulation of response to DNA damage stimulus	83/4924	4.90E-09	6.54E-08
GO:0007254	JNK cascade	63/4924	4.92E-09	6.56E-08
GO:0045927	positive regulation of growth	97/4924	4.97E-09	6.61E-08
GO:0051293	establishment of spindle localization	27/4924	5.71E-09	7.58E-08
GO:1902115	regulation of organelle assembly	71/4924	5.86E-09	7.76E-08
GO:0051348	negative regulation of transferase activity	83/4924	5.97E-09	7.88E-08
GO:0030203	glycosaminoglycan metabolic process	40/4924	6.03E-09	7.94E-08
GO:0032273	positive regulation of protein polymerization	40/4924	6.03E-09	7.94E-08
GO:1905477	positive regulation of protein localization to membrane	45/4924	6.20E-09	8.13E-08
GO:0050680	negative regulation of epithelial cell proliferation	61/4924	6.21E-09	8.13E-08
GO:0009100	glycoprotein metabolic process	99/4924	6.22E-09	8.14E-08
GO:0043271	negative regulation of ion transport	62/4924	6.27E-09	8.18E-08
GO:1903532	positive regulation of secretion by cell	106/4924	6.49E-09	8.45E-08

GO:0043534	blood vessel endothelial cell migration	46/4924	6.91E-09	8.99E-08
GO:0001649	osteoblast differentiation	73/4924	6.96E-09	9.03E-08
GO:0007229	integrin-mediated signaling pathway	41/4924	7.14E-09	9.24E-08
GO:0018107	peptidyl-threonine phosphorylation	47/4924	7.62E-09	9.85E-08
GO:0035107	appendage morphogenesis	61/4924	7.97E-09	1.03E-07
GO:0035108	limb morphogenesis	61/4924	7.97E-09	1.03E-07
GO:0002011	morphogenesis of an epithelial sheet	30/4924	8.02E-09	1.03E-07
GO:0001890	placenta development	62/4924	8.02E-09	1.03E-07
GO:0070374	positive regulation of ERK1 and ERK2 cascade	74/4924	8.38E-09	1.07E-07
GO:0048844	artery morphogenesis	36/4924	8.47E-09	1.08E-07
GO:0048146	positive regulation of fibroblast proliferation	33/4924	8.88E-09	1.13E-07
GO:0071214	cellular response to abiotic stimulus	98/4924	9.33E-09	1.18E-07
GO:0104004	cellular response to environmental stimulus	98/4924	9.33E-09	1.18E-07
GO:0060048	cardiac muscle contraction	50/4924	9.60E-09	1.22E-07
GO:0034599	cellular response to oxidative stress	85/4924	9.68E-09	1.22E-07
GO:0048771	tissue remodeling	68/4924	9.70E-09	1.22E-07
GO:0003351	epithelial cilium movement involved in extracellular fluid movement	24/4924	1.00E-08	1.26E-07
GO:0045580	regulation of T cell differentiation	59/4924	1.00E-08	1.26E-07

GO:0001539	cilium or flagellum-dependent cell motility	60/4924	1.01E-08	1.27E-07
GO:0060285	cilium-dependent cell motility	60/4924	1.01E-08	1.27E-07
GO:1901992	positive regulation of mitotic cell cycle phase transition	41/4924	1.02E-08	1.27E-07
GO:0032233	positive regulation of actin filament bundle assembly	32/4924	1.02E-08	1.27E-07
GO:0007093	mitotic cell cycle checkpoint signaling	51/4924	1.02E-08	1.27E-07
GO:0007369	gastrulation	62/4924	1.02E-08	1.27E-07
GO:0045862	positive regulation of proteolysis	104/4924	1.03E-08	1.27E-07
GO:0006858	extracellular transport	25/4924	1.03E-08	1.27E-07
GO:0090100	positive regulation of transmembrane receptor protein serine/threonine kinase signaling pathway	47/4924	1.04E-08	1.28E-07
GO:1902414	protein localization to cell junction	47/4924	1.04E-08	1.28E-07
GO:0034446	substrate adhesion-dependent cell spreading	44/4924	1.07E-08	1.31E-07
GO:0022011	myelination in peripheral nervous system	20/4924	1.19E-08	1.45E-07
GO:0032292	peripheral nervous system axon ensheathment	20/4924	1.19E-08	1.45E-07
GO:0009913	epidermal cell differentiation	76/4924	1.19E-08	1.45E-07
GO:0044344	cellular response to fibroblast growth factor stimulus	40/4924	1.25E-08	1.52E-07
GO:0061041	regulation of wound healing	50/4924	1.28E-08	1.56E-07
GO:0006302	double-strand break repair	83/4924	1.29E-08	1.57E-07
GO:0051058	negative regulation of small GTPase mediated signal transduction	30/4924	1.30E-08	1.58E-07

GO:0051383	kinetochore organization	16/4924	1.36E-08	1.64E-07
GO:0021782	glial cell development	47/4924	1.41E-08	1.71E-07
GO:0007026	negative regulation of microtubule depolymerization	21/4924	1.45E-08	1.75E-07
GO:1904035	regulation of epithelial cell apoptotic process	39/4924	1.52E-08	1.83E-07
GO:0030834	regulation of actin filament depolymerization	28/4924	1.58E-08	1.89E-07
GO:0030858	positive regulation of epithelial cell differentiation	28/4924	1.58E-08	1.89E-07
GO:0051653	spindle localization	28/4924	1.58E-08	1.89E-07
GO:0002367	cytokine production involved in immune response	45/4924	1.63E-08	1.94E-07
GO:0120034	positive regulation of plasma membrane bounded cell projection assembly	45/4924	1.63E-08	1.94E-07
GO:0030510	regulation of BMP signaling pathway	42/4924	1.64E-08	1.95E-07
GO:0090288	negative regulation of cellular response to growth factor stimulus	42/4924	1.64E-08	1.95E-07
GO:0030866	cortical actin cytoskeleton organization	22/4924	1.65E-08	1.96E-07
GO:0044786	cell cycle DNA replication	22/4924	1.65E-08	1.96E-07
GO:0001570	vasculogenesis	40/4924	1.77E-08	2.09E-07
GO:0046546	development of primary male sexual characteristics	52/4924	1.87E-08	2.20E-07
GO:0009416	response to light stimulus	95/4924	1.88E-08	2.21E-07
GO:0007548	sex differentiation	94/4924	2.01E-08	2.35E-07
GO:0050708	regulation of protein secretion	91/4924	2.04E-08	2.39E-07

GO:0007009	plasma membrane organization	56/4924	2.07E-08	2.42E-07
GO:0048592	eye morphogenesis	63/4924	2.07E-08	2.42E-07
GO:0030865	cortical cytoskeleton organization	30/4924	2.07E-08	2.42E-07
GO:0051592	response to calcium ion	49/4924	2.16E-08	2.51E-07
GO:0051983	regulation of chromosome segregation	39/4924	2.17E-08	2.52E-07
GO:0030042	actin filament depolymerization	29/4924	2.33E-08	2.71E-07
GO:0071774	response to fibroblast growth factor	40/4924	2.50E-08	2.89E-07
GO:0033260	nuclear DNA replication	20/4924	2.53E-08	2.92E-07
GO:0060326	cell chemotaxis	93/4924	2.53E-08	2.92E-07
GO:0043122	regulation of I-kappaB kinase/NF-kappaB signaling	64/4924	2.57E-08	2.95E-07
GO:0032388	positive regulation of intracellular transport	63/4924	2.61E-08	2.99E-07
GO:0016570	histone modification	133/4924	2.63E-08	3.01E-07
GO:0001935	endothelial cell proliferation	62/4924	2.64E-08	3.02E-07
GO:0060976	coronary vasculature development	34/4924	2.66E-08	3.04E-07
GO:1903707	negative regulation of hemopoiesis	44/4924	2.78E-08	3.17E-07
GO:0072080	nephron tubule development	41/4924	2.83E-08	3.20E-07
GO:0000281	mitotic cytokinesis	31/4924	2.84E-08	3.20E-07
GO:0031103	axon regeneration	27/4924	2.84E-08	3.20E-07

GO:0032330	regulation of chondrocyte differentiation	27/4924	2.84E-08	3.20E-07
GO:0035088	establishment or maintenance of apical/basal cell polarity	27/4924	2.84E-08	3.20E-07
GO:0061245	establishment or maintenance of bipolar cell polarity	27/4924	2.84E-08	3.20E-07
GO:0002697	regulation of immune effector process	120/4924	2.94E-08	3.31E-07
GO:0048736	appendage development	67/4924	2.99E-08	3.35E-07
GO:0060173	limb development	67/4924	2.99E-08	3.35E-07
GO:0008277	regulation of G protein-coupled receptor signaling pathway	50/4924	3.00E-08	3.35E-07
GO:0034763	negative regulation of transmembrane transport	51/4924	3.11E-08	3.47E-07
GO:0006942	regulation of striated muscle contraction	37/4924	3.25E-08	3.62E-07
GO:0014037	Schwann cell differentiation	23/4924	3.31E-08	3.68E-07
GO:0043270	positive regulation of ion transport	96/4924	3.41E-08	3.79E-07
GO:0086005	ventricular cardiac muscle cell action potential	18/4924	3.41E-08	3.79E-07
GO:0016311	dephosphorylation	100/4924	3.55E-08	3.94E-07
GO:0045620	negative regulation of lymphocyte differentiation	29/4924	3.72E-08	4.11E-07
GO:0017156	calcium-ion regulated exocytosis	32/4924	3.72E-08	4.11E-07
GO:0055117	regulation of cardiac muscle contraction	32/4924	3.72E-08	4.11E-07
GO:0035050	embryonic heart tube development	38/4924	3.79E-08	4.17E-07
GO:0071692	protein localization to extracellular region	113/4924	3.84E-08	4.22E-07

GO:1901652	response to peptide	116/4924	4.04E-08	4.43E-07
GO:0008584	male gonad development	51/4924	4.06E-08	4.45E-07
GO:0043409	negative regulation of MAPK cascade	63/4924	4.10E-08	4.48E-07
GO:0052547	regulation of peptidase activity	121/4924	4.10E-08	4.48E-07
GO:0001895	retina homeostasis	28/4924	4.20E-08	4.57E-07
GO:0060041	retina development in camera-type eye	61/4924	4.24E-08	4.61E-07
GO:0032612	interleukin-1 production	46/4924	4.34E-08	4.71E-07
GO:0061082	myeloid leukocyte cytokine production	19/4924	4.38E-08	4.74E-07
GO:0071711	basement membrane organization	19/4924	4.38E-08	4.74E-07
GO:1901653	cellular response to peptide	92/4924	4.47E-08	4.82E-07
GO:0071695	anatomical structure maturation	89/4924	4.60E-08	4.96E-07
GO:0002263	cell activation involved in immune response	96/4924	4.71E-08	5.07E-07
GO:0002695	negative regulation of leukocyte activation	66/4924	4.73E-08	5.08E-07
GO:1902106	negative regulation of leukocyte differentiation	43/4924	4.74E-08	5.08E-07
GO:0070830	bicellular tight junction assembly	35/4924	4.79E-08	5.13E-07
GO:0050870	positive regulation of T cell activation	71/4924	4.87E-08	5.20E-07
GO:0048863	stem cell differentiation	75/4924	4.96E-08	5.30E-07
GO:0048008	platelet-derived growth factor receptor signaling pathway	30/4924	5.08E-08	5.41E-07

GO:0021591	ventricular system development	20/4924	5.14E-08	5.47E-07
GO:0034764	positive regulation of transmembrane transport	78/4924	5.16E-08	5.48E-07
GO:0007405	neuroblast proliferation	32/4924	5.61E-08	5.93E-07
GO:0045428	regulation of nitric oxide biosynthetic process	32/4924	5.61E-08	5.93E-07
GO:0048041	focal adhesion assembly	36/4924	5.68E-08	6.00E-07
GO:0003014	renal system process	46/4924	5.79E-08	6.09E-07
GO:0042542	response to hydrogen peroxide	46/4924	5.79E-08	6.09E-07
GO:0045494	photoreceptor cell maintenance	24/4924	5.80E-08	6.10E-07
GO:0006979	response to oxidative stress	113/4924	5.89E-08	6.18E-07
GO:0002718	regulation of cytokine production involved in immune response	39/4924	6.06E-08	6.35E-07
GO:0048639	positive regulation of developmental growth	70/4924	6.20E-08	6.48E-07
GO:1903557	positive regulation of tumor necrosis factor superfamily cytokine production	43/4924	6.43E-08	6.71E-07
GO:0055123	digestive system development	50/4924	6.73E-08	7.01E-07
GO:0098659	inorganic cation import across plasma membrane	44/4924	6.89E-08	7.15E-07
GO:0099587	inorganic ion import across plasma membrane	44/4924	6.89E-08	7.15E-07
GO:0000075	cell cycle checkpoint signaling	59/4924	6.90E-08	7.16E-07
GO:0033044	regulation of chromosome organization	67/4924	6.96E-08	7.20E-07
GO:0080164	regulation of nitric oxide metabolic process	33/4924	6.97E-08	7.20E-07

GO:0006820	anion transport	132/4924	6.99E-08	7.21E-07
GO:0001936	regulation of endothelial cell proliferation	57/4924	7.06E-08	7.26E-07
GO:0003231	cardiac ventricle development	53/4924	7.06E-08	7.26E-07
GO:0072698	protein localization to microtubule cytoskeleton	27/4924	7.55E-08	7.75E-07
GO:0051222	positive regulation of protein transport	93/4924	8.00E-08	8.20E-07
GO:0045807	positive regulation of endocytosis	47/4924	8.02E-08	8.21E-07
GO:0060411	cardiac septum morphogenesis	36/4924	8.10E-08	8.28E-07
GO:0017157	regulation of exocytosis	76/4924	8.32E-08	8.49E-07
GO:0031214	biomineral tissue development	59/4924	8.66E-08	8.82E-07
GO:0010038	response to metal ion	87/4924	8.68E-08	8.82E-07
GO:0009914	hormone transport	106/4924	8.83E-08	8.97E-07
GO:0046328	regulation of JNK cascade	53/4924	9.05E-08	9.16E-07
GO:0000725	recombinational repair	55/4924	9.05E-08	9.16E-07
GO:0045744	negative regulation of G protein-coupled receptor signaling pathway	29/4924	9.08E-08	9.18E-07
GO:0001656	metanephros development	37/4924	9.29E-08	9.36E-07
GO:0060395	SMAD protein signal transduction	40/4924	9.29E-08	9.36E-07
GO:0052548	regulation of endopeptidase activity	101/4924	9.65E-08	9.71E-07
GO:0051963	regulation of synapse assembly	45/4924	9.72E-08	9.74E-07

GO:1904019	epithelial cell apoptotic process	45/4924	9.72E-08	9.74E-07
GO:0009306	protein secretion	110/4924	9.90E-08	9.90E-07
GO:0086003	cardiac muscle cell contraction	31/4924	9.96E-08	9.95E-07
GO:0090162	establishment of epithelial cell polarity	20/4924	1.01E-07	1.00E-06
GO:0002090	regulation of receptor internalization	33/4924	1.02E-07	1.02E-06
GO:0002366	leukocyte activation involved in immune response	94/4924	1.02E-07	1.02E-06
GO:0110148	biomineralization	61/4924	1.04E-07	1.03E-06
GO:0050919	negative chemotaxis	23/4924	1.05E-07	1.04E-06
GO:0045685	regulation of glial cell differentiation	38/4924	1.05E-07	1.04E-06
GO:0035924	cellular response to vascular endothelial growth factor stimulus	28/4924	1.05E-07	1.04E-06
GO:0044380	protein localization to cytoskeleton	28/4924	1.05E-07	1.04E-06
GO:0014013	regulation of gliogenesis	47/4924	1.05E-07	1.04E-06
GO:0060674	placenta blood vessel development	21/4924	1.06E-07	1.04E-06
GO:0033157	regulation of intracellular protein transport	71/4924	1.07E-07	1.05E-06
GO:0003158	endothelium development	48/4924	1.09E-07	1.06E-06
GO:0000082	G1/S transition of mitotic cell cycle	66/4924	1.09E-07	1.07E-06
GO:0032760	positive regulation of tumor necrosis factor production	42/4924	1.09E-07	1.07E-06
GO:0035592	establishment of protein localization to extracellular region	110/4924	1.14E-07	1.11E-06

GO:2001235	positive regulation of apoptotic signaling pathway	54/4924	1.15E-07	1.12E-06
GO:1902305	regulation of sodium ion transmembrane transport	30/4924	1.18E-07	1.15E-06
GO:0032964	collagen biosynthetic process	27/4924	1.20E-07	1.17E-06
GO:0008543	fibroblast growth factor receptor signaling pathway	34/4924	1.21E-07	1.18E-06
GO:1903510	mucopolysaccharide metabolic process	32/4924	1.23E-07	1.19E-06
GO:0014066	regulation of phosphatidylinositol 3-kinase signaling	40/4924	1.27E-07	1.23E-06
GO:0071897	DNA biosynthetic process	60/4924	1.32E-07	1.28E-06
GO:0009612	response to mechanical stimulus	59/4924	1.35E-07	1.31E-06
GO:0008347	glial cell migration	29/4924	1.39E-07	1.34E-06
GO:0051310	metaphase plate congression	29/4924	1.39E-07	1.34E-06
GO:0051250	negative regulation of lymphocyte activation	57/4924	1.41E-07	1.35E-06
GO:0032675	regulation of interleukin-6 production	56/4924	1.43E-07	1.37E-06
GO:0002683	negative regulation of immune system process	120/4924	1.47E-07	1.41E-06
GO:0009101	glycoprotein biosynthetic process	81/4924	1.51E-07	1.45E-06
GO:0045332	phospholipid translocation	25/4924	1.52E-07	1.45E-06
GO:0032092	positive regulation of protein binding	39/4924	1.59E-07	1.51E-06
GO:0051346	negative regulation of hydrolase activity	102/4924	1.61E-07	1.53E-06
GO:0061333	renal tubule morphogenesis	36/4924	1.61E-07	1.53E-06

GO:0002573	myeloid leukocyte differentiation	74/4924	1.61E-07	1.53E-06
GO:0019221	cytokine-mediated signaling pathway	114/4924	1.63E-07	1.54E-06
GO:0045766	positive regulation of angiogenesis	60/4924	1.64E-07	1.55E-06
GO:1904018	positive regulation of vasculature development	60/4924	1.64E-07	1.55E-06
GO:0010718	positive regulation of epithelial to mesenchymal transition	24/4924	1.66E-07	1.57E-06
GO:0030517	negative regulation of axon extension	24/4924	1.66E-07	1.57E-06
GO:0090307	mitotic spindle assembly	30/4924	1.77E-07	1.66E-06
GO:0051047	positive regulation of secretion	113/4924	1.77E-07	1.66E-06
GO:0040036	regulation of fibroblast growth factor receptor signaling pathway	19/4924	1.79E-07	1.68E-06
GO:0051298	centrosome duplication	32/4924	1.80E-07	1.68E-06
GO:0042490	mechanoreceptor differentiation	37/4924	1.80E-07	1.68E-06
GO:0043535	regulation of blood vessel endothelial cell migration	37/4924	1.80E-07	1.68E-06
GO:0060294	cilium movement involved in cell motility	55/4924	1.83E-07	1.70E-06
GO:0048593	camera-type eye morphogenesis	53/4924	1.87E-07	1.74E-06
GO:0120316	sperm flagellum assembly	20/4924	1.90E-07	1.76E-06
GO:1904645	response to amyloid-beta	21/4924	1.93E-07	1.79E-06
GO:0032652	regulation of interleukin-1 production	43/4924	2.06E-07	1.91E-06
GO:0071277	cellular response to calcium ion	33/4924	2.12E-07	1.96E-06

GO:0070661	leukocyte proliferation	102/4924	2.14E-07	1.98E-06
GO:0008306	associative learning	44/4924	2.15E-07	1.98E-06
GO:0046580	negative regulation of Ras protein signal transduction	26/4924	2.16E-07	1.99E-06
GO:0050772	positive regulation of axonogenesis	39/4924	2.17E-07	1.99E-06
GO:0061162	establishment of monopolar cell polarity	16/4924	2.21E-07	2.02E-06
GO:1990845	adaptive thermogenesis	55/4924	2.30E-07	2.10E-06
GO:2001237	negative regulation of extrinsic apoptotic signaling pathway	40/4924	2.33E-07	2.13E-06
GO:0034767	positive regulation of ion transmembrane transport	62/4924	2.34E-07	2.14E-06
GO:1904951	positive regulation of establishment of protein localization	95/4924	2.38E-07	2.17E-06
GO:0006898	receptor-mediated endocytosis	79/4924	2.42E-07	2.20E-06
GO:0043281	regulation of cysteine-type endopeptidase activity involved in apoptotic process	66/4924	2.42E-07	2.20E-06
GO:0045839	negative regulation of mitotic nuclear division	25/4924	2.45E-07	2.22E-06
GO:2000116	regulation of cysteine-type endopeptidase activity	73/4924	2.45E-07	2.22E-06
GO:0010171	body morphogenesis	28/4924	2.48E-07	2.25E-06
GO:0051899	membrane depolarization	37/4924	2.49E-07	2.25E-06
GO:1905818	regulation of chromosome separation	32/4924	2.60E-07	2.34E-06
GO:0060425	lung morphogenesis	30/4924	2.62E-07	2.36E-06
GO:0002700	regulation of production of molecular mediator of immune response	58/4924	2.67E-07	2.41E-06

GO:0006939	smooth muscle contraction	43/4924	2.72E-07	2.45E-06
GO:0038127	ERBB signaling pathway	43/4924	2.72E-07	2.45E-06
GO:0071526	semaphorin-plexin signaling pathway	24/4924	2.73E-07	2.45E-06
GO:0050905	neuromuscular process	63/4924	2.76E-07	2.47E-06
GO:0072028	nephron morphogenesis	35/4924	2.78E-07	2.49E-06
GO:0051321	meiotic cell cycle	94/4924	2.97E-07	2.66E-06
GO:0051145	smooth muscle cell differentiation	33/4924	3.03E-07	2.70E-06
GO:0060688	regulation of morphogenesis of a branching structure	29/4924	3.14E-07	2.79E-06
GO:0098900	regulation of action potential	29/4924	3.14E-07	2.79E-06
GO:1905330	regulation of morphogenesis of an epithelium	31/4924	3.16E-07	2.81E-06
GO:0007288	sperm axoneme assembly	18/4924	3.18E-07	2.81E-06
GO:0042634	regulation of hair cycle	18/4924	3.18E-07	2.81E-06
GO:0061037	negative regulation of cartilage development	18/4924	3.18E-07	2.81E-06
GO:0086002	cardiac muscle cell action potential involved in contraction	22/4924	3.24E-07	2.86E-06
GO:2000649	regulation of sodium ion transmembrane transporter activity	26/4924	3.37E-07	2.98E-06
GO:0090504	epiboly	19/4924	3.40E-07	3.00E-06
GO:0006809	nitric oxide biosynthetic process	34/4924	3.45E-07	3.03E-06
GO:0034109	homotypic cell-cell adhesion	34/4924	3.45E-07	3.03E-06

GO:0050000	chromosome localization	34/4924	3.45E-07	3.03E-06
GO:0035329	hippo signaling	20/4924	3.47E-07	3.03E-06
GO:0048846	axon extension involved in axon guidance	20/4924	3.47E-07	3.03E-06
GO:0060441	epithelial tube branching involved in lung morphogenesis	20/4924	3.47E-07	3.03E-06
GO:1902284	neuron projection extension involved in neuron projection guidance	20/4924	3.47E-07	3.03E-06
GO:0035089	establishment of apical/basal cell polarity	15/4924	3.72E-07	3.24E-06
GO:0000724	double-strand break repair via homologous recombination	53/4924	3.74E-07	3.25E-06
GO:0046879	hormone secretion	102/4924	3.75E-07	3.26E-06
GO:1902017	regulation of cilium assembly	30/4924	3.83E-07	3.32E-06
GO:2001251	negative regulation of chromosome organization	35/4924	3.87E-07	3.35E-06
GO:0000018	regulation of DNA recombination	47/4924	3.87E-07	3.35E-06
GO:0034204	lipid translocation	25/4924	3.88E-07	3.35E-06
GO:0099565	chemical synaptic transmission, postsynaptic	39/4924	3.94E-07	3.40E-06
GO:0070588	calcium ion transmembrane transport	86/4924	3.97E-07	3.42E-06
GO:0070663	regulation of leukocyte proliferation	79/4924	4.00E-07	3.44E-06
GO:0019932	second-messenger-mediated signaling	88/4924	4.01E-07	3.44E-06
GO:0035265	organ growth	67/4924	4.09E-07	3.51E-06
GO:0032635	interleukin-6 production	57/4924	4.22E-07	3.61E-06

GO:0042552	myelination	57/4924	4.22E-07	3.61E-06
GO:1902806	regulation of cell cycle G1/S phase transition	56/4924	4.35E-07	3.71E-06
GO:1903053	regulation of extracellular matrix organization	24/4924	4.41E-07	3.76E-06
GO:0042098	T cell proliferation	69/4924	4.42E-07	3.76E-06
GO:0030307	positive regulation of cell growth	65/4924	4.53E-07	3.85E-06
GO:0150076	neuroinflammatory response	29/4924	4.63E-07	3.93E-06
GO:0001659	temperature homeostasis	60/4924	4.66E-07	3.96E-06
GO:1903828	negative regulation of protein localization	68/4924	4.67E-07	3.96E-06
GO:0072088	nephron epithelium morphogenesis	34/4924	4.81E-07	4.07E-06
GO:0061339	establishment or maintenance of monopolar cell polarity	16/4924	4.82E-07	4.07E-06
GO:0001508	action potential	50/4924	4.91E-07	4.14E-06
GO:0051480	regulation of cytosolic calcium ion concentration	107/4924	4.91E-07	4.14E-06
GO:0030835	negative regulation of actin filament depolymerization	23/4924	4.93E-07	4.15E-06
GO:0030326	embryonic limb morphogenesis	49/4924	4.95E-07	4.15E-06
GO:0035113	embryonic appendage morphogenesis	49/4924	4.95E-07	4.15E-06
GO:0033627	cell adhesion mediated by integrin	35/4924	5.33E-07	4.47E-06
GO:0007596	blood coagulation	56/4924	5.38E-07	4.51E-06
GO:0098534	centriole assembly	22/4924	5.43E-07	4.54E-06

GO:0072676	lymphocyte migration	40/4924	5.54E-07	4.63E-06
GO:0030239	myofibril assembly	30/4924	5.55E-07	4.63E-06
GO:0002819	regulation of adaptive immune response	71/4924	5.63E-07	4.69E-06
GO:0021987	cerebral cortex development	41/4924	5.77E-07	4.80E-06
GO:0070301	cellular response to hydrogen peroxide	36/4924	5.82E-07	4.83E-06
GO:0007099	centriole replication	21/4924	5.85E-07	4.84E-06
GO:0071604	transforming growth factor beta production	21/4924	5.85E-07	4.84E-06
GO:1902692	regulation of neuroblast proliferation	21/4924	5.85E-07	4.84E-06
GO:0034260	negative regulation of GTPase activity	18/4924	6.09E-07	5.04E-06
GO:0031112	positive regulation of microtubule polymerization or depolymerization	20/4924	6.15E-07	5.07E-06
GO:0099003	vesicle-mediated transport in synapse	75/4924	6.16E-07	5.08E-06
GO:0051797	regulation of hair follicle development	14/4924	6.19E-07	5.09E-06
GO:0002224	toll-like receptor signaling pathway	44/4924	6.21E-07	5.10E-06
GO:0032963	collagen metabolic process	44/4924	6.21E-07	5.10E-06
GO:0030282	bone mineralization	45/4924	6.29E-07	5.15E-06
GO:0061982	meiosis I cell cycle process	47/4924	6.33E-07	5.19E-06
GO:0098727	maintenance of cell number	57/4924	6.42E-07	5.25E-06
GO:0031644	regulation of nervous system process	61/4924	6.65E-07	5.42E-06

GO:0032147	activation of protein kinase activity	38/4924	6.66E-07	5.42E-06
GO:0032611	interleukin-1 beta production	38/4924	6.66E-07	5.42E-06
GO:0090630	activation of GTPase activity	38/4924	6.66E-07	5.42E-06
GO:0071248	cellular response to metal ion	55/4924	6.87E-07	5.58E-06
GO:0046330	positive regulation of JNK cascade	39/4924	7.01E-07	5.69E-06
GO:0046209	nitric oxide metabolic process	35/4924	7.30E-07	5.91E-06
GO:0032755	positive regulation of interleukin-6 production	40/4924	7.31E-07	5.92E-06
GO:0006469	negative regulation of protein kinase activity	63/4924	7.33E-07	5.92E-06
GO:0051057	positive regulation of small GTPase mediated signal transduction	32/4924	7.41E-07	5.99E-06
GO:0045446	endothelial cell differentiation	42/4924	7.76E-07	6.26E-06
GO:0098840	protein transport along microtubule	Nov-24	7.87E-07	6.31E-06
GO:0099118	microtubule-based protein transport	Nov-24	7.87E-07	6.31E-06
GO:1902969	mitotic DNA replication	Nov-24	7.87E-07	6.31E-06
GO:0007272	ensheathment of neurons	57/4924	7.88E-07	6.31E-06
GO:0008366	axon ensheathment	57/4924	7.88E-07	6.31E-06
GO:0046545	development of primary female sexual characteristics	43/4924	7.91E-07	6.33E-06
GO:0003382	epithelial cell morphogenesis	23/4924	7.94E-07	6.34E-06
GO:0045197	establishment or maintenance of epithelial cell apical/basal polarity	23/4924	7.94E-07	6.34E-06

GO:0046579	positive regulation of Ras protein signal transduction	30/4924	7.97E-07	6.35E-06
GO:0055002	striated muscle cell development	30/4924	7.97E-07	6.35E-06
GO:1904375	regulation of protein localization to cell periphery	48/4924	7.99E-07	6.35E-06
GO:0030278	regulation of ossification	47/4924	8.05E-07	6.39E-06
GO:0042476	odontogenesis	45/4924	8.07E-07	6.40E-06
GO:1903169	regulation of calcium ion transmembrane transport	56/4924	8.18E-07	6.48E-06
GO:0072078	nephron tubule morphogenesis	33/4924	8.30E-07	6.57E-06
GO:0032331	negative regulation of chondrocyte differentiation	15/4924	8.34E-07	6.59E-06
GO:1904377	positive regulation of protein localization to cell periphery	29/4924	9.76E-07	7.70E-06
GO:0038084	vascular endothelial growth factor signaling pathway	21/4924	9.82E-07	7.74E-06
GO:0031122	cytoplasmic microtubule organization	27/4924	9.86E-07	7.75E-06
GO:0051784	negative regulation of nuclear division	27/4924	9.86E-07	7.75E-06
GO:0000727	double-strand break repair via break-induced replication	Oct-24	9.93E-07	7.79E-06
GO:0060907	positive regulation of macrophage cytokine production	Oct-24	9.93E-07	7.79E-06
GO:0007599	hemostasis	56/4924	1.00E-06	7.86E-06
GO:0050817	coagulation	56/4924	1.00E-06	7.86E-06
GO:0008585	female gonad development	42/4924	1.01E-06	7.86E-06
GO:0032602	chemokine production	42/4924	1.01E-06	7.86E-06

GO:0002285	lymphocyte activation involved in immune response	67/4924	1.02E-06	7.93E-06
GO:0032411	positive regulation of transporter activity	46/4924	1.03E-06	8.00E-06
GO:0048565	digestive tract development	44/4924	1.03E-06	8.00E-06
GO:0002931	response to ischemia	20/4924	1.06E-06	8.22E-06
GO:0014031	mesenchymal cell development	36/4924	1.06E-06	8.22E-06
GO:0048864	stem cell development	36/4924	1.06E-06	8.22E-06
GO:0030178	negative regulation of Wnt signaling pathway	54/4924	1.08E-06	8.37E-06
GO:0045581	negative regulation of T cell differentiation	24/4924	1.09E-06	8.38E-06
GO:0061005	cell differentiation involved in kidney development	24/4924	1.09E-06	8.38E-06
GO:0010719	negative regulation of epithelial to mesenchymal transition	19/4924	1.11E-06	8.56E-06
GO:0014033	neural crest cell differentiation	37/4924	1.12E-06	8.64E-06
GO:0044319	wound healing, spreading of cells	18/4924	1.13E-06	8.64E-06
GO:0090505	epiboly involved in wound healing	18/4924	1.13E-06	8.64E-06
GO:1904646	cellular response to amyloid-beta	18/4924	1.13E-06	8.64E-06
GO:0061045	negative regulation of wound healing	30/4924	1.13E-06	8.68E-06
GO:0033673	negative regulation of kinase activity	68/4924	1.14E-06	8.73E-06
GO:0032722	positive regulation of chemokine production	33/4924	1.14E-06	8.73E-06
GO:0072089	stem cell proliferation	33/4924	1.14E-06	8.73E-06

GO:0006470	protein dephosphorylation	73/4924	1.16E-06	8.84E-06
GO:0032944	regulation of mononuclear cell proliferation	73/4924	1.16E-06	8.84E-06
GO:0010952	positive regulation of peptidase activity	57/4924	1.18E-06	8.97E-06
GO:0097035	regulation of membrane lipid distribution	26/4924	1.18E-06	8.98E-06
GO:0035264	multicellular organism growth	67/4924	1.21E-06	9.22E-06
GO:0051147	regulation of muscle cell differentiation	50/4924	1.22E-06	9.22E-06
GO:1903038	negative regulation of leukocyte cell-cell adhesion	49/4924	1.24E-06	9.42E-06
GO:0032642	regulation of chemokine production	40/4924	1.26E-06	9.49E-06
GO:0046661	male sex differentiation	55/4924	1.28E-06	9.67E-06
GO:0048872	homeostasis of number of cells	100/4924	1.30E-06	9.78E-06
GO:1902107	positive regulation of leukocyte differentiation	59/4924	1.31E-06	9.85E-06
GO:1903708	positive regulation of hemopoiesis	59/4924	1.31E-06	9.85E-06
GO:0042770	signal transduction in response to DNA damage	54/4924	1.33E-06	1.00E-05
GO:0043367	CD4-positive, alpha-beta T cell differentiation	35/4924	1.34E-06	1.00E-05
GO:2001057	reactive nitrogen species metabolic process	35/4924	1.34E-06	1.00E-05
GO:0035051	cardiocyte differentiation	58/4924	1.37E-06	1.03E-05
GO:0035904	aorta development	29/4924	1.39E-06	1.04E-05
GO:0035019	somatic stem cell population maintenance	25/4924	1.41E-06	1.05E-05

GO:0043536	positive regulation of blood vessel endothelial cell migration	25/4924	1.41E-06	1.05E-05
GO:0051304	chromosome separation	37/4924	1.49E-06	1.11E-05
GO:0002822	regulation of adaptive immune response based on somatic recombination of immune receptors built from immunoglobulin superfamily domains	66/4924	1.54E-06	1.15E-05
GO:0006836	neurotransmitter transport	71/4924	1.57E-06	1.16E-05
GO:0019827	stem cell population maintenance	55/4924	1.57E-06	1.16E-05
GO:0051100	negative regulation of binding	54/4924	1.63E-06	1.21E-05
GO:0001666	response to hypoxia	65/4924	1.64E-06	1.21E-05
GO:0046631	alpha-beta T cell activation	58/4924	1.67E-06	1.23E-05
GO:0014009	glial cell proliferation	26/4924	1.75E-06	1.29E-05
GO:0098815	modulation of excitatory postsynaptic potential	26/4924	1.75E-06	1.29E-05
GO:0032232	negative regulation of actin filament bundle assembly	20/4924	1.78E-06	1.31E-05
GO:0030902	hindbrain development	56/4924	1.83E-06	1.35E-05
GO:0097722	sperm motility	50/4924	1.88E-06	1.38E-05
GO:0086091	regulation of heart rate by cardiac conduction	19/4924	1.92E-06	1.41E-05
GO:0030317	flagellated sperm motility	49/4924	1.93E-06	1.42E-05
GO:2001056	positive regulation of cysteine-type endopeptidase activity	49/4924	1.93E-06	1.42E-05
GO:0038034	signal transduction in absence of ligand	32/4924	1.96E-06	1.43E-05
GO:0051303	establishment of chromosome localization	32/4924	1.96E-06	1.43E-05

GO:0097192	extrinsic apoptotic signaling pathway in absence of ligand	32/4924	1.96E-06	1.43E-05
GO:0097061	dendritic spine organization	37/4924	1.97E-06	1.44E-05
GO:0010950	positive regulation of endopeptidase activity	54/4924	2.00E-06	1.46E-05
GO:0031116	positive regulation of microtubule polymerization	18/4924	2.01E-06	1.46E-05
GO:1901889	negative regulation of cell junction assembly	17/4924	2.03E-06	1.48E-05
GO:0031623	receptor internalization	46/4924	2.07E-06	1.50E-05
GO:0042303	molting cycle	46/4924	2.07E-06	1.50E-05
GO:0042633	hair cycle	46/4924	2.07E-06	1.50E-05
GO:0071901	negative regulation of protein serine/threonine kinase activity	39/4924	2.08E-06	1.50E-05
GO:0106027	neuron projection organization	39/4924	2.08E-06	1.50E-05
GO:2000779	regulation of double-strand break repair	39/4924	2.08E-06	1.50E-05
GO:0051893	regulation of focal adhesion assembly	27/4924	2.09E-06	1.51E-05
GO:0090109	regulation of cell-substrate junction assembly	27/4924	2.09E-06	1.51E-05
GO:0006493	protein O-linked glycosylation	25/4924	2.10E-06	1.51E-05
GO:0048538	thymus development	25/4924	2.10E-06	1.51E-05
GO:0061383	trabecula morphogenesis	25/4924	2.10E-06	1.51E-05
GO:0050868	negative regulation of T cell activation	45/4924	2.10E-06	1.51E-05
GO:0046632	alpha-beta T cell differentiation	44/4924	2.12E-06	1.52E-05

GO:0060113	inner ear receptor cell differentiation	33/4924	2.13E-06	1.52E-05
GO:0032943	mononuclear cell proliferation	92/4924	2.19E-06	1.56E-05
GO:0007281	germ cell development	102/4924	2.37E-06	1.69E-05
GO:0034501	protein localization to kinetochore	13/4924	2.44E-06	1.73E-05
GO:1903083	protein localization to condensed chromosome	13/4924	2.44E-06	1.73E-05
GO:0061337	cardiac conduction	28/4924	2.44E-06	1.73E-05
GO:0120161	regulation of cold-induced thermogenesis	48/4924	2.47E-06	1.76E-05
GO:0035272	exocrine system development	24/4924	2.51E-06	1.78E-05
GO:0097193	intrinsic apoptotic signaling pathway	85/4924	2.54E-06	1.80E-05
GO:0060324	face development	26/4924	2.55E-06	1.81E-05
GO:0021543	pallium development	52/4924	2.66E-06	1.88E-05
GO:0032414	positive regulation of ion transmembrane transporter activity	43/4924	2.71E-06	1.92E-05
GO:0010633	negative regulation of epithelial cell migration	29/4924	2.77E-06	1.95E-05
GO:0071805	potassium ion transmembrane transport	62/4924	2.79E-06	1.97E-05
GO:0030595	leukocyte chemotaxis	67/4924	2.85E-06	2.01E-05
GO:0051693	actin filament capping	20/4924	2.91E-06	2.05E-05
GO:1900180	regulation of protein localization to nucleus	49/4924	2.97E-06	2.09E-05
GO:0051496	positive regulation of stress fiber assembly	25/4924	3.09E-06	2.17E-05

GO:0007269	neurotransmitter secretion	57/4924	3.10E-06	2.17E-05
GO:0099643	signal release from synapse	57/4924	3.10E-06	2.17E-05
GO:0032651	regulation of interleukin-1 beta production	35/4924	3.17E-06	2.22E-05
GO:0051294	establishment of spindle orientation	19/4924	3.22E-06	2.25E-05
GO:0071634	regulation of transforming growth factor beta production	19/4924	3.22E-06	2.25E-05
GO:0043123	positive regulation of I-kappaB kinase/NF-kappaB signaling	43/4924	3.43E-06	2.39E-05
GO:0043280	positive regulation of cysteine-type endopeptidase activity involved in apoptotic process	43/4924	3.43E-06	2.39E-05
GO:0003143	embryonic heart tube morphogenesis	28/4924	3.44E-06	2.39E-05
GO:0071677	positive regulation of mononuclear cell migration	28/4924	3.44E-06	2.39E-05
GO:0003401	axis elongation	18/4924	3.48E-06	2.41E-05
GO:0021846	cell proliferation in forebrain	18/4924	3.48E-06	2.41E-05
GO:0022404	molting cycle process	41/4924	3.49E-06	2.41E-05
GO:0022405	hair cycle process	41/4924	3.49E-06	2.41E-05
GO:0071459	protein localization to chromosome, centromeric region	15/4924	3.49E-06	2.41E-05
GO:2000050	regulation of non-canonical Wnt signaling pathway	15/4924	3.49E-06	2.41E-05
GO:0071824	protein-DNA complex subunit organization	58/4924	3.53E-06	2.43E-05
GO:0070831	basement membrane assembly	Oct-24	3.64E-06	2.50E-05
GO:0072393	microtubule anchoring at microtubule organizing center	Oct-24	3.64E-06	2.50E-05

GO:0060711	labyrinthine layer development	26/4924	3.68E-06	2.53E-05
GO:0034113	heterotypic cell-cell adhesion	24/4924	3.73E-06	2.56E-05
GO:0006813	potassium ion transport	70/4924	3.77E-06	2.59E-05
GO:0106106	cold-induced thermogenesis	48/4924	3.79E-06	2.60E-05
GO:0003151	outflow tract morphogenesis	33/4924	3.84E-06	2.63E-05
GO:0060997	dendritic spine morphogenesis	29/4924	3.85E-06	2.63E-05
GO:0014902	myotube differentiation	46/4924	4.03E-06	2.75E-05
GO:0017158	regulation of calcium ion-dependent exocytosis	21/4924	4.07E-06	2.77E-05
GO:0035633	maintenance of blood-brain barrier	Dec-24	4.09E-06	2.78E-05
GO:0061430	bone trabecula morphogenesis	Dec-24	4.09E-06	2.78E-05
GO:0000302	response to reactive oxygen species	58/4924	4.23E-06	2.87E-05
GO:0007127	meiosis I	44/4924	4.23E-06	2.87E-05
GO:0061008	hepaticobiliary system development	44/4924	4.23E-06	2.87E-05
GO:0035637	multicellular organismal signaling	50/4924	4.32E-06	2.93E-05
GO:0007173	epidermal growth factor receptor signaling pathway	37/4924	4.38E-06	2.96E-05
GO:1901890	positive regulation of cell junction assembly	42/4924	4.38E-06	2.96E-05
GO:0098773	skin epidermis development	41/4924	4.43E-06	2.99E-05
GO:0001942	hair follicle development	40/4924	4.45E-06	3.01E-05

GO:0030857	negative regulation of epithelial cell differentiation	23/4924	4.47E-06	3.02E-05
GO:0045637	regulation of myeloid cell differentiation	65/4924	4.55E-06	3.07E-05
GO:0043433	negative regulation of DNA-binding transcription factor activity	53/4924	4.59E-06	3.09E-05
GO:1904064	positive regulation of cation transmembrane transport	53/4924	4.59E-06	3.09E-05
GO:0007204	positive regulation of cytosolic calcium ion concentration	94/4924	4.63E-06	3.11E-05
GO:0071174	mitotic spindle checkpoint signaling	20/4924	4.67E-06	3.13E-05
GO:0019722	calcium-mediated signaling	59/4924	4.76E-06	3.19E-05
GO:0051302	regulation of cell division	59/4924	4.76E-06	3.19E-05
GO:0010569	regulation of double-strand break repair via homologous recombination	28/4924	4.80E-06	3.21E-05
GO:0090316	positive regulation of intracellular protein transport	47/4924	4.84E-06	3.23E-05
GO:0032353	negative regulation of hormone biosynthetic process	Sep-24	4.87E-06	3.25E-05
GO:0014032	neural crest cell development	33/4924	5.10E-06	3.40E-05
GO:0006275	regulation of DNA replication	45/4924	5.15E-06	3.43E-05
GO:0050670	regulation of lymphocyte proliferation	70/4924	5.16E-06	3.43E-05
GO:0001505	regulation of neurotransmitter levels	72/4924	5.18E-06	3.44E-05
GO:0001755	neural crest cell migration	26/4924	5.25E-06	3.48E-05
GO:0072678	T cell migration	26/4924	5.25E-06	3.48E-05
GO:2000401	regulation of lymphocyte migration	26/4924	5.25E-06	3.48E-05

GO:0060675	ureteric bud morphogenesis	29/4924	5.30E-06	3.50E-05
GO:0140029	exocytic process	34/4924	5.31E-06	3.51E-05
GO:0001889	liver development	43/4924	5.41E-06	3.57E-05
GO:2000278	regulation of DNA biosynthetic process	43/4924	5.41E-06	3.57E-05
GO:0072577	endothelial cell apoptotic process	24/4924	5.48E-06	3.61E-05
GO:0042176	regulation of protein catabolic process	102/4924	5.54E-06	3.65E-05
GO:0001892	embryonic placenta development	40/4924	5.66E-06	3.72E-05
GO:0032677	regulation of interleukin-8 production	30/4924	5.74E-06	3.77E-05
GO:0061180	mammary gland epithelium development	30/4924	5.74E-06	3.77E-05
GO:2000379	positive regulation of reactive oxygen species metabolic process	30/4924	5.74E-06	3.77E-05
GO:0040001	establishment of mitotic spindle localization	18/4924	5.84E-06	3.82E-05
GO:0030193	regulation of blood coagulation	27/4924	5.97E-06	3.90E-05
GO:0051984	positive regulation of chromosome segregation	14/4924	6.23E-06	4.07E-05
GO:0060122	inner ear receptor cell stereocilium organization	21/4924	6.28E-06	4.09E-05
GO:1903115	regulation of actin filament-based movement	21/4924	6.28E-06	4.09E-05
GO:0048841	regulation of axon extension involved in axon guidance	17/4924	6.31E-06	4.11E-05
GO:0060669	embryonic placenta morphogenesis	17/4924	6.31E-06	4.11E-05
GO:0051048	negative regulation of secretion	60/4924	6.34E-06	4.12E-05

GO:0002821	positive regulation of adaptive immune response	50/4924	6.44E-06	4.18E-05
GO:0034508	centromere complex assembly	16/4924	6.60E-06	4.28E-05
GO:0034453	microtubule anchoring	15/4924	6.61E-06	4.28E-05
GO:0002042	cell migration involved in sprouting angiogenesis	23/4924	6.63E-06	4.28E-05
GO:0043370	regulation of CD4-positive, alpha-beta T cell differentiation	23/4924	6.63E-06	4.28E-05
GO:2000351	regulation of endothelial cell apoptotic process	23/4924	6.63E-06	4.28E-05
GO:0050818	regulation of coagulation	28/4924	6.63E-06	4.28E-05
GO:0038065	collagen-activated signaling pathway	Nov-24	6.73E-06	4.33E-05
GO:0048103	somatic stem cell division	Nov-24	6.73E-06	4.33E-05
GO:0002088	lens development in camera-type eye	33/4924	6.74E-06	4.33E-05
GO:0008593	regulation of Notch signaling pathway	33/4924	6.74E-06	4.33E-05
GO:0006869	lipid transport	101/4924	6.80E-06	4.36E-05
GO:0015833	peptide transport	86/4924	6.96E-06	4.47E-05
GO:0018205	peptidyl-lysine modification	102/4924	7.01E-06	4.49E-05
GO:0031497	chromatin assembly	52/4924	7.08E-06	4.53E-05
GO:0032091	negative regulation of protein binding	35/4924	7.11E-06	4.55E-05
GO:0038061	NIK/NF-kappaB signaling	40/4924	7.16E-06	4.57E-05
GO:0060078	regulation of postsynaptic membrane potential	40/4924	7.16E-06	4.57E-05

GO:0010657	muscle cell apoptotic process	36/4924	7.21E-06	4.59E-05
GO:0042116	macrophage activation	36/4924	7.21E-06	4.59E-05
GO:0051149	positive regulation of muscle cell differentiation	29/4924	7.23E-06	4.60E-05
GO:0072171	mesonephric tubule morphogenesis	29/4924	7.23E-06	4.60E-05
GO:0045880	positive regulation of smoothened signaling pathway	20/4924	7.33E-06	4.65E-05
GO:1904705	regulation of vascular associated smooth muscle cell proliferation	26/4924	7.42E-06	4.70E-05
GO:0006282	regulation of DNA repair	51/4924	7.45E-06	4.72E-05
GO:0034614	cellular response to reactive oxygen species	46/4924	7.62E-06	4.82E-05
GO:0050729	positive regulation of inflammatory response	46/4924	7.62E-06	4.82E-05
GO:0002221	pattern recognition receptor signaling pathway	54/4924	7.65E-06	4.83E-05
GO:0032637	interleukin-8 production	30/4924	7.76E-06	4.90E-05
GO:0046883	regulation of hormone secretion	83/4924	7.81E-06	4.92E-05
GO:0010463	mesenchymal cell proliferation	24/4924	7.93E-06	4.99E-05
GO:0086001	cardiac muscle cell action potential	24/4924	7.93E-06	4.99E-05
GO:0046651	lymphocyte proliferation	89/4924	7.99E-06	5.02E-05
GO:0070373	negative regulation of ERK1 and ERK2 cascade	31/4924	8.20E-06	5.15E-05
GO:0043491	protein kinase B signaling	61/4924	8.31E-06	5.21E-05
GO:0010876	lipid localization	113/4924	8.34E-06	5.23E-05

GO:1902893	regulation of miRNA transcription	32/4924	8.57E-06	5.36E-05
GO:0034612	response to tumor necrosis factor	60/4924	8.89E-06	5.56E-05
GO:0070665	positive regulation of leukocyte proliferation	51/4924	9.01E-06	5.63E-05
GO:0045666	positive regulation of neuron differentiation	40/4924	9.02E-06	5.63E-05
GO:0033077	T cell differentiation in thymus	34/4924	9.07E-06	5.65E-05
GO:0007091	metaphase/anaphase transition of mitotic cell cycle	25/4924	9.19E-06	5.72E-05
GO:0034766	negative regulation of ion transmembrane transport	37/4924	9.29E-06	5.78E-05
GO:0040037	negative regulation of fibroblast growth factor receptor signaling pathway	Dec-24	9.36E-06	5.81E-05
GO:0090660	cerebrospinal fluid circulation	Dec-24	9.36E-06	5.81E-05
GO:1903524	positive regulation of blood circulation	21/4924	9.53E-06	5.91E-05
GO:0022617	extracellular matrix disassembly	18/4924	9.56E-06	5.92E-05
GO:0070306	lens fiber cell differentiation	18/4924	9.56E-06	5.92E-05
GO:1905508	protein localization to microtubule organizing center	18/4924	9.56E-06	5.92E-05
GO:0045604	regulation of epidermal cell differentiation	23/4924	9.69E-06	5.98E-05
GO:0045773	positive regulation of axon extension	23/4924	9.69E-06	5.98E-05
GO:0099504	synaptic vesicle cycle	65/4924	1.02E-05	6.26E-05
GO:0002699	positive regulation of immune effector process	83/4924	1.02E-05	6.27E-05
GO:0002244	hematopoietic progenitor cell differentiation	52/4924	1.03E-05	6.33E-05

GO:0032615	interleukin-12 production	26/4924	1.04E-05	6.37E-05
GO:0010714	positive regulation of collagen metabolic process	17/4924	1.06E-05	6.52E-05
GO:0010457	centriole-centriole cohesion	Oct-24	1.08E-05	6.61E-05
GO:2000052	positive regulation of non-canonical Wnt signaling pathway	Oct-24	1.08E-05	6.61E-05
GO:0008088	axo-dendritic transport	31/4924	1.09E-05	6.67E-05
GO:0009411	response to UV	47/4924	1.10E-05	6.70E-05
GO:0065004	protein-DNA complex assembly	47/4924	1.10E-05	6.70E-05
GO:0042472	inner ear morphogenesis	40/4924	1.13E-05	6.91E-05
GO:0033045	regulation of sister chromatid segregation	27/4924	1.14E-05	6.95E-05
GO:0051926	negative regulation of calcium ion transport	27/4924	1.14E-05	6.95E-05
GO:0150116	regulation of cell-substrate junction organization	27/4924	1.14E-05	6.95E-05
GO:1900046	regulation of hemostasis	27/4924	1.14E-05	6.95E-05
GO:0032967	positive regulation of collagen biosynthetic process	16/4924	1.15E-05	6.97E-05
GO:0086010	membrane depolarization during action potential	16/4924	1.15E-05	6.97E-05
GO:0035710	CD4-positive, alpha-beta T cell activation	39/4924	1.15E-05	7.00E-05
GO:0060079	excitatory postsynaptic potential	33/4924	1.16E-05	7.00E-05
GO:1900182	positive regulation of protein localization to nucleus	33/4924	1.16E-05	7.00E-05
GO:0070509	calcium ion import	34/4924	1.17E-05	7.11E-05

GO:0008038	neuron recognition	22/4924	1.18E-05	7.12E-05
GO:0009798	axis specification	35/4924	1.19E-05	7.16E-05
GO:0051153	regulation of striated muscle cell differentiation	36/4924	1.19E-05	7.16E-05
GO:0060445	branching involved in salivary gland morphogenesis	15/4924	1.20E-05	7.21E-05
GO:1903055	positive regulation of extracellular matrix organization	15/4924	1.20E-05	7.21E-05
GO:0032732	positive regulation of interleukin-1 production	28/4924	1.24E-05	7.44E-05
GO:0050773	regulation of dendrite development	44/4924	1.24E-05	7.47E-05
GO:0010965	regulation of mitotic sister chromatid separation	25/4924	1.29E-05	7.74E-05
GO:1903078	positive regulation of protein localization to plasma membrane	25/4924	1.29E-05	7.74E-05
GO:0006029	proteoglycan metabolic process	29/4924	1.32E-05	7.88E-05
GO:0043113	receptor clustering	29/4924	1.32E-05	7.88E-05
GO:2001022	positive regulation of response to DNA damage stimulus	47/4924	1.33E-05	7.98E-05
GO:0030534	adult behavior	56/4924	1.37E-05	8.19E-05
GO:0002702	positive regulation of production of molecular mediator of immune response	41/4924	1.38E-05	8.21E-05
GO:0003208	cardiac ventricle morphogenesis	30/4924	1.38E-05	8.25E-05
GO:0048706	embryonic skeletal system development	46/4924	1.40E-05	8.33E-05
GO:0010799	regulation of peptidyl-threonine phosphorylation	21/4924	1.42E-05	8.46E-05
GO:0002720	positive regulation of cytokine production involved in immune response	26/4924	1.43E-05	8.52E-05

GO:0034103	regulation of tissue remodeling	31/4924	1.43E-05	8.53E-05
GO:1903305	regulation of regulated secretory pathway	55/4924	1.46E-05	8.68E-05
GO:0061614	miRNA transcription	32/4924	1.47E-05	8.74E-05
GO:0008630	intrinsic apoptotic signaling pathway in response to DNA damage	38/4924	1.48E-05	8.74E-05
GO:0030168	platelet activation	33/4924	1.50E-05	8.88E-05
GO:0001953	negative regulation of cell-matrix adhesion	18/4924	1.53E-05	9.03E-05
GO:2000514	regulation of CD4-positive, alpha-beta T cell activation	27/4924	1.56E-05	9.20E-05
GO:2001257	regulation of cation channel activity	54/4924	1.56E-05	9.20E-05
GO:0003184	pulmonary valve morphogenesis	Nov-24	1.61E-05	9.49E-05
GO:0051382	kinetochore assembly	Nov-24	1.61E-05	9.49E-05
GO:0032351	negative regulation of hormone metabolic process	Sep-24	1.65E-05	9.69E-05
GO:0038063	collagen-activated tyrosine kinase receptor signaling pathway	Sep-24	1.65E-05	9.69E-05
GO:0072697	protein localization to cell cortex	Sep-24	1.65E-05	9.69E-05
GO:0031577	spindle checkpoint signaling	20/4924	1.70E-05	9.99E-05
GO:0086009	membrane repolarization	20/4924	1.70E-05	9.99E-05
GO:0002688	regulation of leukocyte chemotaxis	41/4924	1.70E-05	9.99E-05
GO:0060428	lung epithelium development	22/4924	1.71E-05	0.000100237
GO:0009952	anterior/posterior pattern specification	66/4924	1.73E-05	0.000101246

GO:0008608	attachment of spindle microtubules to kinetochore	17/4924	1.73E-05	0.000101287
GO:0071539	protein localization to centrosome	17/4924	1.73E-05	0.000101287
GO:0010769	regulation of cell morphogenesis involved in differentiation	40/4924	1.76E-05	0.000102491
GO:0060419	heart growth	40/4924	1.76E-05	0.000102491
GO:0042471	ear morphogenesis	45/4924	1.78E-05	0.000103804
GO:0048260	positive regulation of receptor-mediated endocytosis	25/4924	1.79E-05	0.000104355
GO:0014015	positive regulation of gliogenesis	30/4924	1.83E-05	0.000106101
GO:0010827	regulation of glucose transmembrane transport	32/4924	1.91E-05	0.000111152
GO:0045185	maintenance of protein location	35/4924	1.93E-05	0.000112016
GO:0048525	negative regulation of viral process	35/4924	1.93E-05	0.000112016
GO:0042475	odontogenesis of dentin-containing tooth	33/4924	1.93E-05	0.000112016
GO:1904063	negative regulation of cation transmembrane transport	34/4924	1.94E-05	0.000112258
GO:0002824	positive regulation of adaptive immune response based on somatic recombination of immune receptors built from immunoglobulin superfamily domains	47/4924	1.96E-05	0.000112988
GO:0015698	inorganic anion transport	47/4924	1.96E-05	0.000112988
GO:0046660	female sex differentiation	47/4924	1.96E-05	0.000112988
GO:0043903	regulation of biological process involved in symbiotic interaction	26/4924	1.96E-05	0.000113008
GO:1990874	vascular associated smooth muscle cell proliferation	26/4924	1.96E-05	0.000113008
GO:0002031	G protein-coupled receptor internalization	Dec-24	1.97E-05	0.000113483

GO:0072010	glomerular epithelium development	Dec-24	1.97E-05	0.000113483
GO:0072593	reactive oxygen species metabolic process	68/4924	1.98E-05	0.000113957
GO:0001774	microglial cell activation	19/4924	2.03E-05	0.000115943
GO:0006904	vesicle docking involved in exocytosis	19/4924	2.03E-05	0.000115943
GO:0007143	female meiotic nuclear division	19/4924	2.03E-05	0.000115943
GO:0045841	negative regulation of mitotic metaphase/anaphase transition	19/4924	2.03E-05	0.000115943
GO:2001239	regulation of extrinsic apoptotic signaling pathway in absence of ligand	21/4924	2.09E-05	0.000119397
GO:0021885	forebrain cell migration	27/4924	2.11E-05	0.000120359
GO:0045165	cell fate commitment	77/4924	2.18E-05	0.000124076
GO:0030859	polarized epithelial cell differentiation	14/4924	2.18E-05	0.000124076
GO:0014068	positive regulation of phosphatidylinositol 3-kinase signaling	28/4924	2.23E-05	0.000126766
GO:0045669	positive regulation of osteoblast differentiation	28/4924	2.23E-05	0.000126766
GO:0048278	vesicle docking	24/4924	2.24E-05	0.000127269
GO:0060632	regulation of microtubule-based movement	24/4924	2.24E-05	0.000127269
GO:2001238	positive regulation of extrinsic apoptotic signaling pathway	24/4924	2.24E-05	0.000127269
GO:0030316	osteoclast differentiation	39/4924	2.25E-05	0.000127566
GO:0035270	endocrine system development	44/4924	2.27E-05	0.00012886
GO:0002634	regulation of germinal center formation	Aug-24	2.35E-05	0.000132606

GO:1902746	regulation of lens fiber cell differentiation	Aug-24	2.35E-05	0.000132606
GO:0008625	extrinsic apoptotic signaling pathway via death domain receptors	30/4924	2.40E-05	0.000135314
GO:0044784	metaphase/anaphase transition of cell cycle	25/4924	2.47E-05	0.000139357
GO:0002690	positive regulation of leukocyte chemotaxis	34/4924	2.47E-05	0.00013954
GO:0032946	positive regulation of mononuclear cell proliferation	46/4924	2.49E-05	0.000140551
GO:0007626	locomotory behavior	72/4924	2.52E-05	0.000141672
GO:0002269	leukocyte activation involved in inflammatory response	20/4924	2.53E-05	0.000142227
GO:0033047	regulation of mitotic sister chromatid segregation	20/4924	2.53E-05	0.000142227
GO:0051985	negative regulation of chromosome segregation	20/4924	2.53E-05	0.000142227
GO:0015748	organophosphate ester transport	40/4924	2.69E-05	0.00015101
GO:0098656	anion transmembrane transport	58/4924	2.71E-05	0.00015166
GO:0002790	peptide secretion	79/4924	2.71E-05	0.00015166
GO:0007076	mitotic chromosome condensation	Oct-24	2.73E-05	0.000152911
GO:0120033	negative regulation of plasma membrane bounded cell projection assembly	17/4924	2.76E-05	0.000154529
GO:0051489	regulation of filopodium assembly	23/4924	2.80E-05	0.000156207
GO:2000179	positive regulation of neural precursor cell proliferation	27/4924	2.83E-05	0.000157698
GO:0036293	response to decreased oxygen levels	67/4924	2.86E-05	0.000159307
GO:0031570	DNA integrity checkpoint signaling	38/4924	2.88E-05	0.000160333

GO:0140056	organelle localization by membrane tethering	28/4924	2.96E-05	0.000164488
GO:0055017	cardiac muscle tissue growth	37/4924	2.96E-05	0.000164621
GO:0003254	regulation of membrane depolarization	21/4924	3.02E-05	0.000167816
GO:0045214	sarcomere organization	21/4924	3.02E-05	0.000167816
GO:0030501	positive regulation of bone mineralization	19/4924	3.05E-05	0.000168579
GO:0033046	negative regulation of sister chromatid segregation	19/4924	3.05E-05	0.000168579
GO:0033048	negative regulation of mitotic sister chromatid segregation	19/4924	3.05E-05	0.000168579
GO:0045823	positive regulation of heart contraction	19/4924	3.05E-05	0.000168579
GO:2000816	negative regulation of mitotic sister chromatid separation	19/4924	3.05E-05	0.000168579
GO:0071356	cellular response to tumor necrosis factor	54/4924	3.06E-05	0.000169038
GO:0010812	negative regulation of cell-substrate adhesion	24/4924	3.10E-05	0.000170926
GO:0032731	positive regulation of interleukin-1 beta production	24/4924	3.10E-05	0.000170926
GO:0002063	chondrocyte development	16/4924	3.15E-05	0.000173169
GO:0033687	osteoblast proliferation	16/4924	3.15E-05	0.000173169
GO:1905314	semi-lunar valve development	16/4924	3.15E-05	0.000173169
GO:2000108	positive regulation of leukocyte apoptotic process	16/4924	3.15E-05	0.000173169
GO:0021675	nerve development	31/4924	3.16E-05	0.000173435
GO:0097581	lamellipodium organization	32/4924	3.17E-05	0.000174027

GO:2000045	regulation of G1/S transition of mitotic cell cycle	45/4924	3.18E-05	0.000174027
GO:0030072	peptide hormone secretion	77/4924	3.21E-05	0.000175418
GO:0032649	regulation of interferon-gamma production	40/4924	3.31E-05	0.000181097
GO:0001954	positive regulation of cell-matrix adhesion	25/4924	3.36E-05	0.000183503
GO:0051306	mitotic sister chromatid separation	25/4924	3.36E-05	0.000183503
GO:0060119	inner ear receptor cell development	25/4924	3.36E-05	0.000183503
GO:0006023	aminoglycan biosynthetic process	22/4924	3.48E-05	0.000189631
GO:0036159	inner dynein arm assembly	Nov-24	3.50E-05	0.000190501
GO:0002029	desensitization of G protein-coupled receptor signaling pathway	15/4924	3.52E-05	0.000191
GO:0006929	substrate-dependent cell migration	15/4924	3.52E-05	0.000191
GO:0022401	negative adaptation of signaling pathway	15/4924	3.52E-05	0.000191
GO:0032609	interferon-gamma production	43/4924	3.53E-05	0.000191308
GO:0007094	mitotic spindle assembly checkpoint signaling	18/4924	3.64E-05	0.000196951
GO:0034110	regulation of homotypic cell-cell adhesion	18/4924	3.64E-05	0.000196951
GO:0060325	face morphogenesis	18/4924	3.64E-05	0.000196951
GO:0071173	spindle assembly checkpoint signaling	18/4924	3.64E-05	0.000196951
GO:0050821	protein stabilization	56/4924	3.66E-05	0.000198008
GO:0035296	regulation of tube diameter	51/4924	3.76E-05	0.000202435

GO:0097746	blood vessel diameter maintenance	51/4924	3.76E-05	0.000202435
GO:0008542	visual learning	27/4924	3.76E-05	0.000202435
GO:0043506	regulation of JUN kinase activity	27/4924	3.76E-05	0.000202435
GO:0030204	chondroitin sulfate metabolic process	14/4924	3.81E-05	0.000203871
GO:0031954	positive regulation of protein autophosphorylation	14/4924	3.81E-05	0.000203871
GO:0032528	microvillus organization	14/4924	3.81E-05	0.000203871
GO:0048843	negative regulation of axon extension involved in axon guidance	14/4924	3.81E-05	0.000203871
GO:0060004	reflex	14/4924	3.81E-05	0.000203871
GO:0072012	glomerulus vasculature development	14/4924	3.81E-05	0.000203871
GO:2000316	regulation of T-helper 17 type immune response	14/4924	3.81E-05	0.000203871
GO:2000377	regulation of reactive oxygen species metabolic process	48/4924	3.84E-05	0.000205226
GO:0003177	pulmonary valve development	Dec-24	3.89E-05	0.000207296
GO:0045606	positive regulation of epidermal cell differentiation	Dec-24	3.89E-05	0.000207296
GO:0010761	fibroblast migration	23/4924	3.89E-05	0.000207296
GO:0030071	regulation of mitotic metaphase/anaphase transition	23/4924	3.89E-05	0.000207296
GO:0061097	regulation of protein tyrosine kinase activity	28/4924	3.89E-05	0.000207296
GO:0042886	amide transport	90/4924	3.93E-05	0.000208856
GO:0060021	roof of mouth development	34/4924	3.96E-05	0.000210248

GO:0010842	retina layer formation	13/4924	3.96E-05	0.000210248
GO:0099625	ventricular cardiac muscle cell membrane repolarization	13/4924	3.96E-05	0.000210248
GO:0003407	neural retina development	29/4924	3.99E-05	0.000211338
GO:0070555	response to interleukin-1	33/4924	4.02E-05	0.000212922
GO:0044728	DNA methylation or demethylation	32/4924	4.05E-05	0.000214598
GO:0045621	positive regulation of lymphocyte differentiation	39/4924	4.23E-05	0.000223925
GO:0001947	heart looping	24/4924	4.24E-05	0.000224345
GO:0051588	regulation of neurotransmitter transport	43/4924	4.27E-05	0.000225449
GO:0007435	salivary gland morphogenesis	17/4924	4.30E-05	0.000226598
GO:0035909	aorta morphogenesis	17/4924	4.30E-05	0.000226598
GO:0051647	nucleus localization	17/4924	4.30E-05	0.000226598
GO:0045933	positive regulation of muscle contraction	21/4924	4.32E-05	0.000226778
GO:0090329	regulation of DNA-templated DNA replication	21/4924	4.32E-05	0.000226778
GO:1904407	positive regulation of nitric oxide metabolic process	21/4924	4.32E-05	0.000226778
GO:0035150	regulation of tube size	51/4924	4.44E-05	0.000233092
GO:0031349	positive regulation of defense response	79/4924	4.45E-05	0.000233566
GO:0010464	regulation of mesenchymal cell proliferation	19/4924	4.51E-05	0.00023609
GO:0032508	DNA duplex unwinding	19/4924	4.51E-05	0.00023609

GO:1902100	negative regulation of metaphase/anaphase transition of cell cycle	19/4924	4.51E-05	0.00023609
GO:2000241	regulation of reproductive process	59/4924	4.63E-05	0.00024227
GO:0060401	cytosolic calcium ion transport	57/4924	4.64E-05	0.000242472
GO:0046822	regulation of nucleocytoplasmic transport	41/4924	4.73E-05	0.000246689
GO:0050885	neuromuscular process controlling balance	26/4924	4.78E-05	0.000249335
GO:0010712	regulation of collagen metabolic process	22/4924	4.87E-05	0.000253687
GO:0051101	regulation of DNA binding	40/4924	4.96E-05	0.000257944
GO:0001885	endothelial cell development	27/4924	4.96E-05	0.000257944
GO:0045911	positive regulation of DNA recombination	27/4924	4.96E-05	0.000257944
GO:0048013	ephrin receptor signaling pathway	16/4924	5.01E-05	0.000260112
GO:0051497	negative regulation of stress fiber assembly	16/4924	5.01E-05	0.000260112
GO:0071383	cellular response to steroid hormone stimulus	49/4924	5.08E-05	0.000263513
GO:0006970	response to osmotic stress	32/4924	5.15E-05	0.000266673
GO:0044774	mitotic DNA integrity checkpoint signaling	29/4924	5.17E-05	0.000267037
GO:0045123	cellular extravasation	29/4924	5.17E-05	0.000267037
GO:0060042	retina morphogenesis in camera-type eye	29/4924	5.17E-05	0.000267037
GO:1901379	regulation of potassium ion transmembrane transport	31/4924	5.19E-05	0.000268031
GO:0010921	regulation of phosphatase activity	30/4924	5.20E-05	0.000268031

GO:0060415	muscle tissue morphogenesis	30/4924	5.20E-05	0.000268031
GO:0001941	postsynaptic membrane organization	20/4924	5.33E-05	0.000274321
GO:0032965	regulation of collagen biosynthetic process	20/4924	5.33E-05	0.000274321
GO:0033628	regulation of cell adhesion mediated by integrin	20/4924	5.33E-05	0.000274321
GO:0031647	regulation of protein stability	81/4924	5.38E-05	0.000276347
GO:0048709	oligodendrocyte differentiation	38/4924	5.41E-05	0.000277913
GO:0007431	salivary gland development	18/4924	5.46E-05	0.000279601
GO:0035850	epithelial cell differentiation involved in kidney development	18/4924	5.46E-05	0.000279601
GO:2000463	positive regulation of excitatory postsynaptic potential	18/4924	5.46E-05	0.000279601
GO:0055007	cardiac muscle cell differentiation	45/4924	5.51E-05	0.000282013
GO:0001945	lymph vessel development	15/4924	5.74E-05	0.000292986
GO:0007097	nuclear migration	15/4924	5.74E-05	0.000292986
GO:0048535	lymph node development	15/4924	5.74E-05	0.000292986
GO:0032655	regulation of interleukin-12 production	24/4924	5.75E-05	0.000293474
GO:0045667	regulation of osteoblast differentiation	47/4924	5.80E-05	0.000295437
GO:0008344	adult locomotory behavior	36/4924	5.84E-05	0.000297544
GO:0097529	myeloid leukocyte migration	63/4924	6.01E-05	0.000305615
GO:0016573	histone acetylation	49/4924	6.01E-05	0.000305725

GO:0001658	branching involved in ureteric bud morphogenesis	25/4924	6.08E-05	0.000308407
GO:0055081	anion homeostasis	21/4924	6.08E-05	0.000308407
GO:1902041	regulation of extrinsic apoptotic signaling pathway via death domain receptors	21/4924	6.08E-05	0.000308407
GO:0002467	germinal center formation	Oct-24	6.17E-05	0.000312572
GO:0034121	regulation of toll-like receptor signaling pathway	26/4924	6.33E-05	0.00031984
GO:0042733	embryonic digit morphogenesis	26/4924	6.33E-05	0.00031984
GO:1902895	positive regulation of miRNA transcription	26/4924	6.33E-05	0.00031984
GO:0060713	labyrinthine layer morphogenesis	14/4924	6.42E-05	0.000323592
GO:0061437	renal system vasculature development	14/4924	6.42E-05	0.000323592
GO:0061440	kidney vasculature development	14/4924	6.42E-05	0.000323592
GO:2000178	negative regulation of neural precursor cell proliferation	14/4924	6.42E-05	0.000323592
GO:0060236	regulation of mitotic spindle organization	17/4924	6.55E-05	0.000329484
GO:0086004	regulation of cardiac muscle cell contraction	17/4924	6.55E-05	0.000329484
GO:0042554	superoxide anion generation	19/4924	6.56E-05	0.000329484
GO:1902742	apoptotic process involved in development	19/4924	6.56E-05	0.000329484
GO:1905819	negative regulation of chromosome separation	19/4924	6.56E-05	0.000329484
GO:0035418	protein localization to synapse	30/4924	6.65E-05	0.000333507
GO:0043507	positive regulation of JUN kinase activity	22/4924	6.73E-05	0.000337407

GO:0045861	negative regulation of proteolysis	90/4924	6.82E-05	0.000341258
GO:1903320	regulation of protein modification by small protein conjugation or removal	68/4924	6.82E-05	0.000341258
GO:0055083	monovalent inorganic anion homeostasis	13/4924	6.96E-05	0.000347796
GO:0150105	protein localization to cell-cell junction	13/4924	6.96E-05	0.000347796
GO:0022038	corpus callosum development	Nov-24	7.02E-05	0.000349753
GO:0035313	wound healing, spreading of epidermal cells	Nov-24	7.02E-05	0.000349753
GO:0045198	establishment of epithelial cell apical/basal polarity	Nov-24	7.02E-05	0.000349753
GO:1900452	regulation of long-term synaptic depression	Nov-24	7.02E-05	0.000349753
GO:0045217	cell-cell junction maintenance	Dec-24	7.22E-05	0.000358572
GO:0060307	regulation of ventricular cardiac muscle cell membrane repolarization	Dec-24	7.22E-05	0.000358572
GO:0061081	positive regulation of myeloid leukocyte cytokine production involved in immune response	Dec-24	7.22E-05	0.000358572
GO:0072111	cell proliferation involved in kidney development	Dec-24	7.22E-05	0.000358572
GO:0071478	cellular response to radiation	51/4924	7.24E-05	0.000358727
GO:2001242	regulation of intrinsic apoptotic signaling pathway	51/4924	7.24E-05	0.000358727
GO:0032757	positive regulation of interleukin-8 production	23/4924	7.28E-05	0.000360044
GO:0050922	negative regulation of chemotaxis	23/4924	7.28E-05	0.000360044
GO:0060443	mammary gland morphogenesis	23/4924	7.28E-05	0.000360044
GO:0034454	microtubule anchoring at centrosome	Aug-24	7.30E-05	0.000360145

GO:0060287	epithelial cilium movement involved in determination of left/right asymmetry	Aug-24	7.30E-05	0.000360145
GO:0072102	glomerulus morphogenesis	Aug-24	7.30E-05	0.000360145
GO:0016601	Rac protein signal transduction	20/4924	7.57E-05	0.000372707
GO:0045429	positive regulation of nitric oxide biosynthetic process	20/4924	7.57E-05	0.000372707
GO:0061900	glial cell activation	20/4924	7.57E-05	0.000372707
GO:0070167	regulation of biomineral tissue development	34/4924	7.73E-05	0.000380408
GO:0007020	microtubule nucleation	16/4924	7.77E-05	0.000381365
GO:1901099	negative regulation of signal transduction in absence of ligand	16/4924	7.77E-05	0.000381365
GO:2001240	negative regulation of extrinsic apoptotic signaling pathway in absence of ligand	16/4924	7.77E-05	0.000381365
GO:0002761	regulation of myeloid leukocyte differentiation	42/4924	7.89E-05	0.000387015
GO:0090224	regulation of spindle organization	18/4924	8.03E-05	0.000392943
GO:0110110	positive regulation of animal organ morphogenesis	18/4924	8.03E-05	0.000392943
GO:1900744	regulation of p38MAPK cascade	18/4924	8.03E-05	0.000392943
GO:0006801	superoxide metabolic process	25/4924	8.06E-05	0.000393163
GO:0007585	respiratory gaseous exchange by respiratory system	25/4924	8.06E-05	0.000393163
GO:0022029	telencephalon cell migration	25/4924	8.06E-05	0.000393163
GO:0061371	determination of heart left/right asymmetry	25/4924	8.06E-05	0.000393163
GO:0002287	alpha-beta T cell activation involved in immune response	26/4924	8.30E-05	0.000404287

GO:0043967	histone H4 acetylation	26/4924	8.30E-05	0.000404287
GO:0050671	positive regulation of lymphocyte proliferation	44/4924	8.36E-05	0.00040585
GO:0060759	regulation of response to cytokine stimulus	44/4924	8.36E-05	0.00040585
GO:0010212	response to ionizing radiation	41/4924	8.36E-05	0.00040585
GO:0090090	negative regulation of canonical Wnt signaling pathway	41/4924	8.36E-05	0.00040585
GO:0003281	ventricular septum development	30/4924	8.45E-05	0.000407699
GO:0031397	negative regulation of protein ubiquitination	30/4924	8.45E-05	0.000407699
GO:1901184	regulation of ERBB signaling pathway	27/4924	8.45E-05	0.000407699
GO:1903036	positive regulation of response to wounding	27/4924	8.45E-05	0.000407699
GO:0021795	cerebral cortex cell migration	21/4924	8.46E-05	0.000407699
GO:0038066	p38MAPK cascade	21/4924	8.46E-05	0.000407699
GO:0070169	positive regulation of biomineral tissue development	21/4924	8.46E-05	0.000407699
GO:0072091	regulation of stem cell proliferation	21/4924	8.46E-05	0.000407699
GO:1904036	negative regulation of epithelial cell apoptotic process	21/4924	8.46E-05	0.000407699
GO:1904659	glucose transmembrane transport	37/4924	8.47E-05	0.000408156
GO:0002260	lymphocyte homeostasis	29/4924	8.52E-05	0.000409697
GO:0051965	positive regulation of synapse assembly	28/4924	8.52E-05	0.000409697
GO:0042129	regulation of T cell proliferation	53/4924	8.53E-05	0.000409697

GO:0051053	negative regulation of DNA metabolic process	46/4924	8.72E-05	0.000418535
GO:0000132	establishment of mitotic spindle orientation	15/4924	9.10E-05	0.000434967
GO:0023058	adaptation of signaling pathway	15/4924	9.10E-05	0.000434967
GO:0048668	collateral sprouting	15/4924	9.10E-05	0.000434967
GO:0051150	regulation of smooth muscle cell differentiation	15/4924	9.10E-05	0.000434967
GO:0060390	regulation of SMAD protein signal transduction	15/4924	9.10E-05	0.000434967
GO:0098703	calcium ion import across plasma membrane	15/4924	9.10E-05	0.000434967
GO:0030514	negative regulation of BMP signaling pathway	22/4924	9.21E-05	0.000439369
GO:0031529	ruffle organization	22/4924	9.21E-05	0.000439369
GO:0002065	columnar/cuboidal epithelial cell differentiation	35/4924	9.22E-05	0.000439959
GO:0030279	negative regulation of ossification	19/4924	9.40E-05	0.000446946
GO:0031952	regulation of protein autophosphorylation	19/4924	9.40E-05	0.000446946
GO:0048536	spleen development	19/4924	9.40E-05	0.000446946
GO:0060323	head morphogenesis	19/4924	9.40E-05	0.000446946
GO:0009615	response to virus	85/4924	9.45E-05	0.000448765
GO:0034341	response to interferon-gamma	42/4924	9.46E-05	0.000449282
GO:0070570	regulation of neuron projection regeneration	17/4924	9.77E-05	0.000463049
GO:2000036	regulation of stem cell population maintenance	17/4924	9.77E-05	0.000463049

GO:0045682	regulation of epidermis development	23/4924	9.81E-05	0.000464379
GO:1902099	regulation of metaphase/anaphase transition of cell cycle	23/4924	9.81E-05	0.000464379
GO:0032465	regulation of cytokinesis	33/4924	9.91E-05	0.000468466
GO:0060402	calcium ion transport into cytosol	51/4924	9.91E-05	0.000468466
GO:0030522	intracellular receptor signaling pathway	60/4924	0.000101139	0.000477308
GO:0031396	regulation of protein ubiquitination	60/4924	0.000101139	0.000477308
GO:0010660	regulation of muscle cell apoptotic process	32/4924	0.000102115	0.000481553
GO:0008645	hexose transmembrane transport	37/4924	0.000103335	0.000486577
GO:0043401	steroid hormone mediated signaling pathway	37/4924	0.000103335	0.000486577
GO:0099072	regulation of postsynaptic membrane neurotransmitter receptor levels	31/4924	0.000104733	0.000492791
GO:0050819	negative regulation of coagulation	20/4924	0.000106049	0.000496483
GO:0090102	cochlea development	20/4924	0.000106049	0.000496483
GO:0006940	regulation of smooth muscle contraction	25/4924	0.000106076	0.000496483
GO:0042093	T-helper cell differentiation	25/4924	0.000106076	0.000496483
GO:0046513	ceramide biosynthetic process	25/4924	0.000106076	0.000496483
GO:0006665	sphingolipid metabolic process	43/4924	0.000106147	0.000496483
GO:0050728	negative regulation of inflammatory response	43/4924	0.000106147	0.000496483
GO:0098876	vesicle-mediated transport to the plasma membrane	43/4924	0.000106147	0.000496483

GO:0016081	synaptic vesicle docking	Sep-24	0.000107815	0.000502421
GO:0048755	branching morphogenesis of a nerve	Sep-24	0.000107815	0.000502421
GO:0051764	actin crosslink formation	Sep-24	0.000107815	0.000502421
GO:0090179	planar cell polarity pathway involved in neural tube closure	Sep-24	0.000107815	0.000502421
GO:2000105	positive regulation of DNA-templated DNA replication	Sep-24	0.000107815	0.000502421
GO:0000079	regulation of cyclin-dependent protein serine/threonine kinase activity	26/4924	0.000108175	0.000503352
GO:0046637	regulation of alpha-beta T cell differentiation	26/4924	0.000108175	0.000503352
GO:0030500	regulation of bone mineralization	29/4924	0.000108389	0.000503976
GO:0007632	visual behavior	27/4924	0.000109174	0.000506263
GO:0022406	membrane docking	28/4924	0.000109202	0.000506263
GO:0050709	negative regulation of protein secretion	28/4924	0.000109202	0.000506263
GO:1902807	negative regulation of cell cycle G1/S phase transition	28/4924	0.000109202	0.000506263
GO:0030216	keratinocyte differentiation	45/4924	0.000110398	0.000511432
GO:0050792	regulation of viral process	49/4924	0.000115011	0.000532411
GO:0010765	positive regulation of sodium ion transport	18/4924	0.000116208	0.000536999
GO:0070527	platelet aggregation	21/4924	0.000116257	0.000536999
GO:0110151	positive regulation of biomineralization	21/4924	0.000116257	0.000536999
GO:0099623	regulation of cardiac muscle cell membrane repolarization	13/4924	0.000117464	0.000542174

GO:0048679	regulation of axon regeneration	16/4924	0.000117788	0.000542873
GO:1904037	positive regulation of epithelial cell apoptotic process	16/4924	0.000117788	0.000542873
GO:0045582	positive regulation of T cell differentiation	34/4924	0.000118105	0.000543542
GO:0110149	regulation of biomineralization	34/4924	0.000118105	0.000543542
GO:0043543	protein acylation	69/4924	0.000118253	0.000543826
GO:0006476	protein deacetylation	38/4924	0.000119081	0.000547232
GO:0034504	protein localization to nucleus	81/4924	0.000119801	0.000550143
GO:0009791	post-embryonic development	41/4924	0.000120352	0.000552268
GO:0008643	carbohydrate transport	46/4924	0.000121632	0.000557736
GO:0043502	regulation of muscle adaptation	33/4924	0.000122714	0.000562289
GO:0051896	regulation of protein kinase B signaling	50/4924	0.000124718	0.000571057
GO:0022600	digestive system process	37/4924	0.000125538	0.000574395
GO:0051928	positive regulation of calcium ion transport	43/4924	0.000126233	0.000577091
GO:0003356	regulation of cilium beat frequency	Oct-24	0.000126768	0.000577091
GO:0031268	pseudopodium organization	Oct-24	0.000126768	0.000577091
GO:0051895	negative regulation of focal adhesion assembly	Oct-24	0.000126768	0.000577091
GO:0090136	epithelial cell-cell adhesion	Oct-24	0.000126768	0.000577091
GO:0150118	negative regulation of cell-substrate junction organization	Oct-24	0.000126768	0.000577091

GO:2001044	regulation of integrin-mediated signaling pathway	Oct-24	0.000126768	0.000577091
GO:0034502	protein localization to chromosome	32/4924	0.000127026	0.000577434
GO:1901222	regulation of NIK/NF-kappaB signaling	32/4924	0.000127026	0.000577434
GO:0016202	regulation of striated muscle tissue development	Dec-24	0.00012753	0.000579307
GO:0031663	lipopolysaccharide-mediated signaling pathway	23/4924	0.000130928	0.000593084
GO:0050982	detection of mechanical stimulus	23/4924	0.000130928	0.000593084
GO:0021954	central nervous system neuron development	31/4924	0.000130939	0.000593084
GO:0048644	muscle organ morphogenesis	31/4924	0.000130939	0.000593084
GO:0010766	negative regulation of sodium ion transport	Nov-24	0.00013193	0.000595856
GO:0045019	negative regulation of nitric oxide biosynthetic process	Nov-24	0.00013193	0.000595856
GO:1902430	negative regulation of amyloid-beta formation	Nov-24	0.00013193	0.000595856
GO:1904406	negative regulation of nitric oxide metabolic process	Nov-24	0.00013193	0.000595856
GO:0046634	regulation of alpha-beta T cell activation	36/4924	0.00013205	0.000595974
GO:0003016	respiratory system process	19/4924	0.000132646	0.000596955
GO:0006024	glycosaminoglycan biosynthetic process	19/4924	0.000132646	0.000596955
GO:0043114	regulation of vascular permeability	19/4924	0.000132646	0.000596955
GO:2000404	regulation of T cell migration	19/4924	0.000132646	0.000596955
GO:0006486	protein glycosylation	53/4924	0.000133619	0.000600477

GO:0043413	macromolecule glycosylation	53/4924	0.000133619	0.000600477
GO:0031016	pancreas development	30/4924	0.000134336	0.000603267
GO:0051224	negative regulation of protein transport	42/4924	0.000134771	0.000604789
GO:0002294	CD4-positive, alpha-beta T cell differentiation involved in immune response	25/4924	0.000138445	0.000620836
GO:0021549	cerebellum development	35/4924	0.000138557	0.000620897
GO:0007188	adenylate cyclase-modulating G protein-coupled receptor signaling pathway	61/4924	0.000139646	0.000624887
GO:0048545	response to steroid hormone	61/4924	0.000139646	0.000624887
GO:0006919	activation of cysteine-type endopeptidase activity involved in apoptotic process	27/4924	0.000140025	0.000625698
GO:0044773	mitotic DNA damage checkpoint signaling	27/4924	0.000140025	0.000625698
GO:0060706	cell differentiation involved in embryonic placenta development	15/4924	0.000140548	0.000627146
GO:1902656	calcium ion import into cytosol	15/4924	0.000140548	0.000627146
GO:0001782	B cell homeostasis	17/4924	0.000142952	0.000636972
GO:1905332	positive regulation of morphogenesis of an epithelium	17/4924	0.000142952	0.000636972
GO:0009581	detection of external stimulus	41/4924	0.000143716	0.000639045
GO:0016079	synaptic vesicle exocytosis	41/4924	0.000143716	0.000639045
GO:0046620	regulation of organ growth	38/4924	0.000143722	0.000639045
GO:0060538	skeletal muscle organ development	59/4924	0.000144284	0.000641091
GO:0006643	membrane lipid metabolic process	52/4924	0.000144543	0.00064179

GO:0006821	chloride transport	34/4924	0.000144987	0.000643311
GO:0006687	glycosphingolipid metabolic process	20/4924	0.000146621	0.000649189
GO:0043268	positive regulation of potassium ion transport	20/4924	0.000146621	0.000649189
GO:0045740	positive regulation of DNA replication	20/4924	0.000146621	0.000649189
GO:1904950	negative regulation of establishment of protein localization	43/4924	0.000149696	0.000662337
GO:1903531	negative regulation of secretion by cell	49/4924	0.000156874	0.00069361
GO:1903321	negative regulation of protein modification by small protein conjugation or removal	32/4924	0.000157256	0.000694813
GO:0007062	sister chromatid cohesion	21/4924	0.000157993	0.000695633
GO:0030838	positive regulation of actin filament polymerization	21/4924	0.000157993	0.000695633
GO:0042491	inner ear auditory receptor cell differentiation	21/4924	0.000157993	0.000695633
GO:0045687	positive regulation of glial cell differentiation	21/4924	0.000157993	0.000695633
GO:0048713	regulation of oligodendrocyte differentiation	21/4924	0.000157993	0.000695633
GO:0002831	regulation of response to biotic stimulus	91/4924	0.000159519	0.00070186
GO:0090087	regulation of peptide transport	64/4924	0.00015986	0.000702872
GO:0043500	muscle adaptation	39/4924	0.000162784	0.000715228
GO:0006688	glycosphingolipid biosynthetic process	14/4924	0.000165421	0.000723797
GO:0046885	regulation of hormone biosynthetic process	14/4924	0.000165421	0.000723797
GO:0060563	neuroepithelial cell differentiation	14/4924	0.000165421	0.000723797

GO:0090025	regulation of monocyte chemotaxis	14/4924	0.000165421	0.000723797
GO:0140058	neuron projection arborization	14/4924	0.000165421	0.000723797
GO:0021955	central nervous system neuron axonogenesis	18/4924	0.000165423	0.000723797
GO:0045071	negative regulation of viral genome replication	22/4924	0.000166786	0.000728244
GO:0045778	positive regulation of ossification	22/4924	0.000166786	0.000728244
GO:0051155	positive regulation of striated muscle cell differentiation	22/4924	0.000166786	0.000728244
GO:0043266	regulation of potassium ion transport	35/4924	0.000168777	0.00073643
GO:0002703	regulation of leukocyte mediated immunity	77/4924	0.000169689	0.000739896
GO:0045995	regulation of embryonic development	29/4924	0.000172379	0.000751105
GO:0010658	striated muscle cell apoptotic process	23/4924	0.000173131	0.00075334
GO:0043407	negative regulation of MAP kinase activity	23/4924	0.000173131	0.00075334
GO:0090313	regulation of protein targeting to membrane	16/4924	0.000174754	0.000759354
GO:1904707	positive regulation of vascular associated smooth muscle cell proliferation	16/4924	0.000174754	0.000759354
GO:0033209	tumor necrosis factor-mediated signaling pathway	28/4924	0.000175929	0.000763934
GO:0010469	regulation of signaling receptor activity	43/4924	0.000177025	0.000768164
GO:0002293	alpha-beta T cell differentiation involved in immune response	25/4924	0.000179308	0.000776723
GO:2000134	negative regulation of G1/S transition of mitotic cell cycle	25/4924	0.000179308	0.000776723
GO:0070085	glycosylation	58/4924	0.000179367	0.000776723

GO:0007492	endoderm development	26/4924	0.000179622	0.000777293
GO:0015749	monosaccharide transmembrane transport	37/4924	0.000183255	0.000792471
GO:0003179	heart valve morphogenesis	19/4924	0.000184605	0.000796668
GO:0007080	mitotic metaphase plate congression	19/4924	0.000184605	0.000796668
GO:0030195	negative regulation of blood coagulation	19/4924	0.000184605	0.000796668
GO:0010770	positive regulation of cell morphogenesis involved in differentiation	33/4924	0.000185605	0.000797252
GO:0045921	positive regulation of exocytosis	33/4924	0.000185605	0.000797252
GO:0046849	bone remodeling	33/4924	0.000185605	0.000797252
GO:0034333	adherens junction assembly	Aug-24	0.000186004	0.000797252
GO:0035331	negative regulation of hippo signaling	Aug-24	0.000186004	0.000797252
GO:0051798	positive regulation of hair follicle development	Aug-24	0.000186004	0.000797252
GO:0060433	bronchus development	Aug-24	0.000186004	0.000797252
GO:0072176	nephric duct development	Aug-24	0.000186004	0.000797252
GO:0098911	regulation of ventricular cardiac muscle cell action potential	Aug-24	0.000186004	0.000797252
GO:1900272	negative regulation of long-term synaptic potentiation	Aug-24	0.000186004	0.000797252
GO:0008217	regulation of blood pressure	59/4924	0.000188433	0.000807114
GO:0090276	regulation of peptide hormone secretion	62/4924	0.000189495	0.00081111
GO:0035809	regulation of urine volume	13/4924	0.000191168	0.000815503

GO:0051016	barbed-end actin filament capping	13/4924	0.000191168	0.000815503
GO:0060716	labyrinthine layer blood vessel development	13/4924	0.000191168	0.000815503
GO:0072567	chemokine (C-X-C motif) ligand 2 production	13/4924	0.000191168	0.000815503
GO:2000341	regulation of chemokine (C-X-C motif) ligand 2 production	13/4924	0.000191168	0.000815503
GO:0002791	regulation of peptide secretion	63/4924	0.000197464	0.000841793
GO:0061028	establishment of endothelial barrier	20/4924	0.000200259	0.000852552
GO:0090279	regulation of calcium ion import	20/4924	0.000200259	0.000852552
GO:0035282	segmentation	35/4924	0.000204779	0.000871209
GO:0060603	mammary gland duct morphogenesis	17/4924	0.000205543	0.000872691
GO:0086065	cell communication involved in cardiac conduction	17/4924	0.000205543	0.000872691
GO:2000279	negative regulation of DNA biosynthetic process	17/4924	0.000205543	0.000872691
GO:0048799	animal organ maturation	15/4924	0.000211935	0.000898018
GO:0050654	chondroitin sulfate proteoglycan metabolic process	15/4924	0.000211935	0.000898018
GO:0060306	regulation of membrane repolarization	15/4924	0.000211935	0.000898018
GO:0033135	regulation of peptidyl-serine phosphorylation	47/4924	0.000212974	0.000901812
GO:0006473	protein acetylation	59/4924	0.000214816	0.000908746
GO:0034405	response to fluid shear stress	Dec-24	0.000215445	0.000908746
GO:0055064	chloride ion homeostasis	Dec-24	0.000215445	0.000908746

GO:0060972	left/right pattern formation	Dec-24	0.000215445	0.000908746
GO:0098901	regulation of cardiac muscle cell action potential	Dec-24	0.000215445	0.000908746
GO:0000077	DNA damage checkpoint signaling	34/4924	0.000215764	0.000908746
GO:0042310	vasoconstriction	34/4924	0.000215764	0.000908746
GO:0050886	endocrine process	34/4924	0.000215764	0.000908746
GO:0001776	leukocyte homeostasis	37/4924	0.000220229	0.000926312
GO:0042177	negative regulation of protein catabolic process	37/4924	0.000220229	0.000926312
GO:0002548	monocyte chemotaxis	22/4924	0.000221194	0.000928323
GO:0010659	cardiac muscle cell apoptotic process	22/4924	0.000221194	0.000928323
GO:1900024	regulation of substrate adhesion-dependent cell spreading	22/4924	0.000221194	0.000928323
GO:0048708	astrocyte differentiation	28/4924	0.000221296	0.000928323
GO:0071347	cellular response to interleukin-1	27/4924	0.000225889	0.000946963
GO:0010715	regulation of extracellular matrix disassembly	Sep-24	0.00022854	0.000954899
GO:0031269	pseudopodium assembly	Sep-24	0.00022854	0.000954899
GO:0032530	regulation of microvillus organization	Sep-24	0.00022854	0.000954899
GO:0090178	regulation of establishment of planar polarity involved in neural tube closure	Sep-24	0.00022854	0.000954899
GO:2000095	regulation of Wnt signaling pathway, planar cell polarity pathway	Sep-24	0.00022854	0.000954899
GO:0006305	DNA alkylation	26/4924	0.000229064	0.000955189

GO:0006306	DNA methylation	26/4924	0.000229064	0.000955189
GO:1904029	regulation of cyclin-dependent protein kinase activity	26/4924	0.000229064	0.000955189
GO:0048168	regulation of neuronal synaptic plasticity	25/4924	0.000230515	0.000959338
GO:1990868	response to chemokine	25/4924	0.000230515	0.000959338
GO:1990869	cellular response to chemokine	25/4924	0.000230515	0.000959338
GO:0097178	ruffle assembly	18/4924	0.000231948	0.000964029
GO:1902991	regulation of amyloid precursor protein catabolic process	18/4924	0.000231948	0.000964029
GO:0006268	DNA unwinding involved in DNA replication	Nov-24	0.000234277	0.000971148
GO:0007252	I-kappaB phosphorylation	Nov-24	0.000234277	0.000971148
GO:0090050	positive regulation of cell migration involved in sprouting angiogenesis	Nov-24	0.000234277	0.000971148
GO:1903909	regulation of receptor clustering	Nov-24	0.000234277	0.000971148
GO:0062207	regulation of pattern recognition receptor signaling pathway	32/4924	0.000237671	0.000984569
GO:0006978	DNA damage response, signal transduction by p53 class mediator resulting in transcription of p21 class mediator	Oct-24	0.000241492	0.000993871
GO:0010470	regulation of gastrulation	Oct-24	0.000241492	0.000993871
GO:0030033	microvillus assembly	Oct-24	0.000241492	0.000993871
GO:0035330	regulation of hippo signaling	Oct-24	0.000241492	0.000993871
GO:0060391	positive regulation of SMAD protein signal transduction	Oct-24	0.000241492	0.000993871
GO:0061318	renal filtration cell differentiation	Oct-24	0.000241492	0.000993871

GO:0072112	glomerular visceral epithelial cell differentiation	Oct-24	0.000241492	0.000993871
GO:0072311	glomerular epithelial cell differentiation	Oct-24	0.000241492	0.000993871
GO:1901881	positive regulation of protein depolymerization	Oct-24	0.000241492	0.000993871
GO:2000319	regulation of T-helper 17 cell differentiation	Oct-24	0.000241492	0.000993871
GO:0045598	regulation of fat cell differentiation	43/4924	0.00024555	0.00100991
GO:0089718	amino acid import across plasma membrane	19/4924	0.000253562	0.001040833
GO:1900047	negative regulation of hemostasis	19/4924	0.000253562	0.001040833
GO:0021799	cerebral cortex radially oriented cell migration	16/4924	0.00025422	0.001040833
GO:0070528	protein kinase C signaling	16/4924	0.00025422	0.001040833
GO:0072210	metanephric nephron development	16/4924	0.00025422	0.001040833
GO:0021952	central nervous system projection neuron axonogenesis	14/4924	0.000254389	0.001040833
GO:0099622	cardiac muscle cell membrane repolarization	14/4924	0.000254389	0.001040833
GO:1905523	positive regulation of macrophage migration	14/4924	0.000254389	0.001040833
GO:0018394	peptidyl-lysine acetylation	52/4924	0.00025683	0.001050137
GO:0009582	detection of abiotic stimulus	40/4924	0.000257546	0.001052382
GO:0070828	heterochromatin organization	30/4924	0.00025853	0.001055722
GO:0060349	bone morphogenesis	34/4924	0.000261608	0.001067597
GO:1901019	regulation of calcium ion transmembrane transporter activity	29/4924	0.000268094	0.001092653

GO:1904427	positive regulation of calcium ion transmembrane transport	29/4924	0.000268094	0.001092653
GO:0046942	carboxylic acid transport	73/4924	0.000268722	0.001094505
GO:0007131	reciprocal meiotic recombination	20/4924	0.000270367	0.001099076
GO:0060412	ventricular septum morphogenesis	20/4924	0.000270367	0.001099076
GO:0140527	reciprocal homologous recombination	20/4924	0.000270367	0.001099076
GO:0006304	DNA modification	33/4924	0.000275892	0.001120809
GO:0060998	regulation of dendritic spine development	28/4924	0.000276759	0.001122886
GO:1902117	positive regulation of organelle assembly	28/4924	0.000276759	0.001122886
GO:0050764	regulation of phagocytosis	36/4924	0.000280589	0.001137693
GO:1900271	regulation of long-term synaptic potentiation	21/4924	0.000282596	0.001144358
GO:1903533	regulation of protein targeting	21/4924	0.000282596	0.001144358
GO:0071887	leukocyte apoptotic process	41/4924	0.000283646	0.001147875
GO:0006475	internal protein amino acid acetylation	49/4924	0.00028424	0.001148804
GO:0018393	internal peptidyl-lysine acetylation	49/4924	0.00028424	0.001148804
GO:0055067	monovalent inorganic cation homeostasis	47/4924	0.000287908	0.001162881
GO:0097530	granulocyte migration	45/4924	0.000289317	0.001167824
GO:2001021	negative regulation of response to DNA damage stimulus	26/4924	0.000290184	0.001170575
GO:0140115	export across plasma membrane	22/4924	0.000290628	0.001171097

GO:2000249	regulation of actin cytoskeleton reorganization	17/4924	0.000290685	0.001171097
GO:0071621	granulocyte chemotaxis	38/4924	0.000294769	0.001186793
GO:1901224	positive regulation of NIK/NF-kappaB signaling	24/4924	0.000295965	0.00119085
GO:1903076	regulation of protein localization to plasma membrane	35/4924	0.000298014	0.001198328
GO:0015711	organic anion transport	86/4924	0.000300494	0.001207298
GO:0033688	regulation of osteoblast proliferation	13/4924	0.000301202	0.001207298
GO:0051767	nitric-oxide synthase biosynthetic process	13/4924	0.000301202	0.001207298
GO:0051769	regulation of nitric-oxide synthase biosynthetic process	13/4924	0.000301202	0.001207298
GO:0061484	hematopoietic stem cell homeostasis	13/4924	0.000301202	0.001207298
GO:0030148	sphingolipid biosynthetic process	31/4924	0.000304438	0.001218909
GO:0001959	regulation of cytokine-mediated signaling pathway	40/4924	0.000304486	0.001218909
GO:0097553	calcium ion transmembrane import into cytosol	44/4924	0.000312335	0.001248714
GO:0003149	membranous septum morphogenesis	Jul-24	0.000313713	0.001248714
GO:0006971	hypotonic response	Jul-24	0.000313713	0.001248714
GO:0032836	glomerular basement membrane development	Jul-24	0.000313713	0.001248714
GO:0048671	negative regulation of collateral sprouting	Jul-24	0.000313713	0.001248714
GO:0060484	lung-associated mesenchyme development	Jul-24	0.000313713	0.001248714
GO:0072203	cell proliferation involved in metanephros development	Jul-24	0.000313713	0.001248714

GO:0090091	positive regulation of extracellular matrix disassembly	Jul-24	0.000313713	0.001248714
GO:0099640	axo-dendritic protein transport	Jul-24	0.000313713	0.001248714
GO:0030890	positive regulation of B cell proliferation	18/4924	0.000320631	0.001275443
GO:0030073	insulin secretion	61/4924	0.00033398	0.001350842
GO:0019229	regulation of vasoconstriction	28/4924	0.000344189	0.001366569
GO:0032729	positive regulation of interferon-gamma production	28/4924	0.000344189	0.001366569
GO:0034219	carbohydrate transmembrane transport	38/4924	0.000349883	0.001385508
GO:0034114	regulation of heterotypic cell-cell adhesion	Dec-24	0.000350058	0.001385508
GO:0035024	negative regulation of Rho protein signal transduction	Dec-24	0.000350058	0.001385508
GO:0044331	cell-cell adhesion mediated by cadherin	Dec-24	0.000350058	0.001385508
GO:0045684	positive regulation of epidermis development	Dec-24	0.000350058	0.001385508
GO:0071241	cellular response to inorganic substance	62/4924	0.000351664	0.001390993
GO:0045132	meiotic chromosome segregation	32/4924	0.000352856	0.001394833
GO:0014855	striated muscle cell proliferation	27/4924	0.000355514	0.001404457
GO:0035825	homologous recombination	20/4924	0.00036102	0.001424426
GO:0048701	embryonic cranial skeleton morphogenesis	20/4924	0.00036102	0.001424426
GO:0090049	regulation of cell migration involved in sprouting angiogenesis	16/4924	0.00036312	0.001431814
GO:1903900	regulation of viral life cycle	42/4924	0.000363441	0.001432184

GO:0015849	organic acid transport	79/4924	0.000371026	0.00146116
GO:0006672	ceramide metabolic process	31/4924	0.0003711536	0.001462255
GO:0043666	regulation of phosphoprotein phosphatase activity	21/4924	0.0003722302	0.001462468
GO:0071103	DNA conformation change	21/4924	0.0003722302	0.001462468
GO:0034394	protein localization to cell surface	25/4924	0.0003722981	0.001462468
GO:0046824	positive regulation of nucleocytoplasmic transport	25/4924	0.0003722981	0.001462468
GO:0071867	response to monoamine	25/4924	0.0003722981	0.001462468
GO:0071869	response to catecholamine	25/4924	0.0003722981	0.001462468
GO:0022037	metencephalon development	37/4924	0.0003744465	0.001467373
GO:0006949	syncytium formation	24/4924	0.00037815	0.001477532
GO:0042246	tissue regeneration	24/4924	0.00037815	0.001477532
GO:0048488	synaptic vesicle endocytosis	24/4924	0.00037815	0.001477532
GO:0140238	presynaptic endocytosis	24/4924	0.00037815	0.001477532
GO:0042698	ovulation cycle	22/4924	0.0003778464	0.001477532
GO:0046850	regulation of bone remodeling	22/4924	0.0003778464	0.001477532
GO:0071346	cellular response to interferon-gamma	34/4924	0.0003800091	0.001481515
GO:0090311	regulation of protein deacetylation	23/4924	0.0003800189	0.001481515
GO:1903307	positive regulation of regulated secretory pathway	23/4924	0.0003800189	0.001481515

GO:0006026	aminoglycan catabolic process	14/4924	0.000381474	0.001482856
GO:0060074	synapse maturation	14/4924	0.000381474	0.001482856
GO:0060561	apoptotic process involved in morphogenesis	14/4924	0.000381474	0.001482856
GO:0060977	coronary vasculature morphogenesis	14/4924	0.000381474	0.001482856
GO:0035303	regulation of dephosphorylation	39/4924	0.000385653	0.001498178
GO:0061053	somite development	30/4924	0.000390092	0.001514489
GO:0017121	plasma membrane phospholipid scrambling	Nov-24	0.000396328	0.001531156
GO:0036158	outer dynein arm assembly	Nov-24	0.000396328	0.001531156
GO:0046325	negative regulation of glucose import	Nov-24	0.000396328	0.001531156
GO:0051770	positive regulation of nitric-oxide synthase biosynthetic process	Nov-24	0.000396328	0.001531156
GO:0060602	branch elongation of an epithelium	Nov-24	0.000396328	0.001531156
GO:0090026	positive regulation of monocyte chemotaxis	Nov-24	0.000396328	0.001531156
GO:2000810	regulation of bicellular tight junction assembly	Nov-24	0.000396328	0.001531156
GO:2001224	positive regulation of neuron migration	Nov-24	0.000396328	0.001531156
GO:0032924	activin receptor signaling pathway	17/4924	0.000404765	0.001560884
GO:0072538	T-helper 17 type immune response	17/4924	0.000404765	0.001560884
GO:0098801	regulation of renal system process	17/4924	0.000404765	0.001560884
GO:0006271	DNA strand elongation involved in DNA replication	Aug-24	0.000410924	0.001578839

GO:0043589	skin morphogenesis	Aug-24	0.000410924	0.001578839
GO:0045618	positive regulation of keratinocyte differentiation	Aug-24	0.000410924	0.001578839
GO:0051315	attachment of mitotic spindle microtubules to kinetochore	Aug-24	0.000410924	0.001578839
GO:0072110	glomerular mesangial cell proliferation	Aug-24	0.000410924	0.001578839
GO:1903651	positive regulation of cytoplasmic transport	Aug-24	0.000410924	0.001578839
GO:2001252	positive regulation of chromosome organization	32/4924	0.00042719	0.001640338
GO:0000212	meiotic spindle organization	Oct-24	0.000431786	0.001650948
GO:0010544	negative regulation of platelet activation	Oct-24	0.000431786	0.001650948
GO:0021756	striatum development	Oct-24	0.000431786	0.001650948
GO:0034111	negative regulation of homotypic cell-cell adhesion	Oct-24	0.000431786	0.001650948
GO:0042772	DNA damage response, signal transduction resulting in transcription	Oct-24	0.000431786	0.001650948
GO:0071636	positive regulation of transforming growth factor beta production	Oct-24	0.000431786	0.001650948
GO:1900117	regulation of execution phase of apoptosis	Oct-24	0.000431786	0.001650948
GO:0010543	regulation of platelet activation	18/4924	0.000437311	0.001670045
GO:0120163	negative regulation of cold-induced thermogenesis	18/4924	0.000437311	0.001670045
GO:0048469	cell maturation	58/4924	0.000440436	0.001680961
GO:0048681	negative regulation of axon regeneration	Sep-24	0.000443084	0.001684949
GO:0060026	convergent extension	Sep-24	0.000443084	0.001684949

GO:0090128	regulation of synapse maturation	Sep-24	0.000443084	0.001684949
GO:0090177	establishment of planar polarity involved in neural tube closure	Sep-24	0.000443084	0.001684949
GO:1901722	regulation of cell proliferation involved in kidney development	Sep-24	0.000443084	0.001684949
GO:1902043	positive regulation of extrinsic apoptotic signaling pathway via death domain receptors	Sep-24	0.000443084	0.001684949
GO:0008045	motor neuron axon guidance	15/4924	0.00045174	0.00171373
GO:0022616	DNA strand elongation	15/4924	0.00045174	0.00171373
GO:0034122	negative regulation of toll-like receptor signaling pathway	15/4924	0.00045174	0.00171373
GO:0051491	positive regulation of filopodium assembly	15/4924	0.00045174	0.00171373
GO:2000106	regulation of leukocyte apoptotic process	34/4924	0.000455541	0.001727111
GO:0090330	regulation of platelet aggregation	13/4924	0.000460911	0.001746286
GO:0032392	DNA geometric change	19/4924	0.000461152	0.001746286
GO:0002237	response to molecule of bacterial origin	96/4924	0.000464912	0.001759467
GO:0042982	amyloid precursor protein metabolic process	25/4924	0.000469661	0.001776373
GO:0002312	B cell activation involved in immune response	30/4924	0.000475773	0.001798411
GO:0010596	negative regulation of endothelial cell migration	20/4924	0.000477036	0.001801025
GO:0048546	digestive tract morphogenesis	20/4924	0.000477036	0.001801025
GO:0030888	regulation of B cell proliferation	24/4924	0.000479687	0.001808866
GO:1903556	negative regulation of tumor necrosis factor superfamily cytokine production	24/4924	0.000479687	0.001808866

GO:0048704	embryonic skeletal system morphogenesis	33/4924	0.000484819	0.001827125
GO:0043388	positive regulation of DNA binding	21/4924	0.000485877	0.001827183
GO:0045599	negative regulation of fat cell differentiation	21/4924	0.000485877	0.001827183
GO:0071622	regulation of granulocyte chemotaxis	21/4924	0.000485877	0.001827183
GO:0030330	DNA damage response, signal transduction by p53 class mediator	23/4924	0.000486283	0.001827183
GO:0060986	endocrine hormone secretion	23/4924	0.000486283	0.001827183
GO:0035315	hair cell differentiation	22/4924	0.000488642	0.001834956
GO:0031058	positive regulation of histone modification	35/4924	0.000508999	0.001908396
GO:0051897	positive regulation of protein kinase B signaling	35/4924	0.000508999	0.001908396
GO:0051251	positive regulation of lymphocyte activation	103/4924	0.000509107	0.001908396
GO:2001243	negative regulation of intrinsic apoptotic signaling pathway	31/4924	0.000546054	0.002045675
GO:0021801	cerebral cortex radial glia-guided migration	Dec-24	0.000549424	0.002054637
GO:0022030	telencephalon glial cell migration	Dec-24	0.000549424	0.002054637
GO:0070293	renal absorption	Dec-24	0.000549424	0.002054637
GO:0017145	stem cell division	17/4924	0.000555462	0.002074757
GO:0061098	positive regulation of protein tyrosine kinase activity	17/4924	0.000555462	0.002074757
GO:0042593	glucose homeostasis	72/4924	0.000557738	0.002082023
GO:0051642	centrosome localization	14/4924	0.000558998	0.002085492

GO:0009880	embryonic pattern specification	26/4924	0.000568162	0.002117176
GO:1904888	cranial skeletal system development	26/4924	0.000568162	0.002117176
GO:0048010	vascular endothelial growth factor receptor signaling pathway	18/4924	0.000588934	0.002193285
GO:0055008	cardiac muscle tissue morphogenesis	24/4924	0.000604272	0.002245101
GO:0071868	cellular response to monoamine stimulus	24/4924	0.000604272	0.002245101
GO:0071870	cellular response to catecholamine stimulus	24/4924	0.000604272	0.002245101
GO:1903317	regulation of protein maturation	24/4924	0.000604272	0.002245101
GO:0000768	syncytium formation by plasma membrane fusion	23/4924	0.000617314	0.002289509
GO:0060389	pathway-restricted SMAD protein phosphorylation	23/4924	0.000617314	0.002289509
GO:0140253	cell-cell fusion	23/4924	0.000617314	0.002289509
GO:0033500	carbohydrate homeostasis	72/4924	0.000618401	0.002291079
GO:0045833	negative regulation of lipid metabolic process	32/4924	0.000618464	0.002291079
GO:0007519	skeletal muscle tissue development	54/4924	0.00062259	0.00230501
GO:0043090	amino acid import	20/4924	0.000624058	0.002306381
GO:0061001	regulation of dendritic spine morphogenesis	20/4924	0.000624058	0.002306381
GO:0071470	cellular response to osmotic stress	20/4924	0.000624058	0.002306381
GO:0060393	regulation of pathway-restricted SMAD protein phosphorylation	22/4924	0.000625732	0.002311215
GO:0007095	mitotic G2 DNA damage checkpoint signaling	15/4924	0.000640594	0.002363338

GO:0045746	negative regulation of Notch signaling pathway	15/4924	0.000640594	0.002363338
GO:0006027	glycosaminoglycan catabolic process	Nov-24	0.000642726	0.002364286
GO:0072202	cell differentiation involved in metanephros development	Nov-24	0.000642726	0.002364286
GO:0099149	regulation of postsynaptic neurotransmitter receptor internalization	Nov-24	0.000642726	0.002364286
GO:1902894	negative regulation of miRNA transcription	Nov-24	0.000642726	0.002364286
GO:1902992	negative regulation of amyloid precursor protein catabolic process	Nov-24	0.000642726	0.002364286
GO:0031056	regulation of histone modification	52/4924	0.000652659	0.002399424
GO:0046530	photoreceptor cell differentiation	27/4924	0.000672348	0.002470368
GO:0010800	positive regulation of peptidyl-threonine phosphorylation	13/4924	0.000686873	0.002516406
GO:0045724	positive regulation of cilium assembly	13/4924	0.000686873	0.002516406
GO:0045932	negative regulation of muscle contraction	13/4924	0.000686873	0.002516406
GO:0060343	trabecula formation	13/4924	0.000686873	0.002516406
GO:0070977	bone maturation	13/4924	0.000686873	0.002516406
GO:0045739	positive regulation of DNA repair	33/4924	0.0006924	0.002535182
GO:0030879	mammary gland development	45/4924	0.000695077	0.00254203
GO:0050714	positive regulation of protein secretion	45/4924	0.000695077	0.00254203
GO:0120162	positive regulation of cold-induced thermogenesis	30/4924	0.00069814	0.002551751
GO:0071375	cellular response to peptide hormone stimulus	67/4924	0.000701594	0.002562804

GO:0036465	synaptic vesicle recycling	26/4924	0.000702383	0.002562804
GO:0045670	regulation of osteoclast differentiation	26/4924	0.000702383	0.002562804
GO:0035886	vascular associated smooth muscle cell differentiation	16/4924	0.000704686	0.002569718
GO:0010763	positive regulation of fibroblast migration	Oct-24	0.000731643	0.002661865
GO:0033599	regulation of mammary gland epithelial cell proliferation	Oct-24	0.000731643	0.002661865
GO:0034349	glial cell apoptotic process	Oct-24	0.000731643	0.002661865
GO:1904754	positive regulation of vascular associated smooth muscle cell migration	Oct-24	0.000731643	0.002661865
GO:0001678	cellular glucose homeostasis	46/4924	0.000733801	0.002664711
GO:0010968	regulation of microtubule nucleation	Jul-24	0.000734962	0.002664711
GO:0032342	aldosterone biosynthetic process	Jul-24	0.000734962	0.002664711
GO:0061517	macrophage proliferation	Jul-24	0.000734962	0.002664711
GO:0072178	nephric duct morphogenesis	Jul-24	0.000734962	0.002664711
GO:0120197	mucociliary clearance	Jul-24	0.000734962	0.002664711
GO:0030261	chromosome condensation	17/4924	0.000751882	0.002722926
GO:0042771	intrinsic apoptotic signaling pathway in response to DNA damage by p53 class mediator	17/4924	0.000751882	0.002722926
GO:0001541	ovarian follicle development	24/4924	0.000756128	0.00273516
GO:0043954	cellular component maintenance	24/4924	0.000756128	0.00273516
GO:0019751	polyol metabolic process	34/4924	0.000767305	0.0027774

GO:0030183	B cell differentiation	47/4924	0.000771775	0.002788561
GO:0032507	maintenance of protein location in cell	23/4924	0.000777988	0.002807795
GO:0070613	regulation of protein processing	23/4924	0.000777988	0.002807795
GO:0045665	negative regulation of neuron differentiation	28/4924	0.000780952	0.002815268
GO:0046888	negative regulation of hormone secretion	28/4924	0.000780952	0.002815268
GO:0033173	calcineurin-NFAT signaling cascade	18/4924	0.000783666	0.002821826
GO:1990573	potassium ion import across plasma membrane	18/4924	0.000783666	0.002821826
GO:0001704	formation of primary germ layer	31/4924	0.000789031	0.002839521
GO:0003229	ventricular cardiac muscle tissue development	22/4924	0.000794984	0.002852801
GO:0032613	interleukin-10 production	22/4924	0.000794984	0.002852801
GO:0060038	cardiac muscle cell proliferation	22/4924	0.000794984	0.002852801
GO:0098930	axonal transport	22/4924	0.000794984	0.002852801
GO:2000781	positive regulation of double-strand break repair	22/4924	0.000794984	0.002852801
GO:0045655	regulation of monocyte differentiation	Sep-24	0.00079894	0.002859866
GO:0070571	negative regulation of neuron projection regeneration	Sep-24	0.00079894	0.002859866
GO:0071498	cellular response to fluid shear stress	Sep-24	0.00079894	0.002859866
GO:1902018	negative regulation of cilium assembly	Sep-24	0.00079894	0.002859866
GO:0030850	prostate gland development	19/4924	0.000801921	0.002859866

GO:0097720	calcineurin-mediated signaling	19/4924	0.000801921	0.002859866
GO:0002092	positive regulation of receptor internalization	14/4924	0.000801942	0.002859866
GO:0060512	prostate gland morphogenesis	14/4924	0.000801942	0.002859866
GO:0061842	microtubule organizing center localization	14/4924	0.000801942	0.002859866
GO:0099563	modification of synaptic structure	14/4924	0.000801942	0.002859866
GO:1903959	regulation of anion transmembrane transport	14/4924	0.000801942	0.002859866
GO:0010656	negative regulation of muscle cell apoptotic process	21/4924	0.000805727	0.002870119
GO:0060760	positive regulation of response to cytokine stimulus	21/4924	0.000805727	0.002870119
GO:0052372	modulation by symbiont of entry into host	20/4924	0.000808632	0.002875593
GO:0062237	protein localization to postsynapse	20/4924	0.000808632	0.002875593
GO:0140353	lipid export from cell	20/4924	0.000808632	0.002875593
GO:0007144	female meiosis I	Aug-24	0.000815069	0.002882226
GO:0031915	positive regulation of synaptic plasticity	Aug-24	0.000815069	0.002882226
GO:0032341	aldosterone metabolic process	Aug-24	0.000815069	0.002882226
GO:0035791	platelet-derived growth factor receptor-beta signaling pathway	Aug-24	0.000815069	0.002882226
GO:0051988	regulation of attachment of spindle microtubules to kinetochore	Aug-24	0.000815069	0.002882226
GO:0060346	bone trabecula formation	Aug-24	0.000815069	0.002882226
GO:0060670	branching involved in labyrinthine layer morphogenesis	Aug-24	0.000815069	0.002882226

GO:0086069	bundle of His cell to Purkinje myocyte communication	Aug-24	0.000815069	0.002882226
GO:0090331	negative regulation of platelet aggregation	Aug-24	0.000815069	0.002882226
GO:1901201	regulation of extracellular matrix assembly	Aug-24	0.000815069	0.002882226
GO:0002027	regulation of heart rate	33/4924	0.000822859	0.002908142
GO:0006346	DNA methylation-dependent heterochromatin assembly	Dec-24	0.000836029	0.002949727
GO:0010165	response to X-ray	Dec-24	0.000836029	0.002949727
GO:0010829	negative regulation of glucose transmembrane transport	Dec-24	0.000836029	0.002949727
GO:1903825	organic acid transmembrane transport	39/4924	0.000848153	0.002990831
GO:0033138	positive regulation of peptidyl-serine phosphorylation	37/4924	0.000850447	0.002997243
GO:0050796	regulation of insulin secretion	50/4924	0.000880318	0.003100784
GO:0010737	protein kinase A signaling	15/4924	0.000892577	0.003136955
GO:0045622	regulation of T-helper cell differentiation	15/4924	0.000892577	0.003136955
GO:0060292	long-term synaptic depression	15/4924	0.000892577	0.003136955
GO:1902003	regulation of amyloid-beta formation	15/4924	0.000892577	0.003136955
GO:0002292	T cell differentiation involved in immune response	25/4924	0.000902915	0.003169753
GO:0032413	negative regulation of ion transmembrane transporter activity	25/4924	0.000902915	0.003169753
GO:0044070	regulation of anion transport	34/4924	0.000906568	0.003180809
GO:0035601	protein deacylation	38/4924	0.000917924	0.00321707

GO:0098732	macromolecule deacylation	38/4924	0.000917924	0.00321707
GO:0030032	lamellipodium assembly	24/4924	0.000940038	0.003292743
GO:0015914	phospholipid transport	28/4924	0.000946592	0.003312023
GO:0045814	negative regulation of gene expression, epigenetic	28/4924	0.000946592	0.003312023
GO:0060251	regulation of glial cell proliferation	16/4924	0.000959455	0.003353307
GO:1902275	regulation of chromatin organization	16/4924	0.000959455	0.003353307
GO:0032720	negative regulation of tumor necrosis factor production	23/4924	0.000973654	0.003390425
GO:0042509	regulation of tyrosine phosphorylation of STAT protein	23/4924	0.000973654	0.003390425
GO:0043462	regulation of ATP-dependent activity	23/4924	0.000973654	0.003390425
GO:0046324	regulation of glucose import	23/4924	0.000973654	0.003390425
GO:0048662	negative regulation of smooth muscle cell proliferation	23/4924	0.000973654	0.003390425
GO:0097194	execution phase of apoptosis	23/4924	0.000973654	0.003390425
GO:0045639	positive regulation of myeloid cell differentiation	33/4924	0.000974378	0.003390425
GO:0048477	oogenesis	33/4924	0.000974378	0.003390425
GO:0051648	vesicle localization	50/4924	0.000997662	0.003463386
GO:0010669	epithelial structure maintenance	13/4924	0.000999223	0.003463386
GO:0061036	positive regulation of cartilage development	13/4924	0.000999223	0.003463386
GO:2000352	negative regulation of endothelial cell apoptotic process	13/4924	0.000999223	0.003463386

GO:2000648	positive regulation of stem cell proliferation	13/4924	0.000999223	0.003463386
GO:0032930	positive regulation of superoxide anion generation	Nov-24	0.001004271	0.003463386
GO:0036303	lymph vessel morphogenesis	Nov-24	0.001004271	0.003463386
GO:0043371	negative regulation of CD4-positive, alpha-beta T cell differentiation	Nov-24	0.001004271	0.003463386
GO:0043586	tongue development	Nov-24	0.001004271	0.003463386
GO:0048485	sympathetic nervous system development	Nov-24	0.001004271	0.003463386
GO:0048670	regulation of collateral sprouting	Nov-24	0.001004271	0.003463386
GO:1900006	positive regulation of dendrite development	Nov-24	0.001004271	0.003463386
GO:1902547	regulation of cellular response to vascular endothelial growth factor stimulus	Nov-24	0.001004271	0.003463386
GO:1990776	response to angiotensin	Nov-24	0.001004271	0.003463386
GO:0034142	toll-like receptor 4 signaling pathway	17/4924	0.001004684	0.003463386
GO:1903170	negative regulation of calcium ion transmembrane transport	17/4924	0.001004684	0.003463386
GO:2001258	negative regulation of cation channel activity	17/4924	0.001004684	0.003463386
GO:0010972	negative regulation of G2/M transition of mitotic cell cycle	21/4924	0.00102457	0.003530006
GO:0010862	positive regulation of pathway-restricted SMAD protein phosphorylation	18/4924	0.001030993	0.003548261
GO:0021532	neural tube patterning	18/4924	0.001030993	0.003548261
GO:0048511	rhythmic process	74/4924	0.001038167	0.003571
GO:0006906	vesicle fusion	32/4924	0.001046137	0.003596453

GO:0045732	positive regulation of protein catabolic process	57/4924	0.001095119	0.003762794
GO:0070192	chromosome organization involved in meiotic cell cycle	25/4924	0.001109392	0.003809758
GO:0072331	signal transduction by p53 class mediator	41/4924	0.001115127	0.00382737
GO:0043243	positive regulation of protein-containing complex disassembly	14/4924	0.001128174	0.003861639
GO:0045987	positive regulation of smooth muscle contraction	14/4924	0.001128174	0.003861639
GO:0060479	lung cell differentiation	14/4924	0.001128174	0.003861639
GO:1990089	response to nerve growth factor	14/4924	0.001128174	0.003861639
GO:1990090	cellular response to nerve growth factor stimulus	14/4924	0.001128174	0.003861639
GO:0007260	tyrosine phosphorylation of STAT protein	24/4924	0.001161391	0.003966724
GO:0016445	somatic diversification of immunoglobulins	24/4924	0.001161391	0.003966724
GO:0042130	negative regulation of T cell proliferation	24/4924	0.001161391	0.003966724
GO:0048645	animal organ formation	24/4924	0.001161391	0.003966724
GO:0001946	lymphangiogenesis	Oct-24	0.001183795	0.004034501
GO:0046697	decidualization	Oct-24	0.001183795	0.004034501
GO:0048569	post-embryonic animal organ development	Oct-24	0.001183795	0.004034501
GO:0090036	regulation of protein kinase C signaling	Oct-24	0.001183795	0.004034501
GO:0042058	regulation of epidermal growth factor receptor signaling pathway	23/4924	0.001210347	0.004122765
GO:2000403	positive regulation of lymphocyte migration	15/4924	0.001223487	0.004165274

GO:0002052	positive regulation of neuroblast proliferation	Dec-24	0.001237149	0.004193669
GO:0032928	regulation of superoxide anion generation	Dec-24	0.001237149	0.004193669
GO:0033622	integrin activation	Dec-24	0.001237149	0.004193669
GO:0043931	ossification involved in bone maturation	Dec-24	0.001237149	0.004193669
GO:0046639	negative regulation of alpha-beta T cell differentiation	Dec-24	0.001237149	0.004193669
GO:0048634	regulation of muscle organ development	Dec-24	0.001237149	0.004193669
GO:0060039	pericardium development	Dec-24	0.001237149	0.004193669
GO:1900745	positive regulation of p38MAPK cascade	Dec-24	0.001237149	0.004193669
GO:0006865	amino acid transport	43/4924	0.00123991	0.004199638
GO:0021953	central nervous system neuron differentiation	53/4924	0.001240242	0.004199638
GO:0006611	protein export from nucleus	22/4924	0.001254696	0.004239473
GO:0042461	photoreceptor cell development	22/4924	0.001254696	0.004239473
GO:0060350	endochondral bone morphogenesis	22/4924	0.001254696	0.004239473
GO:0090398	cellular senescence	22/4924	0.001254696	0.004239473
GO:0007189	adenylate cyclase-activating G protein-coupled receptor signaling pathway	41/4924	0.001281979	0.00432934
GO:0031507	heterochromatin assembly	26/4924	0.001283755	0.004333015
GO:0045576	mast cell activation	29/4924	0.001284667	0.004333775
GO:0016572	histone phosphorylation	16/4924	0.001288161	0.004333966

GO:0034331	cell junction maintenance	16/4924	0.001288161	0.004333966
GO:0045601	regulation of endothelial cell differentiation	16/4924	0.001288161	0.004333966
GO:0046636	negative regulation of alpha-beta T cell activation	16/4924	0.001288161	0.004333966
GO:1902116	negative regulation of organelle assembly	16/4924	0.001288161	0.004333966
GO:0043434	response to peptide hormone	84/4924	0.001295995	0.004357998
GO:0003170	heart valve development	20/4924	0.001321563	0.004439241
GO:0010662	regulation of striated muscle cell apoptotic process	20/4924	0.001321563	0.004439241
GO:0045005	DNA-templated DNA replication maintenance of fidelity	17/4924	0.001326196	0.004450061
GO:0071300	cellular response to retinoic acid	17/4924	0.001326196	0.004450061
GO:0001961	positive regulation of cytokine-mediated signaling pathway	19/4924	0.001339055	0.004488433
GO:0042531	positive regulation of tyrosine phosphorylation of STAT protein	19/4924	0.001339055	0.004488433
GO:0042088	T-helper 1 type immune response	18/4924	0.001341816	0.004490525
GO:0044818	mitotic G2/M transition checkpoint	18/4924	0.001341816	0.004490525
GO:0061647	histone H3-K9 modification	18/4924	0.001341816	0.004490525
GO:0002286	T cell activation involved in immune response	35/4924	0.001354807	0.004524878
GO:0042249	establishment of planar polarity of embryonic epithelium	Sep-24	0.001356387	0.004524878
GO:0043217	myelin maintenance	Sep-24	0.001356387	0.004524878
GO:0046184	aldehyde biosynthetic process	Sep-24	0.001356387	0.004524878

GO:0051580	regulation of neurotransmitter uptake	Sep-24	0.001356387	0.004524878
GO:1904424	regulation of GTP binding	Sep-24	0.001356387	0.004524878
GO:0048814	regulation of dendrite morphogenesis	28/4924	0.001371358	0.004572401
GO:0009895	negative regulation of catabolic process	75/4924	0.001383615	0.004610831
GO:0002456	T cell mediated immunity	40/4924	0.001394579	0.004644914
GO:0016032	viral process	83/4924	0.001411595	0.004699106
GO:0045830	positive regulation of isotype switching	13/4924	0.001421893	0.004728395
GO:2000515	negative regulation of CD4-positive, alpha-beta T cell activation	13/4924	0.001421893	0.004728395
GO:0032456	endocytic recycling	24/4924	0.001426218	0.004740276
GO:0036473	cell death in response to oxidative stress	30/4924	0.001427922	0.004743438
GO:1905039	carboxylic acid transmembrane transport	38/4924	0.001430474	0.004749415
GO:0051607	defense response to virus	68/4924	0.001448251	0.004805907
GO:0010522	regulation of calcium ion transport into cytosol	32/4924	0.001457844	0.004830115
GO:0030518	intracellular steroid hormone receptor signaling pathway	32/4924	0.001457844	0.004830115
GO:0099175	regulation of postsynapse organization	32/4924	0.001457844	0.004830115
GO:0034644	cellular response to UV	27/4924	0.001461061	0.00483569
GO:0046323	glucose import	27/4924	0.001461061	0.00483569
GO:0008637	apoptotic mitochondrial changes	34/4924	0.001465558	0.00484803

GO:0001977	renal system process involved in regulation of blood volume	Aug-24	0.001485388	0.004887981
GO:0010172	embryonic body morphogenesis	Aug-24	0.001485388	0.004887981
GO:0030320	cellular monovalent inorganic anion homeostasis	Aug-24	0.001485388	0.004887981
GO:0033690	positive regulation of osteoblast proliferation	Aug-24	0.001485388	0.004887981
GO:0034350	regulation of glial cell apoptotic process	Aug-24	0.001485388	0.004887981
GO:0061577	calcium ion transmembrane transport via high voltage-gated calcium channel	Aug-24	0.001485388	0.004887981
GO:0072074	kidney mesenchyme development	Aug-24	0.001485388	0.004887981
GO:0086014	atrial cardiac muscle cell action potential	Aug-24	0.001485388	0.004887981
GO:0086026	atrial cardiac muscle cell to AV node cell signaling	Aug-24	0.001485388	0.004887981
GO:0086066	atrial cardiac muscle cell to AV node cell communication	Aug-24	0.001485388	0.004887981
GO:0071496	cellular response to external stimulus	72/4924	0.001493078	0.004904075
GO:0006705	mineralocorticoid biosynthetic process	Jul-24	0.001503502	0.004904075
GO:0031272	regulation of pseudopodium assembly	Jul-24	0.001503502	0.004904075
GO:0031274	positive regulation of pseudopodium assembly	Jul-24	0.001503502	0.004904075
GO:0033262	regulation of nuclear cell cycle DNA replication	Jul-24	0.001503502	0.004904075
GO:0048251	elastic fiber assembly	Jul-24	0.001503502	0.004904075
GO:0048934	peripheral nervous system neuron differentiation	Jul-24	0.001503502	0.004904075
GO:0048935	peripheral nervous system neuron development	Jul-24	0.001503502	0.004904075

GO:0060430	lung saccule development	Jul-24	0.001503502	0.004904075
GO:0060623	regulation of chromosome condensation	Jul-24	0.001503502	0.004904075
GO:0070316	regulation of G0 to G1 transition	Jul-24	0.001503502	0.004904075
GO:0072124	regulation of glomerular mesangial cell proliferation	Jul-24	0.001503502	0.004904075
GO:0072182	regulation of nephron tubule epithelial cell differentiation	Jul-24	0.001503502	0.004904075
GO:0072497	mesenchymal stem cell differentiation	Jul-24	0.001503502	0.004904075
GO:1902166	negative regulation of intrinsic apoptotic signaling pathway in response to DNA damage by p53 class mediator	Jul-24	0.001503502	0.004904075
GO:2000317	negative regulation of T-helper 17 type immune response	Jul-24	0.001503502	0.004904075
GO:2001225	regulation of chloride transport	Jul-24	0.001503502	0.004904075
GO:0007413	axonal fasciculation	Nov-24	0.001518296	0.004931919
GO:0010759	positive regulation of macrophage chemotaxis	Nov-24	0.001518296	0.004931919
GO:0010894	negative regulation of steroid biosynthetic process	Nov-24	0.001518296	0.004931919
GO:0030325	adrenal gland development	Nov-24	0.001518296	0.004931919
GO:0032288	myelin assembly	Nov-24	0.001518296	0.004931919
GO:0035988	chondrocyte proliferation	Nov-24	0.001518296	0.004931919
GO:0106030	neuron projection fasciculation	Nov-24	0.001518296	0.004931919
GO:1905820	positive regulation of chromosome separation	Nov-24	0.001518296	0.004931919
GO:0010466	negative regulation of peptidase activity	64/4924	0.001532003	0.004973883

GO:0002053	positive regulation of mesenchymal cell proliferation	14/4924	0.001558633	0.005049938
GO:0010573	vascular endothelial growth factor production	14/4924	0.001558633	0.005049938
GO:0045686	negative regulation of glial cell differentiation	14/4924	0.001558633	0.005049938
GO:0140718	facultative heterochromatin assembly	14/4924	0.001558633	0.005049938
GO:2000243	positive regulation of reproductive process	31/4924	0.001570629	0.005086192
GO:0006650	glycerophospholipid metabolic process	67/4924	0.001581253	0.005117967
GO:0051952	regulation of amine transport	33/4924	0.001584436	0.005125639
GO:0140546	defense response to symbiont	68/4924	0.001595067	0.005157384
GO:0050435	amyloid-beta metabolic process	21/4924	0.00161818	0.005226755
GO:1902750	negative regulation of cell cycle G2/M phase transition	21/4924	0.00161818	0.005226755
GO:0032088	negative regulation of NF-kappaB transcription factor activity	25/4924	0.001646933	0.005316904
GO:0070849	response to epidermal growth factor	15/4924	0.001651641	0.005329374
GO:0042987	amyloid precursor protein catabolic process	20/4924	0.001668146	0.005377126
GO:0055078	sodium ion homeostasis	20/4924	0.001668146	0.005377126
GO:0016571	histone methylation	44/4924	0.00168329	0.00542317
GO:0007140	male meiotic nuclear division	19/4924	0.001706216	0.005482252
GO:0010665	regulation of cardiac muscle cell apoptotic process	19/4924	0.001706216	0.005482252
GO:0048016	inositol phosphate-mediated signaling	19/4924	0.001706216	0.005482252

GO:1903539	protein localization to postsynaptic membrane	19/4924	0.001706216	0.005482252
GO:0048854	brain morphogenesis	16/4924	0.001706845	0.005482252
GO:1900026	positive regulation of substrate adhesion-dependent cell spreading	16/4924	0.001706845	0.005482252
GO:0032715	negative regulation of interleukin-6 production	18/4924	0.001728526	0.005546237
GO:0061756	leukocyte adhesion to vascular endothelial cell	18/4924	0.001728526	0.005546237
GO:0034205	amyloid-beta formation	17/4924	0.0017305	0.005549748
GO:0006664	glycolipid metabolic process	27/4924	0.001752536	0.005617559
GO:0033081	regulation of T cell differentiation in thymus	Dec-24	0.001785082	0.00571027
GO:0060444	branching involved in mammary gland duct morphogenesis	Dec-24	0.001785082	0.00571027
GO:0061311	cell surface receptor signaling pathway involved in heart development	Dec-24	0.001785082	0.00571027
GO:1903649	regulation of cytoplasmic transport	Dec-24	0.001785082	0.00571027
GO:0034968	histone lysine methylation	37/4924	0.001793567	0.005734503
GO:0019233	sensory perception of pain	40/4924	0.001833241	0.005853334
GO:0044851	hair cycle phase	Oct-24	0.001840017	0.005853334
GO:0046599	regulation of centriole replication	Oct-24	0.001840017	0.005853334
GO:0048791	calcium ion-regulated exocytosis of neurotransmitter	Oct-24	0.001840017	0.005853334
GO:0060009	Sertoli cell development	Oct-24	0.001840017	0.005853334
GO:0060080	inhibitory postsynaptic potential	Oct-24	0.001840017	0.005853334

GO:0060438	trachea development	Oct-24	0.001840017	0.005853334
GO:0099515	actin filament-based transport	Oct-24	0.001840017	0.005853334
GO:1900746	regulation of vascular endothelial growth factor signaling pathway	Oct-24	0.001840017	0.005853334
GO:1902254	negative regulation of intrinsic apoptotic signaling pathway by p53 class mediator	Oct-24	0.001840017	0.005853334
GO:0002200	somatic diversification of immune receptors	26/4924	0.00186981	0.00594511
GO:0031060	regulation of histone methylation	28/4924	0.001951547	0.006201866
GO:0010847	regulation of chromatin assembly	13/4924	0.001982775	0.006285267
GO:0044030	regulation of DNA methylation	13/4924	0.001982775	0.006285267
GO:0048566	embryonic digestive tract development	13/4924	0.001982775	0.006285267
GO:0060740	prostate gland epithelium morphogenesis	13/4924	0.001982775	0.006285267
GO:0060914	heart formation	13/4924	0.001982775	0.006285267
GO:0046928	regulation of neurotransmitter secretion	34/4924	0.001986659	0.006294413
GO:0090174	organelle membrane fusion	32/4924	0.002003181	0.006343574
GO:0002792	negative regulation of peptide secretion	21/4924	0.002011069	0.006362164
GO:0060999	positive regulation of dendritic spine development	21/4924	0.002011069	0.006362164
GO:0007586	digestion	37/4924	0.002065061	0.006529695
GO:0060420	regulation of heart growth	27/4924	0.002092543	0.006609963
GO:1903509	liposaccharide metabolic process	27/4924	0.002092543	0.006609963

GO:0010923	negative regulation of phosphatase activity	14/4924	0.002117439	0.006681908
GO:0046677	response to antibiotic	14/4924	0.002117439	0.006681908
GO:1901861	regulation of muscle tissue development	19/4924	0.002155008	0.006797061
GO:0009743	response to carbohydrate	53/4924	0.002176639	0.006861855
GO:0014072	response to isoquinoline alkaloid	Sep-24	0.002188527	0.006868425
GO:0032570	response to progesterone	Sep-24	0.002188527	0.006868425
GO:0032823	regulation of natural killer cell differentiation	Sep-24	0.002188527	0.006868425
GO:0033623	regulation of integrin activation	Sep-24	0.002188527	0.006868425
GO:0035457	cellular response to interferon-alpha	Sep-24	0.002188527	0.006868425
GO:0045837	negative regulation of membrane potential	Sep-24	0.002188527	0.006868425
GO:0055003	cardiac myofibril assembly	Sep-24	0.002188527	0.006868425
GO:0070314	G1 to G0 transition	Sep-24	0.002188527	0.006868425
GO:0086011	membrane repolarization during action potential	Sep-24	0.002188527	0.006868425
GO:0010762	regulation of fibroblast migration	15/4924	0.002197961	0.006880907
GO:0030513	positive regulation of BMP signaling pathway	15/4924	0.002197961	0.006880907
GO:0032733	positive regulation of interleukin-10 production	15/4924	0.002197961	0.006880907
GO:0038179	neurotrophin signaling pathway	15/4924	0.002197961	0.006880907
GO:1901381	positive regulation of potassium ion transmembrane transport	15/4924	0.002197961	0.006880907

GO:0045907	positive regulation of vasoconstriction	18/4924	0.002205066	0.006899721
GO:0010742	macrophage derived foam cell differentiation	Nov-24	0.002228802	0.006953289
GO:0035020	regulation of Rac protein signal transduction	Nov-24	0.002228802	0.006953289
GO:0045737	positive regulation of cyclin-dependent protein serine/threonine kinase activity	Nov-24	0.002228802	0.006953289
GO:0045939	negative regulation of steroid metabolic process	Nov-24	0.002228802	0.006953289
GO:1902230	negative regulation of intrinsic apoptotic signaling pathway in response to DNA damage	Nov-24	0.002228802	0.006953289
GO:1902473	regulation of protein localization to synapse	Nov-24	0.002228802	0.006953289
GO:0001569	branching involved in blood vessel morphogenesis	16/4924	0.002233722	0.006958308
GO:0006509	membrane protein ectodomain proteolysis	16/4924	0.002233722	0.006958308
GO:1905521	regulation of macrophage migration	16/4924	0.002233722	0.006958308
GO:0032623	interleukin-2 production	23/4924	0.00223808	0.006965002
GO:0043966	histone H3 acetylation	23/4924	0.00223808	0.006965002
GO:0032410	negative regulation of transporter activity	26/4924	0.002240089	0.006967814
GO:0055021	regulation of cardiac muscle tissue growth	25/4924	0.002393575	0.007441562
GO:0048515	spermatid differentiation	62/4924	0.002456046	0.007632019
GO:0001836	release of cytochrome c from mitochondria	21/4924	0.00248179	0.007708221
GO:0090314	positive regulation of protein targeting to membrane	Dec-24	0.002517221	0.007804651
GO:1903523	negative regulation of blood circulation	Dec-24	0.002517221	0.007804651

GO:0002024	diet induced thermogenesis	Aug-24	0.002527692	0.007804651
GO:0008212	mineralocorticoid metabolic process	Aug-24	0.002527692	0.007804651
GO:0031115	negative regulation of microtubule polymerization	Aug-24	0.002527692	0.007804651
GO:0042048	olfactory behavior	Aug-24	0.002527692	0.007804651
GO:0042640	anagen	Aug-24	0.002527692	0.007804651
GO:0048715	negative regulation of oligodendrocyte differentiation	Aug-24	0.002527692	0.007804651
GO:0055057	neuroblast division	Aug-24	0.002527692	0.007804651
GO:0072109	glomerular mesangium development	Aug-24	0.002527692	0.007804651
GO:0086103	G protein-coupled receptor signaling pathway involved in heart process	Aug-24	0.002527692	0.007804651
GO:2001212	regulation of vasculogenesis	Aug-24	0.002527692	0.007804651
GO:0055006	cardiac cell development	31/4924	0.002531692	0.007813176
GO:0071322	cellular response to carbohydrate stimulus	42/4924	0.002580102	0.007958682
GO:0046467	membrane lipid biosynthetic process	36/4924	0.002583403	0.007964967
GO:0046622	positive regulation of organ growth	20/4924	0.002595632	0.007994851
GO:0060612	adipose tissue development	20/4924	0.002595632	0.007994851
GO:0007286	spermatid development	60/4924	0.002659562	0.008187762
GO:0015872	dopamine transport	19/4924	0.002699055	0.008301239
GO:0051055	negative regulation of lipid biosynthetic process	19/4924	0.002699055	0.008301239

GO:0031294	lymphocyte costimulation	13/4924	0.002713767	0.00831 57
GO:0035335	peptidyl-tyrosine dephosphorylation	13/4924	0.002713767	0.00831 57
GO:0055075	potassium ion homeostasis	13/4924	0.002713767	0.00831 57
GO:0060487	lung epithelial cell differentiation	13/4924	0.002713767	0.00831 57
GO:0090322	regulation of superoxide metabolic process	13/4924	0.002713767	0.00831 57
GO:2000406	positive regulation of T cell migration	13/4924	0.002713767	0.00831 57
GO:0010611	regulation of cardiac muscle hypertrophy	23/4924	0.002714308	0.00831 57
GO:0071479	cellular response to ionizing radiation	23/4924	0.002714308	0.00831 57
GO:0051781	positive regulation of cell division	28/4924	0.002730555	0.008361413
GO:0002043	blood vessel endothelial cell proliferation involved in sprouting angiogenesis	Oct-24	0.002761011	0.008387501
GO:0021535	cell migration in hindbrain	Oct-24	0.002761011	0.008387501
GO:0031649	heat generation	Oct-24	0.002761011	0.008387501
GO:0033630	positive regulation of cell adhesion mediated by integrin	Oct-24	0.002761011	0.008387501
GO:0042481	regulation of odontogenesis	Oct-24	0.002761011	0.008387501
GO:0003093	regulation of glomerular filtration	Jul-24	0.002778176	0.008387501
GO:0030836	positive regulation of actin filament depolymerization	Jul-24	0.002778176	0.008387501
GO:0031652	positive regulation of heat generation	Jul-24	0.002778176	0.008387501
GO:0035810	positive regulation of urine volume	Jul-24	0.002778176	0.008387501

GO:0040034	regulation of development, heterochronic	Jul-24	0.002778176	0.008387501
GO:0045023	G0 to G1 transition	Jul-24	0.002778176	0.008387501
GO:0051639	actin filament network formation	Jul-24	0.002778176	0.008387501
GO:0051974	negative regulation of telomerase activity	Jul-24	0.002778176	0.008387501
GO:0060601	lateral sprouting from an epithelium	Jul-24	0.002778176	0.008387501
GO:0071679	commissural neuron axon guidance	Jul-24	0.002778176	0.008387501
GO:0090557	establishment of endothelial intestinal barrier	Jul-24	0.002778176	0.008387501
GO:0140059	dendrite arborization	Jul-24	0.002778176	0.008387501
GO:1905048	regulation of metallopeptidase activity	Jul-24	0.002778176	0.008387501
GO:2001223	negative regulation of neuron migration	Jul-24	0.002778176	0.008387501
GO:0002315	marginal zone B cell differentiation	Jun-24	0.002780902	0.008387501
GO:0010216	maintenance of DNA methylation	Jun-24	0.002780902	0.008387501
GO:0010692	regulation of alkaline phosphatase activity	Jun-24	0.002780902	0.008387501
GO:0021957	corticospinal tract morphogenesis	Jun-24	0.002780902	0.008387501
GO:0022027	interkinetic nuclear migration	Jun-24	0.002780902	0.008387501
GO:0036363	transforming growth factor beta activation	Jun-24	0.002780902	0.008387501
GO:0070673	response to interleukin-18	Jun-24	0.002780902	0.008387501
GO:0090269	fibroblast growth factor production	Jun-24	0.002780902	0.008387501

GO:0090270	regulation of fibroblast growth factor production	Jun-24	0.002780902	0.008387501
GO:0090306	meiotic spindle assembly	Jun-24	0.002780902	0.008387501
GO:1900119	positive regulation of execution phase of apoptosis	Jun-24	0.002780902	0.008387501
GO:1904526	regulation of microtubule binding	Jun-24	0.002780902	0.008387501
GO:0014014	negative regulation of gliogenesis	18/4924	0.002786965	0.008387501
GO:0031018	endocrine pancreas development	18/4924	0.002786965	0.008387501
GO:0044060	regulation of endocrine process	18/4924	0.002786965	0.008387501
GO:0045912	negative regulation of carbohydrate metabolic process	18/4924	0.002786965	0.008387501
GO:0048483	autonomic nervous system development	18/4924	0.002786965	0.008387501
GO:0006338	chromatin remodeling	56/4924	0.002814682	0.008466876
GO:0007212	dopamine receptor signaling pathway	14/4924	0.002831942	0.00850662
GO:1901385	regulation of voltage-gated calcium channel activity	14/4924	0.002831942	0.00850662
GO:1905168	positive regulation of double-strand break repair via homologous recombination	14/4924	0.002831942	0.00850662
GO:0035305	negative regulation of dephosphorylation	17/4924	0.002852982	0.008561661
GO:0043618	regulation of transcription from RNA polymerase II promoter in response to stress	17/4924	0.002852982	0.008561661
GO:2001259	positive regulation of cation channel activity	25/4924	0.002863837	0.00859015
GO:0002763	positive regulation of myeloid leukocyte differentiation	22/4924	0.002878426	0.008629804
GO:0014046	dopamine secretion	15/4924	0.00288602	0.008640246

GO:0014059	regulation of dopamine secretion	15/4924	0.00288602	0.008640246
GO:0045066	regulatory T cell differentiation	15/4924	0.00288602	0.008640246
GO:0002691	regulation of cellular extravasation	16/4924	0.002889209	0.008641584
GO:0015804	neutral amino acid transport	16/4924	0.002889209	0.008641584
GO:0007498	mesoderm development	33/4924	0.002893682	0.00865086
GO:0071482	cellular response to light stimulus	31/4924	0.002947808	0.008808498
GO:0040029	regulation of gene expression, epigenetic	43/4924	0.003011861	0.008995635
GO:0032608	interferon-beta production	21/4924	0.003041986	0.009072716
GO:0032648	regulation of interferon-beta production	21/4924	0.003041986	0.009072716
GO:0032663	regulation of interleukin-2 production	21/4924	0.003041986	0.009072716
GO:2000573	positive regulation of DNA biosynthetic process	24/4924	0.003064948	0.009136878
GO:0051650	establishment of vesicle localization	44/4924	0.00310057	0.009238701
GO:0050805	negative regulation of synaptic transmission	26/4924	0.003170457	0.00944248
GO:0003338	metanephros morphogenesis	Nov-24	0.003186333	0.009454038
GO:0006376	mRNA splice site selection	Nov-24	0.003186333	0.009454038
GO:0007274	neuromuscular synaptic transmission	Nov-24	0.003186333	0.009454038
GO:0031338	regulation of vesicle fusion	Nov-24	0.003186333	0.009454038
GO:0032069	regulation of nuclease activity	Nov-24	0.003186333	0.009454038

GO:0060008	Sertoli cell differentiation	Nov-24	0.003186333	0.009454038
GO:0062009	secondary palate development	Nov-24	0.003186333	0.009454038
GO:0086019	cell-cell signaling involved in cardiac conduction	Nov-24	0.003186333	0.009454038
GO:0010517	regulation of phospholipase activity	20/4924	0.003201724	0.009490772
GO:0032653	regulation of interleukin-10 production	20/4924	0.003201724	0.009490772
GO:0002698	negative regulation of immune effector process	35/4924	0.003229431	0.009568402
GO:0043484	regulation of RNA splicing	42/4924	0.003293232	0.009748275
GO:0061025	membrane fusion	42/4924	0.003293232	0.009748275
GO:0015837	amine transport	33/4924	0.003337942	0.009875985
GO:0009948	anterior/posterior axis specification	19/4924	0.003353363	0.00991231
GO:0050775	positive regulation of dendrite morphogenesis	19/4924	0.003353363	0.00991231
GO:0001956	positive regulation of neurotransmitter secretion	Sep-24	0.003380523	0.009941325
GO:0003159	morphogenesis of an endothelium	Sep-24	0.003380523	0.009941325
GO:0007063	regulation of sister chromatid cohesion	Sep-24	0.003380523	0.009941325
GO:0030050	vesicle transport along actin filament	Sep-24	0.003380523	0.009941325
GO:0032695	negative regulation of interleukin-12 production	Sep-24	0.003380523	0.009941325
GO:0036120	cellular response to platelet-derived growth factor stimulus	Sep-24	0.003380523	0.009941325
GO:0045623	negative regulation of T-helper cell differentiation	Sep-24	0.003380523	0.009941325

GO:0061154	endothelial tube morphogenesis	Sep-24	0.003380523	0.009941325
GO:0090030	regulation of steroid hormone biosynthetic process	Sep-24	0.003380523	0.009941325
GO:2000773	negative regulation of cellular senescence	Sep-24	0.003380523	0.009941325
GO:2001135	regulation of endocytic recycling	Sep-24	0.003380523	0.009941325
GO:0046887	positive regulation of hormone secretion	43/4924	0.00339102	0.009967542
