# Supplementary material for: Physiological reprogramming in vivo mediated by Sox4 pioneer factor activity
Source: bioRxiv. 2023 Feb 14:2023.02.14.528556. Preprint. [Version 1] doi: 10.1101/2023.02.14.528556 (PMC9948957; doi:10.1101/2023.02.14.528556)
Supplement: Supplement 6 [file media-6.pdf]

Table S4. PCR primers used for cloning by NEBuilder assembly.

Construct	Block Fw/Rv	Primer sequence
AAV-*HA*-*Sox4*-P2A-*Cre*	Block-1 Fw	GGTGTCCAGGCGGCCACCATGTACCCATACGATGTTCCAGATTACGCTATGGTACAACAGACCAACAACGC
	Block-1 Rv	CTTCAGCAGGCTGAAGTTAGTAGCTCCGCTTCCGTAGGTGAAGACCAGGTTAGAGATGC
	Block-2 Fw	CTAACTTCAGCCTGCTGAAGCAGGCTGGCGACGTGGAGGAGAACCCTGGACCTCCCAAGAAGAAGAGGAAGGTGTCC
	Block-2 Rv	TGTAATCCAGAGGTTGATTGTTAGTCACCATCTTCGAGCAGTCTC
AAV-*HA*-*Sox9*-P2A-*Cre*	Block-1 Fw	GTGTCCAGGCGGCCGCCATGTACCCATACGATGTTCCAGATTACGCTATGAATCTCCTGGACCCCTTCA
	Block-1 Rv	CTTCAGCAGGCTGAAGTTAGTAGCTCCGCTTCCGGGTCTGGTGAGCTGTGTGT
	Block-2 Fw (same as Sox4 block-2)	CTAACTTCAGCCTGCTGAAGCAGGCTGGCGACGTGGAGGAGAACCCTGGACCTCCCAAGAAGAAGAGGAAGGTGTCC
	Block-2 Rv (same as Sox4 block-2)	TGTAATCCAGAGGTTGATTGTTAGTCACCATCTTCGAGCAGTCTC
AAV-*FLAG*-*Sox4*-P2A-*Cre*	Block-1 Fw	GGTGTCCAGGCGGCCACCATGGACTACAAAGACGATGACGACAAGATGGTACAACAGACCAACAACGC
	Block-1 Rv (same as Sox4 block-1 Fw)	CTTCAGCAGGCTGAAGTTAGTAGCTCCGCTTCCGTAGGTGAAGACCAGGTTAGAGATGC
	Block-2 Fw (same as Sox4 block-2)	CTAACTTCAGCCTGCTGAAGCAGGCTGGCGACGTGGAGGAGAACCCTGGACCTCCCAAGAAGAAGAGGAAGGTGTCC
	Block-2 Rv (same as Sox4 block-2)	TGTAATCCAGAGGTTGATTGTTAGTCACCATCTTCGAGCAGTCTC
