# Supplementary material for: Physiological reprogramming in vivo mediated by Sox4 pioneer factor activity
Source: bioRxiv. 2023 Feb 14:2023.02.14.528556. Preprint. [Version 1] doi: 10.1101/2023.02.14.528556 (PMC9948957; doi:10.1101/2023.02.14.528556)
Supplement: Supplement 7 [file media-7.pdf]

Table S5. Antibodies used in this study.

Antibody	Host	Catalog #	Dilution	Manufacturer	Application
PE/Cy7-Cd11b	Rat	101216	1:100	BioLegend	Flow / FACS
PE/Cy7-Cd31	Rat	102418	1:100	BioLegend	Flow / FACS
PE/Cy7-Cd45	Rat	103114	1:100	BioLegend	Flow / FACS
BV421-Cd24	Rat	101826	1:100	BioLegend	Flow / FACS
BV421-Epcam	Rat	118225	1:100	BioLegend	Flow / FACS
PE/Dazzle594-Epcam	Rat	118236	1:100	BioLegend	Flow / FACS
Cd11b	Rat	101202	1:100	BioLegend	Dynabeads for NPC removal
Cd31	Rat	102402	1:100	BioLegend	Dynabeads for NPC removal
Cd45	Rat	103102	1:100	BioLegend	Dynabeads for NPC removal
Dynabeads™ anti-Rat IgG	Sheep	11035	1:10	Thermo	Dynabeads for NPC removal
MicroBeads-Epcam	Rat	130-105-958	1:11	Miltenyi	MACS for Epcam+ cell enrichment
GFP	Goat	ab6673	1:500	Abcam	Immunofluorescence
Cd24	Rat	101801	1:100	BioLegend	Immunofluorescence
Prom1	Rat	14-1331-80	1:100	eBioscience	Immunofluorescence
Itga6	Rat	N/A	1:100	V. Factor Lab	Immunofluorescence
Epcam	Rabbit	50591-R002	1:500	Sino Biological	Immunofluorescence
Krt19	Rabbit	N/A	1:1000	In-house	Immunofluorescence
HA-Tag	Rabbit	3724S	1:1000	CST	Immunofluorescence
Isotype control	Rabbit	3900S	1:1000	CST	Immunofluorescence
AlexaFluor488-anti-goat IgG	Donkey	A-11055	1:300	Invitrogen	Immunofluorescence
AlexaFluor594-anti-rabbit IgG	Donkey	A-21207	1:300	Invitrogen	Immunofluorescence
AlexaFluor594-anti-rat IgG	Donkey	A-21209	1:300	Invitrogen	Immunofluorescence
AlexaFluor-647-anti-rabbit IgG	Donkey	A-31573	1:300	Invitrogen	Immunofluorescence
HA-Tag	Rabbit	3724S	1:100	CST	CUT&RUN-seq
Isotype control	Rabbit	3900S	1:100	CST	CUT&RUN-seq
H3K27ac	Rabbit	ab4729	1:100	Abcam	CUT&RUN-seq
H3K27me3	Rabbit	9733	1:100	CST	CUT&RUN-seq
H3K4me1	Rabbit	ab8895	1:100	Abcam	CUT&RUN-seq
H3K4me3	Rabbit	ab8580	1:100	Abcam	CUT&RUN-seq
