# Supplementary material for: Physiological reprogramming in vivo mediated by Sox4 pioneer factor activity
Source: bioRxiv. 2023 Feb 14:2023.02.14.528556. Preprint. [Version 1] doi: 10.1101/2023.02.14.528556 (PMC9948957; doi:10.1101/2023.02.14.528556)
Supplement: Supplement 8 [file media-8.pdf]

Table S6. Primers used for qRT-PCR.

Target	Forward	Reverse
Ctgf	GGGCCTCTTCTGCGATTTC	ATCCAGGCAAGTGCATTGGTA
Cd44	TCGATTTGAATGTAACCTGCCG	CAGTCCGGGAGATACTGTAGC
Vim	CGTCCACACGCACCTACAG	GGGGGATGAGGAATAGAGGCT
Spp1	GCTTGGCTTATGGACTGAGG	CGCTCTTCATGTGAGAGGTG
Epcam	TCTACAAGGAAGAAATCAGCAAAA	CCCTCCTCAGTTCAGCACTC
Cftr	TGCACAGTCATCCTCTGTGA	AAGGGAGTCGTACTGCCAGA
Prom1	CTGCCCAAGCTGGAAGAATA	AGCCCAGGAAAAAGAAGGTC
Sox4	CCTCGCTCTCCTCGTCCT	TCGTCTTCGAACTCGTCGT
Cd24a	CTTCTGGCACTGCTCCTACC	TACTTGGATTTGGGGAAGCA
Itga6	TCATCCTCCTGGCTGTTCTT	GTATCGGGGAATGCTGTCAT
Krt7	CATTGAGATCGCCACCTACC	GATAAGCTTGCCACCATTGC
Krt19	TTGAGAGCCTGAAGGAGGAG	AATCCACCTCCACACTGACC
Cav1	GCGACCCCAAGCATCTCAA	ATGCCGTCGAAACTGTGTGT
Itga3	CCTCTTCGGCTACTCGGTC	CCAGTCCGGTTGGTATAGTCATC
Sox9	GACTCCCCACATTCCTCCTC	CCCTCTCGCTTCAGATCAAC
Hnf1b	TCTCACCAGCATGTCTTCCA	AAAATGGGGTCCTTGTTGCT
Asgr1	TTGGATTGGCCTAACTGACC	GCCCATGTCCGTACCAGTTA
Tdo2	GGGGATCCTCAGGCTATCAT	TACCCAGTGTCTGGGAACCA
Ttr	TGGACACCAAATCGTACTGG	CAGAGTCGTTGGCTGTGAAA
Fah	CGGCGATGAAGTCATCATAA	GAGCTTCAGGCTGGTGAAAG
Cebpa	CTCCCAGAGGACCAATGAAA	AAGTCTTAGCCGGAGGAAGC
Hnf4a	GCCTCAAAGCCATCATCTTC	CCGGTCGTTGATGTAATCCT
Alb	GCTGAGACCTTCACCTTCCA	CTTGTGCTTCACCAGCTCAG
G6pc	CTGTGCAGCTGAACGTCTGT	GAAAGTTTCAGCCACAGCAA
